# Supplementary material for: Multiscale detrended cross-correlation coefficient: estimating coupling in non-stationary neurophysiological signals
Source: Front Neurosci. 2024 Nov 13;18:1422085. doi: 10.3389/fnins.2024.1422085 (PMC11599215; doi:10.3389/fnins.2024.1422085)
Supplement: Supplementary file 1 [file Data_Sheet_1.zip › Additional Analysis.pdf]

Additional Analysis

MDC$_3$ Signal Integration

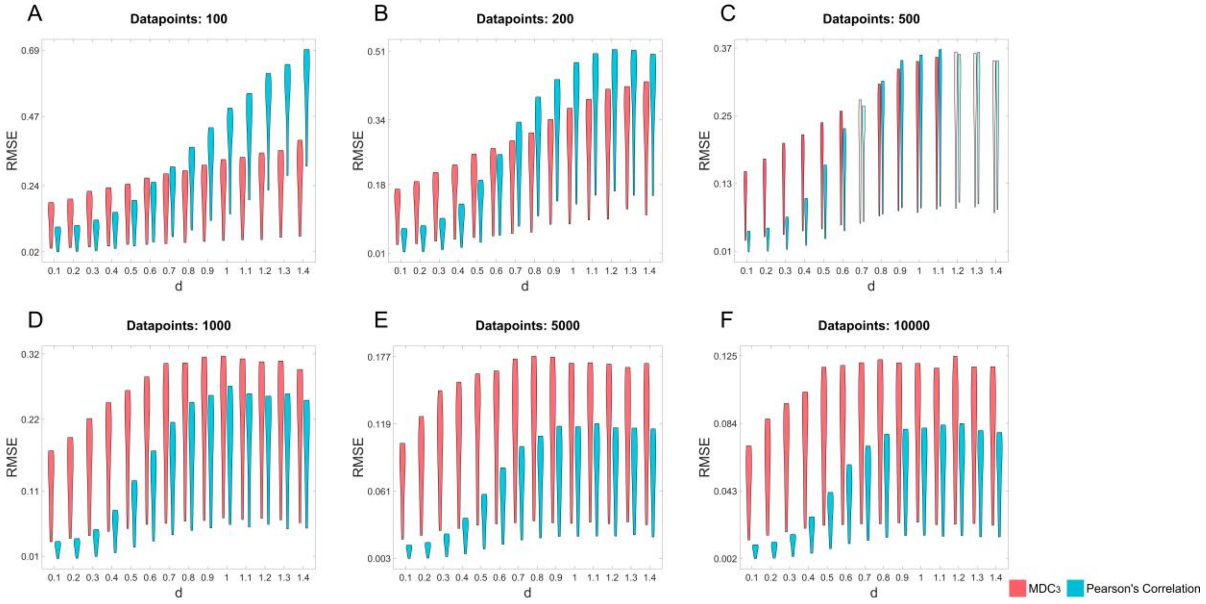

Figure S1 || *Root mean squared error (RMSE) of multiscale detrended cross-correlation coefficient (MDC$_3$) and Pearson's correlation for different levels of non-stationairity (d) and signal length (panels A-F).* Signals were integrated before the MDC$_3$ estimation. We simulated auto-regressive fractionally integrated moving-average (ARFIMA) processes with varying *d*, signal length and coupling strength (ρ). ρ was used to estimate the RMSE of MDC$_3$ and Pearson's correlation. Pairs of distributions whose difference was statistically significant (Benjamini-Hochberg adjusted $p<0.05$) are fully colored.

MDC$_3$ vs r_P

In the main analysis we constructed simulated functional magnetic resonance imaging (fMRI) signals using the The Virtual Brain (TVB) (Sanz Leon et al., 2013; Schirner et al., 2022). We used the regression dynamic causal modeling (rDCM) (Frässle et al., 2017, 2018, 2021) matrix as ground truth in our directed multiscale detrended cross-correlation coefficient (dMDC$_3$) vs lagged covariance (LG) comparisons. Here we repeated this analysis but using the structural connectivity (SC) matrix as ground truth. This time we decided to study the accuracy differences between the undirected multiscale detrended cross-correlation coefficient (MDC$_3$) and Pearson's correlation (r_P). We analyzed the first 5, 10, 15 and 20 minutes of the simulated fMRI. This resulted in 12 matrices (4 signal lengths x 3 metrics) for every simulated brain. Since the SC takes only positive values, we used the absolute values of MDC$_3$ and r_P. We also calculated the Z-scores of every SC, MDC$_3$ and r_P matrix, which we then used for the comparisons. We finally estimated the room mean squared error (RMSE) of MDC$_3$ and r_P for each simulation, using SC as ground truth. This resulted in 8 (2 FC estimators x 4 signal lengths) 100-point (100 simulated brains) distributions. We compared every pair of distributions using a paired t-test or Wilcoxon signed rank test, depending on the normality of the underlying distributions (evaluated using Lilliefors test). The 4 *p* values were adjusted using BH correction. MDC$_3$ was calculated for the frequencies between 0.011 to 0.17 Hz with increments of 0.01. 0.17 Hz was selected as the highest cutoff so each window during the estimation of MDC$_3$ had 8 datapoints. Second-degree polynomials were fitted for the detrending in MDC3. As seen in **Figure S2** r_P had a significantly ($p<0.05$ after BH correction) lower RMSE than MDC$_3$ in all signal lengths.

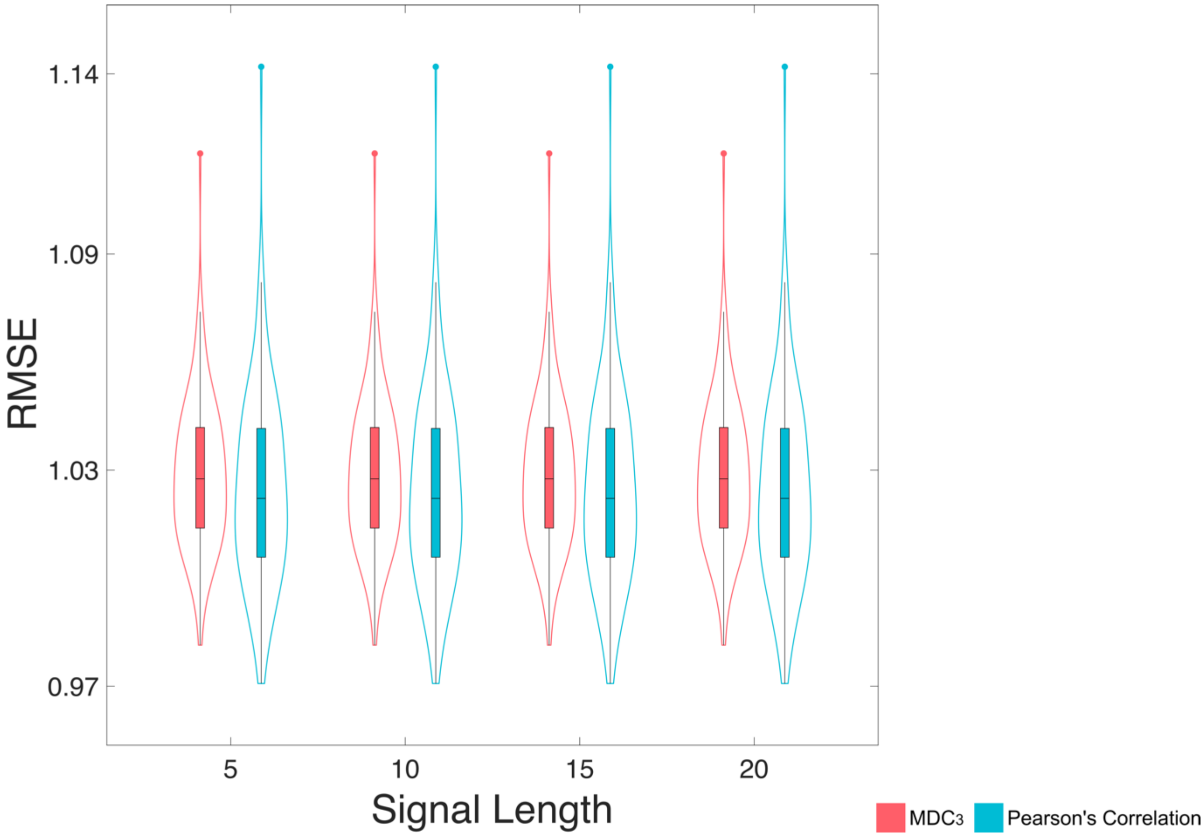

Figure S2 || *Root mean squared error (RMSE) of multiscale detrended cross-correlation coefficient (MDC₃) and Pearson's correlation, for four different signal lengths (5 minutes, 10 minutes, 15 minutes and 20 minutes).*

In a previous publication, Smith et al. (Smith et al., 2011) also used SC as ground truth for the comparison of different FC estimators. We used their simulations in tandem with our TVB analysis, except: *i*) simulations 26 and 27 whose length was not appropriate for MDC_3 and *ii*) simulation 13 due to the negative SC values. Their dataset consisted of several simulations, each with a different network architecture. For each simulation 50 different "subjects" were created, resulting to 50 fMRI datasets per simulation. Again, we decided to use both rDCM and SC as ground truth. MDC_3 and $dMDC_3$ were calculated for the frequencies between $fmin = 4 * SR/L$ (SR is the sampling rate and L is the length of the signal in datapoints) to $fmax = SR/8$ with increments of 0.01. The choice of $fmin$ and $fmax$ was made in order to achieve an adequate number of scales and datapoints per scale, respectively. Second degree polynomials were fitted for the detrending in MDC_3. When rDCM was used as ground truth, we used $dMDC_3$ and LG. Since rDCM and LG are not constrained between -1 and 1 as MDC_3, we calculated the Z-scores of every matrix; which then used for the comparisons. Using rDCM as our ground truth, we calculated the RMSE of MDC_3 and LG for each simulation. We then had 25 (25 simulations) pairs of RMSE distributions that we compared. Depending on the normality of the distributions (evaluated with Lilliefors test), we performed a paired t-test or Wilcoxon signed rank test, followed by BH correction. Results were considered significant when the when BH-adjusted $p<0.05$. As earlier, when using SC as ground truth we used the MDC_3 and r_P. Since SC does not have negative values, the absolute MDC_3 and r_P values were analyzed. The rest of the analysis remained the same. In most cases MDC_3 and $dMDC_3$ had a significantly lower RMSE, especially when rDCM was used as ground truth. The results of this analysis can be found in the supplementary files: *i*) **NetSim Matrices** (FC, EC and SC matrices) *ii*) **NetSim Violin Plots rDCM** (violin plots of RMSE

distributions when rDCM is used as ground truth) and *iii*) **NetSim Violin Plots SC** (violin plots of RMSE distributions when SC is used as ground truth).

Constrained vs Unconstrained Dipoles

As mentioned in the main body of the manuscript, one advantage of MDC_3 compared to several other FC estimators is the ability to differentiate between correlation and anticorrelation. We decided to explore this further by comparing two different source reconstruction methods for the magnetoencephalography (MEG) dataset. In the original study, we used constrained dipoles (Tadel et al., 2019), where the dipole orientations are considered to be normal to the cortical surface of the participant. This technique models the orientation of the macrocolumns of pyramidal neurons of the cortex and results in a more plausible phase and sign of the signals. Using unconstrained dipoles (Zhang et al., 2021) on the other hand does not have the same discriminatory power. This drawback of unconstrained dipoles originates because a three-dimensional source signal is estimated at each vertex, and principal component analysis is then used to reduce the signal to the one-dimensional time series of the principal component (Zhang et al., 2021). As a result, for every participant we had two MEG datasets, one for each source reconstruction pipeline (see **MEG Pipeline Specifications and PLV Computation** below).

We wanted to see if MDC_3 and r_P could capture more accurately the nuance of constrained dipoles compared to phase locking value (PLV) (Lachaux et al., 1999), a commonly used nonlinear FC estimator. PLV is reliable only within narrow ranges of frequency, so we used a finite impulse response filter with 8 and 12 Hz as lower and upper cutoffs, respectively. For every participant we analyzed multiple (ranging from 45 to 61) 4 seconds segments. We estimate the FC of each window using MDC_3, r_P and PLV. Then, we averaged the values obtained within each metric, so every participant had six connectivity matrices (MDC_3 constrained, MDC_3 unconstrained, r_P constrained, r_P unconstrained, PLV constrained and PLV unconstrained). Finally, we calculated the node strength of the brain regions based on every FC matrix and compared the two source reconstruction methods. This means that we had three groups of comparisons: *i)* constrained vs unconstrained dipoles using MDC_3 *ii)* constrained vs unconstrained dipoles using r_P and *iii)* constrained vs unconstrained dipoles using PLV. We employed a series of paired t-tests or Wilcoxon signed rank tests, depending on the normality of the distributions (Lilliefors test). The *p*-values of each comparison group were adjusted using BH correction. MDC_3 was calculated for the frequencies between 8 and 12 with increments of 0.5. Second-degree polynomials were fitted for the detrending in MDC_3 in both cases.

As seen in **Figure S3** more regions of interest (ROIs) had significantly different (BH-adjusted $p<0.05$) node strengths when MDC_3 and r_P were used [50 ROIs (74%) were different in MDC_3 and r_P, 33 ROIs (49%) were different in PLV].

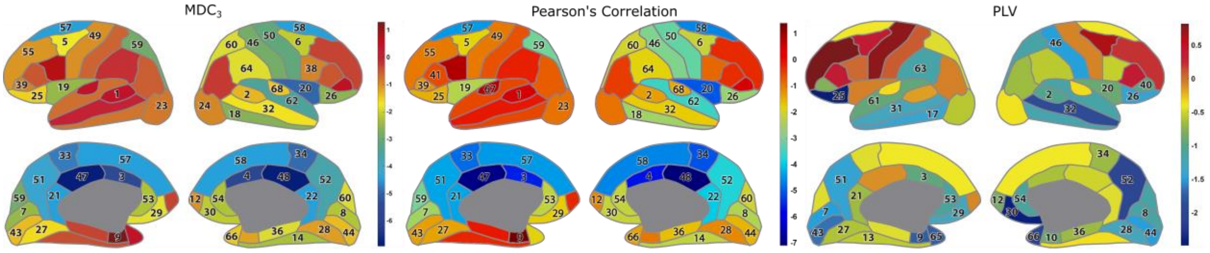

Figure S3 || *Difference between the node strengths calculated with MDC₃ (left), Pearson's correlation (middle) and PLV (right) using eyes closed resting-state magnetoencephalography: lateral view (up); medial view (down).* The colors represent the difference (constrained- unconstrained) in the node strengths while the numbers indicate the brain regions whose node strength was significantly different between the two preprocessing methods (BH-adjusted $p<0.05$). The numbers correspond to the regions of interest as defined in the Desikan-Killiany atlas (Desikan et al. 2006), list provided in **Table S2**.

Additionally, we decided to compare MDC_3 and r_P when unconstrained dipoles were used for source reconstruction. The same analysis as in the main body of the manuscript was followed except that we used unconstrained dipoles instead of constrained dipoles. In **Figure S4** we see that in unconstrained dipoles the significant differences are always due to higher estimation by MDC_3, while in constrained dipoles MDC_3 estimated both higher and lower values of node strength.

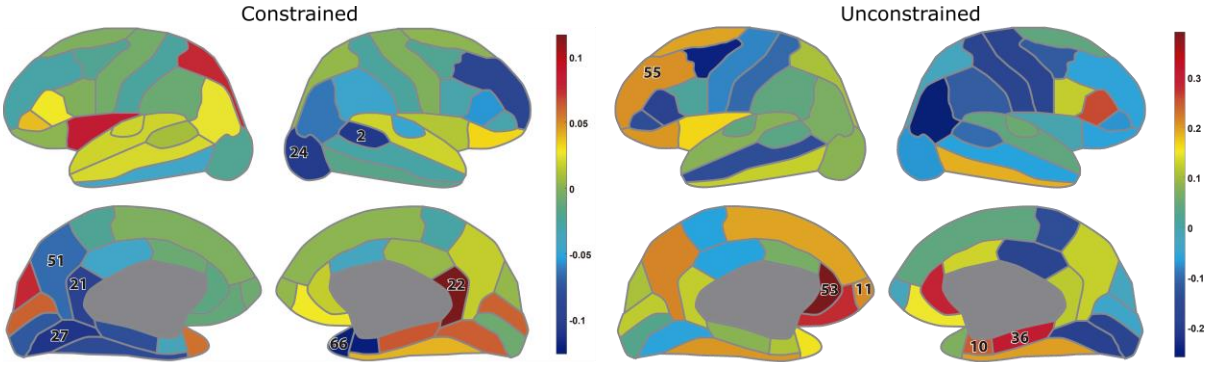

Figure S4 || *Difference between the node strengths calculated during eyes closed resting-state magnetoencephalography using constrained (left) and unconstrained (right) dipoles as source reconstruction.* The colors represent the difference (MDC_3- r_P) in the node strengths while the numbers indicate the brain regions whose node strength was significantly different between the two estimators (BH-adjusted $p<0.05$). The numbers correspond to the regions of interest as defined in the Desikan-Killiany atlas (Desikan et al. 2006), list provided in **Table S2**. lateral view (up); medial view (down).

Scale-Free Character of ARFIMA Processes

Several physiological time series have scale-free (i.e. fractal) characteristics (Eke et al., 2002). The scale-free character of these time series can be described using Hurst exponent (*H*). Traditionally, *H* analysis dichotomizes the signals into two categories: *i*) fractional Gaussian noise (fGn) and *ii*) fractional Brownian motion (fBm) (Eke et al., 2002). fGn corresponds to weakly-stationary signals, while fBM corresponds to non-stationary signals. In both cases *H* ranges between 0 and 1. $H<0.5$ indicates that the signal shows negative autocorrelation, i.e. a step to one direction is usually followed by another step in the opposite direction. $H=0.5$ indicates that the signal shows no autocorrelation. Finally, $H>0.5$ indicates that the signal shows positive autocorrelation, i.e. a step to one direction is usually followed by another step in the same direction. The main differences between fGn and fBm is that fGn's autocorrelation is only of short-term nature, while the fBm's autocorrelation is long-term. Further explanations can be found at Eke et al., 2002 and Ibe, 2013.

In the current analysis we decided to modify H's calculation so that it ranges between 0 and 2. Values between 0 and 1 correspond to weakly-stationary (i.e. fGn) signals, while values between 1 and 2 correspond to non-stationary (i.e. fBm) signals (Mukli et al., 2015). Under this definition, $H<0.5$ indicates that the signal shows negative short-term autocorrelation. $H=0.5$ or $H=1.5$ indicates that the signal shows no autocorrelation. $0.5<H<1$ indicates that the signal shows positive short-term autocorrelation. $1<H<1.5$ indicates that the signal shows negative long-term autocorrelation. Finally, $1.5<H<2$ indicates that the signal shows positive long-term autocorrelation.

We decided to calculate H for each of the auto-regressive fractionally integrated moving-average (ARFIMA) processes we used in our simulations. This was achieved by fitting a linear regression between the logarithm of power and the logarithm of frequency for every signal. The opposite value of this slope is termed spectral slope (β). Using β we estimated H, since $H = \frac{\beta+1}{2}$ (Eke et al., 2000, 2002)[1]. We then averaged the calculated H ending up with 14 values for every datapoint length, corresponding to the different levels of non-stationarity.

Finally, we explored the correlation between the H and the RMSE of MDC_3. When both distributions in question were normally distributed (evaluated using Lilliefors test) we calculated the Pearson's correlation between H and RMSE of MDC_3. When at least one of the distributions was not normally distributed, we used Spearman's correlation. This resulted in 6 p values, one for each signal length. The 6 p values were adjusted using the Benjamini-Hochberg (BH) correction. The same analysis was repeated for the calculation of the correlation between H and the RMSE of r_P. In all cases the RMSE of MDC_3 and r_P showed positive correlations with H that were statistically significant ($p<0.05$ after BH correction, **Figure S5** and **Table S1**).

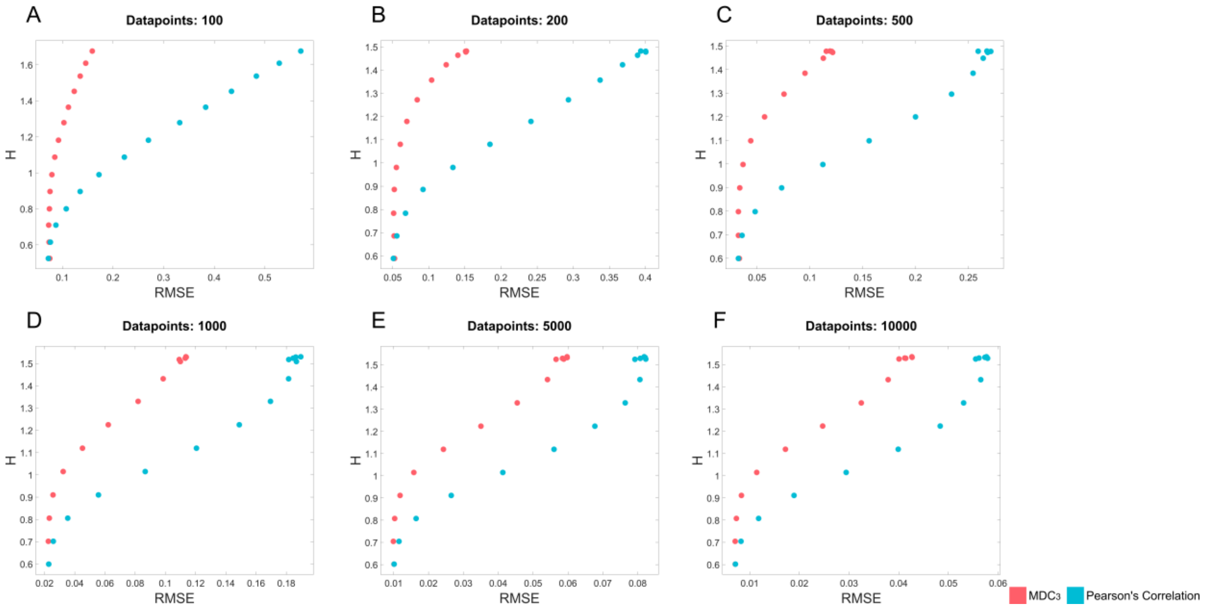

Figure S5 || *Root mean squared error (RMSE) of multiscale detrended cross-correlation coefficient (MDC_3) and Pearson's correlation for different levels of Hurst exponent (H) and signal length (panels A-F)***.**

[1] This is a modified formula that allows H to range between 0 and 2.

Table S1 || *The correlation between the Hurst exponent and the root mean squared error of multiscale detrended cross-correlation coefficient (MDC$_3$) and Pearson's correlation for different signal lengths of ARFIMA processes.* r: correlation coefficient, p: p-value after Benjamini Hochberg correction

	MDC$_3$		Pearson's Correlation	
Signal Length	r	p	r	p
100	0.93	$<10^{-4}$	0.98	$<10^{-4}$
200	0.90	$<10^{-4}$	0.99	$<10^{-4}$
500	0.93	$<10^{-4}$	0.99	$<10^{-4}$
1000	0.96	$<10^{-4}$	0.97	$<10^{-4}$
5000	0.97	$<10^{-4}$	0.96	$<10^{-4}$
10000	0.97	$<10^{-4}$	0.97	$<10^{-4}$

MEG Pipeline Specifications and PLV Computation

In the constrained case, a ROI time series was obtained by using the *mean* operator across all vertex-level constrained time series within that ROI. Finally, the PLV was computed for each pairwise combination of ROIs.

In the unconstrained case, a single-dimension time series was obtained at each vertex using PCA decomposition. Like the previous approach, we collapsed the vertex-level time series within each ROI using the *mean* operator, and subsequently applied the PLV to each pairwise combination of ROIs. Additionally, for the unconstrained case, we repeated the process by computing the PLV between each vertex pair directly, and then averaged the resulting PLV values across all vertex pairs within each pair of ROIs. This second variant produced similar results (although with a higher computational load), so we have reported the results of the previous variant for uniformity with the previous case.

Table S2 || *Numbered regions of interest in the Desikan-Killiany atlas (Desikan et al. 2006).*

1. L bankssts	2. R bankssts	3. L caudalanteriorcingulate	4. R caudalanteriorcingulate
5. L caudalmiddlefrontal	6. R caudalmiddlefrontal	7. L cuneus	8. R cuneus
9. L entorhinal	10. R entorhinal	11. L frontalpole	12. R frontalpole
13. L fusiform	14. R fusiform	15. L inferiorparietal	16. R inferiorparietal
17. L inferiortemporal	18. R inferiortemporal	19. L insula	20. R insula
21. L isthmuscingulate	22. R isthmuscingulate	23. L lateraloccipital	24. R lateraloccipital
25. L lateralorbitofrontal	26. R lateralorbitofrontal	27. L lingual	28. R lingual
29. L medialorbitofrontal	30. R medialorbitofrontal	31. L middletemporal	32. R middletemporal

33. L paracentral	34. R paracentral	35. L parahippocampal	36. R parahippocampal
37. L parsopercularis	38. R parsopercularis	39. L parsorbitalis	40. R parsorbitalis
41. L parstriangularis	42. R parstriangularis	43. L pericalcarine	44. R pericalcarine
45. L postcentral	46. R postcentral	47. L posteriorcingulate	48. R posteriorcingulate
49. L precentral	50. R precentral	51. L precuneus	52. R precuneus
53. L rostralanteriorcingulate	54. R rostralanteriorcingulate	55. L rostralmiddlefrontal	56. R rostralmiddlefrontal
57. L superiorfrontal	58. R superiorfrontal	59. L superiorparietal	60. R superiorparietal
61. L superiortemporal	62. R superiortemporal	63. L supramarginal	64. R supramarginal
65. L temporalpole	66. R temporalpole	67. L transversetemporal	68. R transversetemporal

References

Eke, A., Hermán, P., Bassingthwaighte, J., Raymond, G., Percival, D., Cannon, M., et al. (2000). Physiological time series: distinguishing fractal noises from motions. *Pflüg. Arch. - Eur. J. Physiol.* 439, 403–415. doi: 10.1007/s004249900135

Eke, A., Herman, P., Kocsis, L., and Kozak, L. R. (2002). Fractal characterization of complexity in temporal physiological signals. *Physiol. Meas.* 23, R1–R38. doi: 10.1088/0967-3334/23/1/201

Frässle, S., Harrison, S. J., Heinzle, J., Clementz, B. A., Tamminga, C. A., Sweeney, J. A., et al. (2021). Regression dynamic causal modeling for resting-state fMRI. *Hum. Brain Mapp.* 42, 2159–2180. doi: 10.1002/hbm.25357

Frässle, S., Lomakina, E. I., Kasper, L., Manjaly, Z. M., Leff, A., Pruessmann, K. P., et al. (2018). A generative model of whole-brain effective connectivity. *NeuroImage* 179, 505–529. doi: 10.1016/j.neuroimage.2018.05.058

Frässle, S., Lomakina, E. I., Razi, A., Friston, K. J., Buhmann, J. M., and Stephan, K. E. (2017). Regression DCM for fMRI. *NeuroImage* 155, 406–421. doi: 10.1016/j.neuroimage.2017.02.090

Ibe, O. C. (2013). “9 - Brownian Motion,” in *Markov Processes for Stochastic Modeling (Second Edition)*, ed. O. C. Ibe (Oxford: Elsevier), 263–293. doi: 10.1016/B978-0-12-407795-9.00009-8

Lachaux, J.-P., Rodriguez, E., Martinerie, J., and Varela, F. J. (1999). Measuring phase synchrony in brain signals. *Hum. Brain Mapp.* 8, 194–208. doi: 10.1002/(SICI)1097-0193(1999)8:4<194::AID-HBM4>3.0.CO;2-C

Mukli, P., Nagy, Z., and Eke, A. (2015). Multifractal formalism by enforcing the universal behavior of scaling functions. *Phys. Stat. Mech. Its Appl.* 417, 150–167. doi: 10.1016/j.physa.2014.09.002

Sanz Leon, P., Knock, S., Woodman, M., Domide, L., Mersmann, J., McIntosh, A., et al. (2013). The Virtual Brain: a simulator of primate brain network dynamics. *Front. Neuroinformatics* 7. Available at: https://www.frontiersin.org/articles/10.3389/fninf.2013.00010 (Accessed June 21, 2023).

Schirner, M., Domide, L., Perdikis, D., Triebkorn, P., Stefanovski, L., Pai, R., et al. (2022). Brain simulation as a cloud service: The Virtual Brain on EBRAINS. *NeuroImage* 251, 118973. doi: 10.1016/j.neuroimage.2022.118973

Smith, S. M., Miller, K. L., Salimi-Khorshidi, G., Webster, M., Beckmann, C. F., Nichols, T. E., et al. (2011). Network modelling methods for FMRI. *NeuroImage* 54, 875–891. doi: 10.1016/j.neuroimage.2010.08.063

Tadel, F., Bock, E., Niso, G., Mosher, J. C., Cousineau, M., Pantazis, D., et al. (2019). MEG/EEG Group Analysis With Brainstorm. *Front. Neurosci.* 13. doi: 10.3389/fnins.2019.00076

Zhang, Y., Wu, W., Toll, R. T., Naparstek, S., Maron-Katz, A., Watts, M., et al. (2021). Identification of psychiatric disorder subtypes from functional connectivity patterns in resting-state electroencephalography. *Nat. Biomed. Eng.* 5, 309–323. doi: 10.1038/s41551-020-00614-8
