# Supplementary material for: Multiscale detrended cross-correlation coefficient: estimating coupling in non-stationary neurophysiological signals
Source: Front Neurosci. 2024 Nov 13;18:1422085. doi: 10.3389/fnins.2024.1422085 (PMC11599215; doi:10.3389/fnins.2024.1422085)
Supplement: Supplementary file 1 [file Data_Sheet_1.zip › NetSim Matrices.zip!/NetSim Matrices/sim19.pdf]

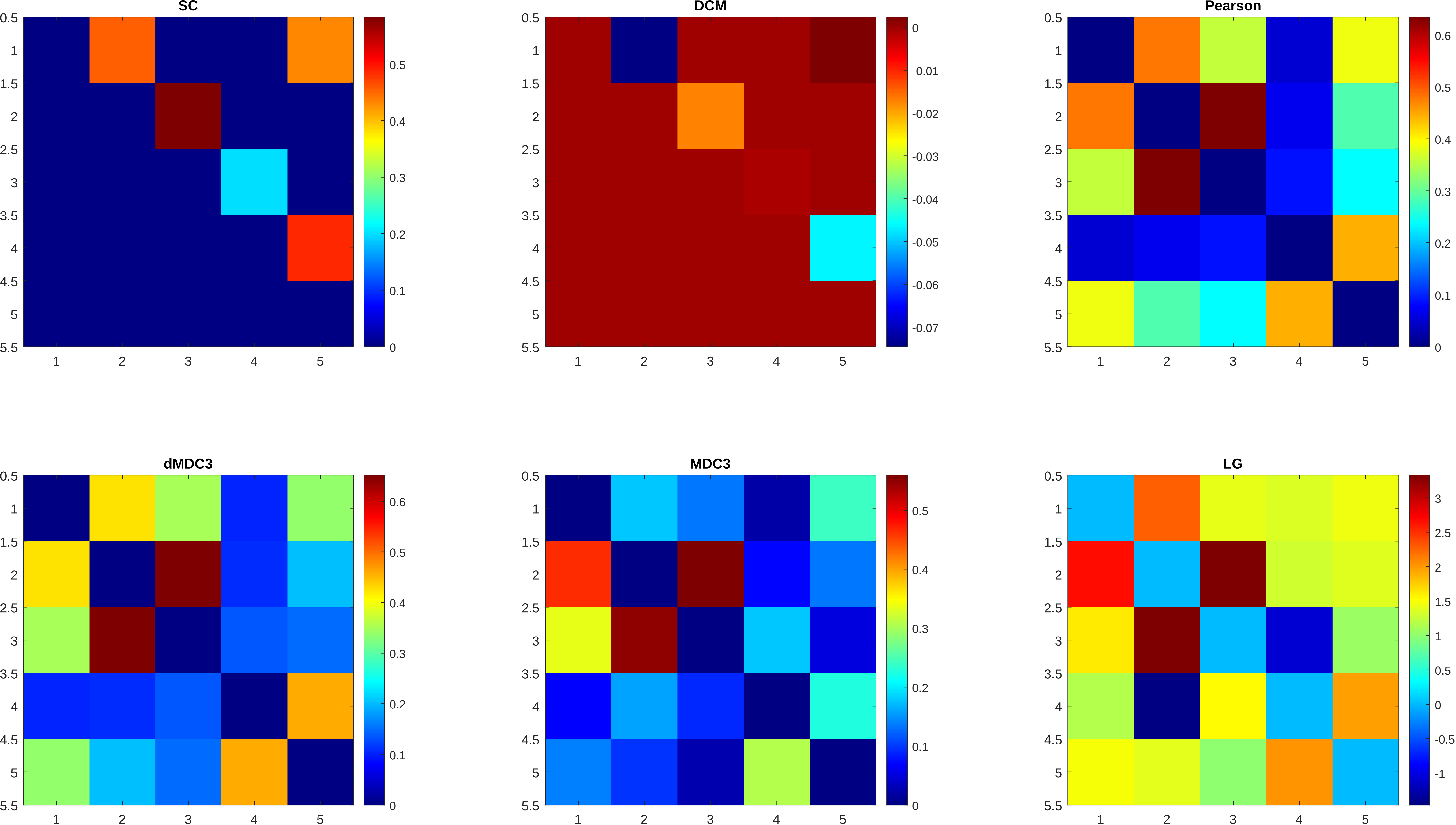
SC
DCM
Pearson
dMDC3
MDC3
LG
0.5 1 1.5 2 2.5 3 3.5 4 4.5 5 5.5
1 2 3 4 5
0 0.1 0.2 0.3 0.4 0.5
0 -0.01 -0.02 -0.03 -0.04 -0.05 -0.06 -0.07
0 0.1 0.2 0.3 0.4 0.5 0.6
-1 -0.5 0 0.5 1 1.5 2 2.5 3

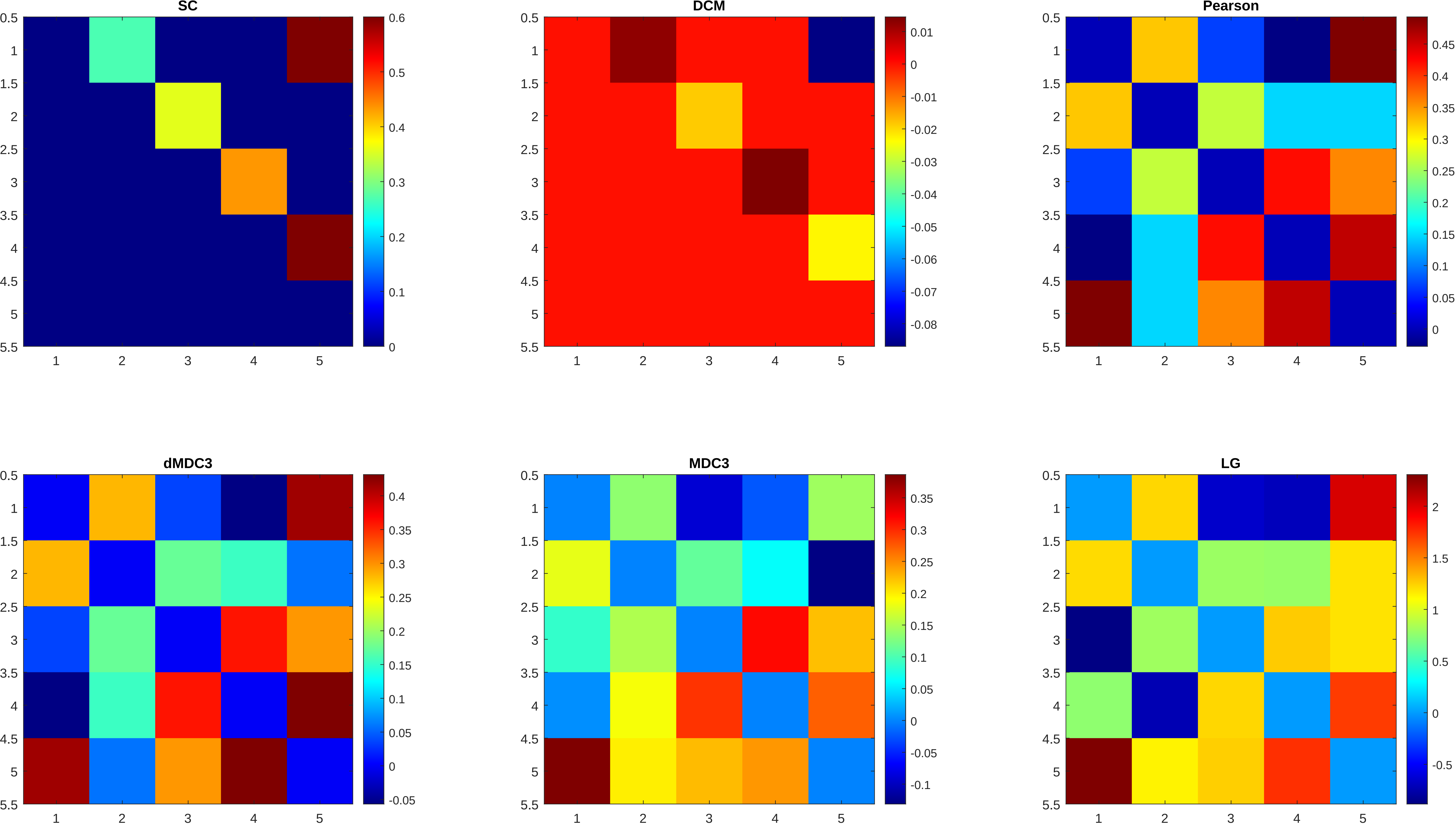
SC
0.5 1 1.5 2 2.5 3 3.5 4 4.5 5 5.5
1 2 3 4 5
0 0.1 0.2 0.3 0.4 0.5 0.6
DCM
0.5 1 1.5 2 2.5 3 3.5 4 4.5 5 5.5
1 2 3 4 5
-0.08 -0.07 -0.06 -0.05 -0.04 -0.03 -0.02 -0.01 0 0.01
Pearson
0.5 1 1.5 2 2.5 3 3.5 4 4.5 5 5.5
1 2 3 4 5
0 0.05 0.1 0.15 0.2 0.25 0.3 0.35 0.4 0.45
dMDC3
0.5 1 1.5 2 2.5 3 3.5 4 4.5 5 5.5
1 2 3 4 5
-0.05 0 0.05 0.1 0.15 0.2 0.25 0.3 0.35 0.4
MDC3
0.5 1 1.5 2 2.5 3 3.5 4 4.5 5 5.5
1 2 3 4 5
-0.1 -0.05 0 0.05 0.1 0.15 0.2 0.25 0.3 0.35
LG
0.5 1 1.5 2 2.5 3 3.5 4 4.5 5 5.5
1 2 3 4 5
-0.5 0 0.5 1 1.5 2

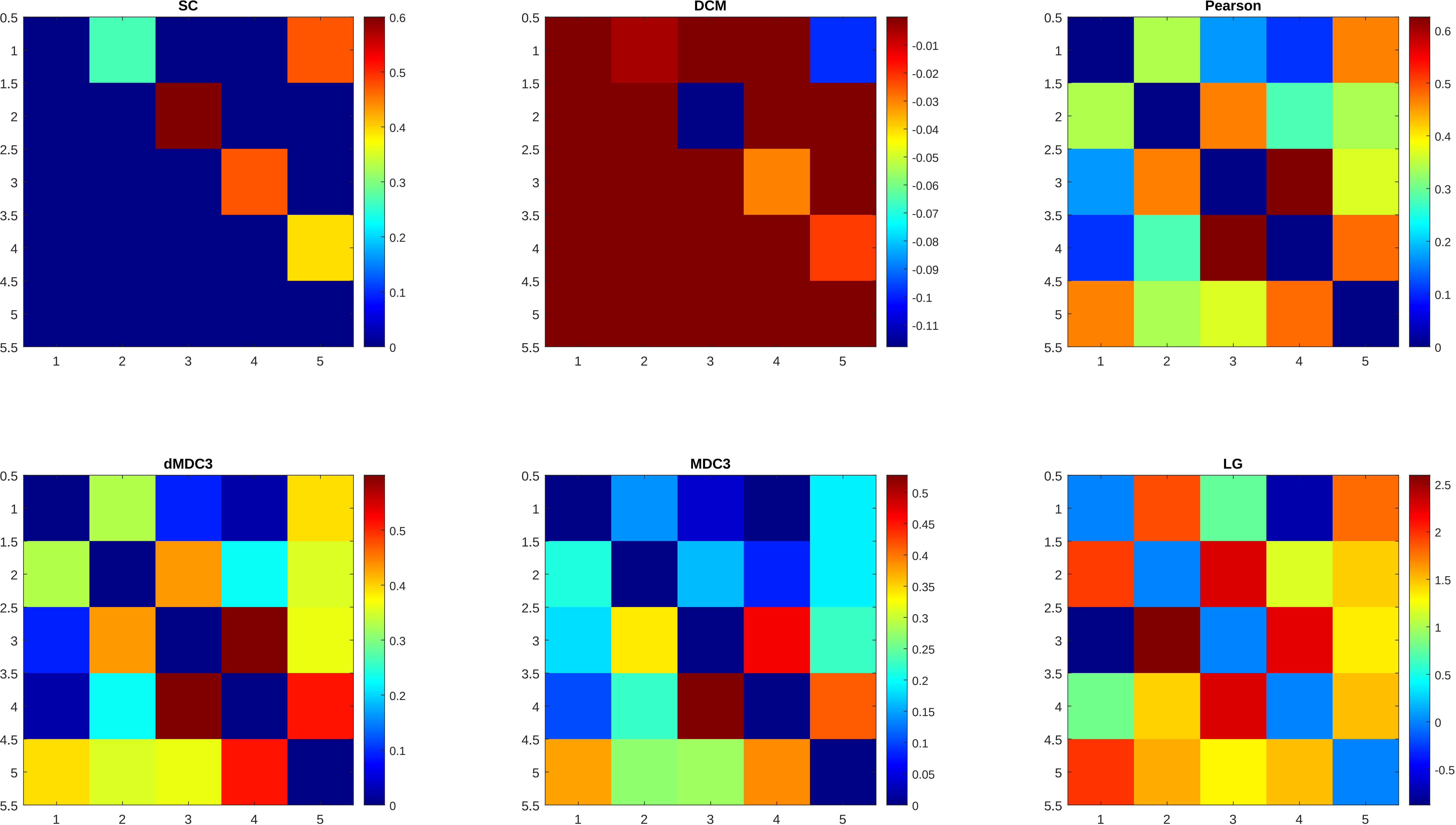
SC
DCM
Pearson
dMDC3
MDC3
LG

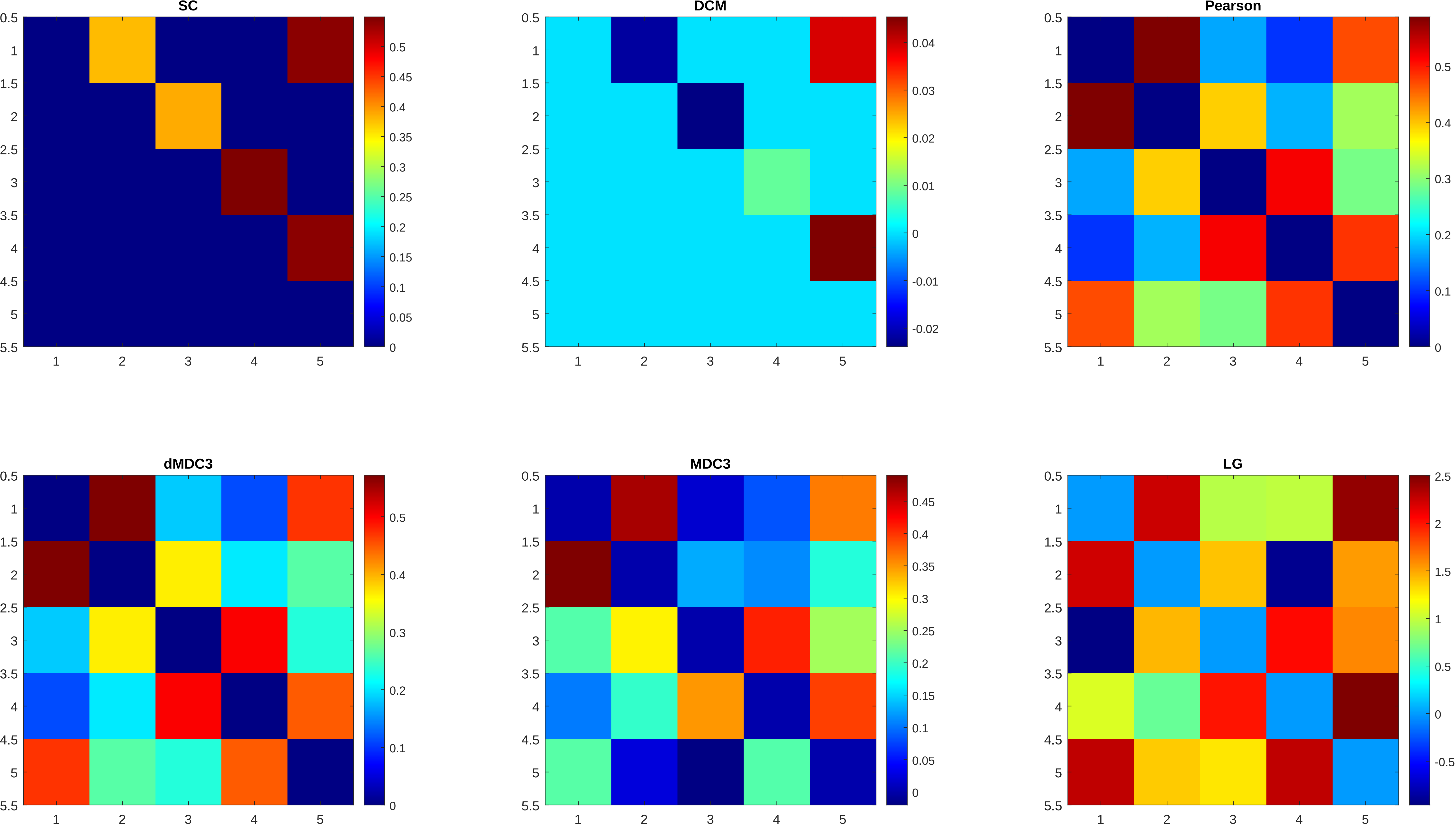
SC
DCM
Pearson
dMDC3
MDC3
LG

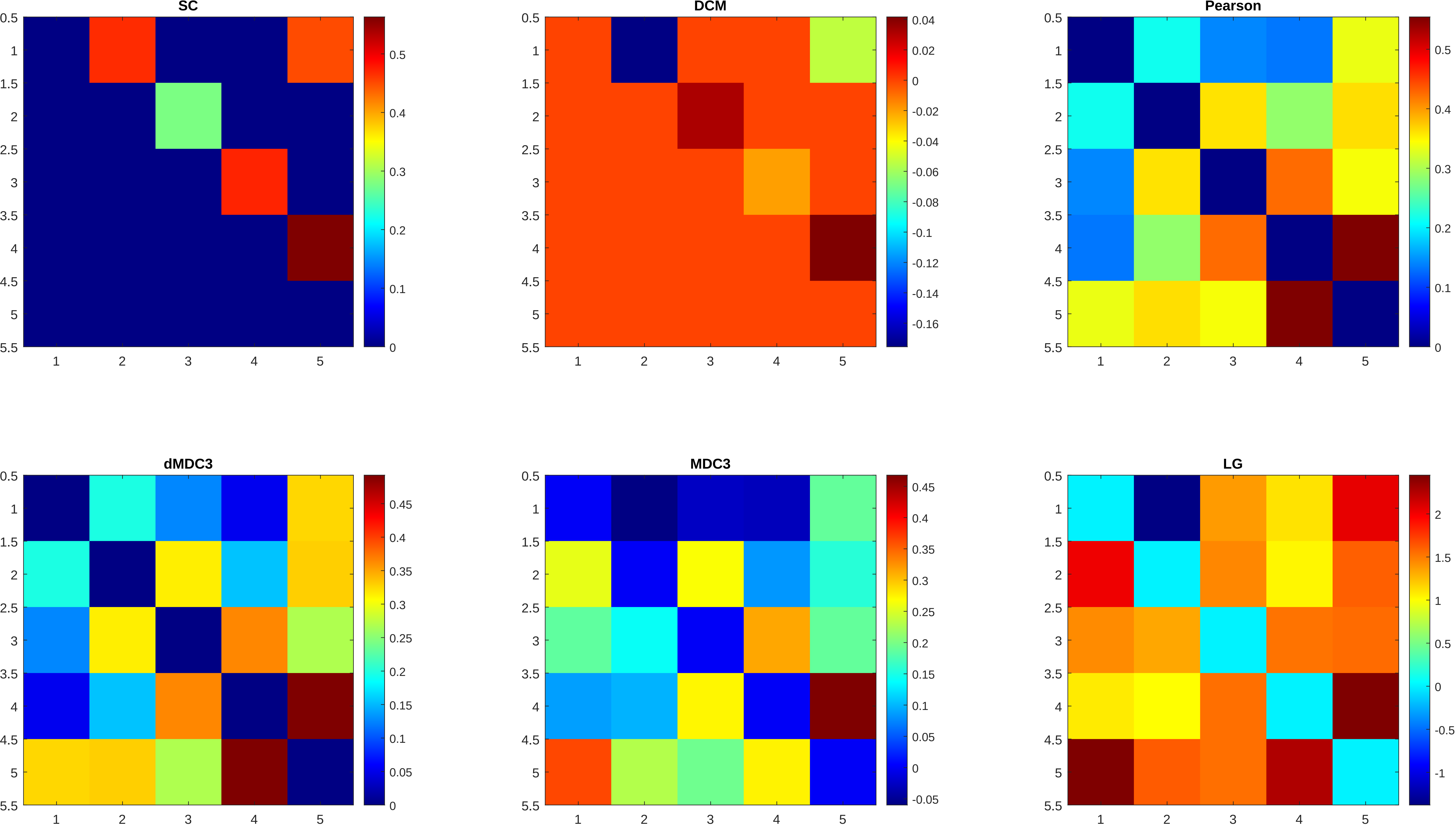
SC
DCM
Pearson
dMDC3
MDC3
LG

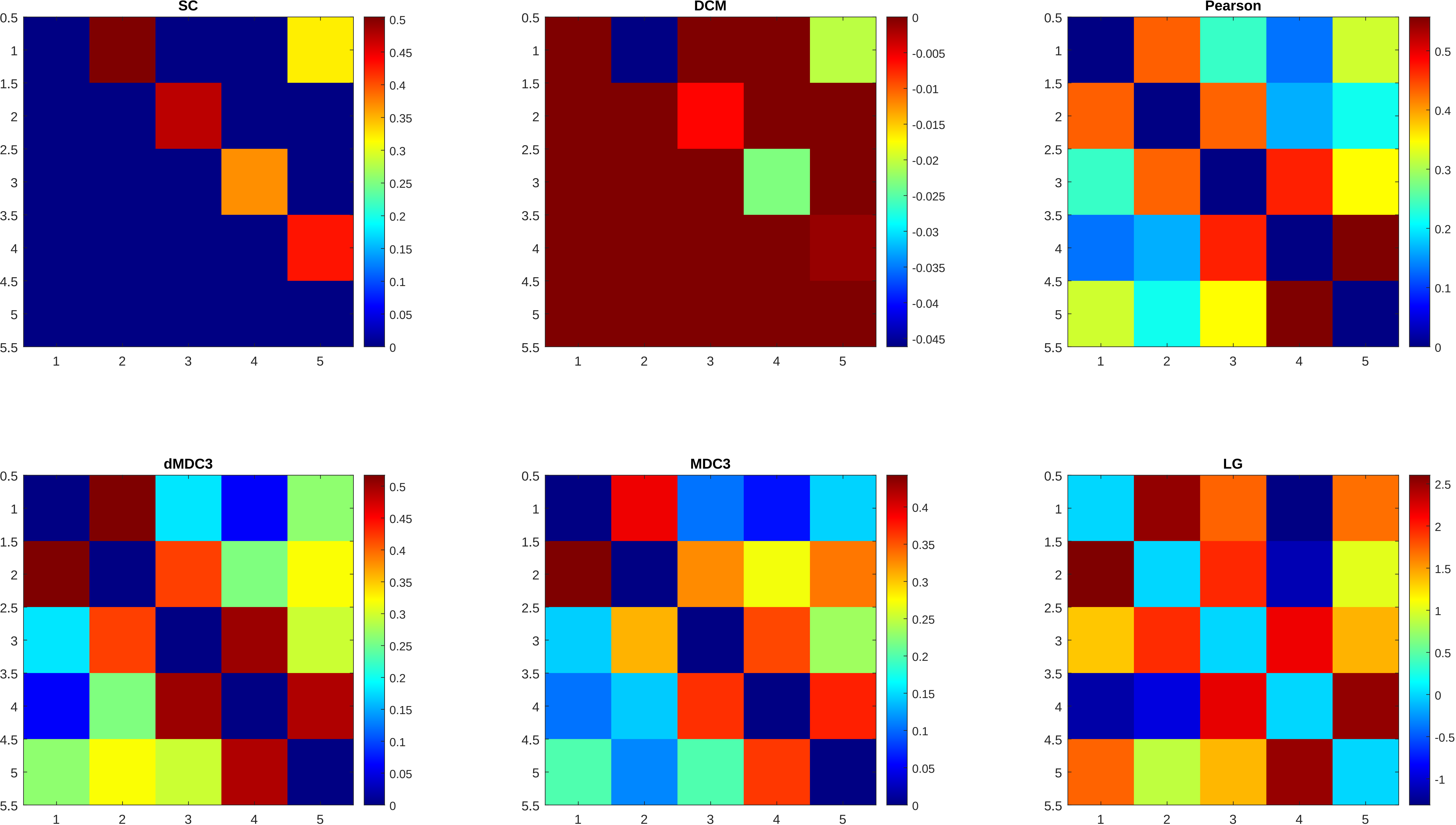

SC
DCM
Pearson
dMDC3
MDC3
LG
0.5
1
1.5
2
2.5
3
3.5
4
4.5
5
5.5
1
2
3
4
5
0
0.05
0.1
0.15
0.2
0.25
0.3
0.35
0.4
0.45
0.5
-0.005
-0.01
-0.015
-0.02
-0.025
-0.03
-0.035
-0.04
-0.045
2.5
-0.5
-1

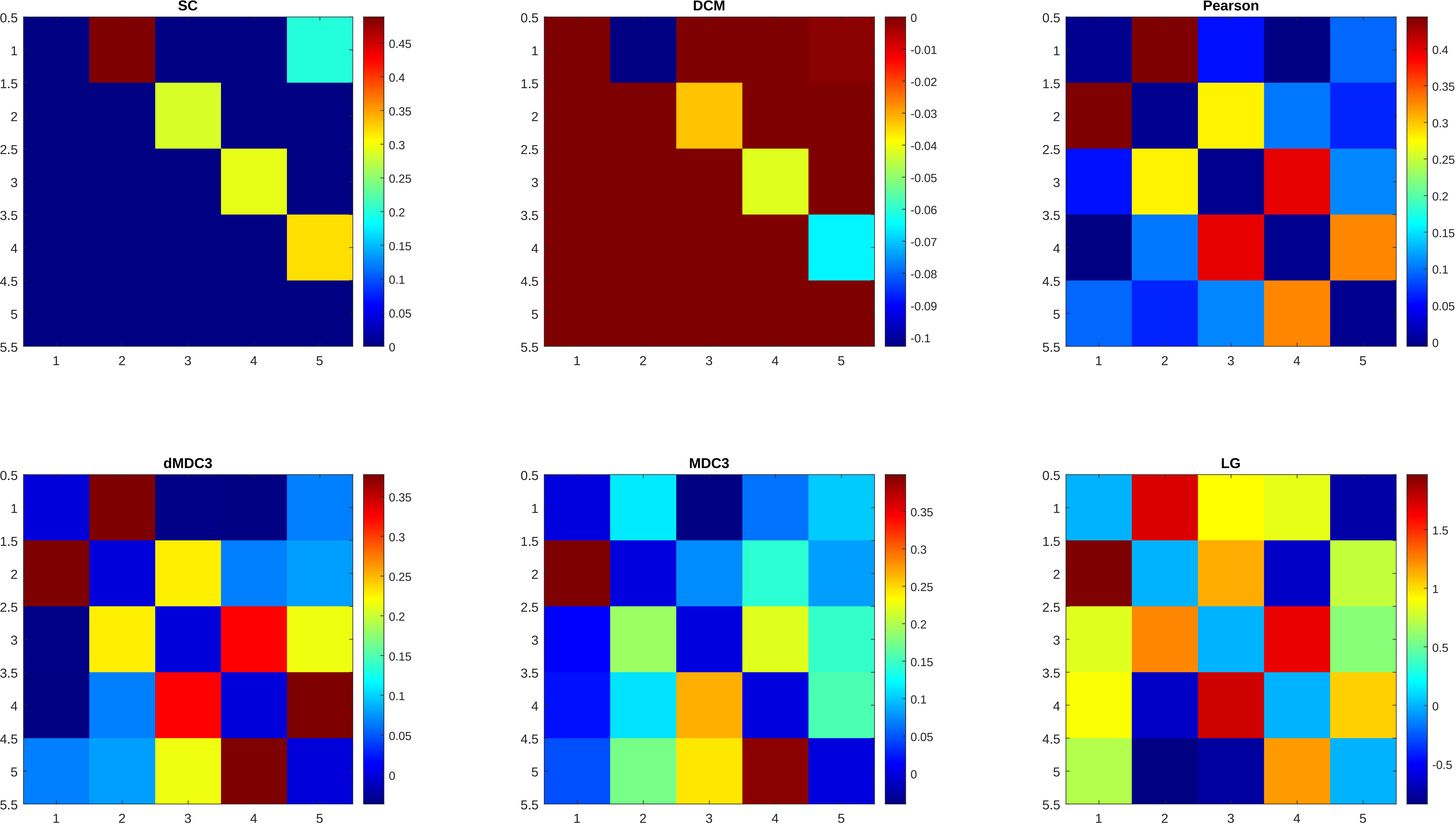
SC
DCM
Pearson
dMDC3
MDC3
LG

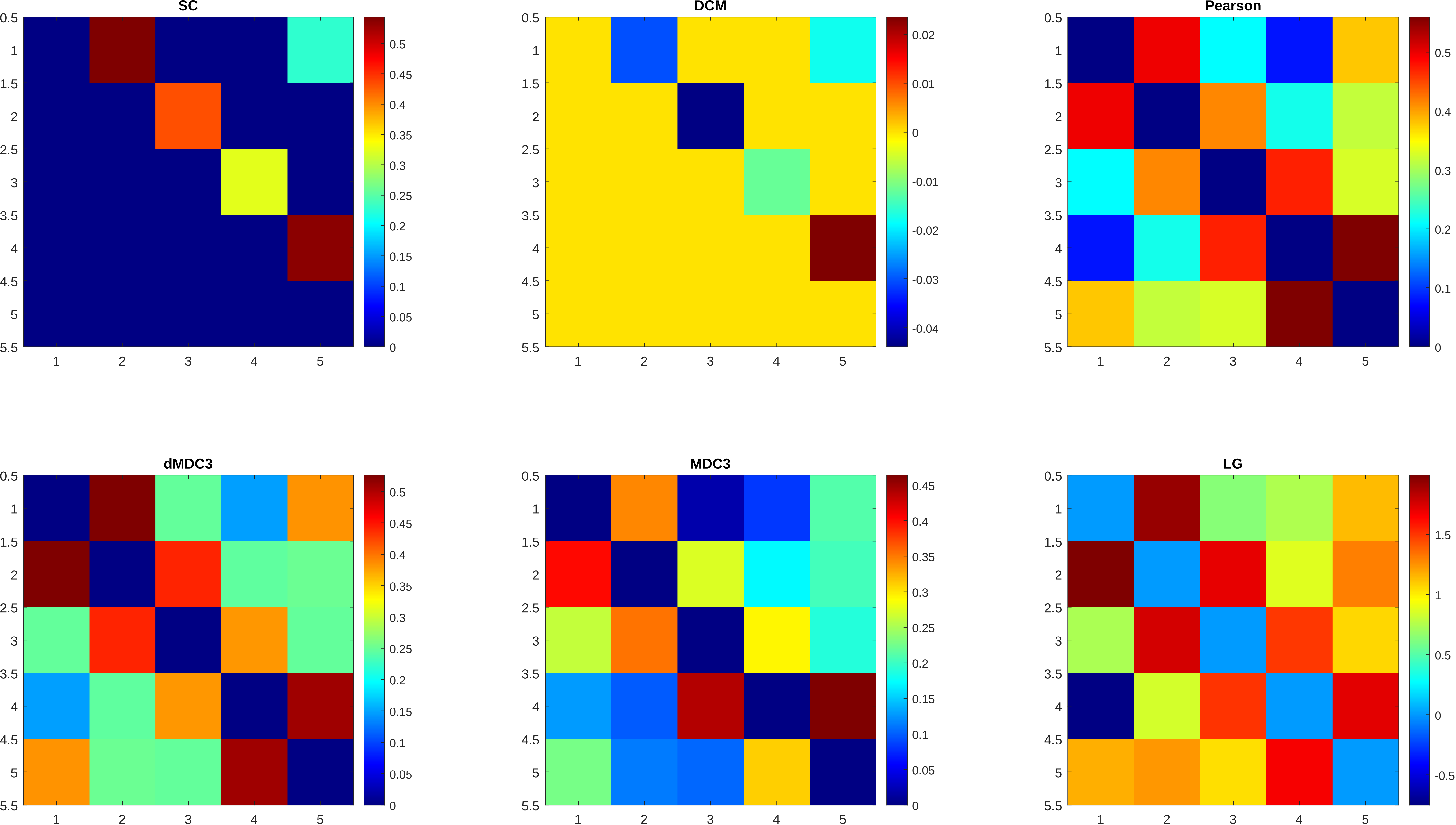
SC
DCM
Pearson
dMDC3
MDC3
LG
0.5
1
1.5
2
2.5
3
3.5
4
4.5
5
5.5
1
2
3
4
5
0
0.05
0.1
0.15
0.2
0.25
0.3
0.35
0.4
0.45
0.5
-0.04
-0.03
-0.02
-0.01
0.01
0.02
-0.5
1.5

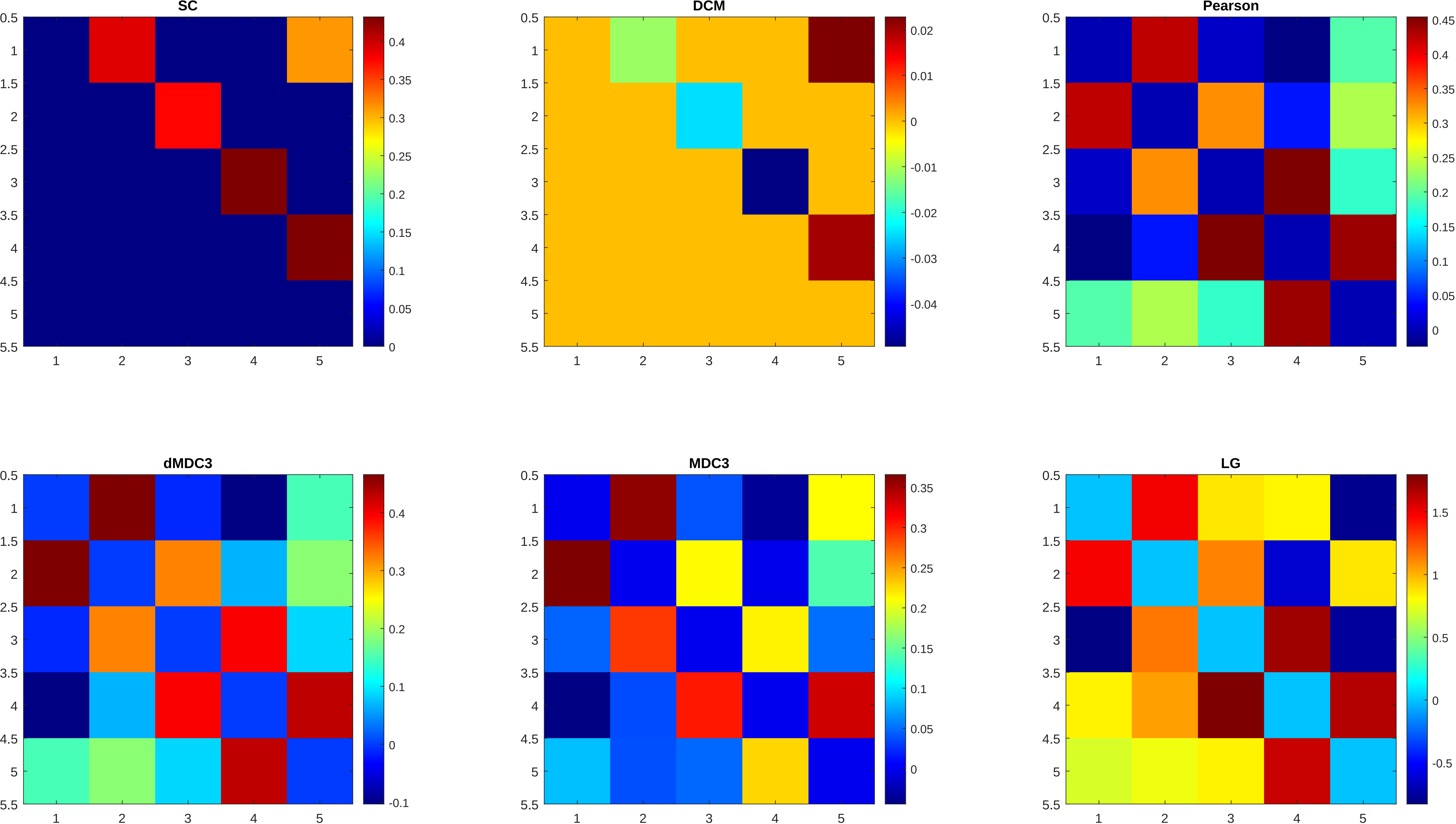
SC
DCM
Pearson
dMDC3
MDC3
LG

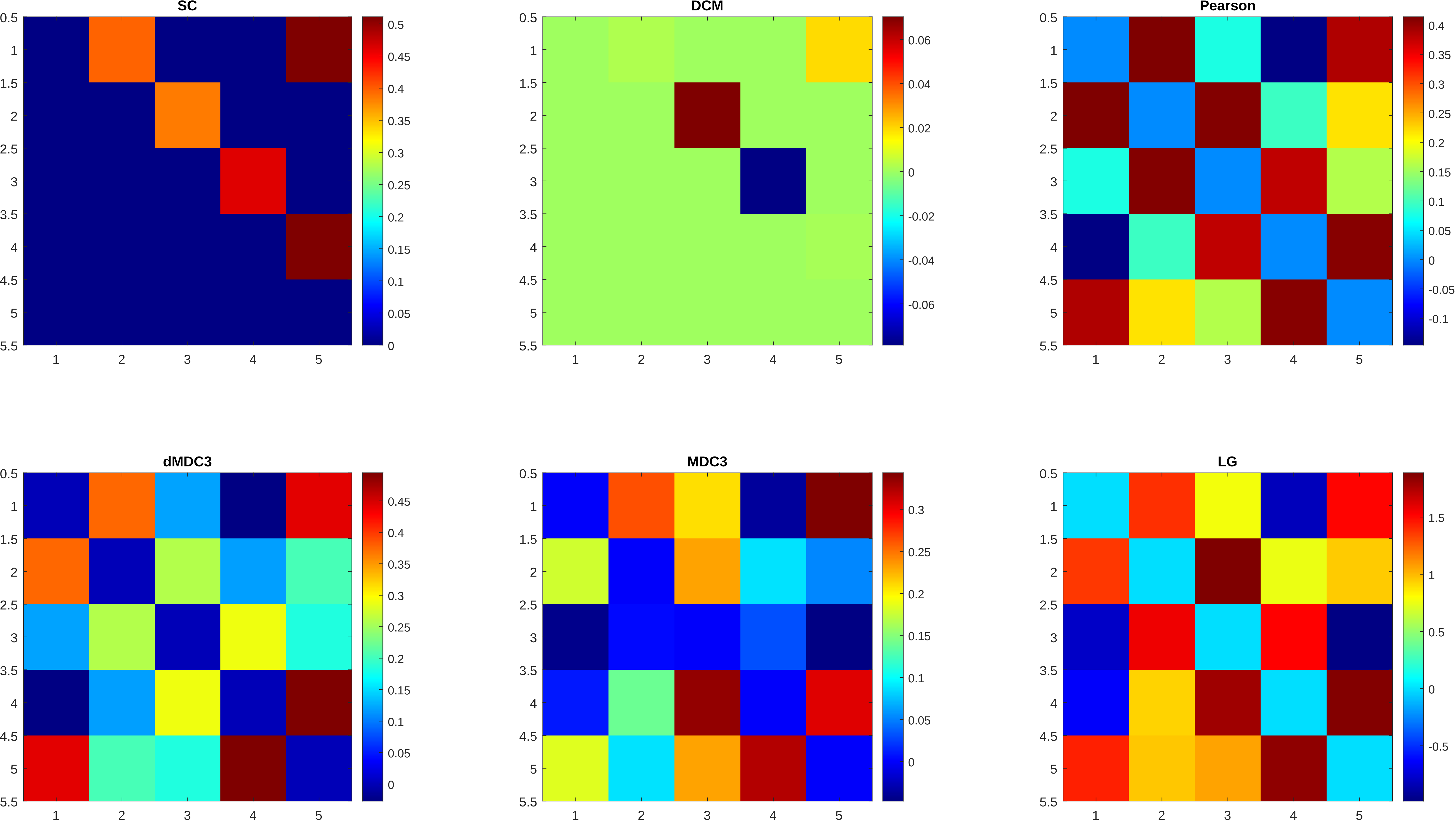
SC
DCM
Pearson
dMDC3
MDC3
LG
0.5 1 1.5 2 2.5 3 3.5 4 4.5 5 5.5
1 2 3 4 5

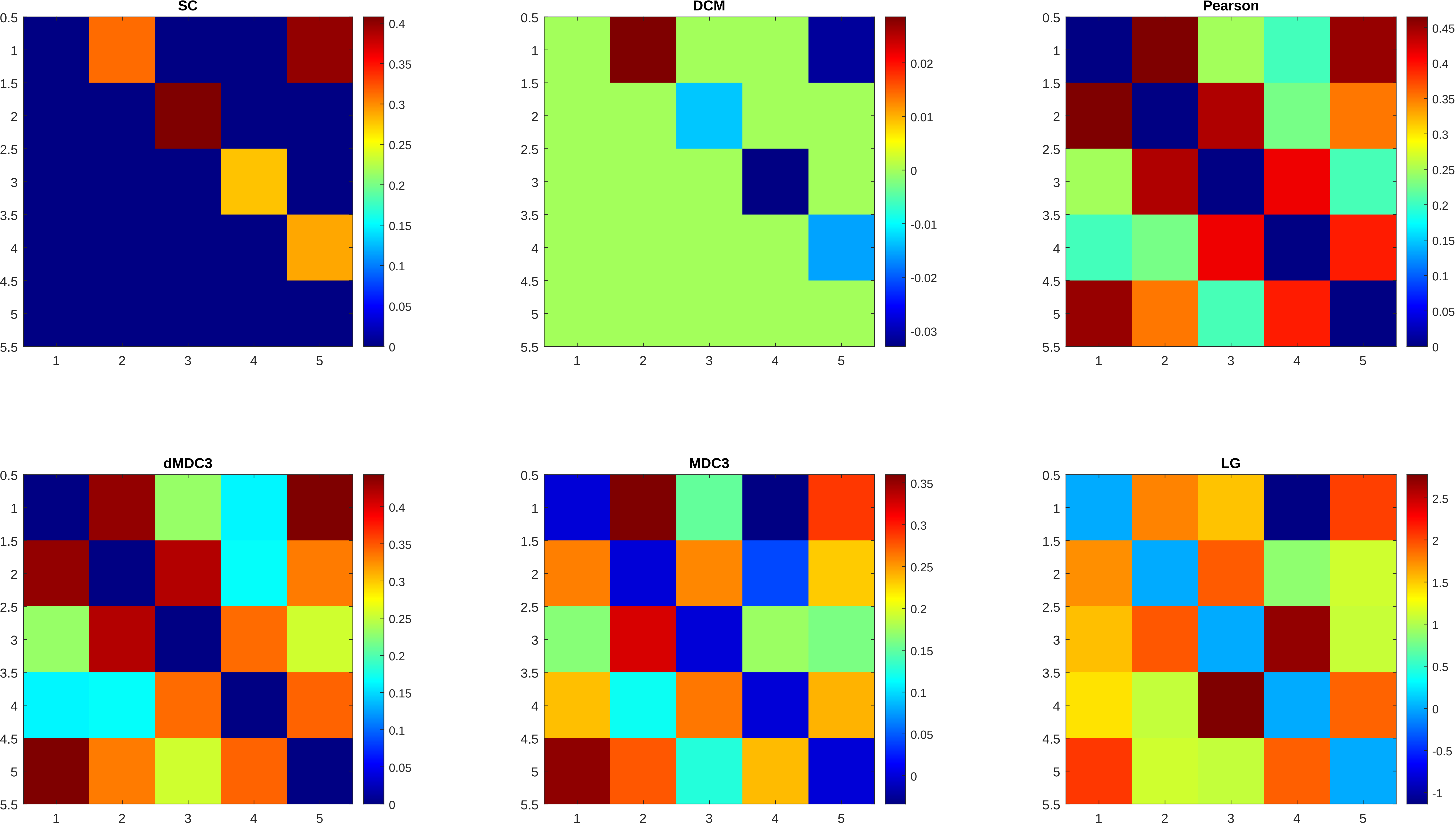

SC
DCM
Pearson
dMDC3
MDC3
LG

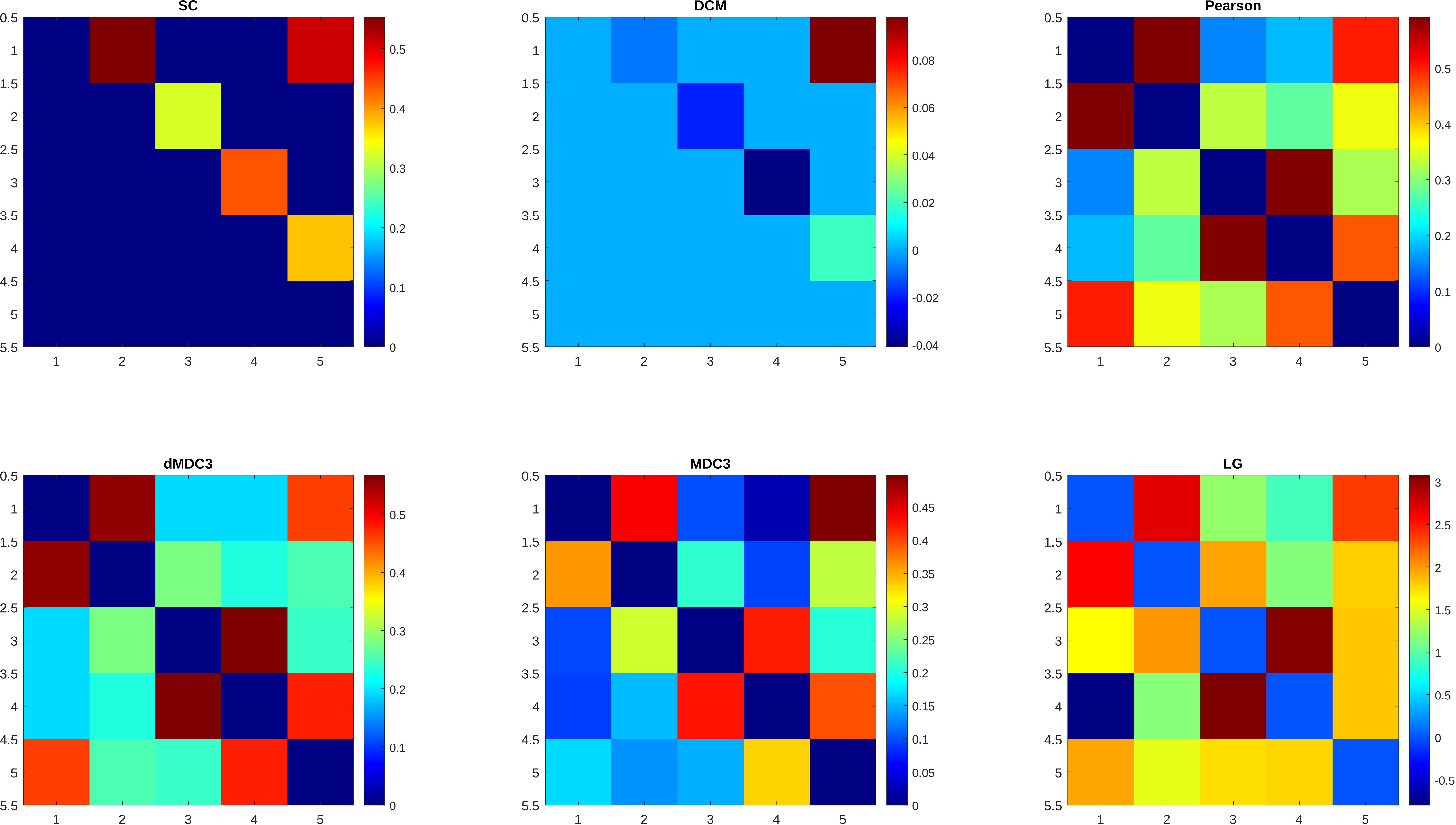
SC
DCM
Pearson
dMDC3
MDC3
LG

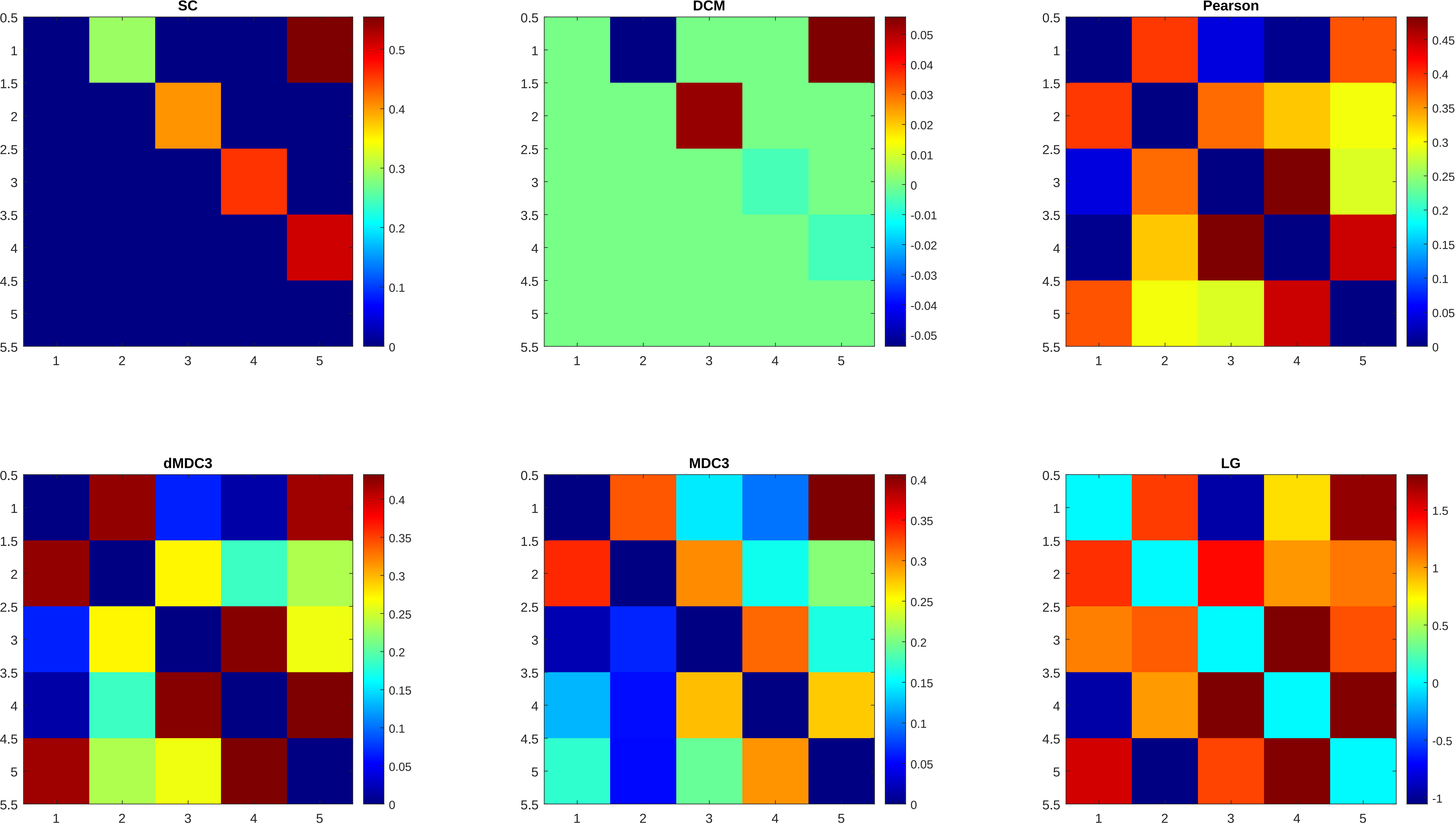
SC
DCM
Pearson
dMDC3
MDC3
LG
0.5
1
1.5
2
2.5
3
3.5
4
4.5
5
5.5
1
2
3
4
5
0
0.1
0.2
0.3
0.4
0.5
-0.05
-0.04
-0.03
-0.02
-0.01
0
0.01
0.02
0.03
0.04
0.05
0
0.05
0.1
0.15
0.2
0.25
0.3
0.35
0.4
0.45
-1
-0.5
0
0.5
1
1.5

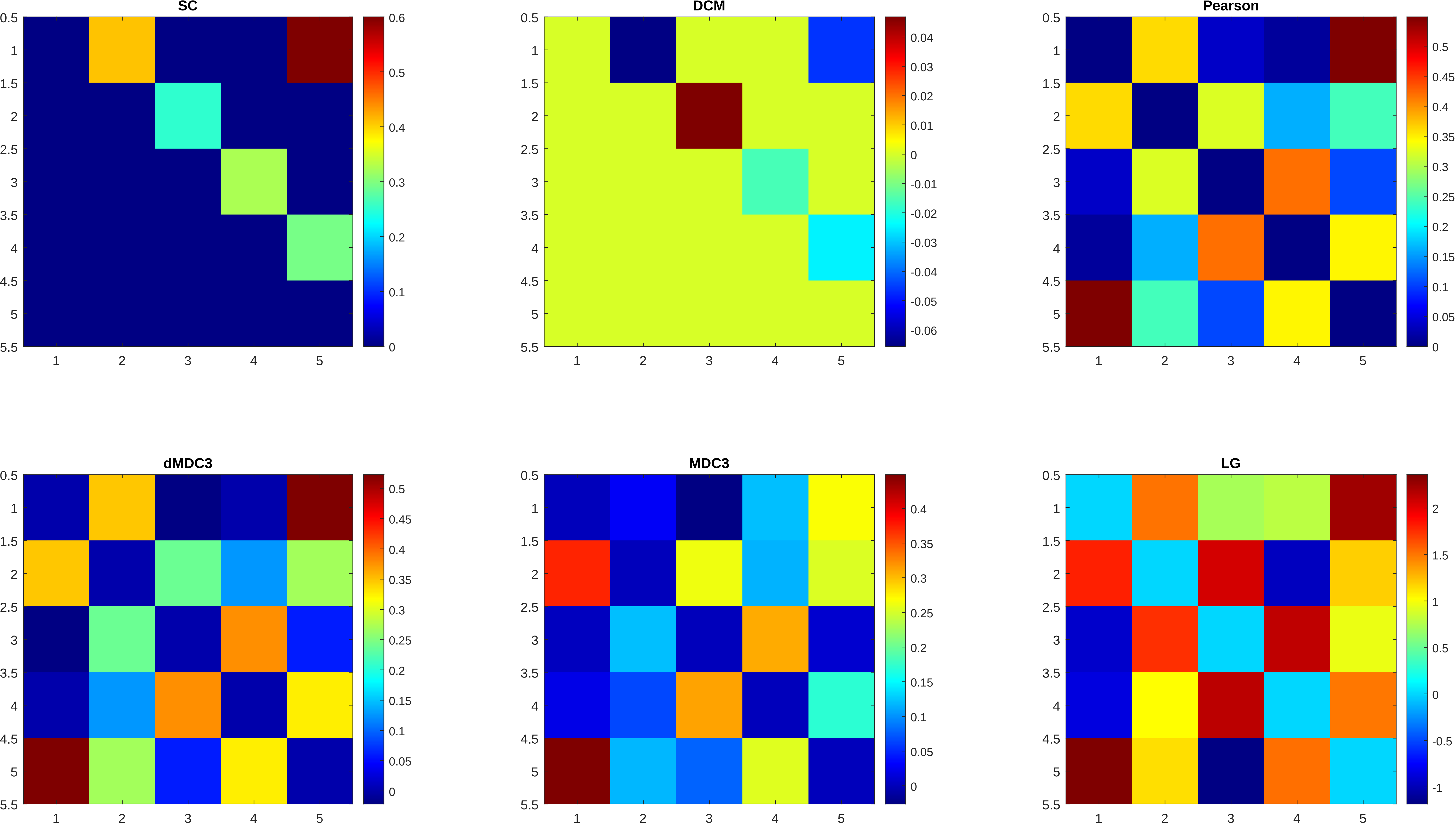
SC
DCM
Pearson
dMDC3
MDC3
LG
0.5 1 1.5 2 2.5 3 3.5 4 4.5 5 5.5
1 2 3 4 5
0 0.1 0.2 0.3 0.4 0.5 0.6
-0.06 -0.05 -0.04 -0.03 -0.02 -0.01 0 0.01 0.02 0.03 0.04
0 0.05 0.1 0.15 0.2 0.25 0.3 0.35 0.4 0.45 0.5
0 0.05 0.1 0.15 0.2 0.25 0.3 0.35 0.4 0.45 0.5
0 0.05 0.1 0.15 0.2 0.25 0.3 0.35 0.4
-1 -0.5 0 0.5 1 1.5 2

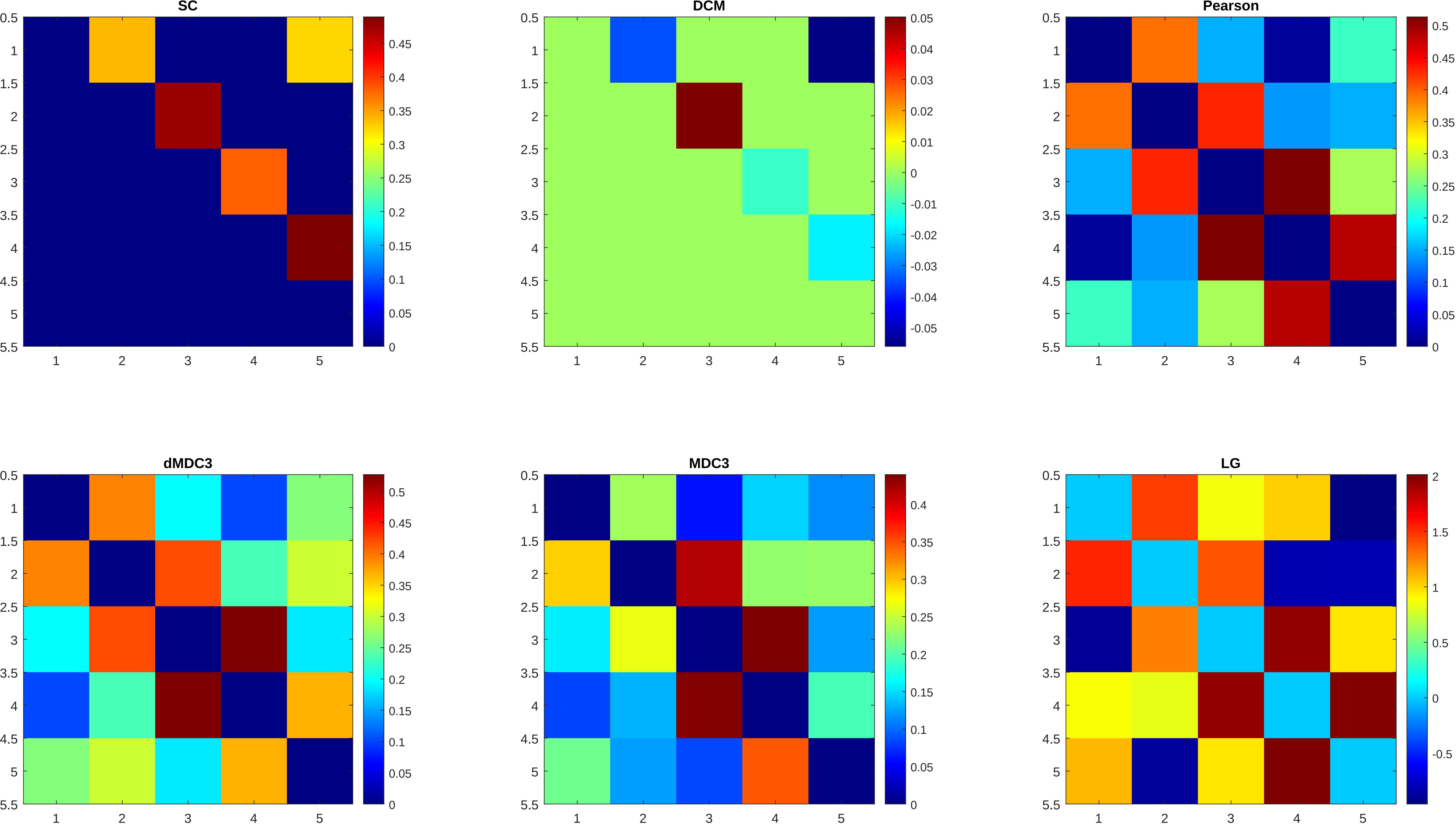

SC
DCM
Pearson
dMDC3
MDC3
LG
0.5
1
1.5
2
2.5
3
3.5
4
4.5
5
5.5
1
2
3
4
5
0
0.05
0.1
0.15
0.2
0.25
0.3
0.35
0.4
0.45
0.5
-0.05
-0.04
-0.03
-0.02
-0.01
0.01
0.02
0.03
0.04
-0.5
0
0.5
1
1.5
2

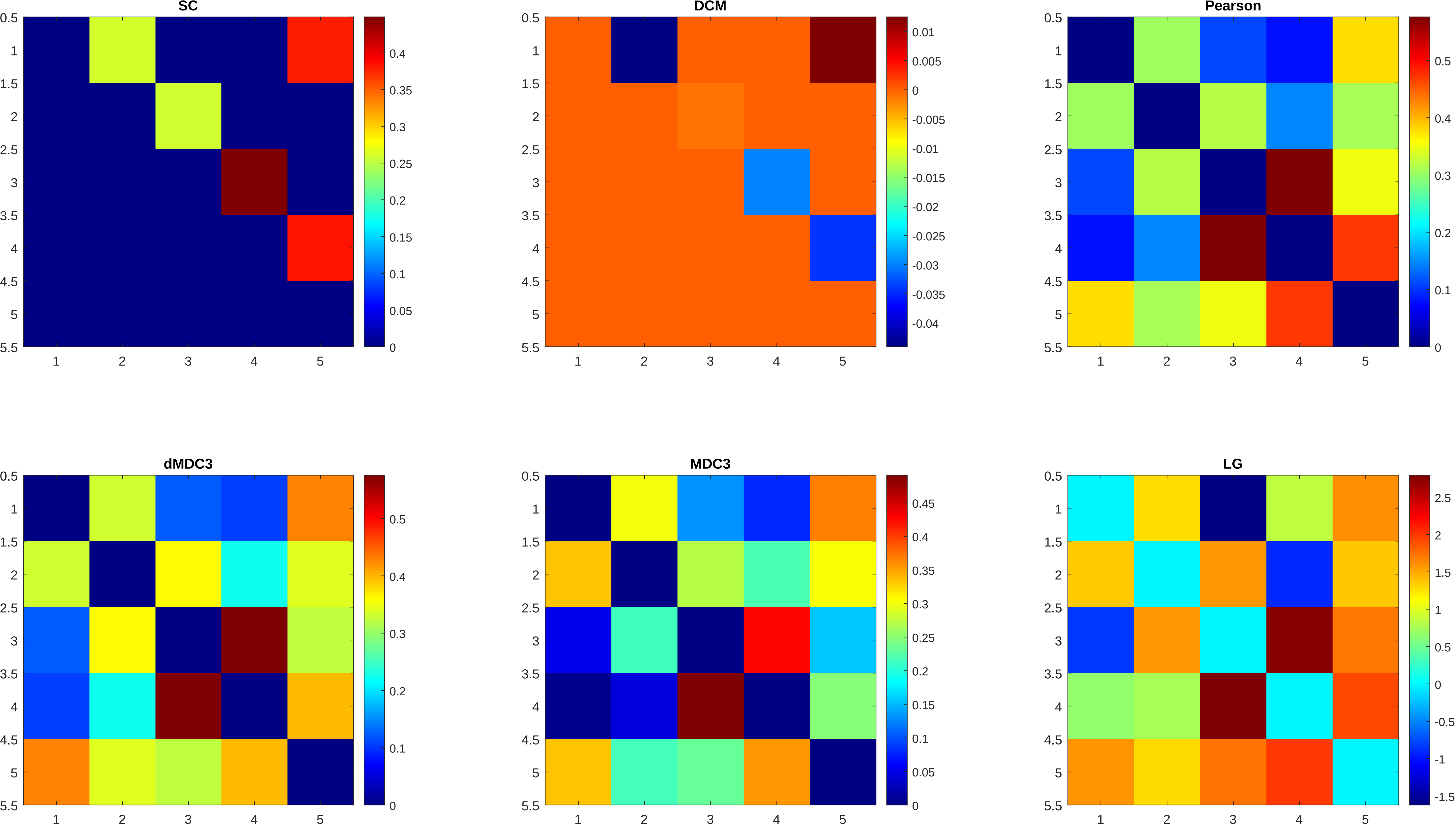
SC
DCM
Pearson
dMDC3
MDC3
LG

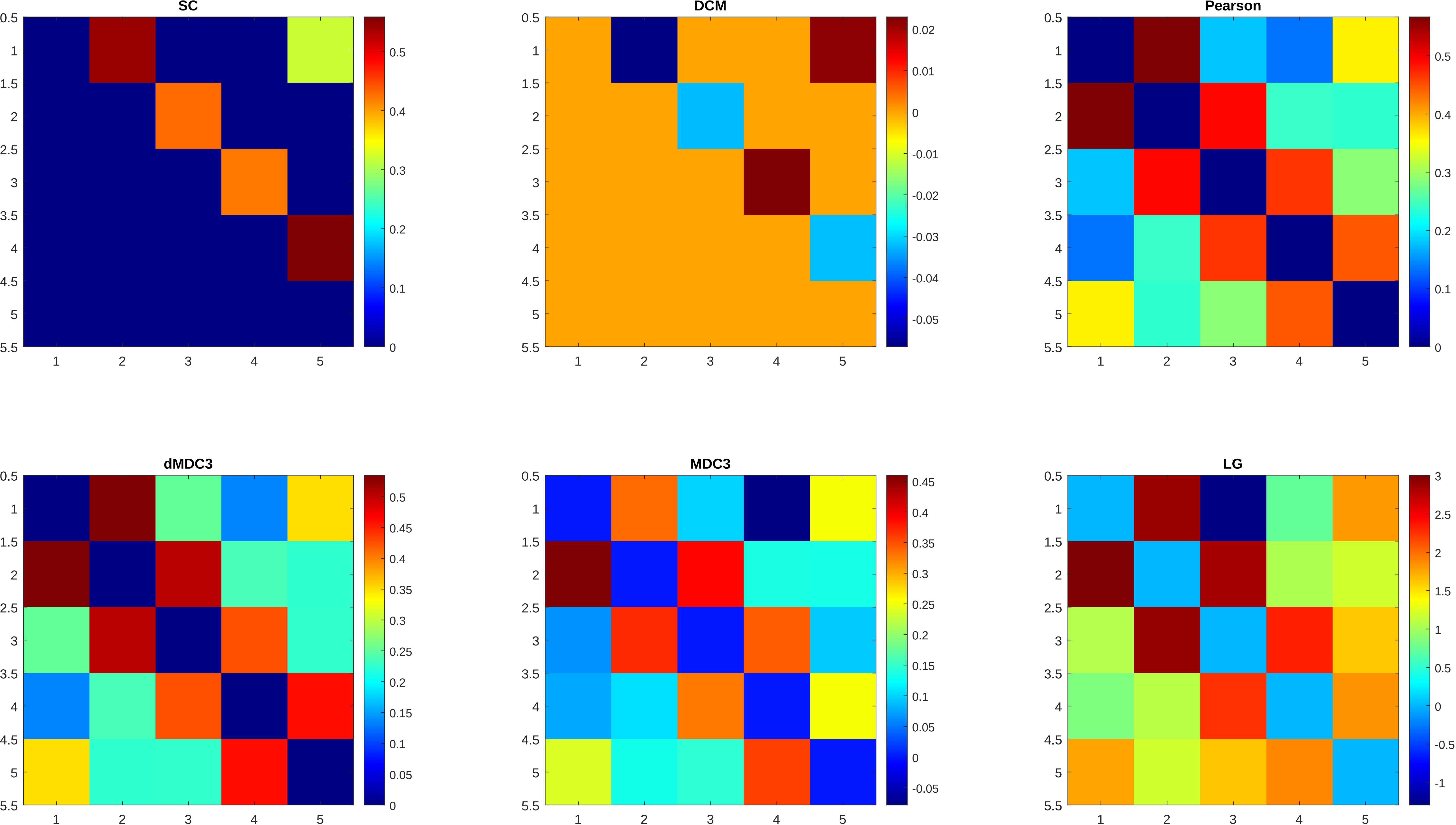
SC
DCM
Pearson
dMDC3
MDC3
LG

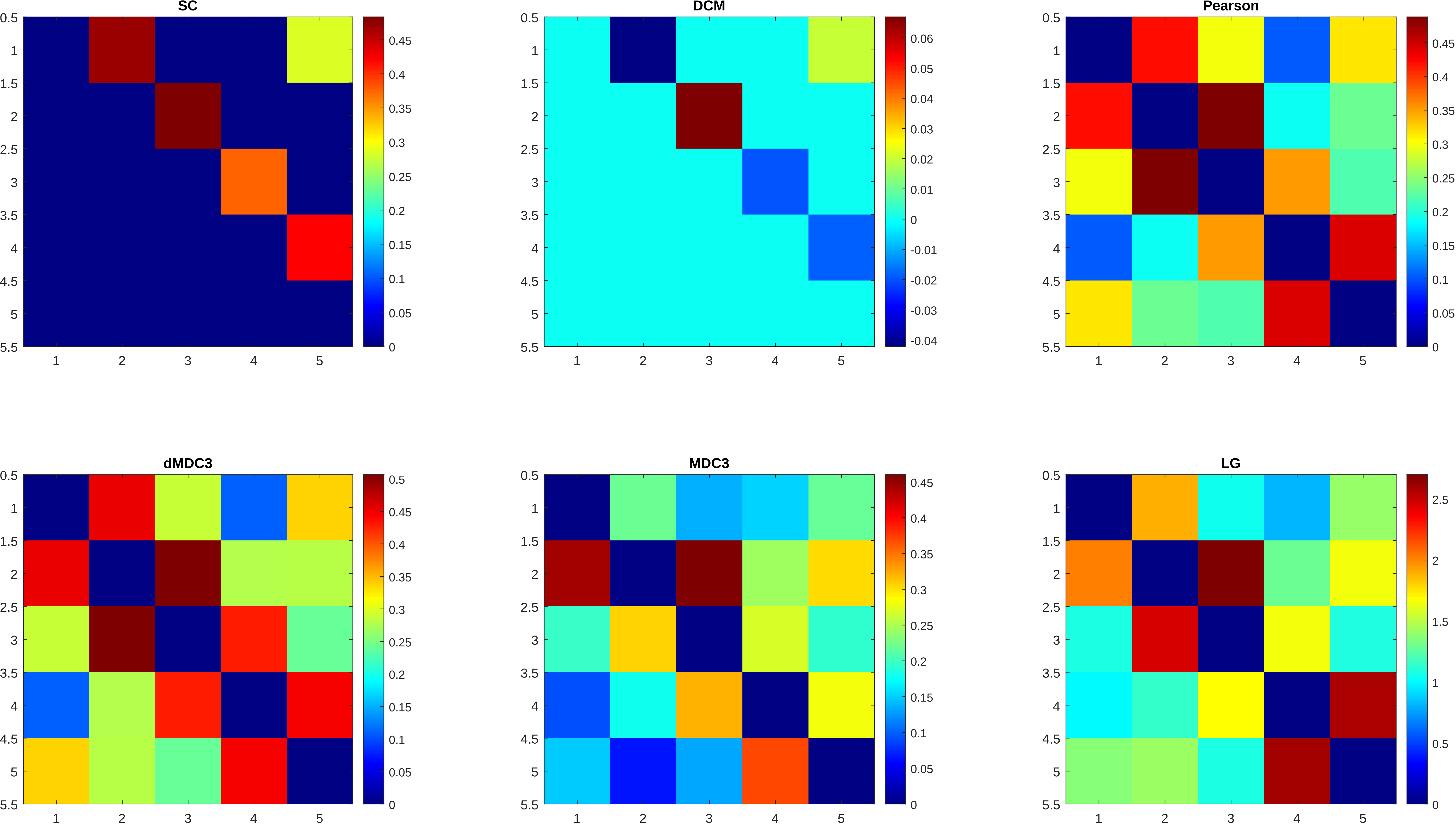
SC
DCM
Pearson
dMDC3
MDC3
LG

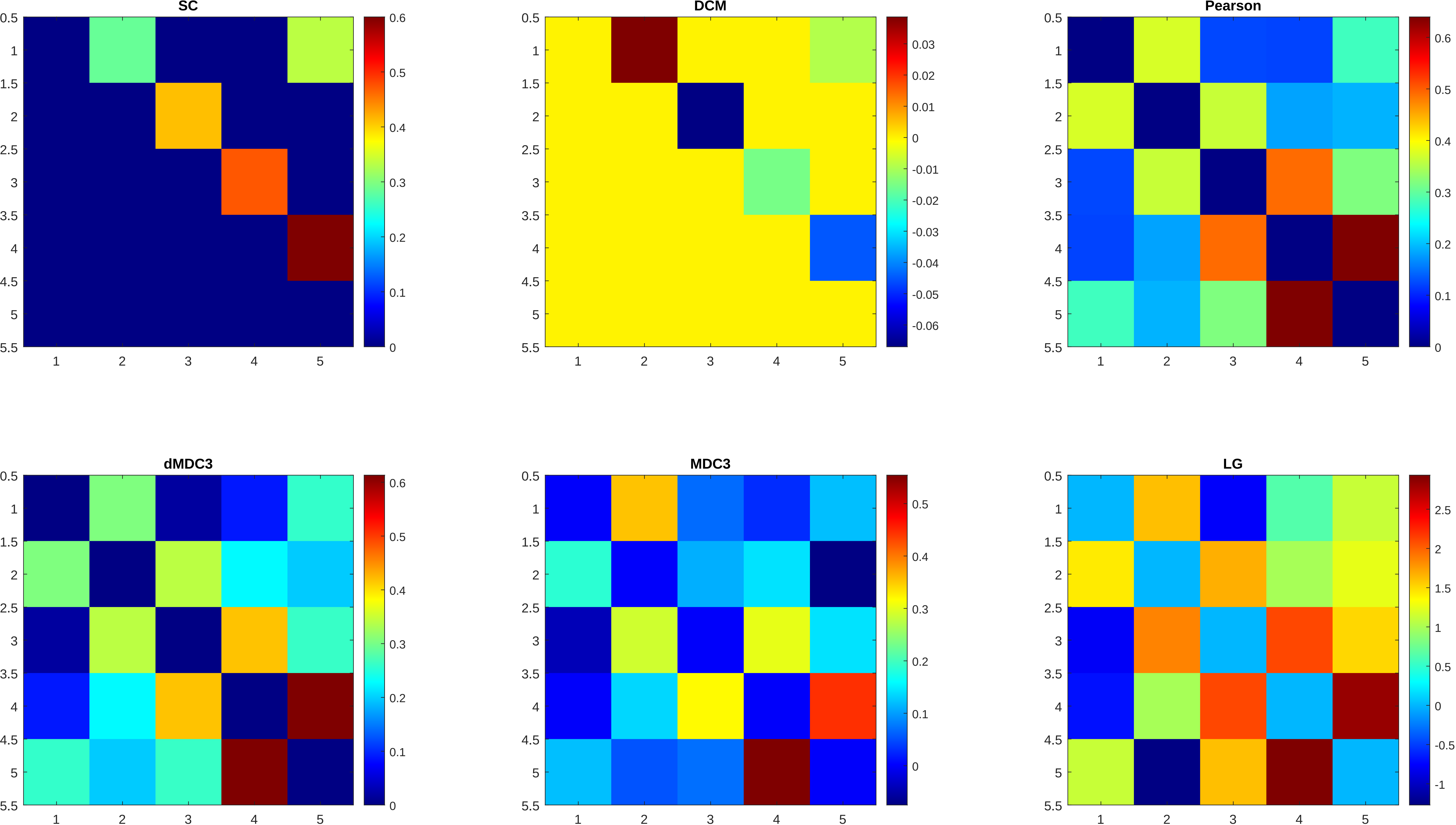
SC
DCM
Pearson
dMDC3
MDC3
LG
0.5 1 1.5 2 2.5 3 3.5 4 4.5 5 5.5
1 2 3 4 5
0 0.1 0.2 0.3 0.4 0.5 0.6
-0.06 -0.05 -0.04 -0.03 -0.02 -0.01 0 0.01 0.02 0.03
0 0.1 0.2 0.3 0.4 0.5
-1 -0.5 0 0.5 1 1.5 2 2.5

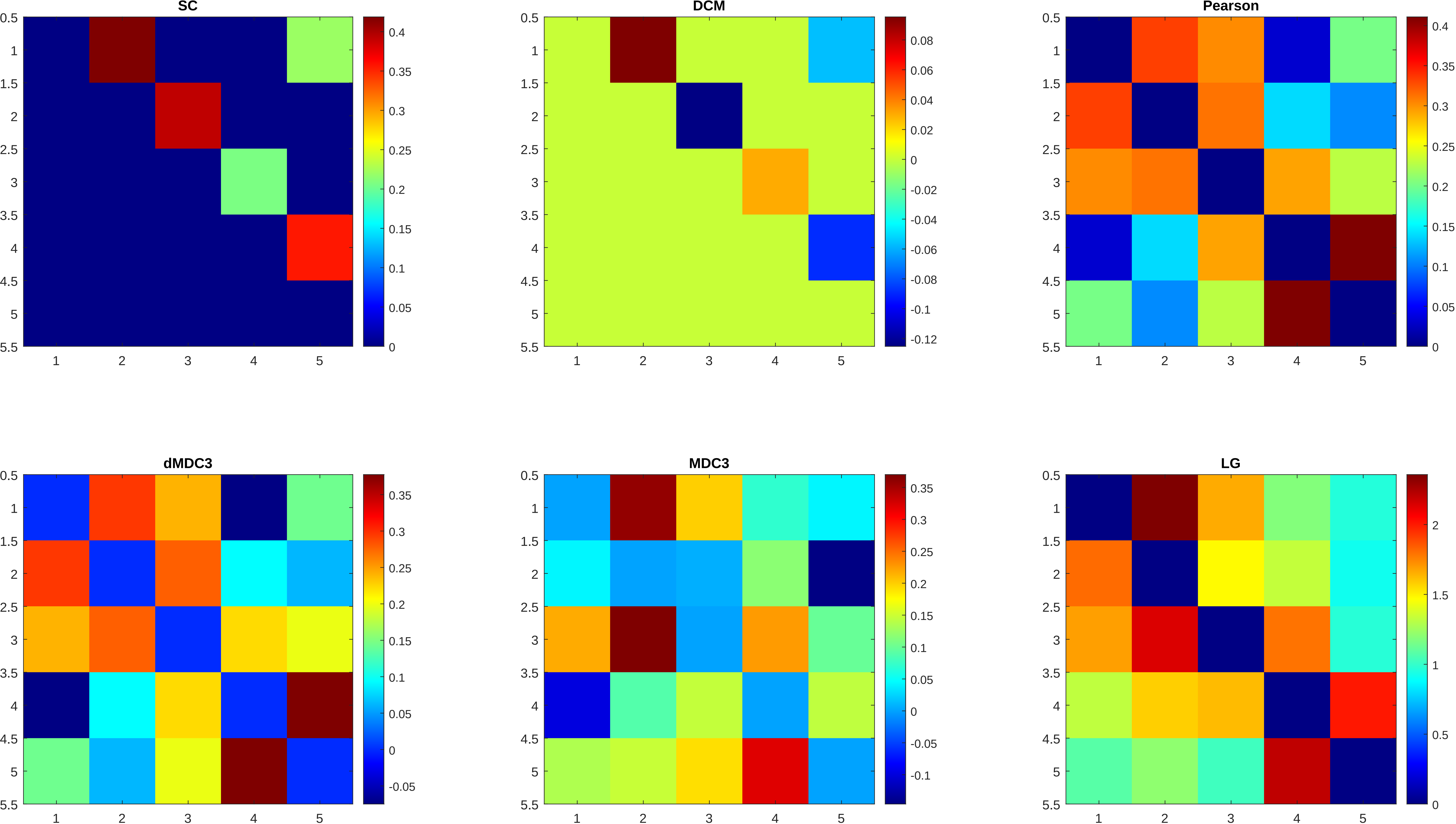
SC
0.5
1
1.5
2
2.5
3
3.5
4
4.5
5
5.5
1 2 3 4 5
0 0.05 0.1 0.15 0.2 0.25 0.3 0.35 0.4
DCM
-0.12 -0.1 -0.08 -0.06 -0.04 -0.02 0 0.02 0.04 0.06 0.08
Pearson
0 0.05 0.1 0.15 0.2 0.25 0.3 0.35 0.4
dMDC3
-0.05 0 0.05 0.1 0.15 0.2 0.25 0.3 0.35
MDC3
-0.1 -0.05 0 0.05 0.1 0.15 0.2 0.25 0.3 0.35
LG
0 0.5 1 1.5 2

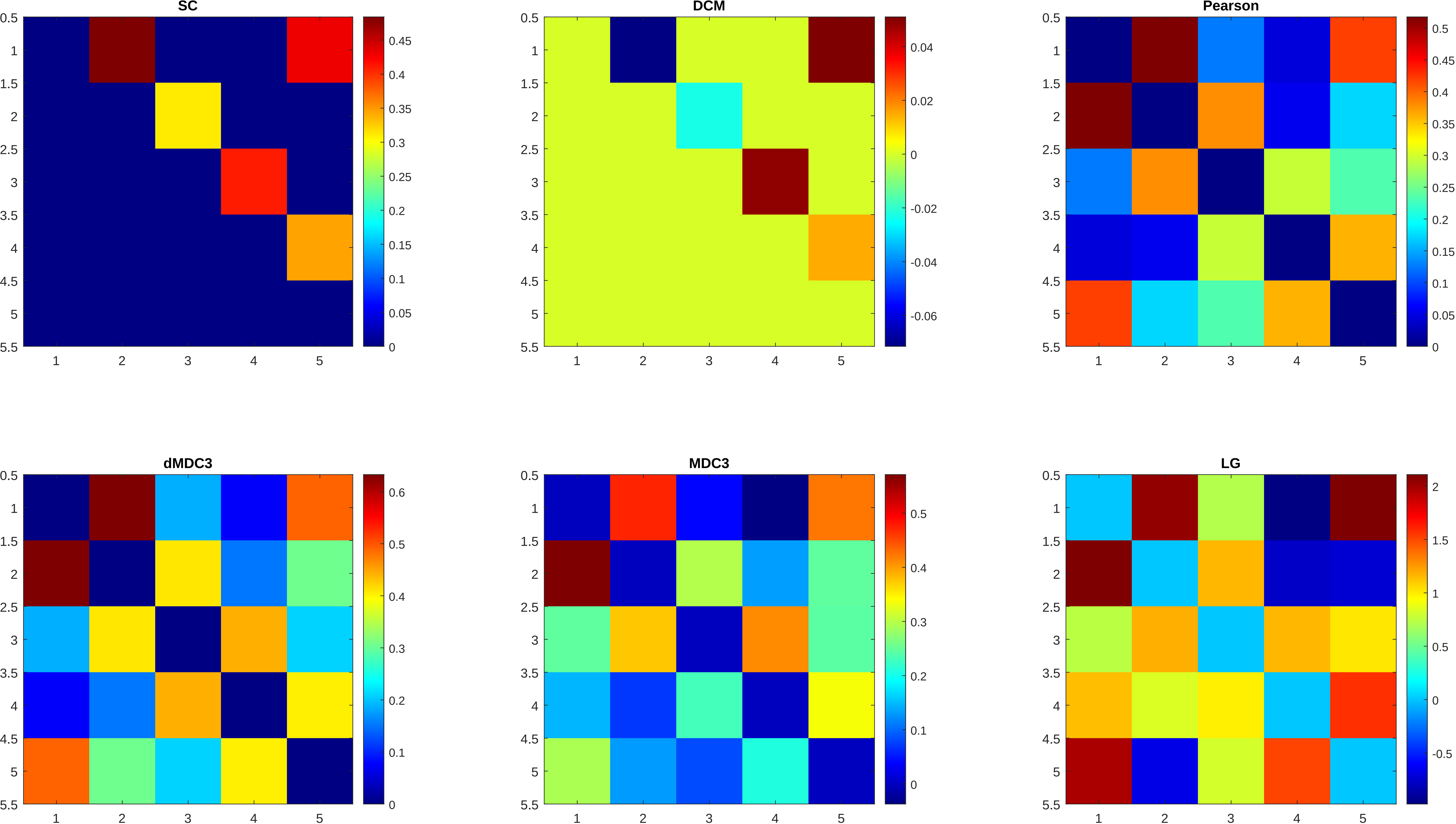
SC
DCM
Pearson
dMDC3
MDC3
LG

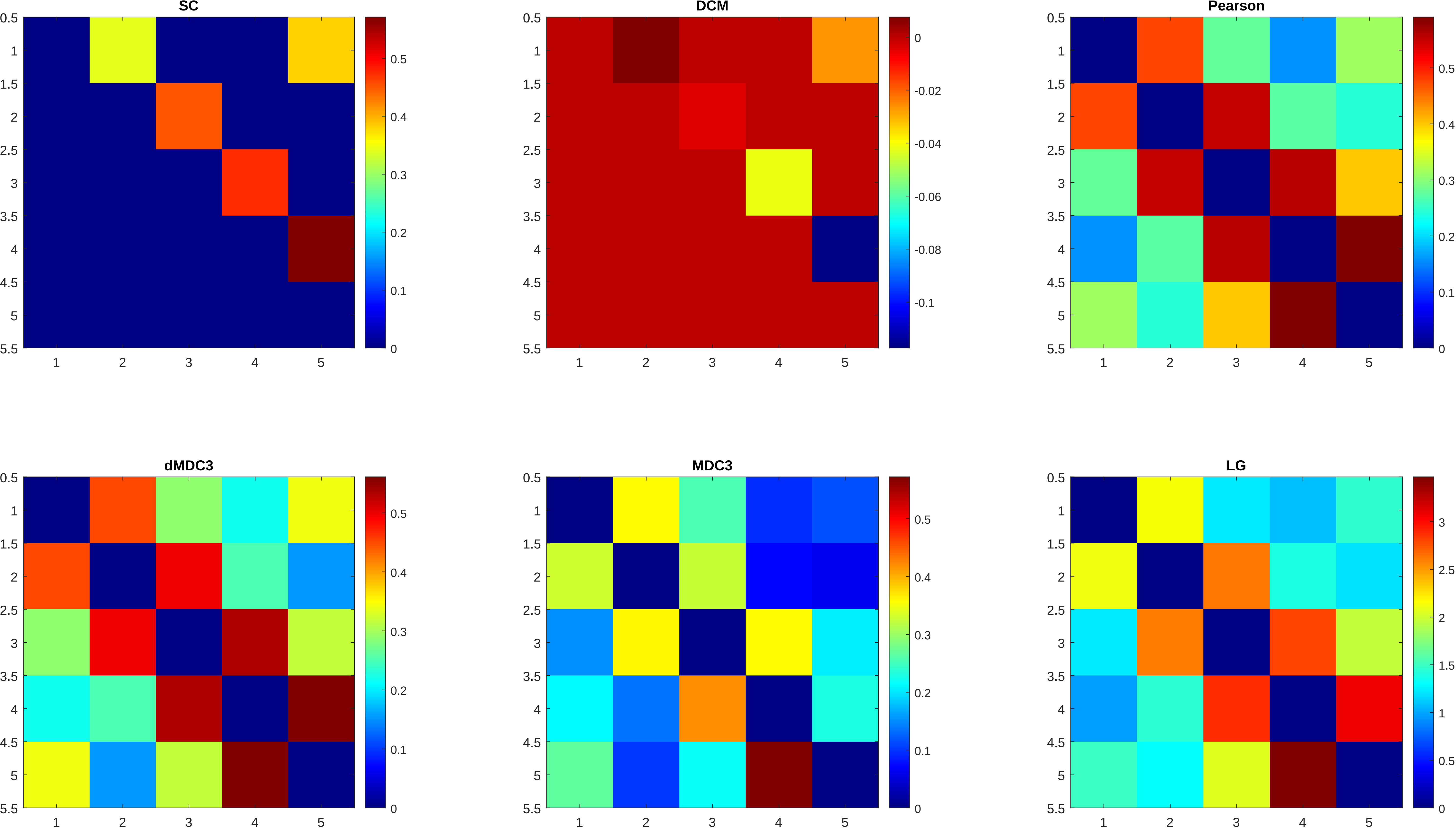
SC
DCM
Pearson
dMDC3
MDC3
LG

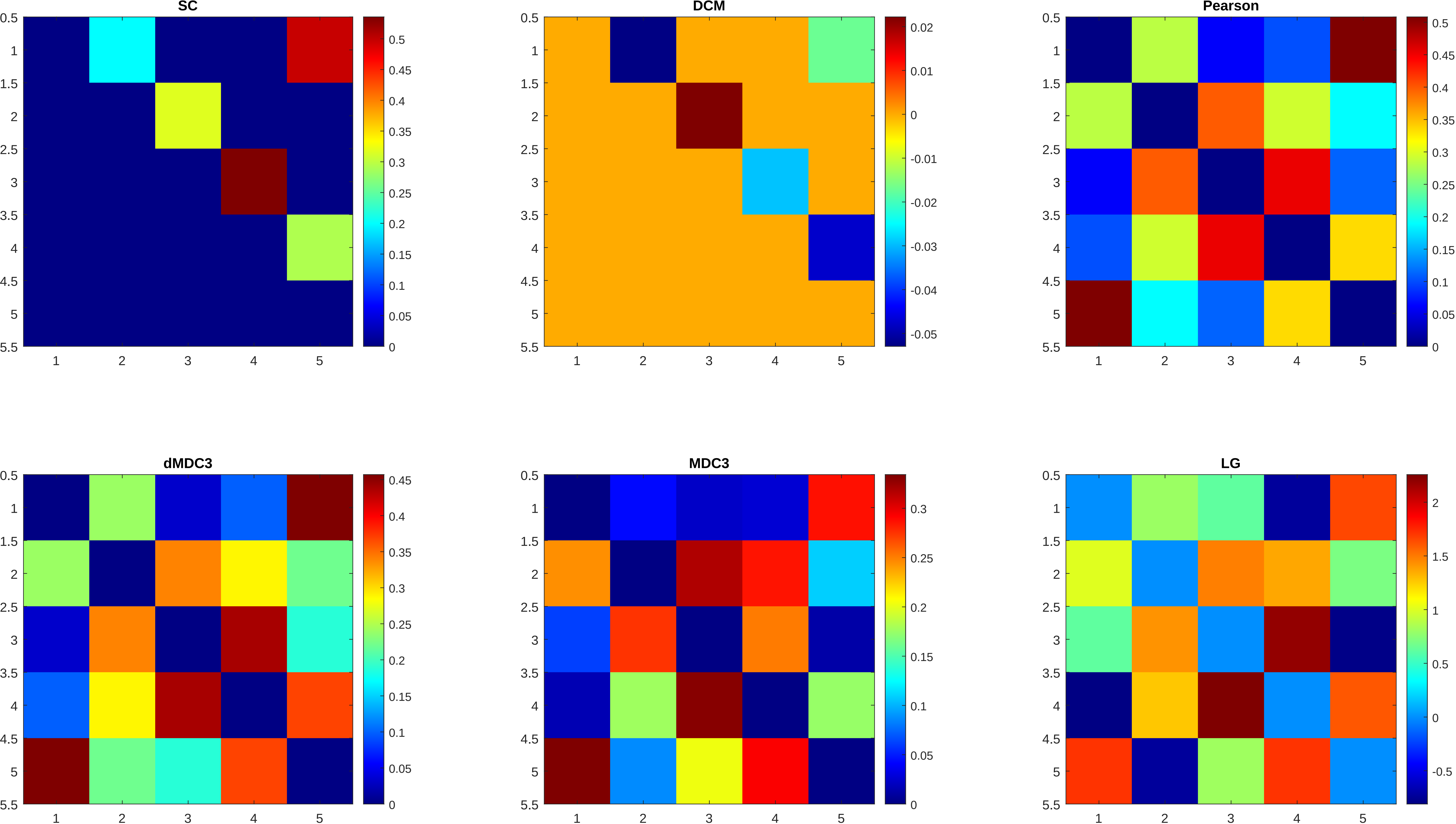
SC
DCM
Pearson
dMDC3
MDC3
LG
0.5
1
1.5
2
2.5
3
3.5
4
4.5
5
5.5
1
2
3
4
5
0.05
0.1
0.15
0.2
0.25
0.3
0.35
0.4
0.45
0.5
0
0.02
0.01
-0.01
-0.02
-0.03
-0.04
-0.05
2
1.5
1
0.5
-0.5

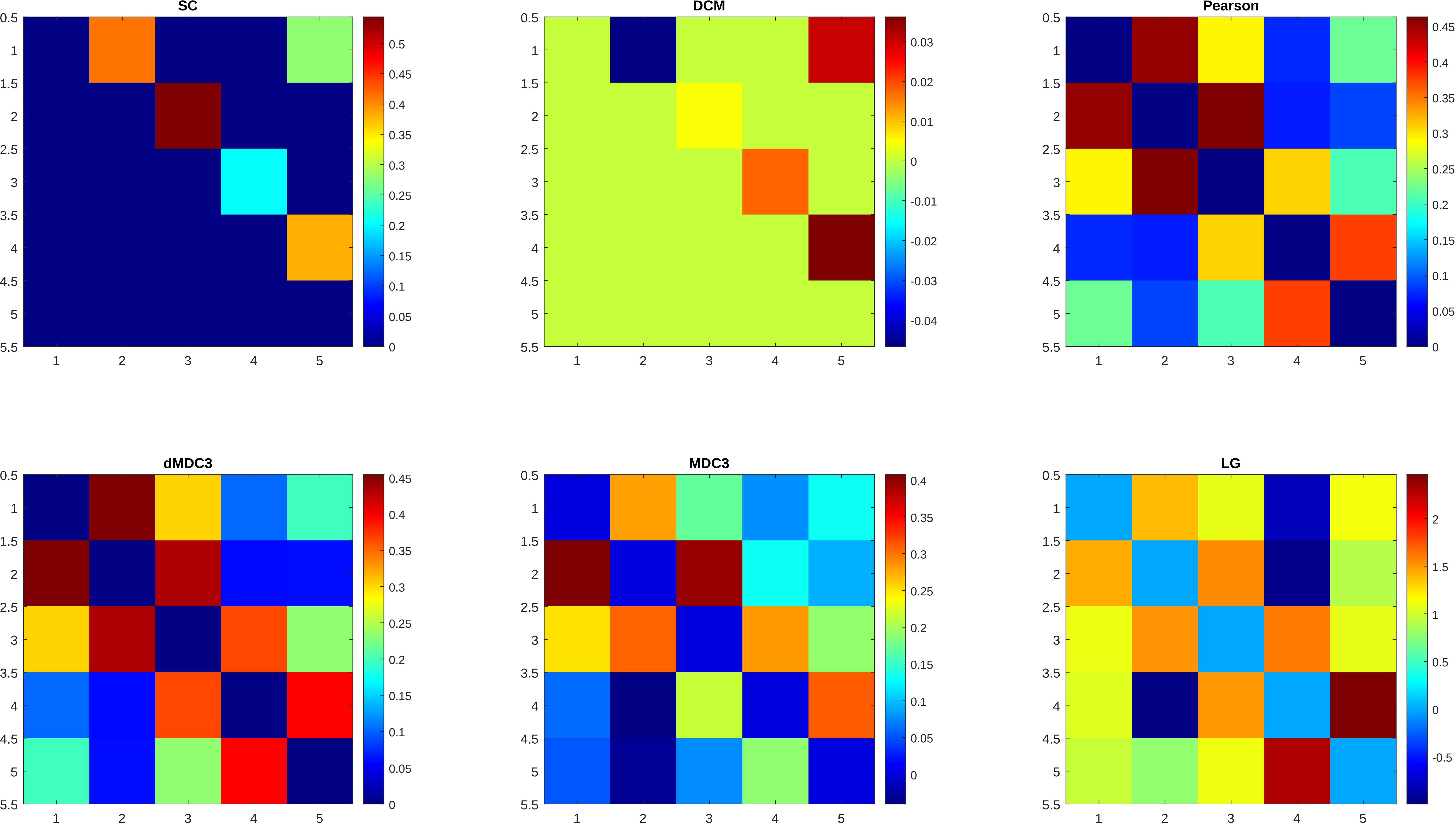
SC
DCM
Pearson
dMDC3
MDC3
LG

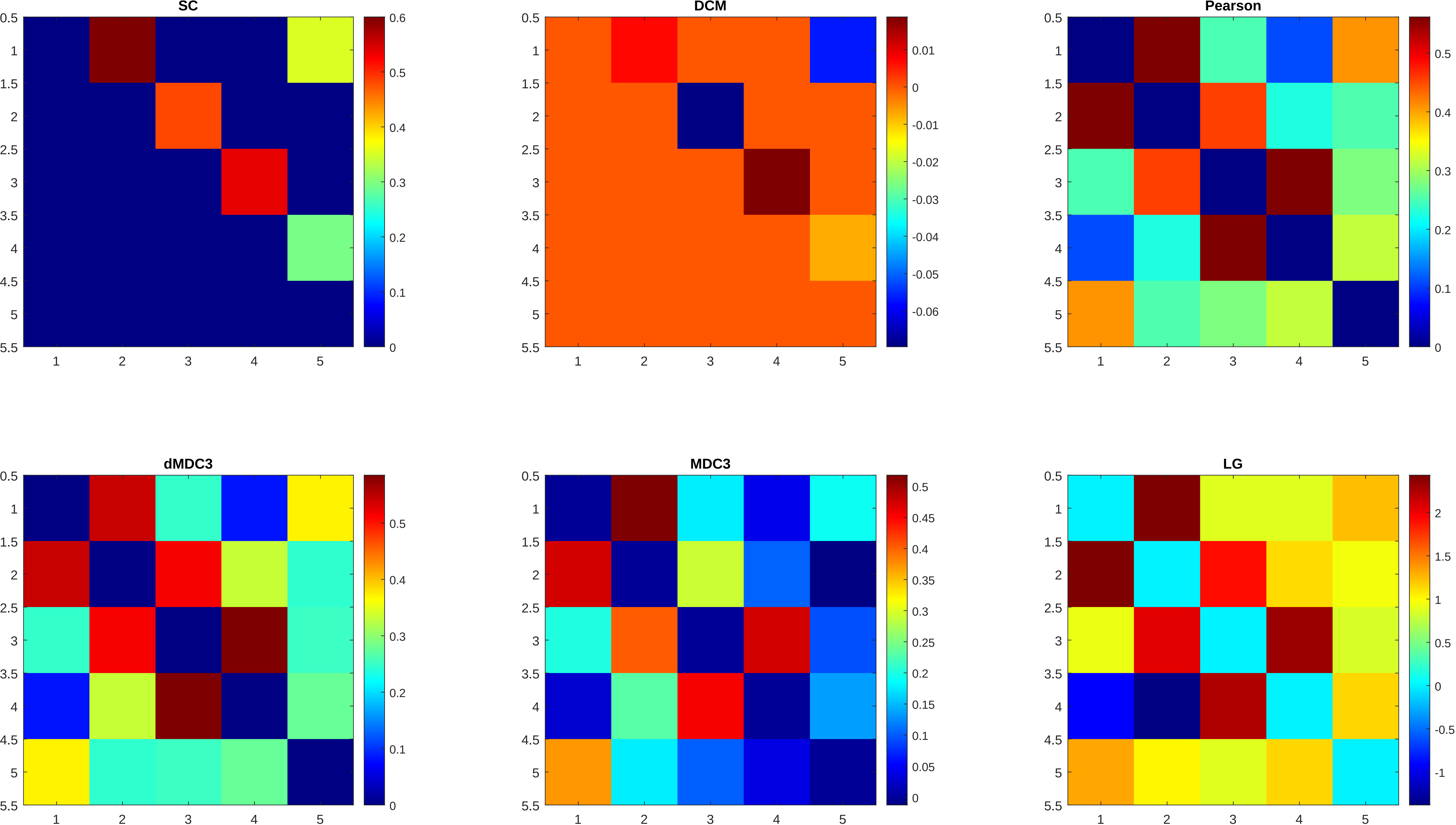
SC
DCM
Pearson
dMDC3
MDC3
LG
0.5
1
1.5
2
2.5
3
3.5
4
4.5
5
5.5
1
2
3
4
5
0.6
0.5
0.4
0.3
0.2
0.1
0
0.01
0
-0.01
-0.02
-0.03
-0.04
-0.05
-0.06
0.45
0.35
0.25
0.15
0.05
2
1.5
1
0.5
0
-0.5
-1

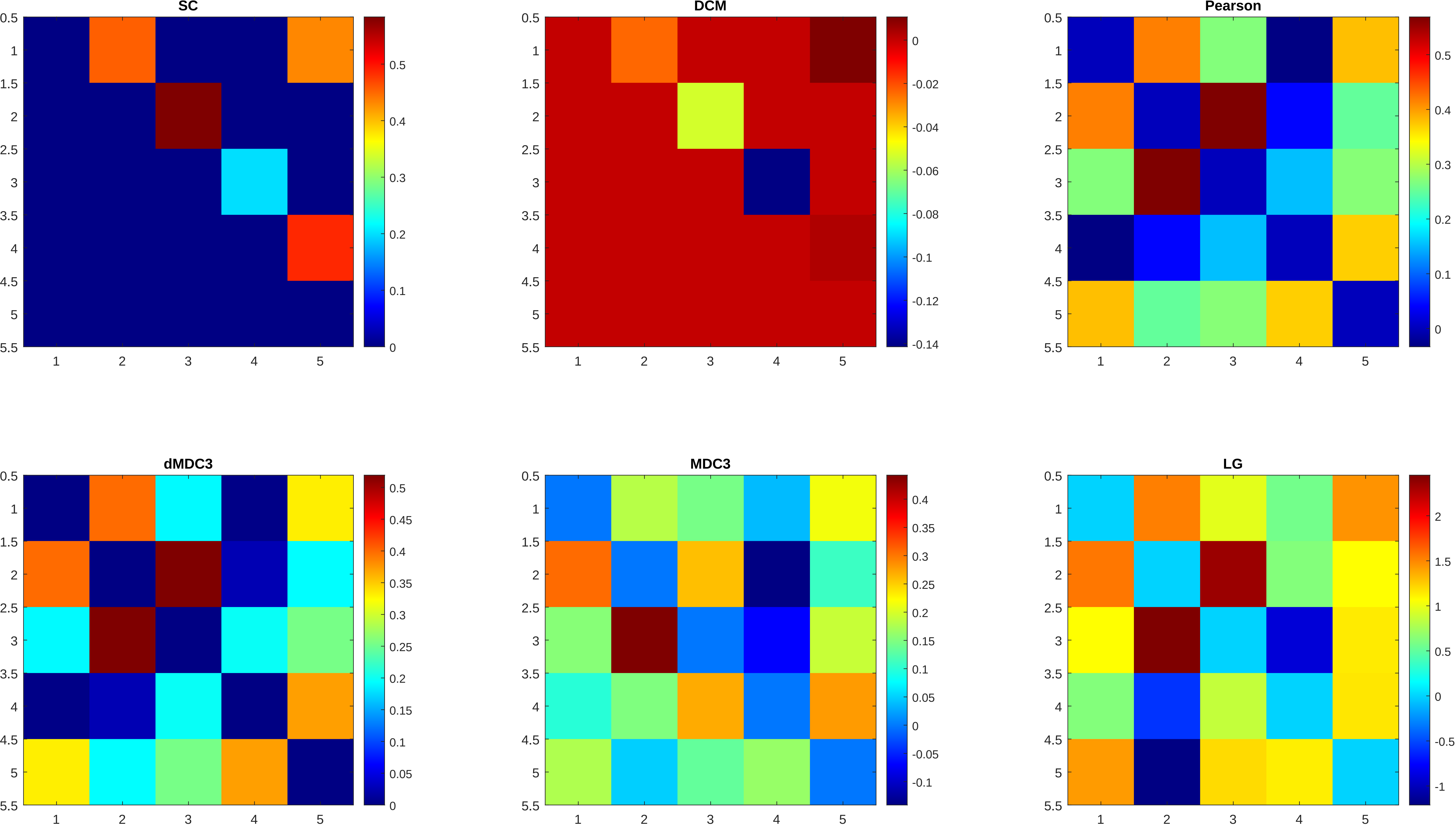

SC
DCM
Pearson
dMDC3
MDC3
LG
0.5
1
1.5
2
2.5
3
3.5
4
4.5
5
5.5
1 2 3 4 5

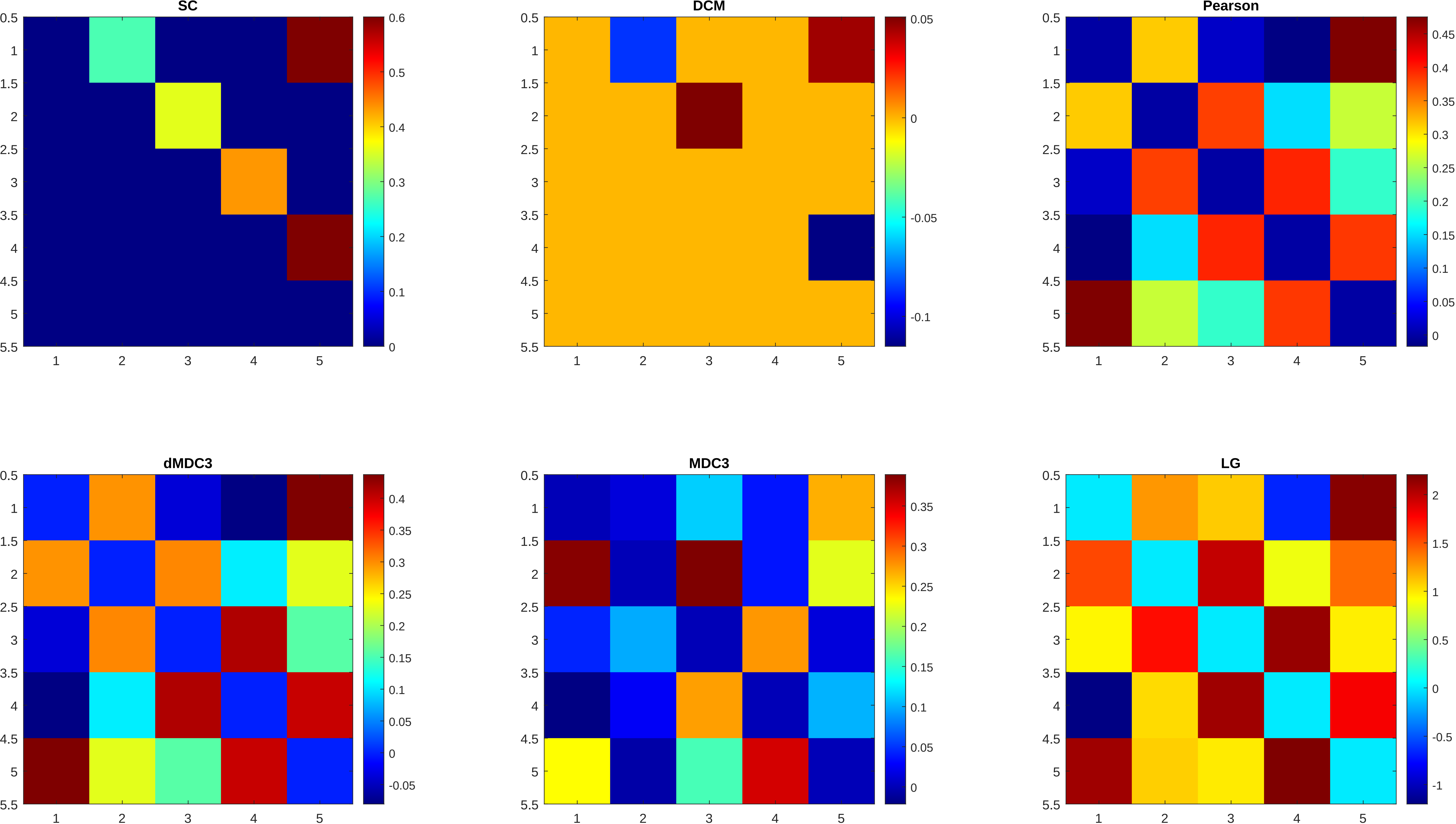

SC
DCM
Pearson
dMDC3
MDC3
LG
0.5
1
1.5
2
2.5
3
3.5
4
4.5
5
5.5
1 2 3 4 5
0 0.1 0.2 0.3 0.4 0.5 0.6
-0.1 -0.05 0 0.05
0 0.05 0.1 0.15 0.2 0.25 0.3 0.35 0.4 0.45
-0.05 0 0.05 0.1 0.15 0.2 0.25 0.3 0.35 0.4
0 0.05 0.1 0.15 0.2 0.25 0.3 0.35
-1 -0.5 0 0.5 1 1.5 2

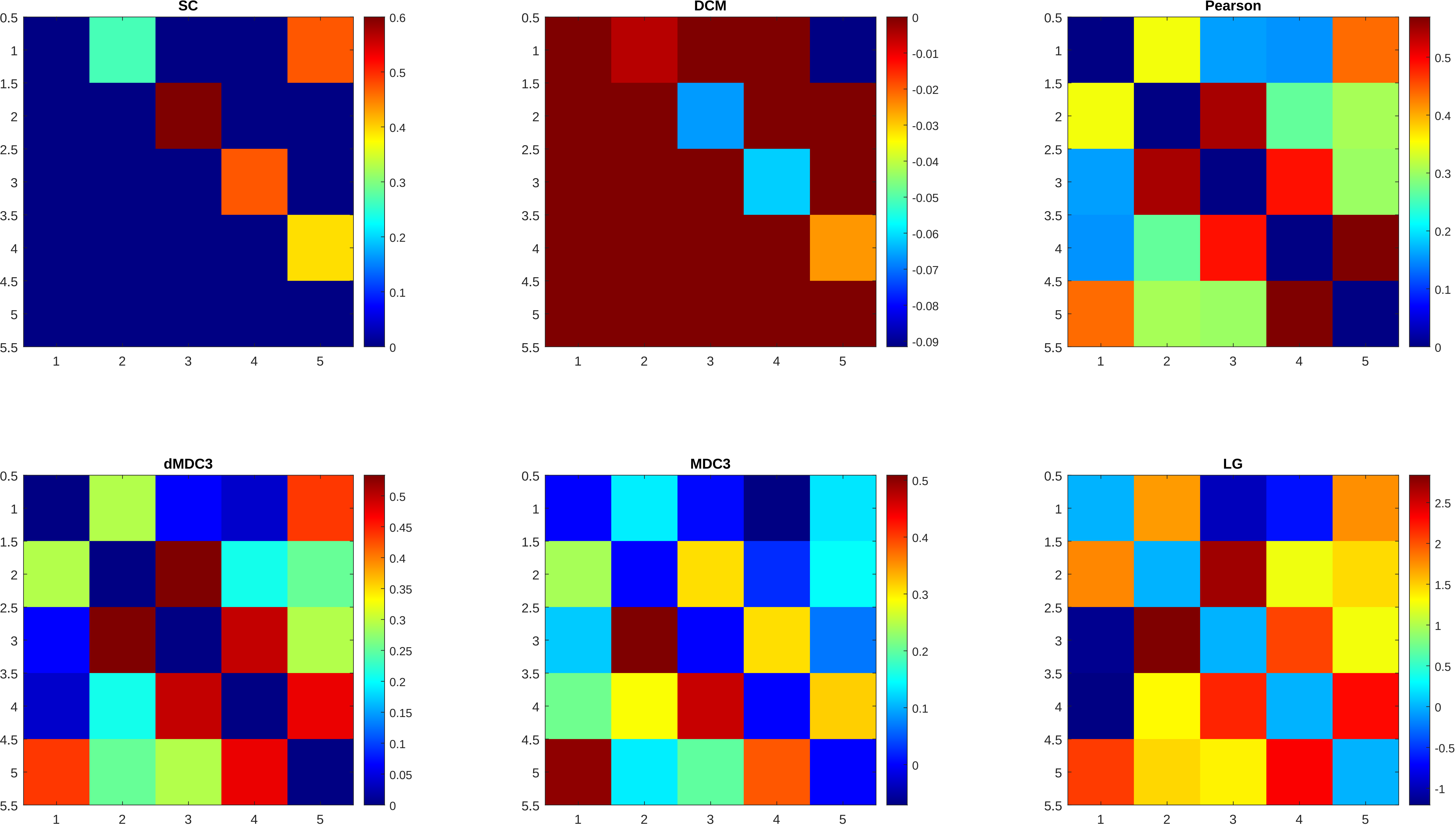
SC
DCM
Pearson
dMDC3
MDC3
LG

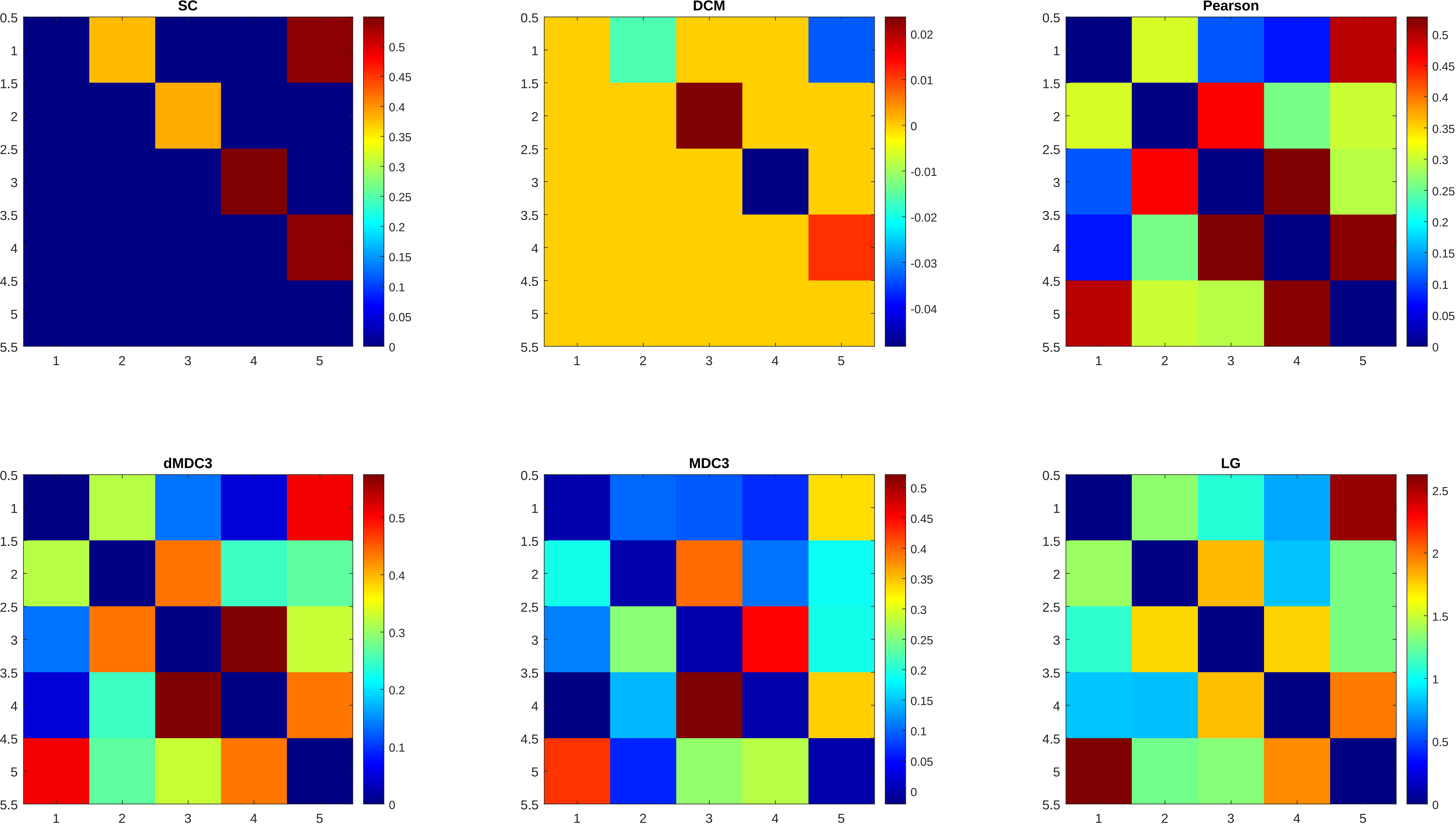
SC
DCM
Pearson
dMDC3
MDC3
LG

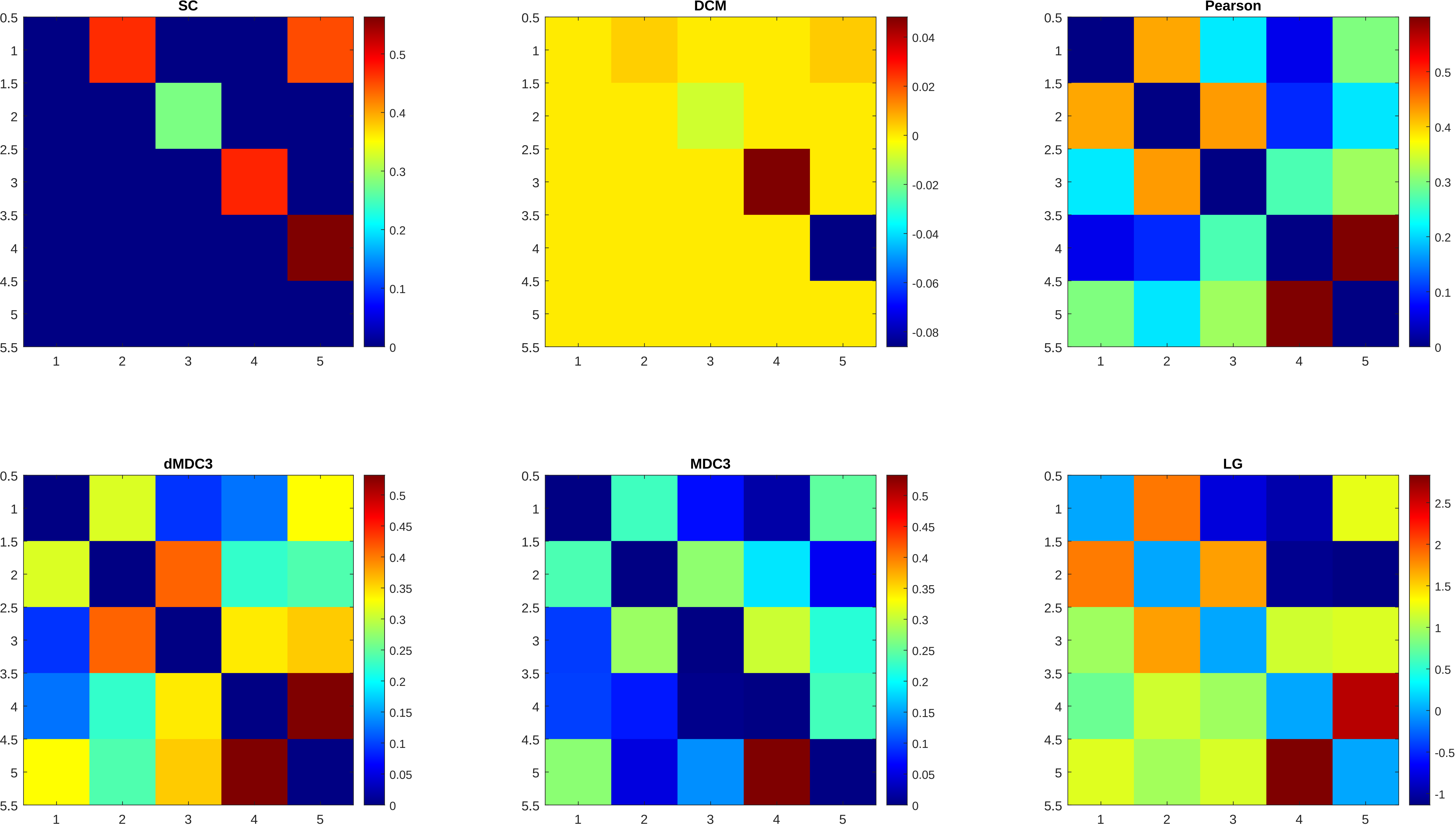
SC
DCM
Pearson
dMDC3
MDC3
LG

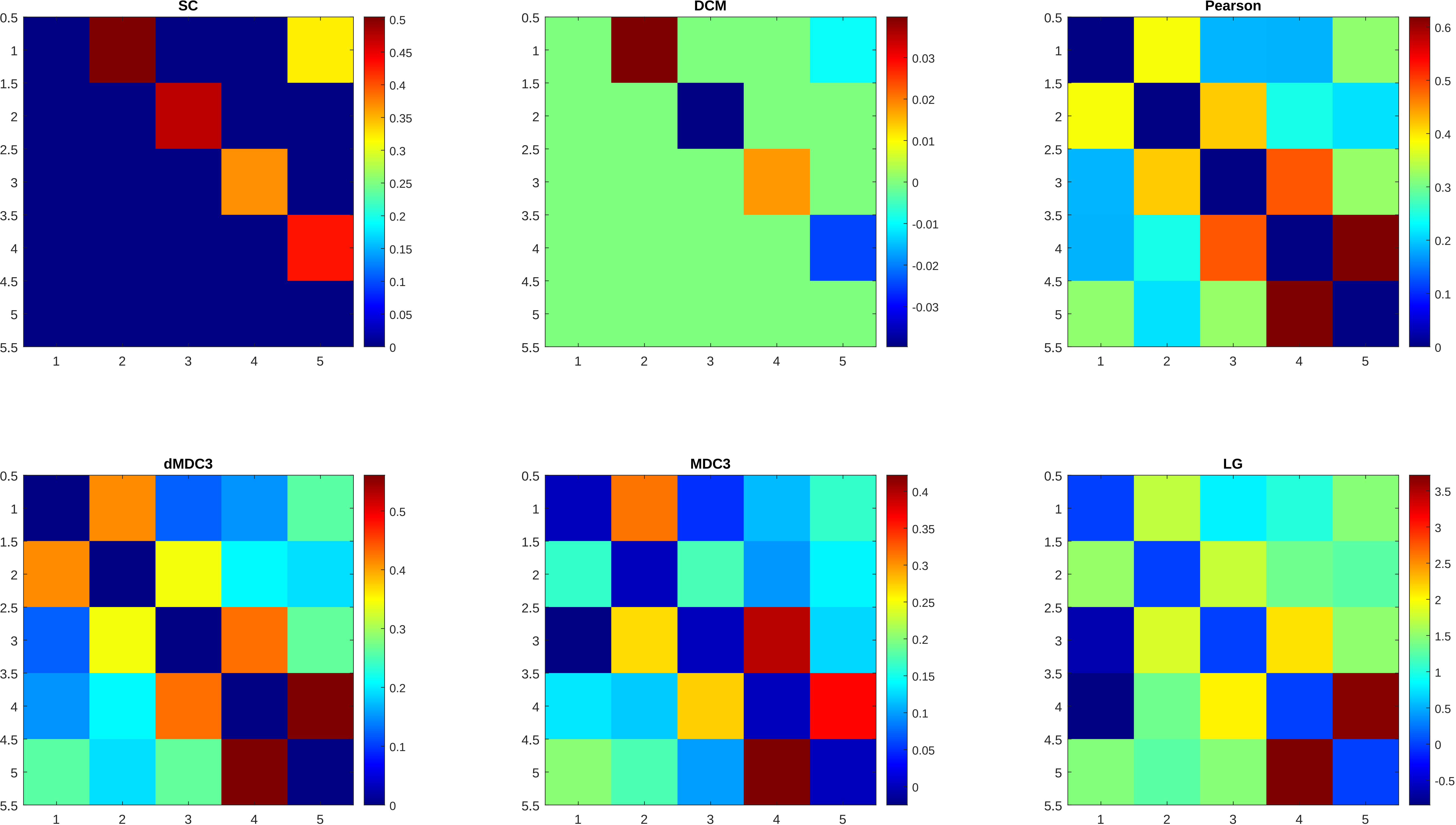
SC
DCM
Pearson
dMDC3
MDC3
LG
0.5 1 1.5 2 2.5 3 3.5 4 4.5 5 5.5
1 2 3 4 5
0 0.05 0.1 0.15 0.2 0.25 0.3 0.35 0.4 0.45 0.5
-0.03 -0.02 -0.01 0 0.01 0.02 0.03
0 0.1 0.2 0.3 0.4 0.5 0.6
0 0.1 0.2 0.3 0.4 0.5
0 0.05 0.1 0.15 0.2 0.25 0.3 0.35 0.4
-0.5 0 0.5 1 1.5 2 2.5 3 3.5

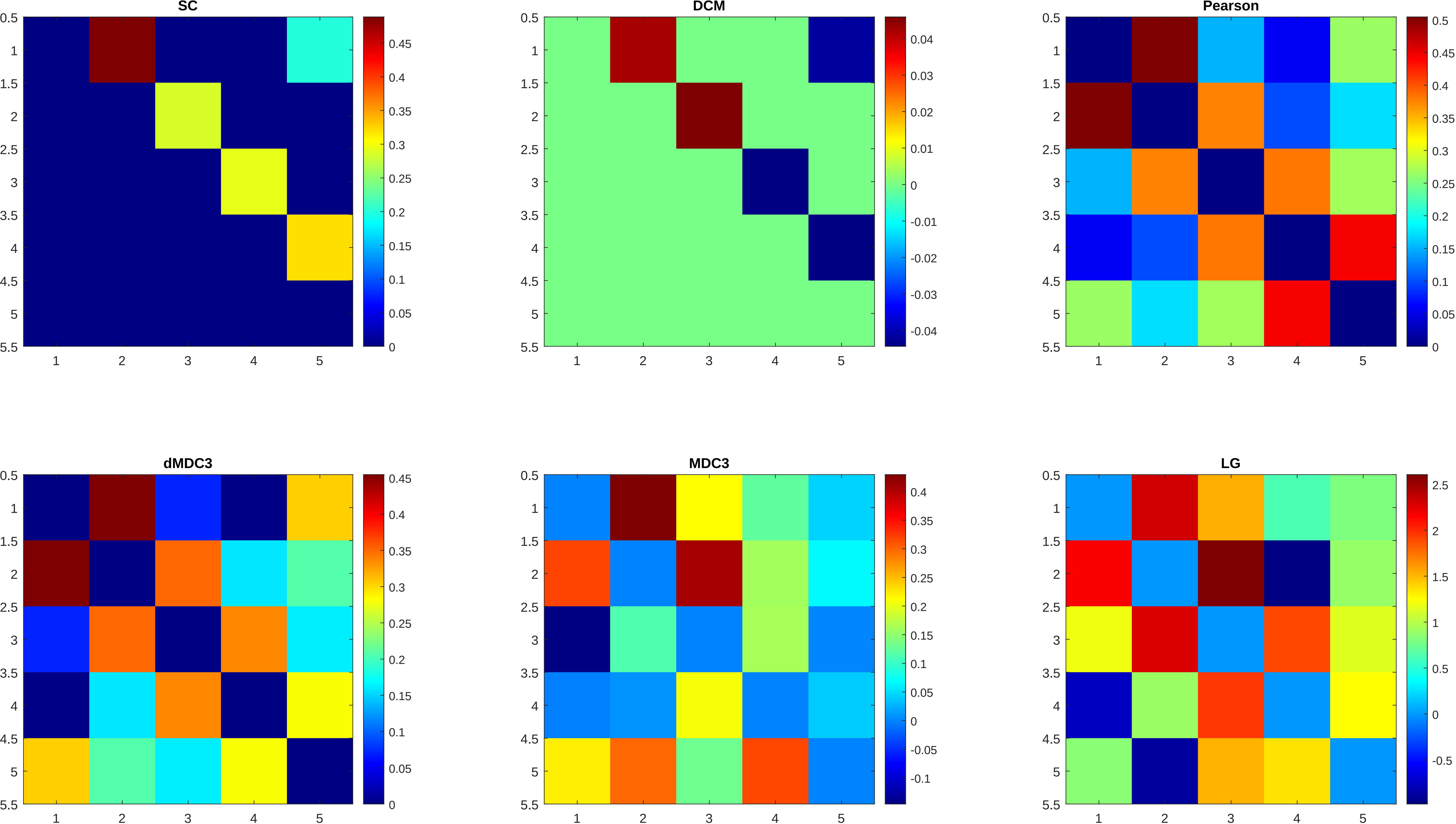
SC
DCM
Pearson
dMDC3
MDC3
LG

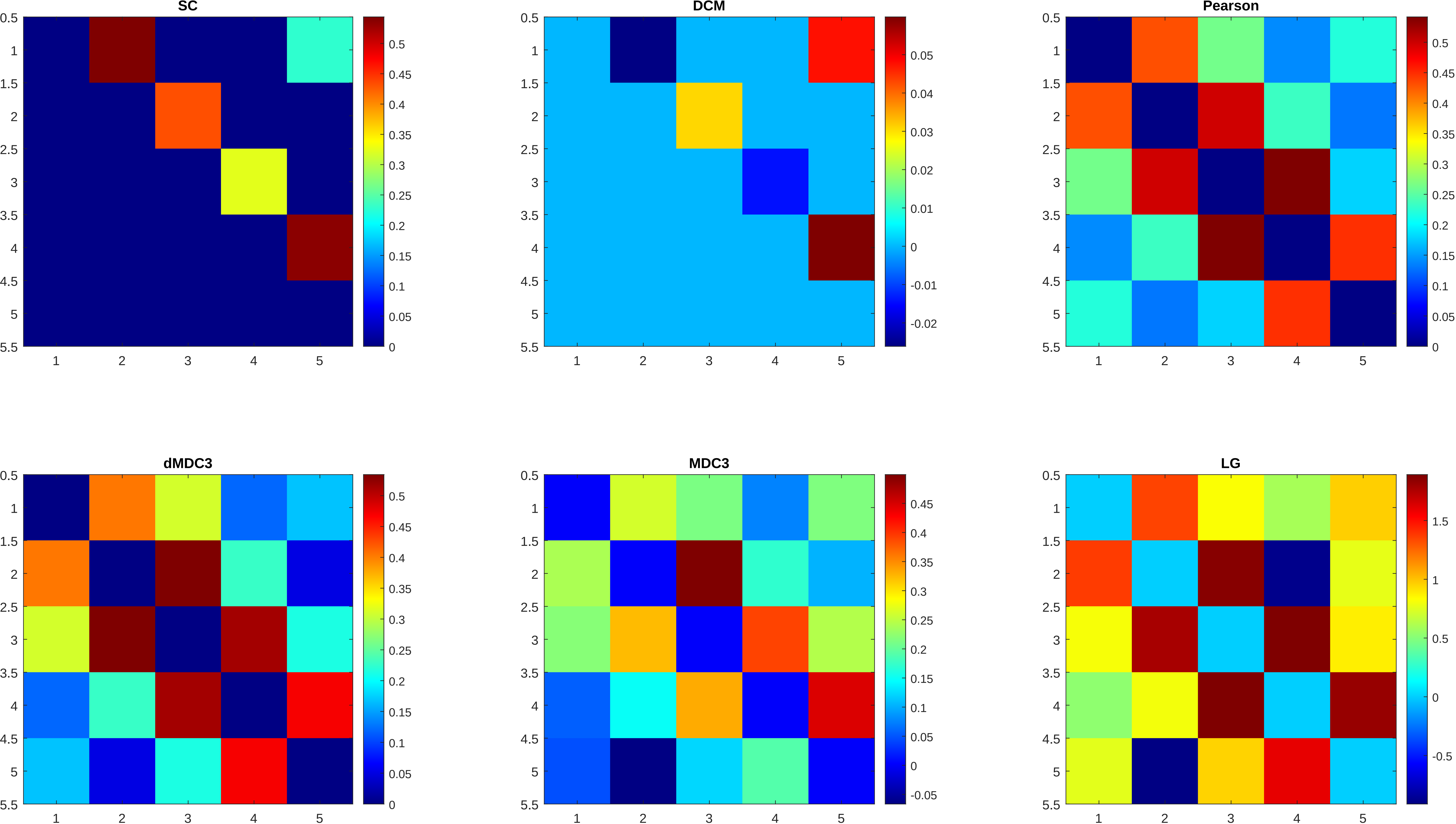
SC
DCM
Pearson
dMDC3
MDC3
LG

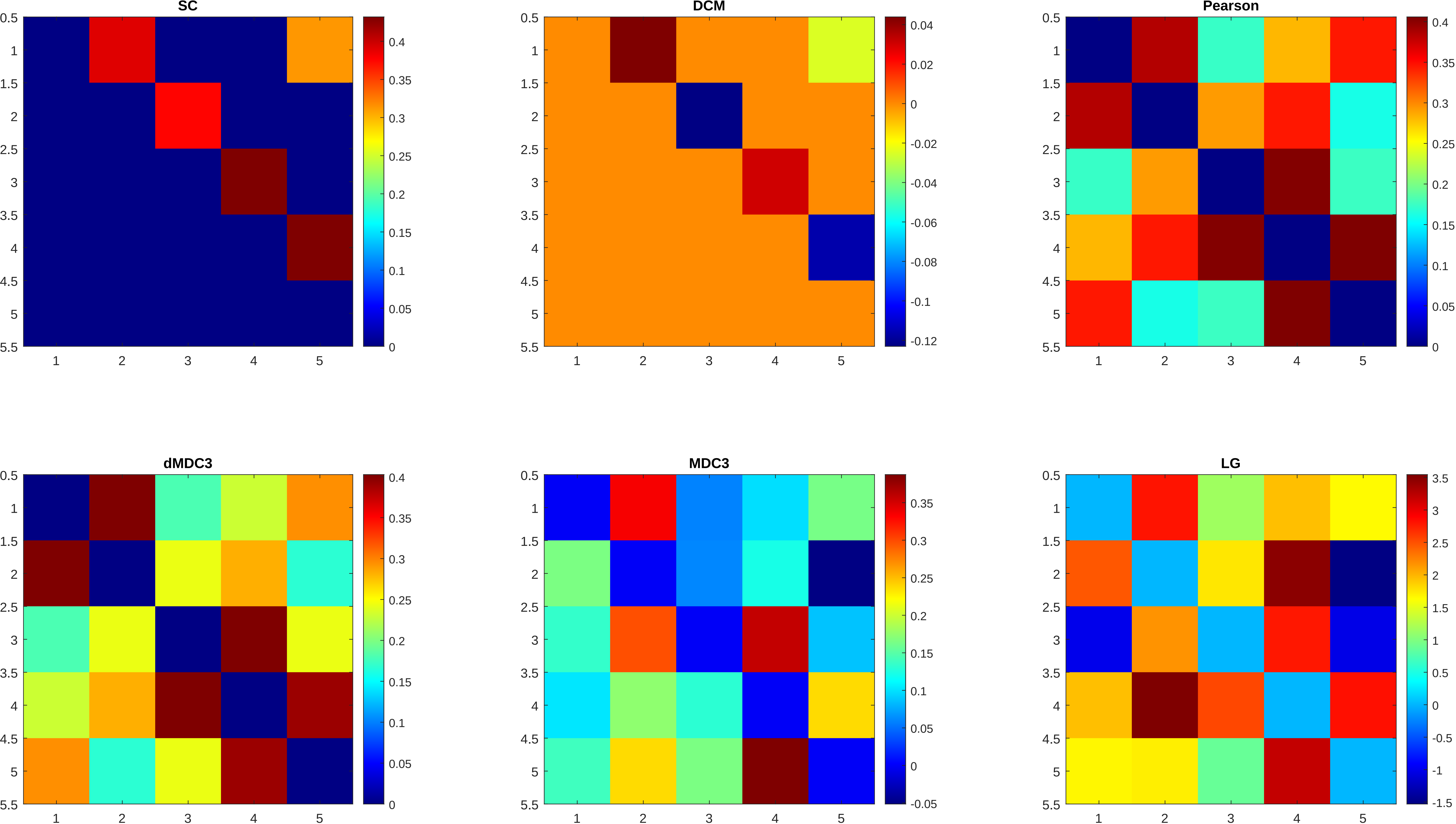
SC
DCM
Pearson
dMDC3
MDC3
LG
0.5 1 1.5 2 2.5 3 3.5 4 4.5 5 5.5
1 2 3 4 5
0 0.05 0.1 0.15 0.2 0.25 0.3 0.35 0.4
-0.12 -0.1 -0.08 -0.06 -0.04 -0.02 0 0.02 0.04
-0.05 0 0.05 0.1 0.15 0.2 0.25 0.3 0.35
-1.5 -1 -0.5 0 0.5 1 1.5 2 2.5 3 3.5

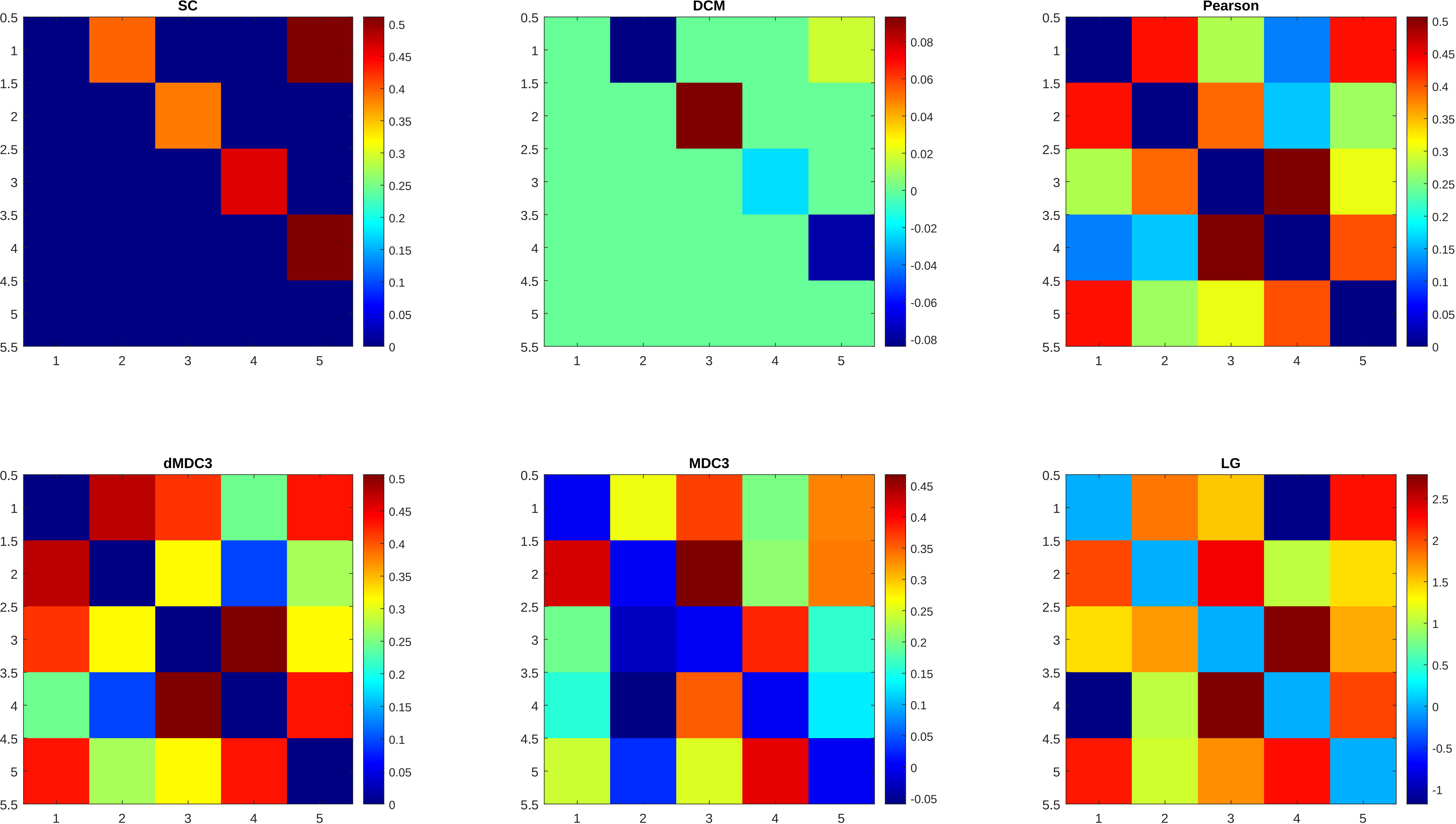
SC
DCM
Pearson
dMDC3
MDC3
LG
0.5 1 1.5 2 2.5 3 3.5 4 4.5 5 5.5
1 2 3 4 5
0 0.05 0.1 0.15 0.2 0.25 0.3 0.35 0.4 0.45 0.5
-0.08 -0.06 -0.04 -0.02 0 0.02 0.04 0.06 0.08
-0.05 0 0.05 0.1 0.15 0.2 0.25 0.3 0.35 0.4 0.45
-1 -0.5 0 0.5 1 1.5 2 2.5

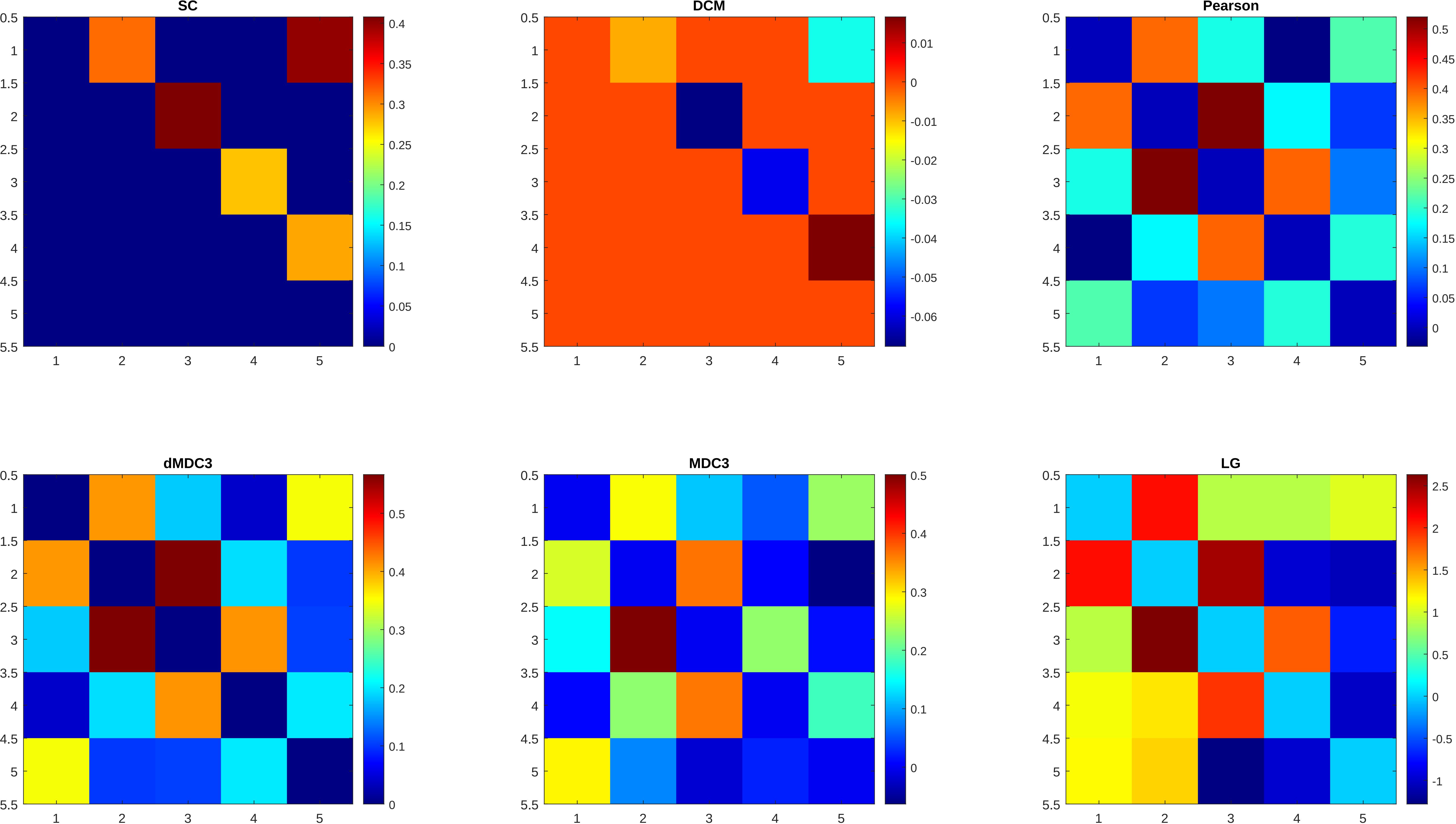
SC
DCM
Pearson
dMDC3
MDC3
LG
0.5 1 1.5 2 2.5 3 3.5 4 4.5 5 5.5
1 2 3 4 5
0 0.05 0.1 0.15 0.2 0.25 0.3 0.35 0.4
-0.06 -0.05 -0.04 -0.03 -0.02 -0.01 0 0.01
0 0.05 0.1 0.15 0.2 0.25 0.3 0.35 0.4 0.45 0.5
0 0.1 0.2 0.3 0.4 0.5
0 0.1 0.2 0.3 0.4 0.5
-1 -0.5 0 0.5 1 1.5 2 2.5

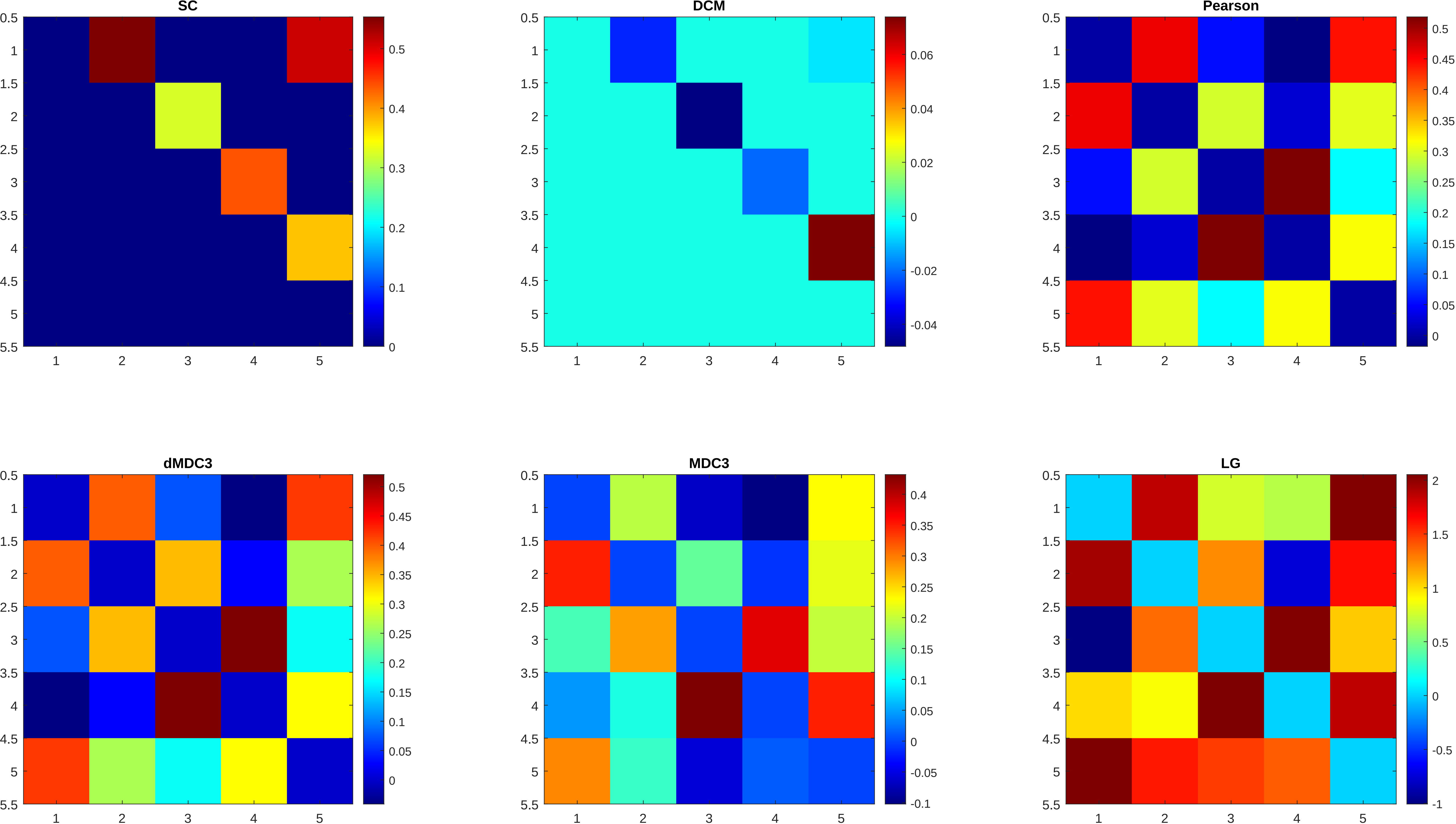
SC
0 0.1 0.2 0.3 0.4 0.5
DCM
-0.04 -0.02 0 0.02 0.04 0.06
Pearson
0 0.05 0.1 0.15 0.2 0.25 0.3 0.35 0.4 0.45 0.5
dMDC3
0 0.05 0.1 0.15 0.2 0.25 0.3 0.35 0.4 0.45 0.5
MDC3
-0.1 -0.05 0 0.05 0.1 0.15 0.2 0.25 0.3 0.35 0.4
LG
-1 -0.5 0 0.5 1 1.5 2
0.5 1 1.5 2 2.5 3 3.5 4 4.5 5 5.5
1 2 3 4 5

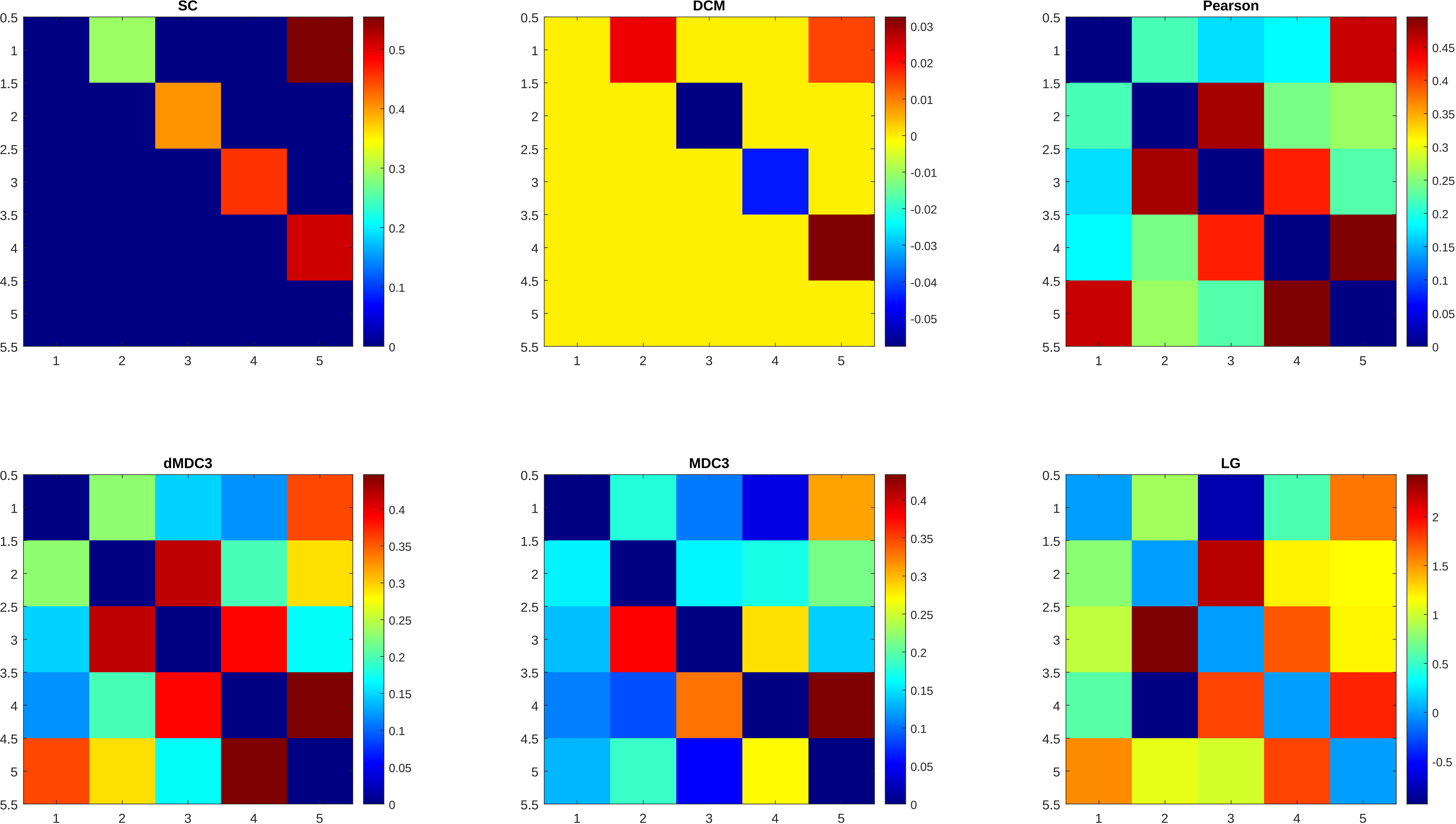
SC
DCM
Pearson
dMDC3
MDC3
LG

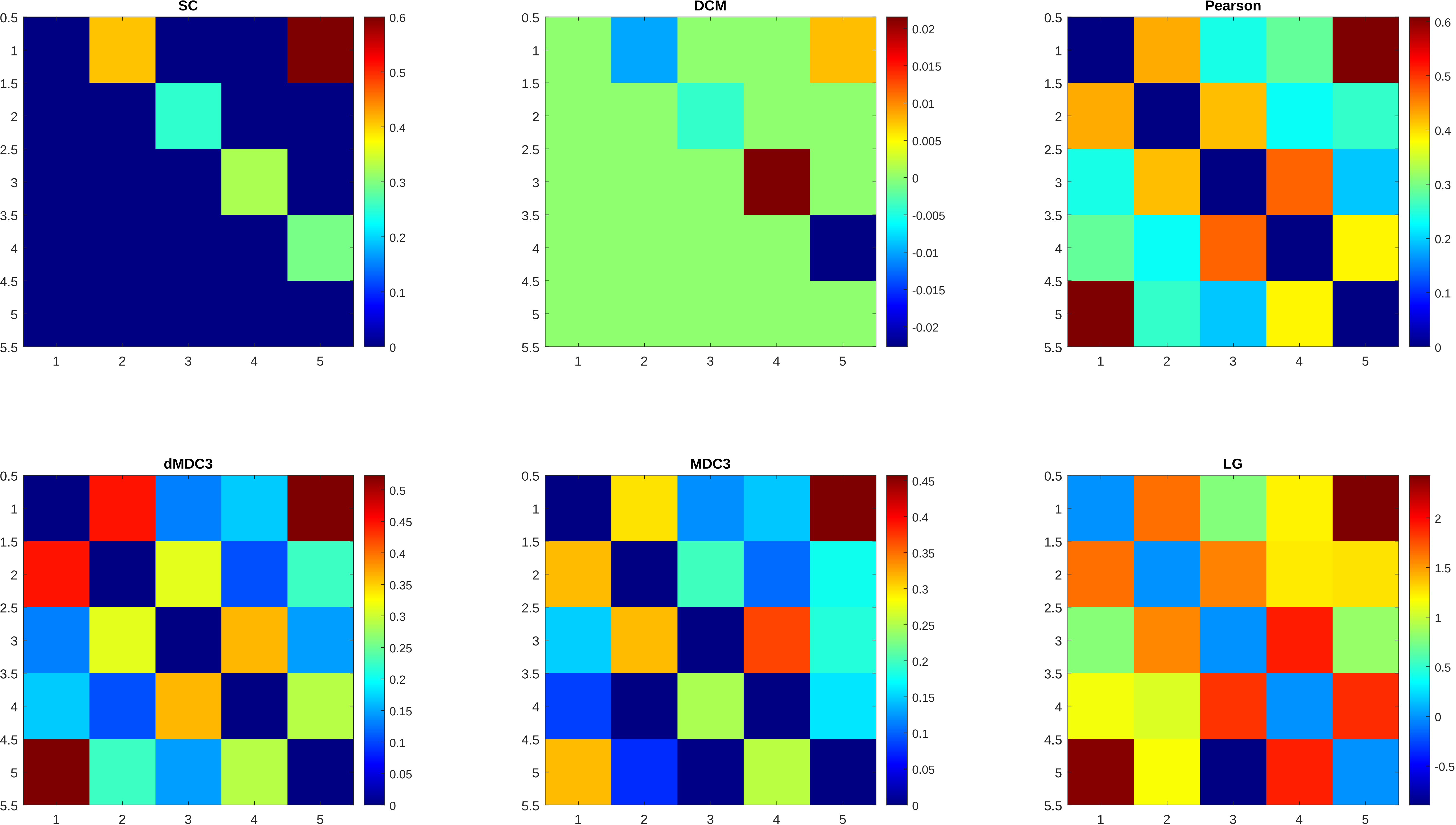
SC
DCM
Pearson
dMDC3
MDC3
LG
0.5 1 1.5 2 2.5 3 3.5 4 4.5 5 5.5
1 2 3 4 5
0 0.1 0.2 0.3 0.4 0.5 0.6
-0.02 -0.015 -0.01 -0.005 0 0.005 0.01 0.015 0.02
0 0.05 0.1 0.15 0.2 0.25 0.3 0.35 0.4 0.45 0.5
0 0.05 0.1 0.15 0.2 0.25 0.3 0.35 0.4 0.45
-0.5 0 0.5 1 1.5 2

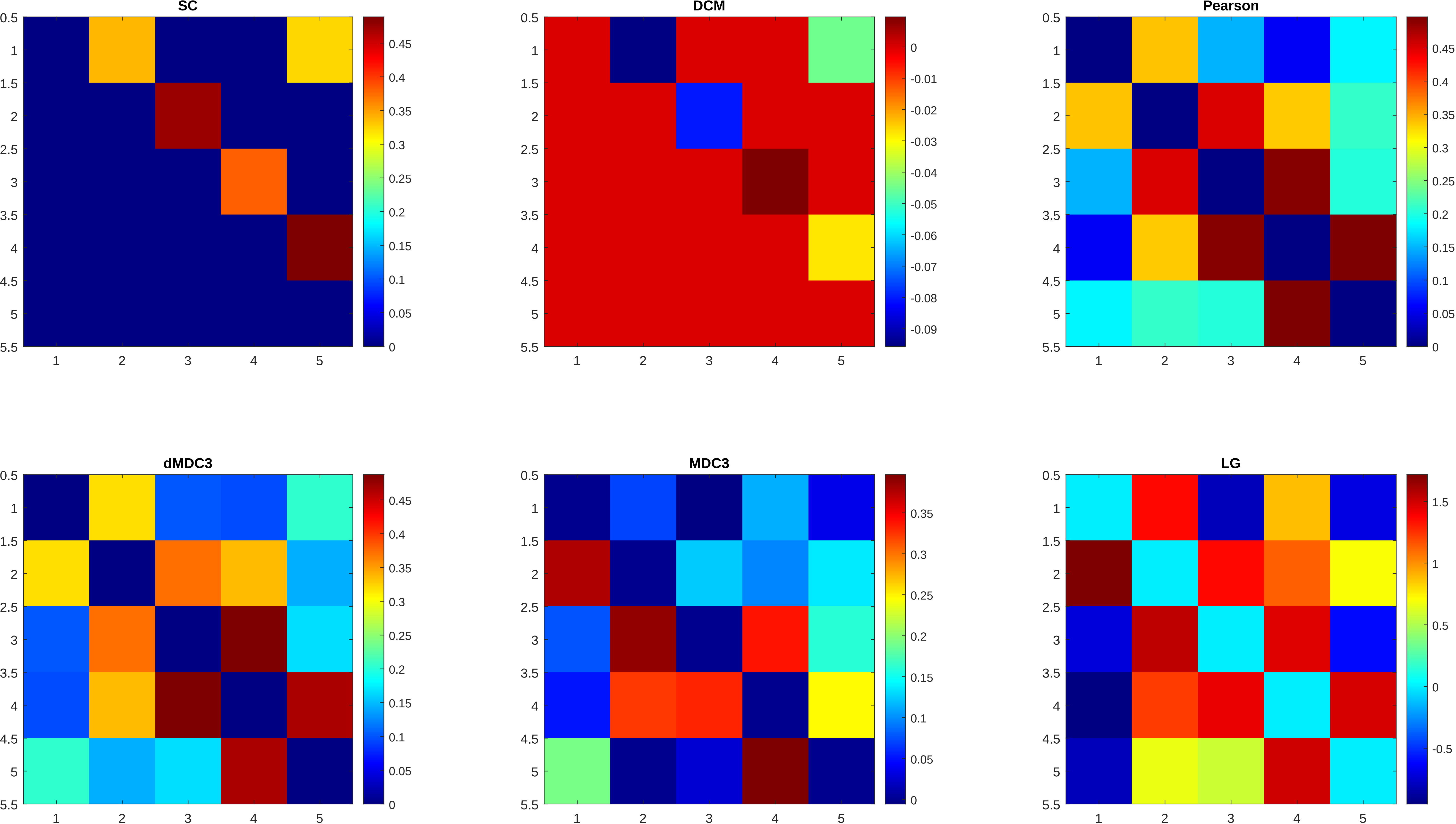
SC
DCM
Pearson
dMDC3
MDC3
LG

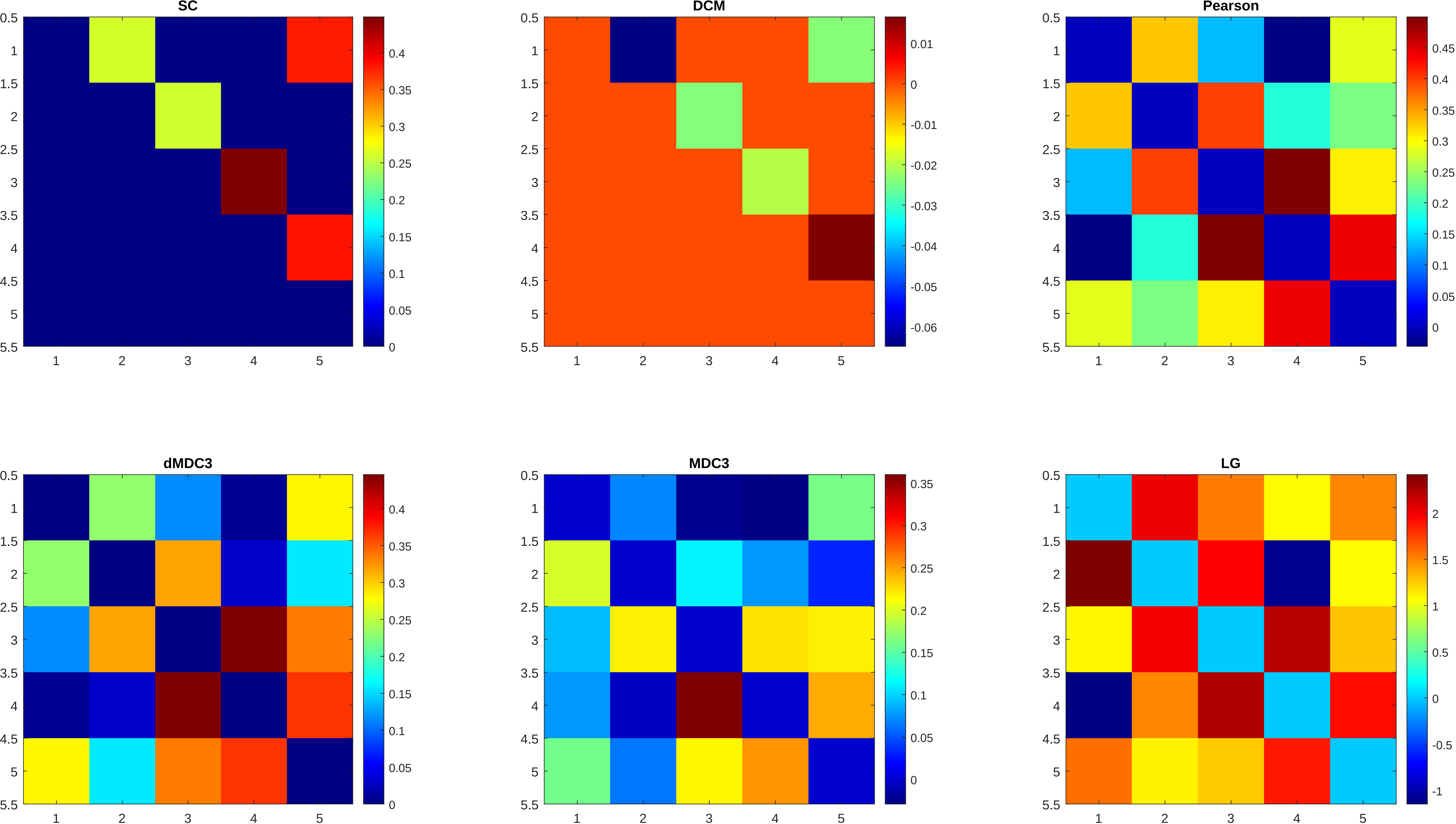
SC
DCM
Pearson
dMDC3
MDC3
LG
0.5 1 1.5 2 2.5 3 3.5 4 4.5 5 5.5
1 2 3 4 5
0 0.05 0.1 0.15 0.2 0.25 0.3 0.35 0.4
-0.06 -0.05 -0.04 -0.03 -0.02 -0.01 0 0.01
0 0.05 0.1 0.15 0.2 0.25 0.3 0.35 0.4 0.45
0 0.05 0.1 0.15 0.2 0.25 0.3 0.35
-1 -0.5 0 0.5 1 1.5 2

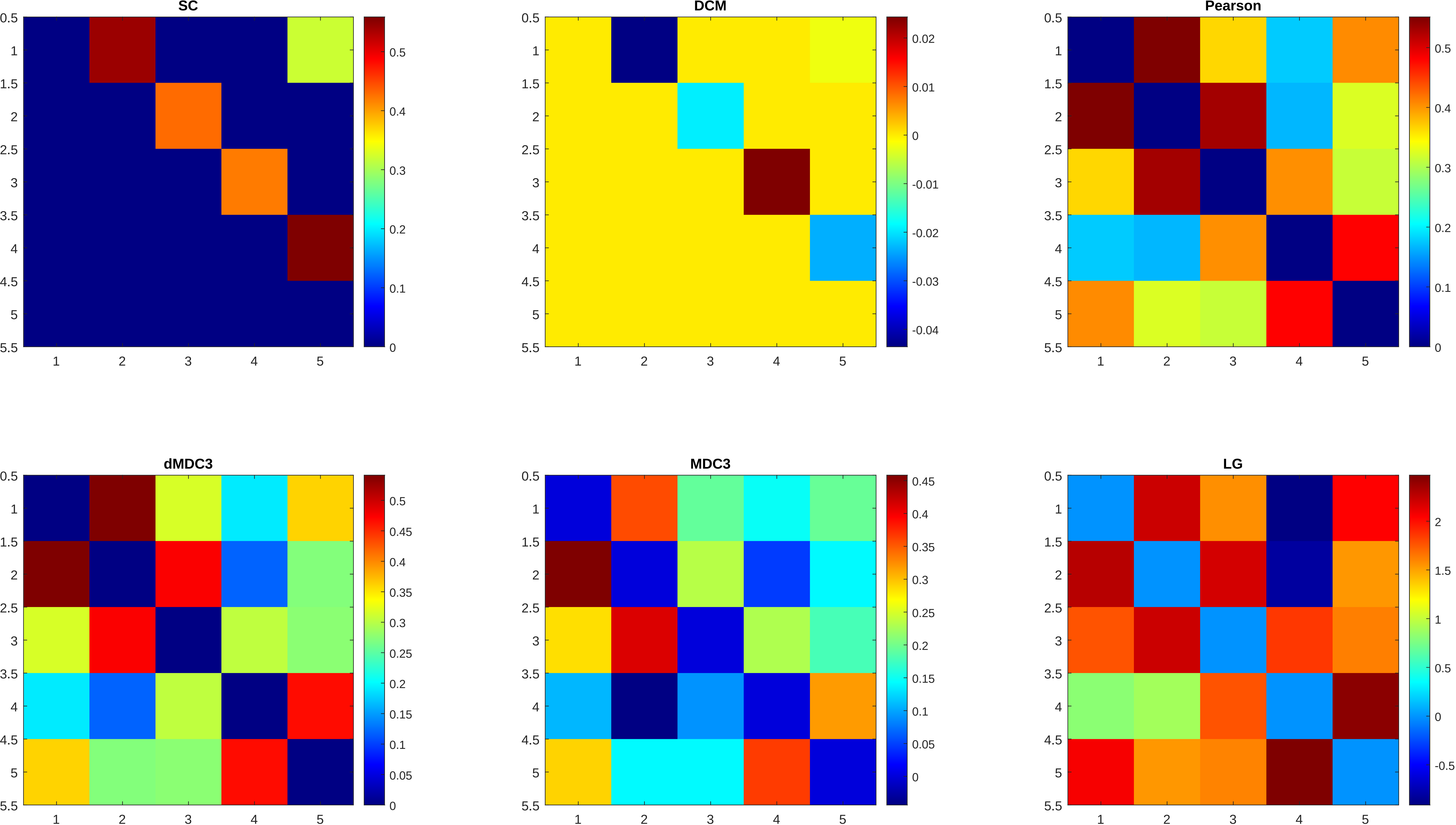
SC
0.5
1
1.5
2
2.5
3
3.5
4
4.5
5
5.5
1 2 3 4 5
0 0.1 0.2 0.3 0.4 0.5
DCM
-0.04 -0.03 -0.02 -0.01 0 0.01 0.02
Pearson
0 0.1 0.2 0.3 0.4 0.5
dMDC3
0 0.05 0.1 0.15 0.2 0.25 0.3 0.35 0.4 0.45 0.5
MDC3
0 0.05 0.1 0.15 0.2 0.25 0.3 0.35 0.4 0.45
LG
-0.5 0 0.5 1 1.5 2

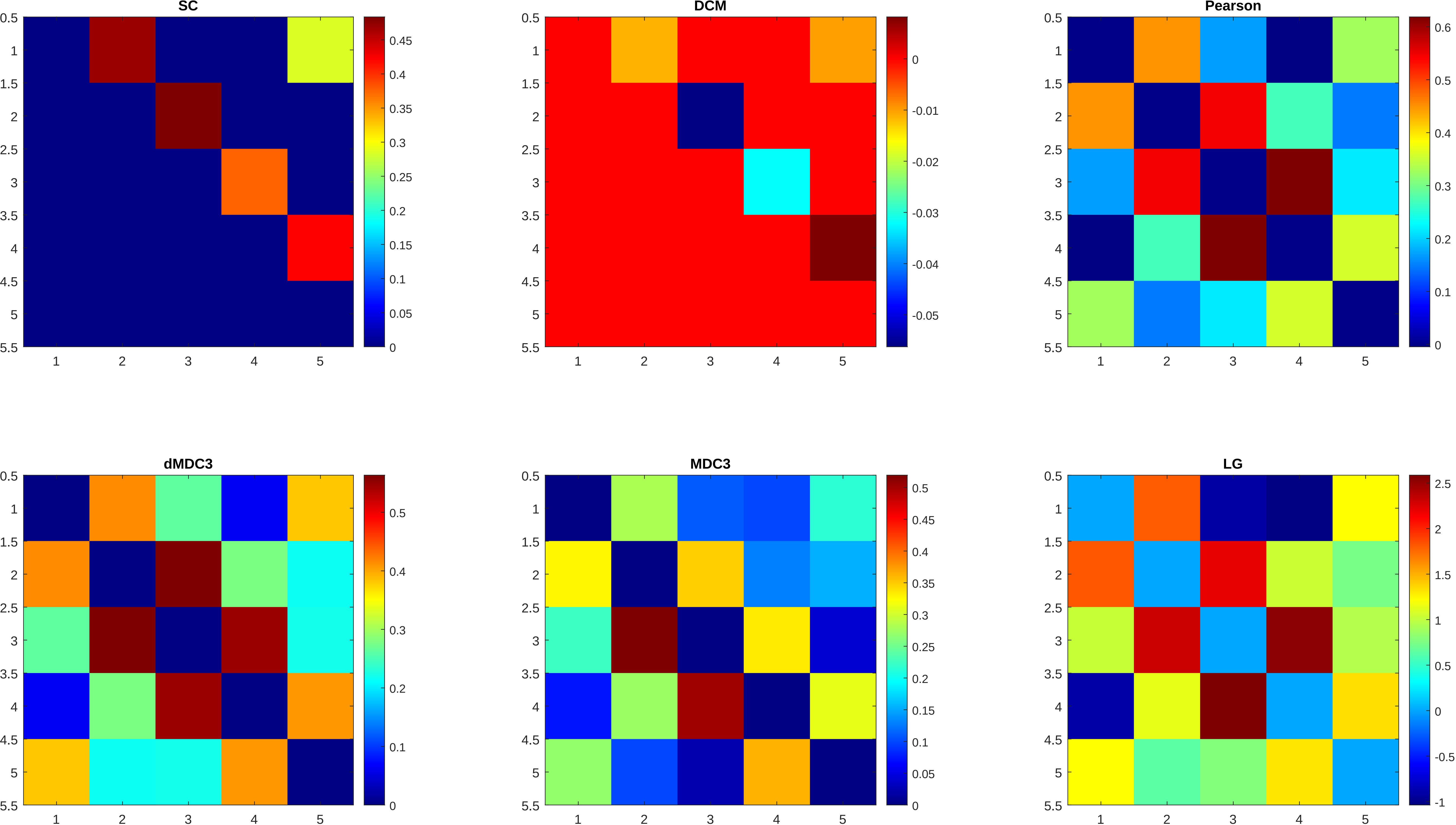
SC
DCM
Pearson
dMDC3
MDC3
LG

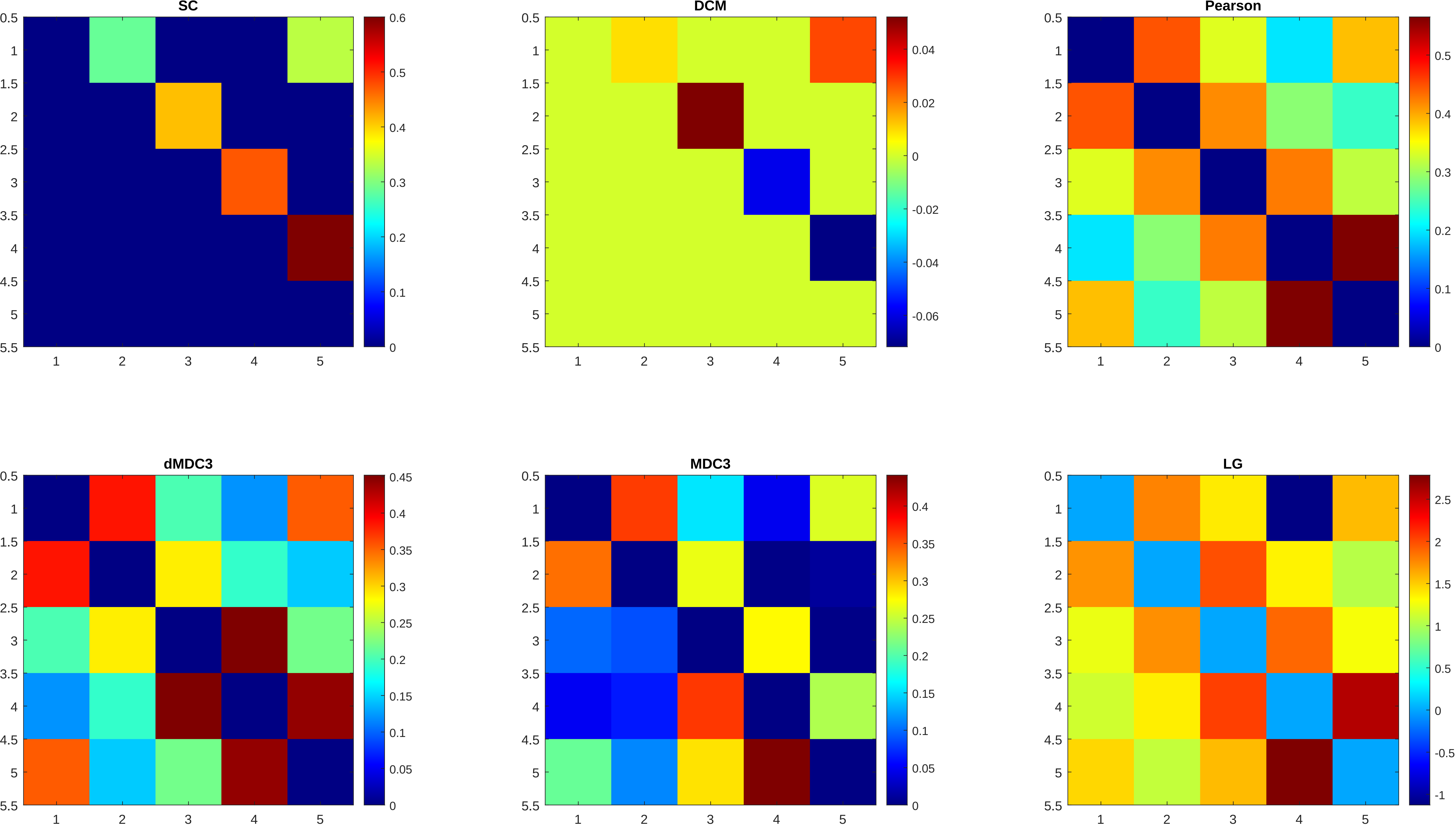

SC
DCM
Pearson
dMDC3
MDC3
LG

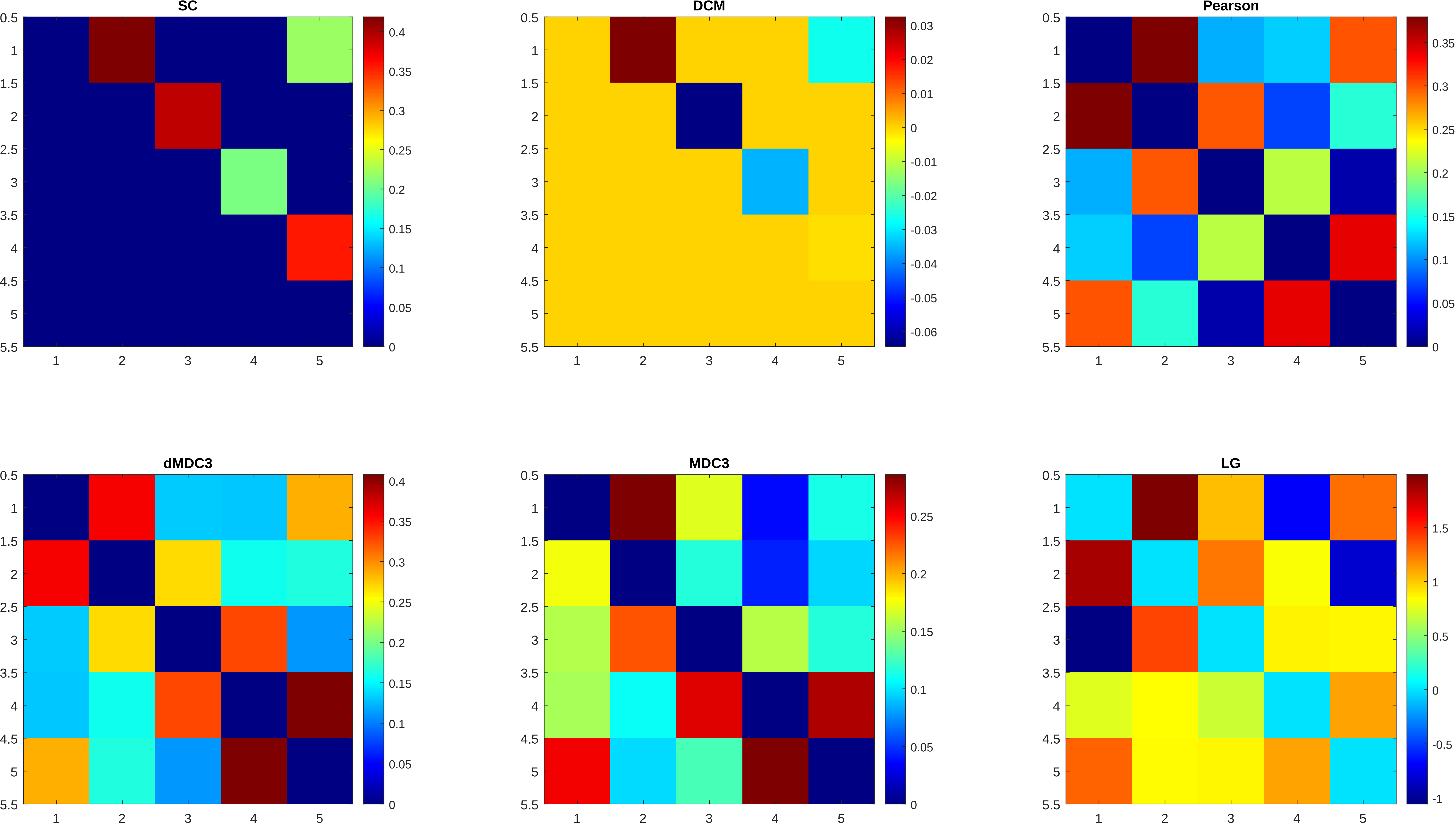

SC
DCM
Pearson
dMDC3
MDC3
LG

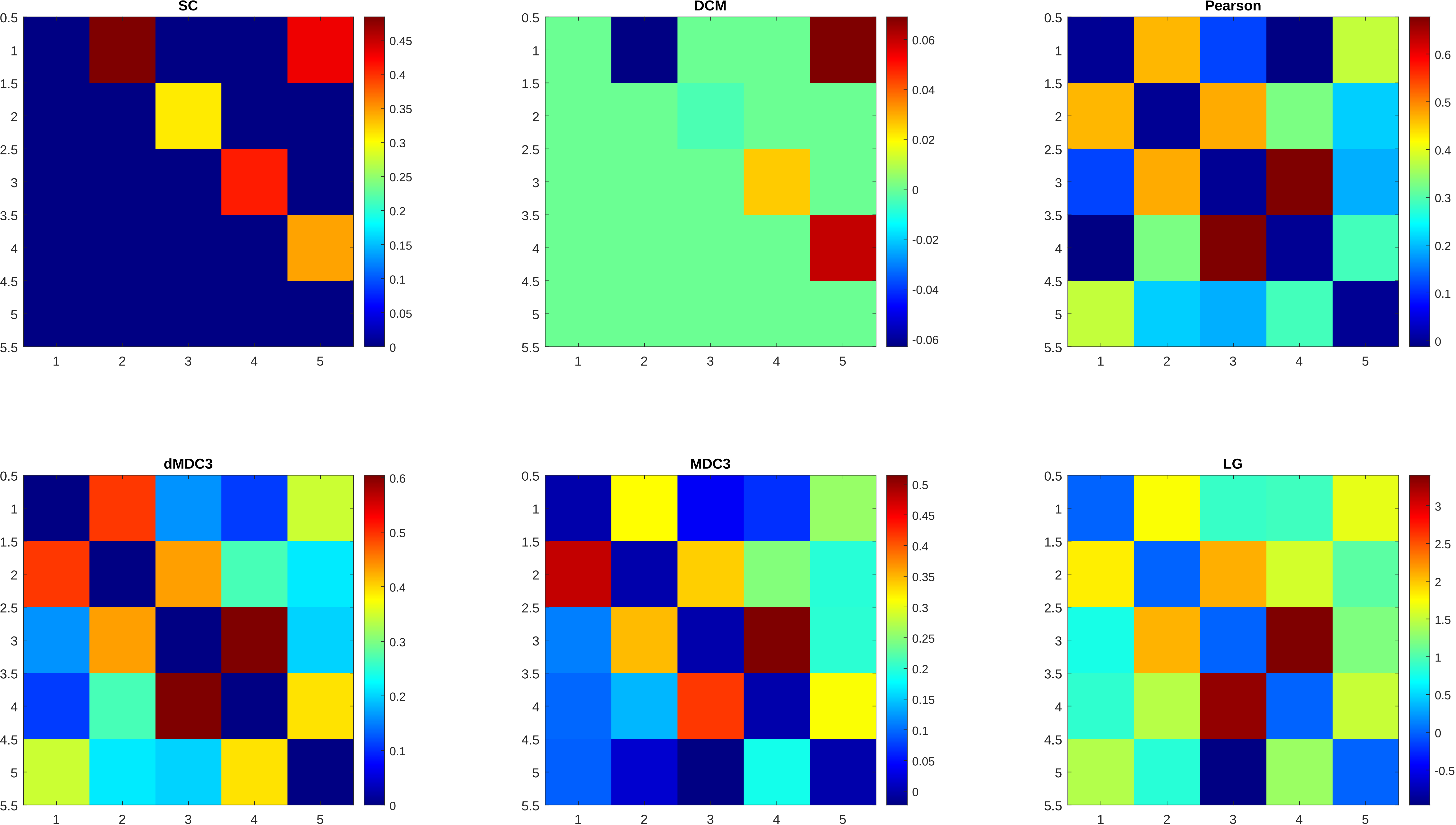
SC
DCM
Pearson
dMDC3
MDC3
LG

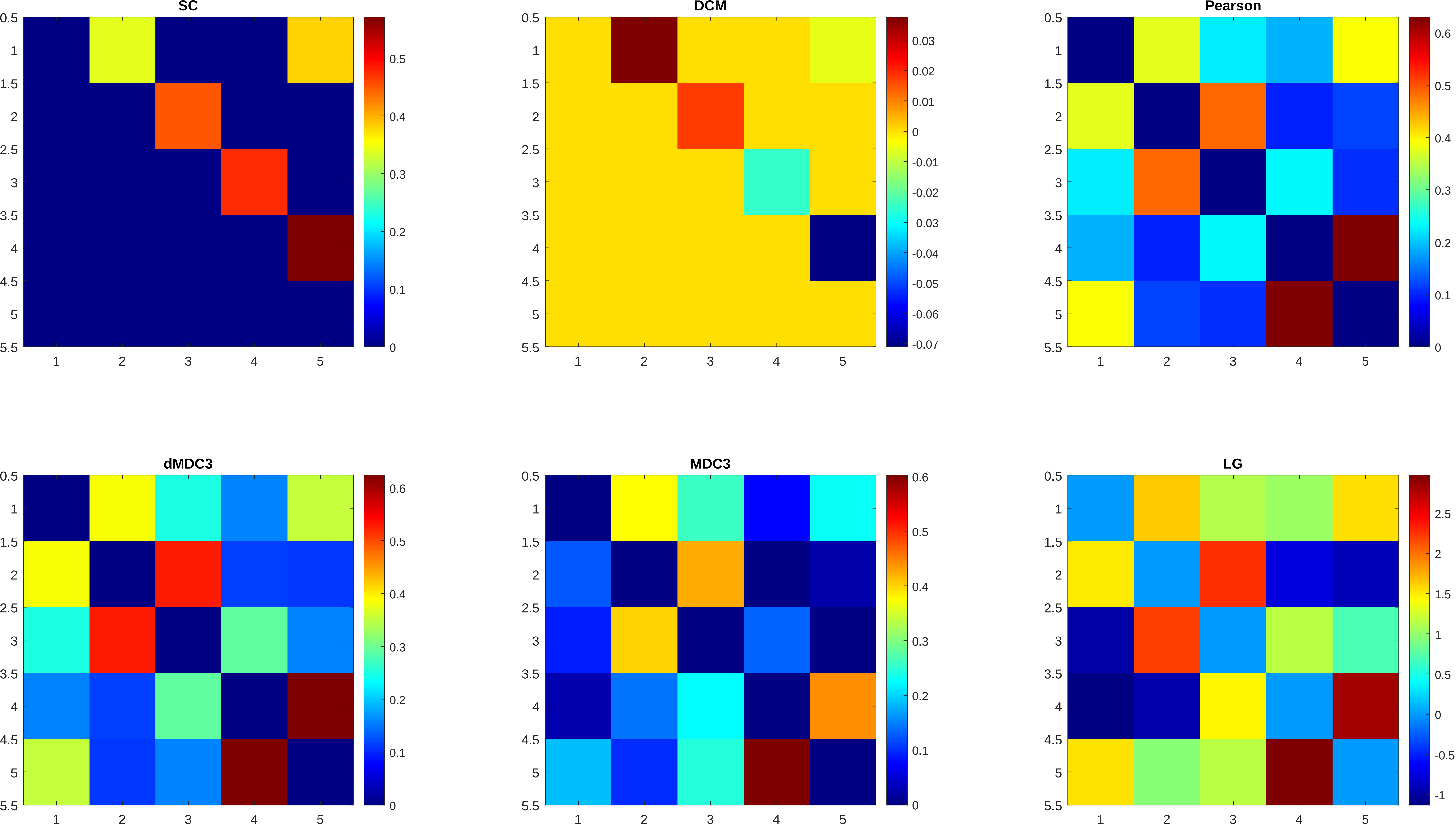

SC
DCM
Pearson
dMDC3
MDC3
LG
0.5
1
1.5
2
2.5
3
3.5
4
4.5
5
5.5
0
0.1
0.2
0.3
0.4
0.5
0.6
0.03
0.02
0.01
-0.01
-0.02
-0.03
-0.04
-0.05
-0.06
-0.07
2.5
2
1.5
-0.5
-1

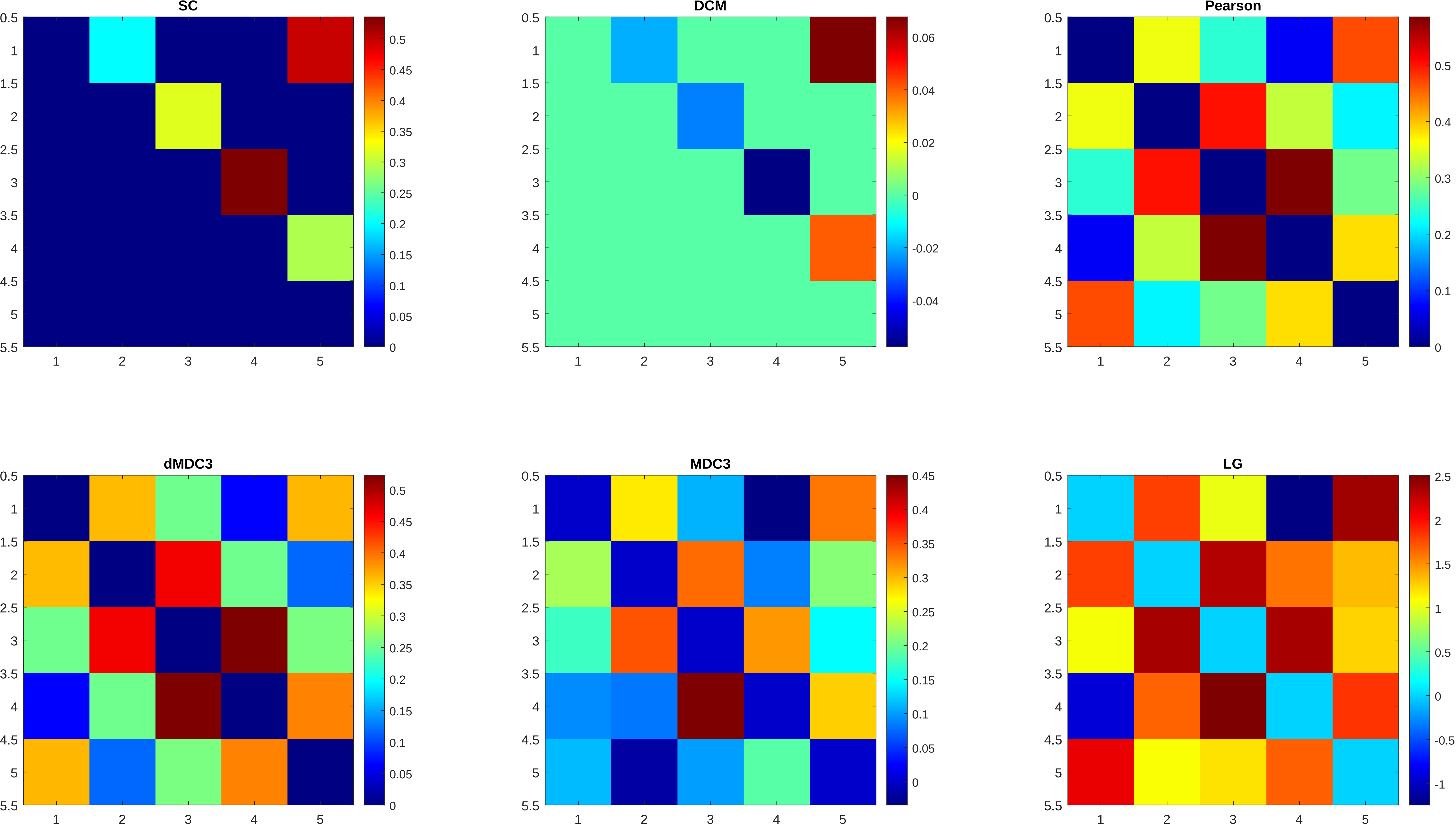

SC
DCM
Pearson
dMDC3
MDC3
LG
0.5
1
1.5
2
2.5
3
3.5
4
4.5
5
5.5
0.5 0.45 0.4 0.35 0.3 0.25 0.2 0.15 0.1 0.05 0
0.06 0.04 0.02 0 -0.02 -0.04
0.5 0.4 0.3 0.2 0.1 0
0.5 0.45 0.4 0.35 0.3 0.25 0.2 0.15 0.1 0.05 0
0.45 0.4 0.35 0.3 0.25 0.2 0.15 0.1 0.05 0
2.5 2 1.5 1 0.5 0 -0.5 -1

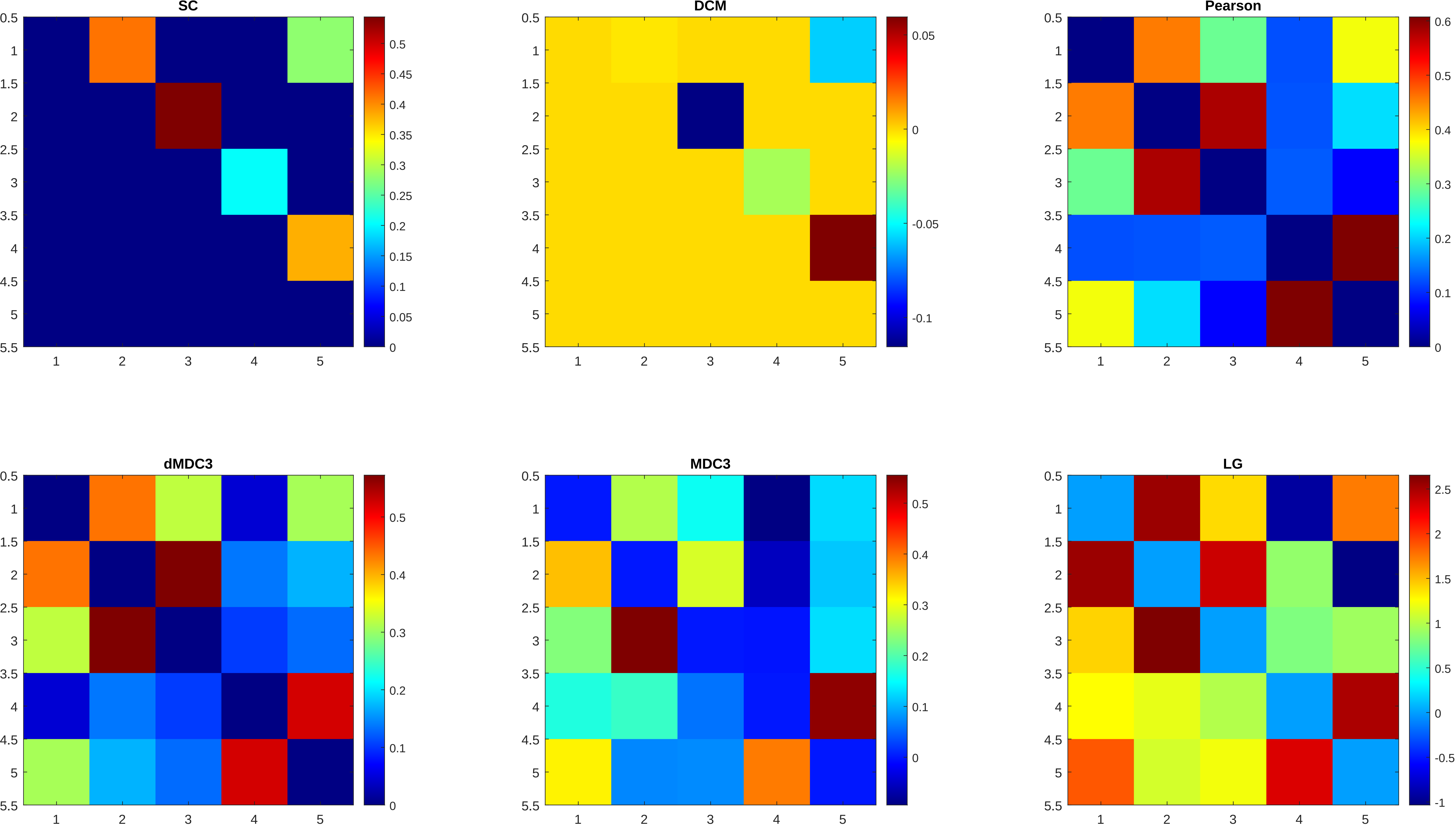
SC
DCM
Pearson
dMDC3
MDC3
LG
0.5
1
1.5
2
2.5
3
3.5
4
4.5
5
5.5

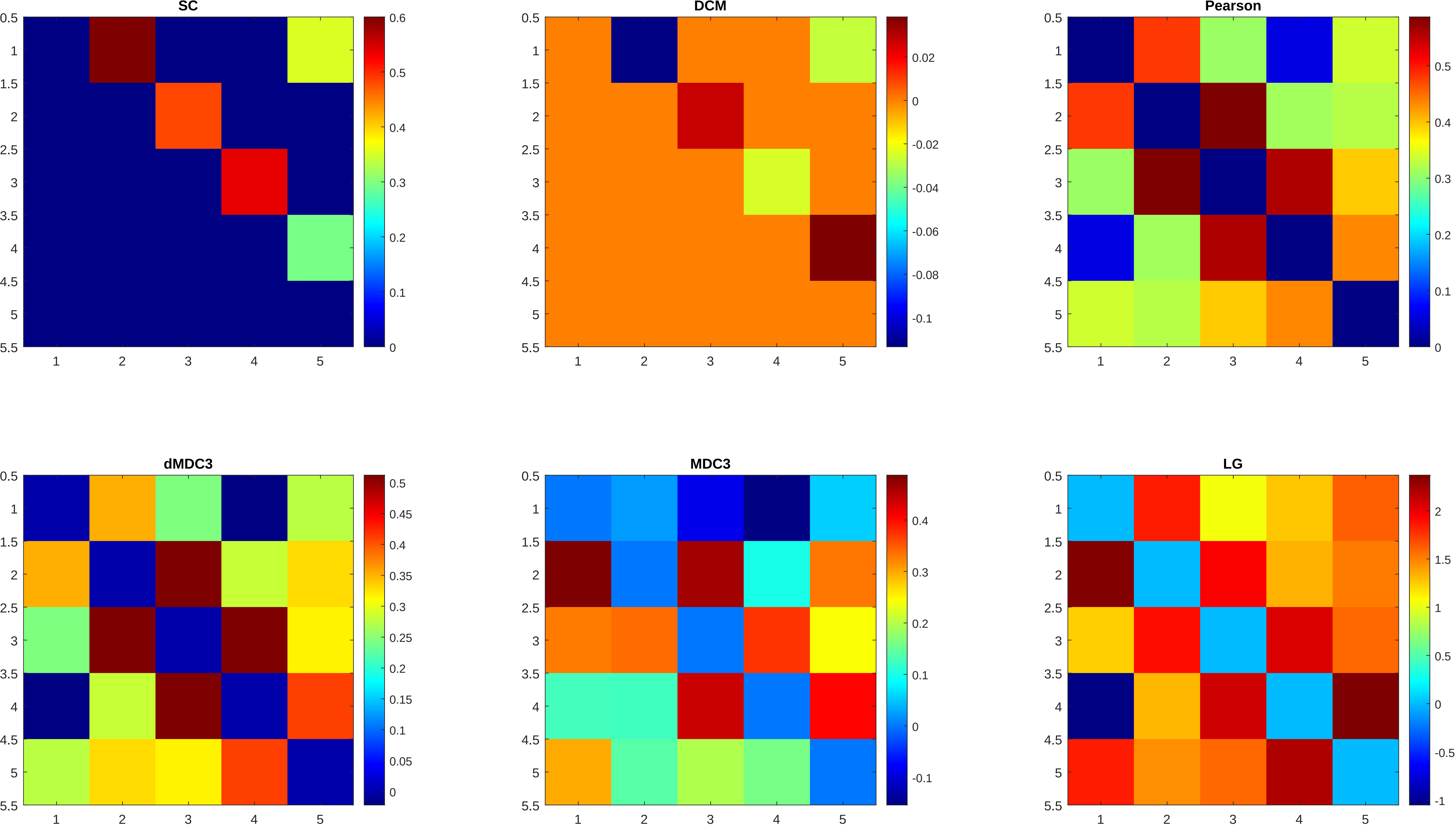

SC
DCM
Pearson
dMDC3
MDC3
LG
