# Supplementary material for: Multiscale detrended cross-correlation coefficient: estimating coupling in non-stationary neurophysiological signals
Source: Front Neurosci. 2024 Nov 13;18:1422085. doi: 10.3389/fnins.2024.1422085 (PMC11599215; doi:10.3389/fnins.2024.1422085)
Supplement: Supplementary file 1 [file Data_Sheet_1.zip › NetSim Matrices.zip!/NetSim Matrices/sim22.pdf]

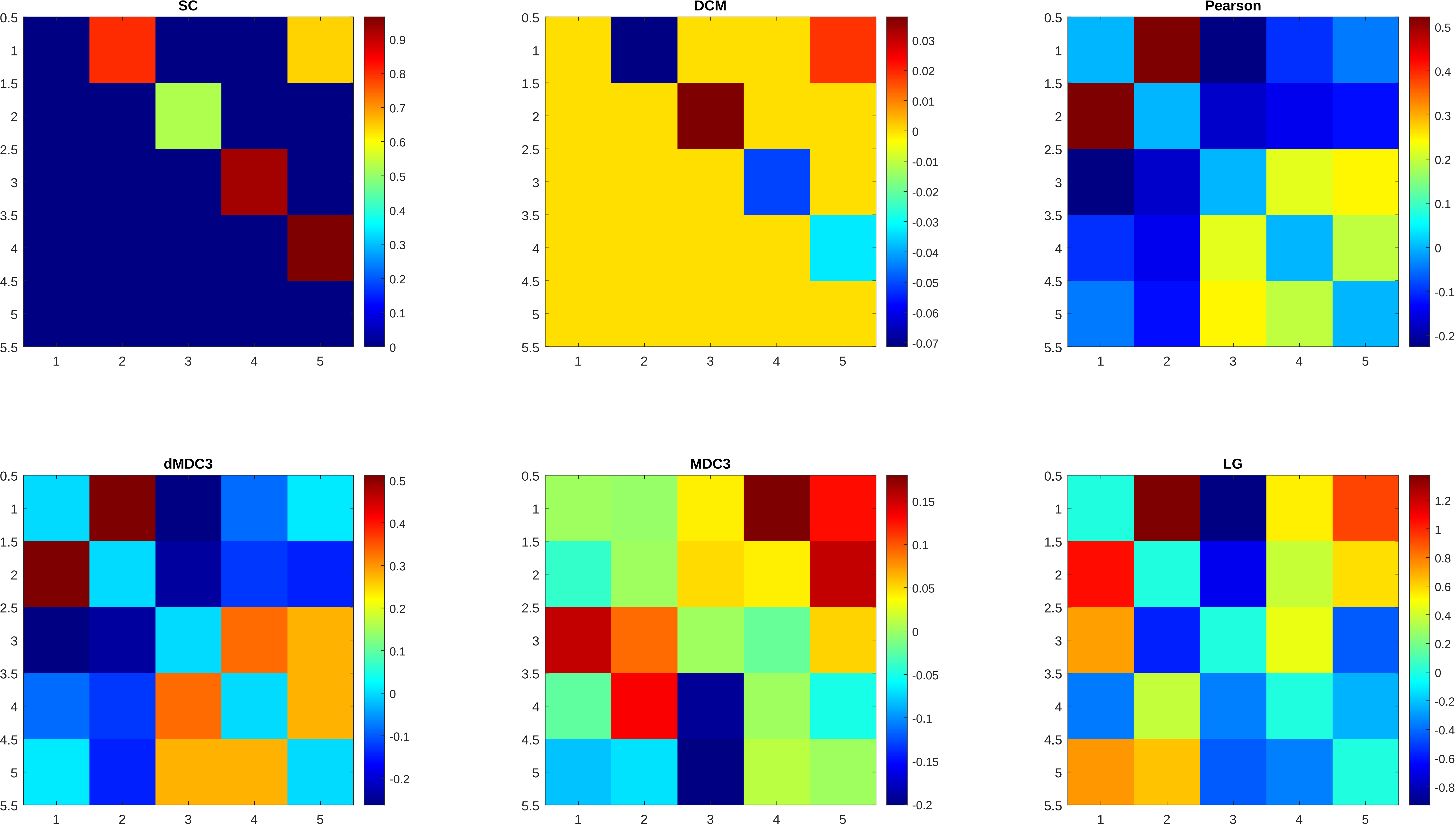
SC
0.5 1 1.5 2 2.5 3 3.5 4 4.5 5 5.5
1 2 3 4 5
0.9 0.8 0.7 0.6 0.5 0.4 0.3 0.2 0.1 0
DCM
0.5 1 1.5 2 2.5 3 3.5 4 4.5 5 5.5
1 2 3 4 5
0.03 0.02 0.01 0 -0.01 -0.02 -0.03 -0.04 -0.05 -0.06 -0.07
Pearson
0.5 1 1.5 2 2.5 3 3.5 4 4.5 5 5.5
1 2 3 4 5
0.5 0.4 0.3 0.2 0.1 0 -0.1 -0.2
dMDC3
0.5 1 1.5 2 2.5 3 3.5 4 4.5 5 5.5
1 2 3 4 5
0.5 0.4 0.3 0.2 0.1 0 -0.1 -0.2
MDC3
0.5 1 1.5 2 2.5 3 3.5 4 4.5 5 5.5
1 2 3 4 5
0.15 0.1 0.05 0 -0.05 -0.1 -0.15 -0.2
LG
0.5 1 1.5 2 2.5 3 3.5 4 4.5 5 5.5
1 2 3 4 5
1.2 1 0.8 0.6 0.4 0.2 0 -0.2 -0.4 -0.6 -0.8

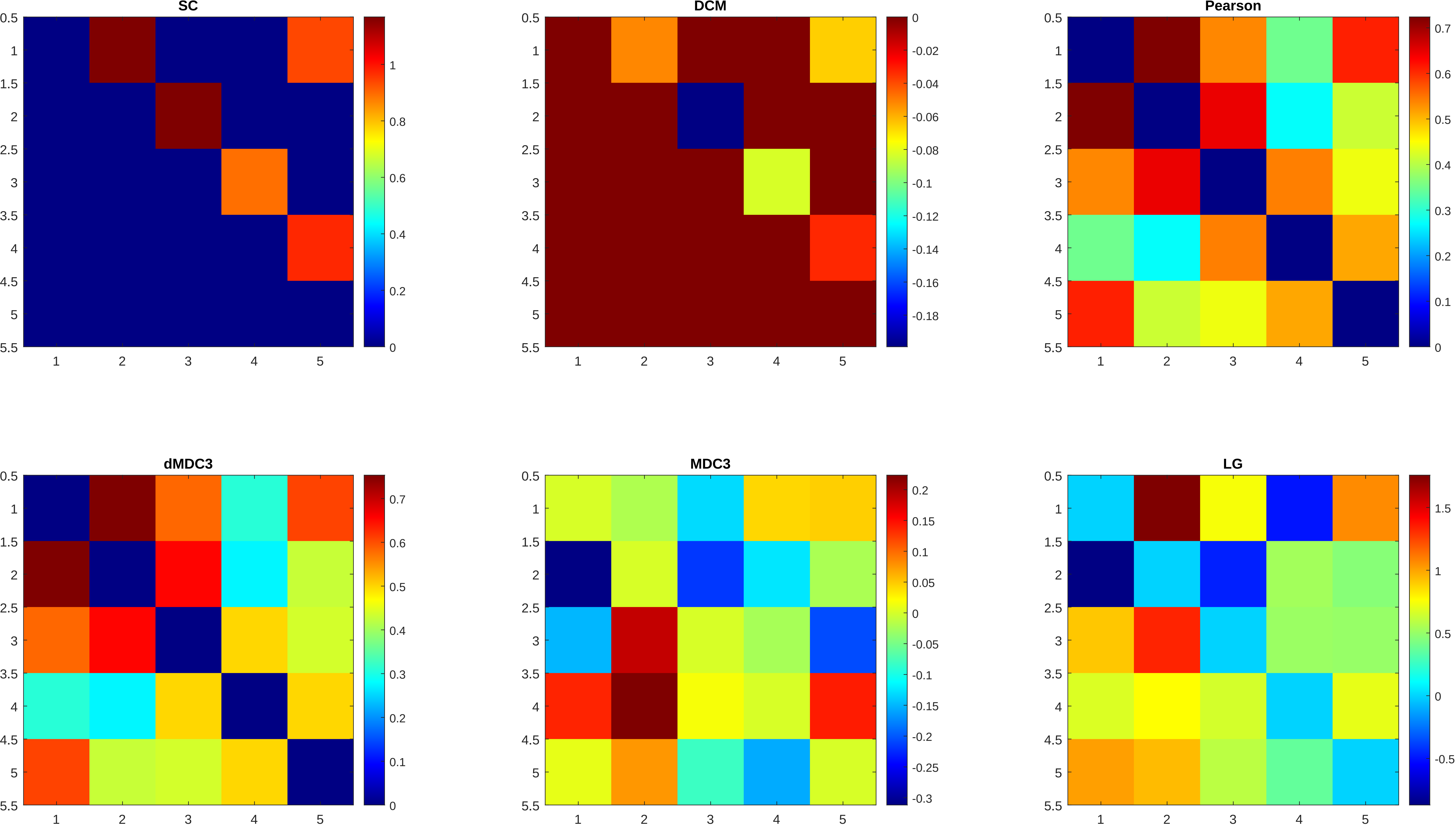
SC
DCM
Pearson
dMDC3
MDC3
LG

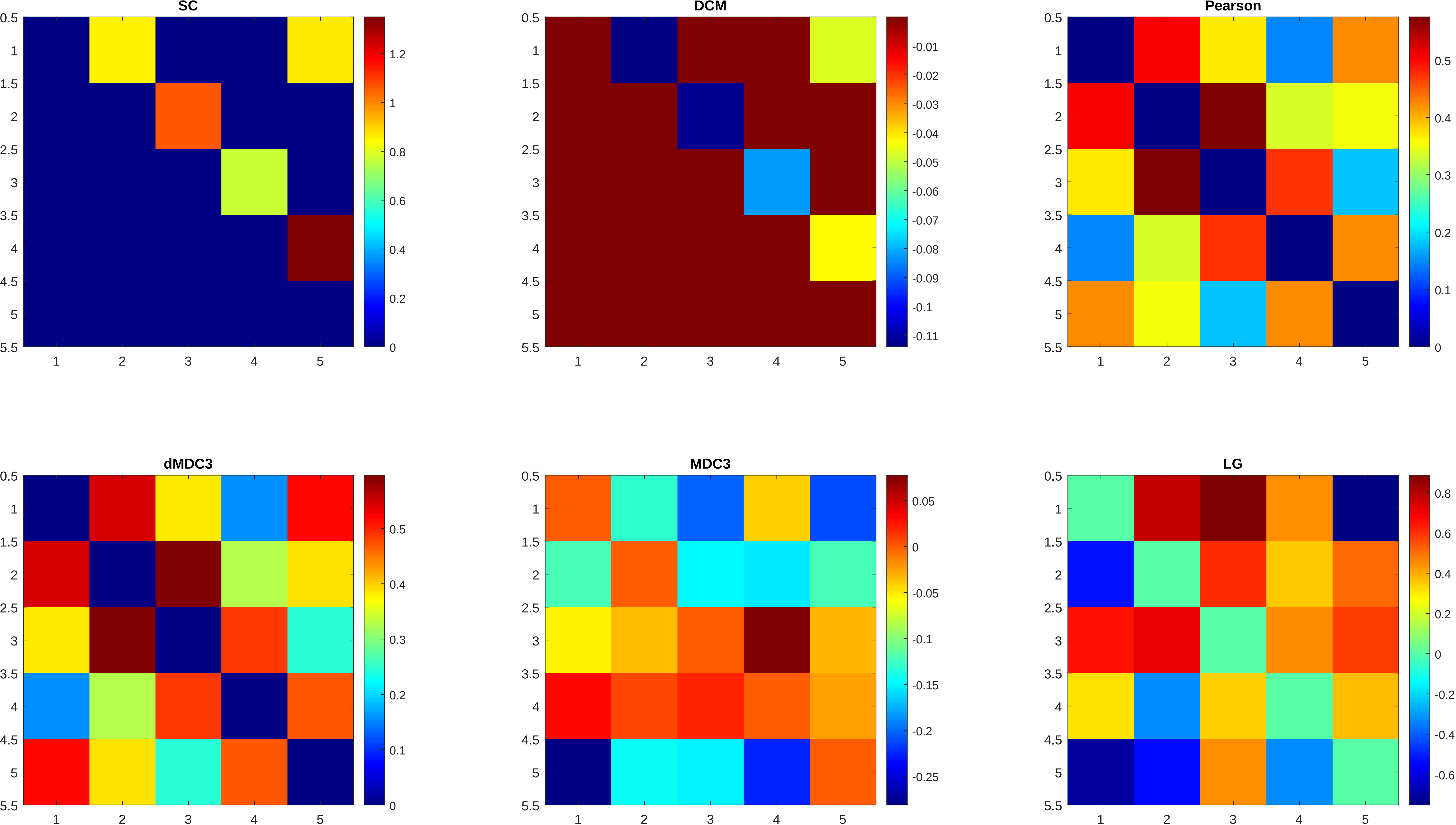

SC
DCM
Pearson
dMDC3
MDC3
LG

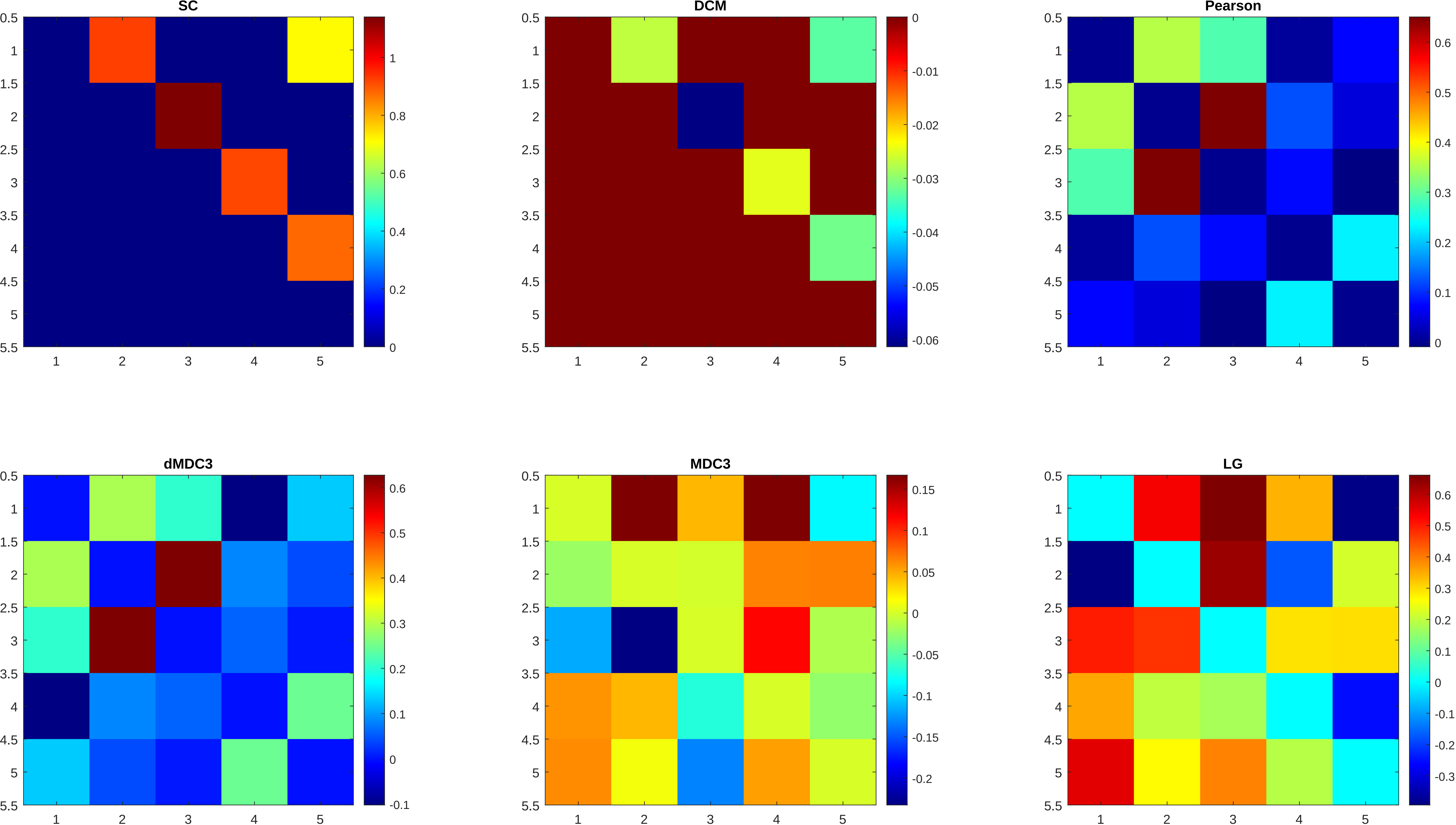

SC
DCM
Pearson
dMDC3
MDC3
LG

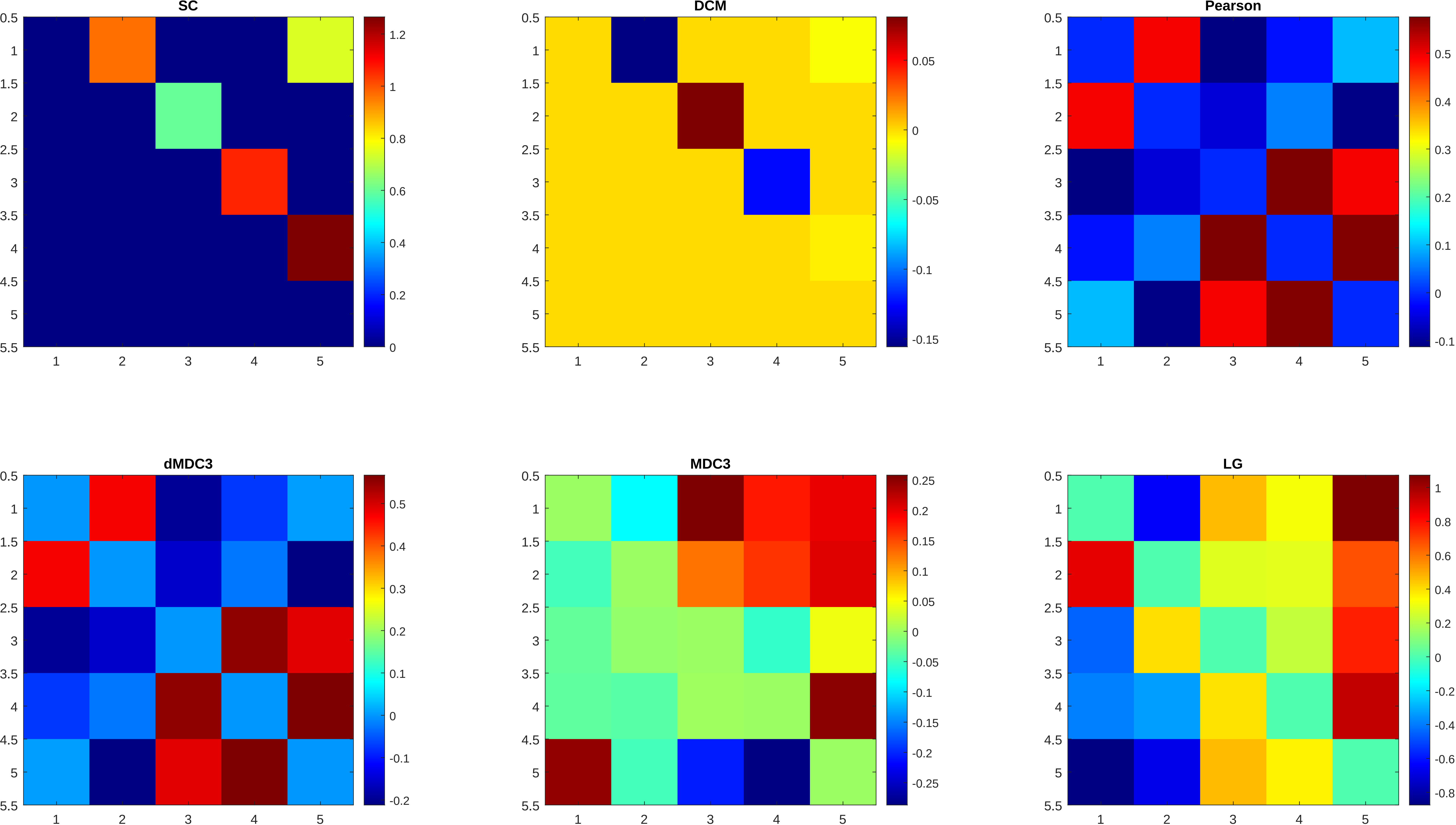
SC
0.5
1
1.5
2
2.5
3
3.5
4
4.5
5
5.5
1 2 3 4 5
0 0.2 0.4 0.6 0.8 1 1.2
DCM
-0.15 -0.1 -0.05 0 0.05
Pearson
-0.1 0 0.1 0.2 0.3 0.4 0.5
dMDC3
-0.2 -0.1 0 0.1 0.2 0.3 0.4 0.5
MDC3
-0.25 -0.2 -0.15 -0.1 -0.05 0 0.05 0.1 0.15 0.2 0.25
LG
-0.8 -0.6 -0.4 -0.2 0 0.2 0.4 0.6 0.8 1

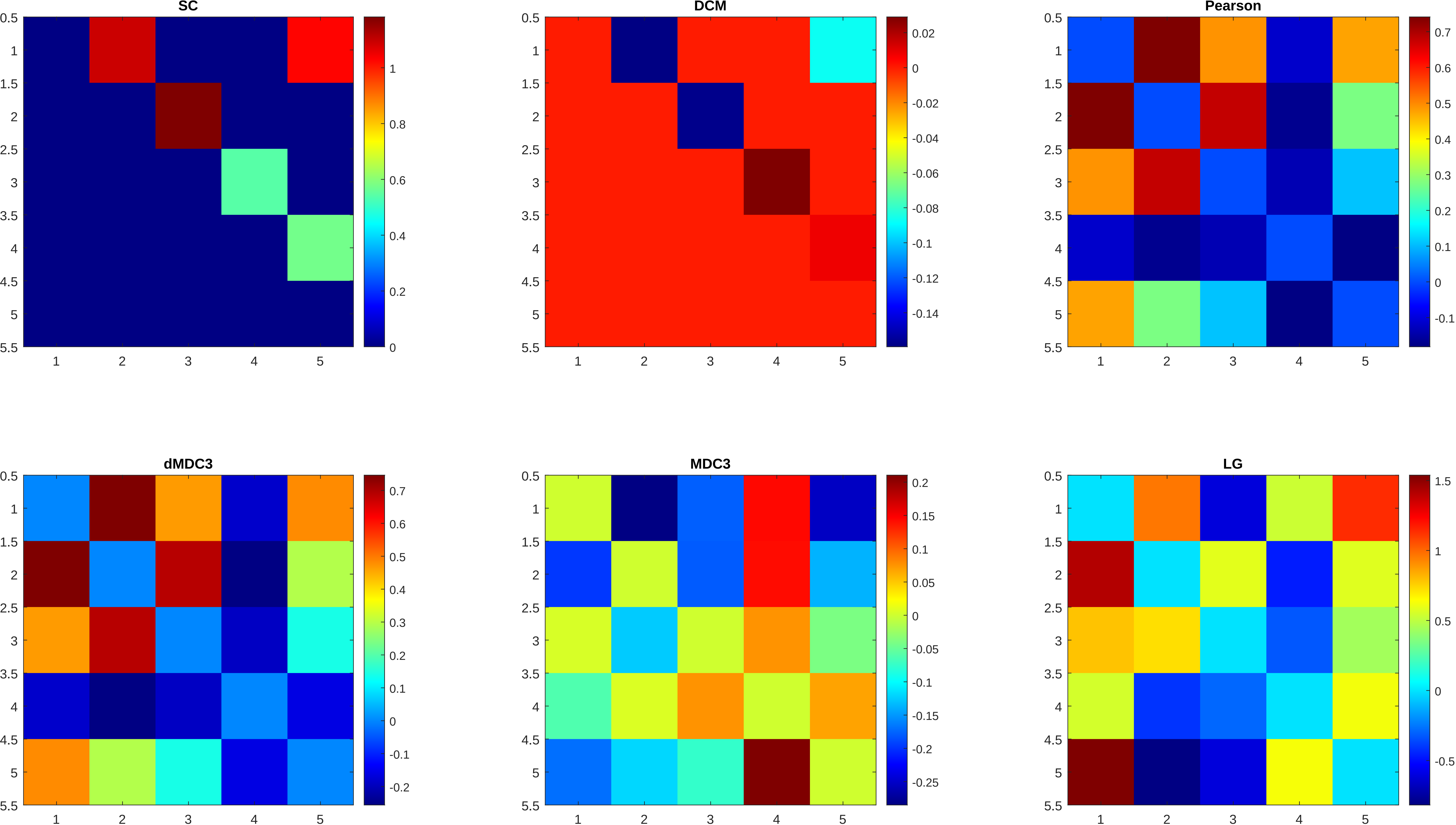

SC
DCM
Pearson
dMDC3
MDC3
LG

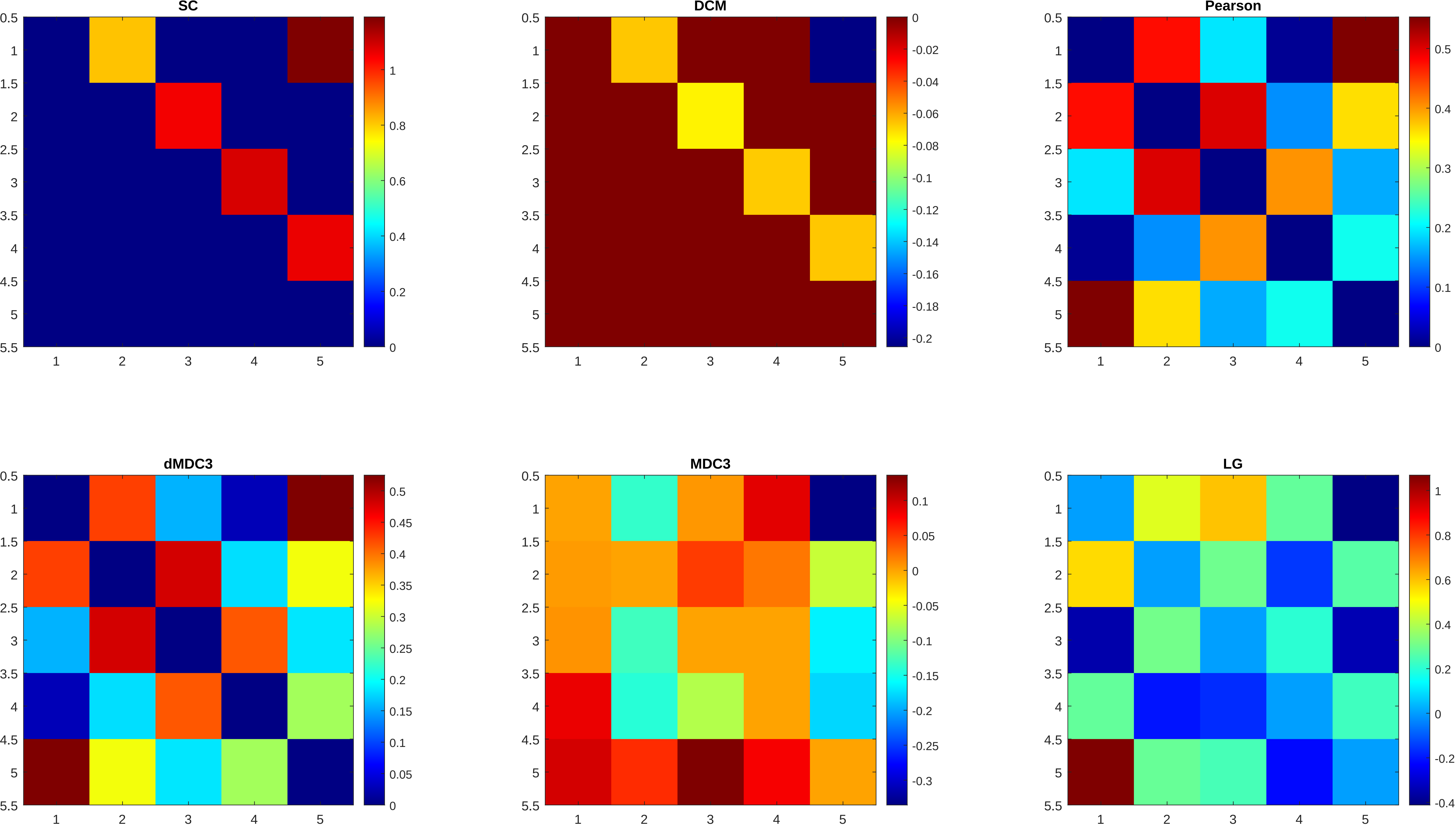
SC
DCM
Pearson
dMDC3
MDC3
LG
0.5 1 1.5 2 2.5 3 3.5 4 4.5 5 5.5
1 2 3 4 5
0 0.2 0.4 0.6 0.8 1
0 -0.02 -0.04 -0.06 -0.08 -0.1 -0.12 -0.14 -0.16 -0.18 -0.2
0 0.1 0.2 0.3 0.4 0.5
0 0.05 0.1 0.15 0.2 0.25 0.3 0.35 0.4 0.45 0.5
0.1 0.05 0 -0.05 -0.1 -0.15 -0.2 -0.25 -0.3
1 0.8 0.6 0.4 0.2 0 -0.2 -0.4

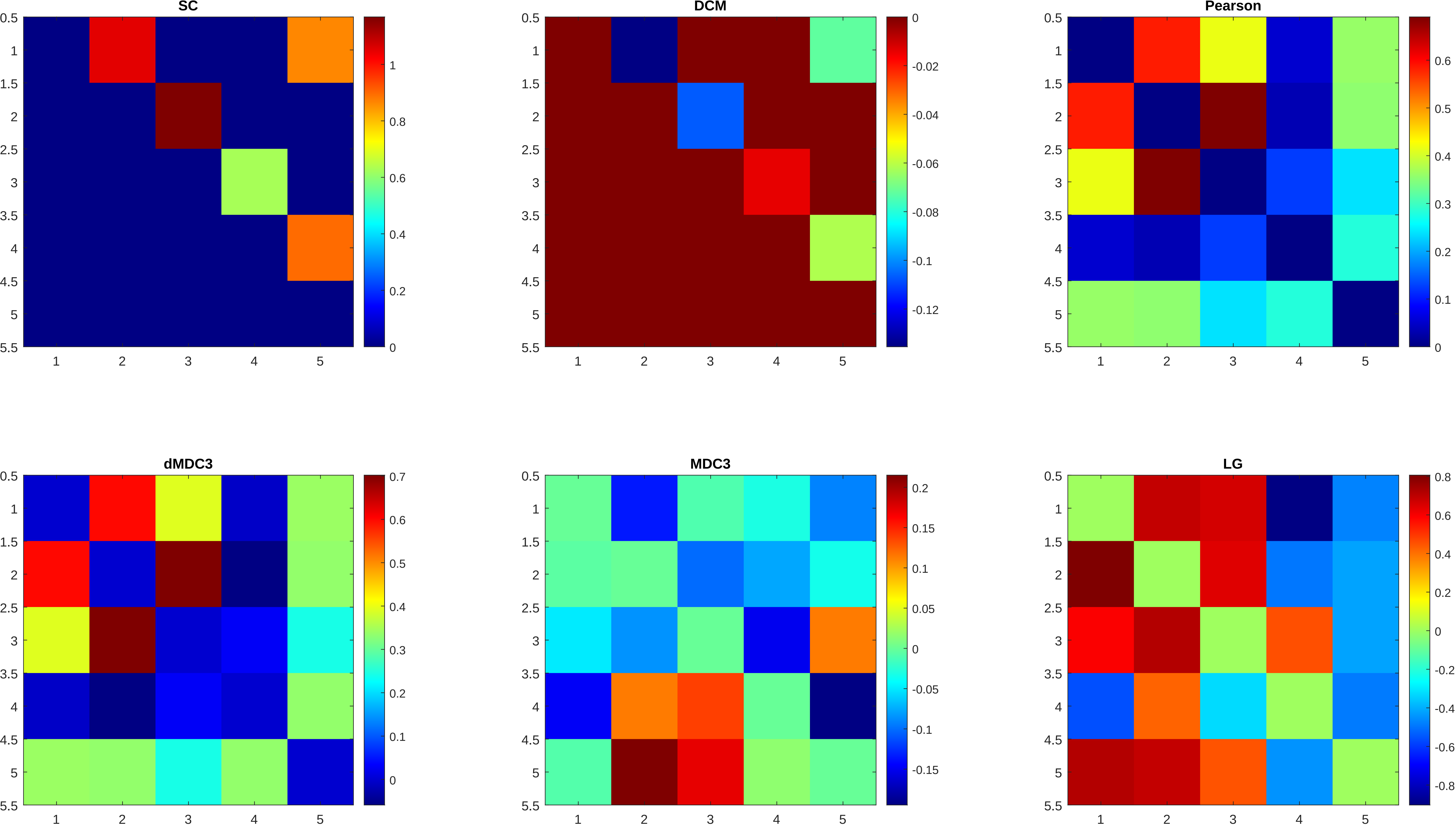
SC
DCM
Pearson
dMDC3
MDC3
LG
0.5
1
1.5
2
2.5
3
3.5
4
4.5
5
5.5
1 2 3 4 5

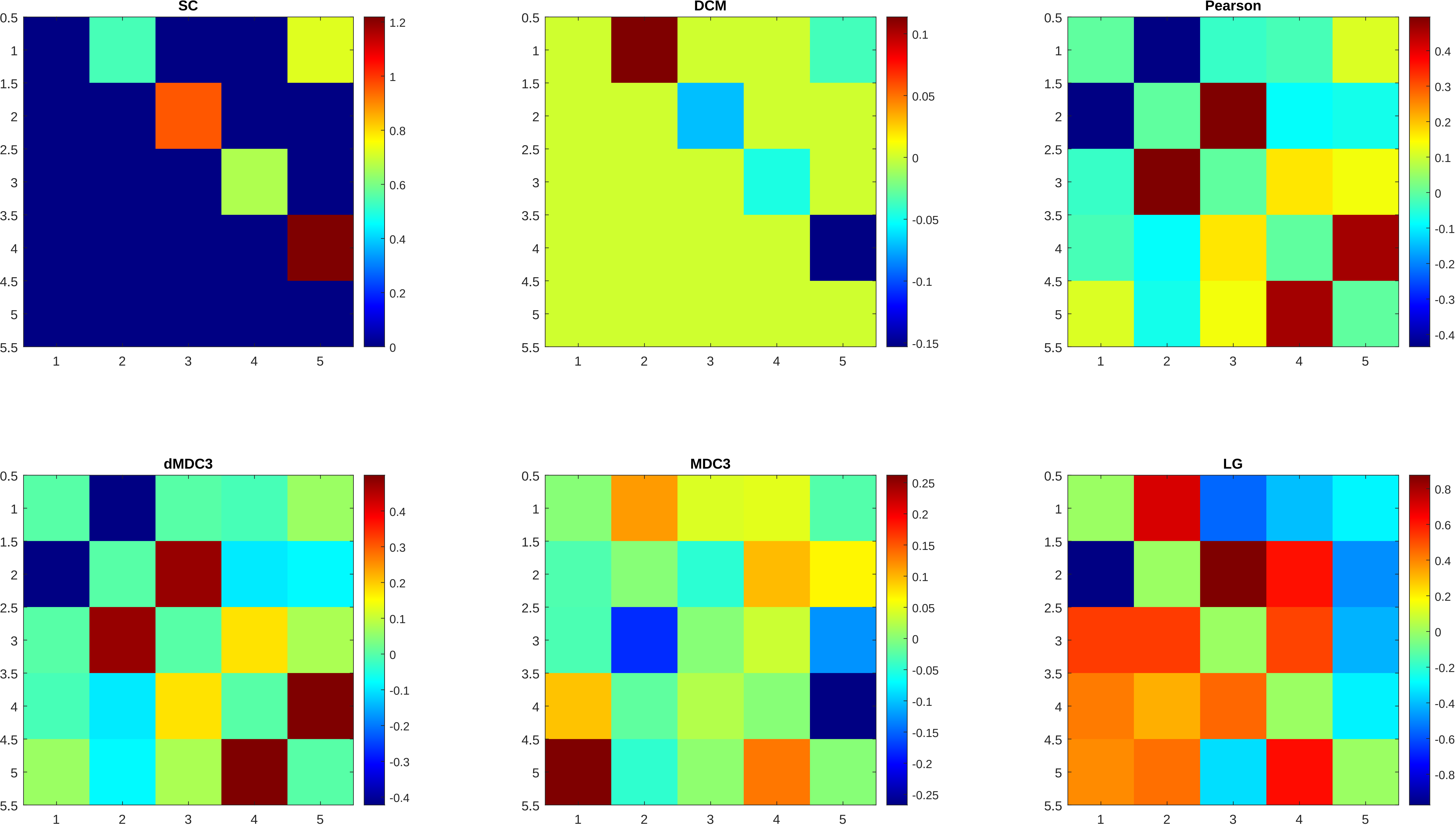
SC
DCM
Pearson
dMDC3
MDC3
LG

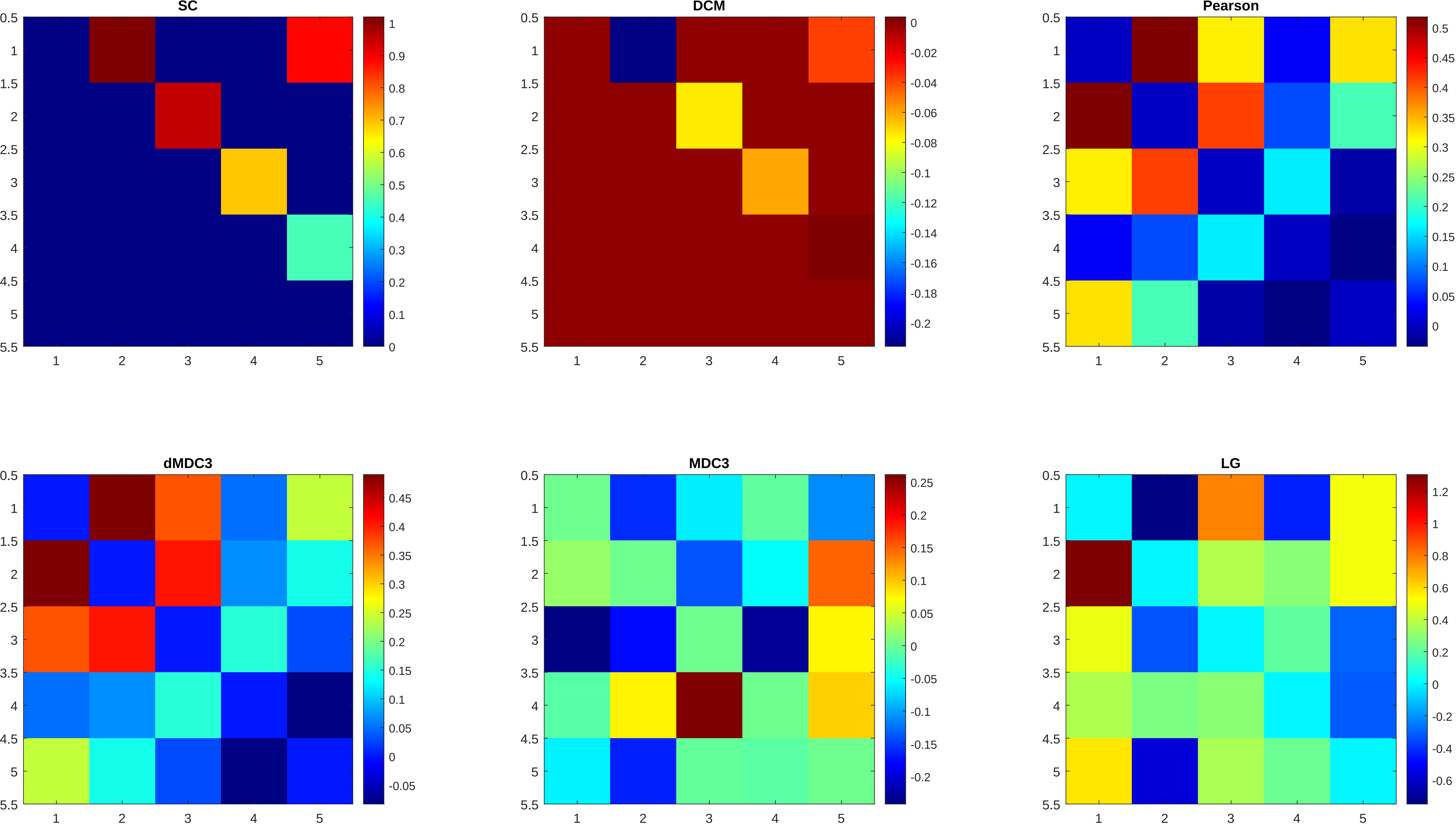

SC
DCM
Pearson
dMDC3
MDC3
LG

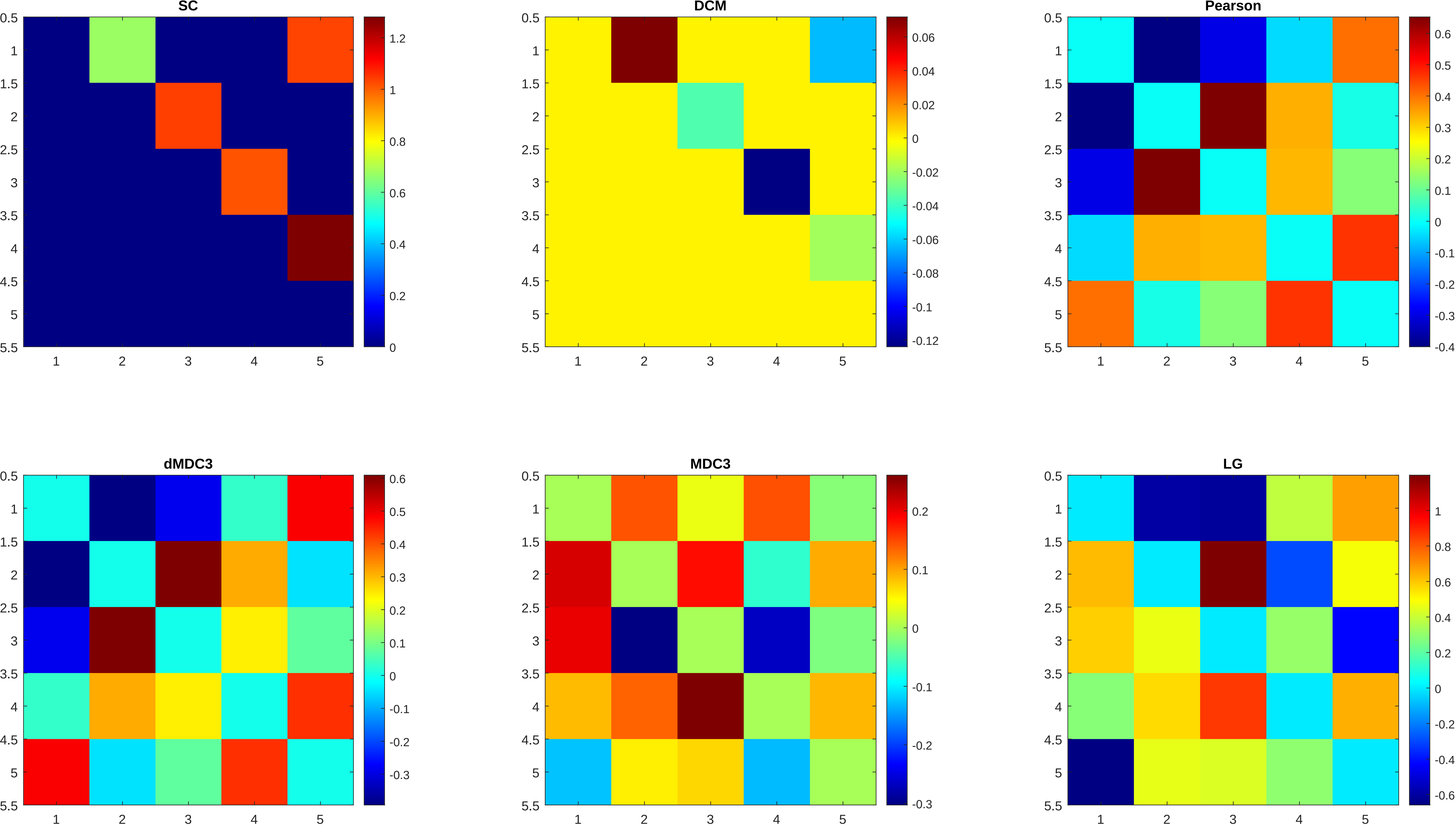
SC
DCM
Pearson
dMDC3
MDC3
LG
0.5 1 1.5 2 2.5 3 3.5 4 4.5 5 5.5
1 2 3 4 5
0 0.2 0.4 0.6 0.8 1 1.2
0.06 0.04 0.02 0 -0.02 -0.04 -0.06 -0.08 -0.1 -0.12
0.6 0.5 0.4 0.3 0.2 0.1 0 -0.1 -0.2 -0.3 -0.4
0.6 0.5 0.4 0.3 0.2 0.1 0 -0.1 -0.2 -0.3
0.2 0.1 0 -0.1 -0.2 -0.3
1 0.8 0.6 0.4 0.2 0 -0.2 -0.4 -0.6

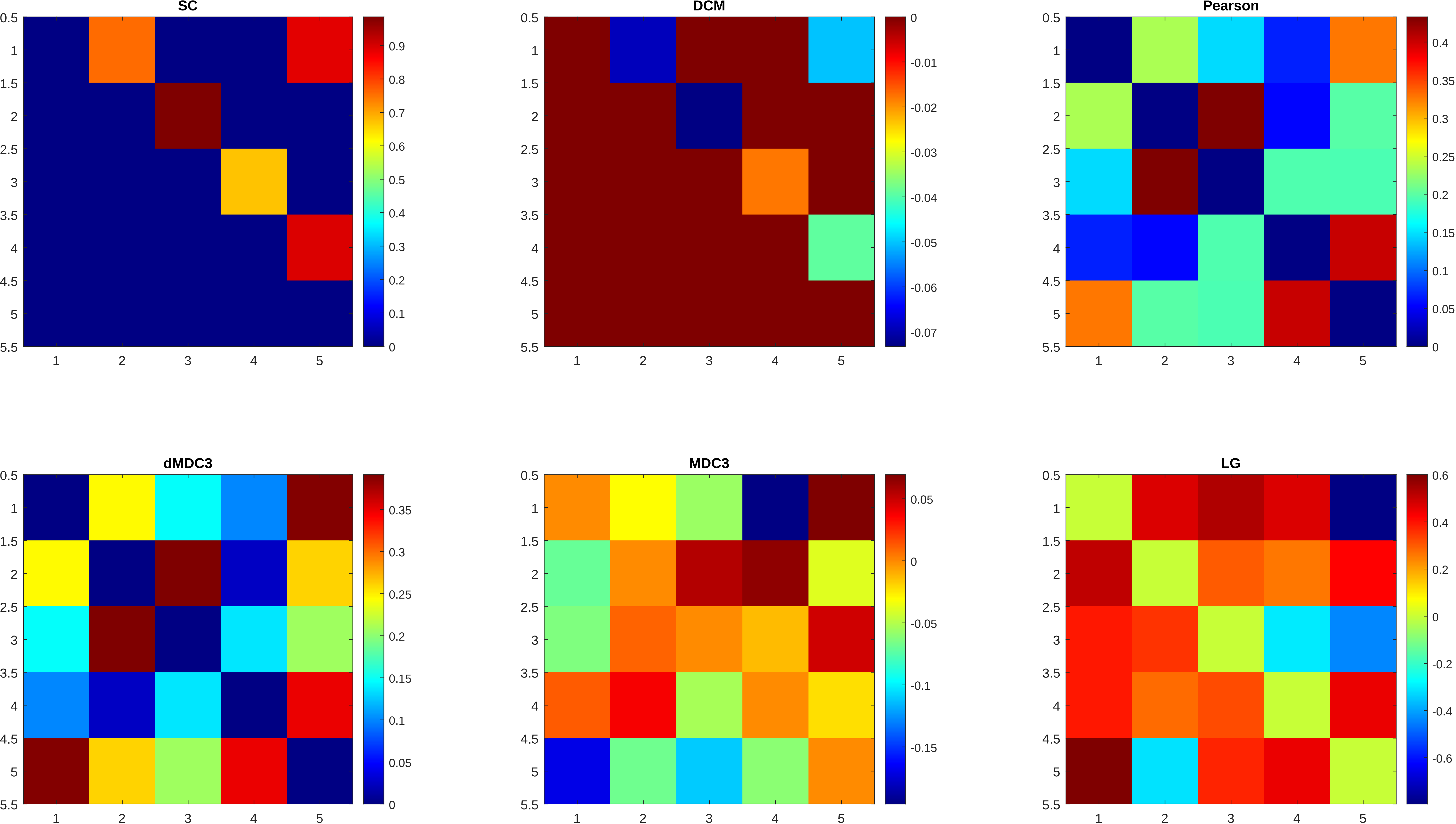
SC
DCM
Pearson
dMDC3
MDC3
LG

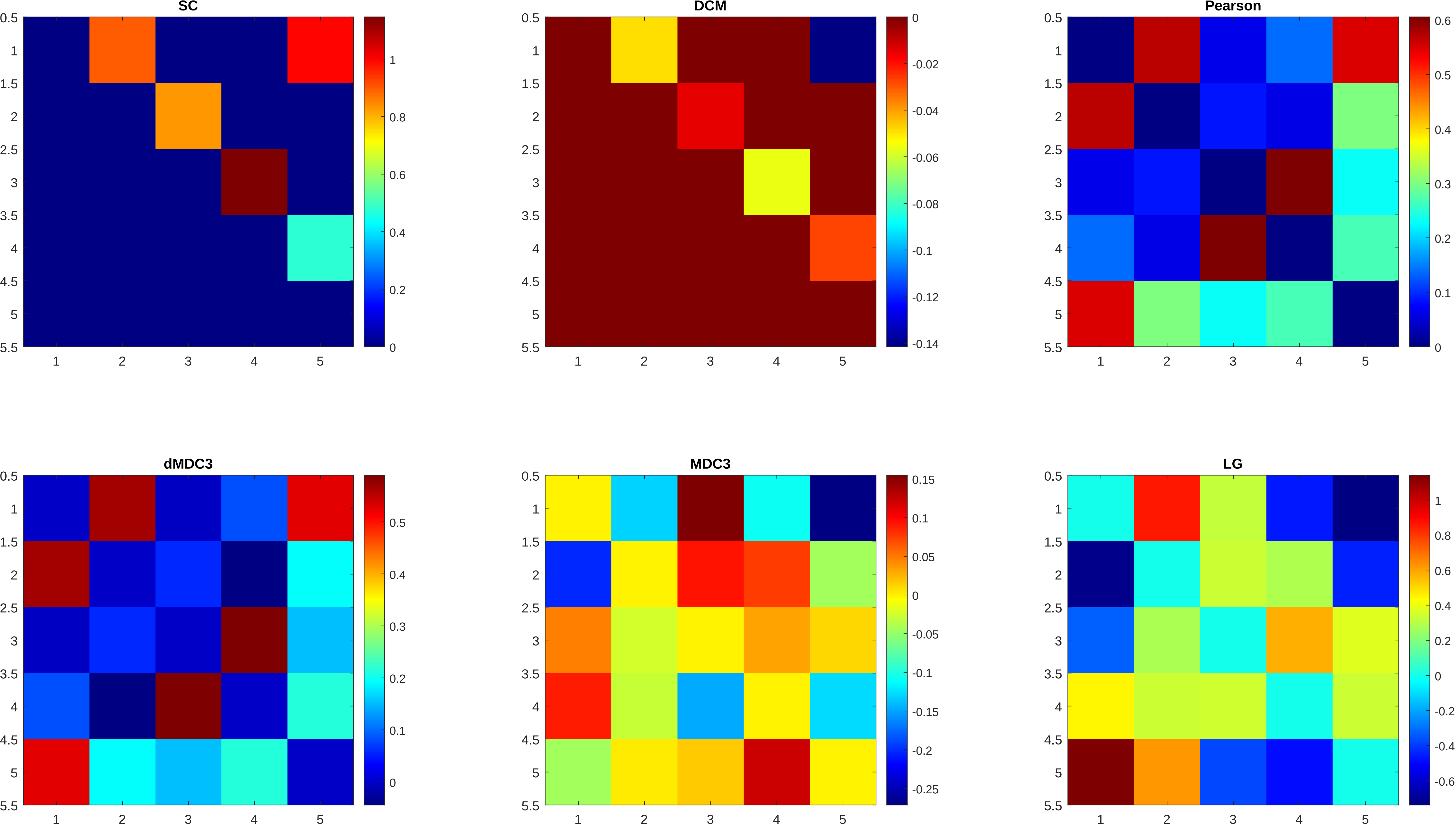
SC
DCM
Pearson
dMDC3
MDC3
LG

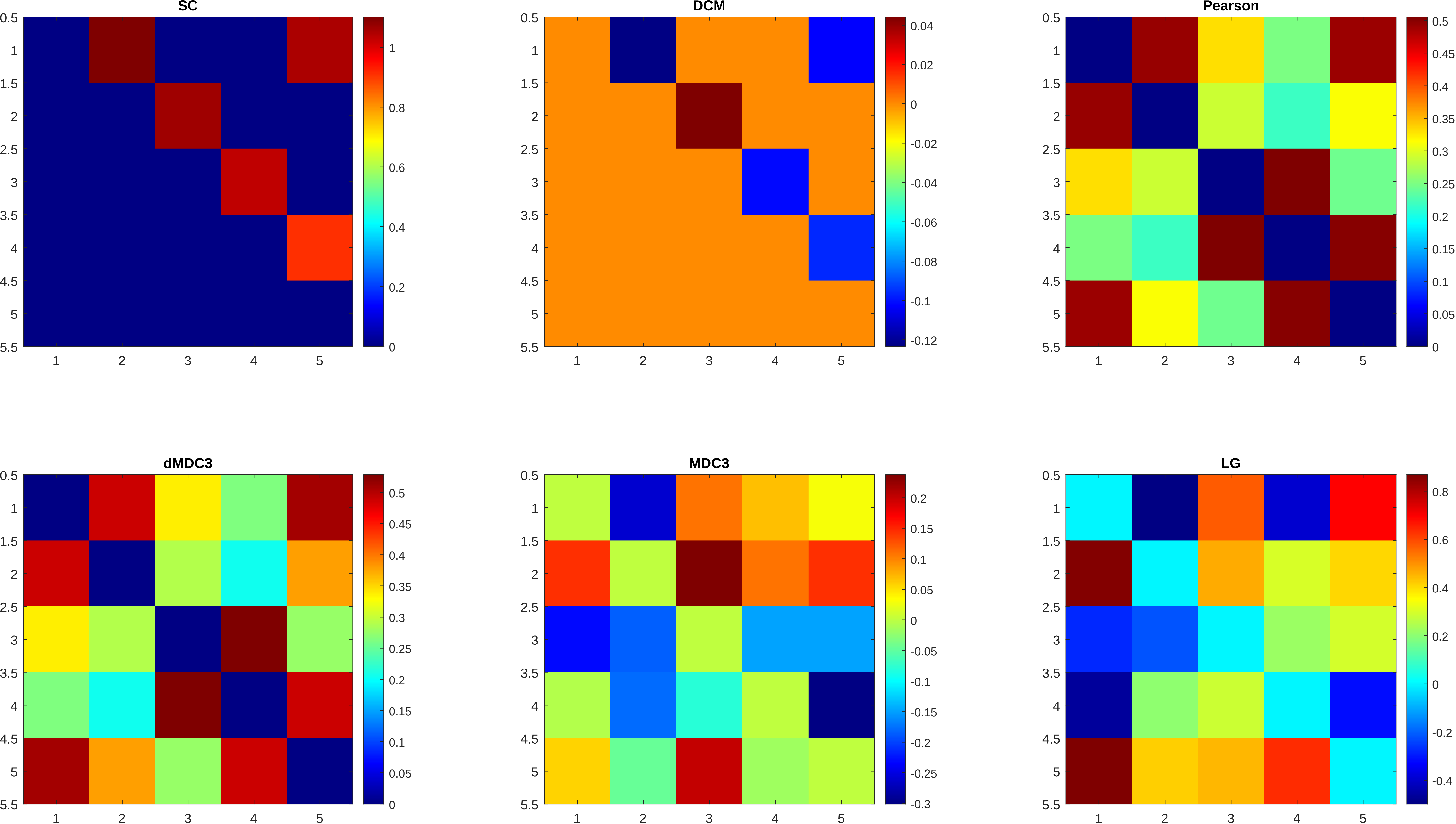
SC
DCM
Pearson
dMDC3
MDC3
LG

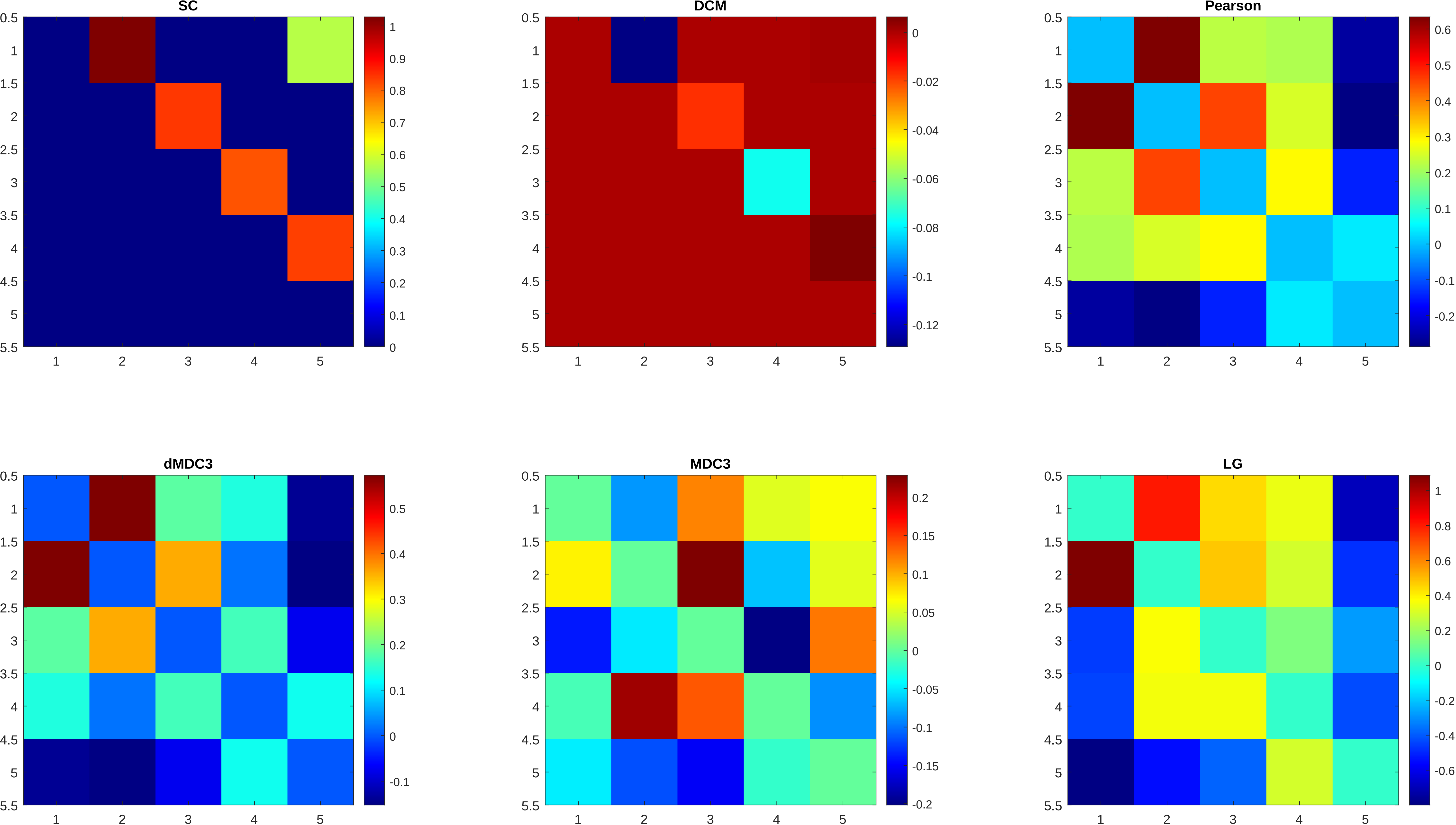
SC
0.5 1 1.5 2 2.5 3 3.5 4 4.5 5 5.5
1 2 3 4 5
0 0.1 0.2 0.3 0.4 0.5 0.6 0.7 0.8 0.9 1
DCM
0.5 1 1.5 2 2.5 3 3.5 4 4.5 5 5.5
1 2 3 4 5
-0.12 -0.1 -0.08 -0.06 -0.04 -0.02 0
Pearson
0.5 1 1.5 2 2.5 3 3.5 4 4.5 5 5.5
1 2 3 4 5
-0.2 -0.1 0 0.1 0.2 0.3 0.4 0.5 0.6
dMDC3
0.5 1 1.5 2 2.5 3 3.5 4 4.5 5 5.5
1 2 3 4 5
-0.1 0 0.1 0.2 0.3 0.4 0.5
MDC3
0.5 1 1.5 2 2.5 3 3.5 4 4.5 5 5.5
1 2 3 4 5
-0.2 -0.15 -0.1 -0.05 0 0.05 0.1 0.15 0.2
LG
0.5 1 1.5 2 2.5 3 3.5 4 4.5 5 5.5
1 2 3 4 5
-0.6 -0.4 -0.2 0 0.2 0.4 0.6 0.8 1

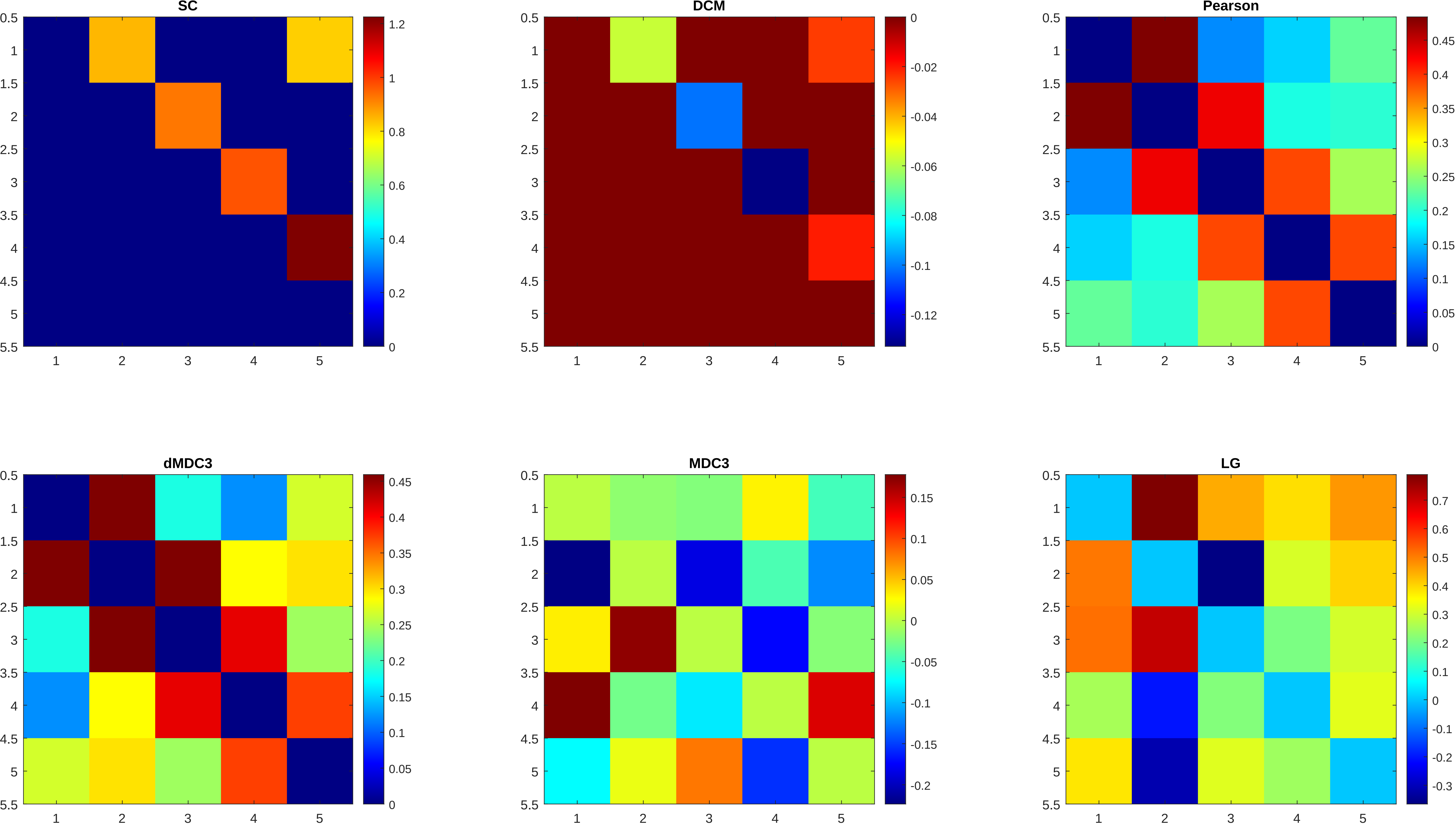

SC
DCM
Pearson
dMDC3
MDC3
LG

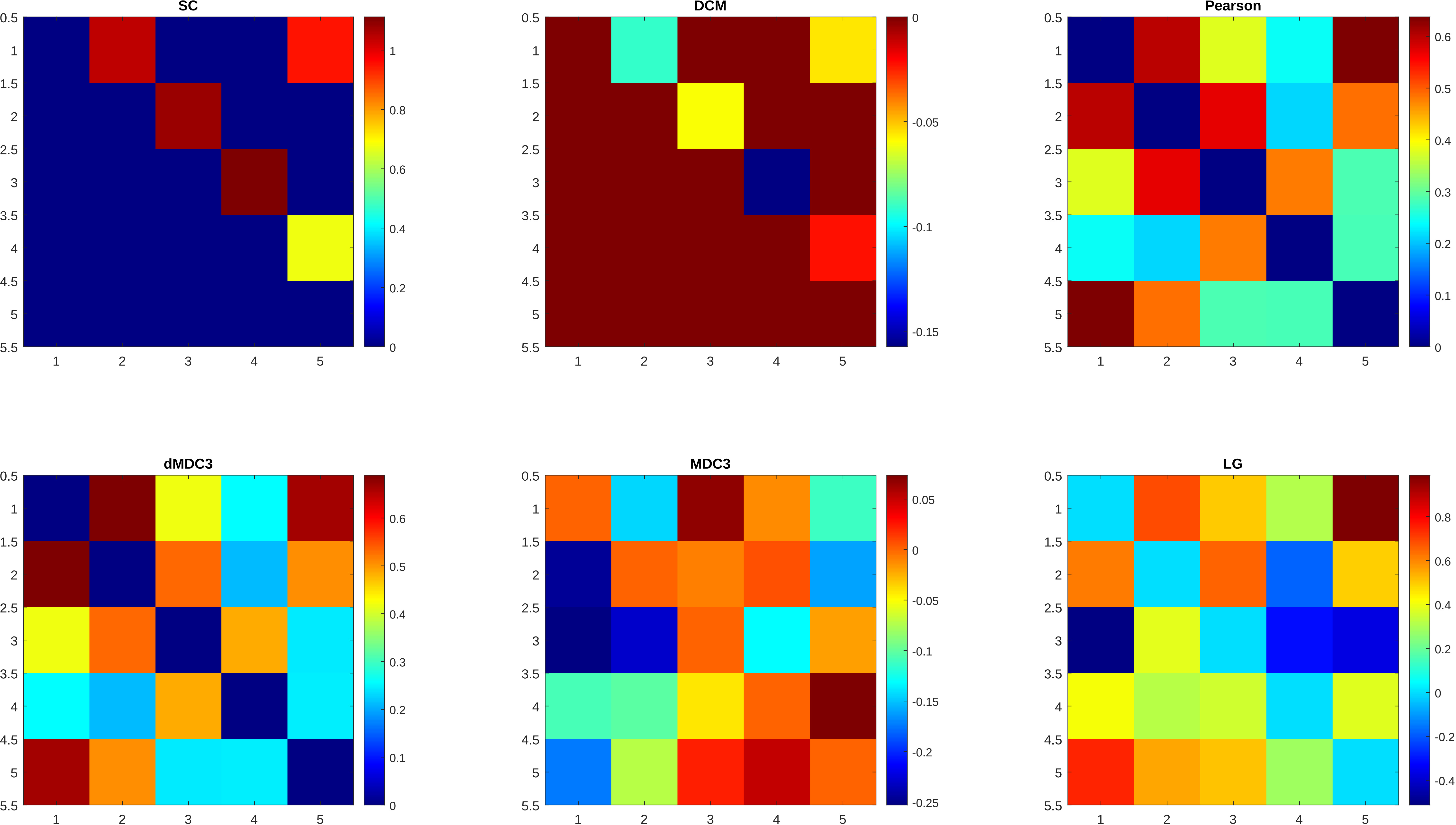

SC
0.5 1 1.5 2 2.5 3 3.5 4 4.5 5 5.5
1 2 3 4 5
0 0.2 0.4 0.6 0.8 1
DCM
0.5 1 1.5 2 2.5 3 3.5 4 4.5 5 5.5
1 2 3 4 5
0 -0.05 -0.1 -0.15
Pearson
0.5 1 1.5 2 2.5 3 3.5 4 4.5 5 5.5
1 2 3 4 5
0 0.1 0.2 0.3 0.4 0.5 0.6
dMDC3
0.5 1 1.5 2 2.5 3 3.5 4 4.5 5 5.5
1 2 3 4 5
0 0.1 0.2 0.3 0.4 0.5 0.6
MDC3
0.5 1 1.5 2 2.5 3 3.5 4 4.5 5 5.5
1 2 3 4 5
0.05 0 -0.05 -0.1 -0.15 -0.2 -0.25
LG
0.5 1 1.5 2 2.5 3 3.5 4 4.5 5 5.5
1 2 3 4 5
0.8 0.6 0.4 0.2 0 -0.2 -0.4

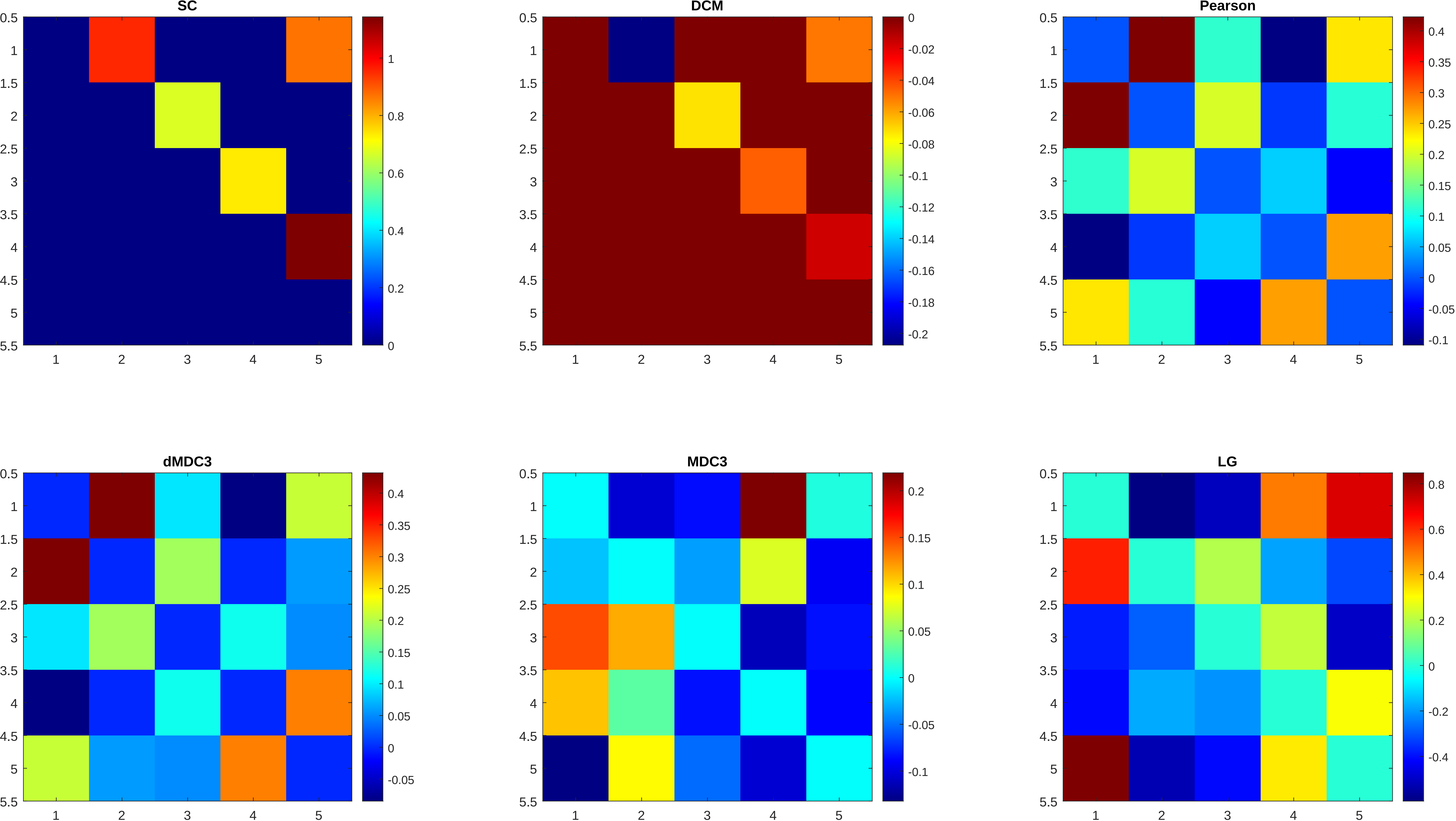

SC
DCM
Pearson
dMDC3
MDC3
LG

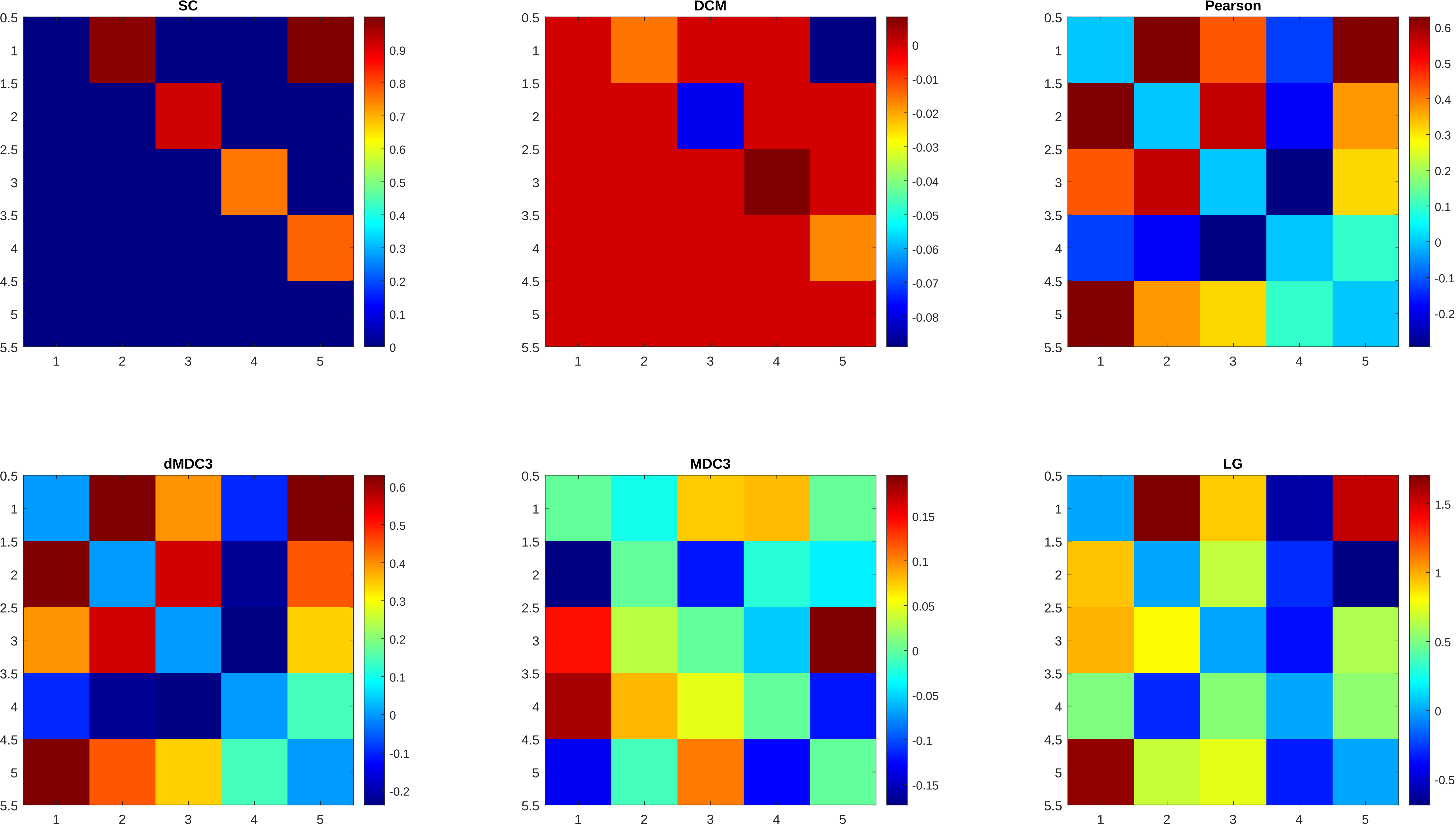
SC
DCM
Pearson
dMDC3
MDC3
LG

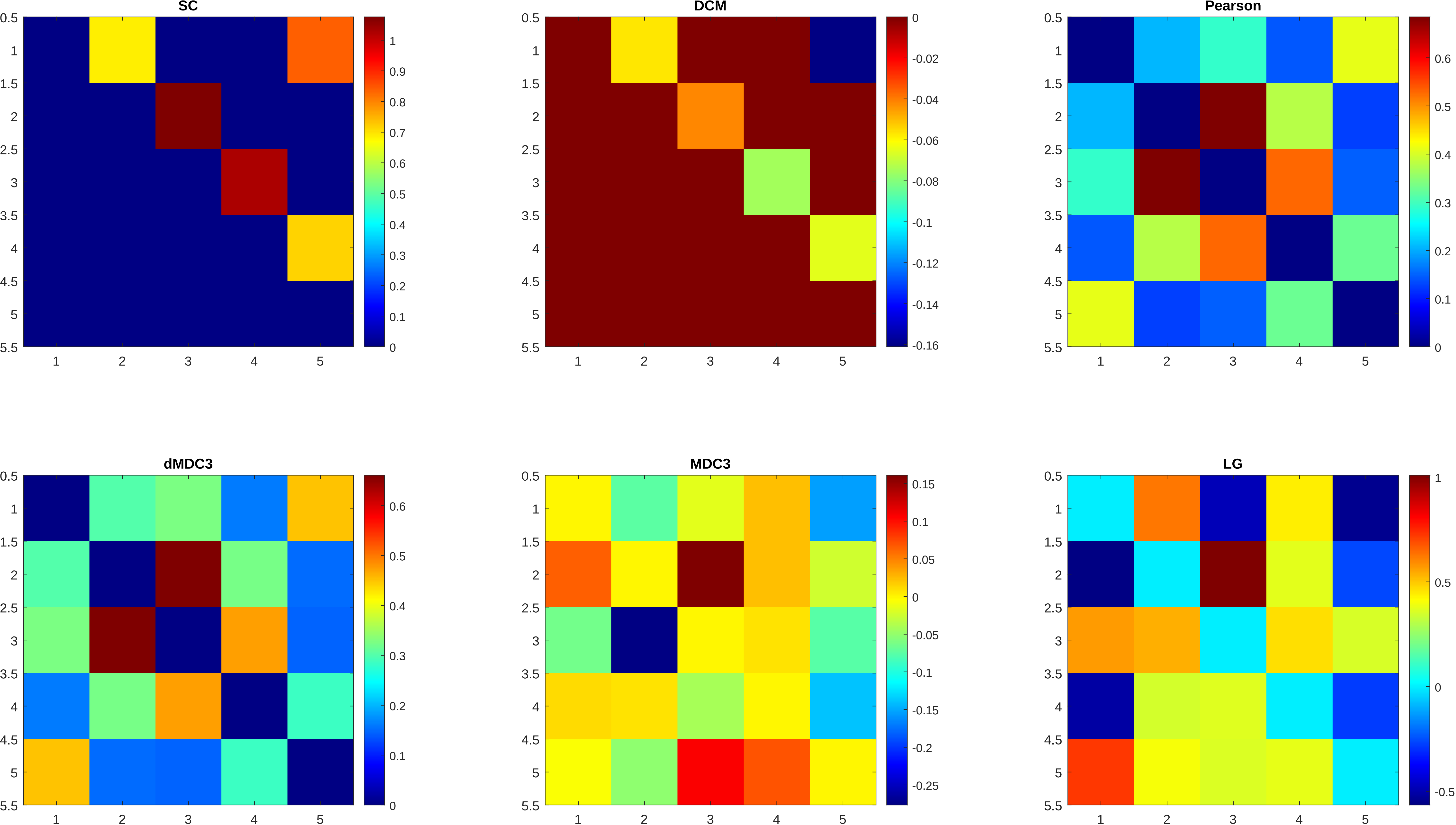

SC
DCM
Pearson
dMDC3
MDC3
LG

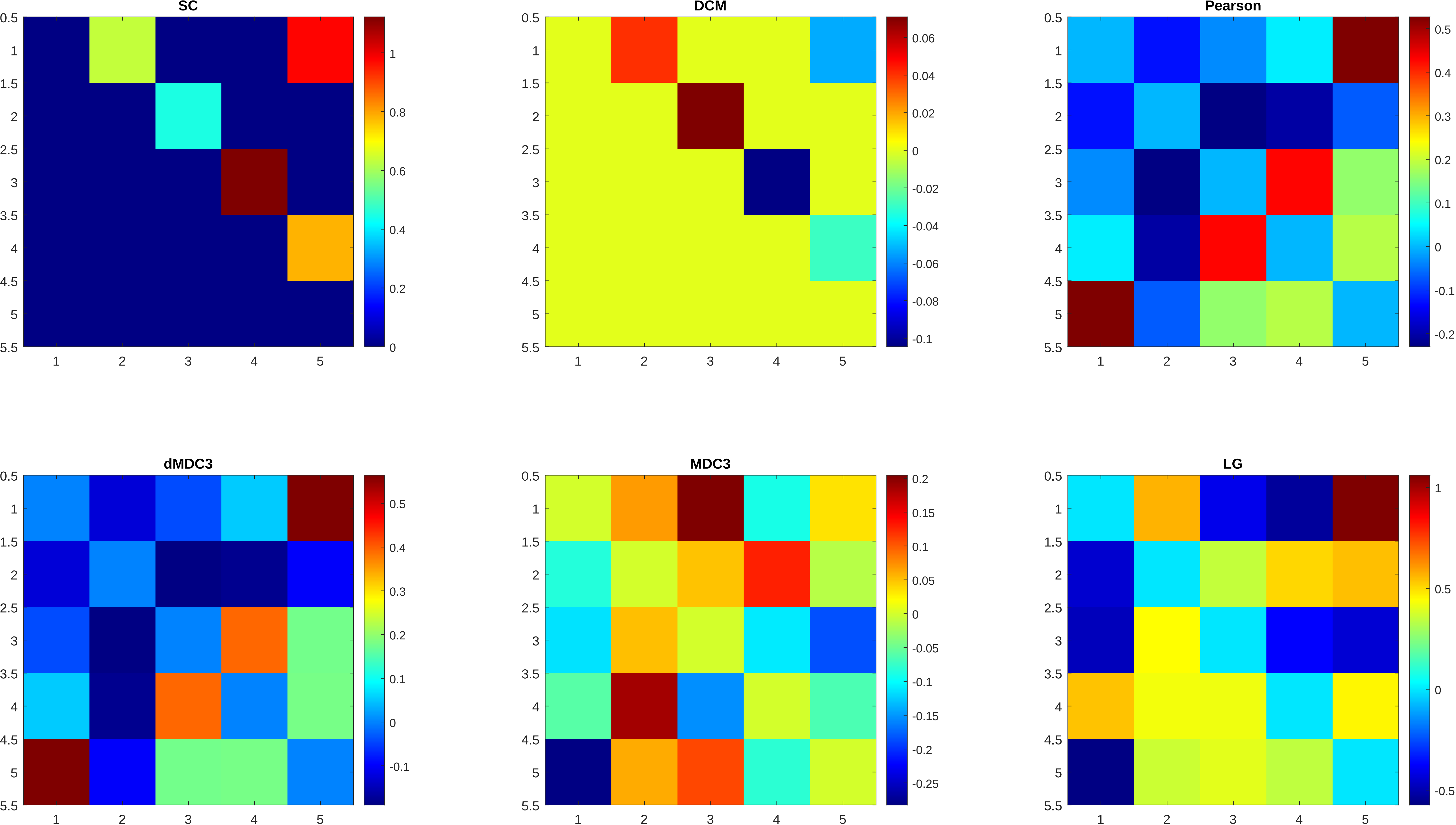

SC
DCM
Pearson
dMDC3
MDC3
LG

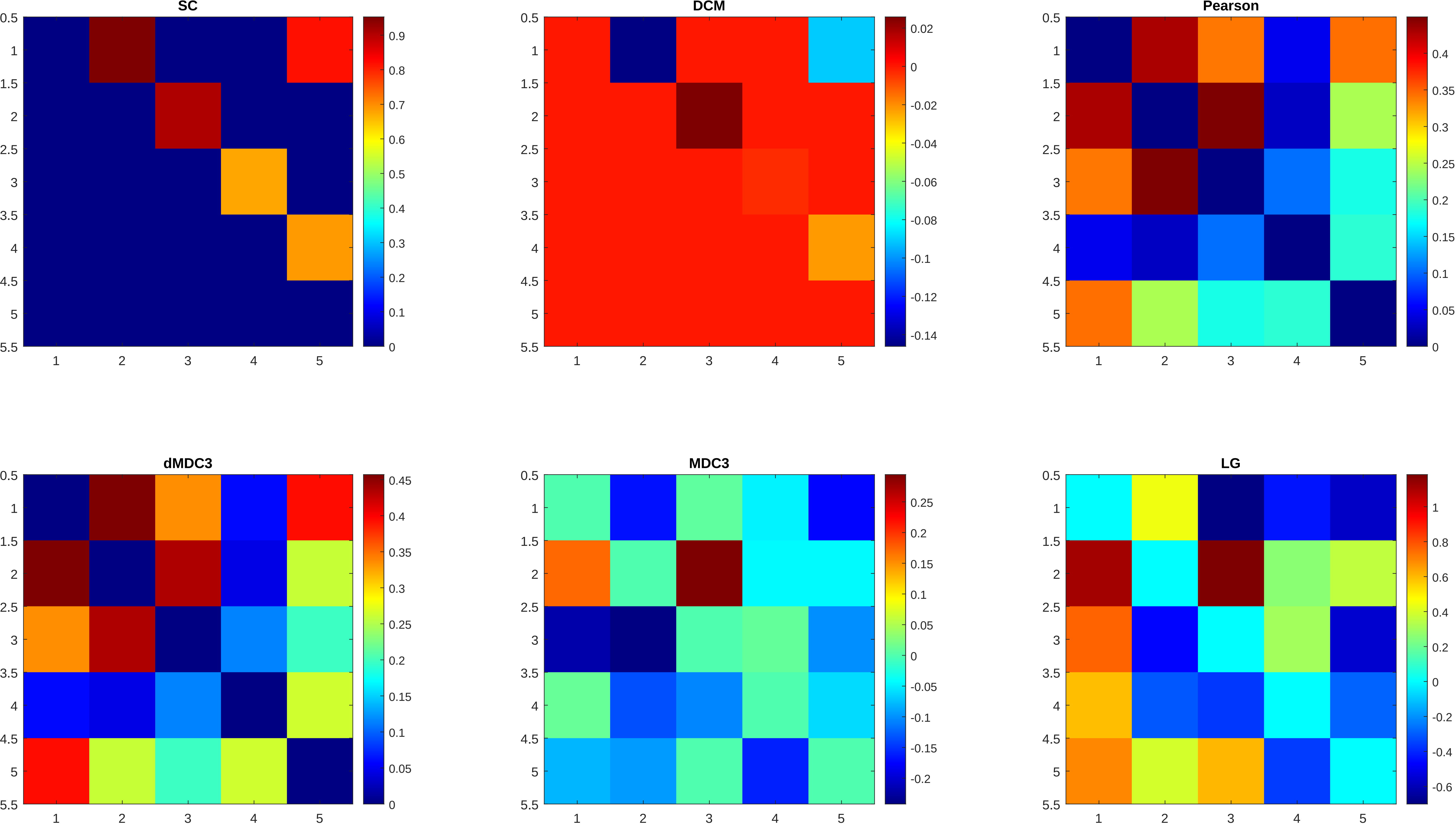

SC
0 0.1 0.2 0.3 0.4 0.5 0.6 0.7 0.8 0.9
DCM
-0.14 -0.12 -0.1 -0.08 -0.06 -0.04 -0.02 0 0.02
Pearson
0 0.05 0.1 0.15 0.2 0.25 0.3 0.35 0.4
dMDC3
0 0.05 0.1 0.15 0.2 0.25 0.3 0.35 0.4 0.45
MDC3
-0.2 -0.15 -0.1 -0.05 0 0.05 0.1 0.15 0.2 0.25
LG
-0.6 -0.4 -0.2 0 0.2 0.4 0.6 0.8 1

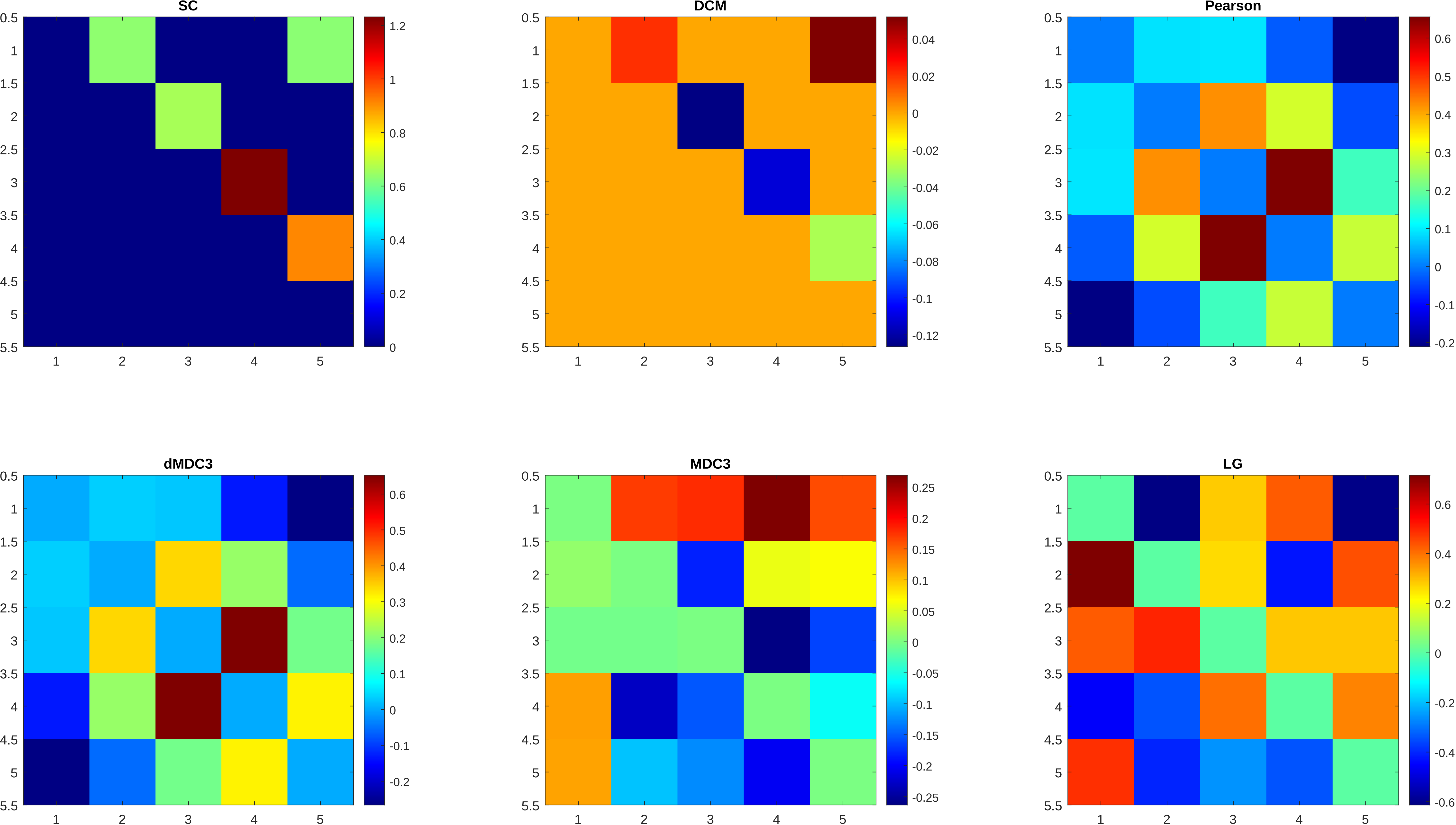
SC
DCM
Pearson
dMDC3
MDC3
LG

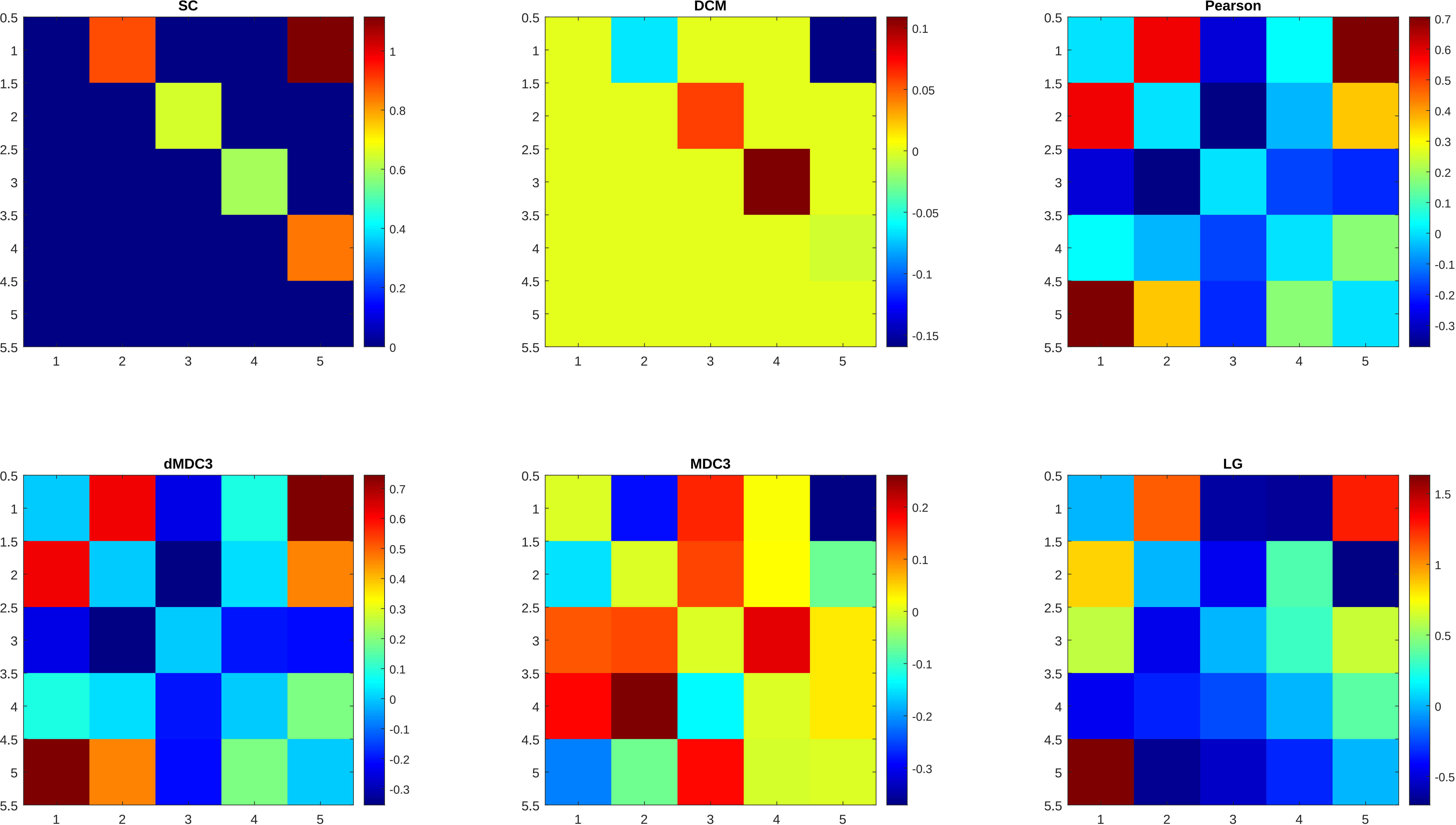

SC
DCM
Pearson
dMDC3
MDC3
LG

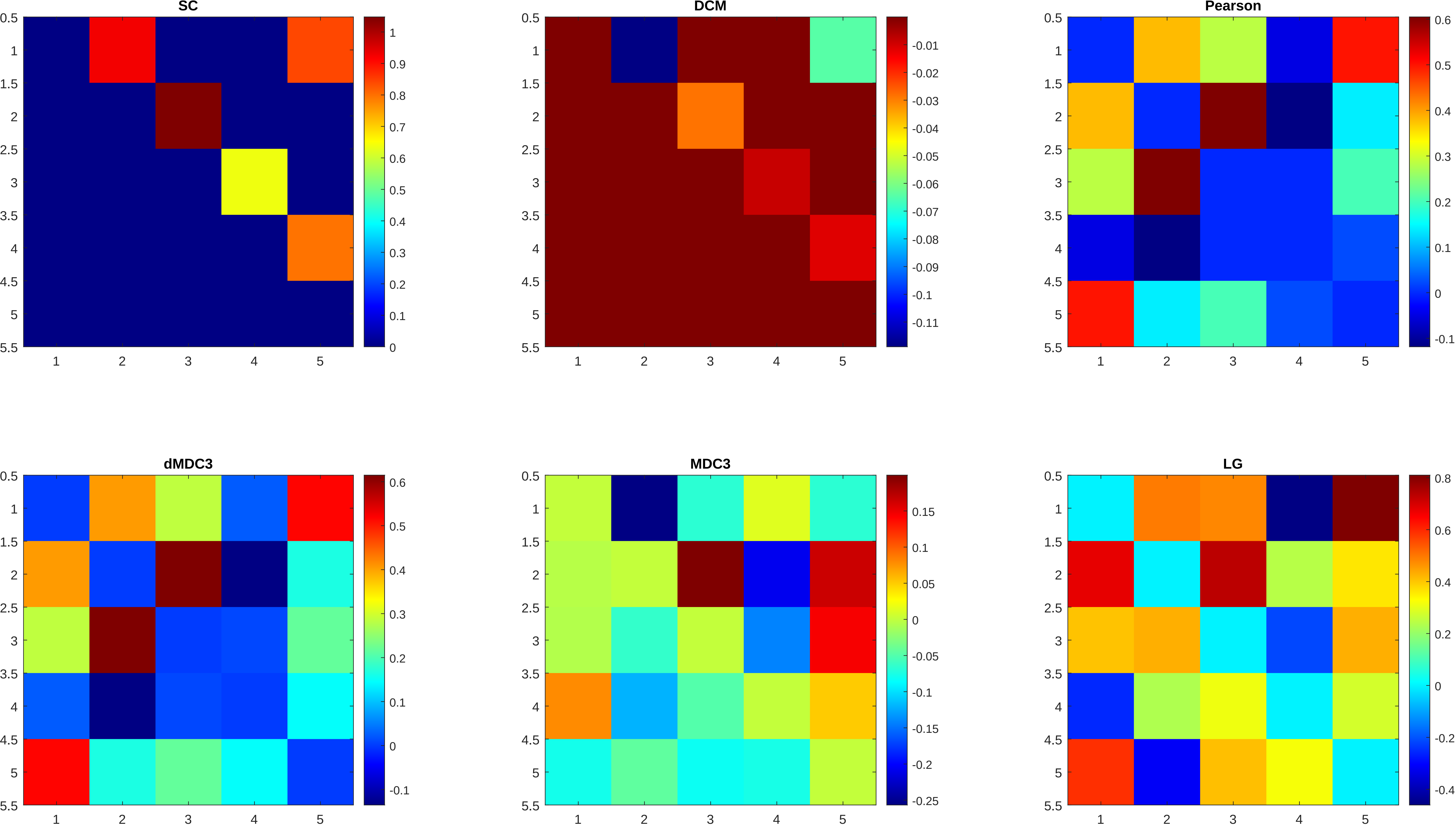

SC
0.5
1
1.5
2
2.5
3
3.5
4
4.5
5
5.5
1 2 3 4 5
1 0.9 0.8 0.7 0.6 0.5 0.4 0.3 0.2 0.1 0
DCM
-0.01 -0.02 -0.03 -0.04 -0.05 -0.06 -0.07 -0.08 -0.09 -0.1 -0.11
Pearson
0.6 0.5 0.4 0.3 0.2 0.1 0 -0.1
dMDC3
0.6 0.5 0.4 0.3 0.2 0.1 0 -0.1
MDC3
0.15 0.1 0.05 0 -0.05 -0.1 -0.15 -0.2 -0.25
LG
0.8 0.6 0.4 0.2 0 -0.2 -0.4

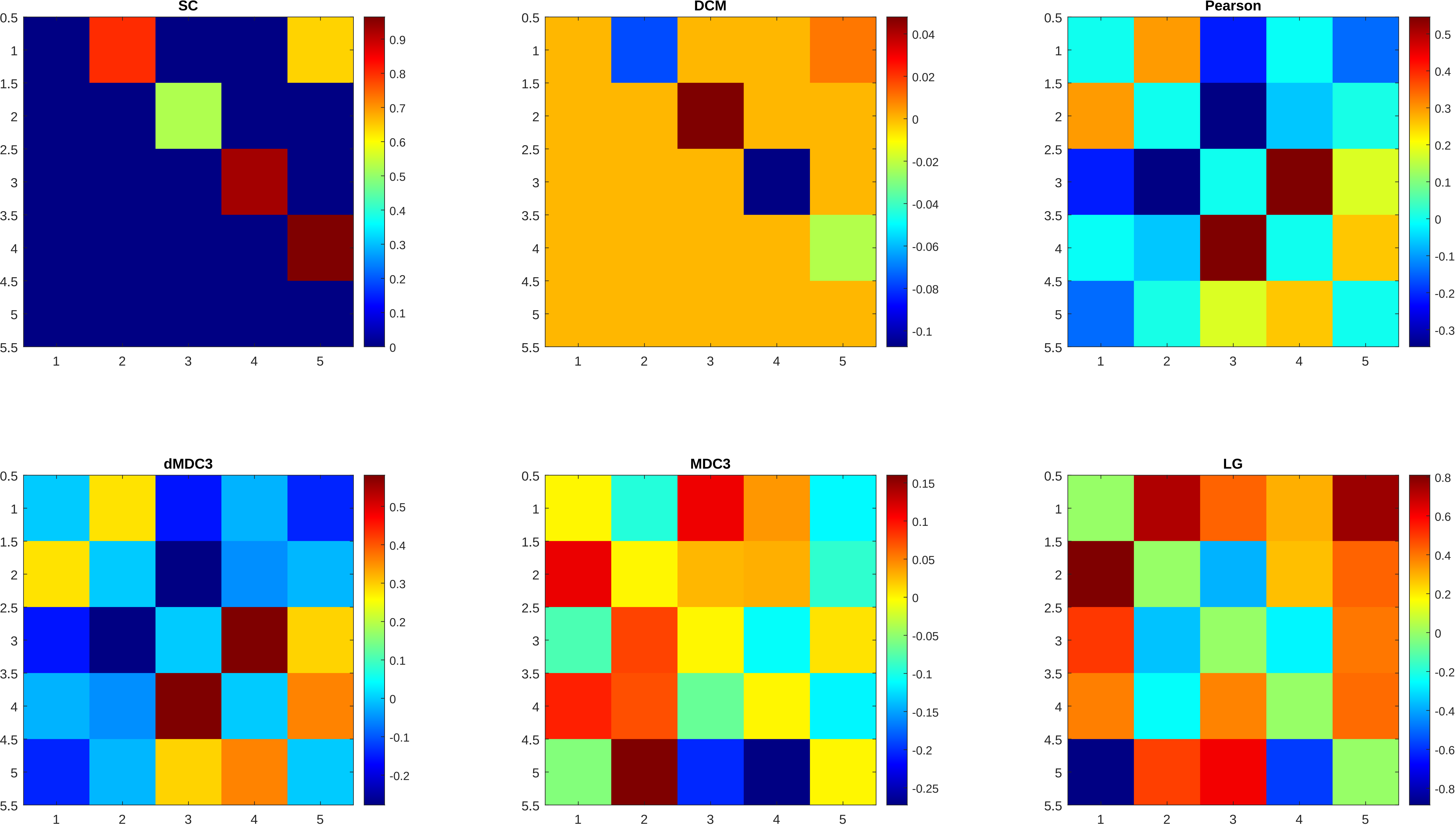
SC
DCM
Pearson
dMDC3
MDC3
LG

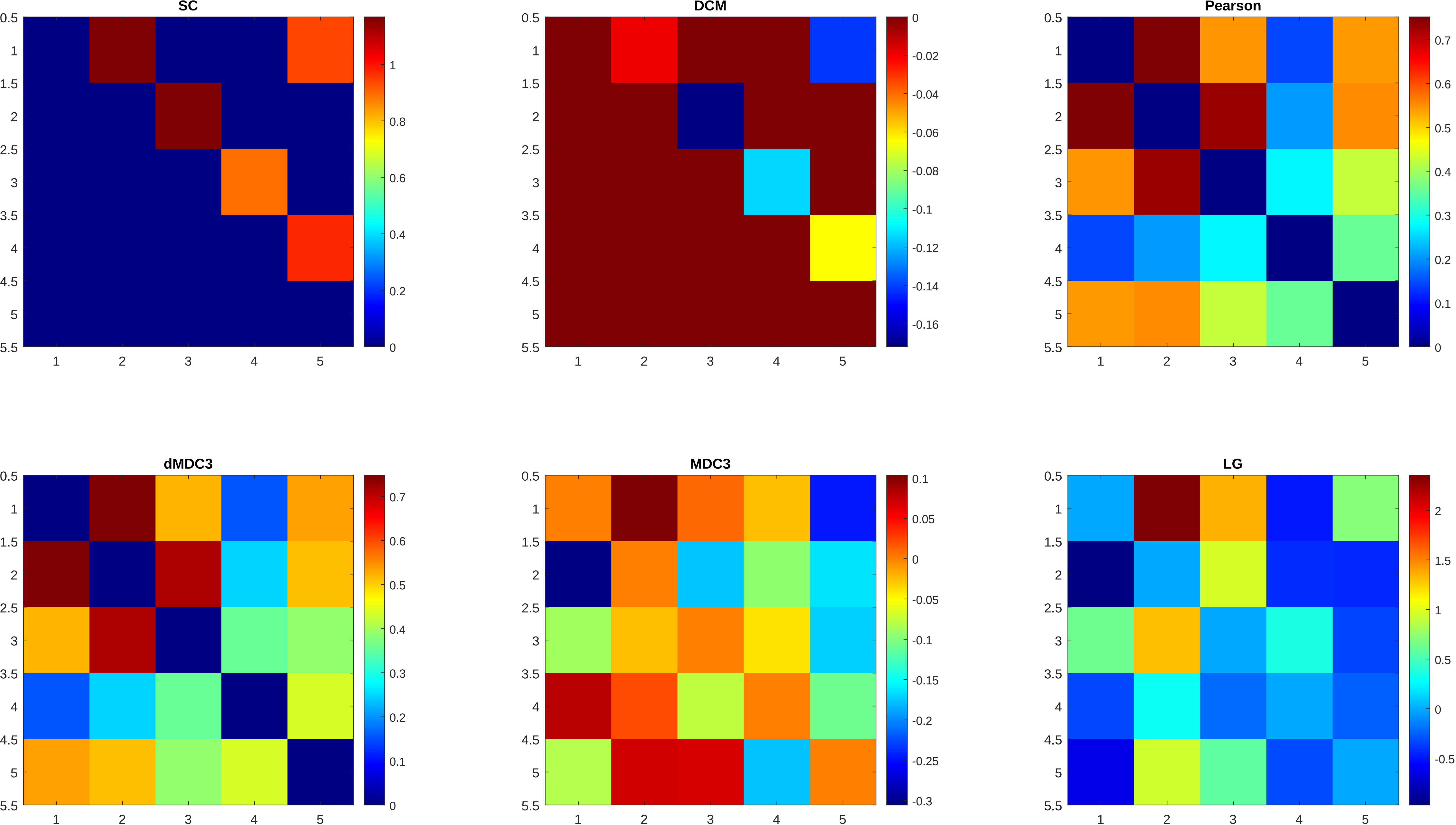
SC
DCM
Pearson
dMDC3
MDC3
LG

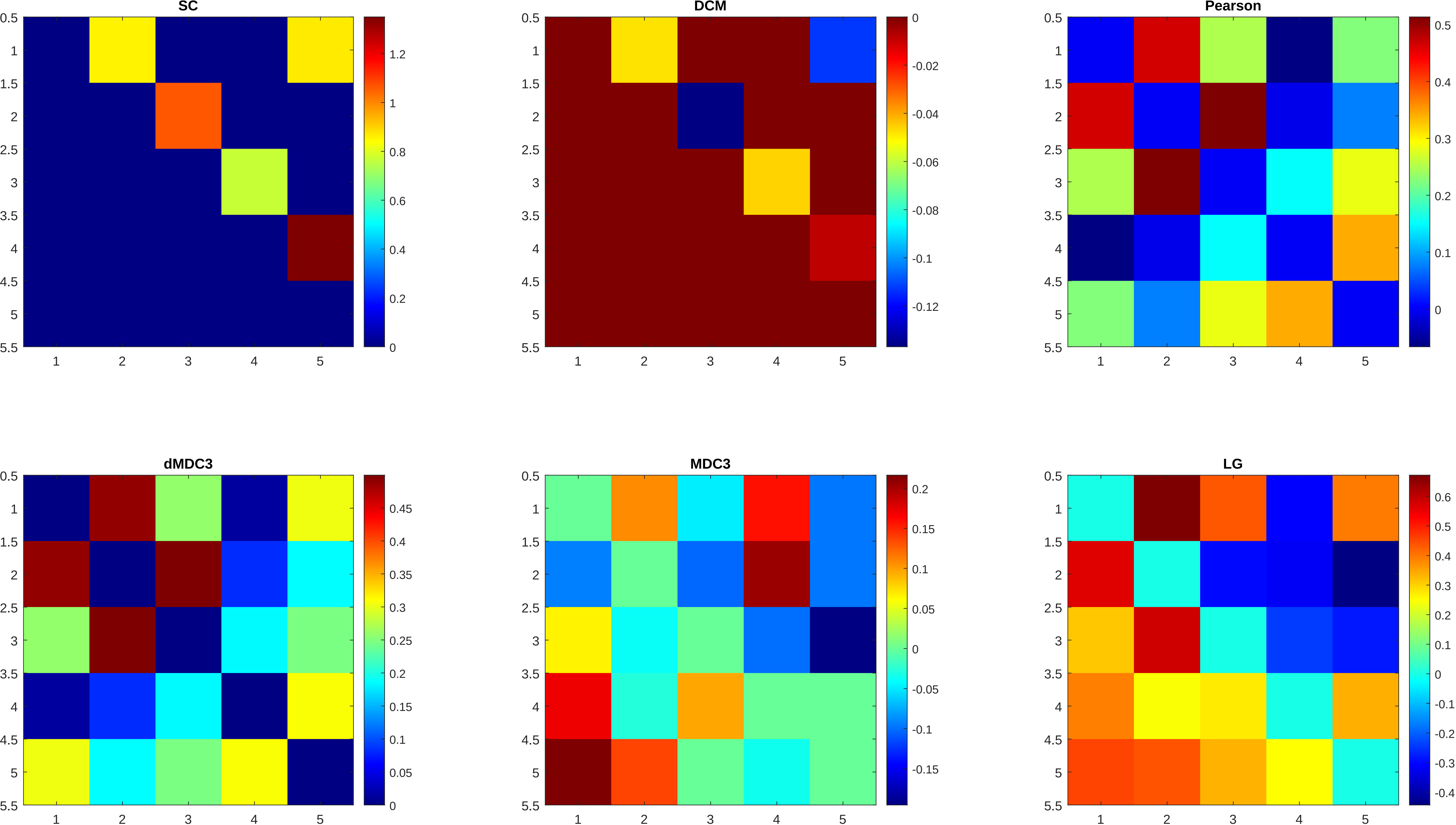
SC
DCM
Pearson
dMDC3
MDC3
LG

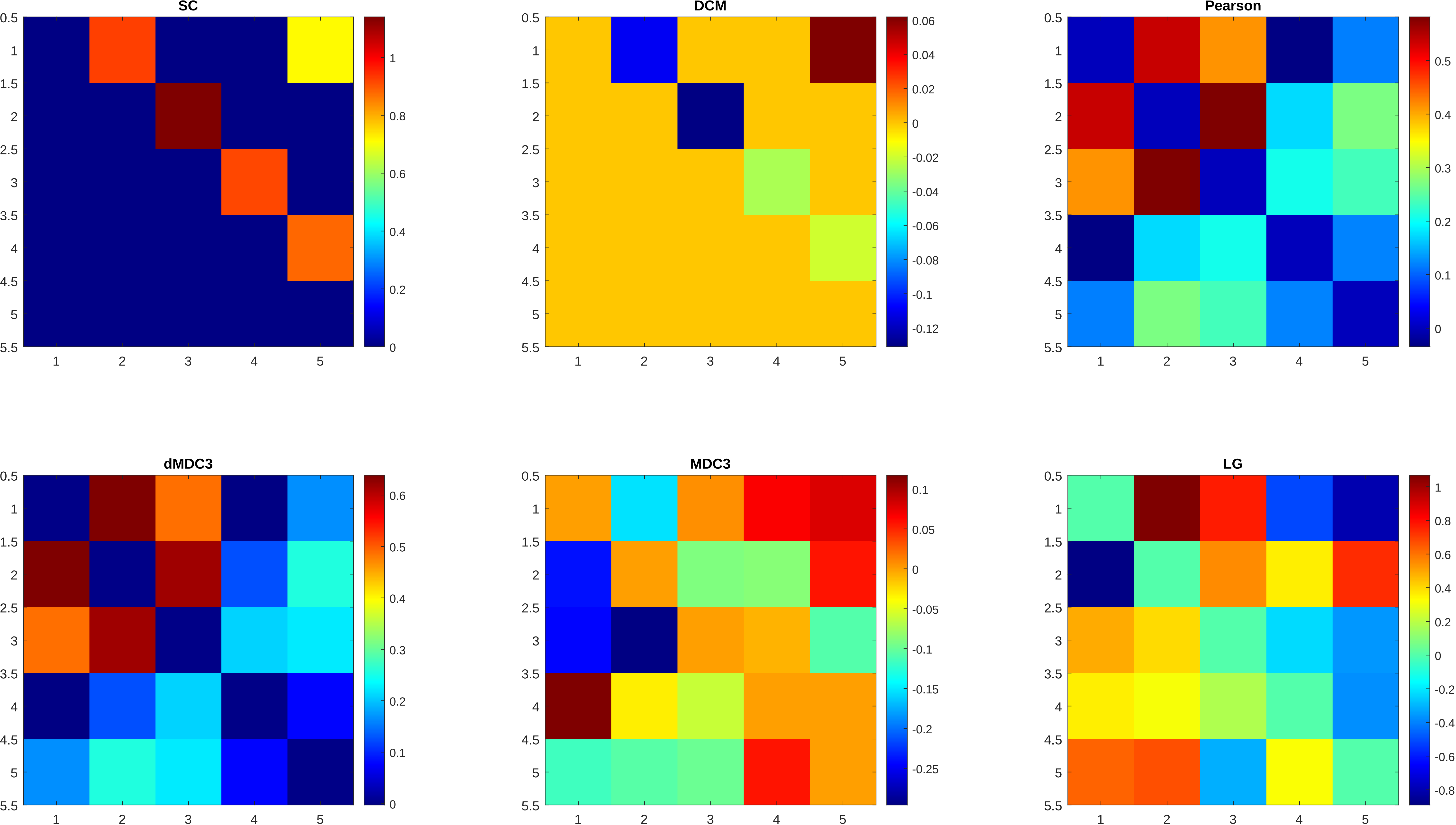

SC
DCM
Pearson
dMDC3
MDC3
LG

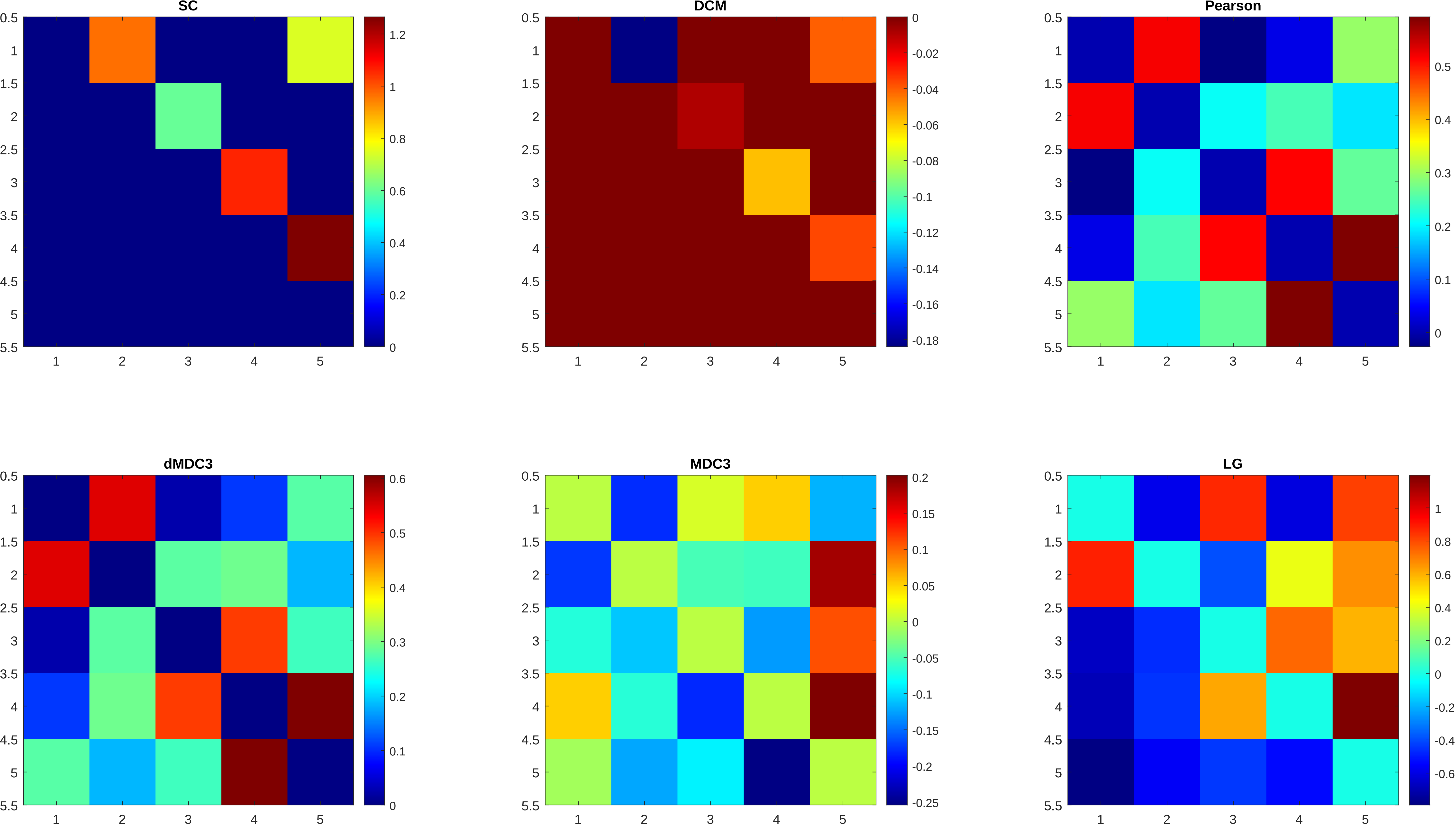
SC
DCM
Pearson
dMDC3
MDC3
LG

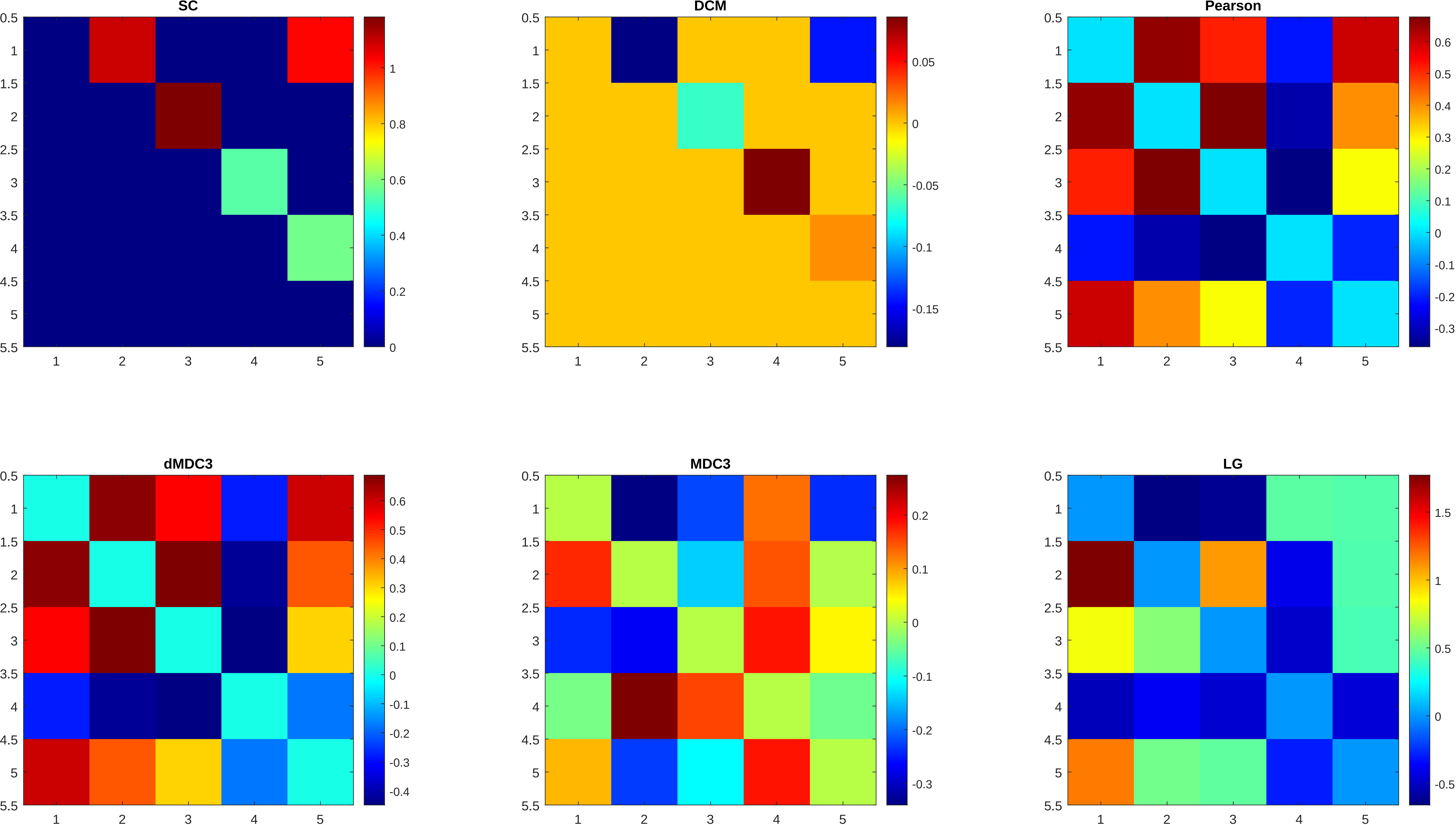

SC
DCM
Pearson
dMDC3
MDC3
LG

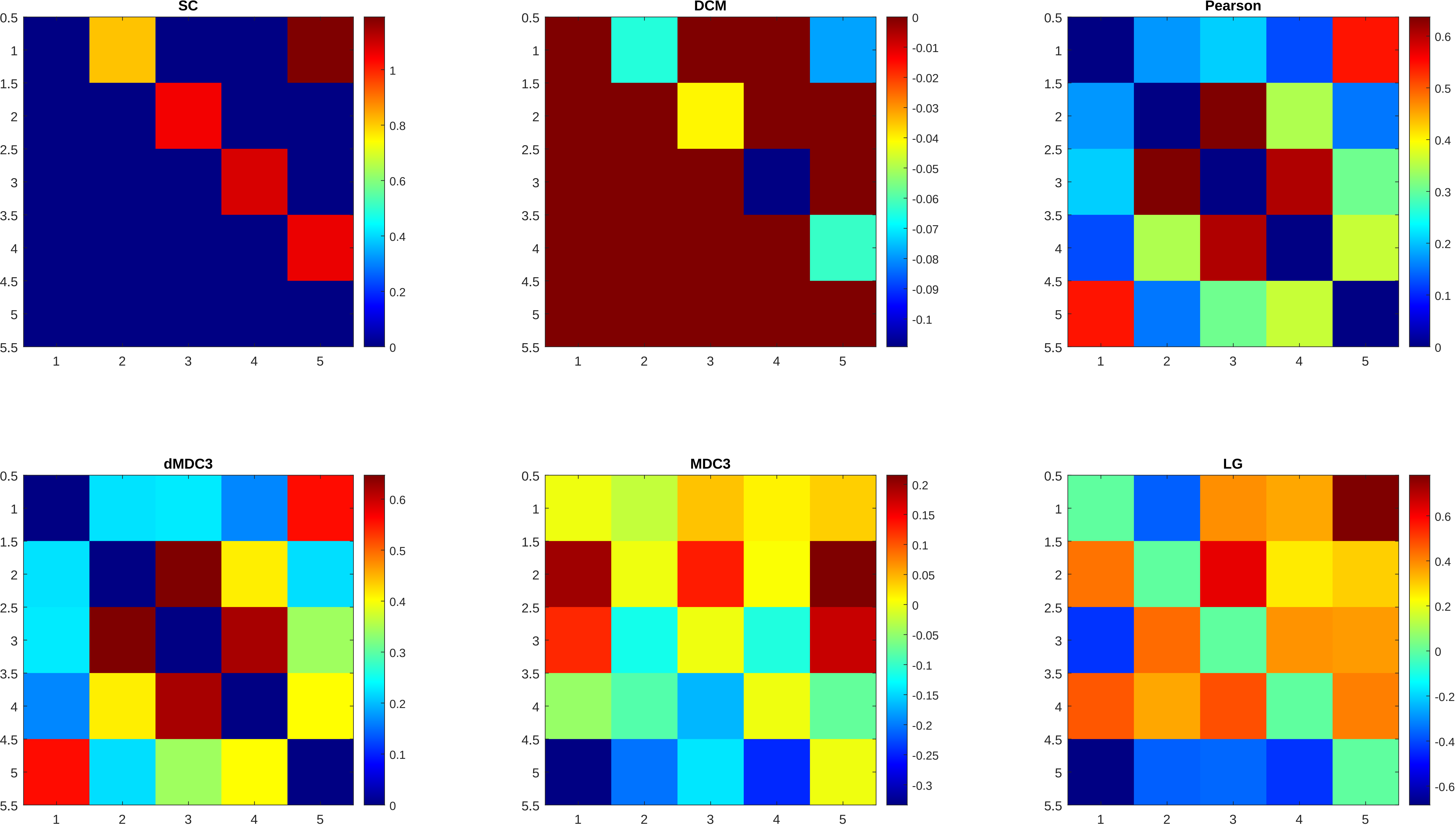
SC
DCM
Pearson
dMDC3
MDC3
LG

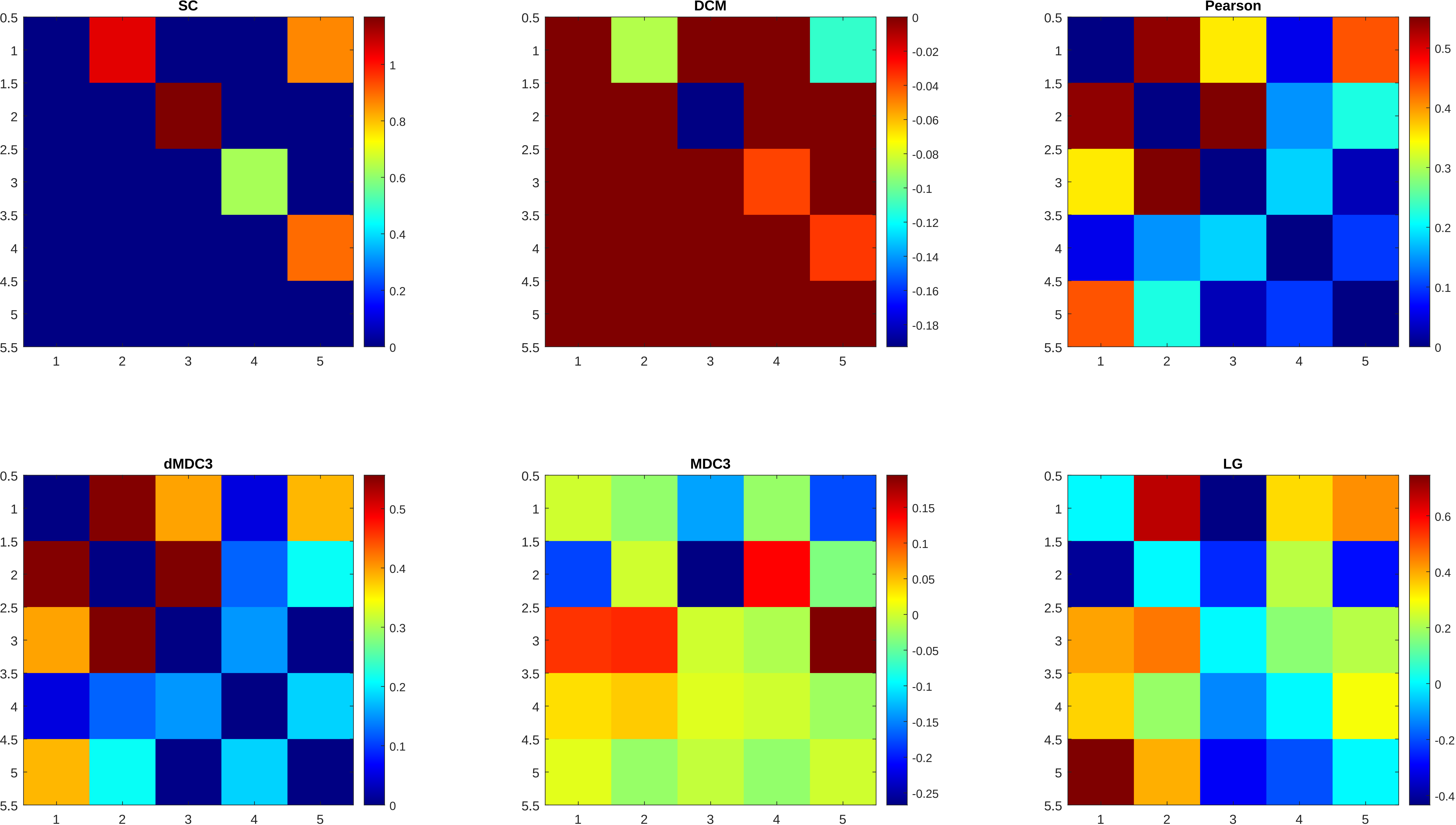
SC
DCM
Pearson
dMDC3
MDC3
LG
0.5
1
1.5
2
2.5
3
3.5
4
4.5
5
5.5
1
2
3
4
5
0
0.2
0.4
0.6
0.8
1
0
-0.02
-0.04
-0.06
-0.08
-0.1
-0.12
-0.14
-0.16
-0.18
0.1
0.3
0.5
0.15
0.05
-0.05
-0.15
-0.2
-0.25
-0.4
-0.2
0.6

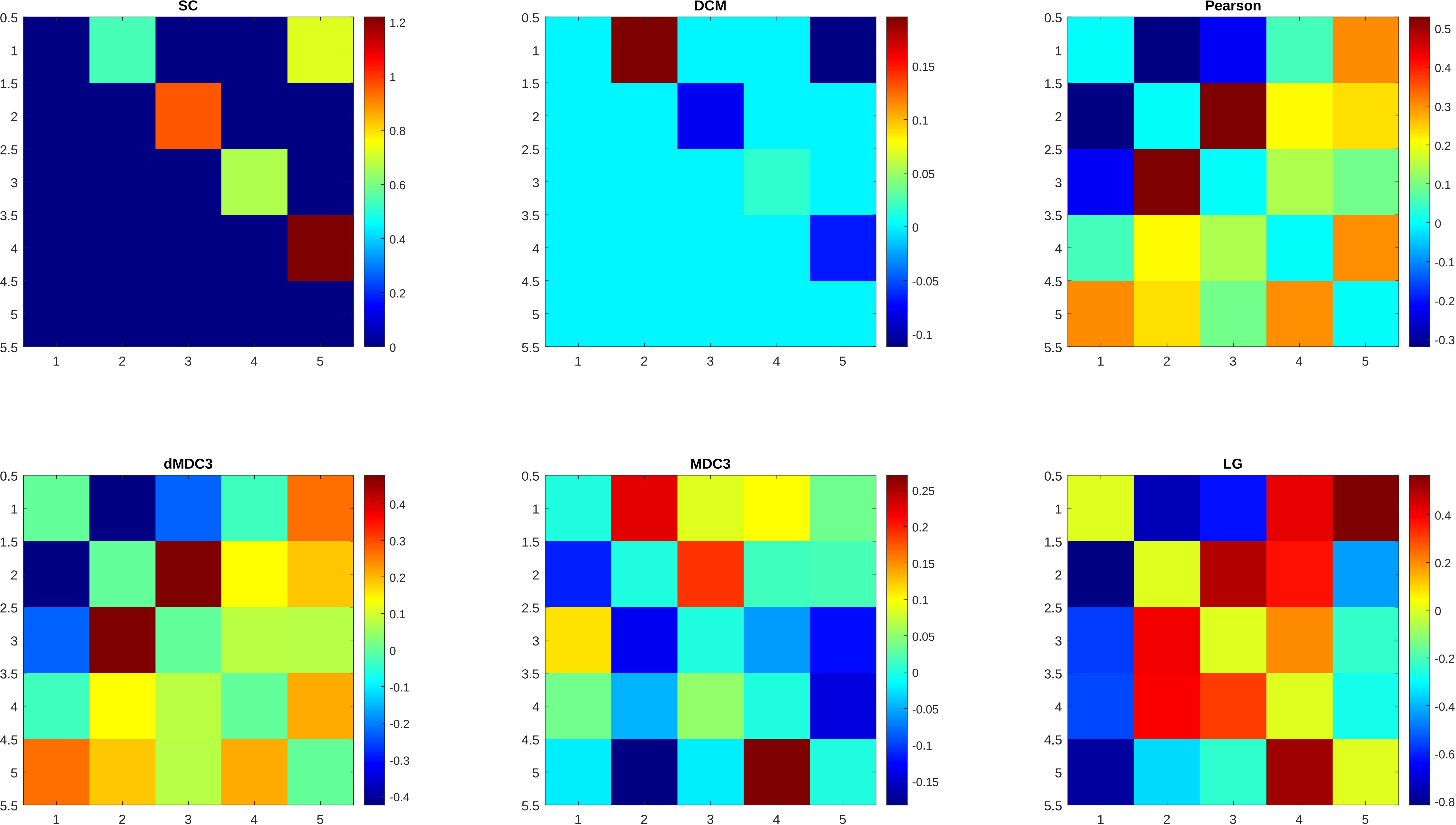
SC
DCM
Pearson
dMDC3
MDC3
LG

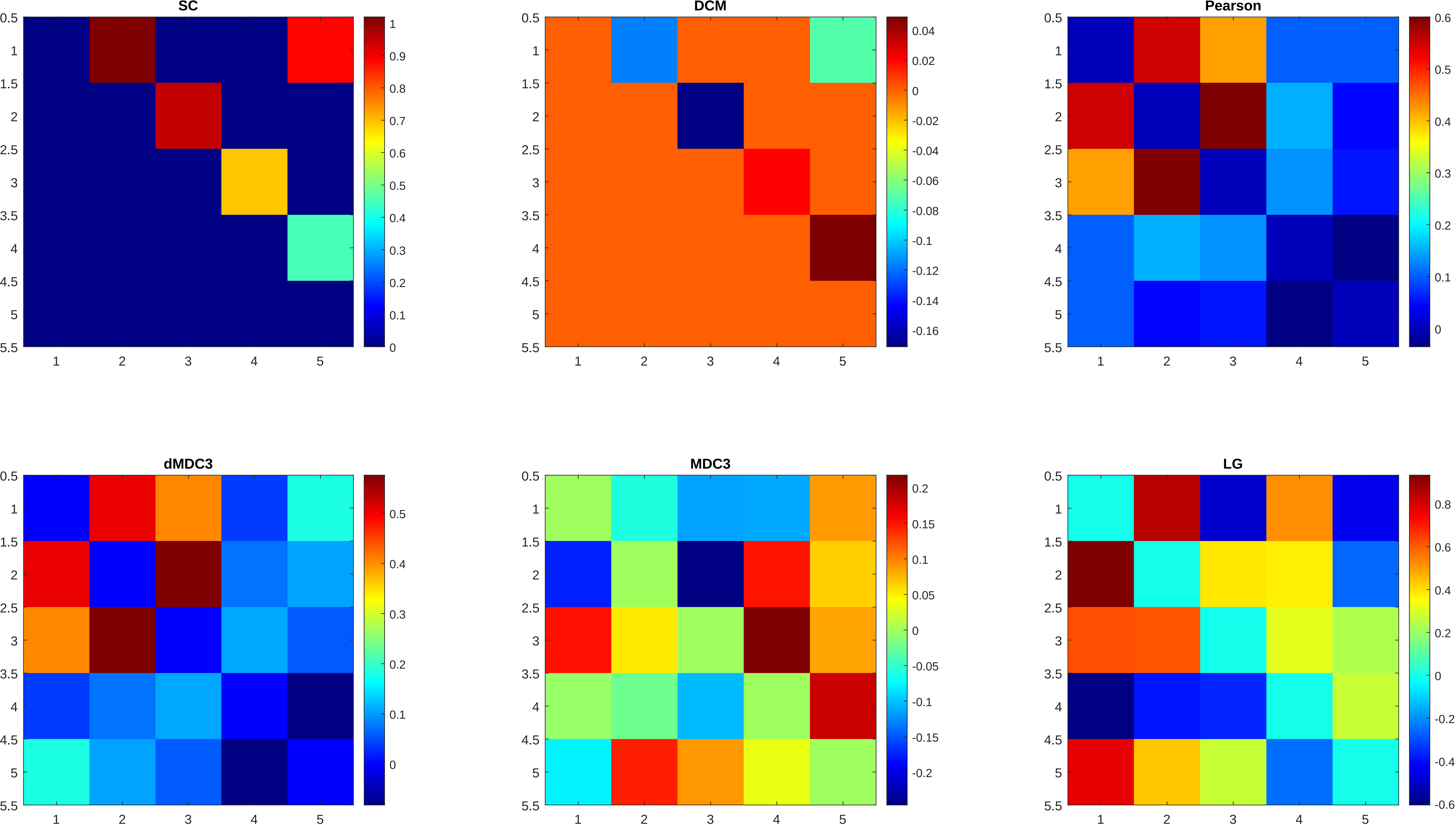
SC
DCM
Pearson
dMDC3
MDC3
LG
0.5 1 1.5 2 2.5 3 3.5 4 4.5 5 5.5
1 2 3 4 5
0 0.1 0.2 0.3 0.4 0.5 0.6 0.7 0.8 0.9 1
-0.16 -0.14 -0.12 -0.1 -0.08 -0.06 -0.04 -0.02 0 0.02 0.04
0 0.1 0.2 0.3 0.4 0.5 0.6
0 0.1 0.2 0.3 0.4 0.5
-0.2 -0.15 -0.1 -0.05 0 0.05 0.1 0.15 0.2
-0.6 -0.4 -0.2 0 0.2 0.4 0.6 0.8

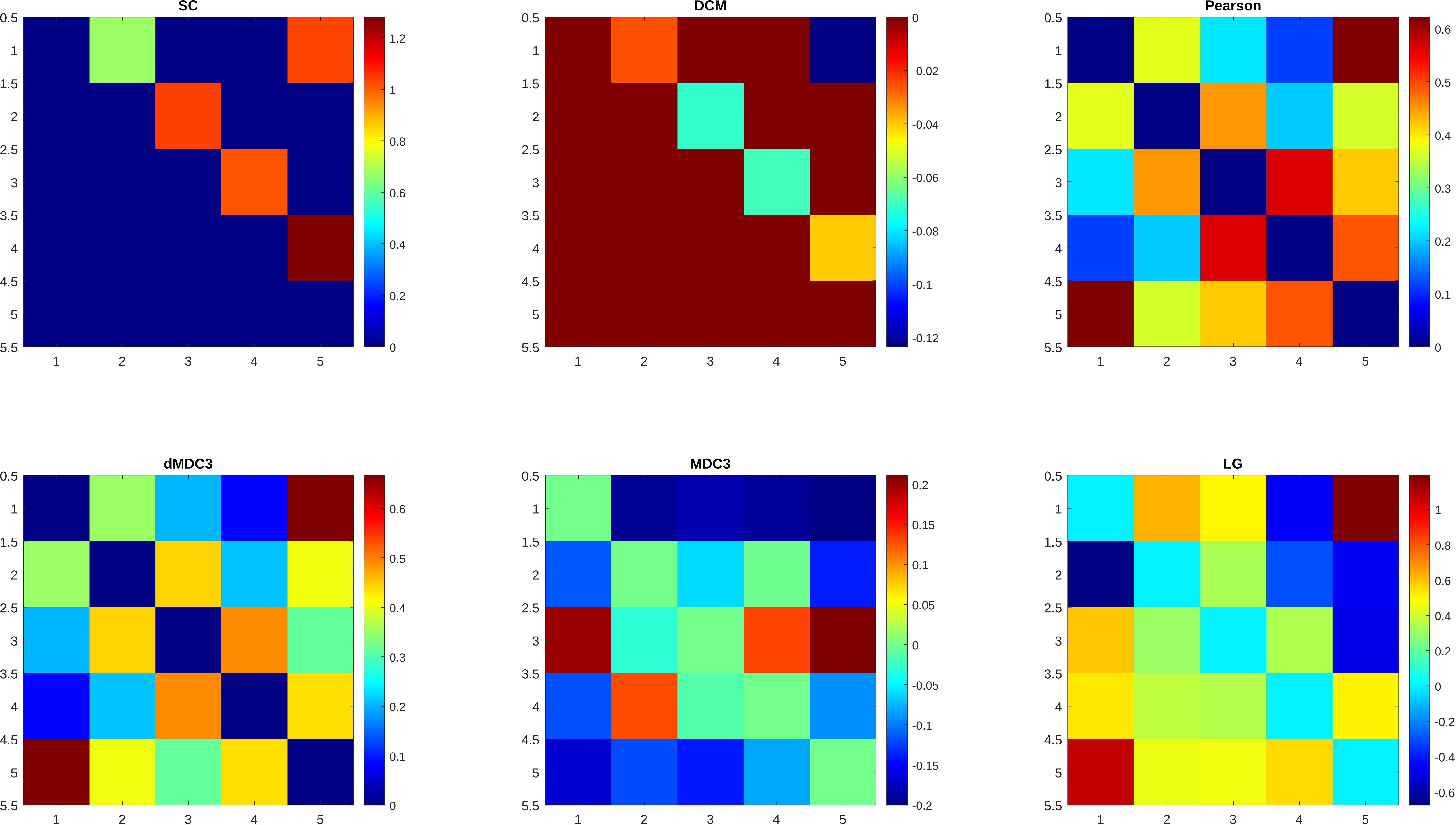
SC
DCM
Pearson
dMDC3
MDC3
LG

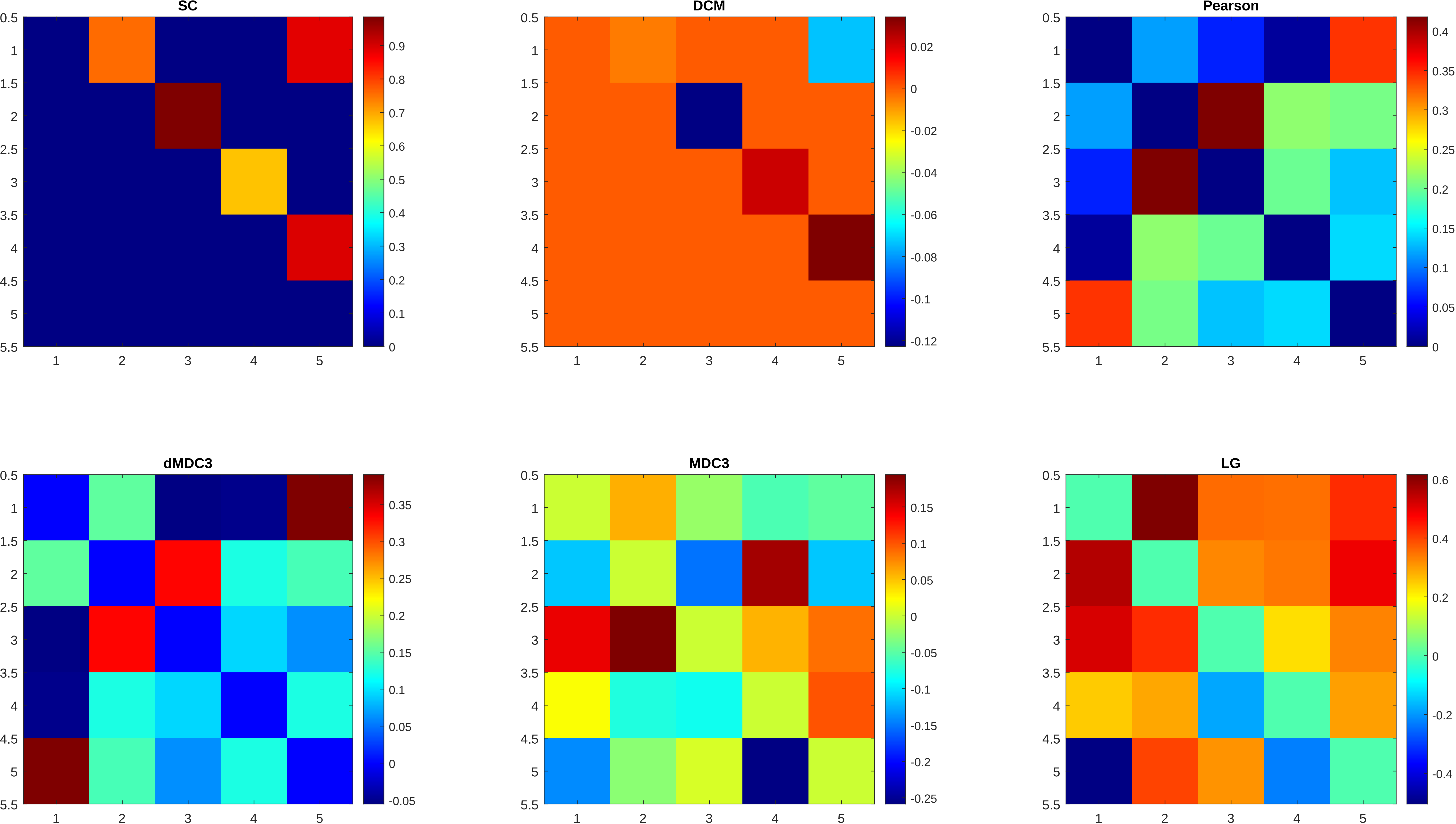

SC
DCM
Pearson
dMDC3
MDC3
LG
0.5
1
1.5
2
2.5
3
3.5
4
4.5
5
5.5
1 2 3 4 5
0 0.1 0.2 0.3 0.4 0.5 0.6 0.7 0.8 0.9
-0.12 -0.1 -0.08 -0.06 -0.04 -0.02 0 0.02
0 0.05 0.1 0.15 0.2 0.25 0.3 0.35 0.4
-0.05 0 0.05 0.1 0.15 0.2 0.25 0.3 0.35
-0.25 -0.2 -0.15 -0.1 -0.05 0 0.05 0.1 0.15
-0.4 -0.2 0 0.2 0.4 0.6

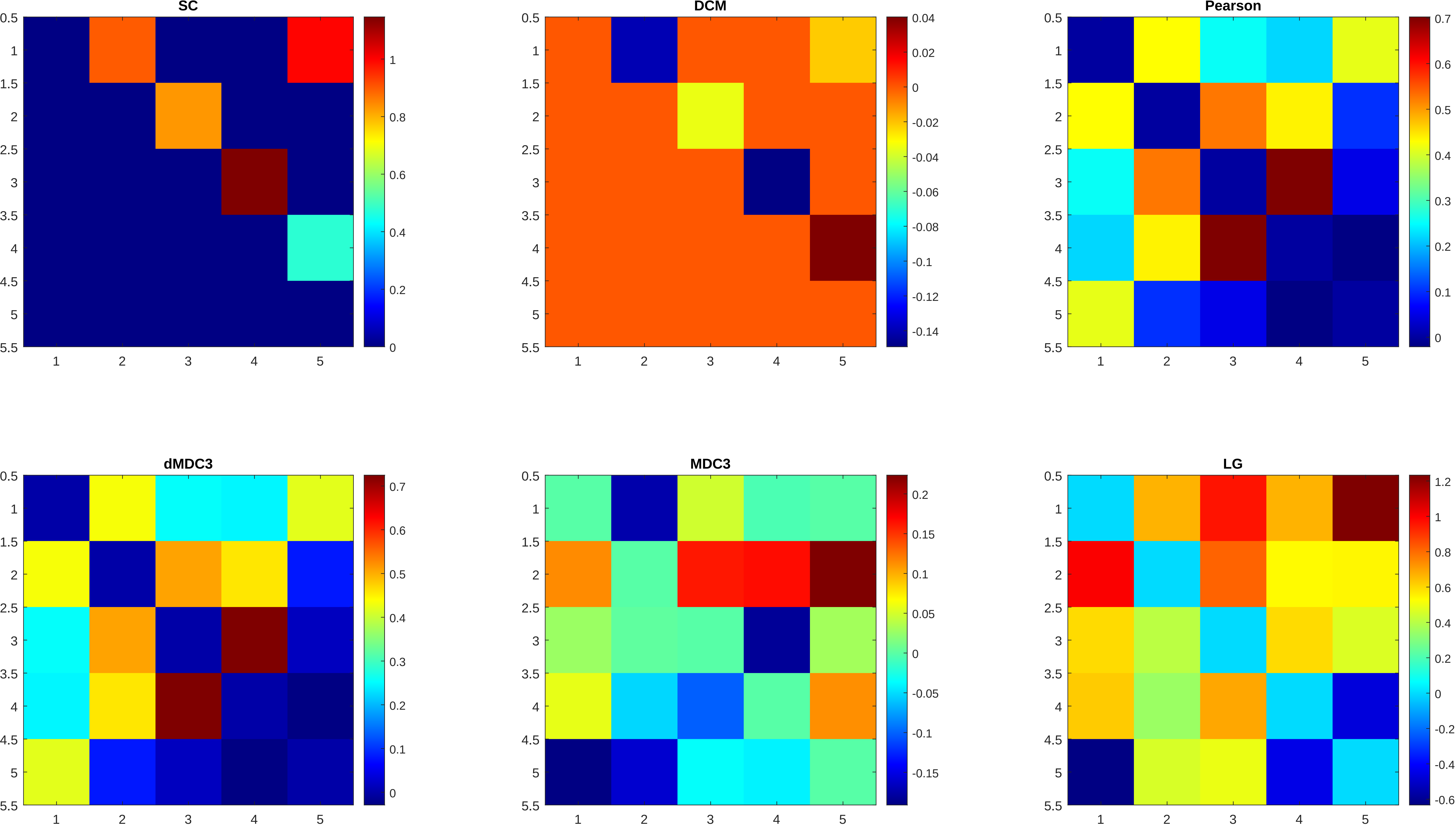
SC
DCM
Pearson
dMDC3
MDC3
LG
0.5
1
1.5
2
2.5
3
3.5
4
4.5
5
5.5
1
2
3
4
5
0
0.2
0.4
0.6
0.8
1
0.04
0.02
0
-0.02
-0.04
-0.06
-0.08
-0.1
-0.12
-0.14
0.1
0.3
0.5
0.7
0.15
0.05
-0.05
-0.15
1.2
-0.2
-0.4
-0.6

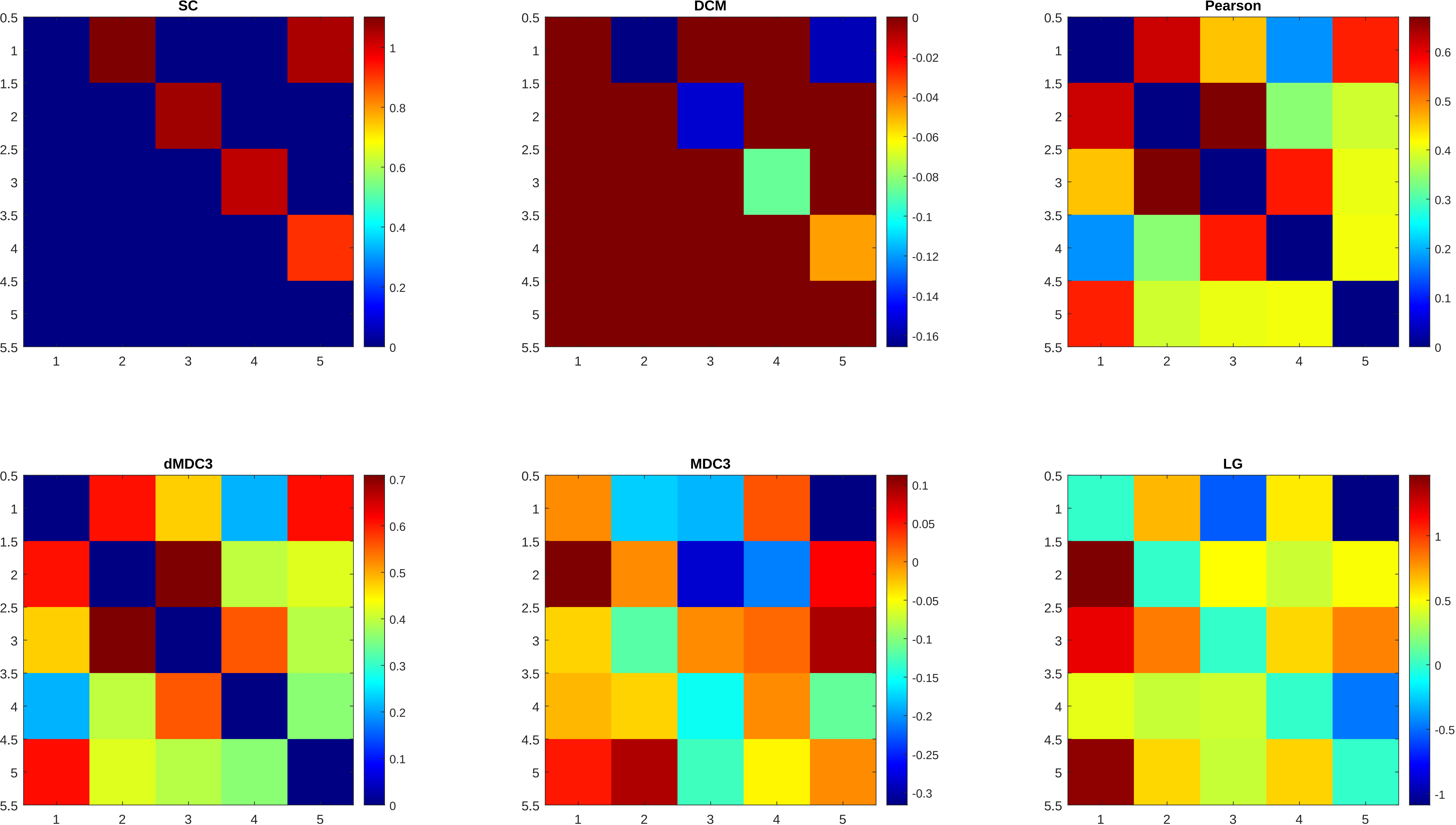
SC
DCM
Pearson
dMDC3
MDC3
LG

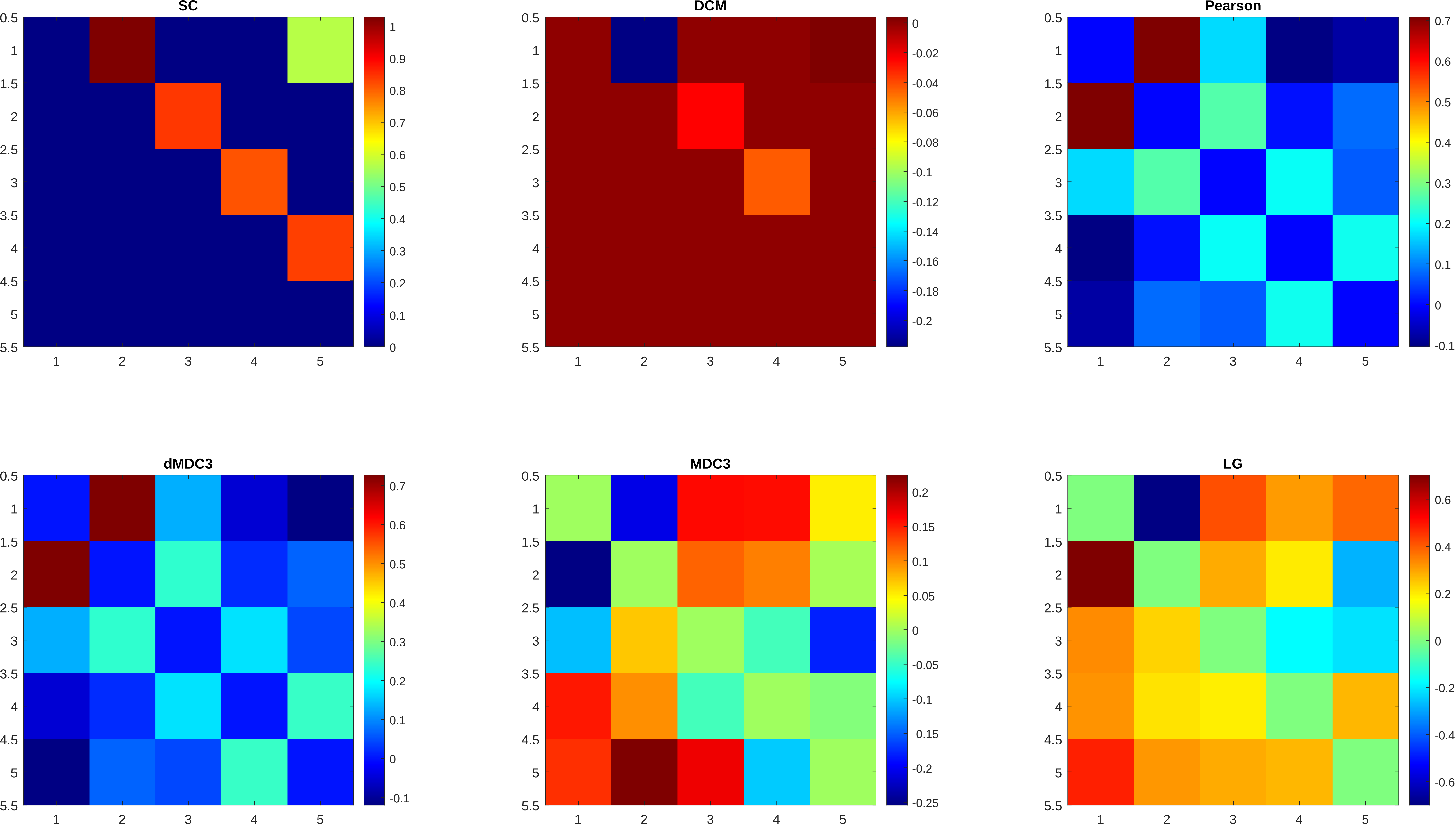
SC
DCM
Pearson
dMDC3
MDC3
LG
0.5
1
1.5
2
2.5
3
3.5
4
4.5
5
5.5
1
2
3
4
5
0
0.1
0.2
0.3
0.4
0.5
0.6
0.7
0.8
0.9
1
0
-0.02
-0.04
-0.06
-0.08
-0.1
-0.12
-0.14
-0.16
-0.18
-0.2
-0.1
0
0.1
0.2
0.3
0.4
0.5
0.6
0.7
-0.1
0
0.1
0.2
0.3
0.4
0.5
0.6
0.7
-0.25
-0.2
-0.15
-0.1
-0.05
0
0.05
0.1
0.15
0.2
-0.6
-0.4
-0.2
0
0.2
0.4
0.6

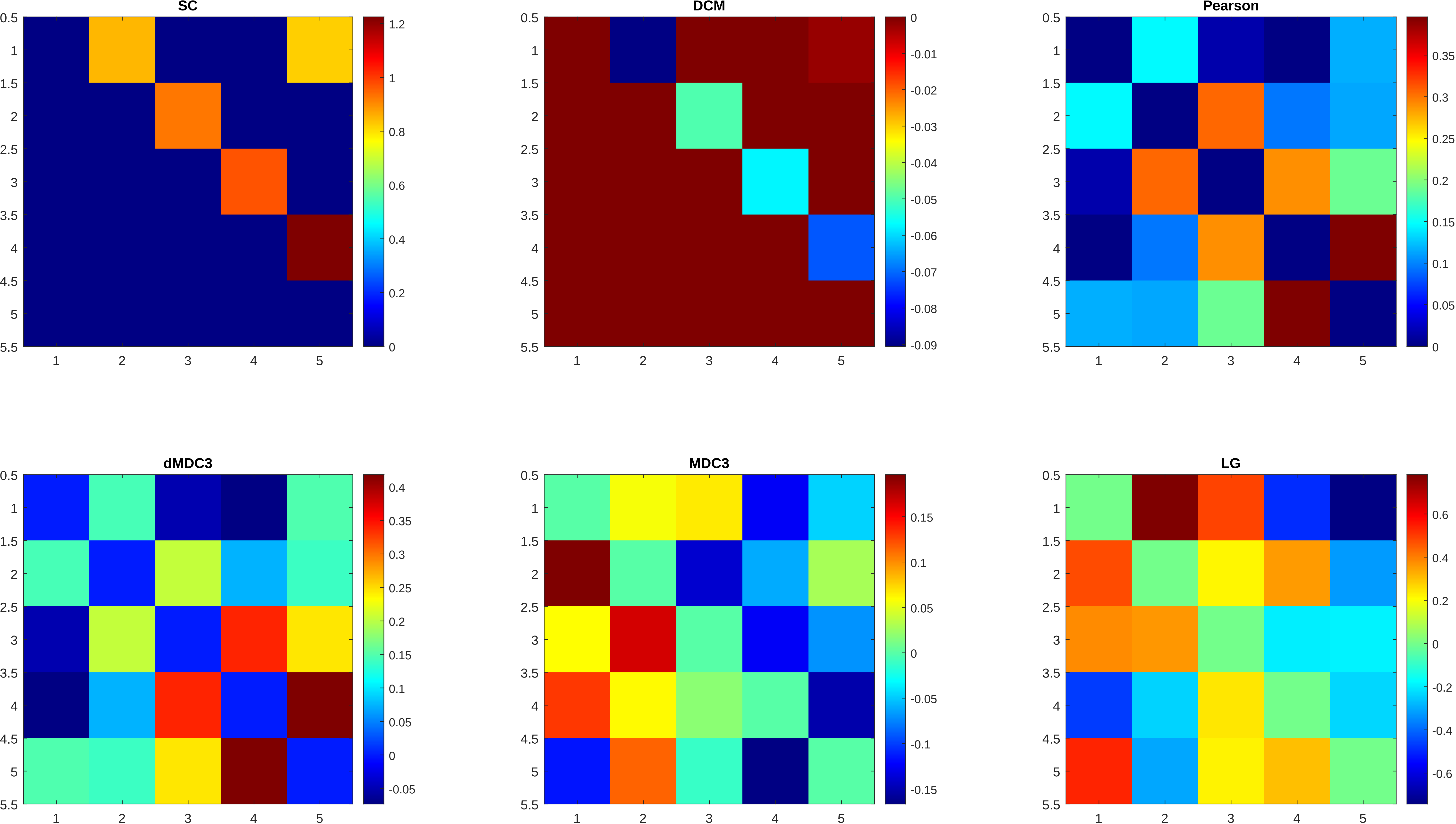

SC
0.5 1 1.5 2 2.5 3 3.5 4 4.5 5 5.5
1 2 3 4 5
0 0.2 0.4 0.6 0.8 1 1.2
DCM
0.5 1 1.5 2 2.5 3 3.5 4 4.5 5 5.5
1 2 3 4 5
0 -0.01 -0.02 -0.03 -0.04 -0.05 -0.06 -0.07 -0.08 -0.09
Pearson
0.5 1 1.5 2 2.5 3 3.5 4 4.5 5 5.5
1 2 3 4 5
0 0.05 0.1 0.15 0.2 0.25 0.3 0.35
dMDC3
0.5 1 1.5 2 2.5 3 3.5 4 4.5 5 5.5
1 2 3 4 5
-0.05 0 0.05 0.1 0.15 0.2 0.25 0.3 0.35 0.4
MDC3
0.5 1 1.5 2 2.5 3 3.5 4 4.5 5 5.5
1 2 3 4 5
-0.15 -0.1 -0.05 0 0.05 0.1 0.15
LG
0.5 1 1.5 2 2.5 3 3.5 4 4.5 5 5.5
1 2 3 4 5
-0.6 -0.4 -0.2 0 0.2 0.4 0.6

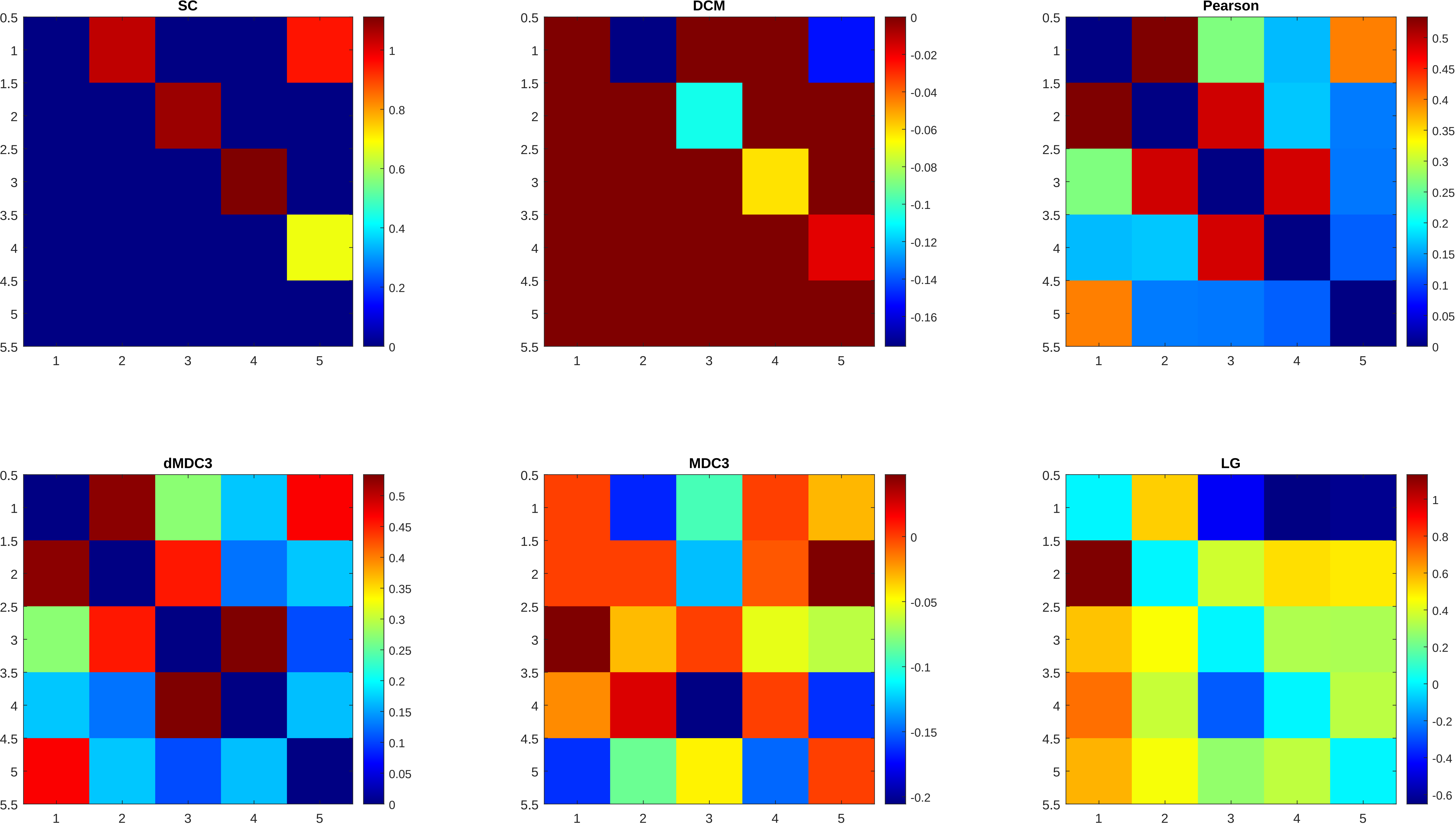
SC
DCM
Pearson
dMDC3
MDC3
LG

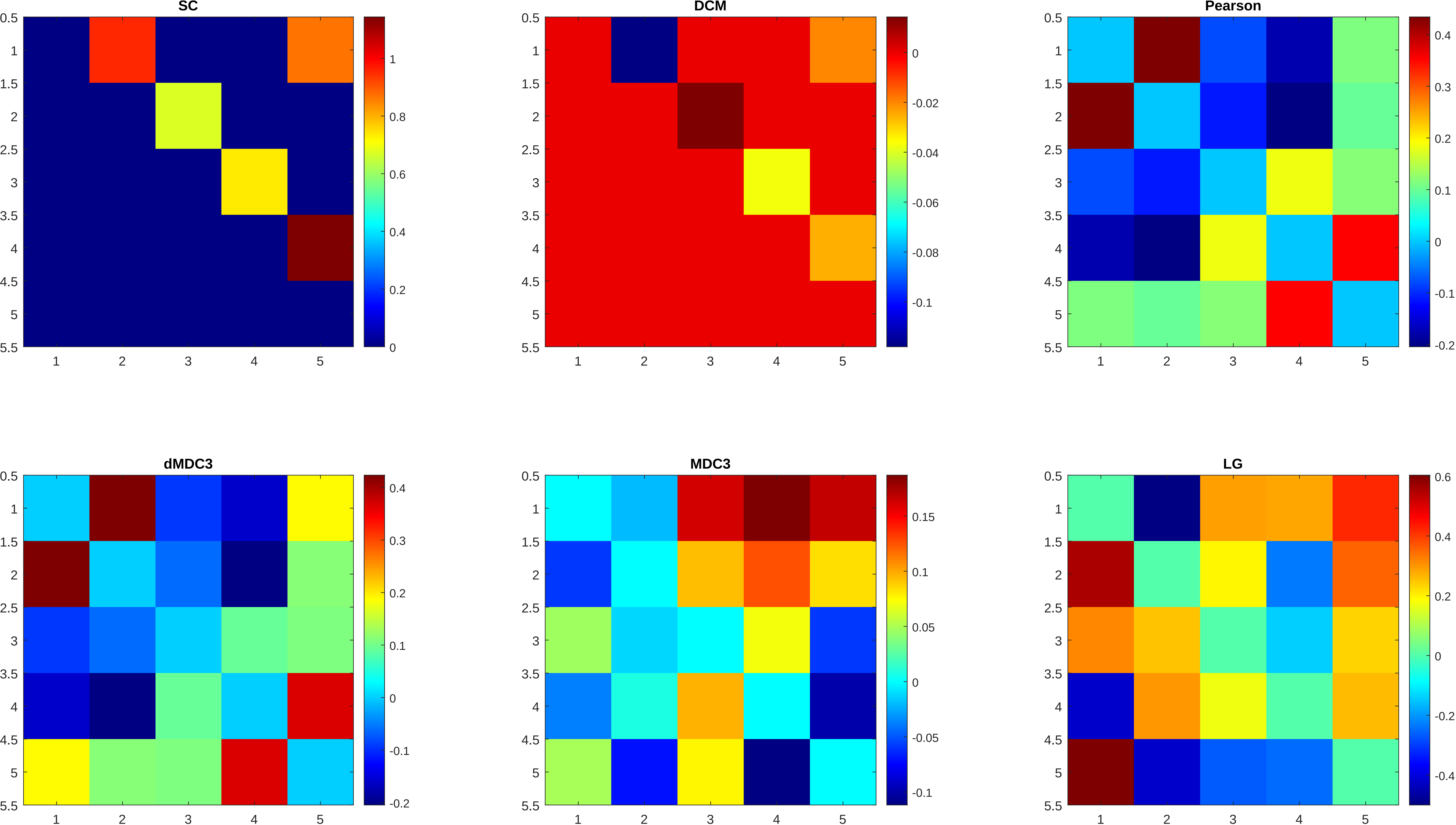

SC
DCM
Pearson
dMDC3
MDC3
LG

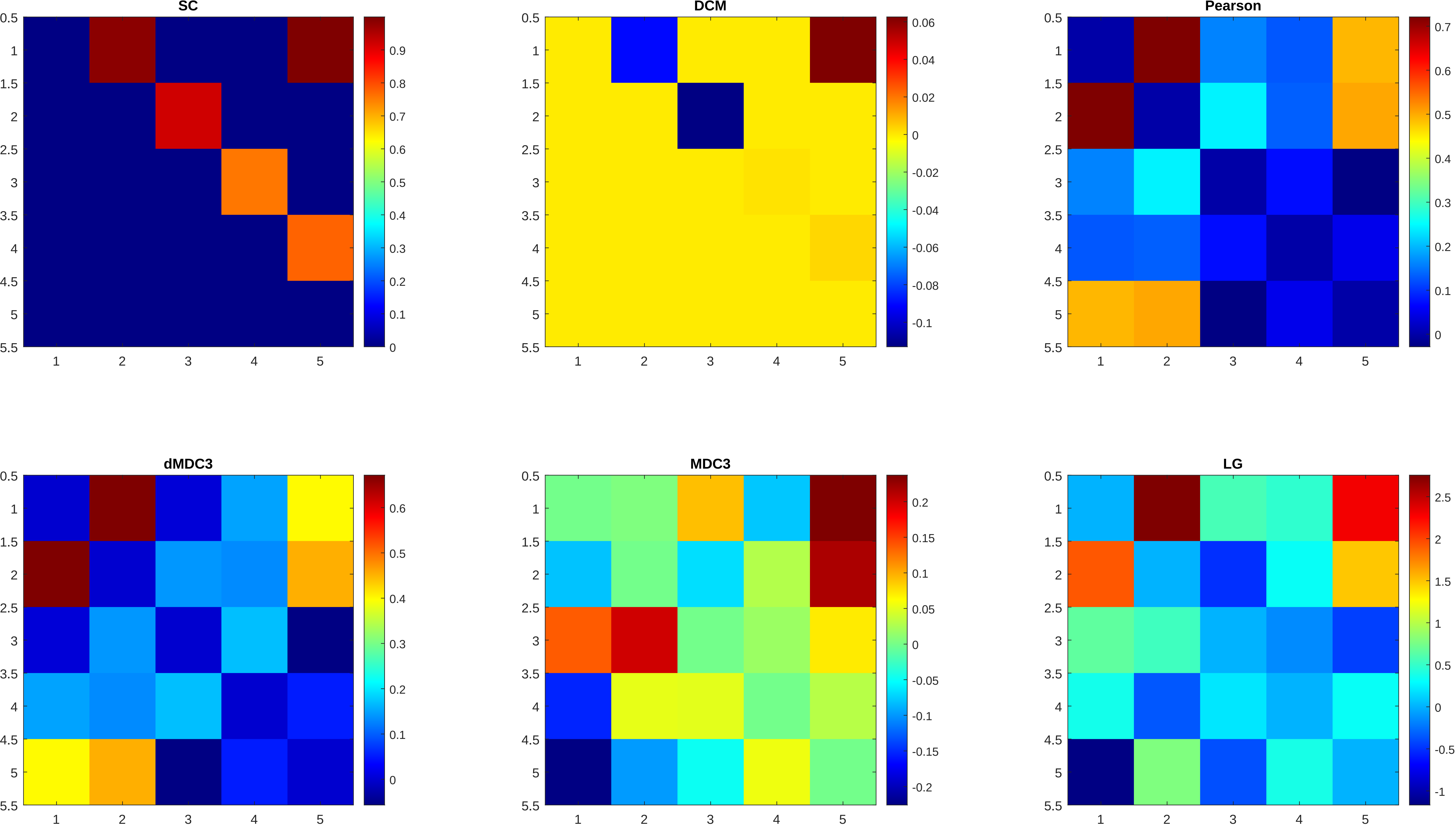

SC
DCM
Pearson
dMDC3
MDC3
LG

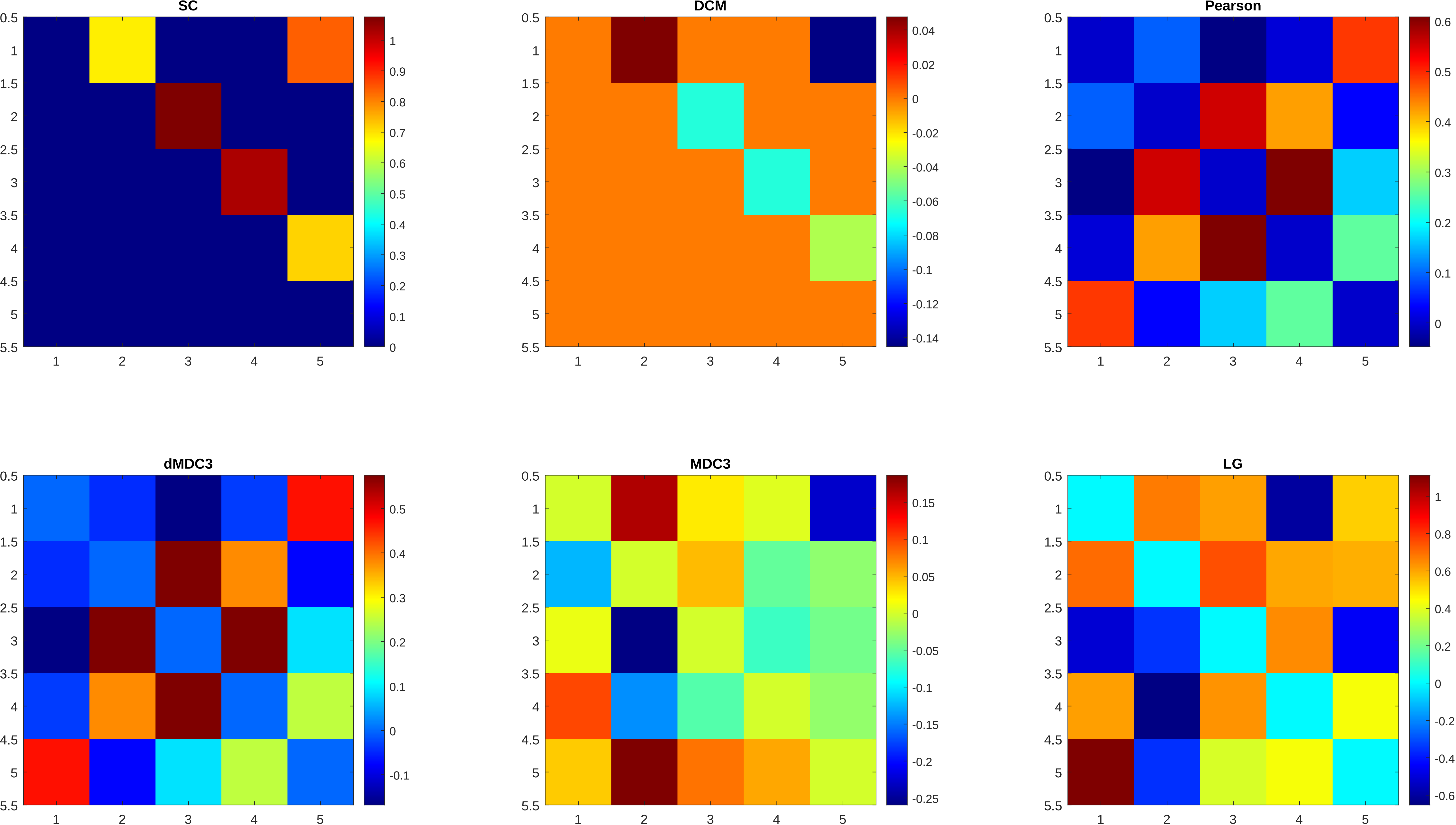
SC
DCM
Pearson
dMDC3
MDC3
LG
0.5
1
1.5
2
2.5
3
3.5
4
4.5
5
5.5
1 2 3 4 5
0 0.1 0.2 0.3 0.4 0.5 0.6 0.7 0.8 0.9 1
-0.14 -0.12 -0.1 -0.08 -0.06 -0.04 -0.02 0 0.02 0.04
0 0.1 0.2 0.3 0.4 0.5 0.6
-0.1 0 0.1 0.2 0.3 0.4 0.5
-0.25 -0.2 -0.15 -0.1 -0.05 0 0.05 0.1 0.15
-0.6 -0.4 -0.2 0 0.2 0.4 0.6 0.8 1

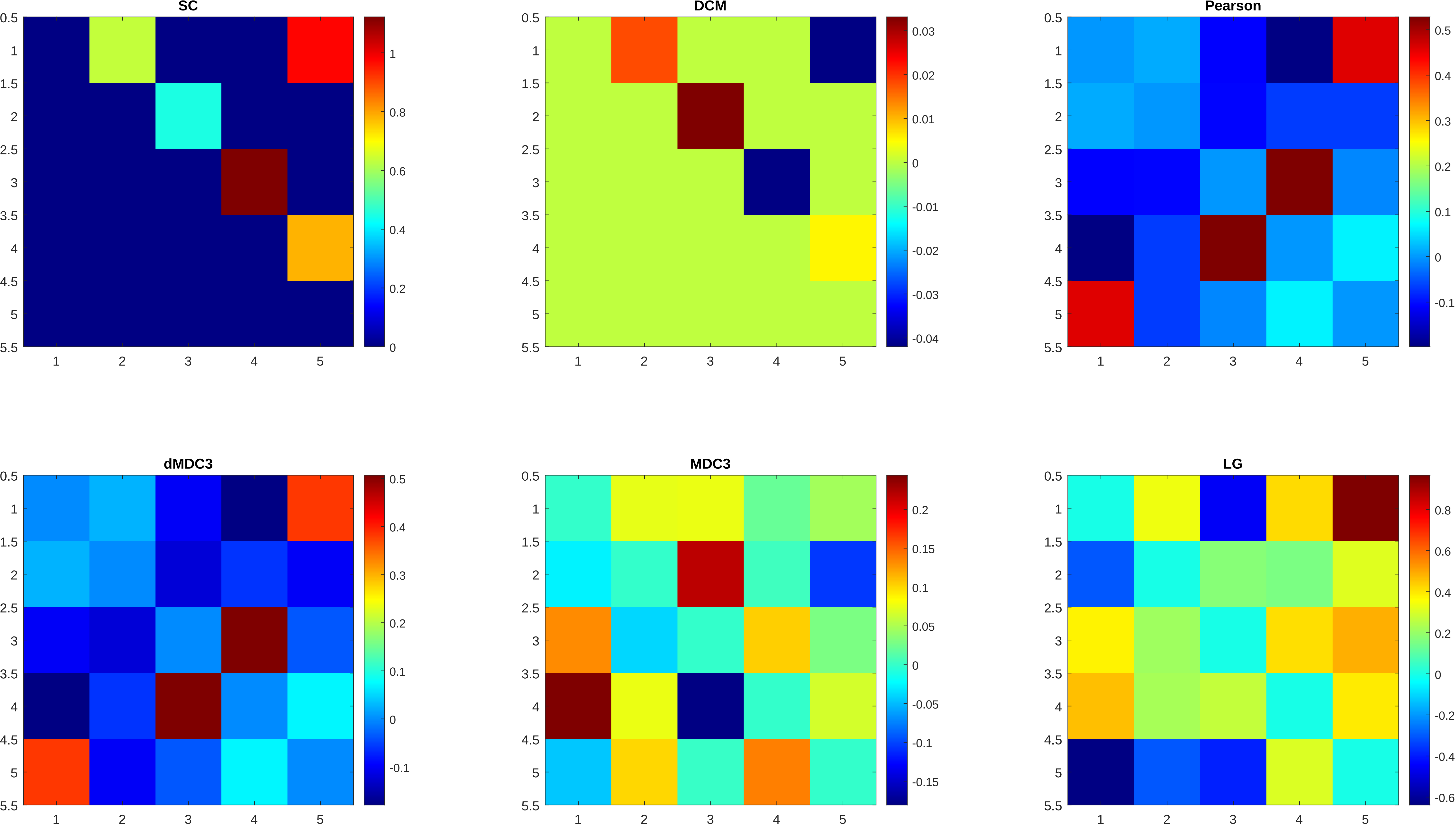
SC
DCM
Pearson
dMDC3
MDC3
LG

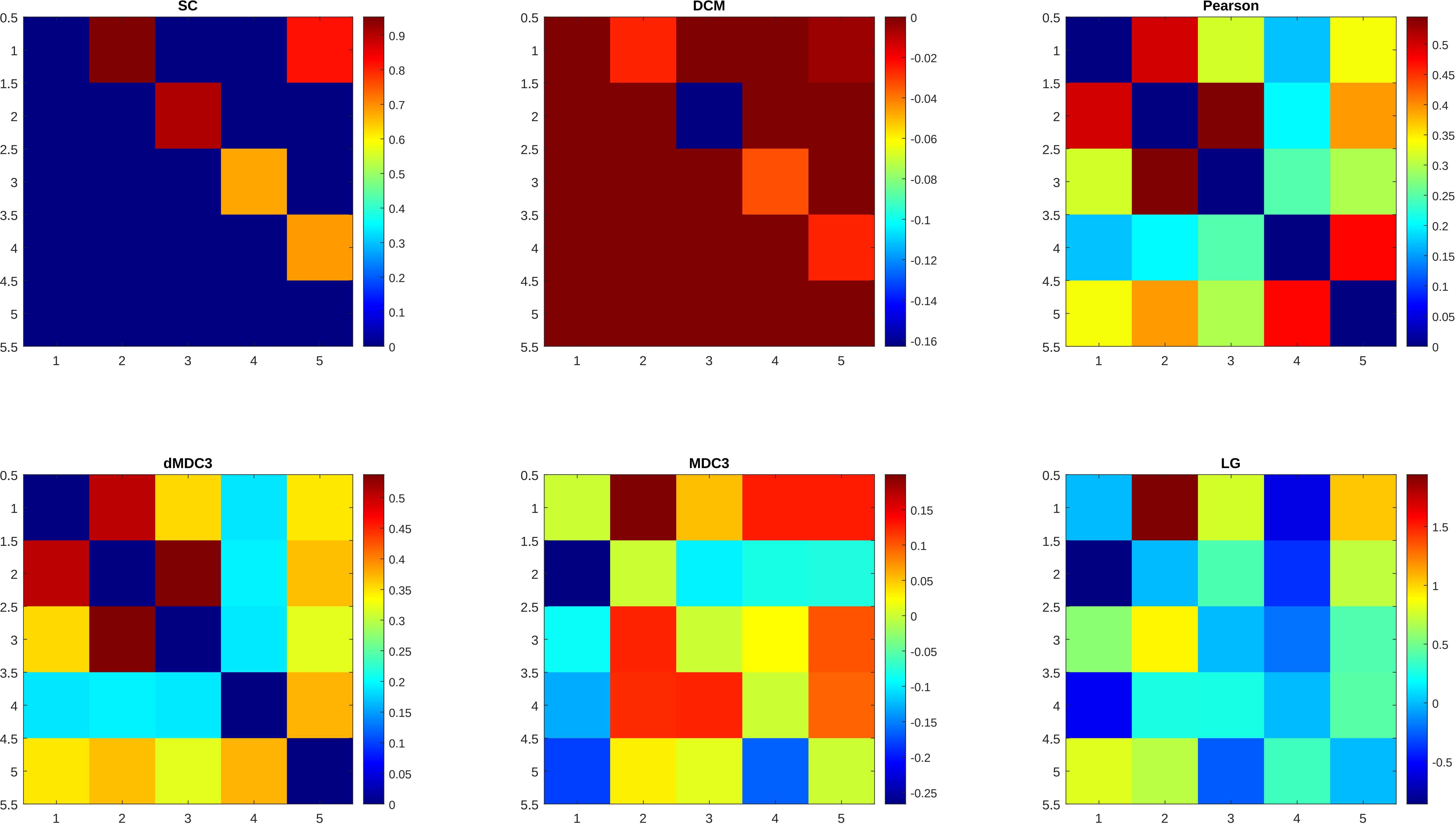
SC
DCM
Pearson
dMDC3
MDC3
LG
0.5
1
1.5
2
2.5
3
3.5
4
4.5
5
5.5
1
2
3
4
5
0
0.1
0.2
0.3
0.4
0.5
0.6
0.7
0.8
0.9
-0.02
-0.04
-0.06
-0.08
-0.1
-0.12
-0.14
-0.16
0.05
0.15
0.25
0.35
0.45
-0.05
-0.15
-0.2
-0.25
-0.5

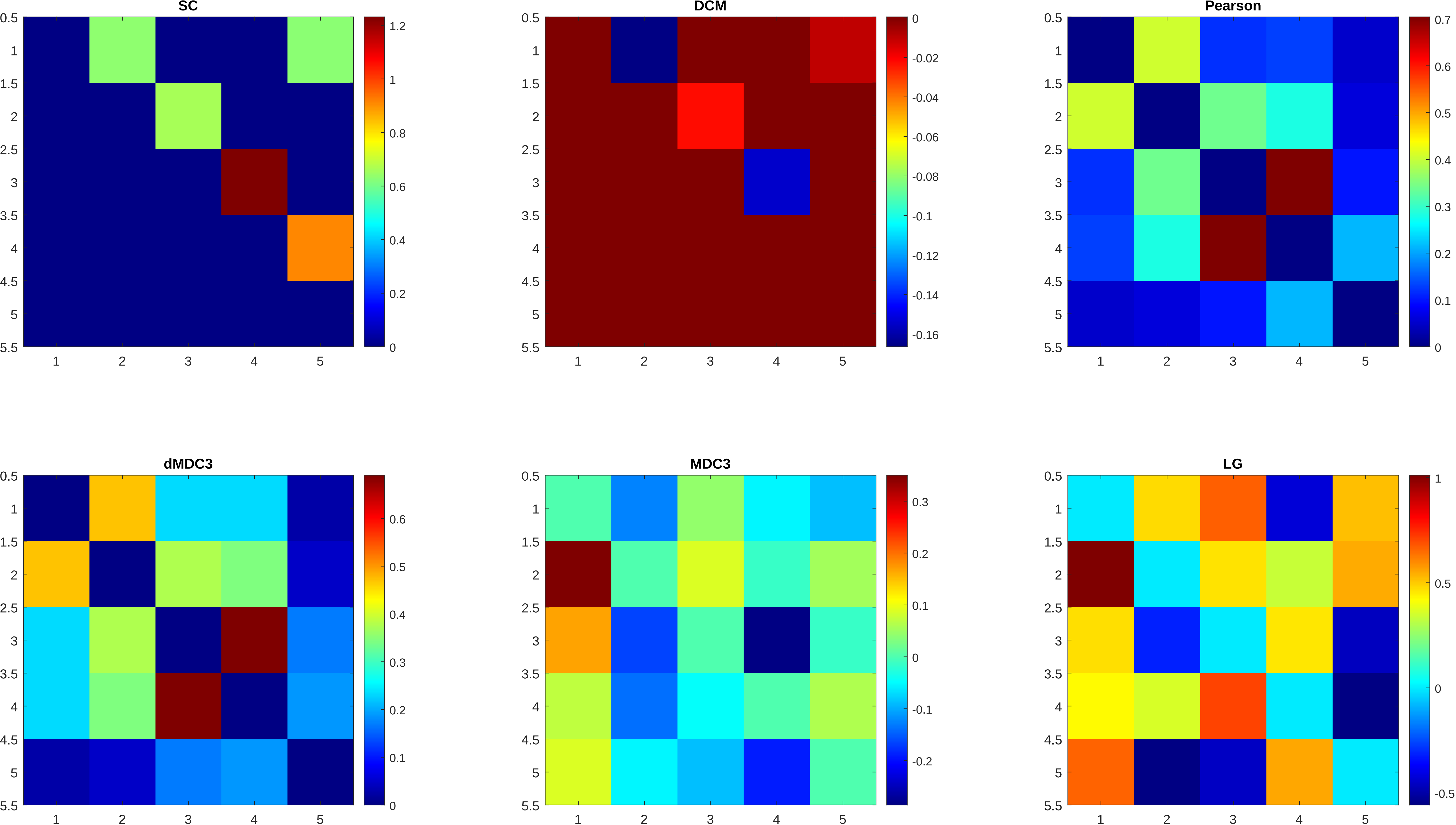
SC
0.5 1 1.5 2 2.5 3 3.5 4 4.5 5 5.5
1 2 3 4 5
0 0.2 0.4 0.6 0.8 1 1.2
DCM
0.5 1 1.5 2 2.5 3 3.5 4 4.5 5 5.5
1 2 3 4 5
0 -0.02 -0.04 -0.06 -0.08 -0.1 -0.12 -0.14 -0.16
Pearson
0.5 1 1.5 2 2.5 3 3.5 4 4.5 5 5.5
1 2 3 4 5
0 0.1 0.2 0.3 0.4 0.5 0.6 0.7
dMDC3
0.5 1 1.5 2 2.5 3 3.5 4 4.5 5 5.5
1 2 3 4 5
0 0.1 0.2 0.3 0.4 0.5 0.6
MDC3
0.5 1 1.5 2 2.5 3 3.5 4 4.5 5 5.5
1 2 3 4 5
0.3 0.2 0.1 0 -0.1 -0.2
LG
0.5 1 1.5 2 2.5 3 3.5 4 4.5 5 5.5
1 2 3 4 5
1 0.5 0 -0.5

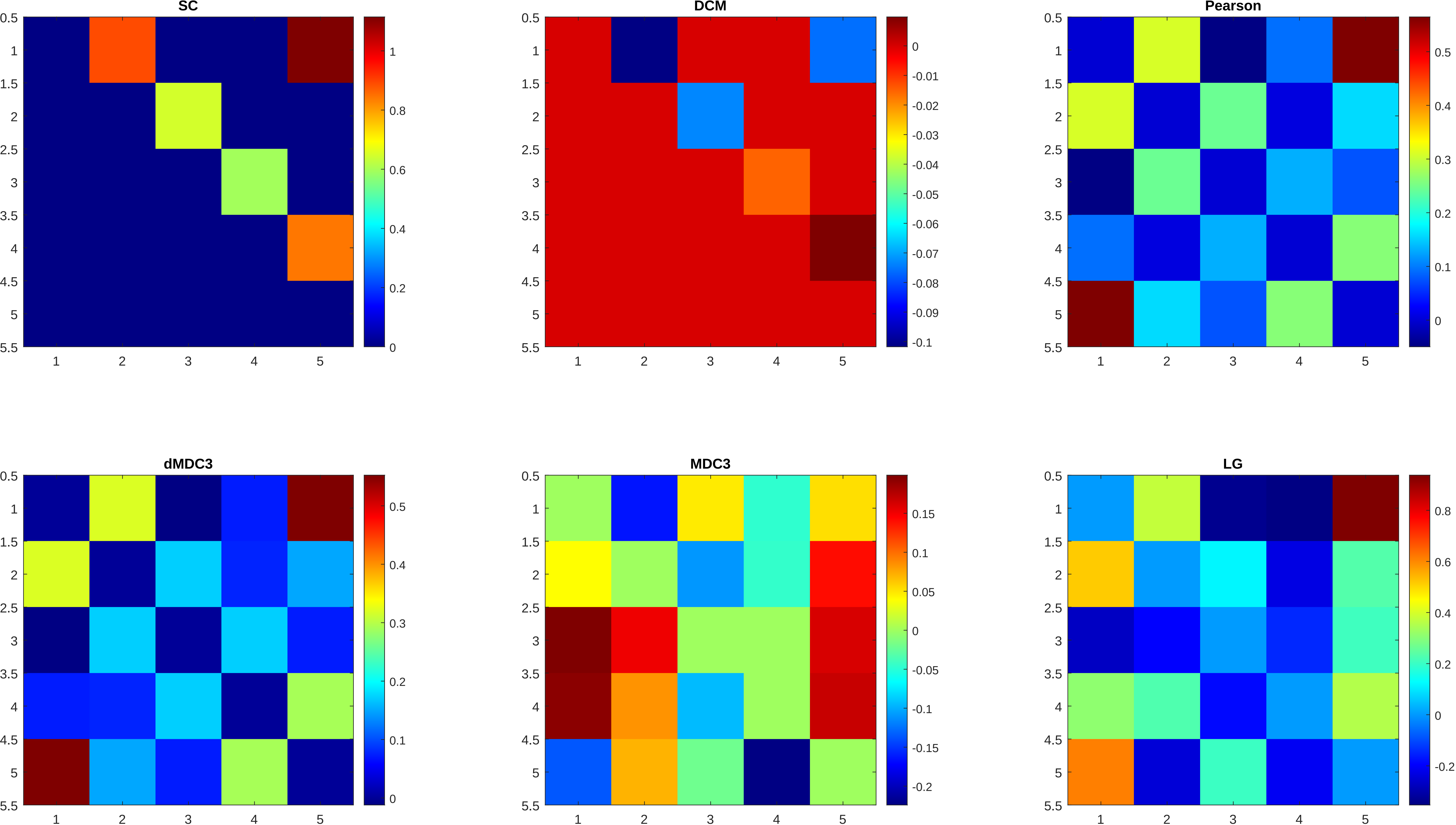
SC
DCM
Pearson
dMDC3
MDC3
LG

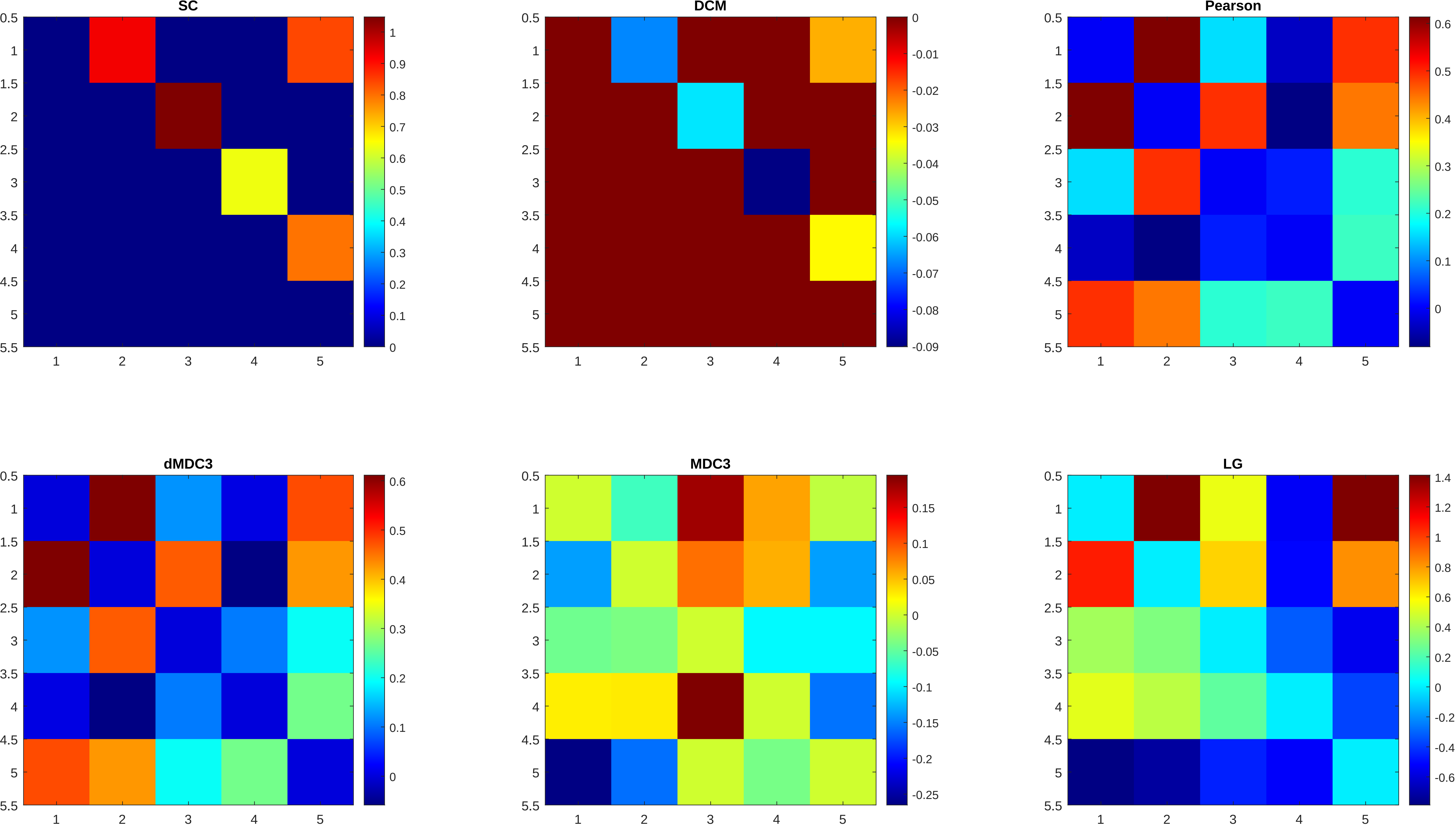
SC
DCM
Pearson
dMDC3
MDC3
LG
