# Supplementary material for: Multiscale detrended cross-correlation coefficient: estimating coupling in non-stationary neurophysiological signals
Source: Front Neurosci. 2024 Nov 13;18:1422085. doi: 10.3389/fnins.2024.1422085 (PMC11599215; doi:10.3389/fnins.2024.1422085)
Supplement: Supplementary file 1 [file Data_Sheet_1.zip › NetSim Matrices.zip!/NetSim Matrices/sim28.pdf]

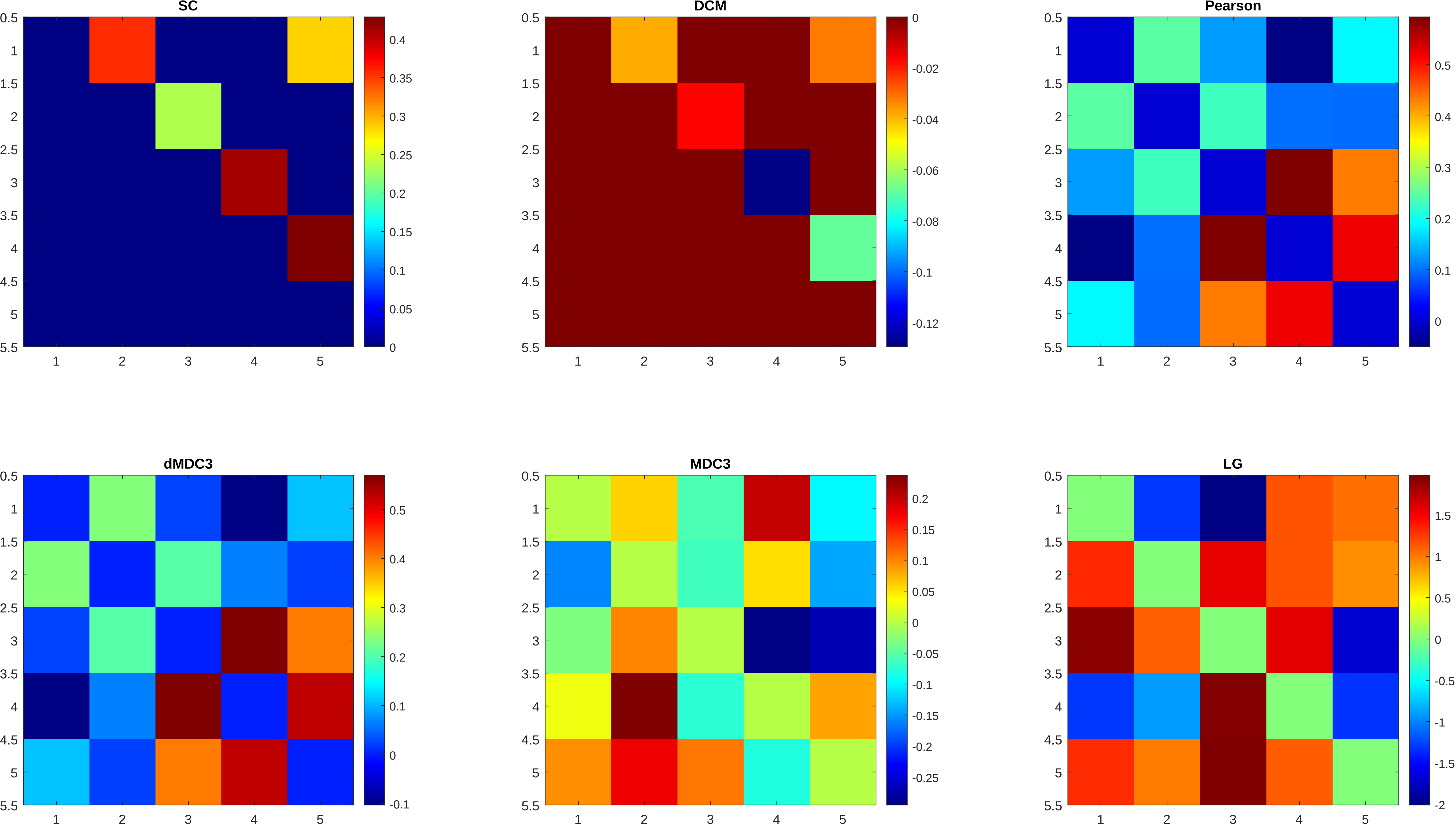
SC
DCM
Pearson
dMDC3
MDC3
LG

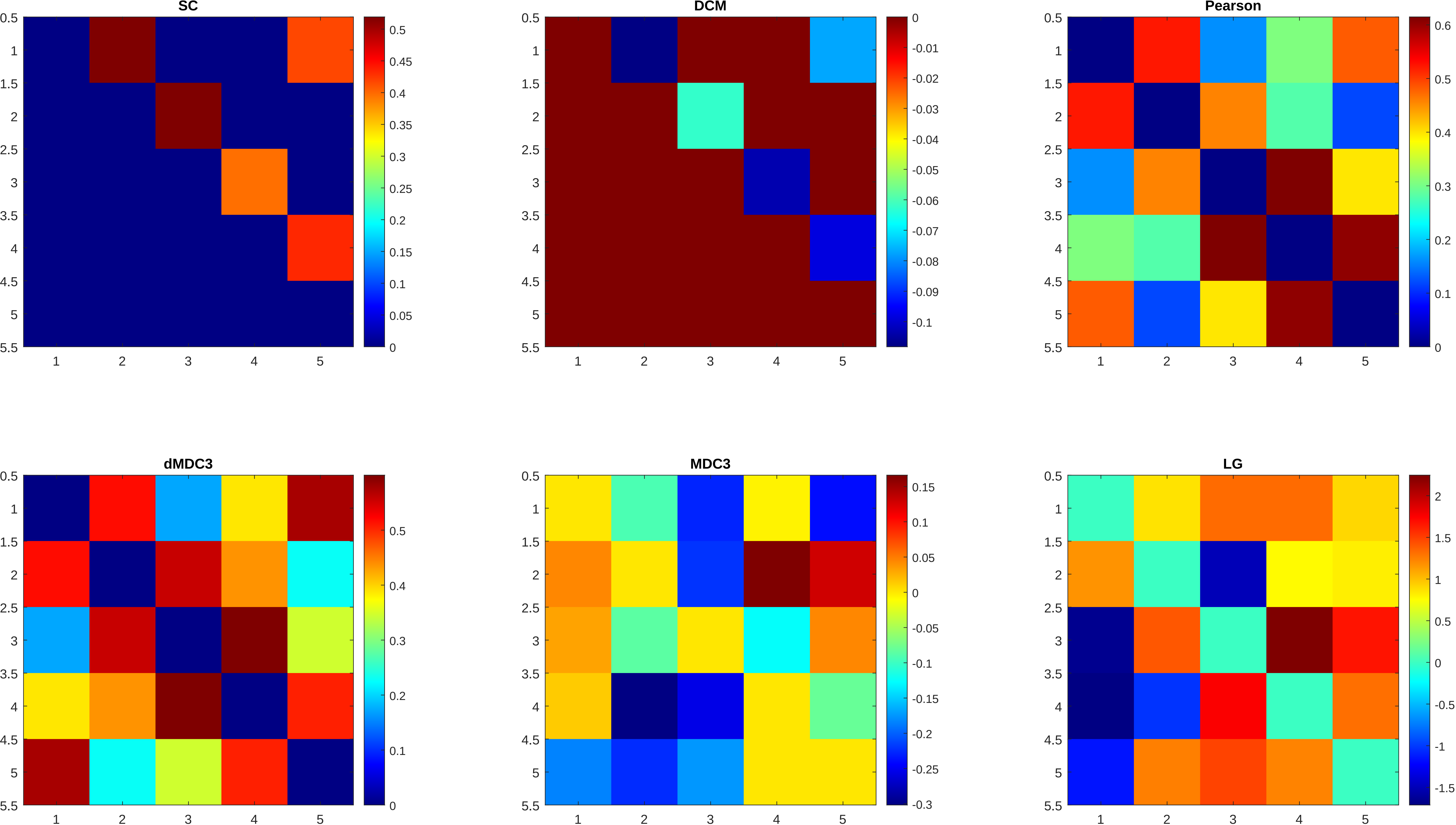

SC
DCM
Pearson
dMDC3
MDC3
LG

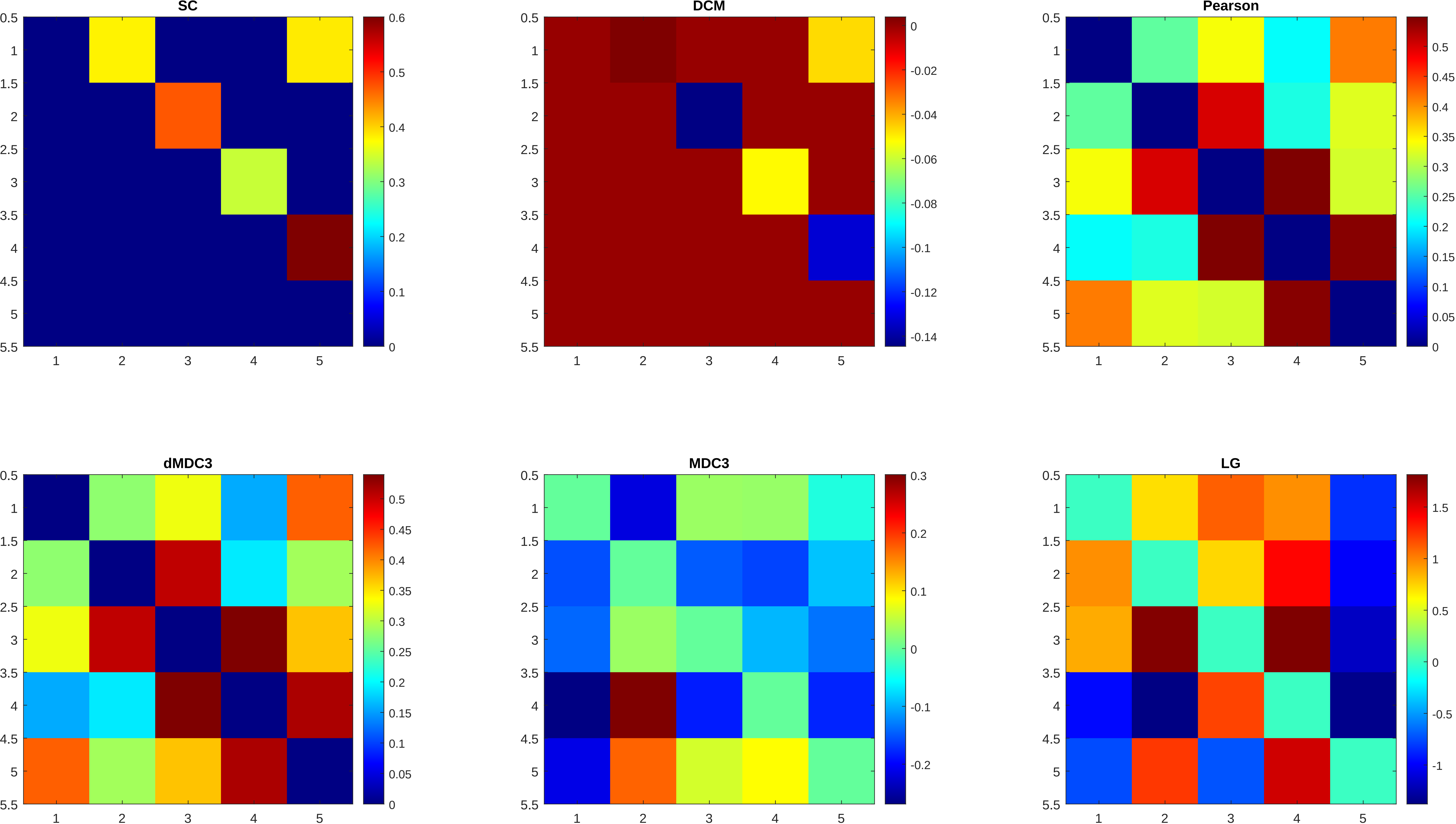
SC
DCM
Pearson
dMDC3
MDC3
LG
0.5
1
1.5
2
2.5
3
3.5
4
4.5
5
5.5
1
2
3
4
5

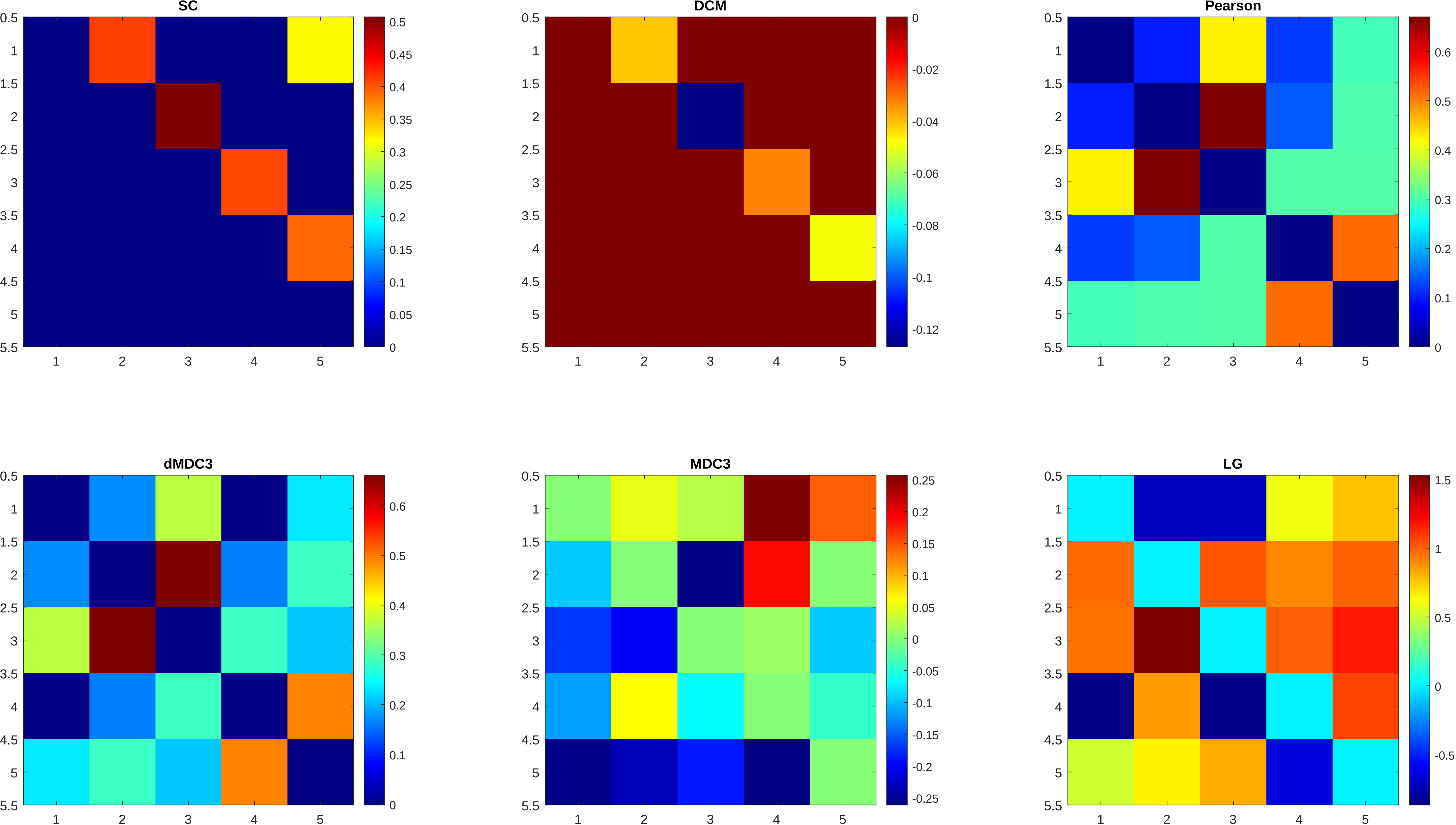

SC
DCM
Pearson
dMDC3
MDC3
LG

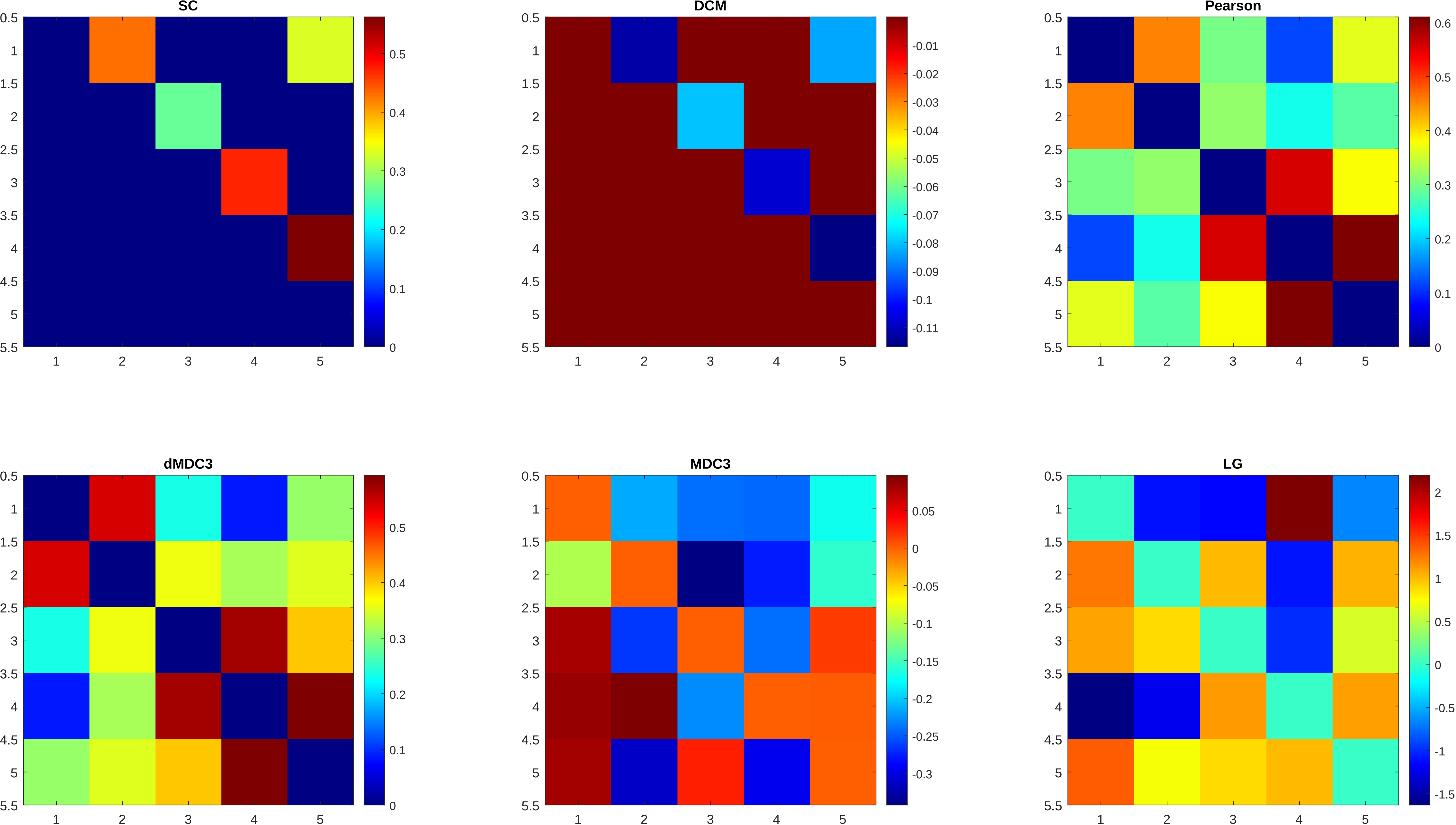
SC
DCM
Pearson
dMDC3
MDC3
LG

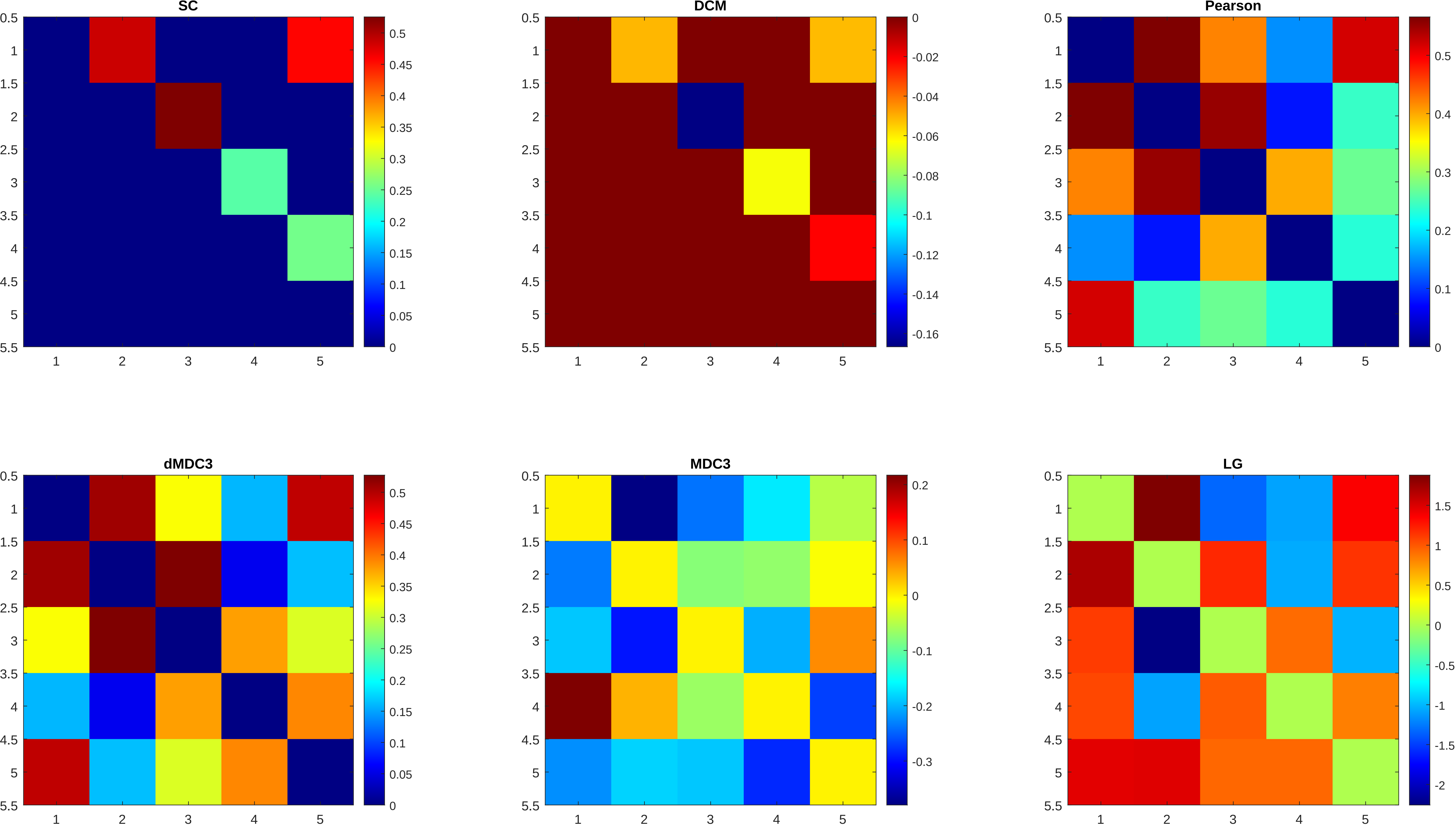
SC
DCM
Pearson
dMDC3
MDC3
LG

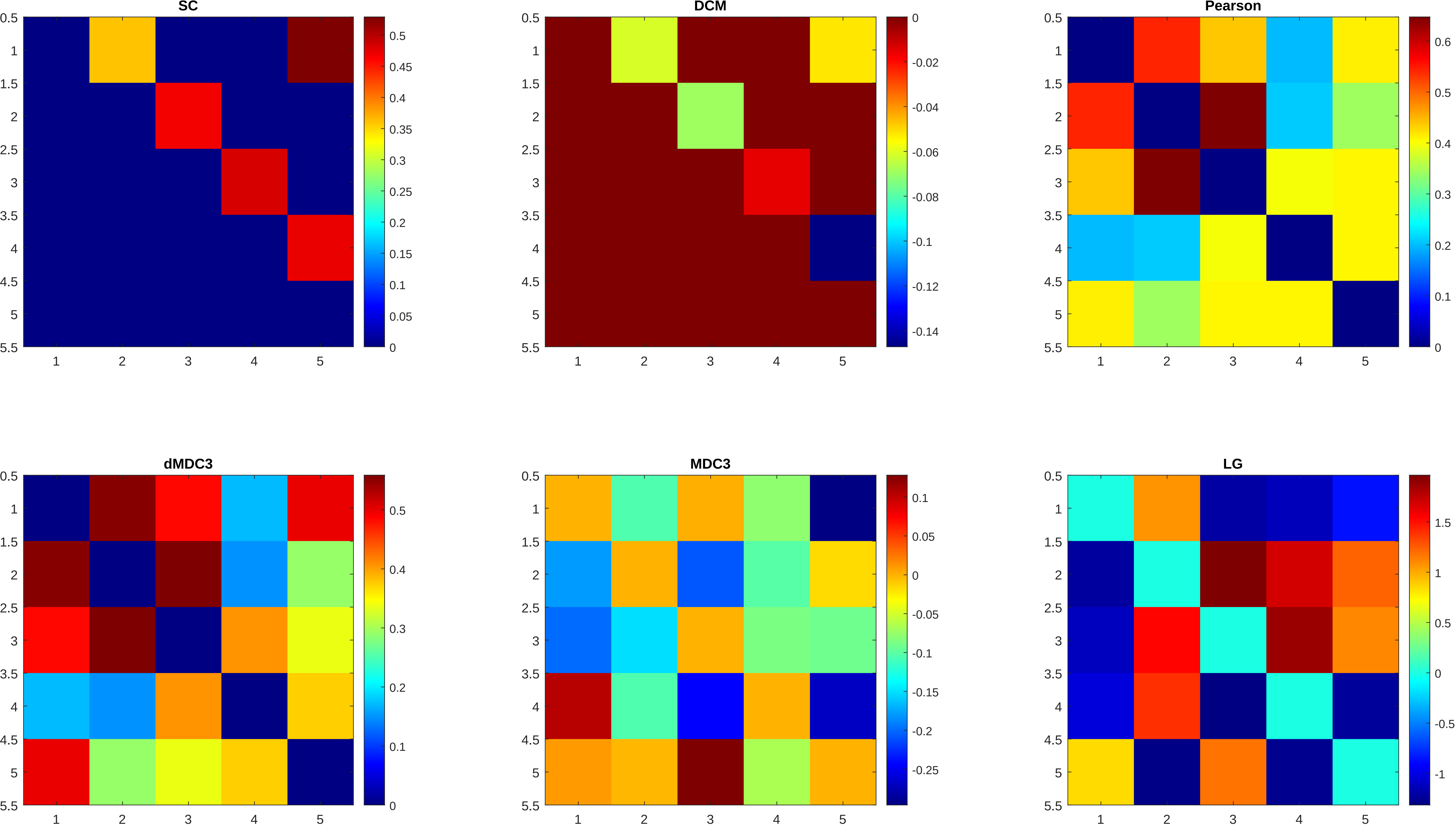
SC
DCM
Pearson
dMDC3
MDC3
LG

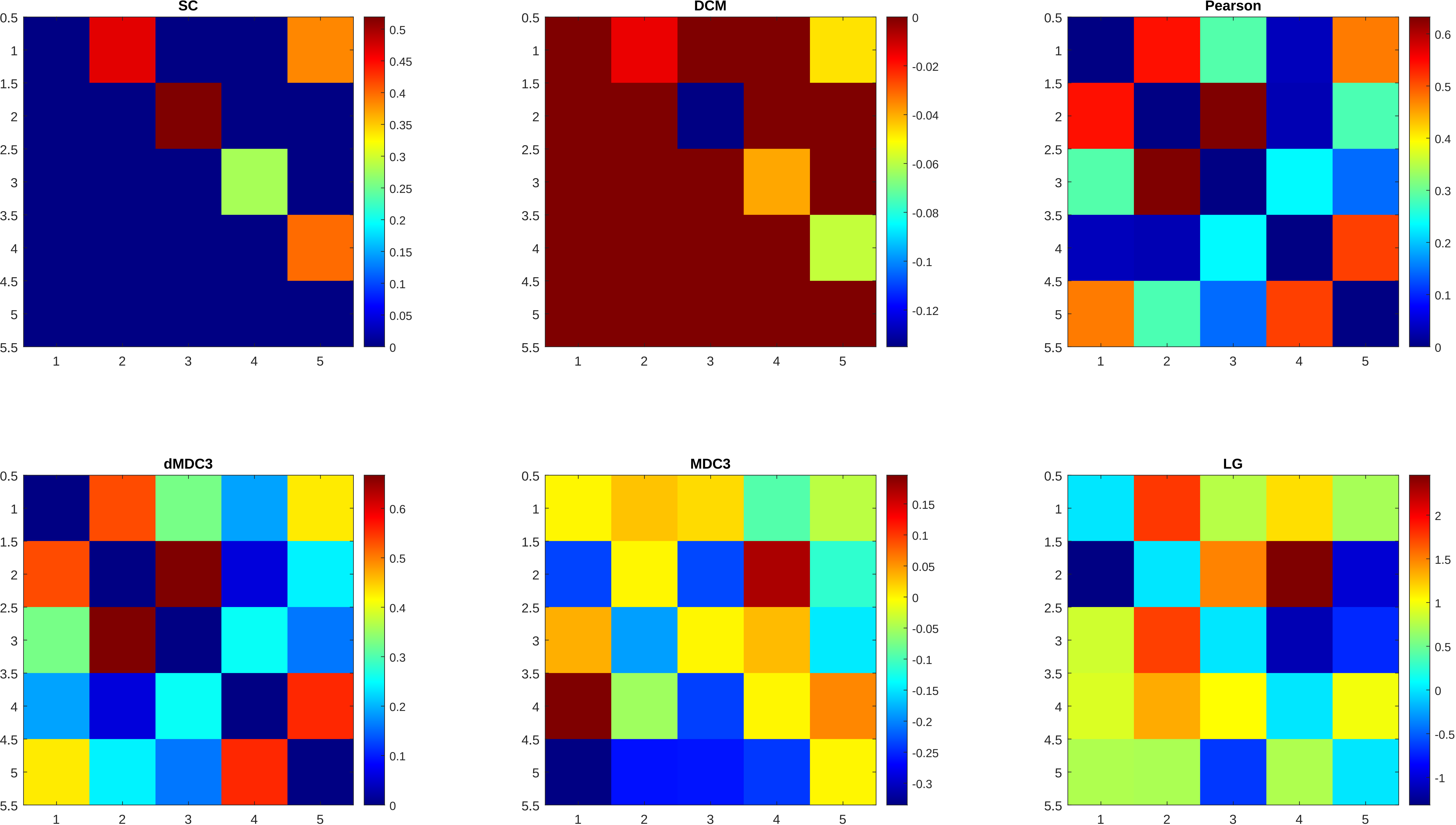
SC
DCM
Pearson
dMDC3
MDC3
LG
0.5 1 1.5 2 2.5 3 3.5 4 4.5 5 5.5
1 2 3 4 5
0 0.05 0.1 0.15 0.2 0.25 0.3 0.35 0.4 0.45 0.5
0 -0.02 -0.04 -0.06 -0.08 -0.1 -0.12
0 0.1 0.2 0.3 0.4 0.5 0.6
0 0.1 0.2 0.3 0.4 0.5 0.6
0.15 0.1 0.05 0 -0.05 -0.1 -0.15 -0.2 -0.25 -0.3
2 1.5 1 0.5 0 -0.5 -1

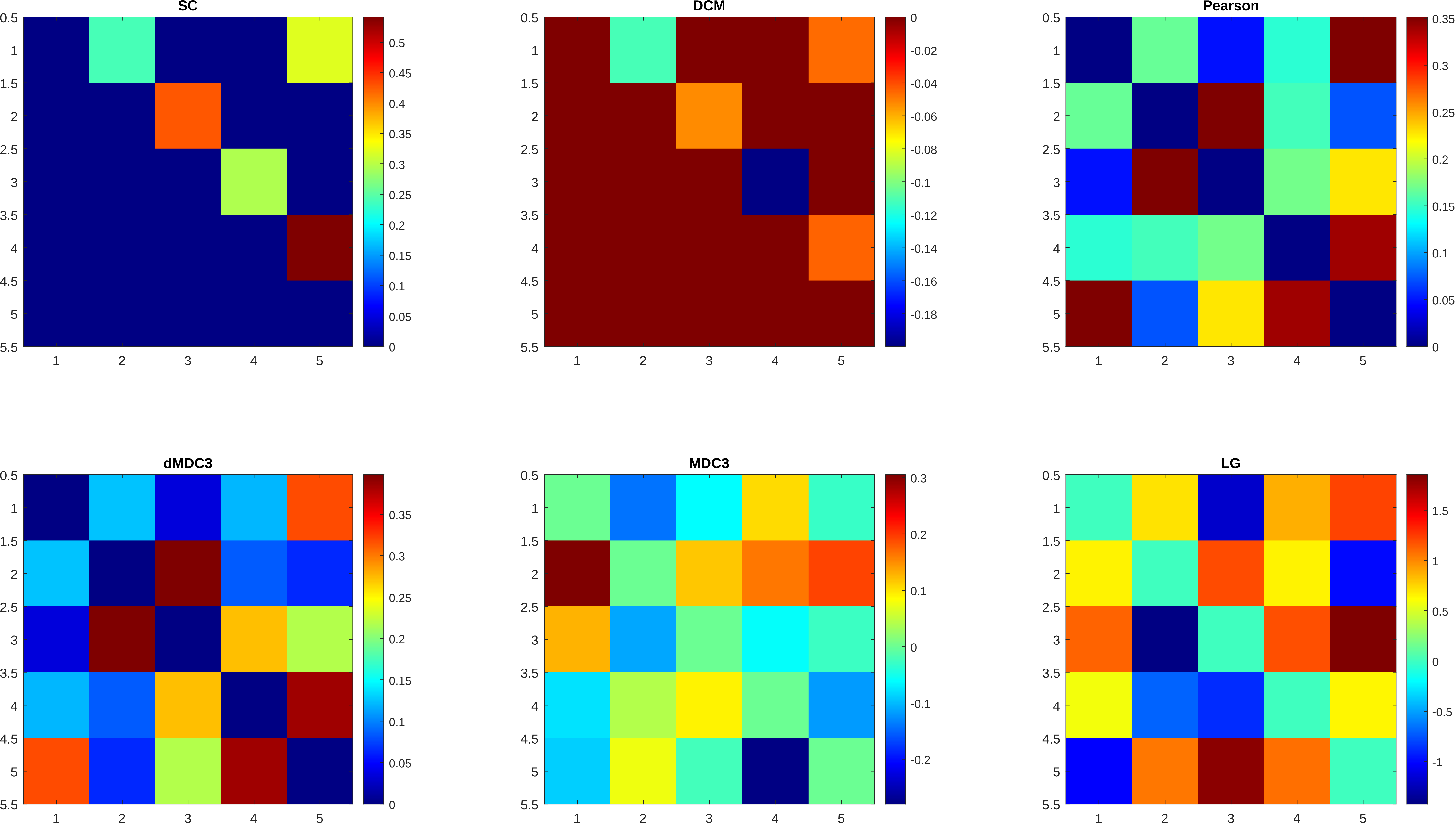
SC
0 0.05 0.1 0.15 0.2 0.25 0.3 0.35 0.4 0.45 0.5
DCM
0 -0.02 -0.04 -0.06 -0.08 -0.1 -0.12 -0.14 -0.16 -0.18
Pearson
0 0.05 0.1 0.15 0.2 0.25 0.3 0.35
dMDC3
0 0.05 0.1 0.15 0.2 0.25 0.3 0.35
MDC3
-0.2 -0.1 0 0.1 0.2 0.3
LG
-1 -0.5 0 0.5 1 1.5
0.5 1 1.5 2 2.5 3 3.5 4 4.5 5 5.5
1 2 3 4 5

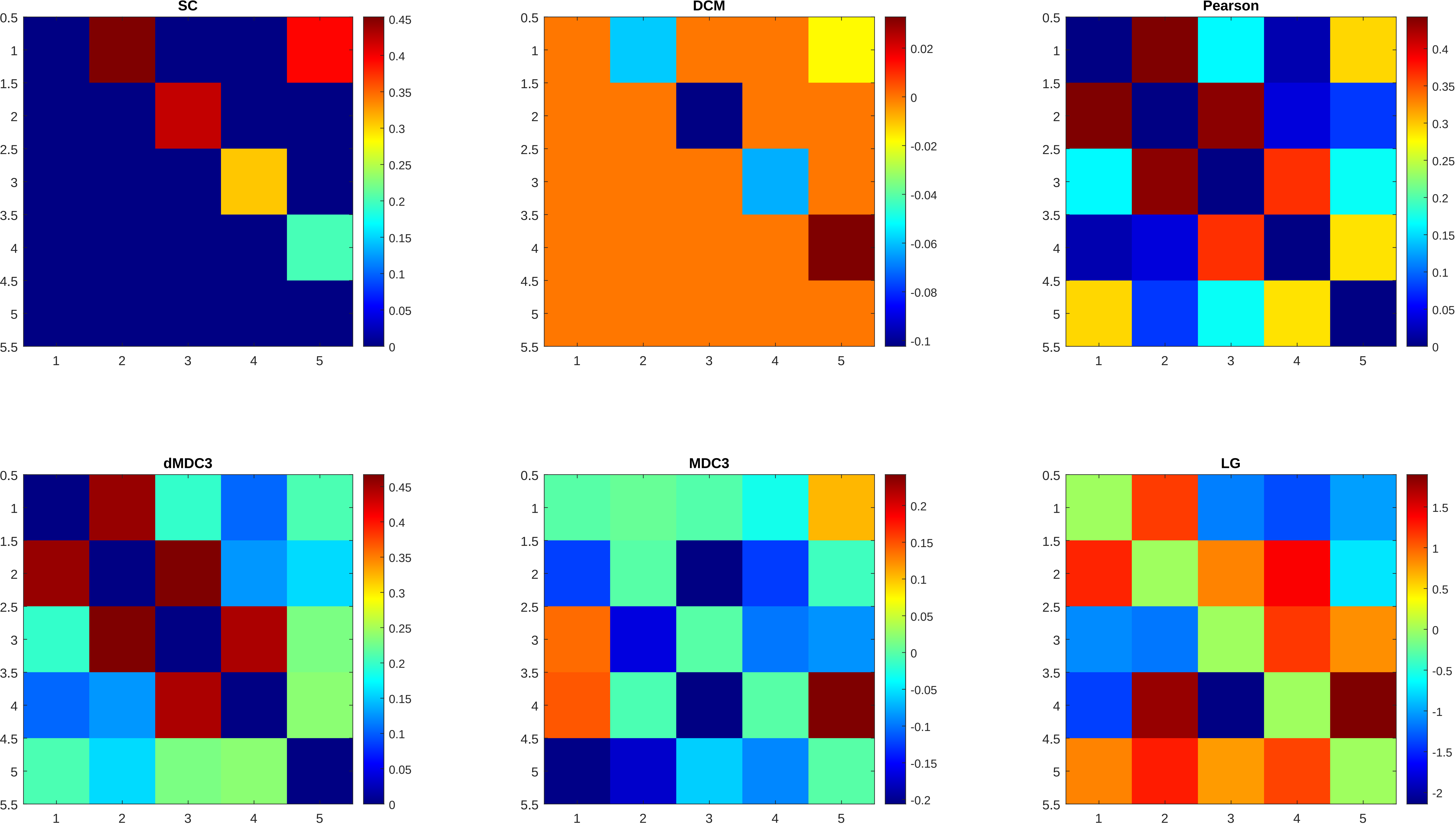
SC
0.5 1 1.5 2 2.5 3 3.5 4 4.5 5 5.5
1 2 3 4 5
0 0.05 0.1 0.15 0.2 0.25 0.3 0.35 0.4 0.45
DCM
0.5 1 1.5 2 2.5 3 3.5 4 4.5 5 5.5
1 2 3 4 5
-0.1 -0.08 -0.06 -0.04 -0.02 0 0.02
Pearson
0.5 1 1.5 2 2.5 3 3.5 4 4.5 5 5.5
1 2 3 4 5
0 0.05 0.1 0.15 0.2 0.25 0.3 0.35 0.4
dMDC3
0.5 1 1.5 2 2.5 3 3.5 4 4.5 5 5.5
1 2 3 4 5
0 0.05 0.1 0.15 0.2 0.25 0.3 0.35 0.4 0.45
MDC3
0.5 1 1.5 2 2.5 3 3.5 4 4.5 5 5.5
1 2 3 4 5
-0.2 -0.15 -0.1 -0.05 0 0.05 0.1 0.15 0.2
LG
0.5 1 1.5 2 2.5 3 3.5 4 4.5 5 5.5
1 2 3 4 5
-2 -1.5 -1 -0.5 0 0.5 1 1.5

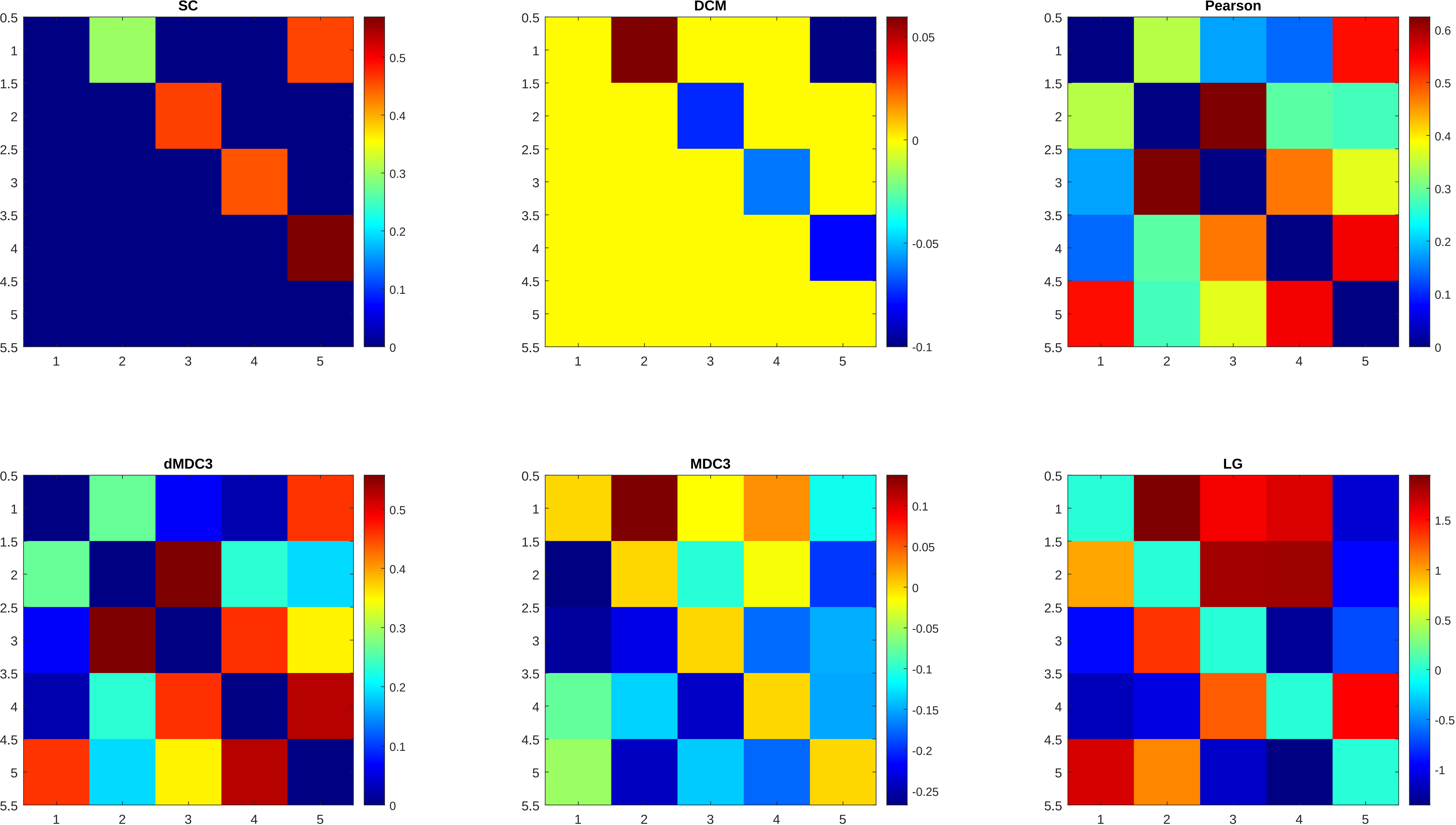
SC
DCM
Pearson
dMDC3
MDC3
LG

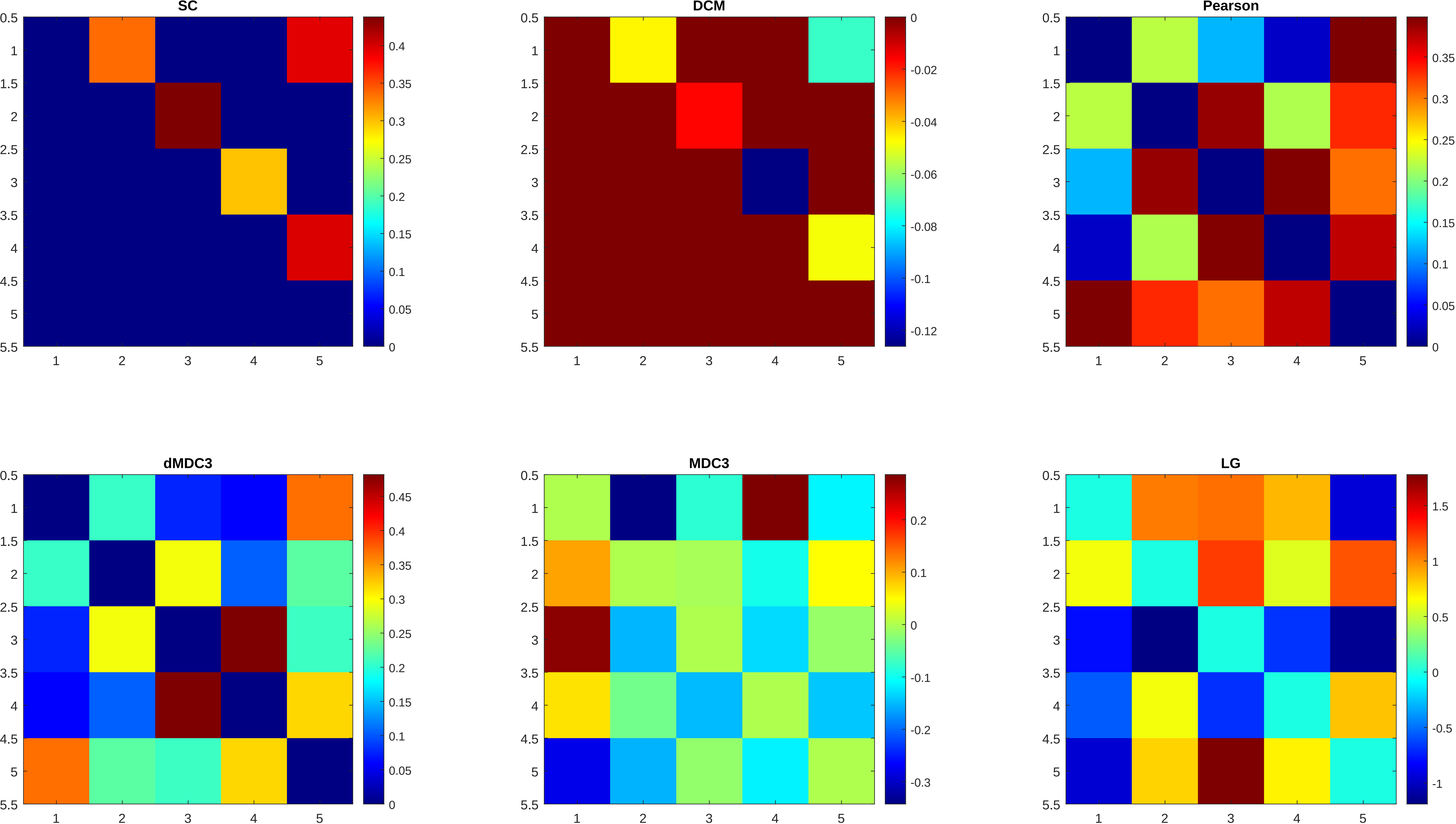
SC
DCM
Pearson
dMDC3
MDC3
LG
0.5
1
1.5
2
2.5
3
3.5
4
4.5
5
5.5
1
2
3
4
5
0
0.05
0.1
0.15
0.2
0.25
0.3
0.35
0.4
0.45
-0.02
-0.04
-0.06
-0.08
-0.1
-0.12
-0.1
-0.2
-0.3
-0.5
-1
0.5
1.5

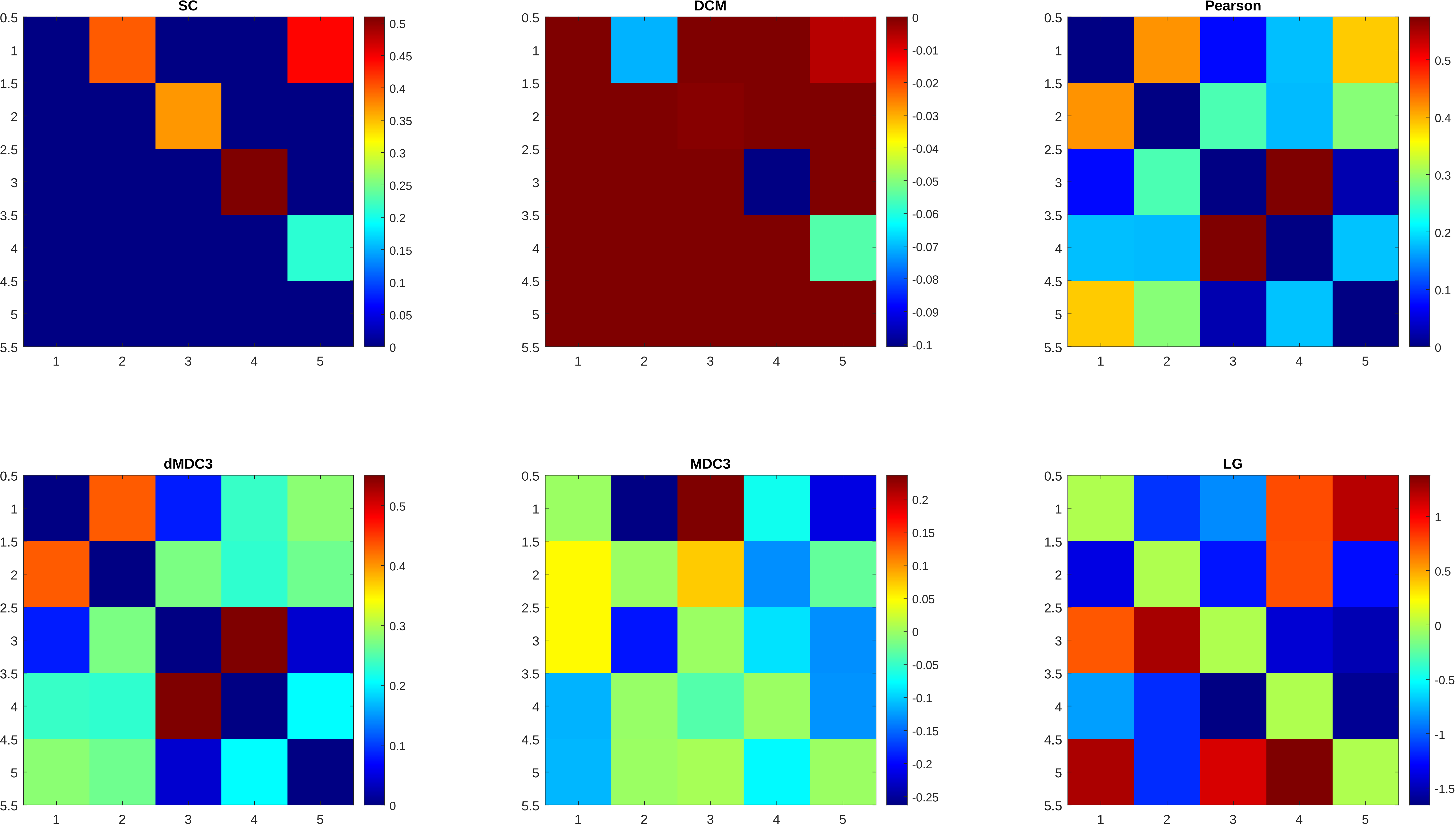
SC
DCM
Pearson
dMDC3
MDC3
LG
0.5
1
1.5
2
2.5
3
3.5
4
4.5
5
5.5
1
2
3
4
5

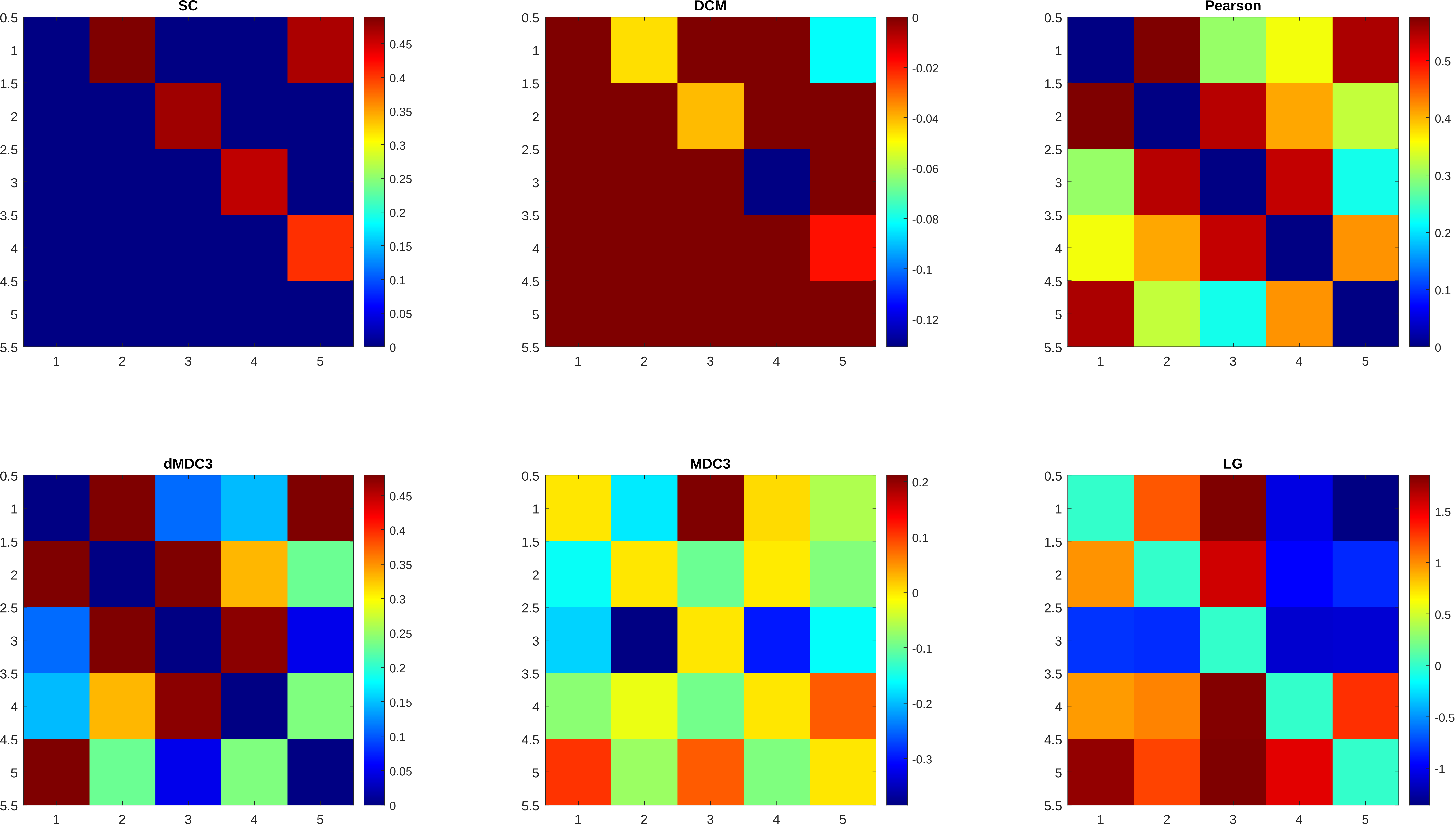
SC
DCM
Pearson
dMDC3
MDC3
LG
0.5
1
1.5
2
2.5
3
3.5
4
4.5
5
5.5
0.45
0.4
0.35
0.3
0.25
0.2
0.15
0.1
0.05
0
-0.02
-0.04
-0.06
-0.08
-0.1
-0.12
0.2
0.1
-0.1
-0.2
-0.3
-0.5
-1

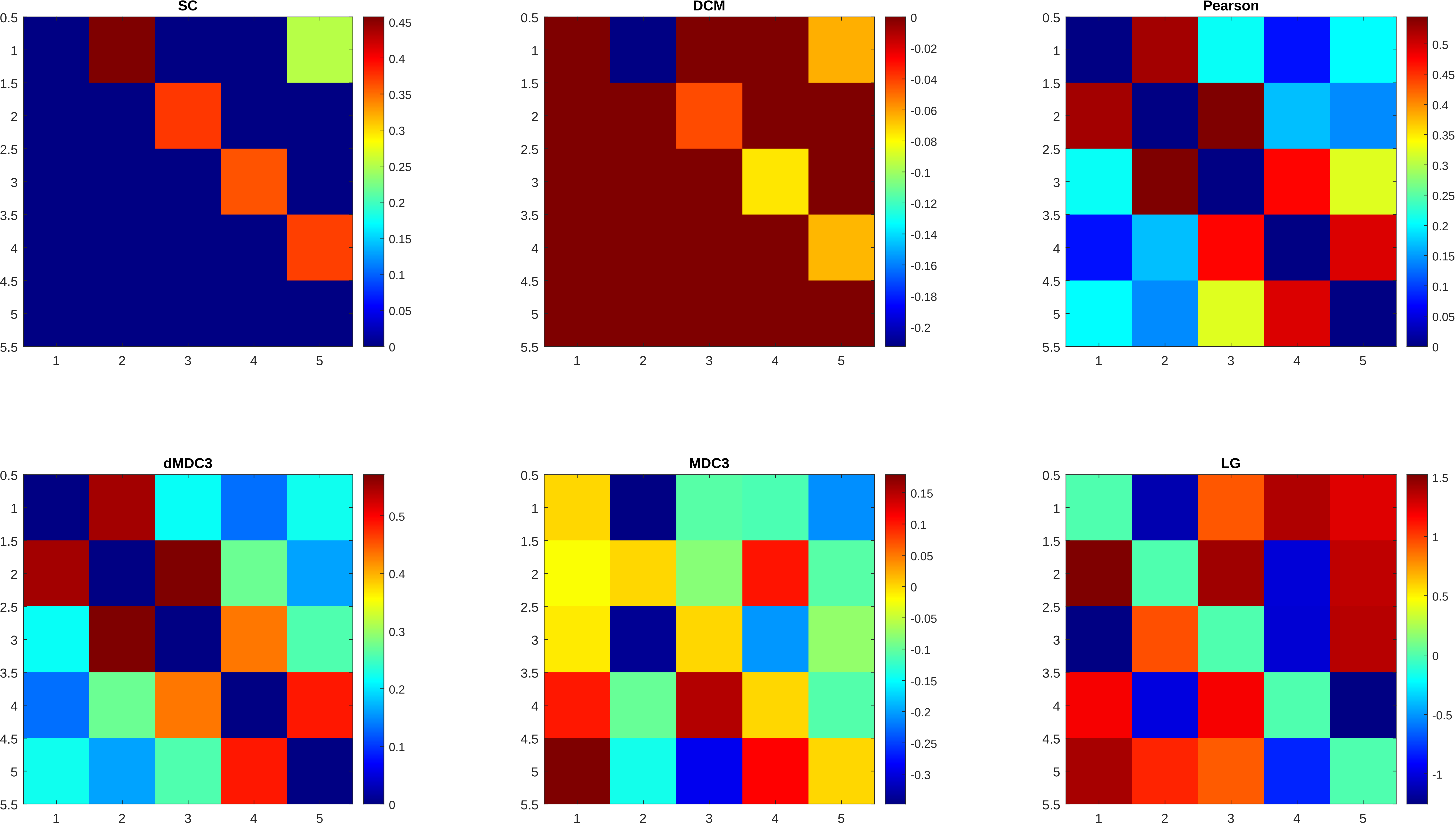
SC
DCM
Pearson
dMDC3
MDC3
LG

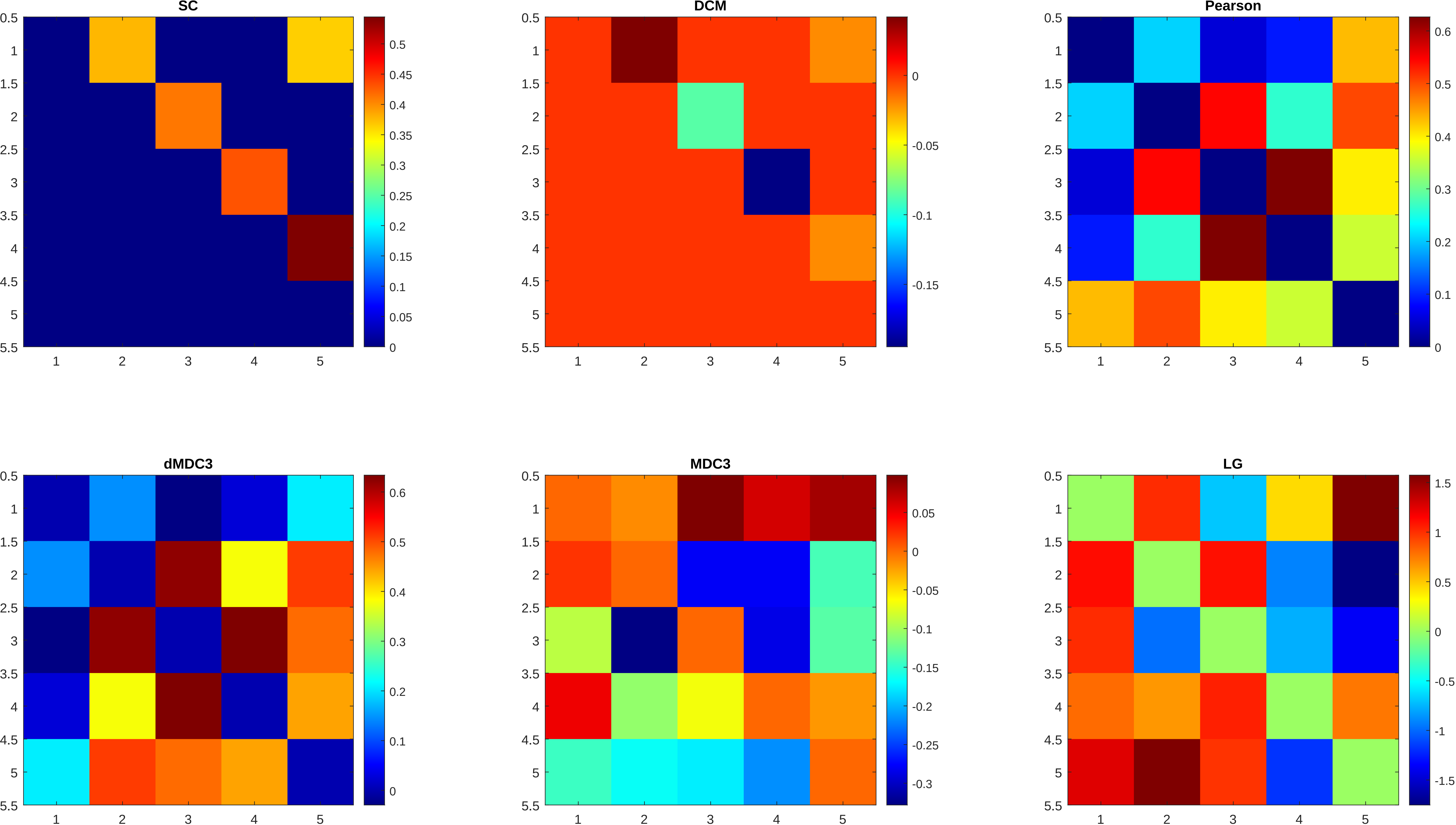
SC
DCM
Pearson
dMDC3
MDC3
LG

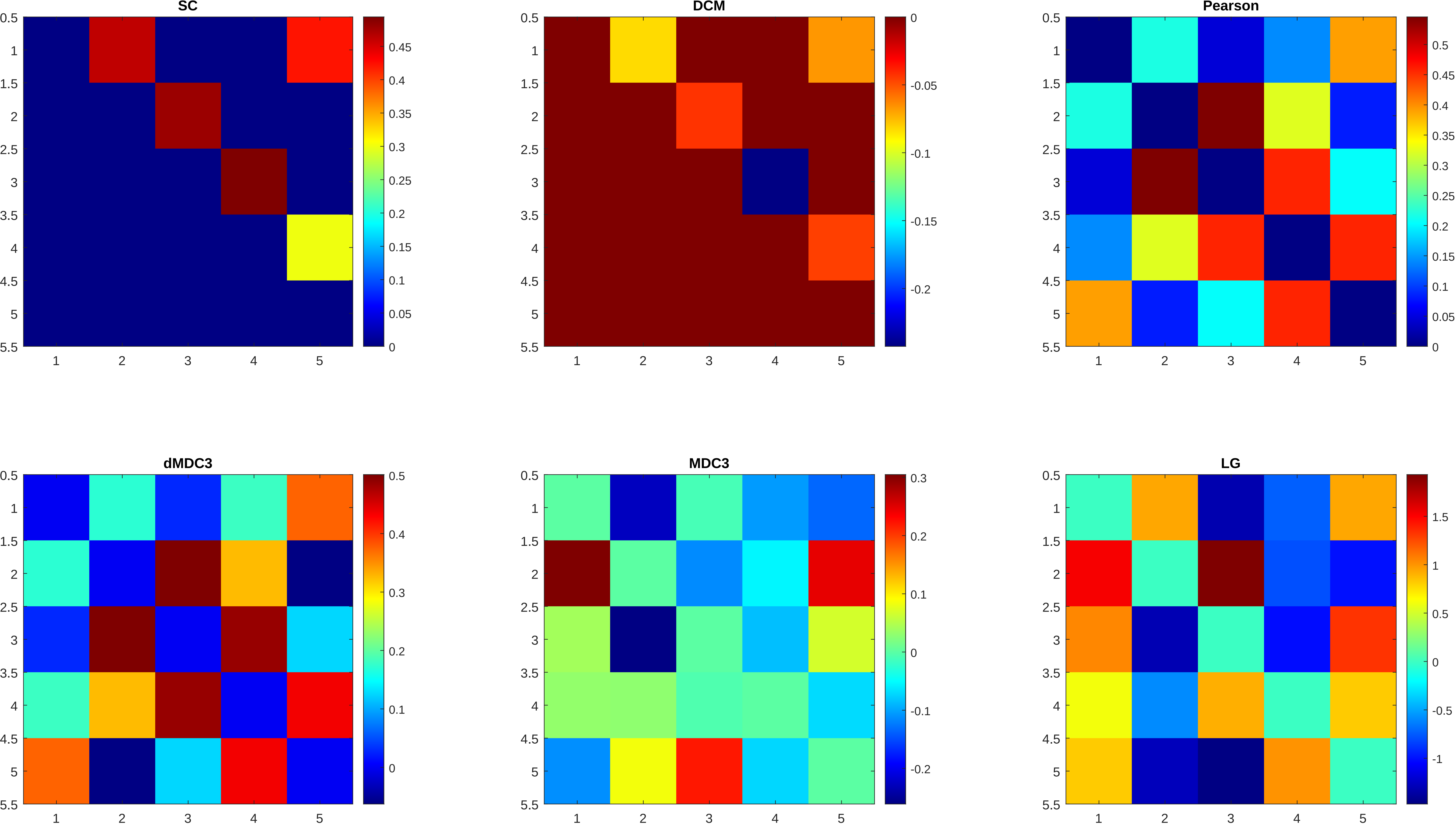
SC
0.5 1 1.5 2 2.5 3 3.5 4 4.5 5 5.5
1 2 3 4 5
0 0.05 0.1 0.15 0.2 0.25 0.3 0.35 0.4 0.45
DCM
0.5 1 1.5 2 2.5 3 3.5 4 4.5 5 5.5
1 2 3 4 5
0 -0.05 -0.1 -0.15 -0.2
Pearson
0.5 1 1.5 2 2.5 3 3.5 4 4.5 5 5.5
1 2 3 4 5
0 0.05 0.1 0.15 0.2 0.25 0.3 0.35 0.4 0.45 0.5
dMDC3
0.5 1 1.5 2 2.5 3 3.5 4 4.5 5 5.5
1 2 3 4 5
0 0.1 0.2 0.3 0.4 0.5
MDC3
0.5 1 1.5 2 2.5 3 3.5 4 4.5 5 5.5
1 2 3 4 5
0.3 0.2 0.1 0 -0.1 -0.2
LG
0.5 1 1.5 2 2.5 3 3.5 4 4.5 5 5.5
1 2 3 4 5
1.5 1 0.5 0 -0.5 -1

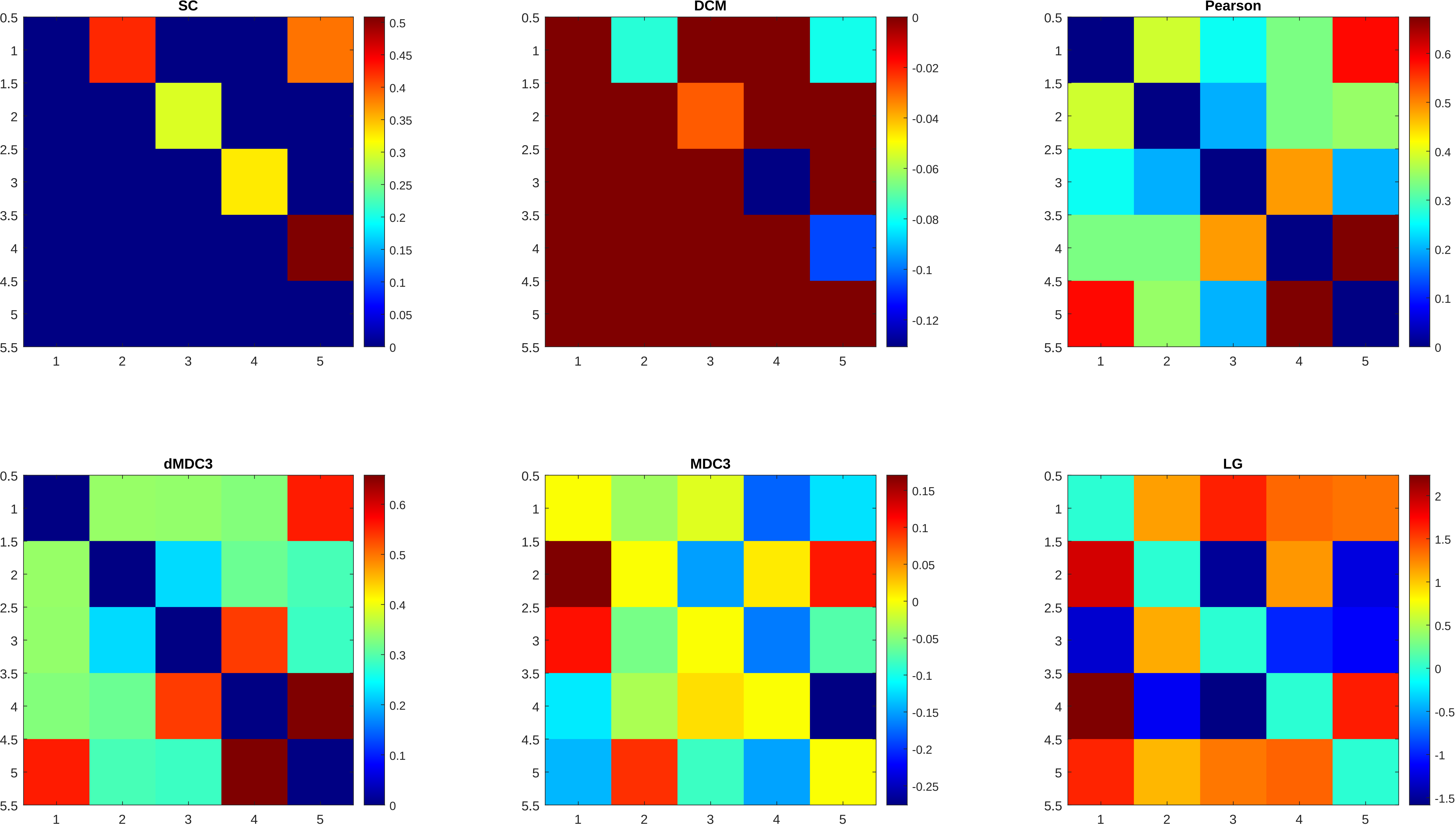
SC
DCM
Pearson
dMDC3
MDC3
LG
0.5
1
1.5
2
2.5
3
3.5
4
4.5
5
5.5
1
2
3
4
5

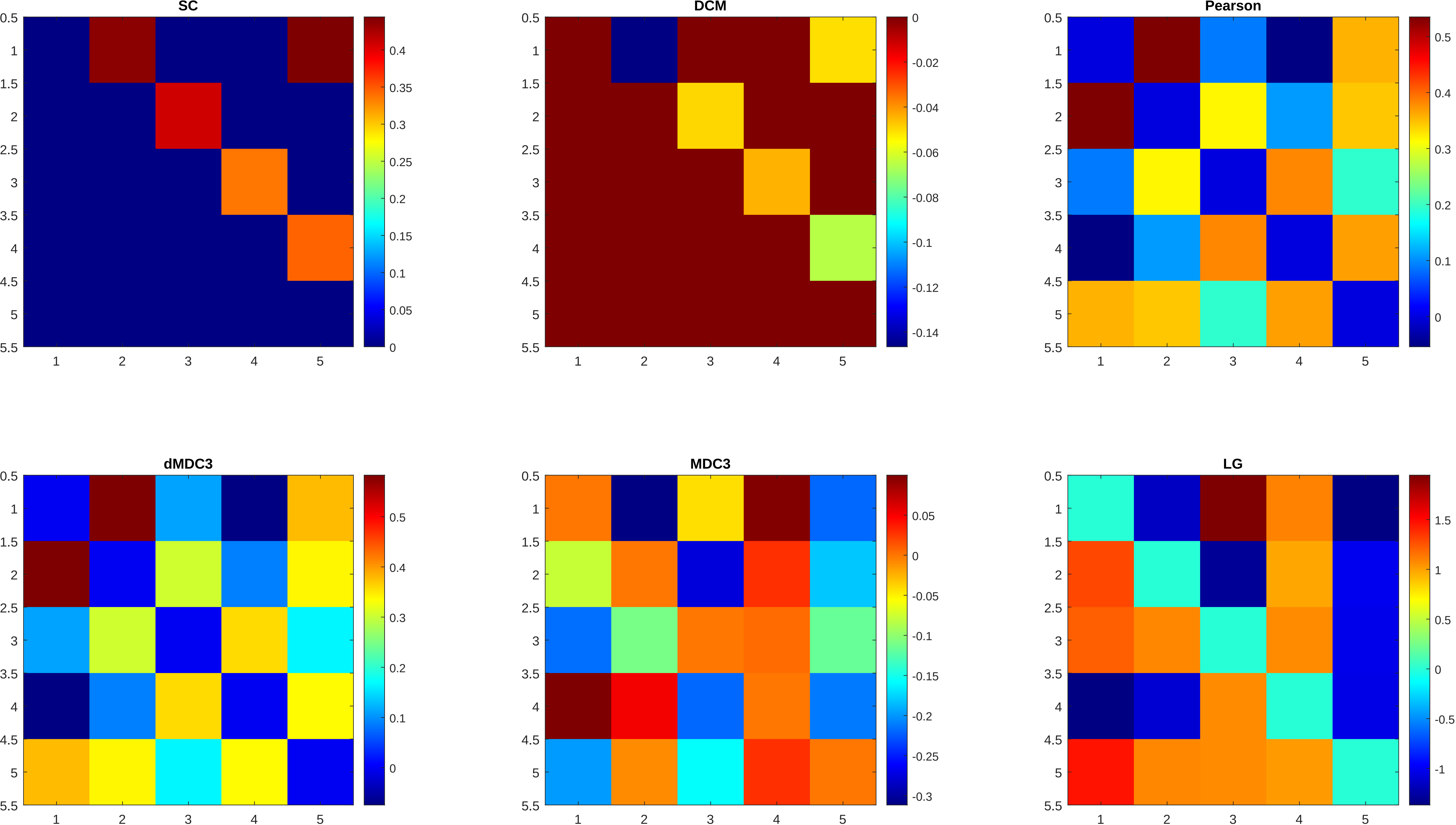
SC
DCM
Pearson
dMDC3
MDC3
LG

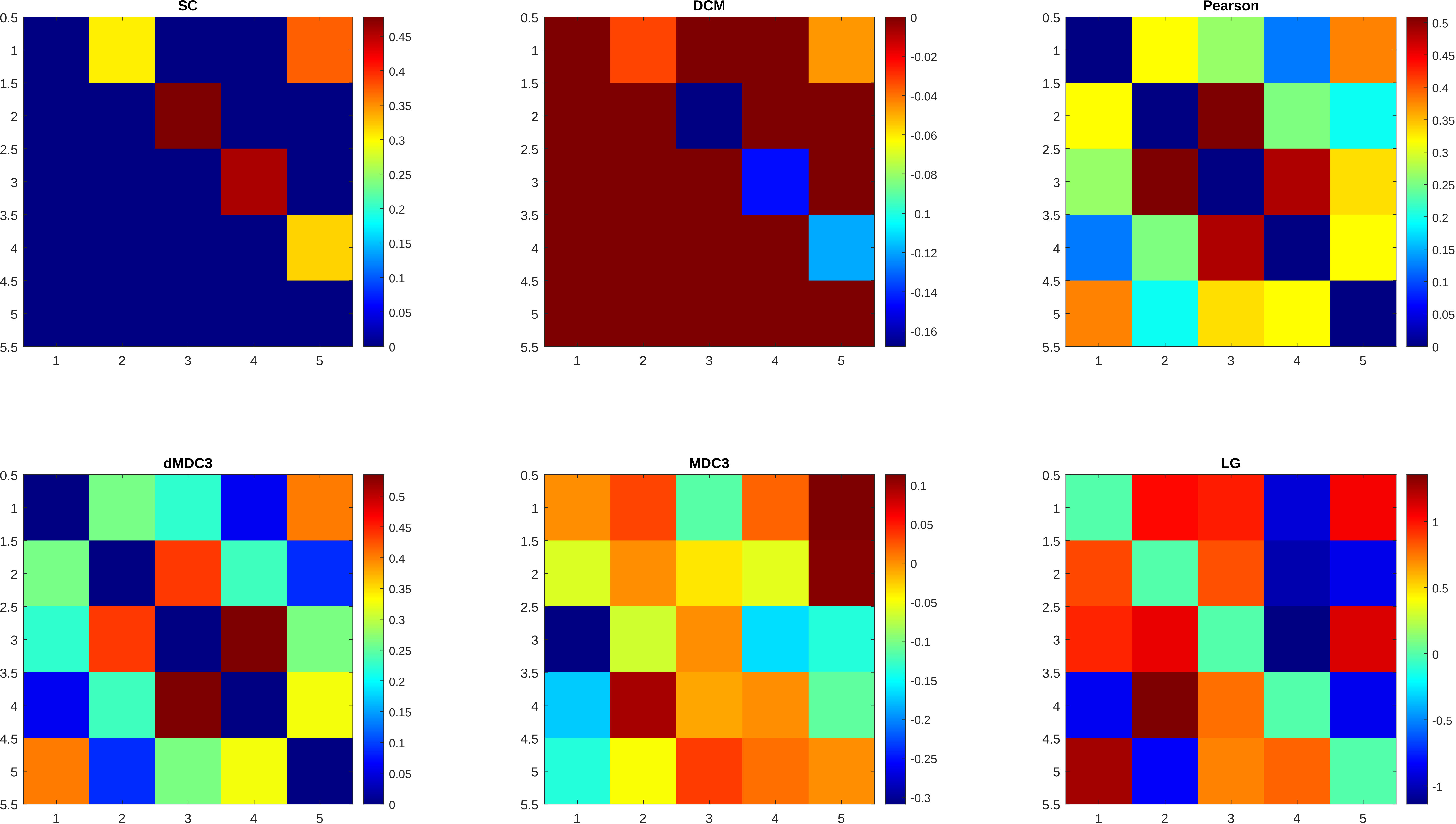
SC
DCM
Pearson
dMDC3
MDC3
LG
0.5
1
1.5
2
2.5
3
3.5
4
4.5
5
5.5
0
0.05
0.1
0.15
0.2
0.25
0.3
0.35
0.4
0.45
0.5
-0.02
-0.04
-0.06
-0.08
-0.1
-0.12
-0.14
-0.16
-0.05
-0.15
-0.2
-0.25
-0.3
-0.5
-1

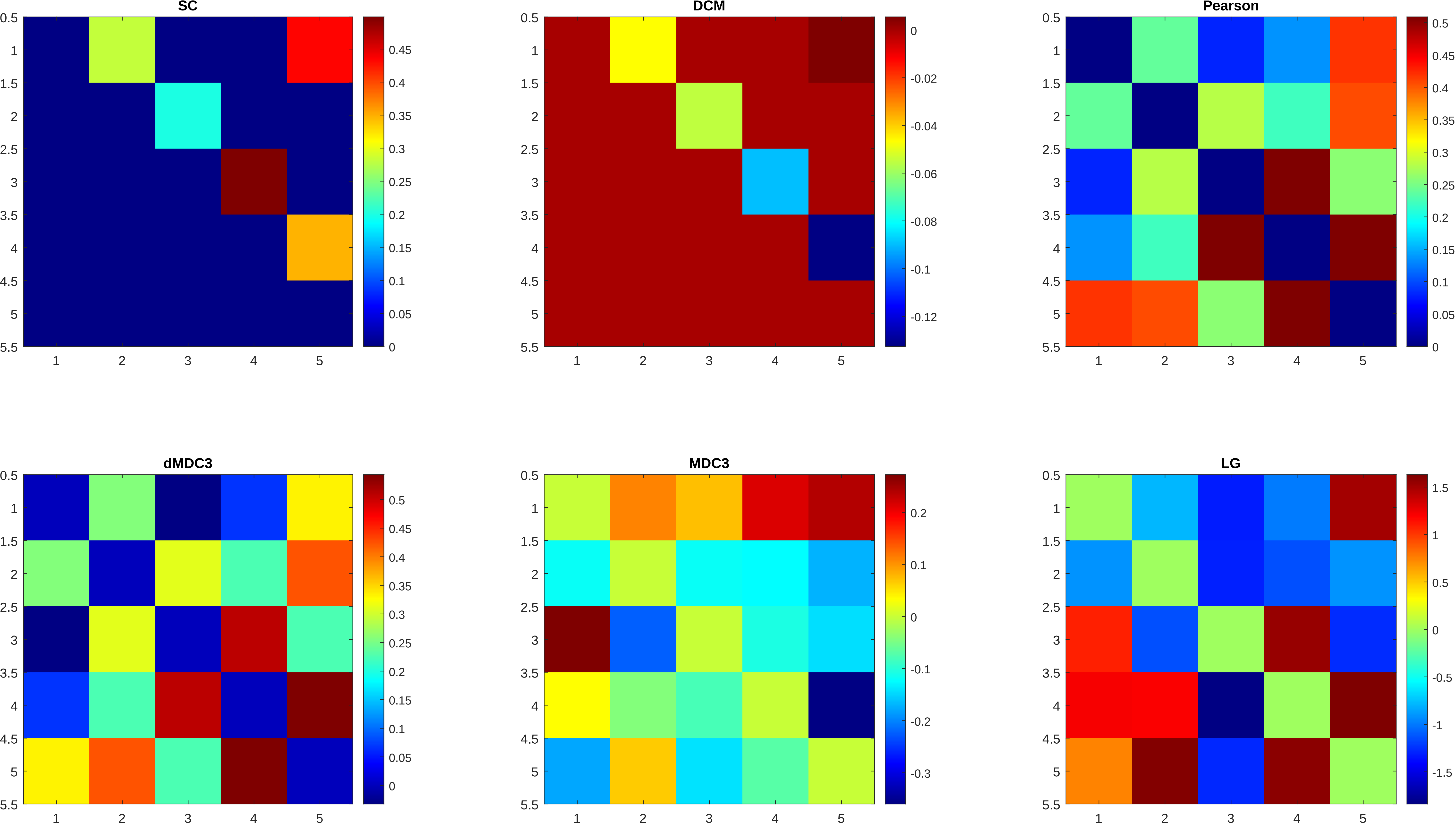
SC
DCM
Pearson
dMDC3
MDC3
LG

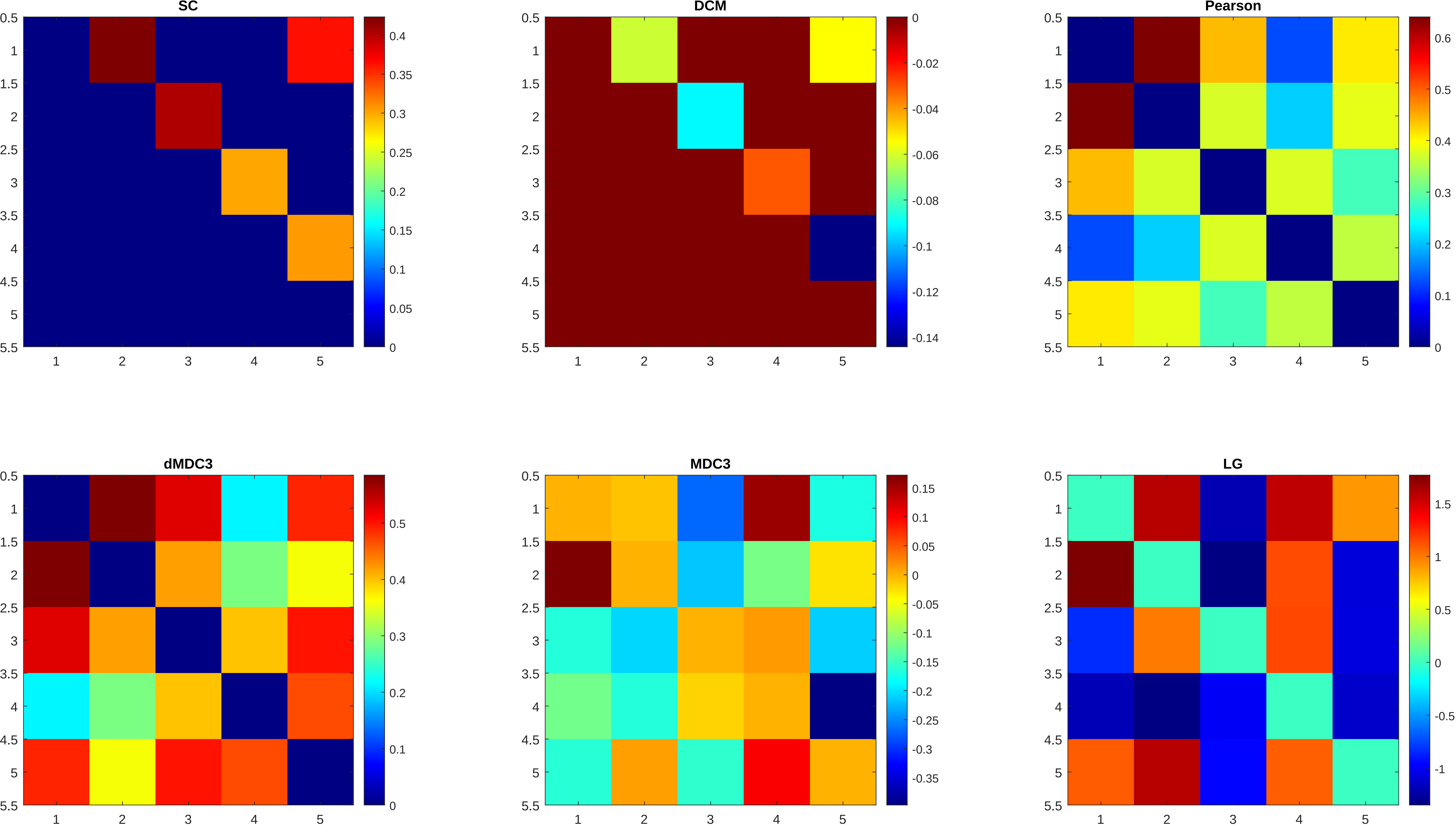

SC
DCM
Pearson
dMDC3
MDC3
LG

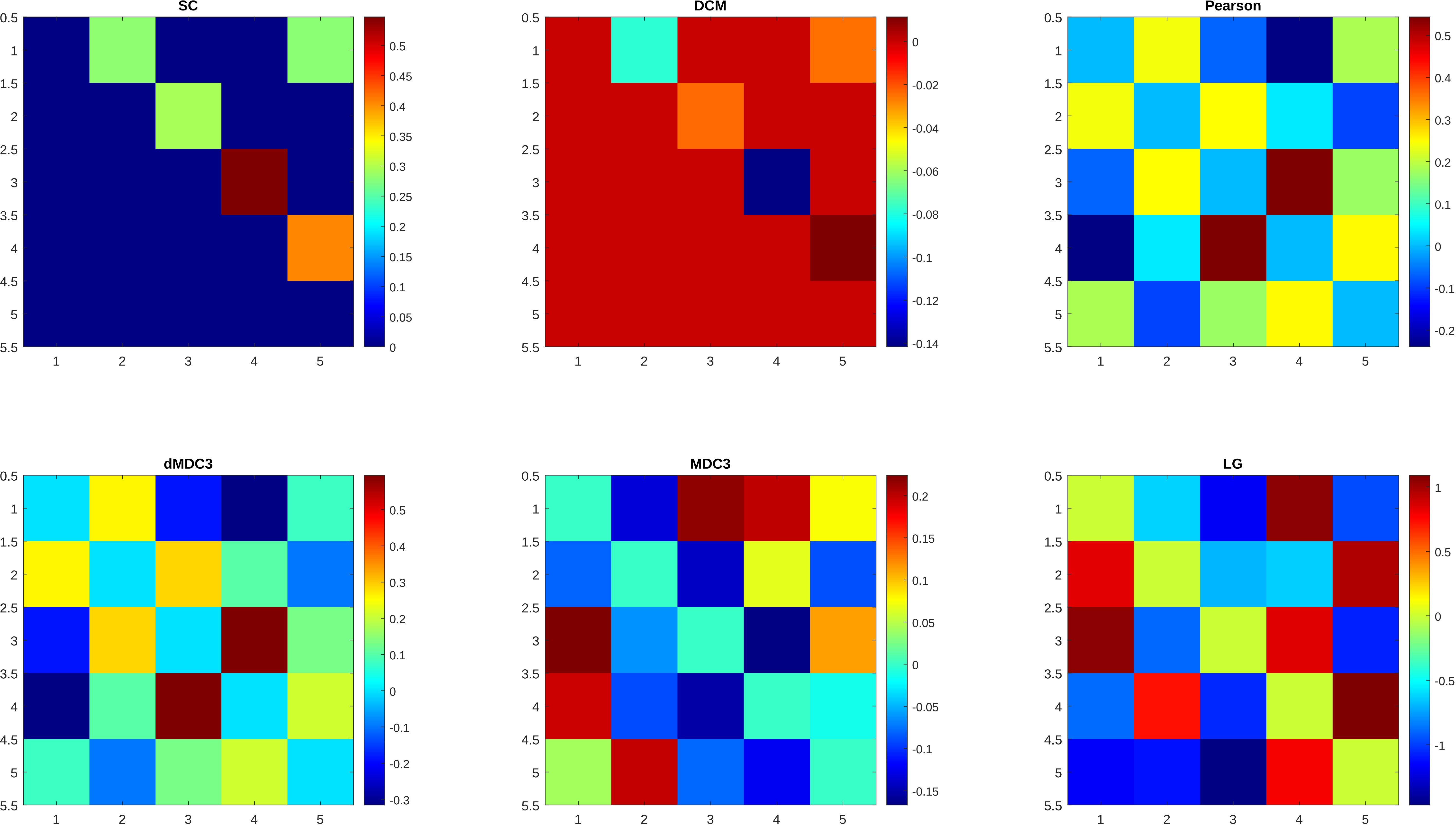
SC
DCM
Pearson
dMDC3
MDC3
LG
0.5 1 1.5 2 2.5 3 3.5 4 4.5 5 5.5
1 2 3 4 5
0 0.05 0.1 0.15 0.2 0.25 0.3 0.35 0.4 0.45 0.5
-0.14 -0.12 -0.1 -0.08 -0.06 -0.04 -0.02 0
-0.2 -0.1 0 0.1 0.2 0.3 0.4 0.5
-0.3 -0.2 -0.1 0 0.1 0.2 0.3 0.4 0.5
-0.15 -0.1 -0.05 0 0.05 0.1 0.15 0.2
-1 -0.5 0 0.5 1

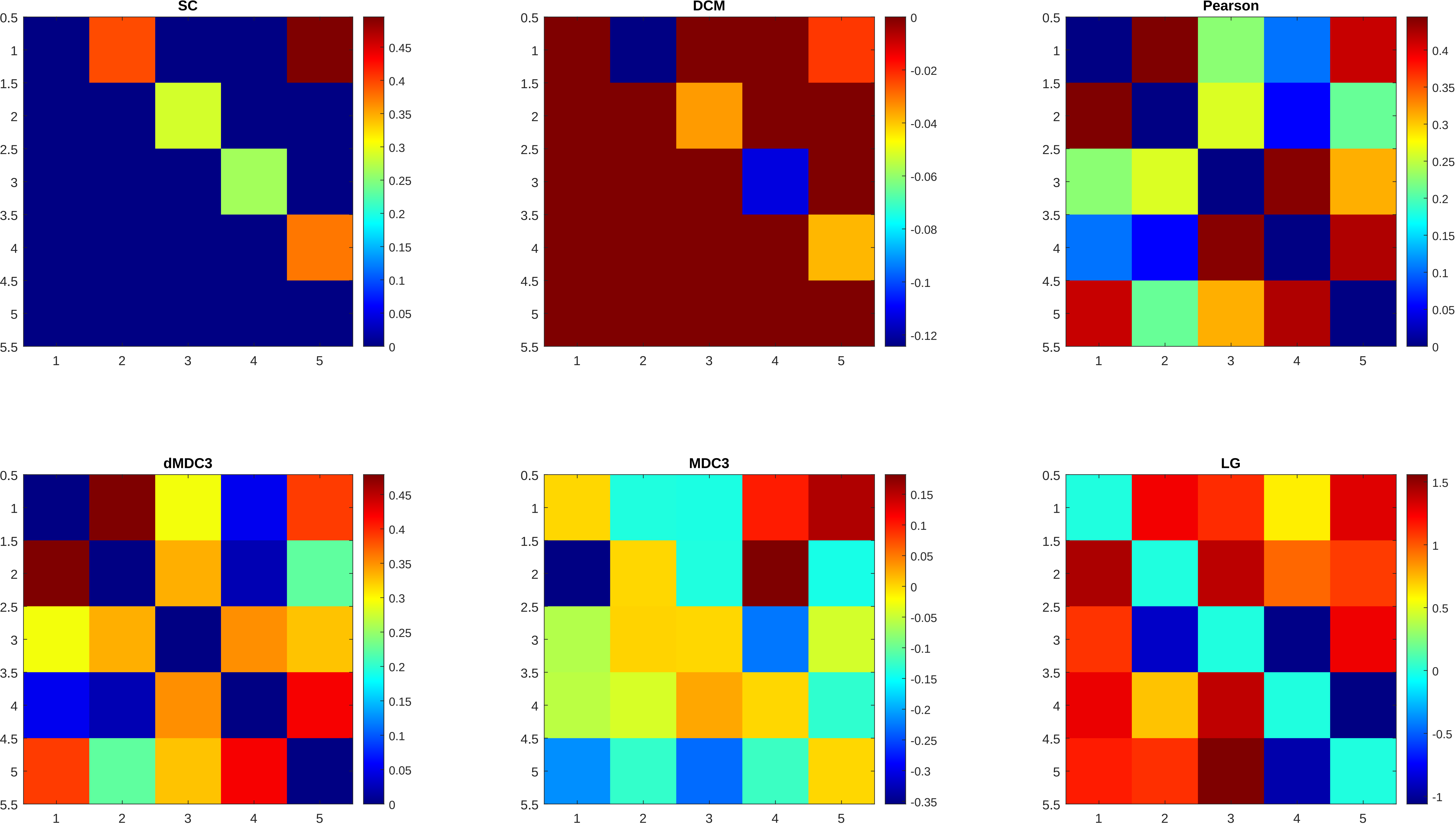
SC
DCM
Pearson
dMDC3
MDC3
LG

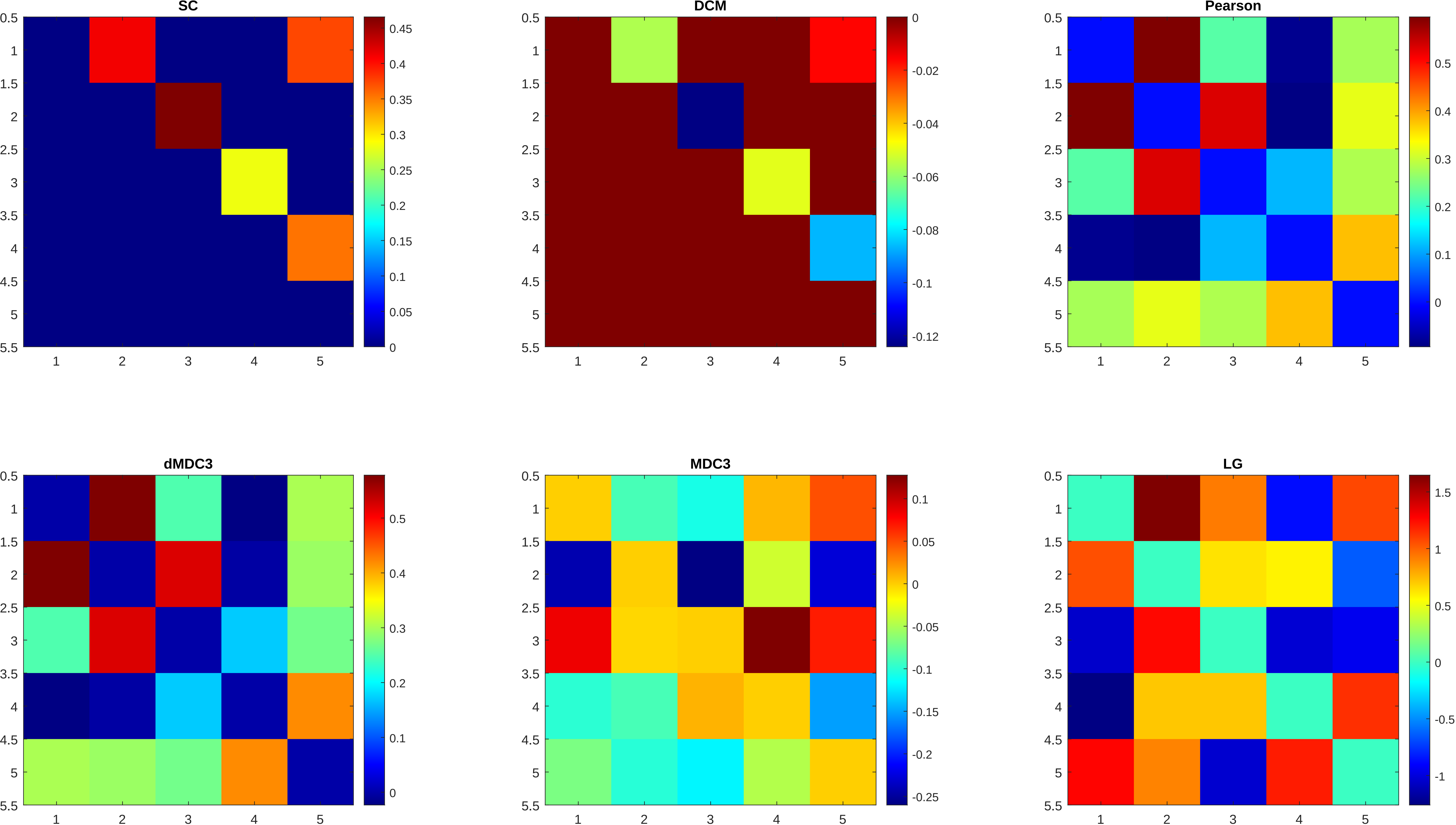
SC
DCM
Pearson
dMDC3
MDC3
LG

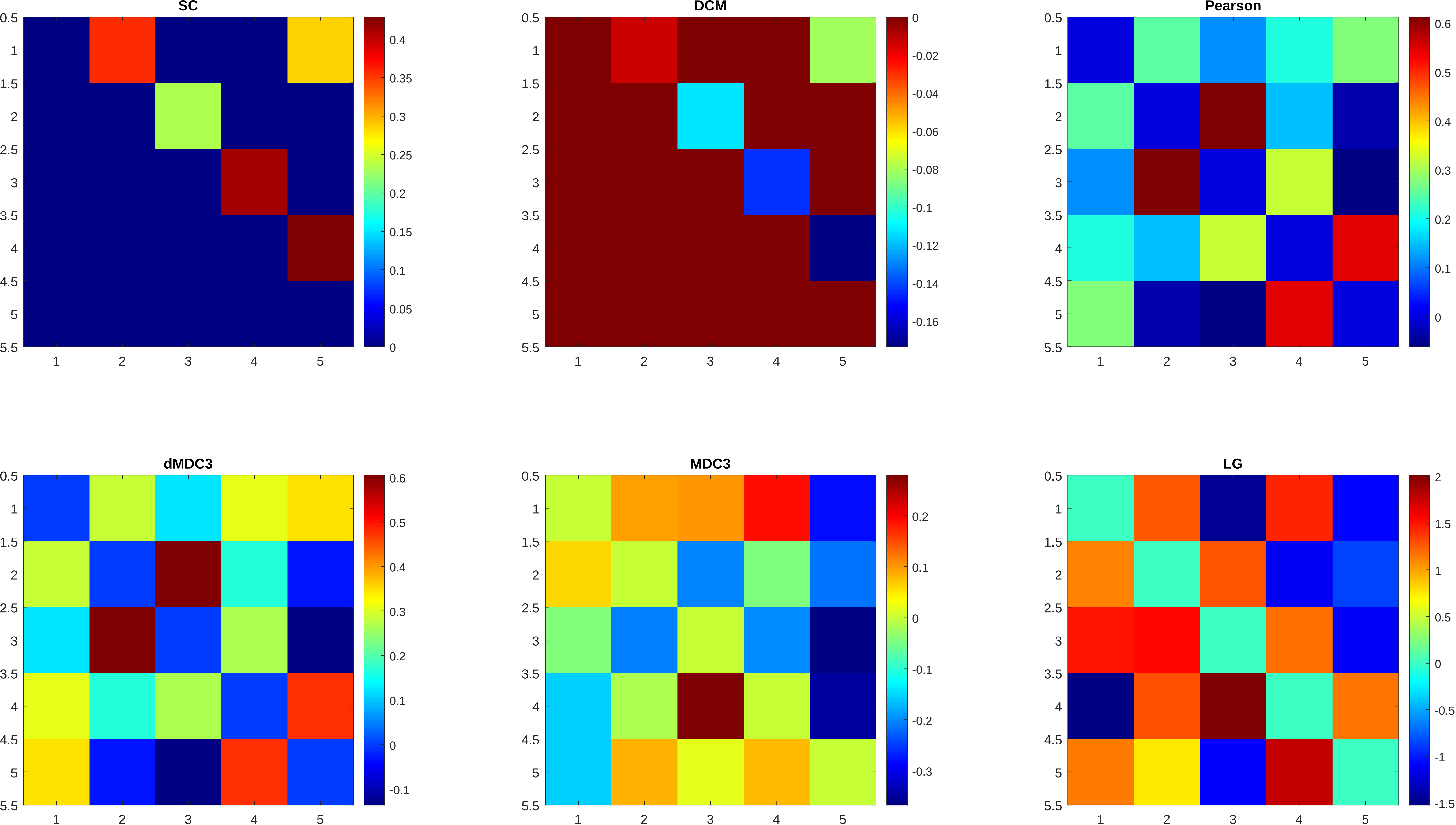
SC
DCM
Pearson
dMDC3
MDC3
LG

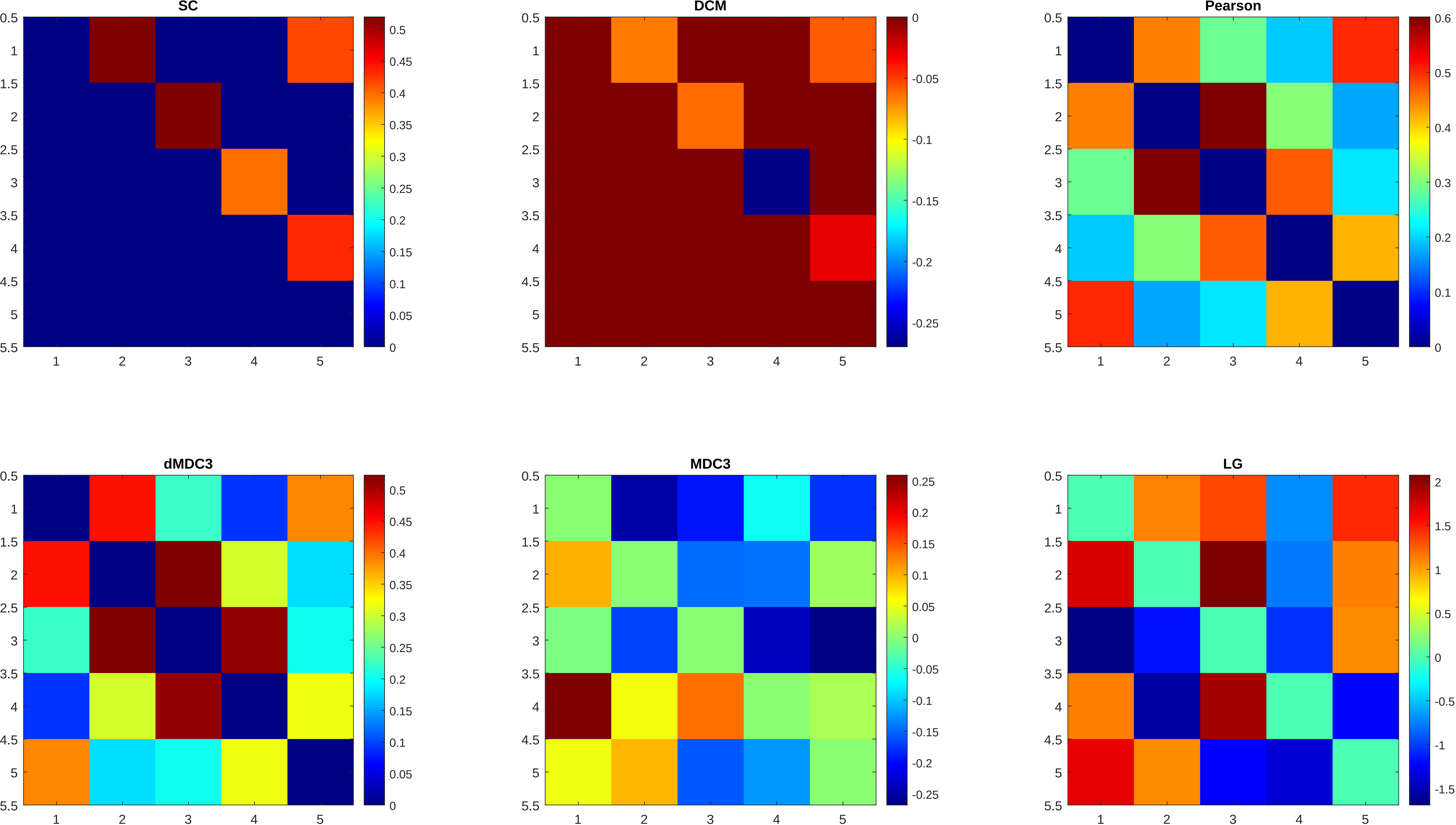
SC
DCM
Pearson
dMDC3
MDC3
LG
0.5
1
1.5
2
2.5
3
3.5
4
4.5
5
5.5
1
2
3
4
5
0.45
0.4
0.35
0.3
0.25
0.2
0.15
0.1
0.05
0
-0.05
-0.1
-0.15
-0.2
-0.25
0.6
0.25
-0.5
-1
-1.5

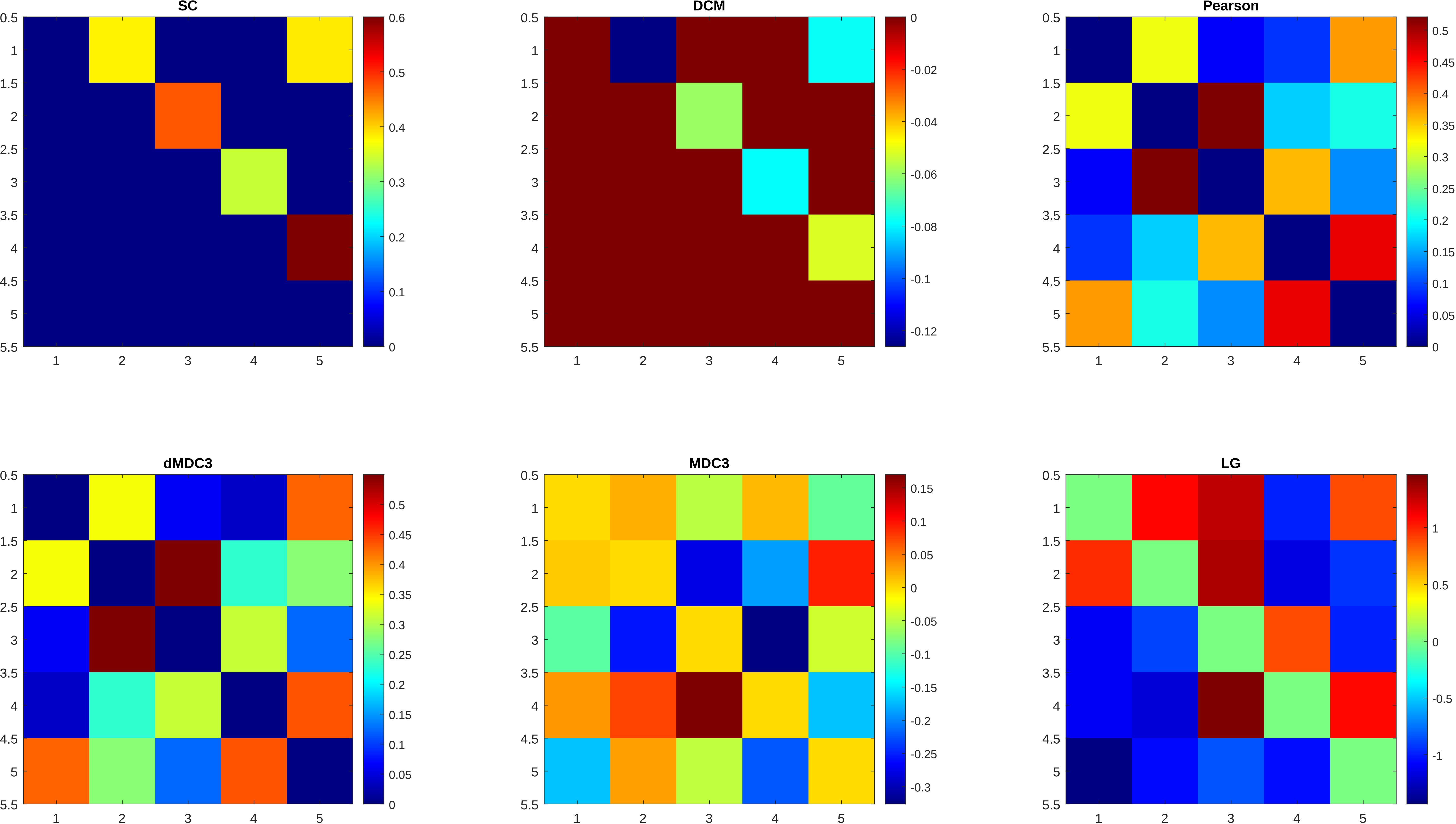
SC
0.6
0.5
0.4
0.3
0.2
0.1
0
DCM
0
-0.02
-0.04
-0.06
-0.08
-0.1
-0.12
Pearson
0.5
0.45
0.4
0.35
0.3
0.25
0.2
0.15
0.1
0.05
0
dMDC3
0.5
0.45
0.4
0.35
0.3
0.25
0.2
0.15
0.1
0.05
0
MDC3
0.15
0.1
0.05
0
-0.05
-0.1
-0.15
-0.2
-0.25
-0.3
LG
1
0.5
0
-0.5
-1
0.5
1
1.5
2
2.5
3
3.5
4
4.5
5
5.5
1
2
3
4
5

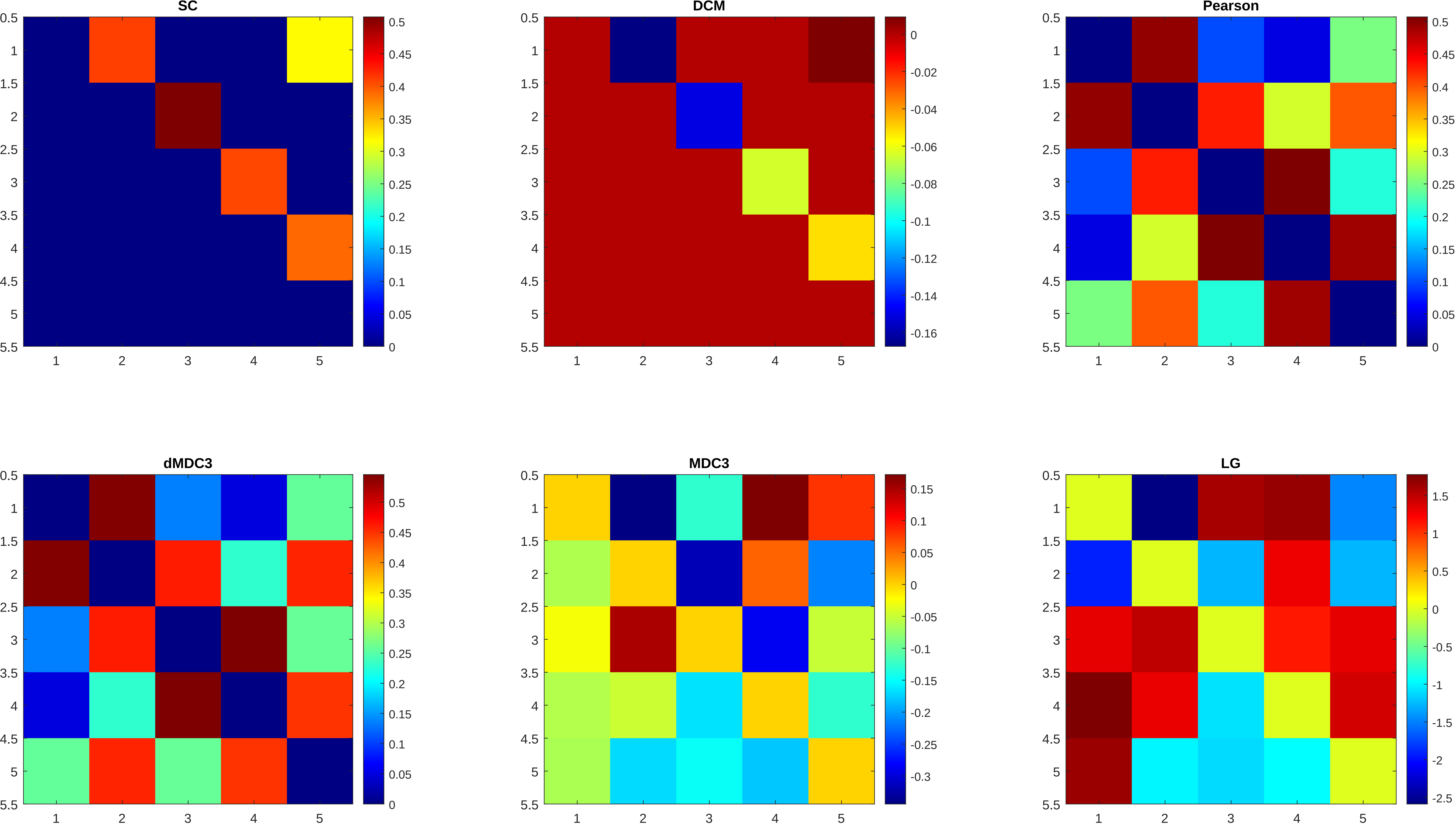
SC
DCM
Pearson
dMDC3
MDC3
LG

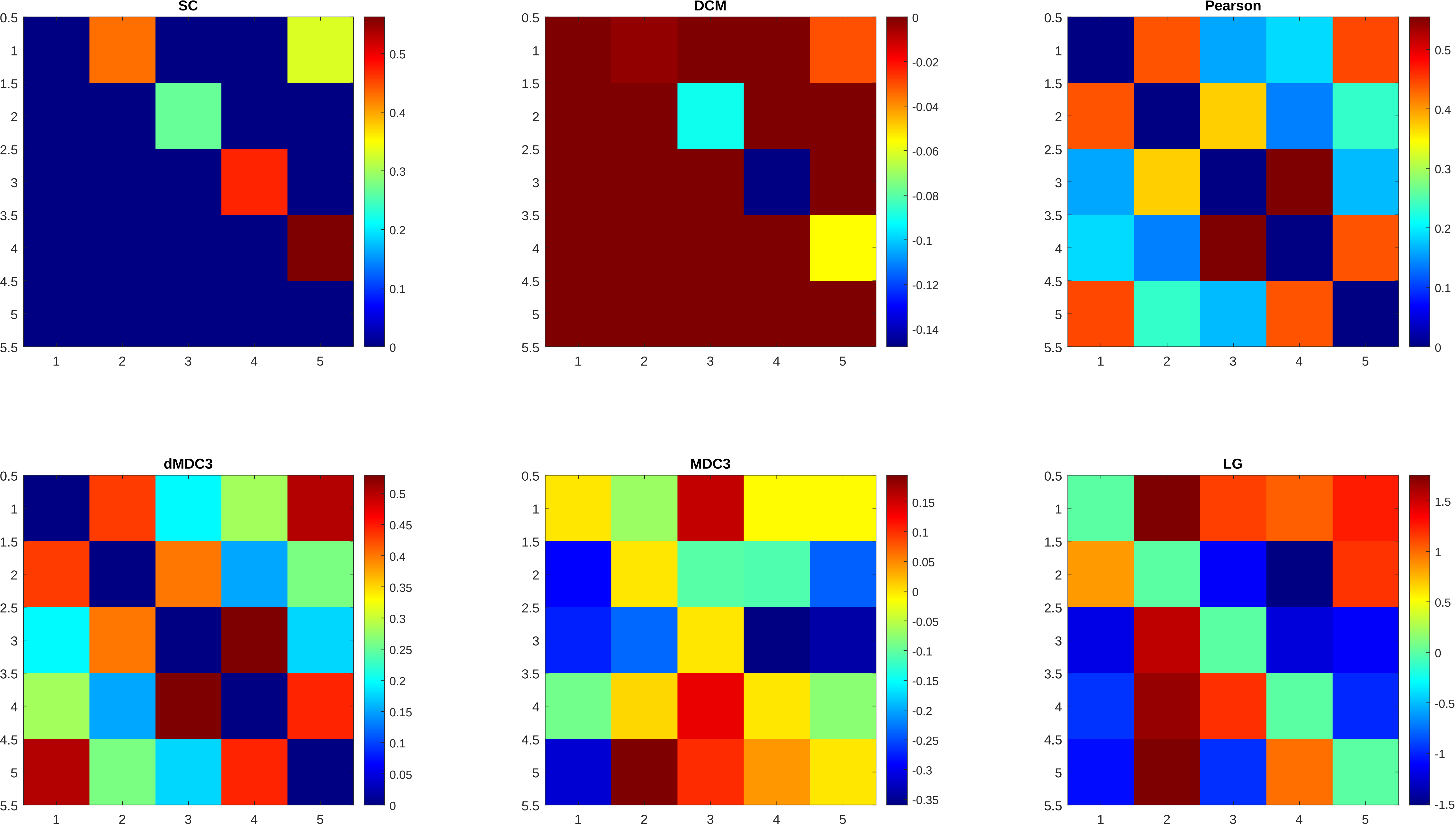
SC
DCM
Pearson
dMDC3
MDC3
LG

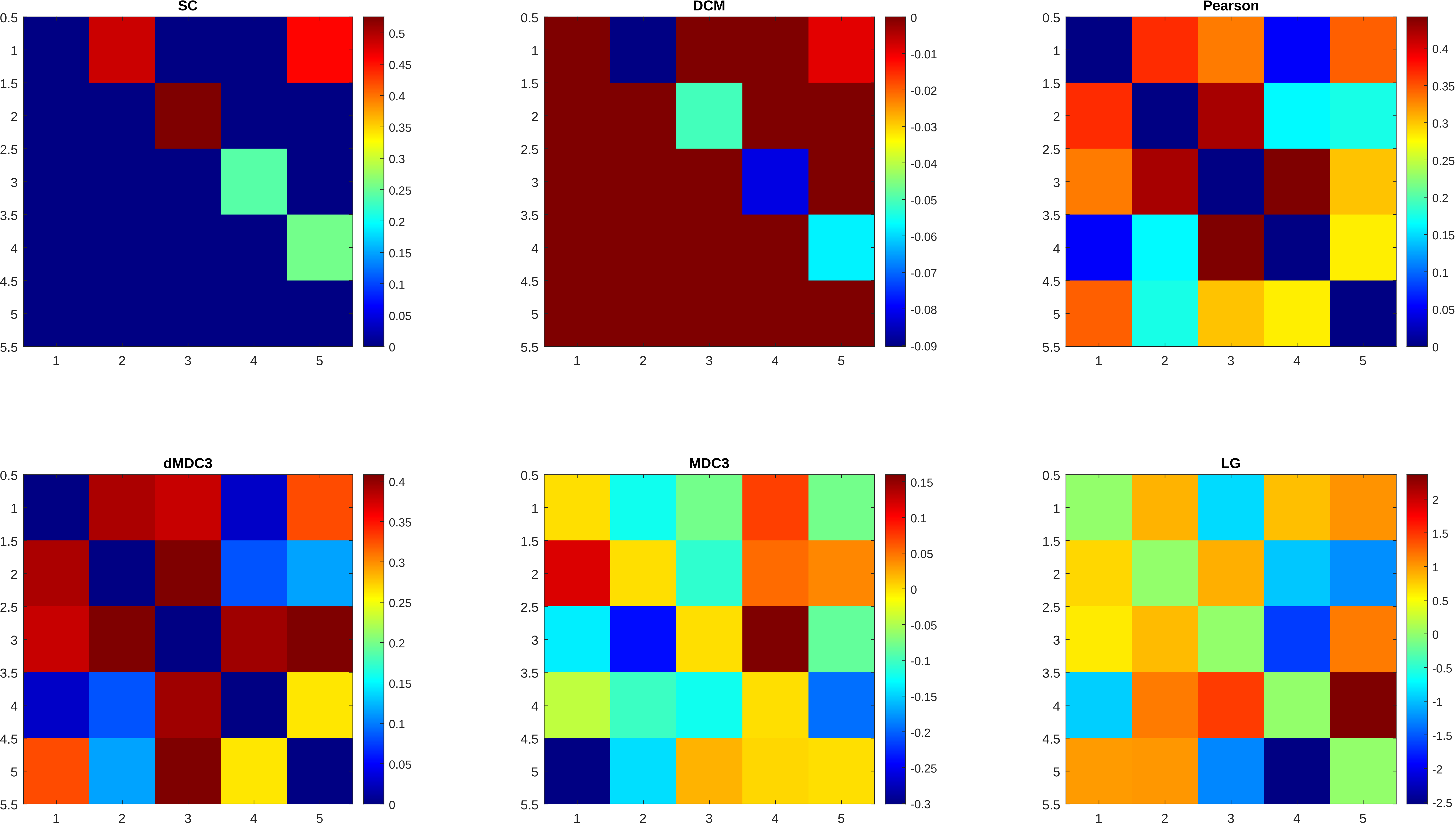
SC
DCM
Pearson
dMDC3
MDC3
LG
0.5 1 1.5 2 2.5 3 3.5 4 4.5 5 5.5
1 2 3 4 5
0 0.05 0.1 0.15 0.2 0.25 0.3 0.35 0.4 0.45 0.5
0 -0.01 -0.02 -0.03 -0.04 -0.05 -0.06 -0.07 -0.08 -0.09
0 0.05 0.1 0.15 0.2 0.25 0.3 0.35 0.4
0 0.05 0.1 0.15 0.2 0.25 0.3 0.35 0.4
0.15 0.1 0.05 0 -0.05 -0.1 -0.15 -0.2 -0.25 -0.3
2 1.5 1 0.5 0 -0.5 -1 -1.5 -2 -2.5

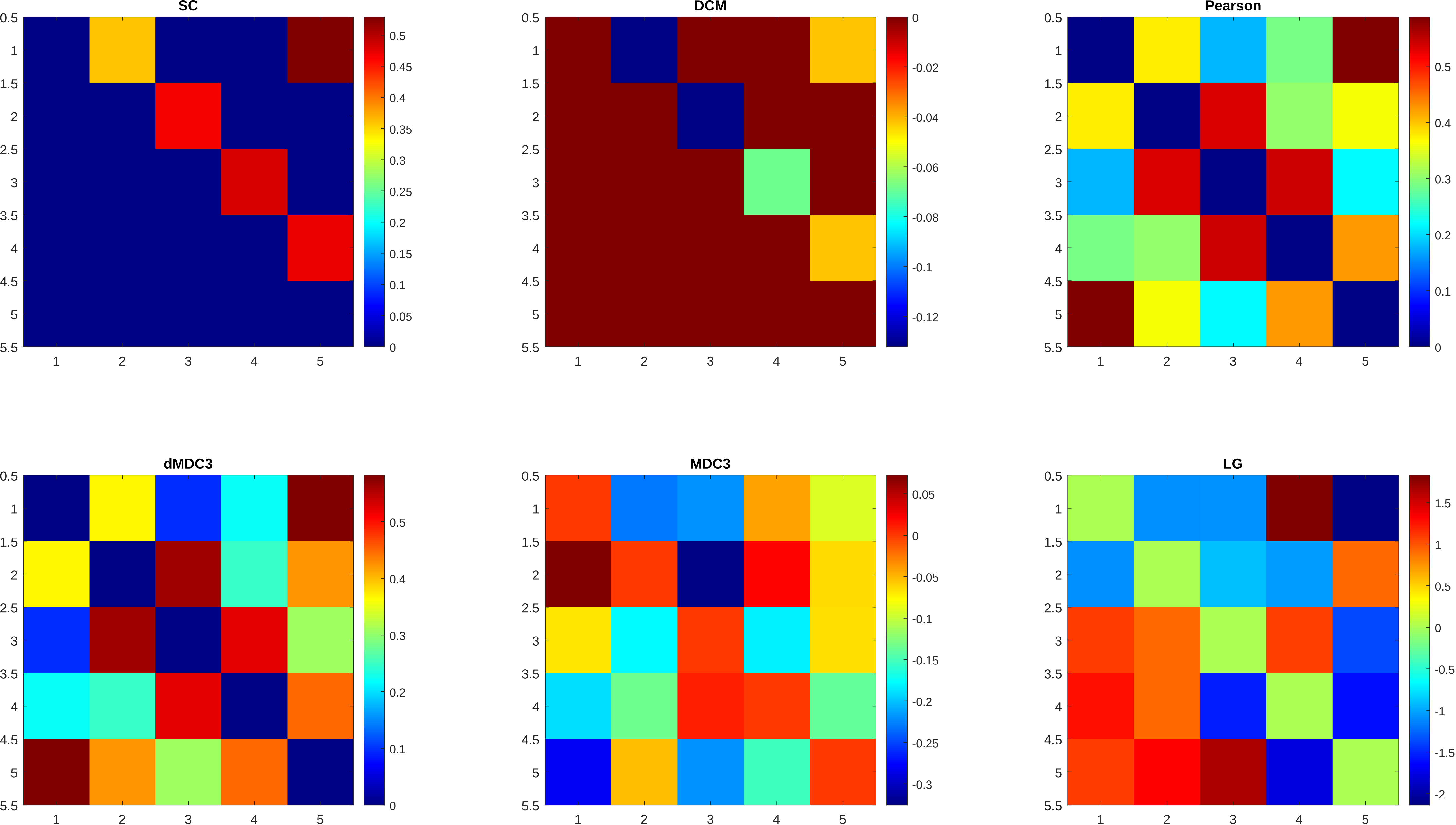
SC
DCM
Pearson
dMDC3
MDC3
LG

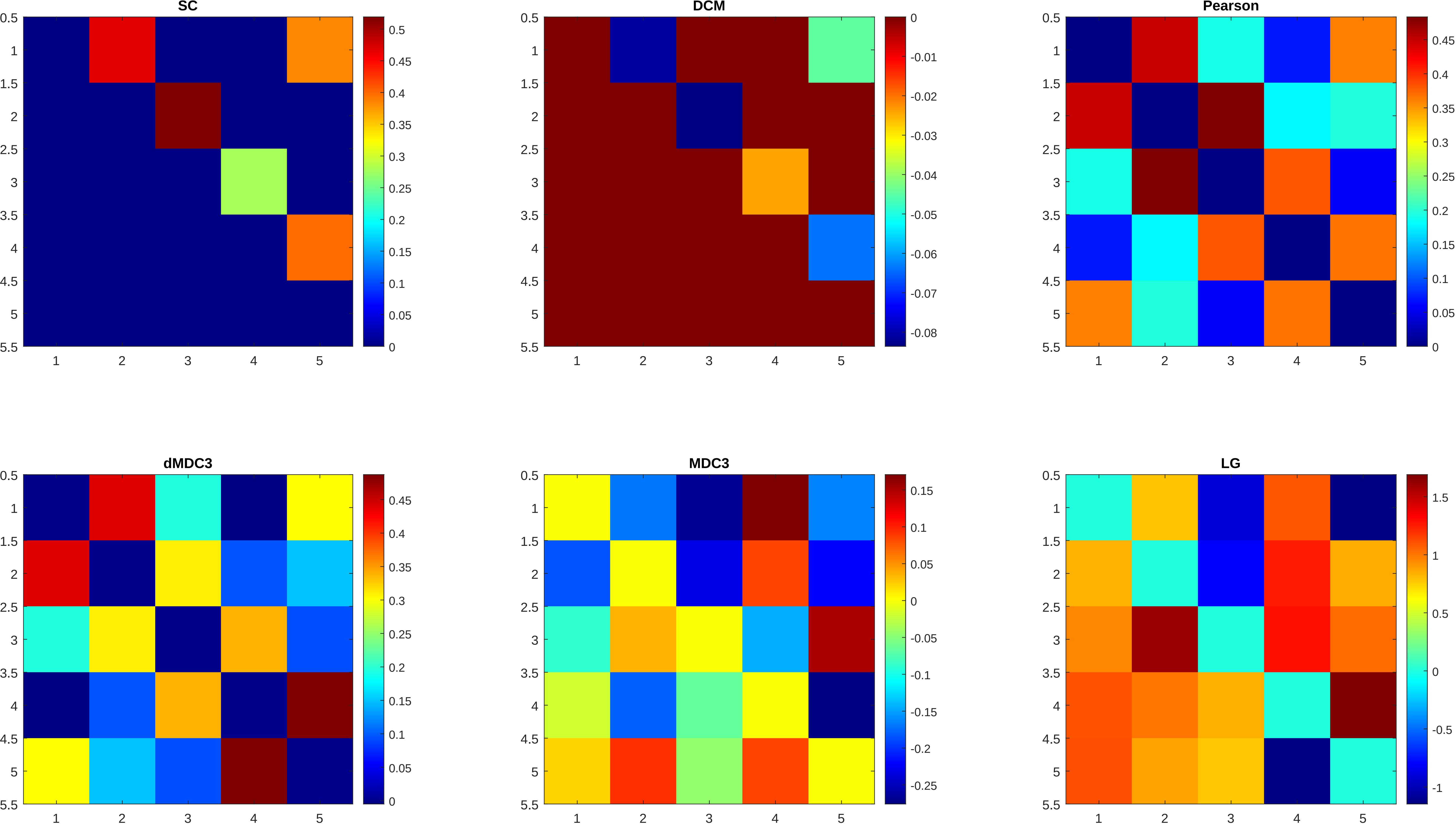
SC
DCM
Pearson
dMDC3
MDC3
LG

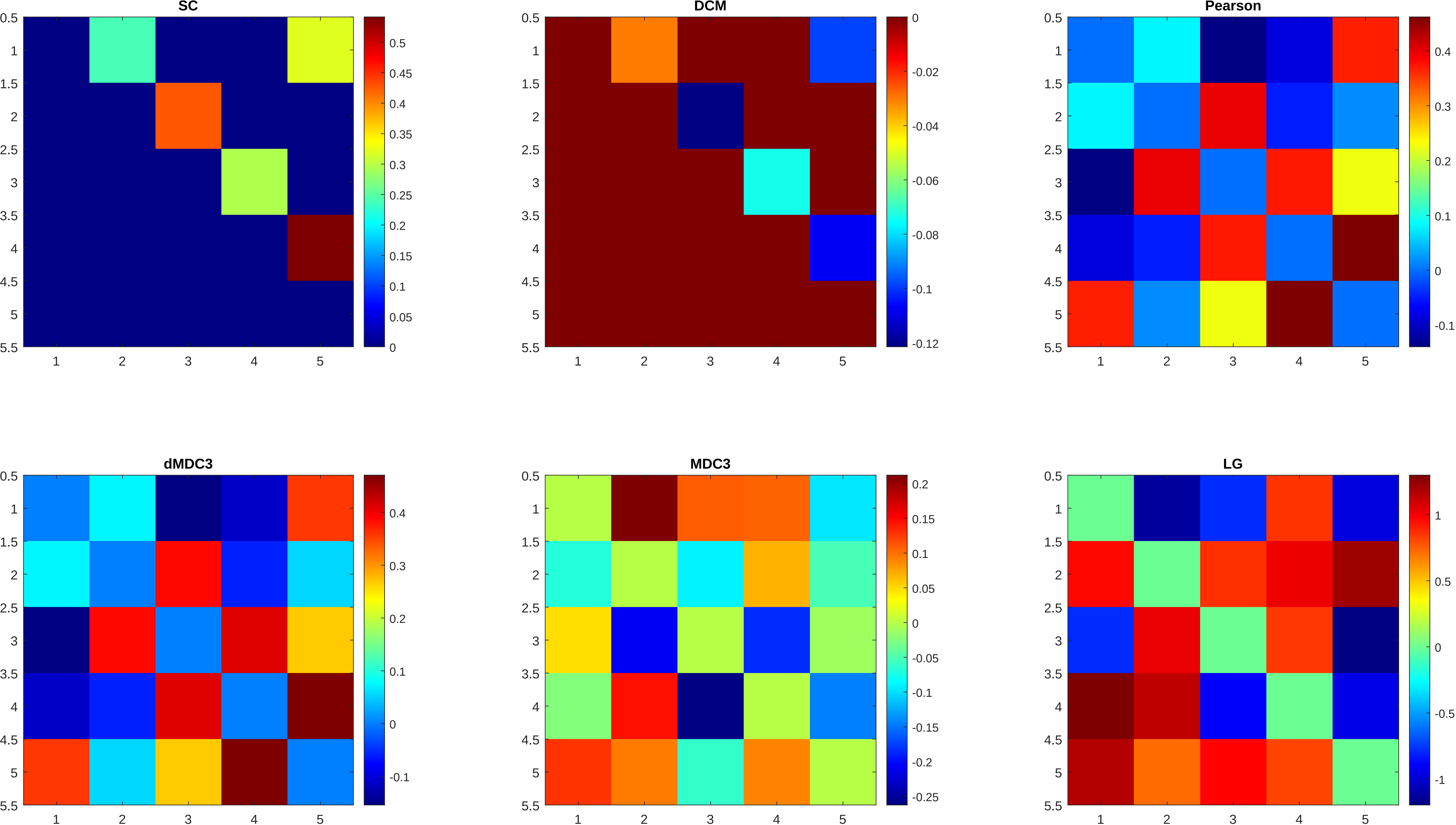
SC
DCM
Pearson
dMDC3
MDC3
LG
0.5
1
1.5
2
2.5
3
3.5
4
4.5
5
5.5
1
2
3
4
5
0.5
0.45
0.4
0.35
0.3
0.25
0.2
0.15
0.1
0.05
0
-0.02
-0.04
-0.06
-0.08
-0.1
-0.12
0.4
0.3
0.2
0.1
-0.1
0.15
-0.05
-0.15
-0.2
-0.25
-0.5
-1

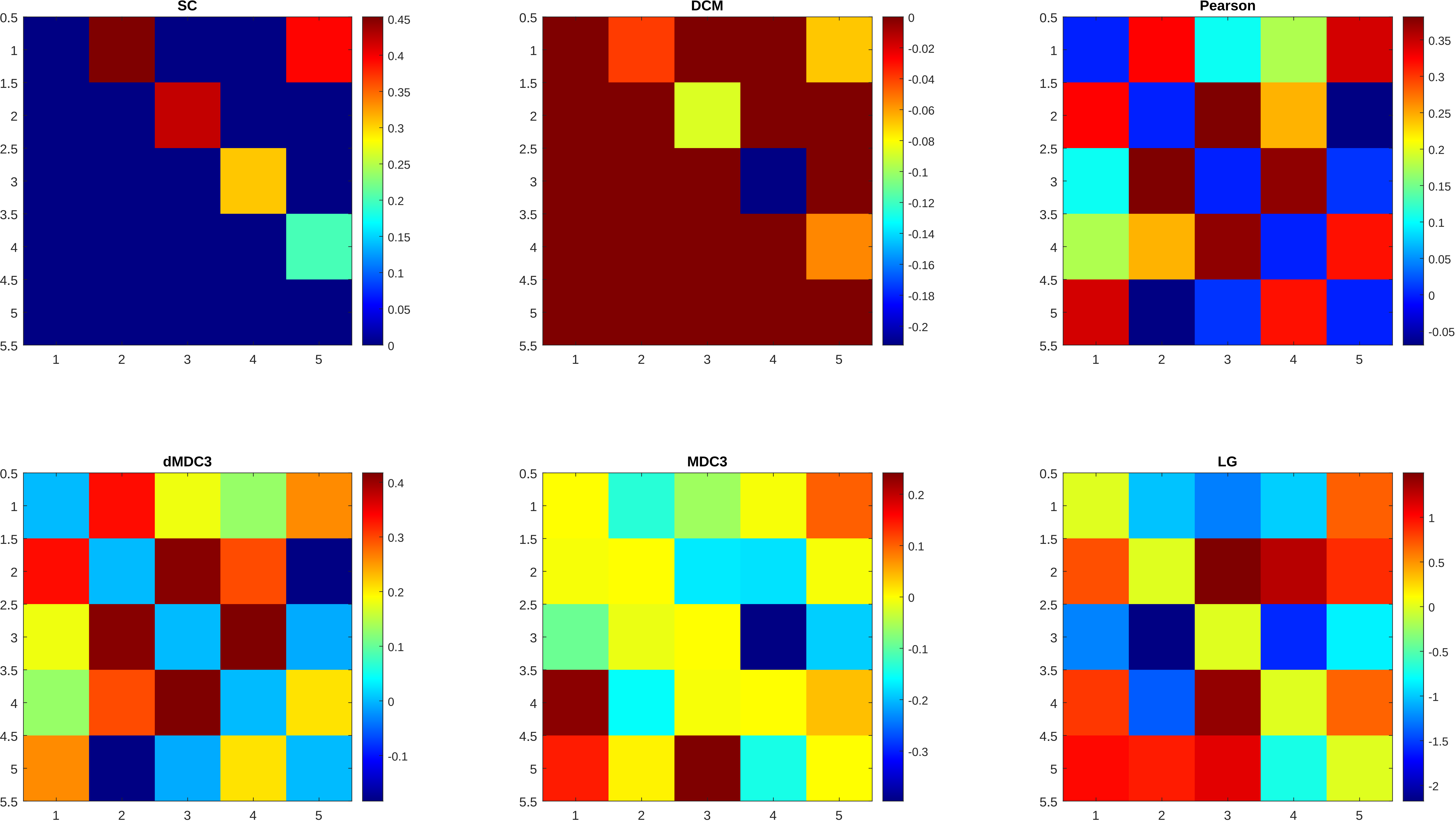
SC
DCM
Pearson
dMDC3
MDC3
LG
0.5
1
1.5
2
2.5
3
3.5
4
4.5
5
5.5
1
2
3
4
5
0.45
0.4
0.35
0.3
0.25
0.2
0.15
0.1
0.05
0
-0.02
-0.04
-0.06
-0.08
-0.1
-0.12
-0.14
-0.16
-0.18
-0.2
-0.05
-0.1
-0.3
-0.5
-1
-1.5
-2

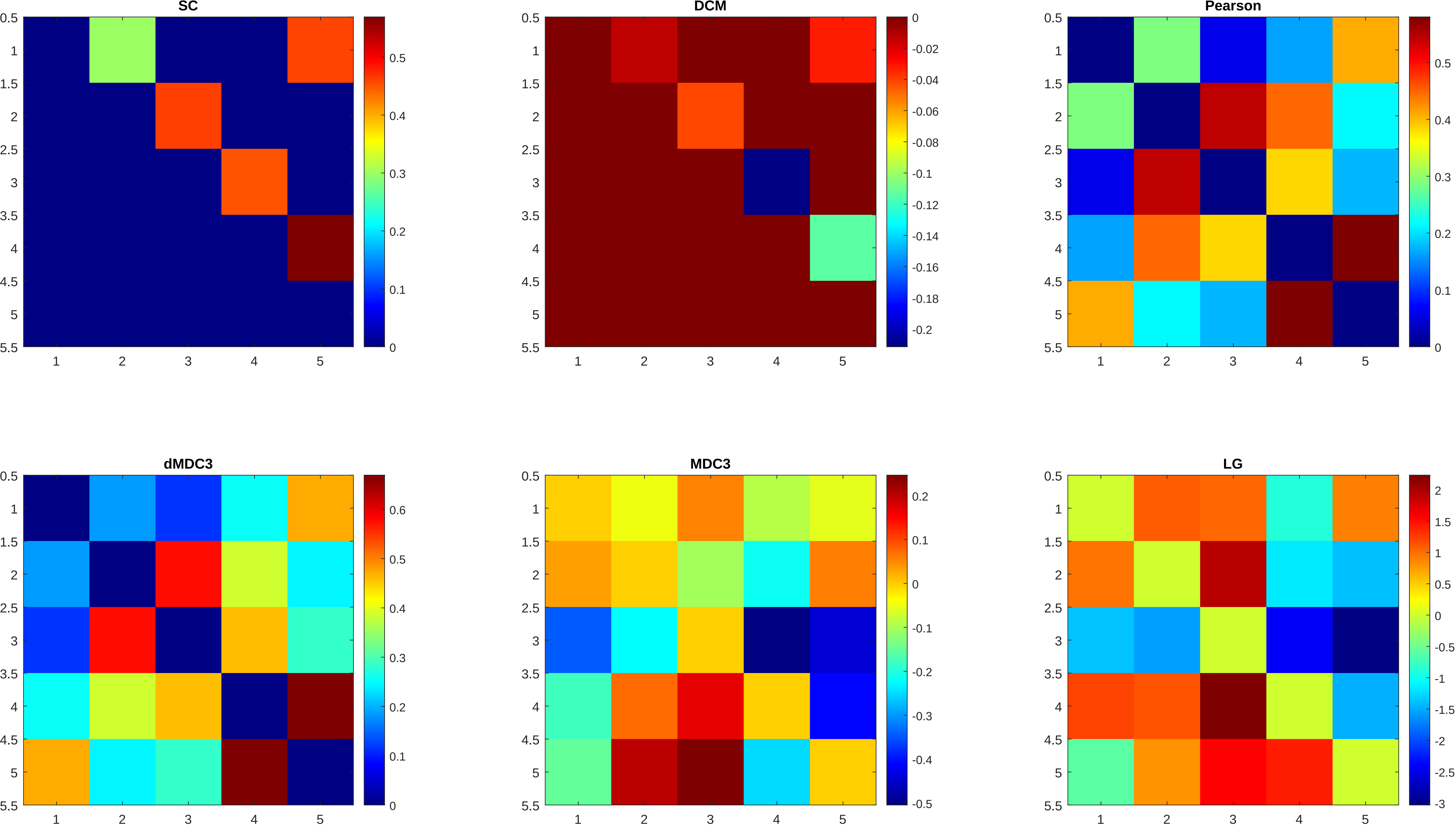
SC
DCM
Pearson
dMDC3
MDC3
LG

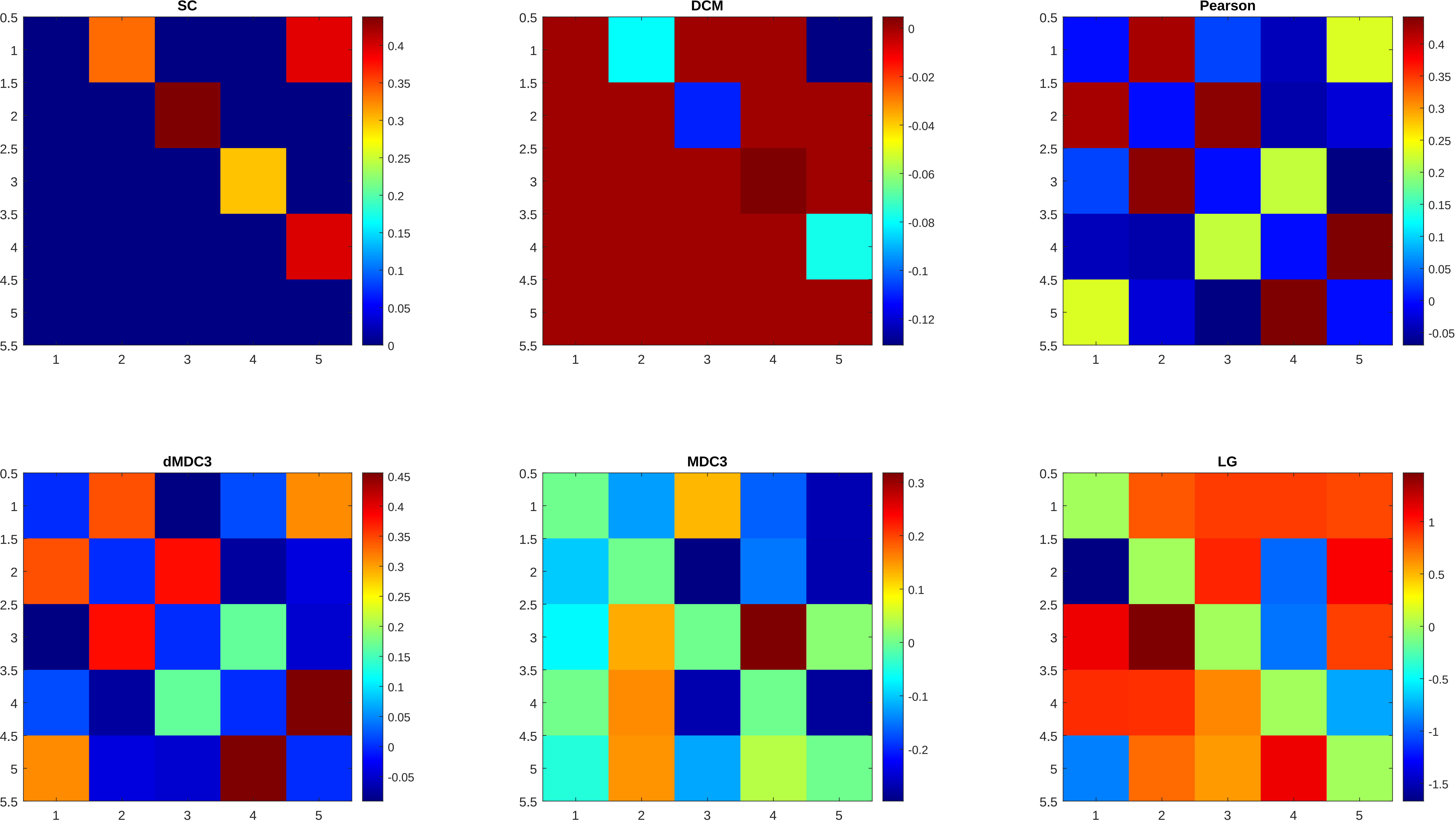
SC
0.5 1 1.5 2 2.5 3 3.5 4 4.5 5 5.5
1 2 3 4 5
0 0.05 0.1 0.15 0.2 0.25 0.3 0.35 0.4
DCM
0 -0.02 -0.04 -0.06 -0.08 -0.1 -0.12
Pearson
0.4 0.35 0.3 0.25 0.2 0.15 0.1 0.05 0 -0.05
dMDC3
0.45 0.4 0.35 0.3 0.25 0.2 0.15 0.1 0.05 0 -0.05
MDC3
0.3 0.2 0.1 0 -0.1 -0.2
LG
1 0.5 0 -0.5 -1 -1.5

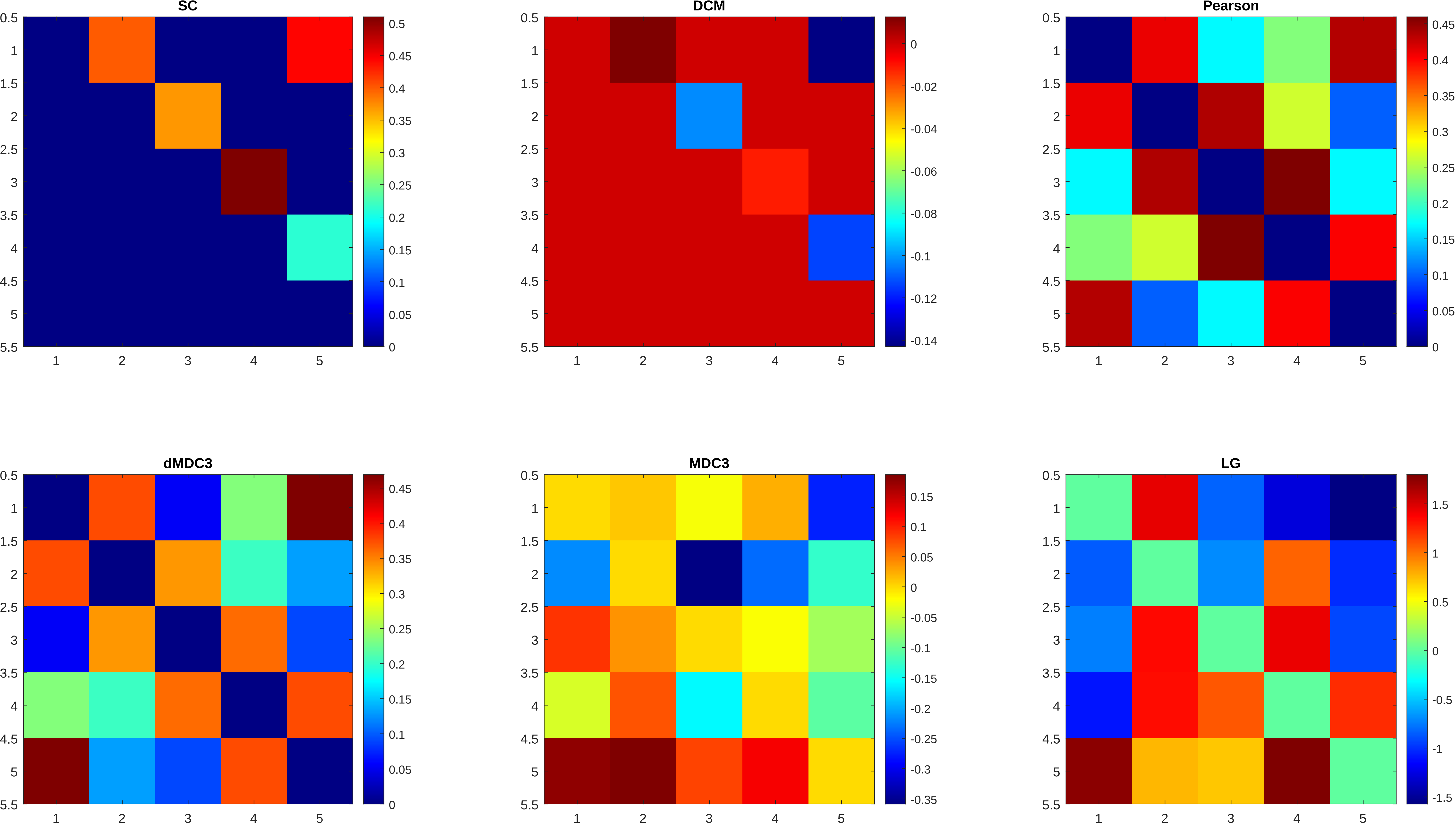
SC
DCM
Pearson
dMDC3
MDC3
LG

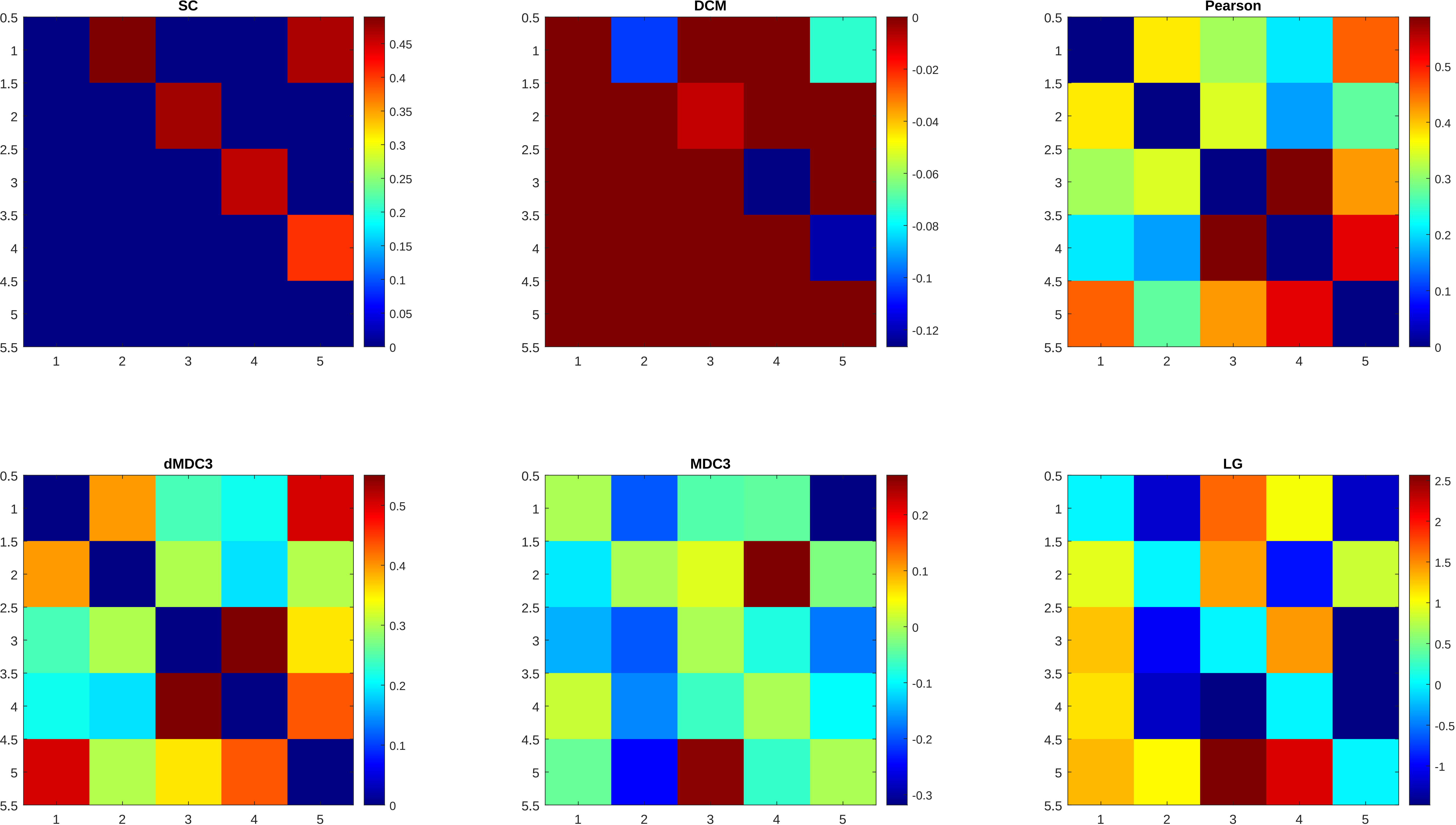

SC
DCM
Pearson
dMDC3
MDC3
LG

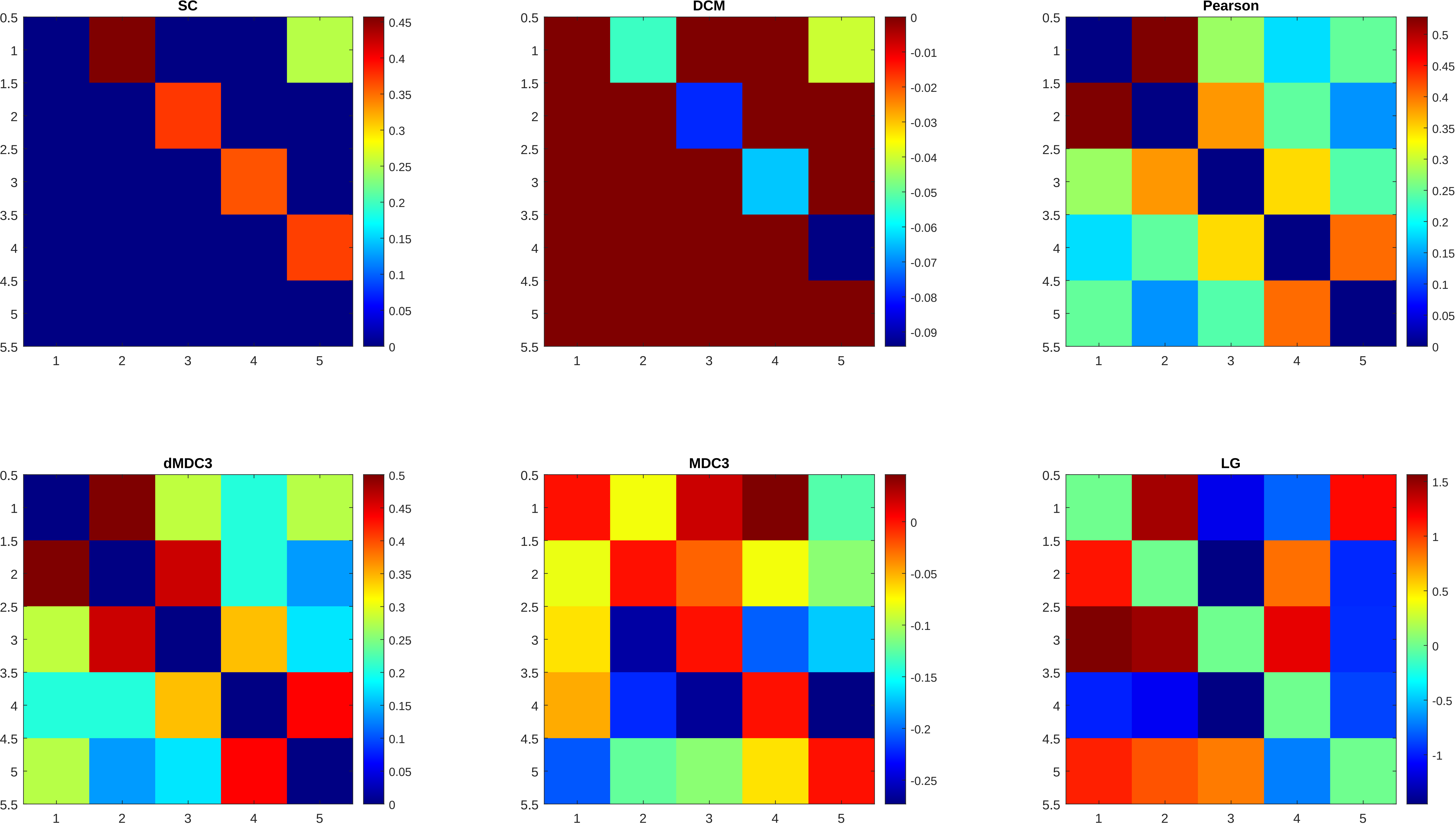
SC
DCM
Pearson
dMDC3
MDC3
LG
0.5
1
1.5
2
2.5
3
3.5
4
4.5
5
5.5
0.45
0.4
0.35
0.3
0.25
0.2
0.15
0.1
0.05
0
-0.01
-0.02
-0.03
-0.04
-0.05
-0.06
-0.07
-0.08
-0.09
-0.1
-0.15
-0.2
-0.25
-0.5
-1

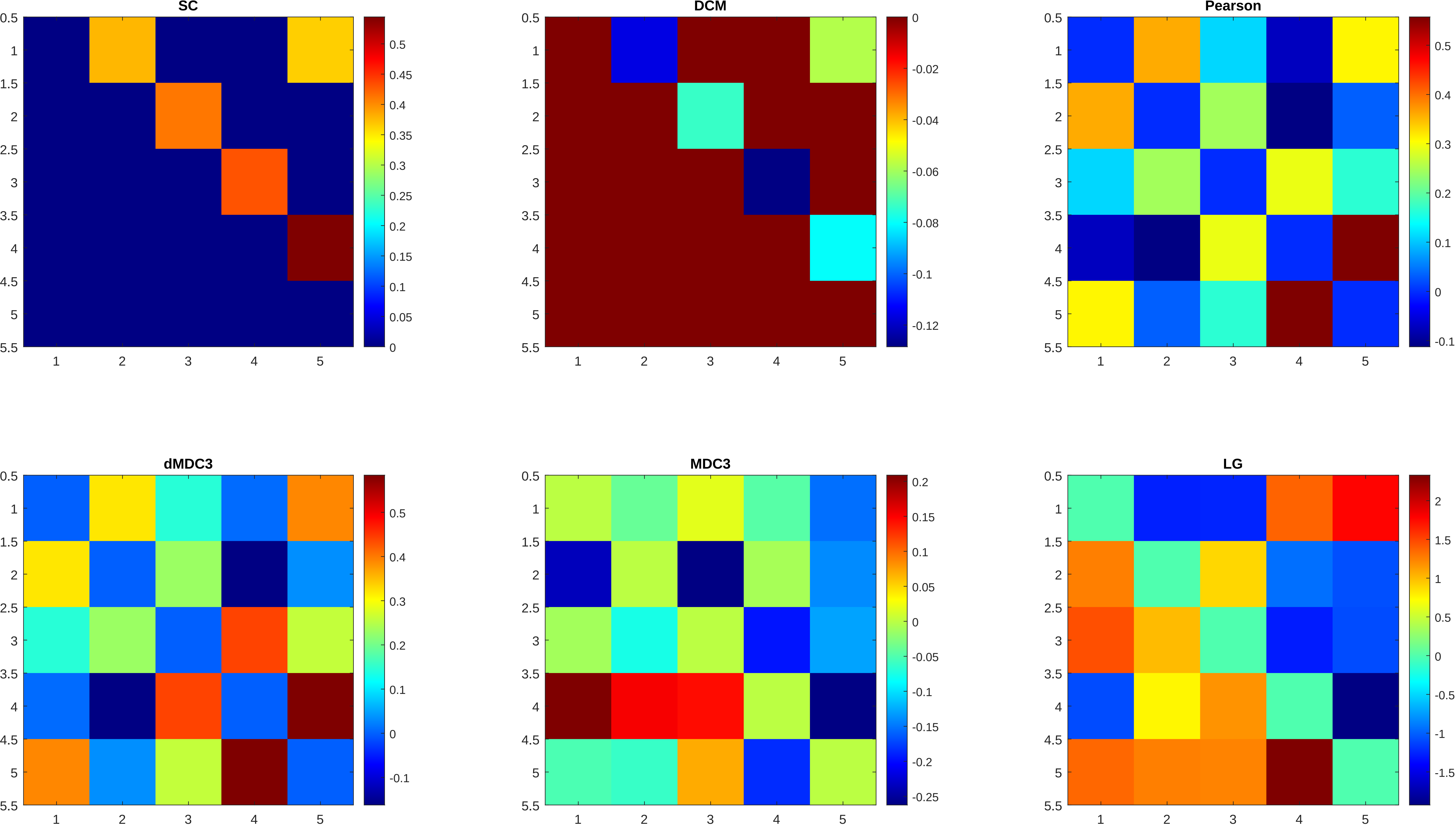
SC
DCM
Pearson
dMDC3
MDC3
LG
0.5 1 1.5 2 2.5 3 3.5 4 4.5 5 5.5
1 2 3 4 5
0 0.05 0.1 0.15 0.2 0.25 0.3 0.35 0.4 0.45 0.5
0 -0.02 -0.04 -0.06 -0.08 -0.1 -0.12
-0.1 0 0.1 0.2 0.3 0.4 0.5
-0.1 0 0.1 0.2 0.3 0.4 0.5
-0.25 -0.2 -0.15 -0.1 -0.05 0 0.05 0.1 0.15 0.2
-1.5 -1 -0.5 0 0.5 1 1.5 2

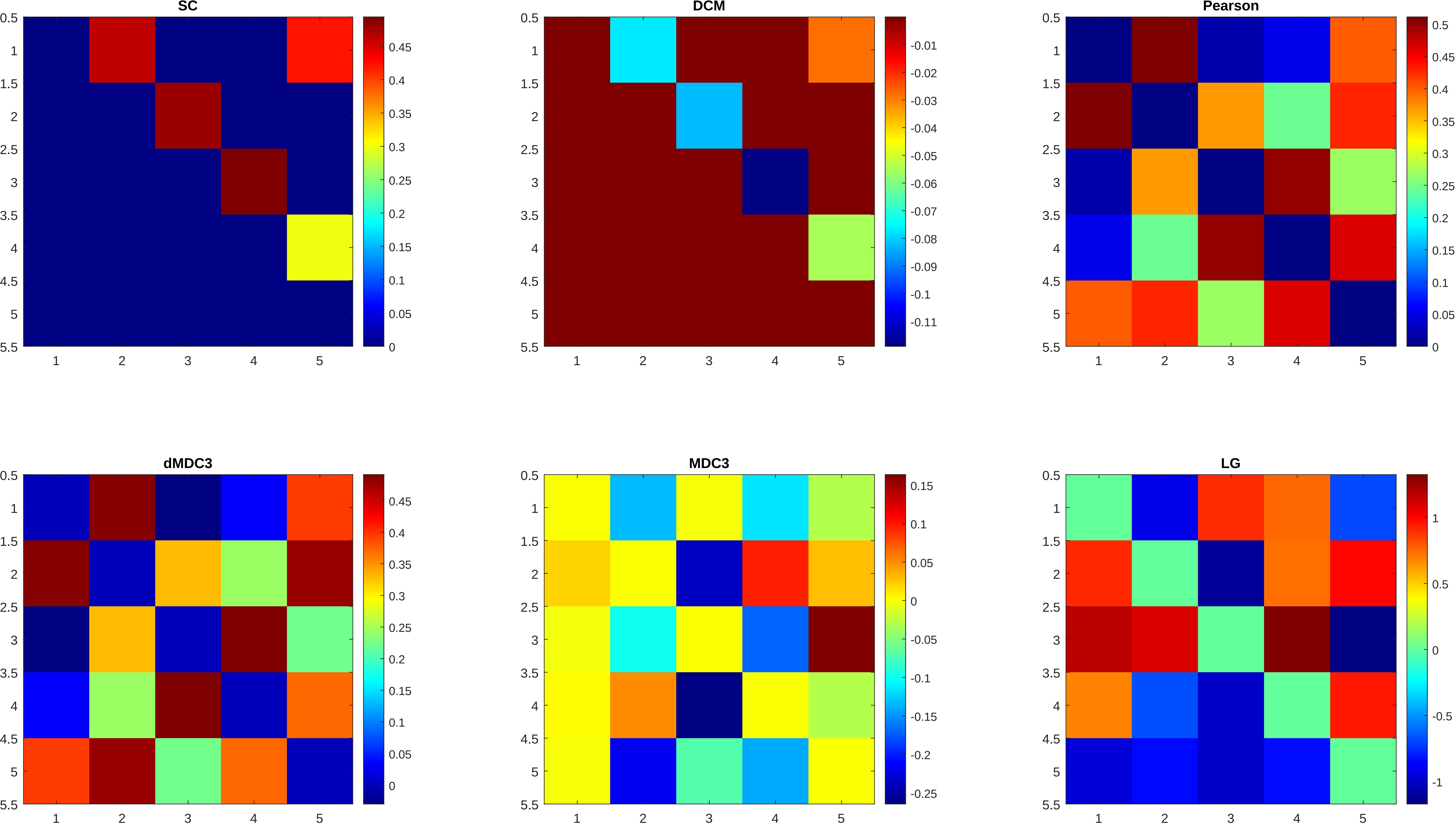
SC
DCM
Pearson
dMDC3
MDC3
LG
0.5
1
1.5
2
2.5
3
3.5
4
4.5
5
5.5
1
2
3
4
5
0.45
0.4
0.35
0.3
0.25
0.2
0.15
0.1
0.05
0
-0.01
-0.02
-0.03
-0.04
-0.05
-0.06
-0.07
-0.08
-0.09
-0.1
-0.11
0.15
0.1
0.05
0
-0.05
-0.1
-0.15
-0.2
-0.25
1
0.5
0
-0.5
-1

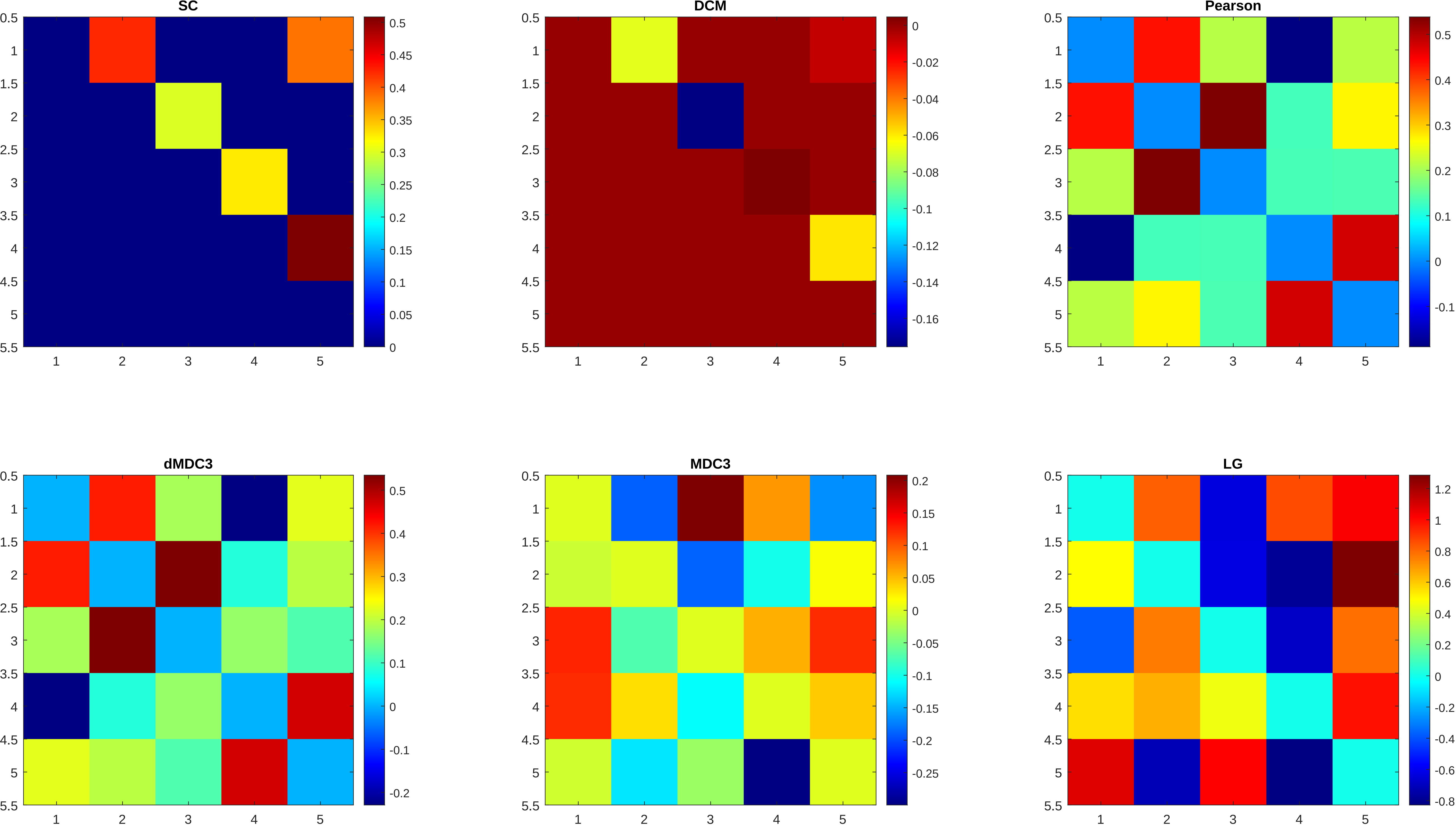
SC
0.5
0.45
0.4
0.35
0.3
0.25
0.2
0.15
0.1
0.05
0
DCM
0
-0.02
-0.04
-0.06
-0.08
-0.1
-0.12
-0.14
-0.16
Pearson
0.5
0.4
0.3
0.2
0.1
0
-0.1
dMDC3
0.5
0.4
0.3
0.2
0.1
0
-0.1
-0.2
MDC3
0.2
0.15
0.1
0.05
0
-0.05
-0.1
-0.15
-0.2
-0.25
LG
1.2
1
0.8
0.6
0.4
0.2
0
-0.2
-0.4
-0.6
-0.8
0.5
1
1.5
2
2.5
3
3.5
4
4.5
5
5.5
1
2
3
4
5

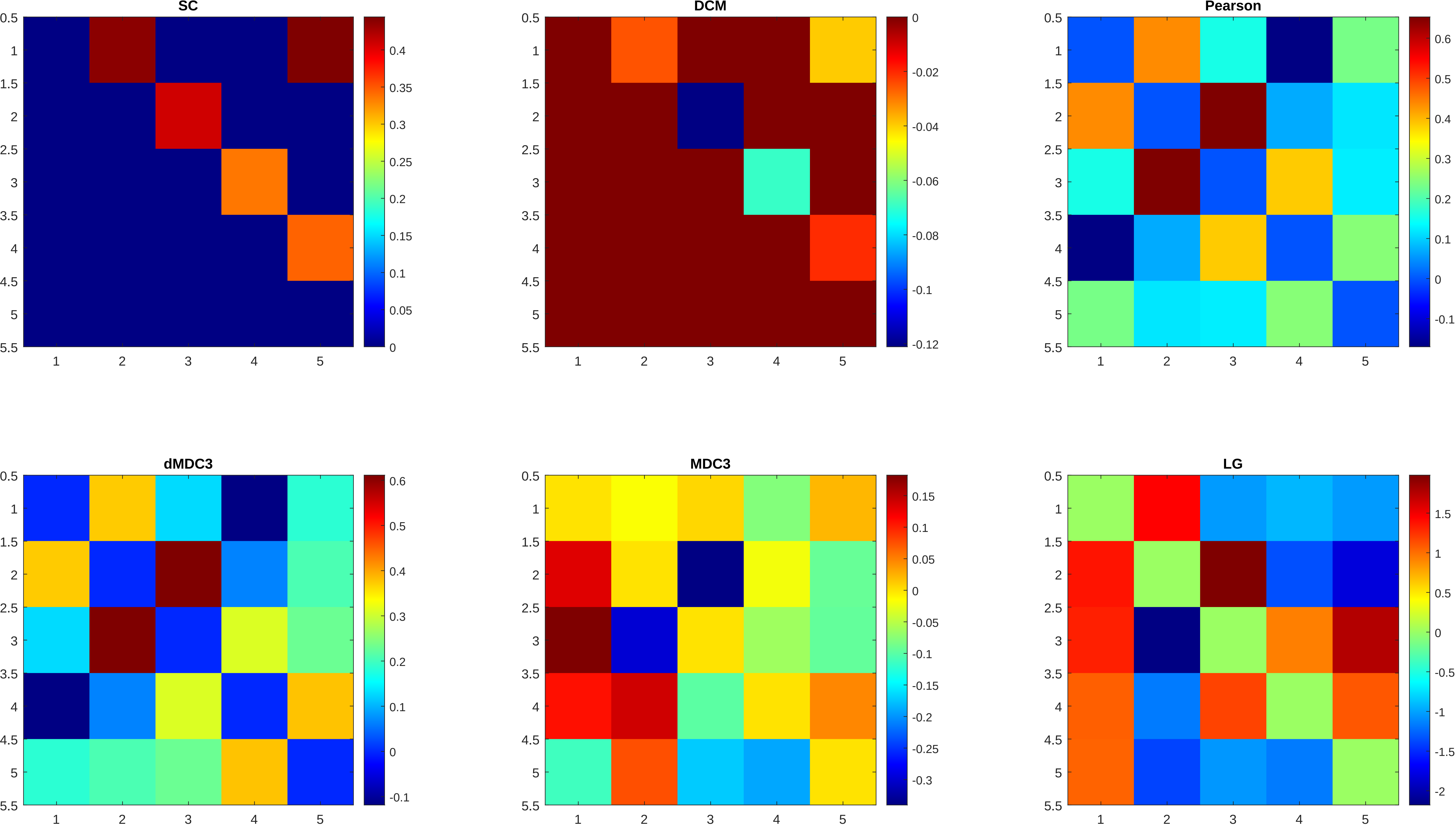
SC
DCM
Pearson
dMDC3
MDC3
LG

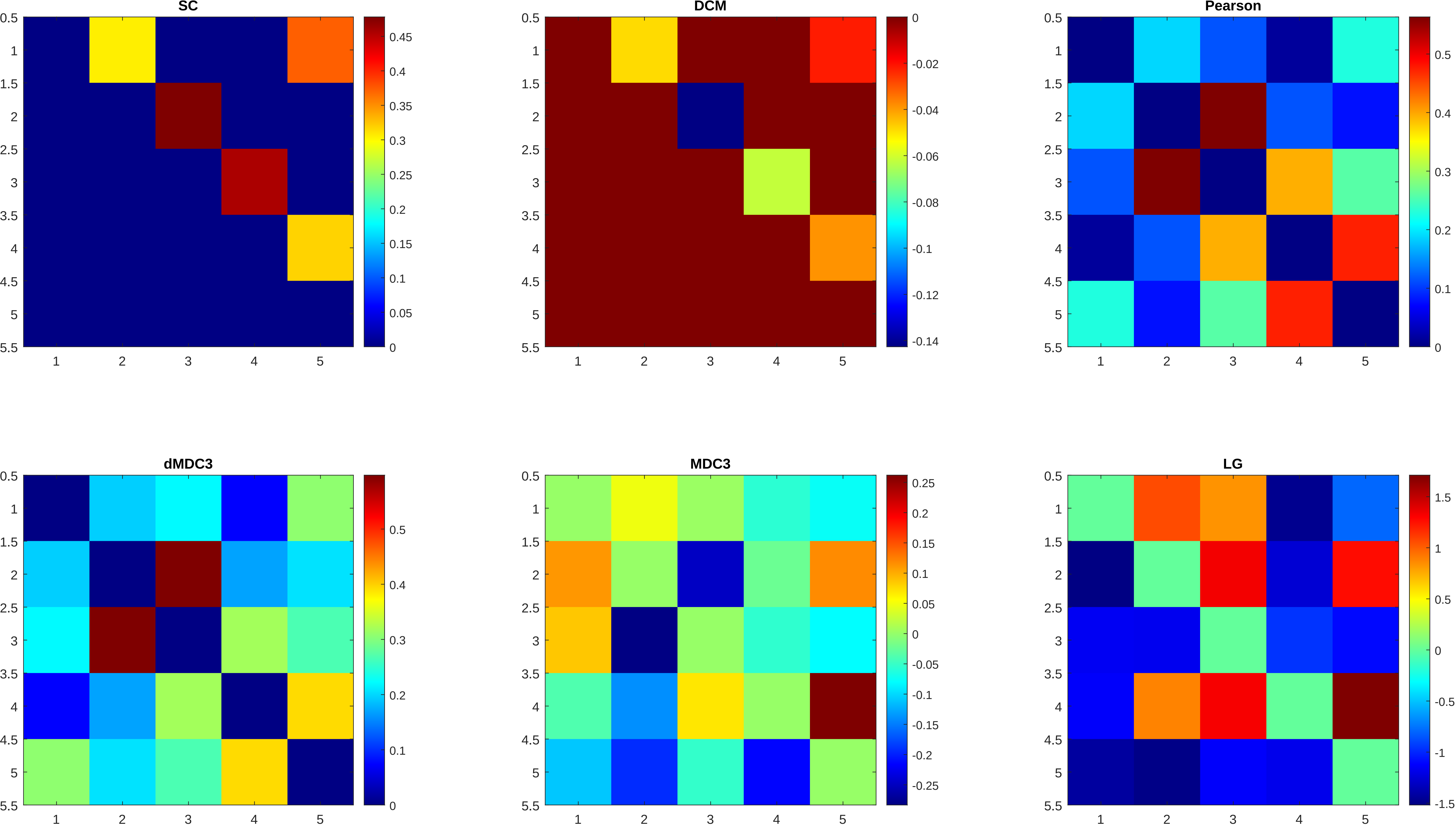
SC
0.5
1
1.5
2
2.5
3
3.5
4
4.5
5
5.5
1 2 3 4 5
0.45
0.4
0.35
0.3
0.25
0.2
0.15
0.1
0.05
0
DCM
0
-0.02
-0.04
-0.06
-0.08
-0.1
-0.12
-0.14
Pearson
0.5
0.4
0.3
0.2
0.1
0
dMDC3
0.5
0.4
0.3
0.2
0.1
0
MDC3
0.25
0.2
0.15
0.1
0.05
0
-0.05
-0.1
-0.15
-0.2
-0.25
LG
1.5
1
0.5
0
-0.5
-1
-1.5

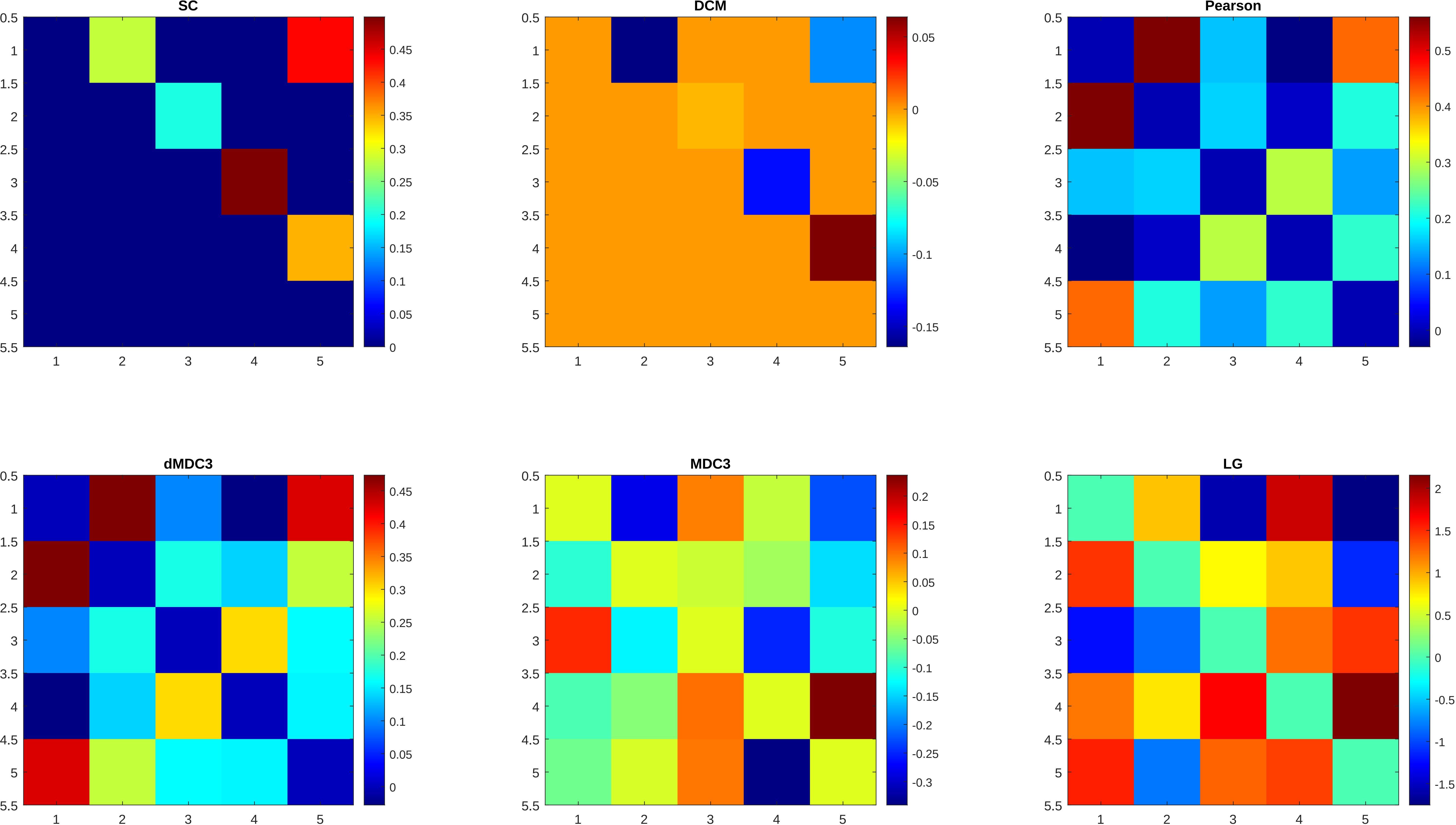
SC
DCM
Pearson
dMDC3
MDC3
LG

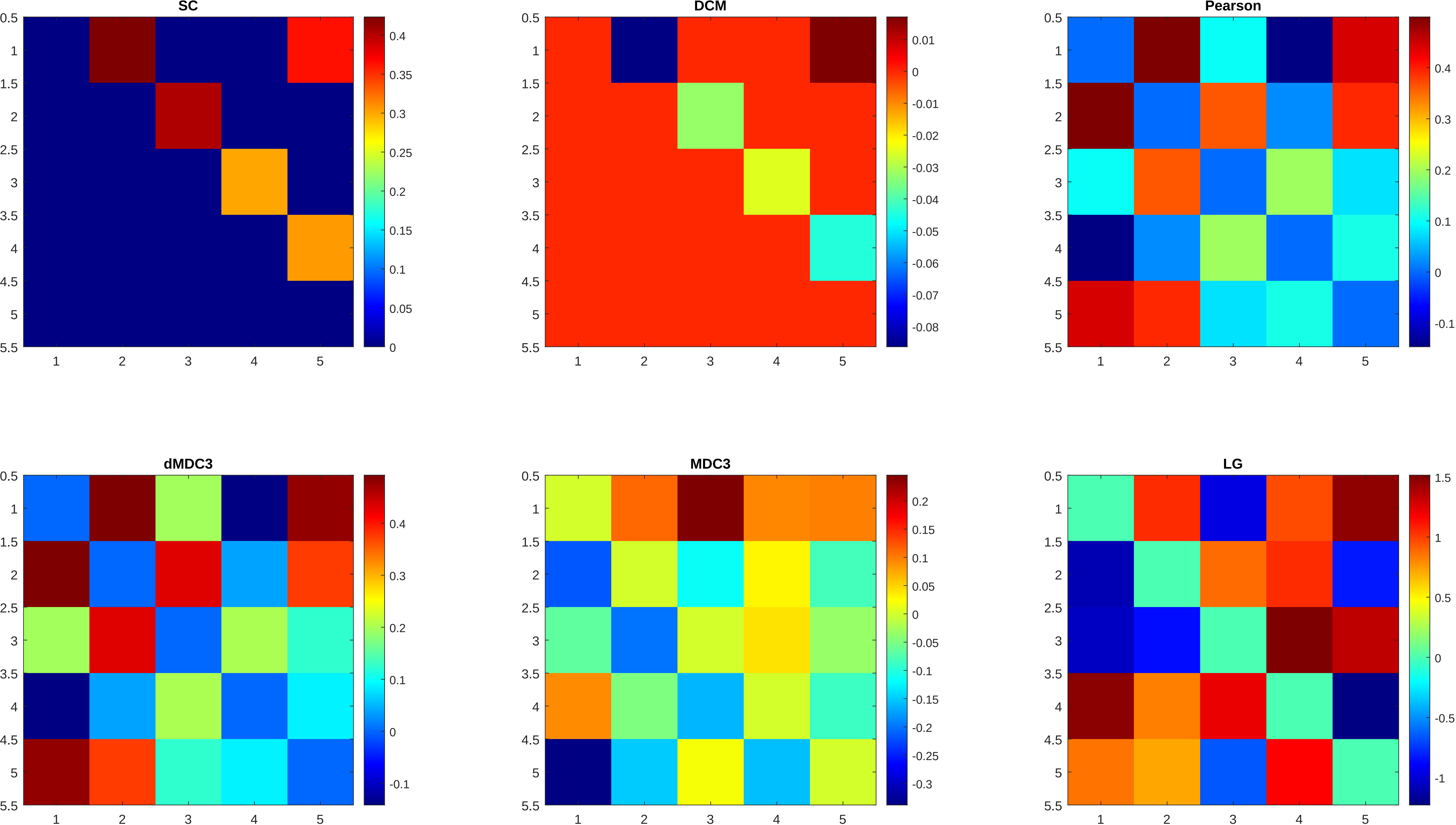
SC
DCM
Pearson
dMDC3
MDC3
LG

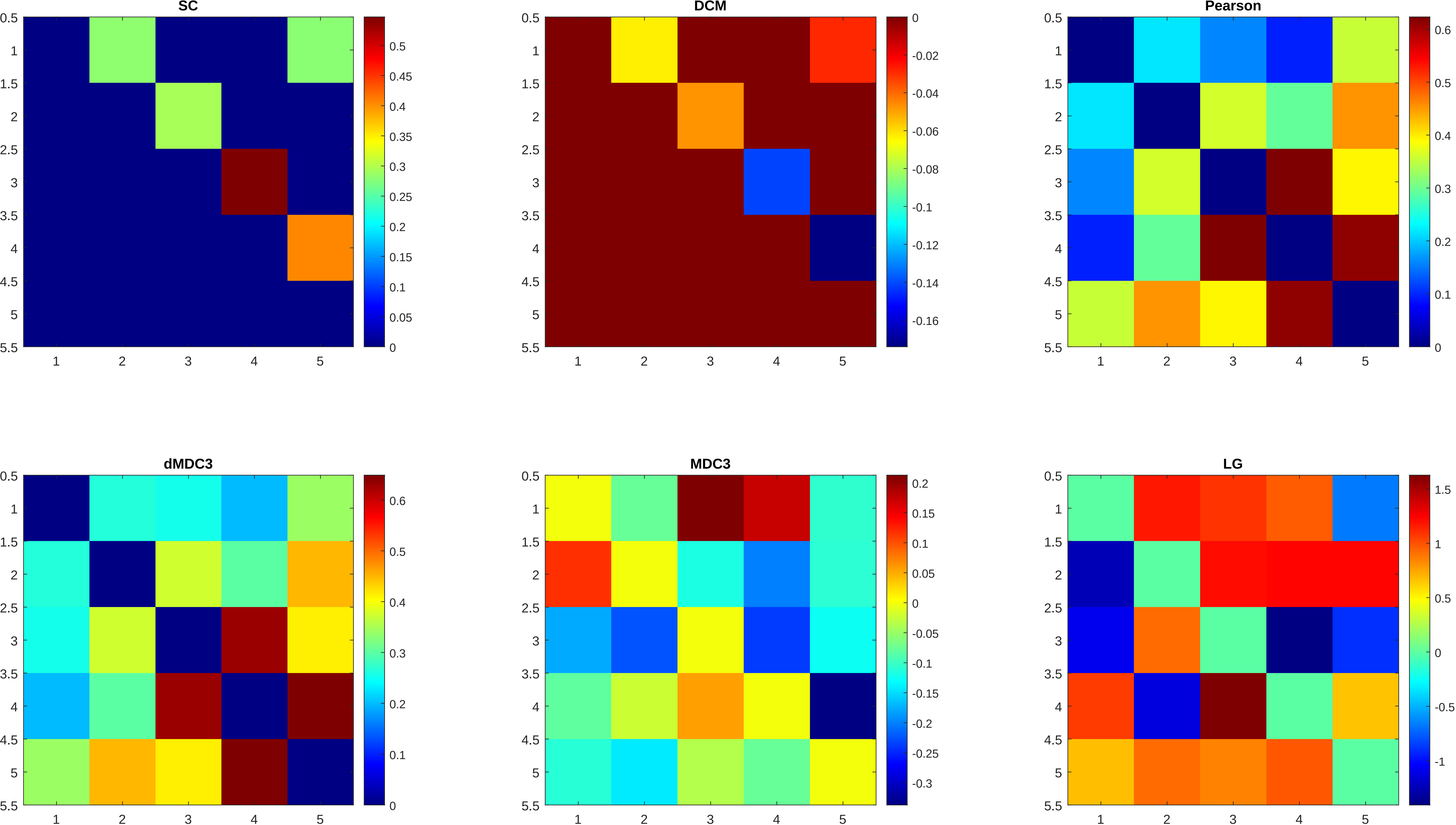
SC
0.5
1
1.5
2
2.5
3
3.5
4
4.5
5
5.5
1 2 3 4 5
0.5 0.45 0.4 0.35 0.3 0.25 0.2 0.15 0.1 0.05 0
DCM
0 -0.02 -0.04 -0.06 -0.08 -0.1 -0.12 -0.14 -0.16
Pearson
0.6 0.5 0.4 0.3 0.2 0.1 0
dMDC3
0.6 0.5 0.4 0.3 0.2 0.1 0
MDC3
0.2 0.15 0.1 0.05 0 -0.05 -0.1 -0.15 -0.2 -0.25 -0.3
LG
1.5 1 0.5 0 -0.5 -1

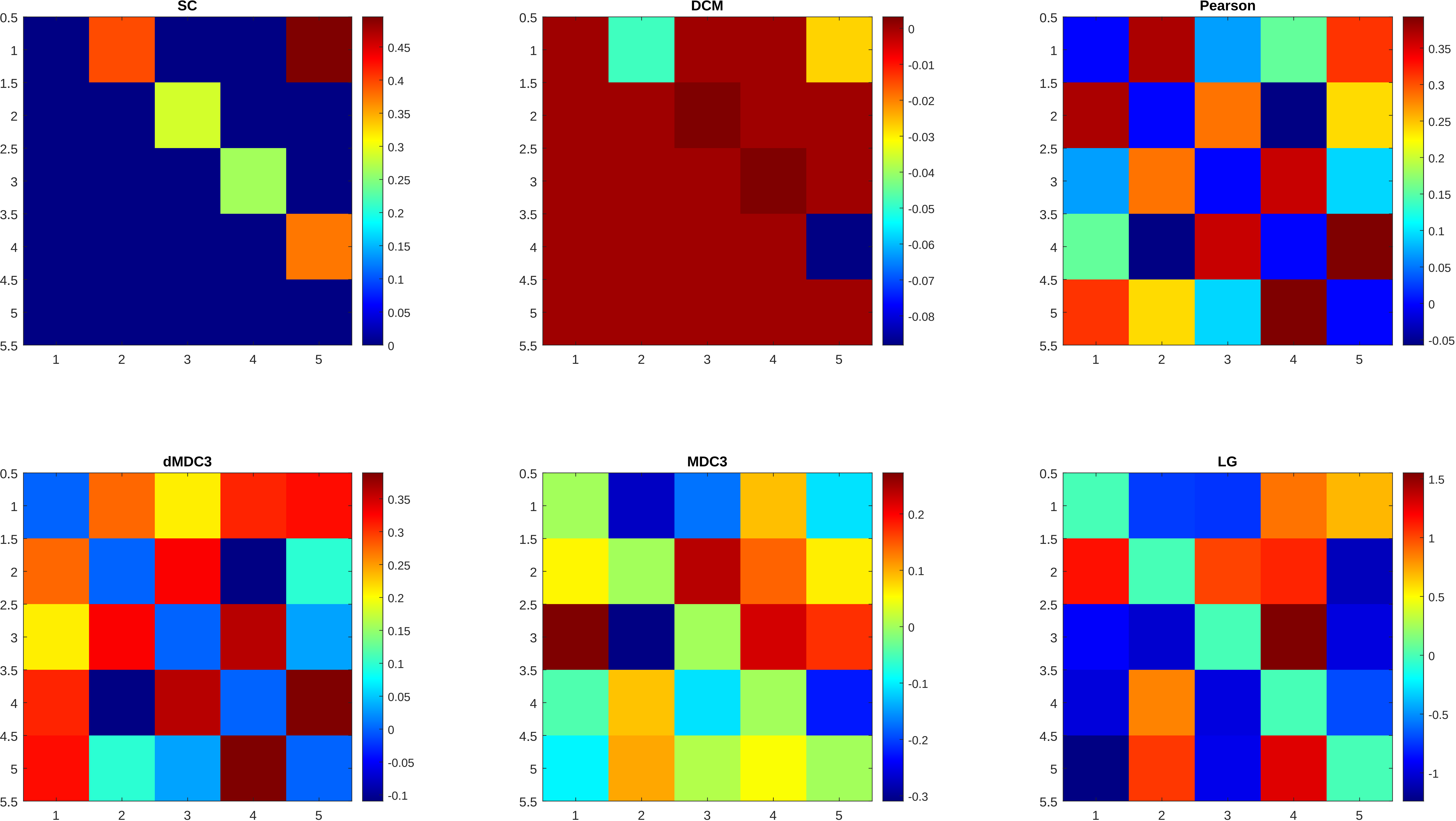
SC
DCM
Pearson
dMDC3
MDC3
LG
0.5
1
1.5
2
2.5
3
3.5
4
4.5
5
5.5
1
2
3
4
5
0.45
0.4
0.35
0.3
0.25
0.2
0.15
0.1
0.05
0
-0.01
-0.02
-0.03
-0.04
-0.05
-0.06
-0.07
-0.08
-0.05
-0.1
-0.2
-0.3
1.5
1
0.5
-0.5
-1

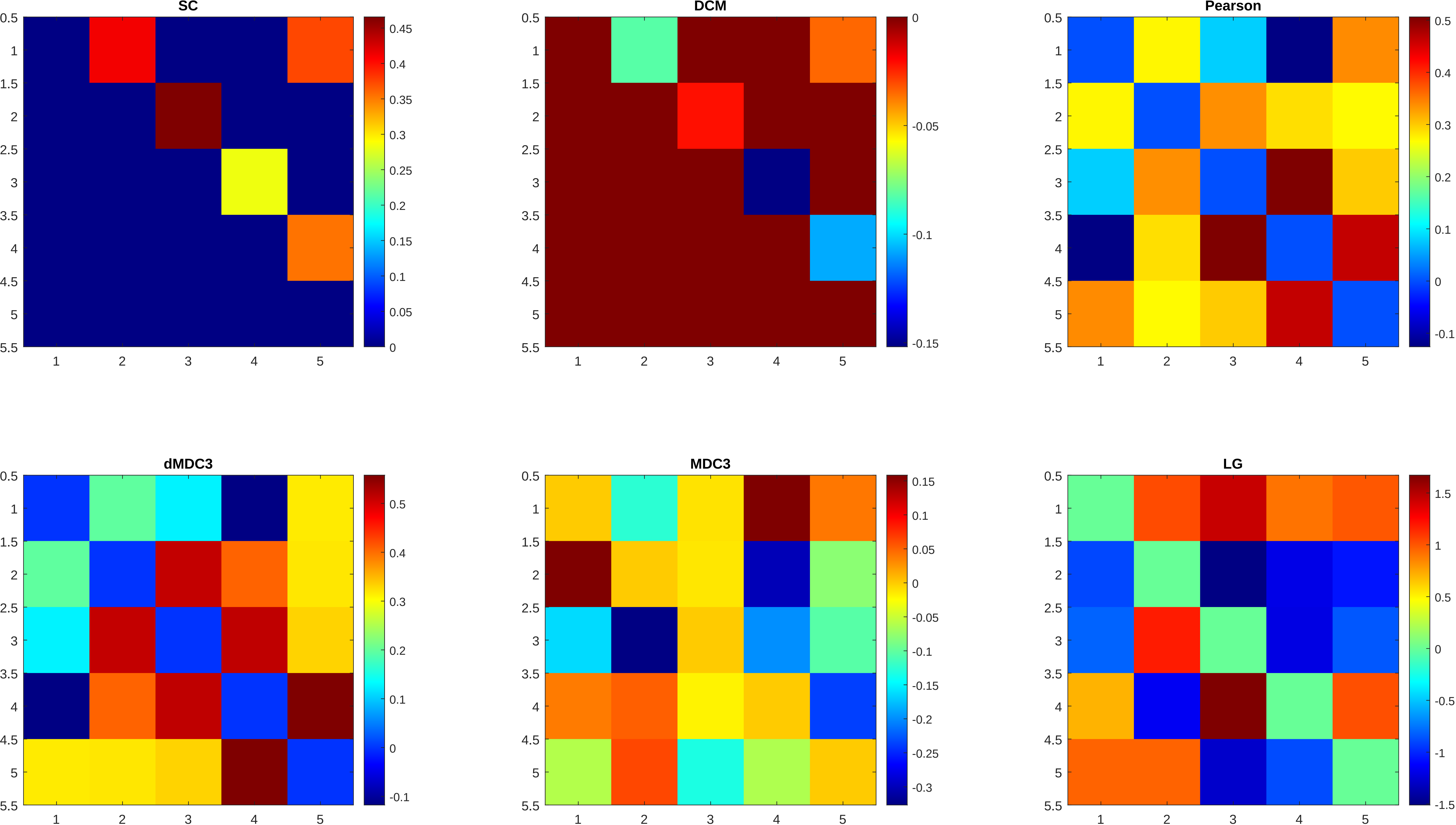
SC
DCM
Pearson
dMDC3
MDC3
LG
