# Supplementary material for: Multiscale detrended cross-correlation coefficient: estimating coupling in non-stationary neurophysiological signals
Source: Front Neurosci. 2024 Nov 13;18:1422085. doi: 10.3389/fnins.2024.1422085 (PMC11599215; doi:10.3389/fnins.2024.1422085)
Supplement: Supplementary file 1 [file Data_Sheet_1.zip › NetSim Matrices.zip!/NetSim Matrices/sim7.pdf]

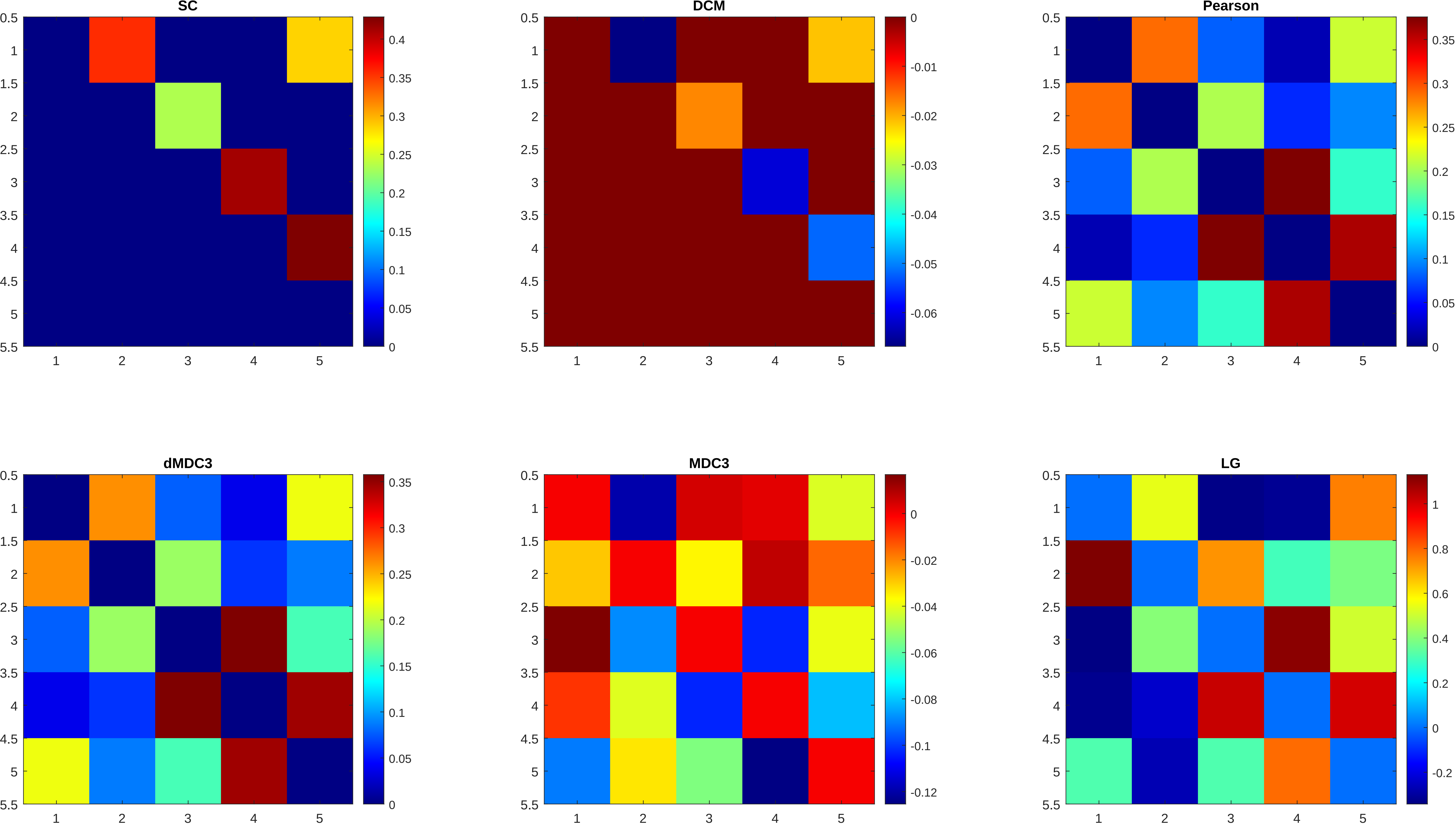
SC
DCM
Pearson
dMDC3
MDC3
LG
0.5
1
1.5
2
2.5
3
3.5
4
4.5
5
5.5
1 2 3 4 5
0 0.05 0.1 0.15 0.2 0.25 0.3 0.35 0.4
0 -0.01 -0.02 -0.03 -0.04 -0.05 -0.06
0 0.05 0.1 0.15 0.2 0.25 0.3 0.35
0 0.05 0.1 0.15 0.2 0.25 0.3 0.35
0 -0.02 -0.04 -0.06 -0.08 -0.1 -0.12
-0.2 0 0.2 0.4 0.6 0.8 1

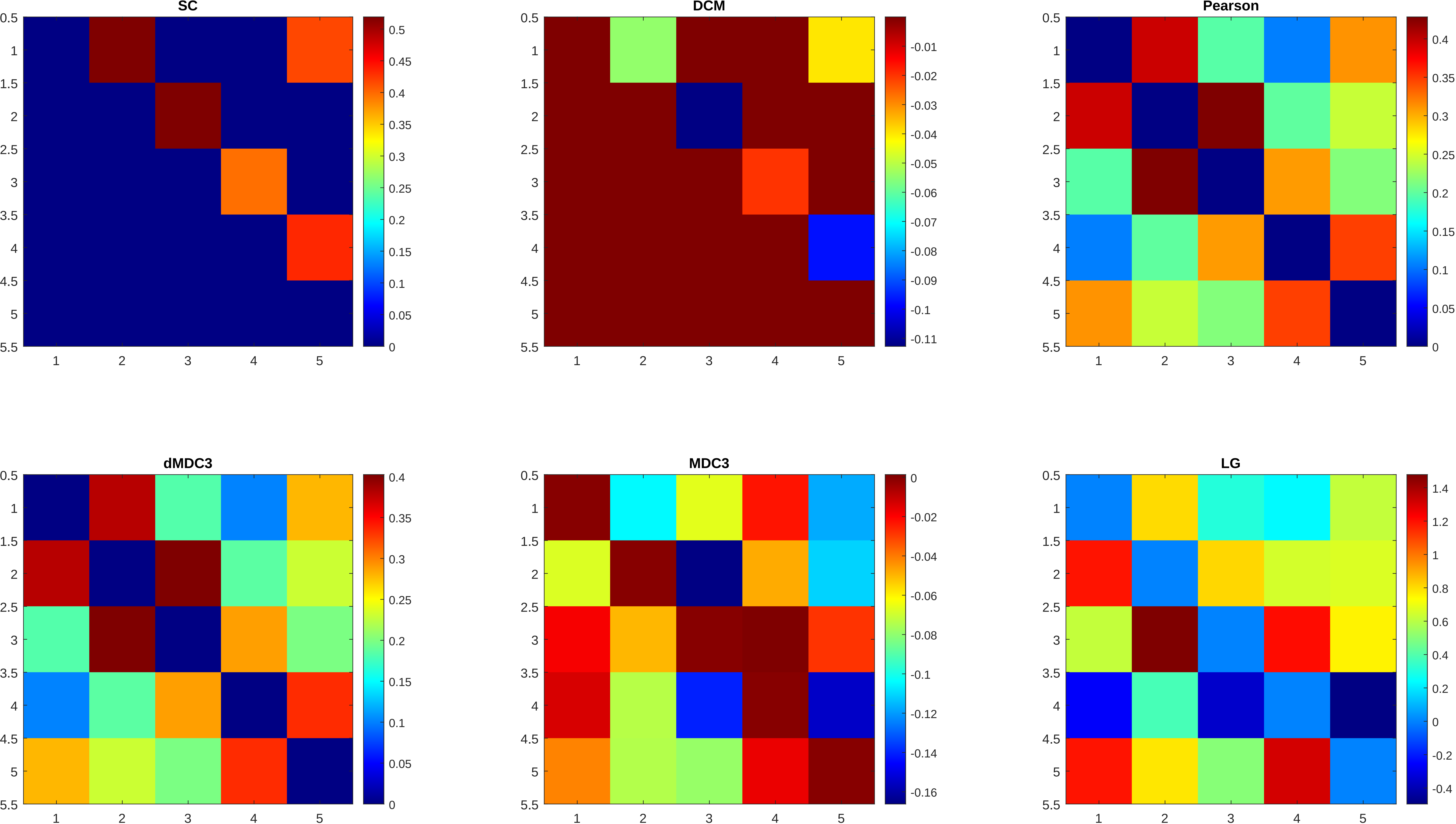
SC
DCM
Pearson
dMDC3
MDC3
LG

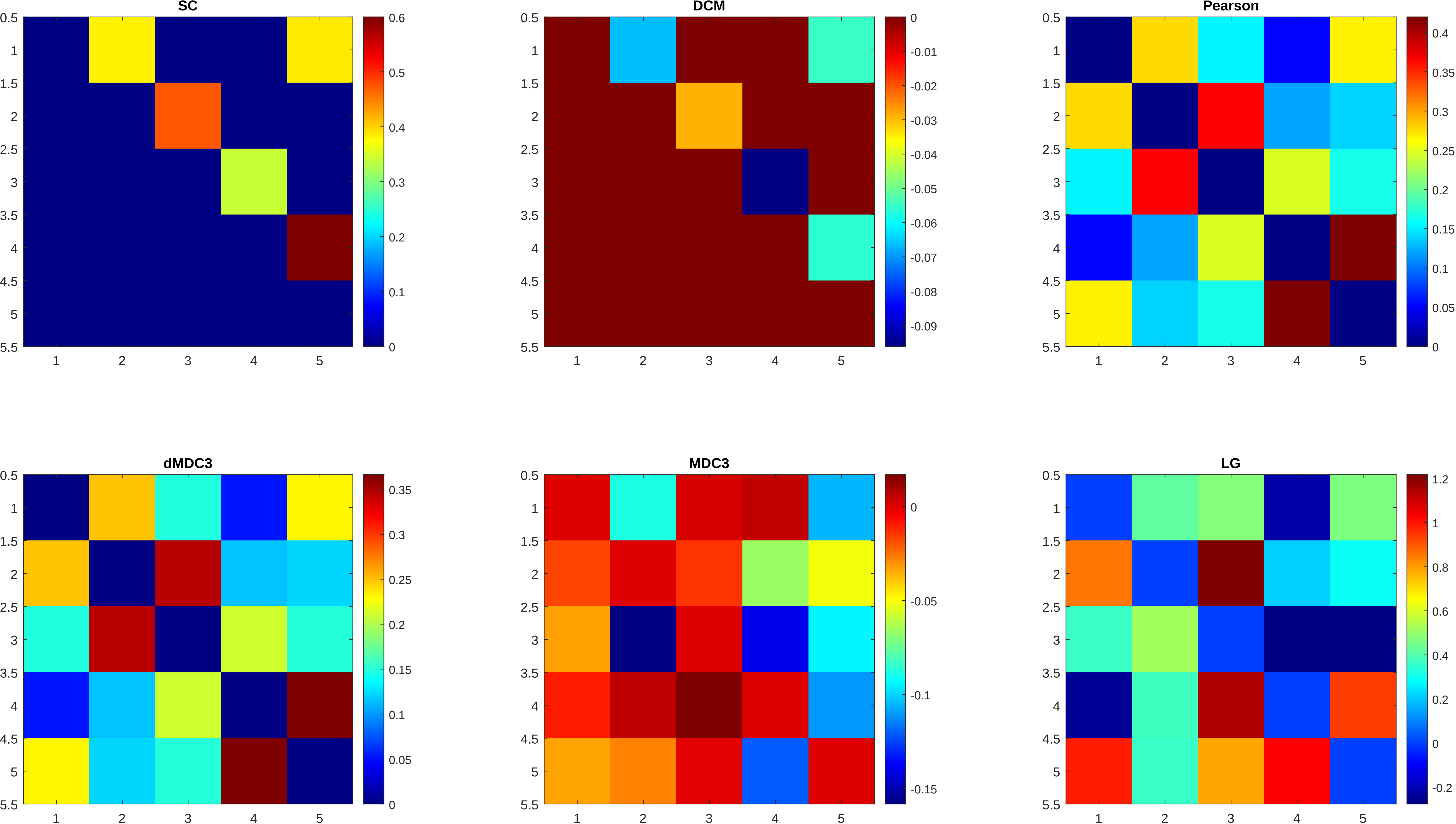
SC
DCM
Pearson
dMDC3
MDC3
LG

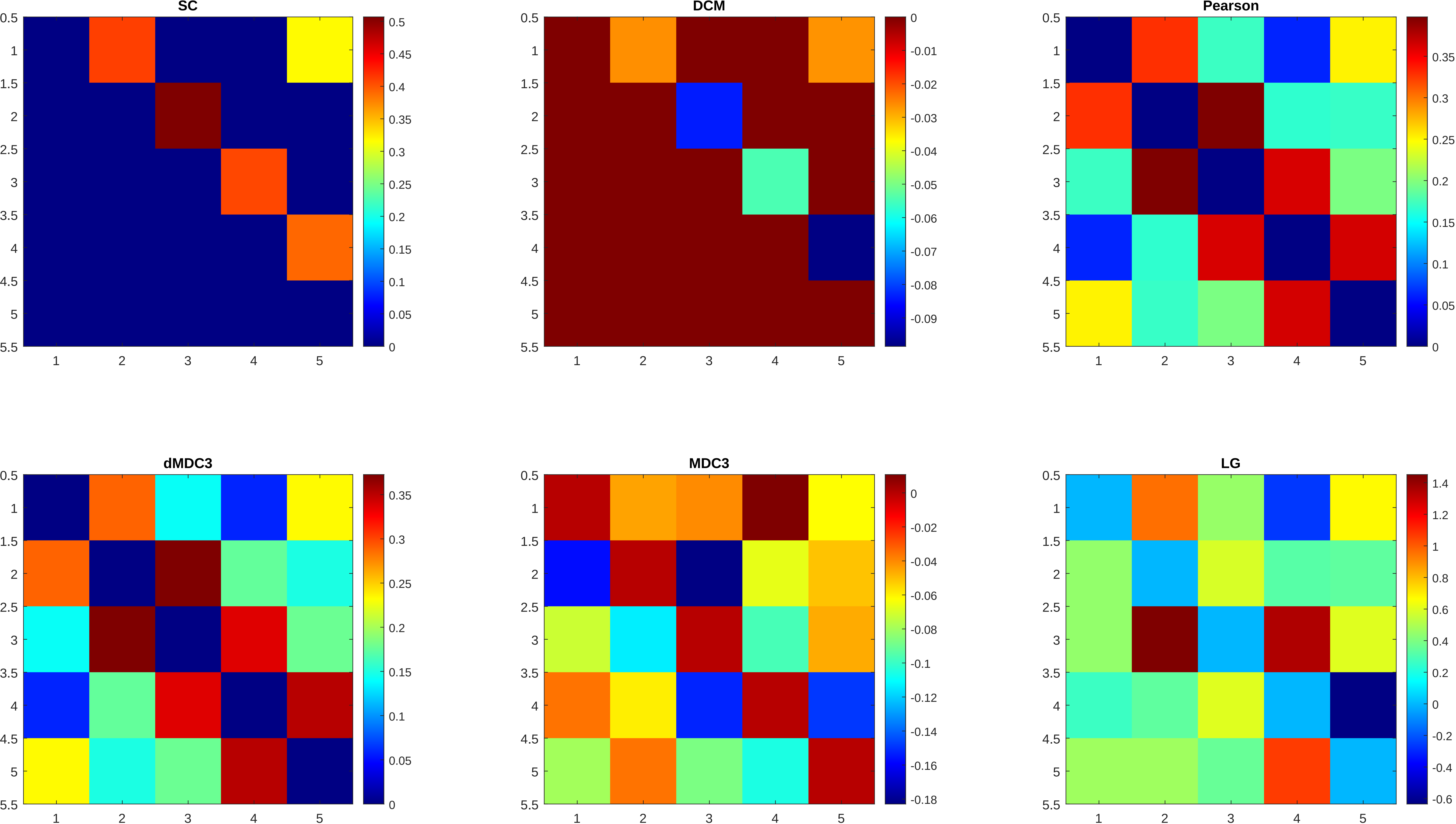
SC
DCM
Pearson
dMDC3
MDC3
LG

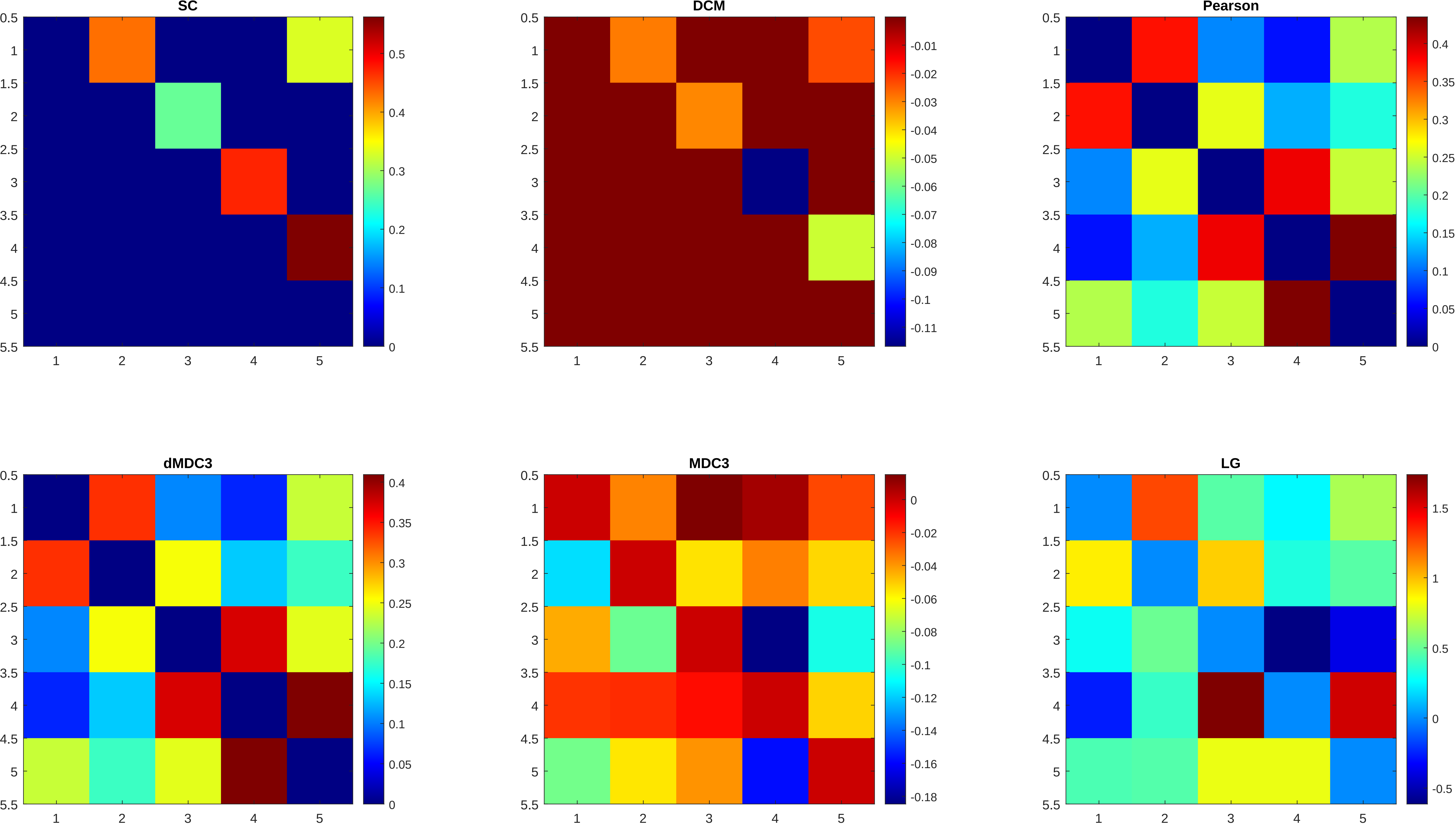

SC
DCM
Pearson
dMDC3
MDC3
LG

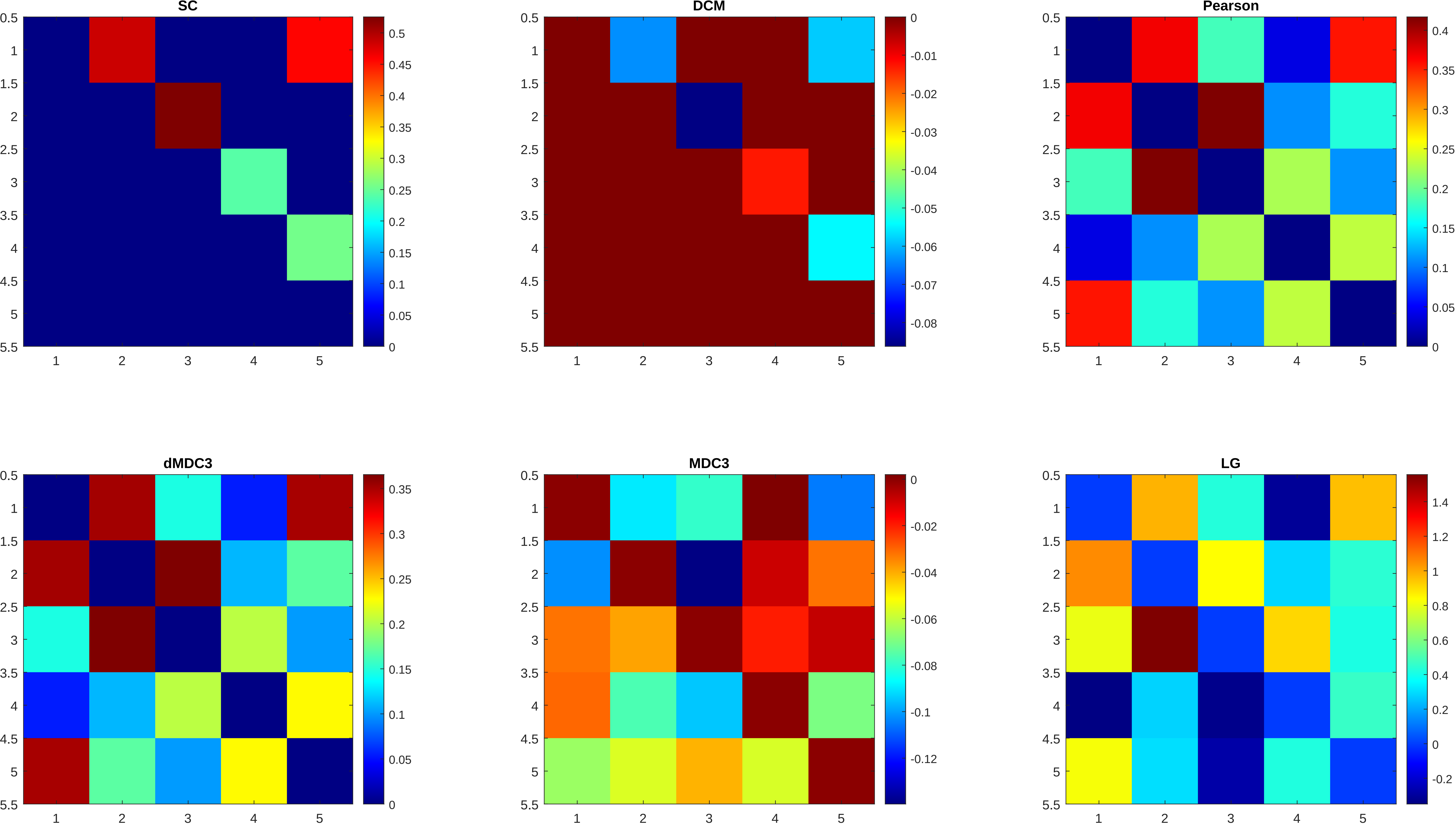

SC
0.5 1 1.5 2 2.5 3 3.5 4 4.5 5 5.5
1 2 3 4 5
0 0.05 0.1 0.15 0.2 0.25 0.3 0.35 0.4 0.45 0.5
DCM
0.5 1 1.5 2 2.5 3 3.5 4 4.5 5 5.5
1 2 3 4 5
0 -0.01 -0.02 -0.03 -0.04 -0.05 -0.06 -0.07 -0.08
Pearson
0.5 1 1.5 2 2.5 3 3.5 4 4.5 5 5.5
1 2 3 4 5
0 0.05 0.1 0.15 0.2 0.25 0.3 0.35 0.4
dMDC3
0.5 1 1.5 2 2.5 3 3.5 4 4.5 5 5.5
1 2 3 4 5
0 0.05 0.1 0.15 0.2 0.25 0.3 0.35
MDC3
0.5 1 1.5 2 2.5 3 3.5 4 4.5 5 5.5
1 2 3 4 5
0 -0.02 -0.04 -0.06 -0.08 -0.1 -0.12
LG
0.5 1 1.5 2 2.5 3 3.5 4 4.5 5 5.5
1 2 3 4 5
-0.2 0 0.2 0.4 0.6 0.8 1 1.2 1.4

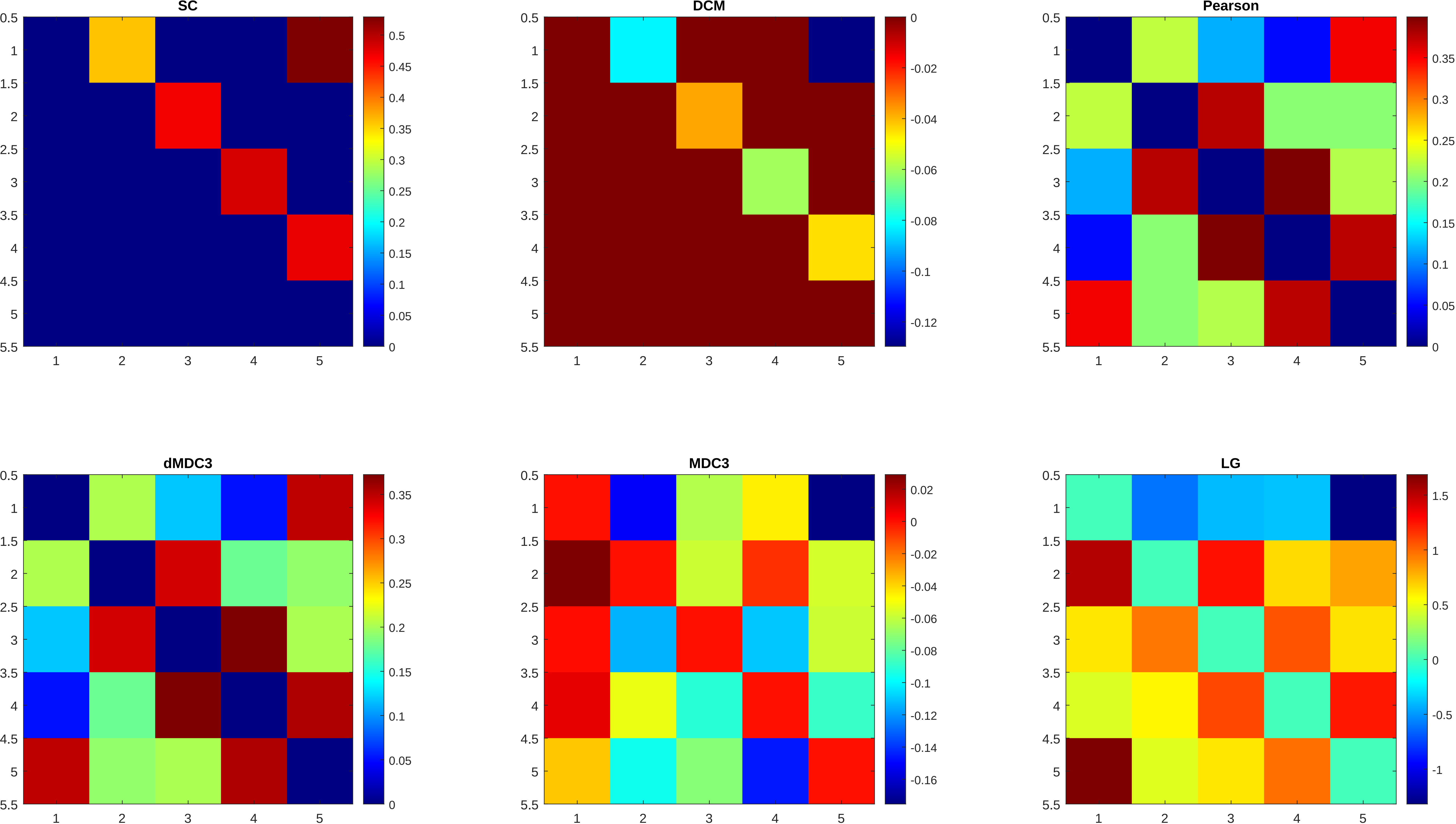
SC
DCM
Pearson
dMDC3
MDC3
LG
0.5
1
1.5
2
2.5
3
3.5
4
4.5
5
5.5
1
2
3
4
5
0
0.05
0.1
0.15
0.2
0.25
0.3
0.35
0.4
0.45
0.5
-0.02
-0.04
-0.06
-0.08
-0.1
-0.12
-0.14
-0.16
0.02
-1
-0.5
1.5

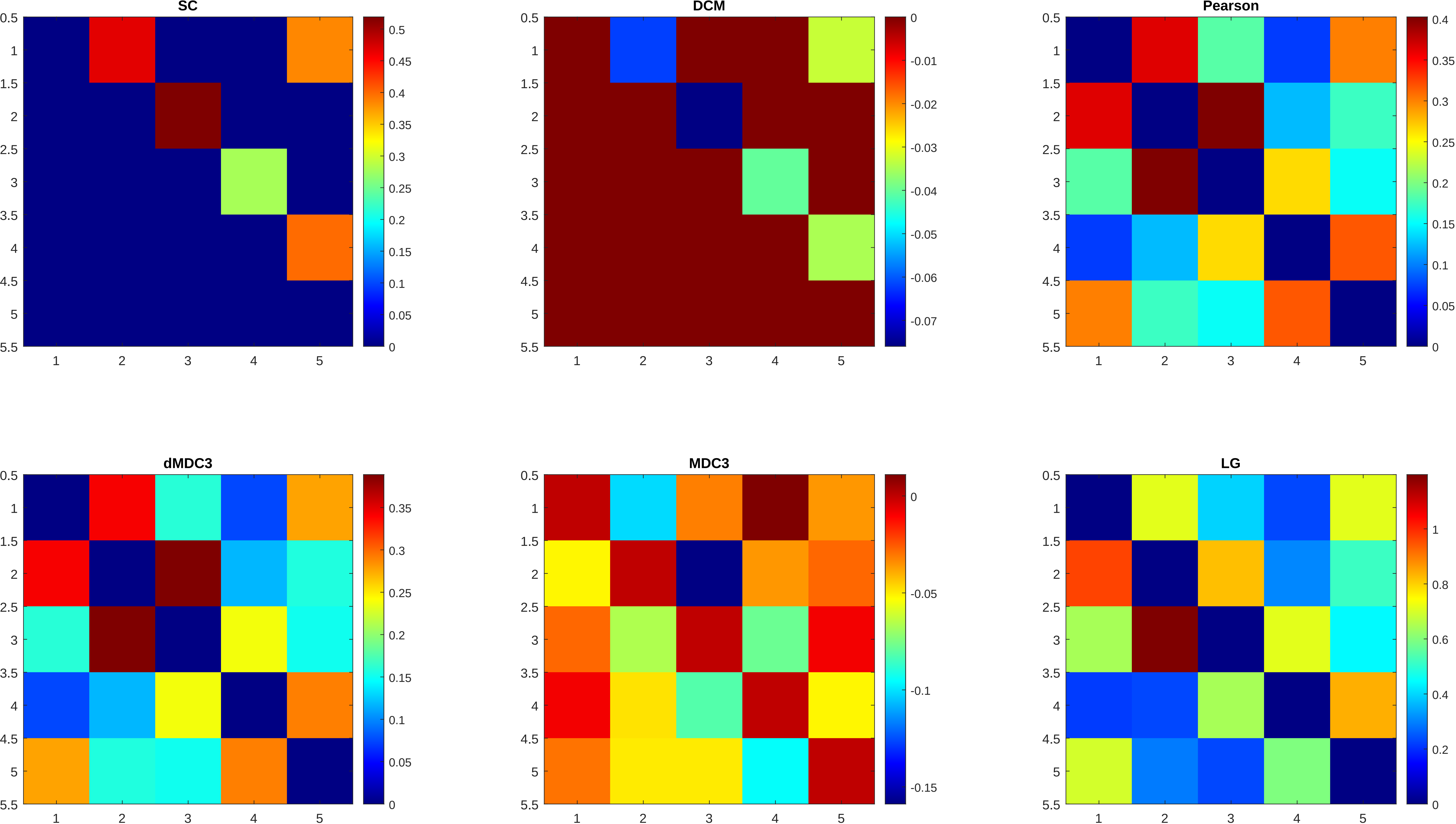
SC
DCM
Pearson
dMDC3
MDC3
LG

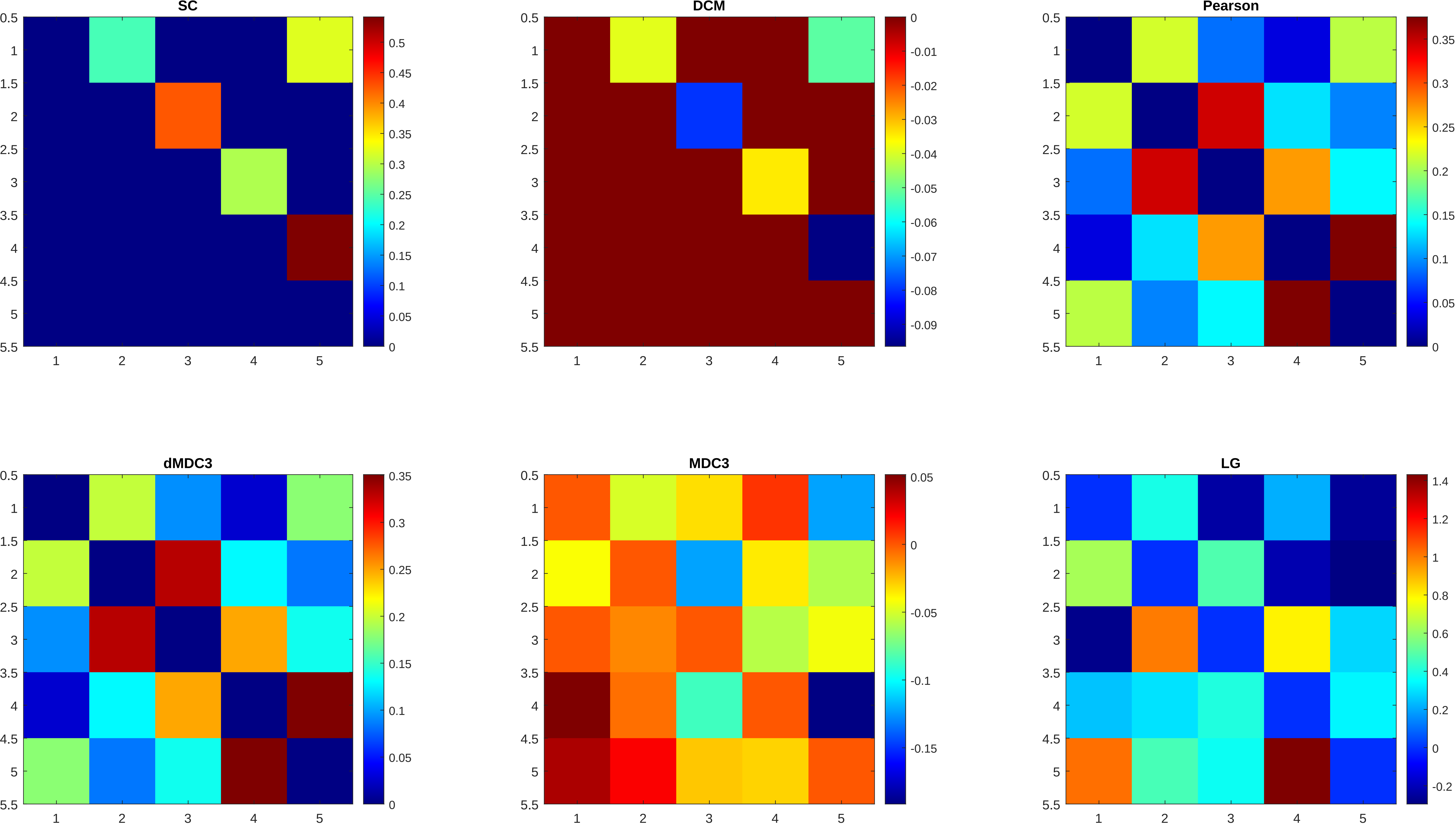

SC
0 0.05 0.1 0.15 0.2 0.25 0.3 0.35 0.4 0.45 0.5
DCM
0 -0.01 -0.02 -0.03 -0.04 -0.05 -0.06 -0.07 -0.08 -0.09
Pearson
0 0.05 0.1 0.15 0.2 0.25 0.3 0.35
dMDC3
0 0.05 0.1 0.15 0.2 0.25 0.3 0.35
MDC3
0.05 0 -0.05 -0.1 -0.15
LG
1.4 1.2 1 0.8 0.6 0.4 0.2 0 -0.2

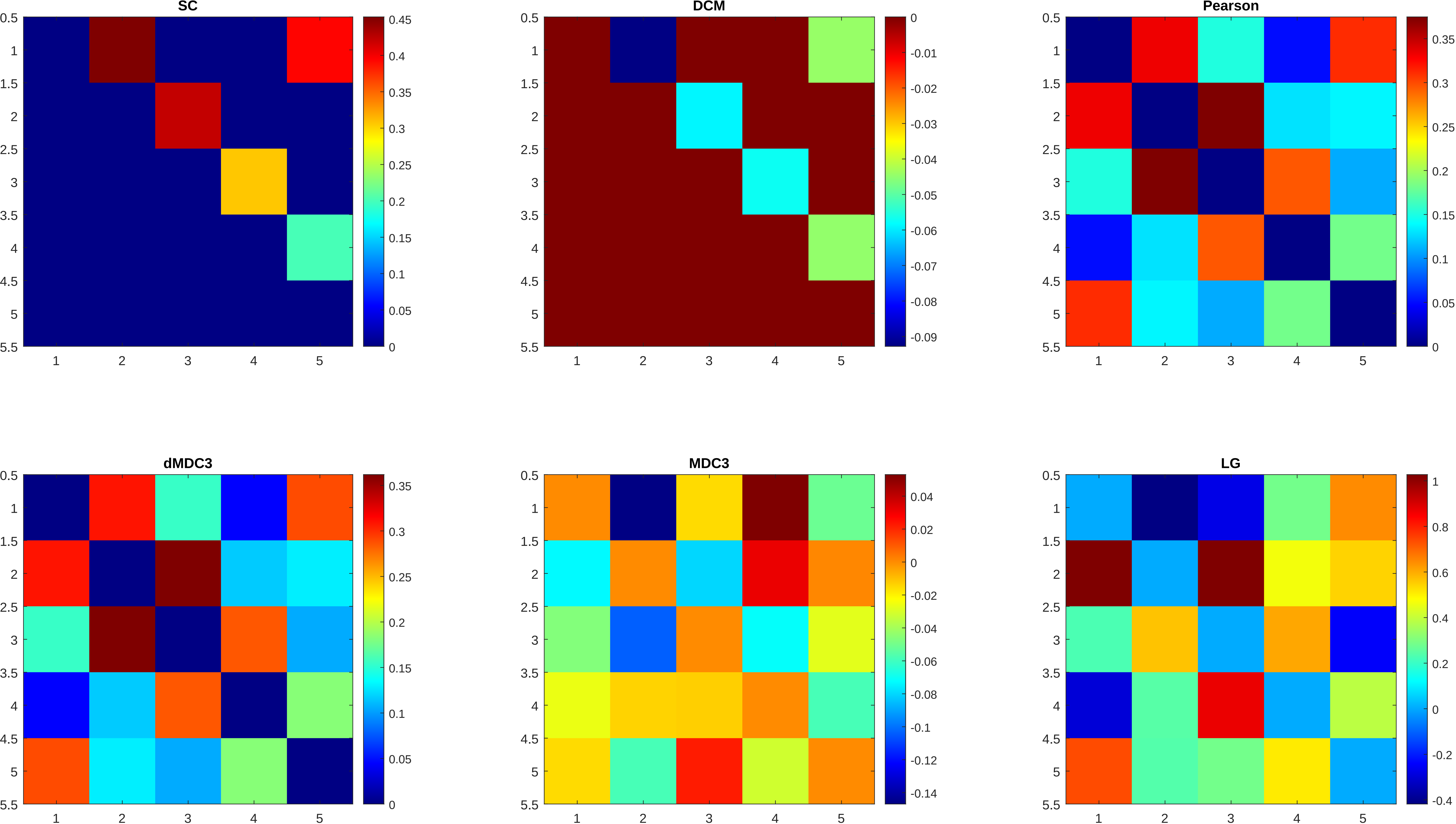
SC
DCM
Pearson
dMDC3
MDC3
LG

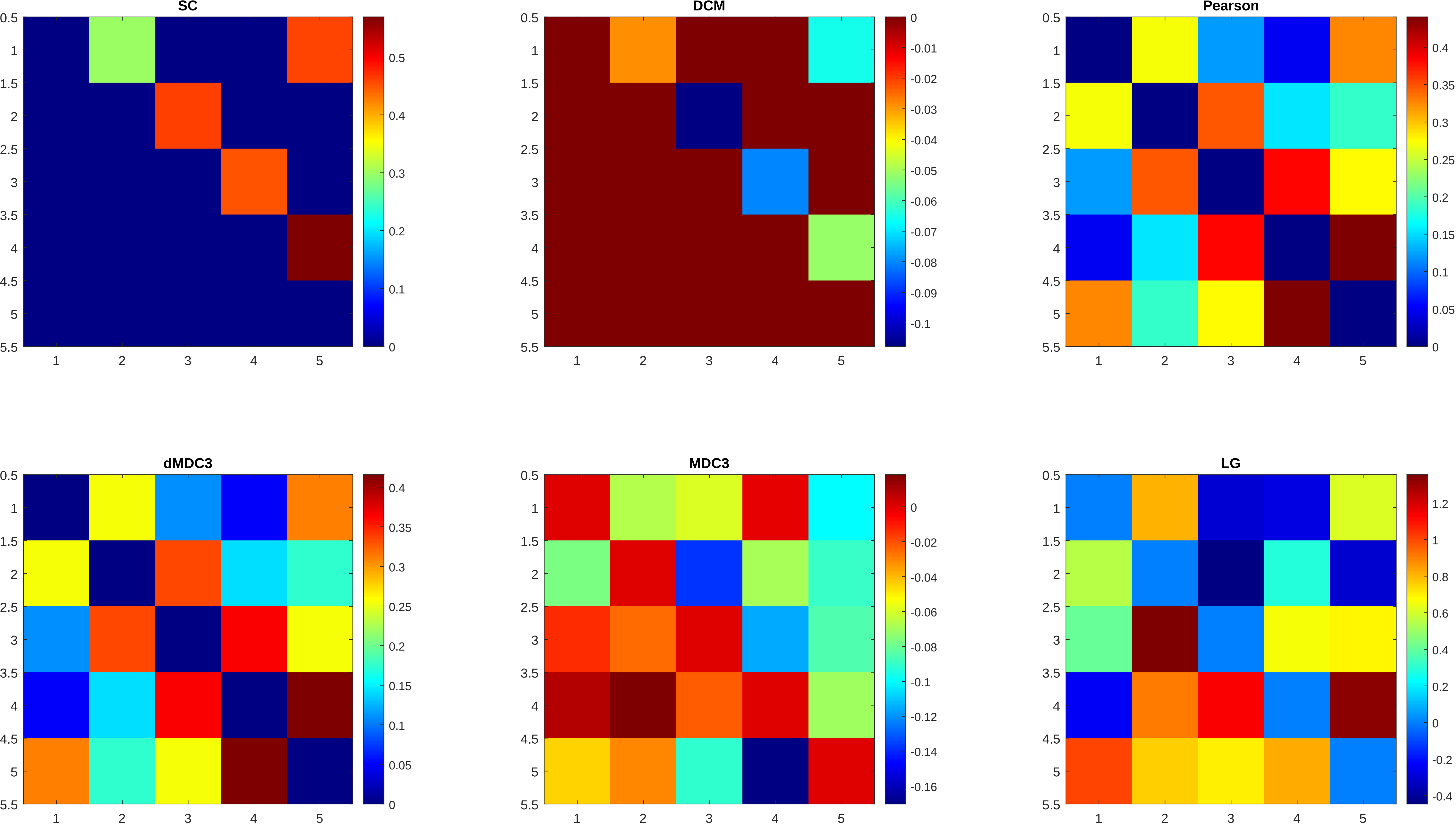
SC
DCM
Pearson
dMDC3
MDC3
LG
0.5 1 1.5 2 2.5 3 3.5 4 4.5 5 5.5
1 2 3 4 5
0 0.1 0.2 0.3 0.4 0.5
0 -0.01 -0.02 -0.03 -0.04 -0.05 -0.06 -0.07 -0.08 -0.09 -0.1
0 0.05 0.1 0.15 0.2 0.25 0.3 0.35 0.4
0 0.05 0.1 0.15 0.2 0.25 0.3 0.35 0.4
0 -0.02 -0.04 -0.06 -0.08 -0.1 -0.12 -0.14 -0.16
-0.4 -0.2 0 0.2 0.4 0.6 0.8 1 1.2

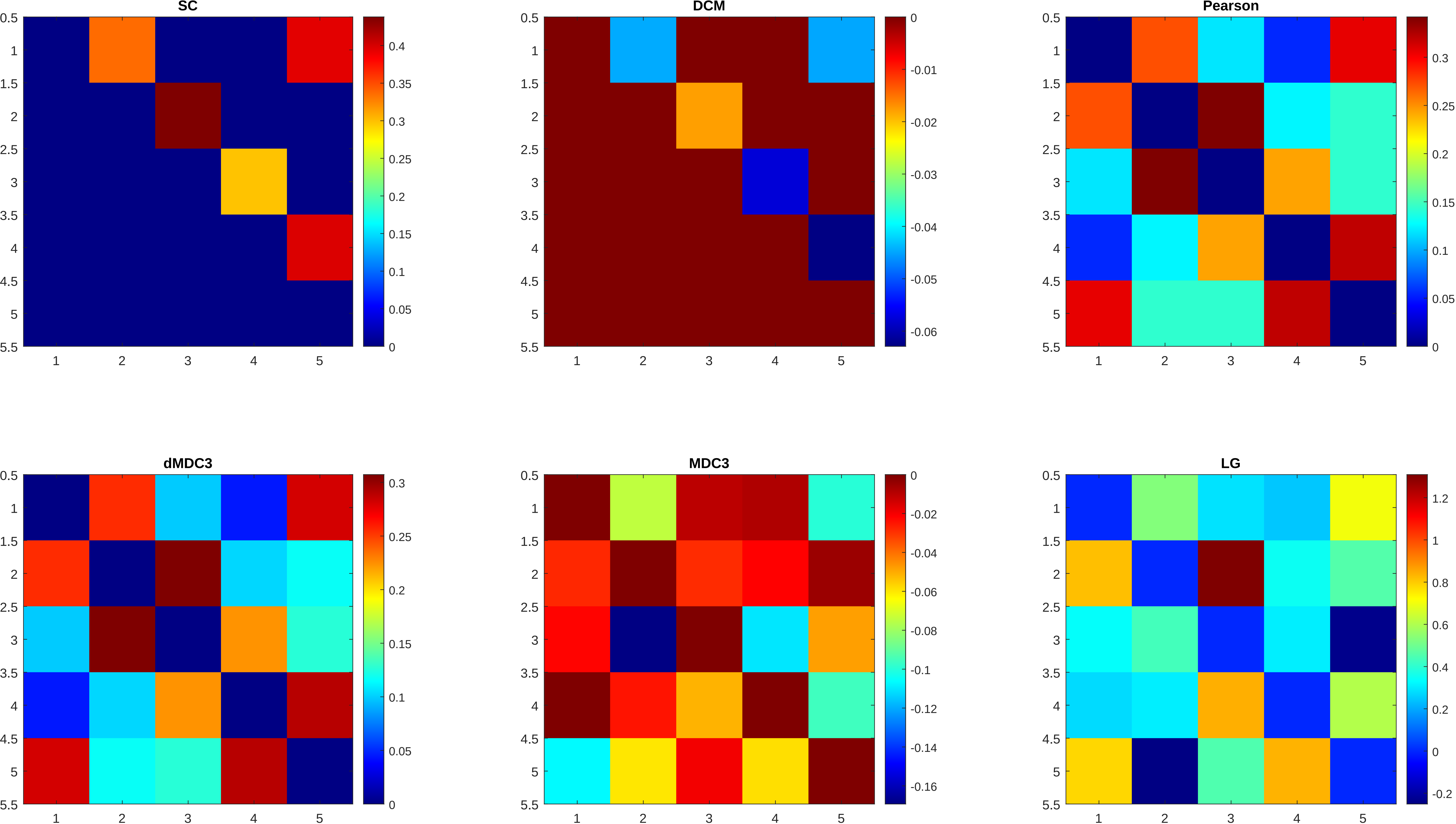
SC
0.5 1 1.5 2 2.5 3 3.5 4 4.5 5 5.5
1 2 3 4 5
0 0.05 0.1 0.15 0.2 0.25 0.3 0.35 0.4
DCM
0.5 1 1.5 2 2.5 3 3.5 4 4.5 5 5.5
1 2 3 4 5
0 -0.01 -0.02 -0.03 -0.04 -0.05 -0.06
Pearson
0.5 1 1.5 2 2.5 3 3.5 4 4.5 5 5.5
1 2 3 4 5
0 0.05 0.1 0.15 0.2 0.25 0.3
dMDC3
0.5 1 1.5 2 2.5 3 3.5 4 4.5 5 5.5
1 2 3 4 5
0 0.05 0.1 0.15 0.2 0.25 0.3
MDC3
0.5 1 1.5 2 2.5 3 3.5 4 4.5 5 5.5
1 2 3 4 5
0 -0.02 -0.04 -0.06 -0.08 -0.1 -0.12 -0.14 -0.16
LG
0.5 1 1.5 2 2.5 3 3.5 4 4.5 5 5.5
1 2 3 4 5
-0.2 0 0.2 0.4 0.6 0.8 1 1.2

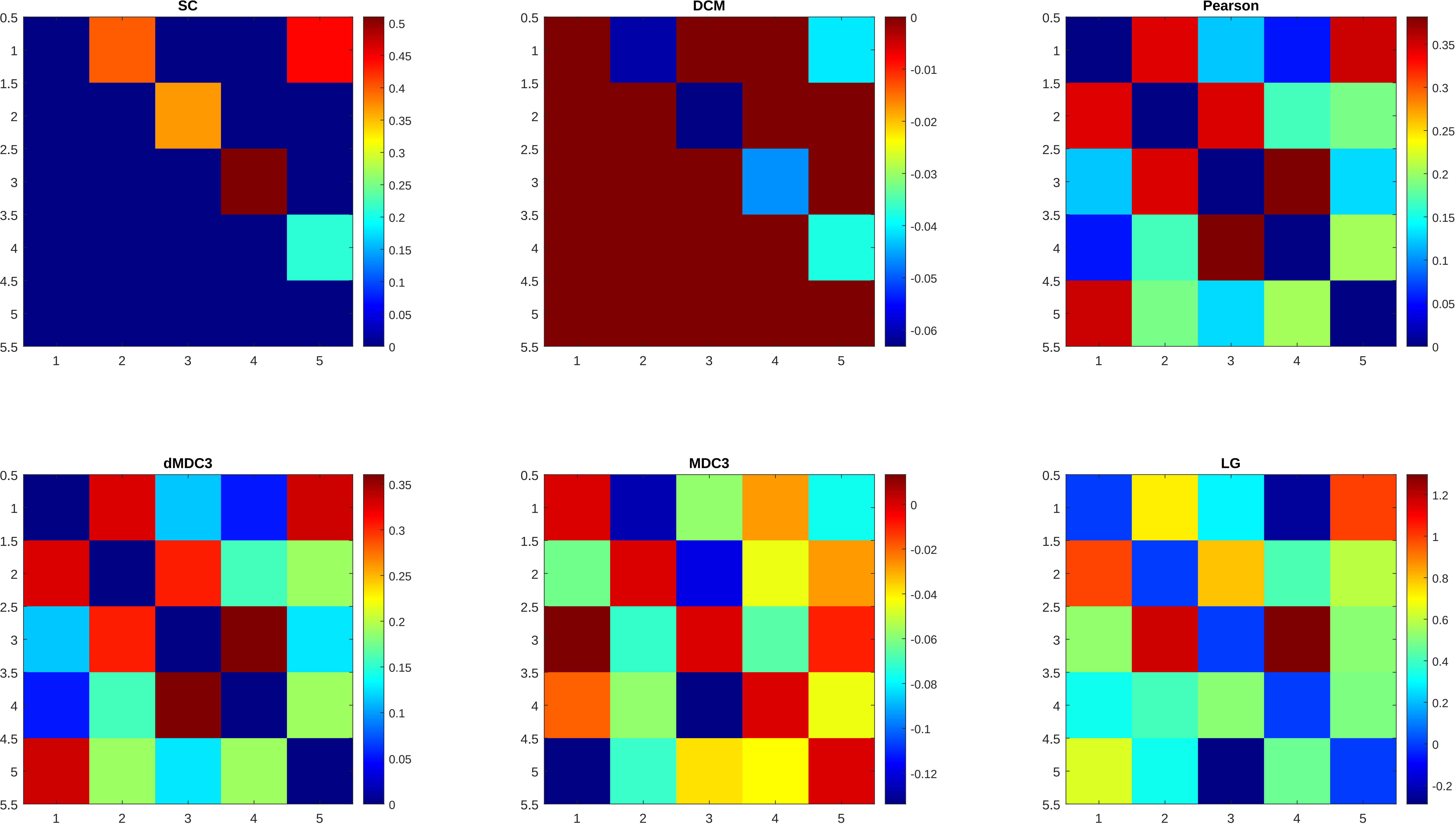
SC
DCM
Pearson
dMDC3
MDC3
LG
0.5
1
1.5
2
2.5
3
3.5
4
4.5
5
5.5
1
2
3
4
5

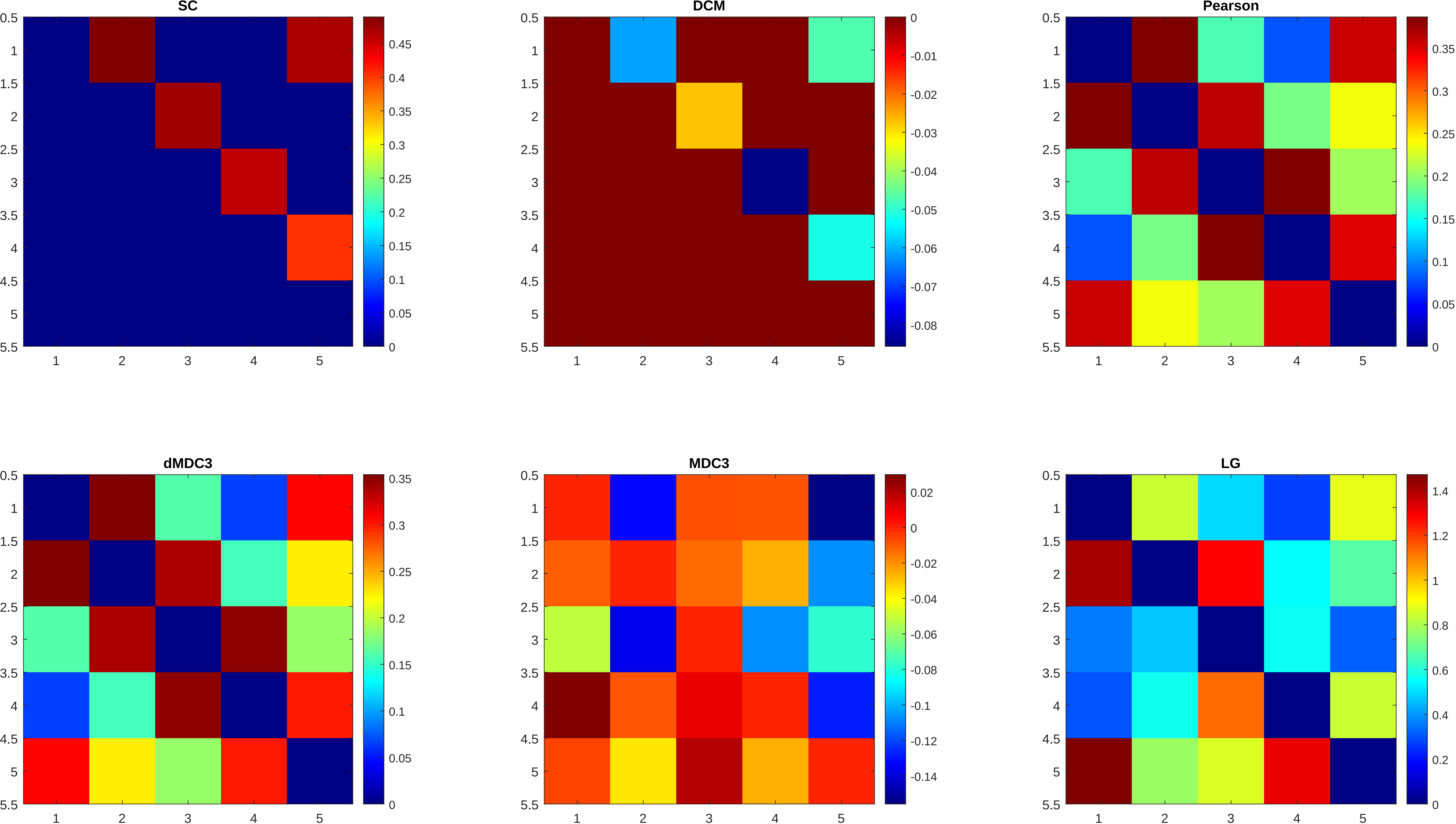
SC
DCM
Pearson
dMDC3
MDC3
LG
0.5
1
1.5
2
2.5
3
3.5
4
4.5
5
5.5
1
2
3
4
5
0
0.05
0.1
0.15
0.2
0.25
0.3
0.35
0.4
0.45
0
-0.01
-0.02
-0.03
-0.04
-0.05
-0.06
-0.07
-0.08
0.02
-0.1
-0.12
-0.14
0.2
0.4
0.6
0.8
1
1.2
1.4

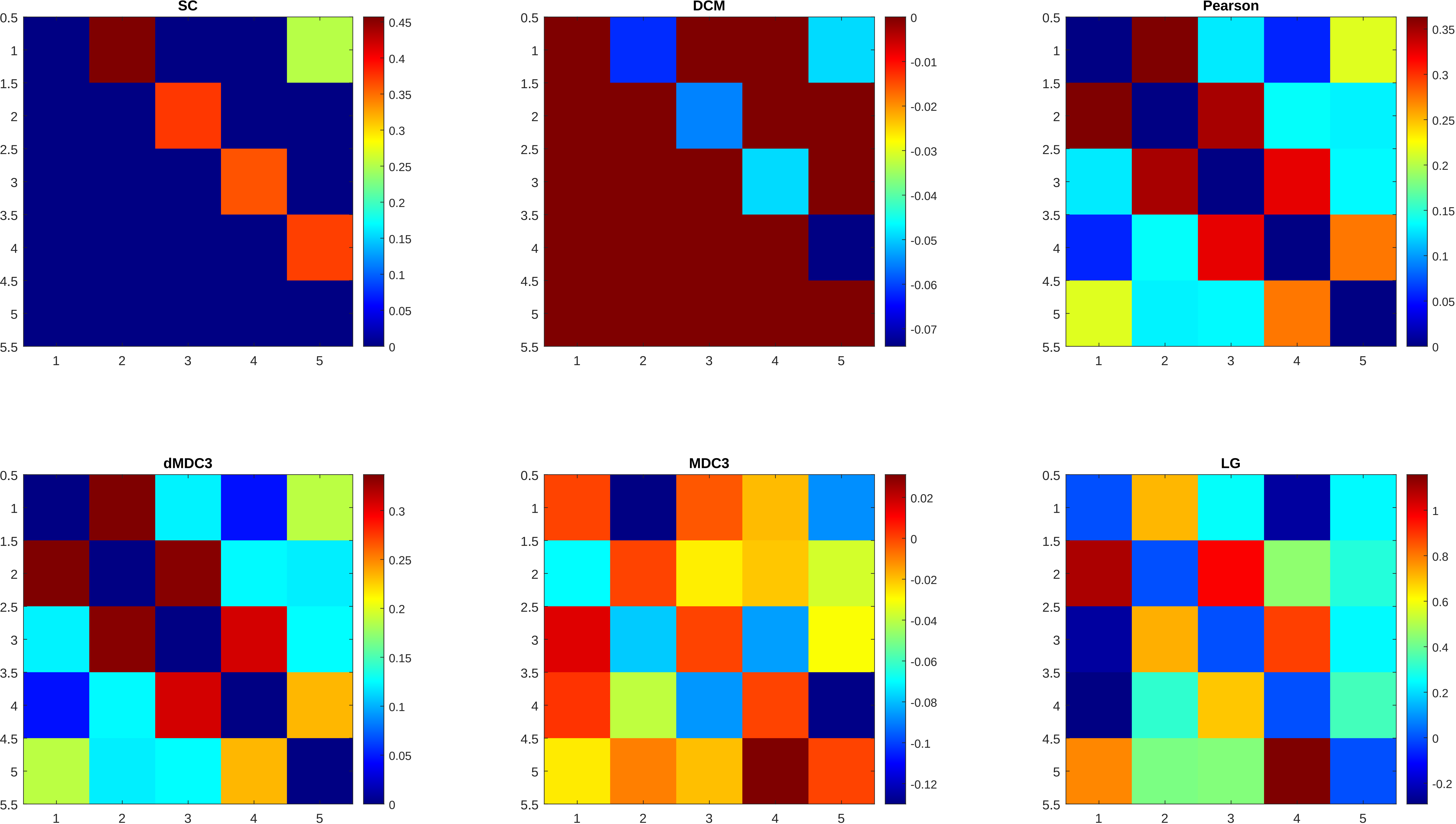
SC
DCM
Pearson
dMDC3
MDC3
LG
0.5 1 1.5 2 2.5 3 3.5 4 4.5 5 5.5
1 2 3 4 5
0 0.05 0.1 0.15 0.2 0.25 0.3 0.35 0.4 0.45
0 -0.01 -0.02 -0.03 -0.04 -0.05 -0.06 -0.07
0 0.05 0.1 0.15 0.2 0.25 0.3 0.35
0 0.05 0.1 0.15 0.2 0.25 0.3
0.02 0 -0.02 -0.04 -0.06 -0.08 -0.1 -0.12
1 0.8 0.6 0.4 0.2 0 -0.2

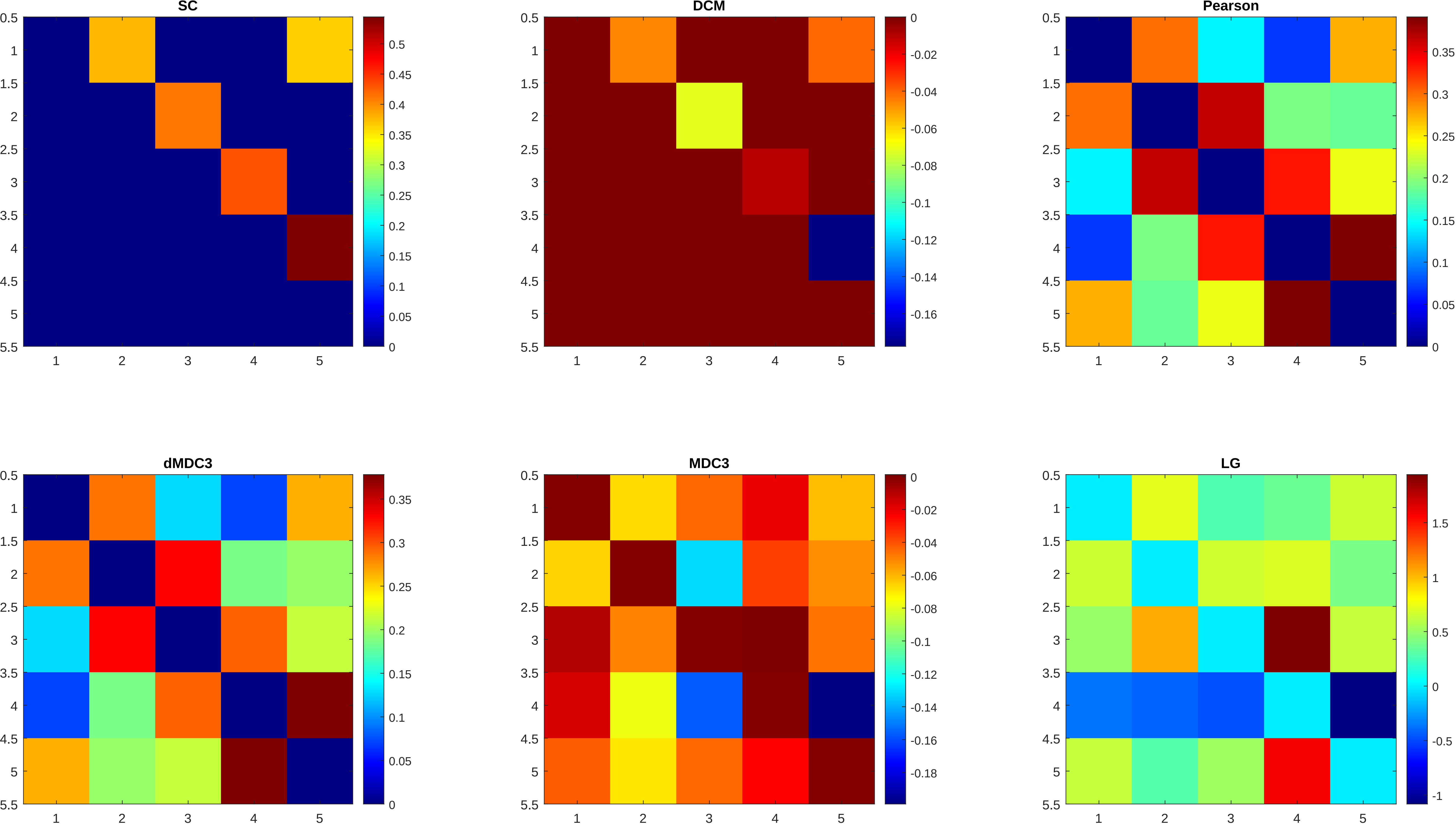
SC
DCM
Pearson
dMDC3
MDC3
LG

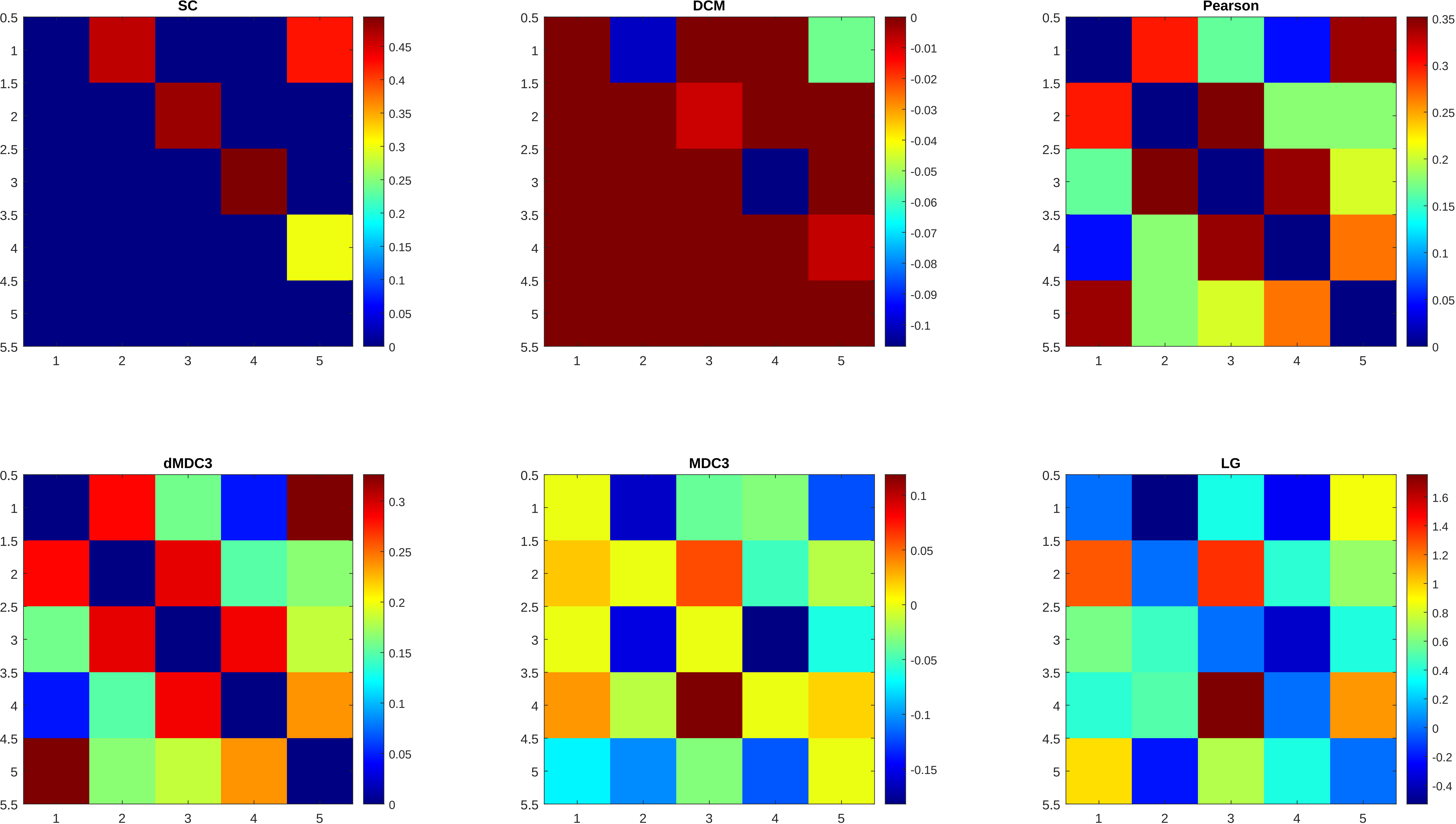
SC
0.5 1 1.5 2 2.5 3 3.5 4 4.5 5 5.5
1 2 3 4 5
0 0.05 0.1 0.15 0.2 0.25 0.3 0.35 0.4 0.45
DCM
0.5 1 1.5 2 2.5 3 3.5 4 4.5 5 5.5
1 2 3 4 5
0 -0.01 -0.02 -0.03 -0.04 -0.05 -0.06 -0.07 -0.08 -0.09 -0.1
Pearson
0.5 1 1.5 2 2.5 3 3.5 4 4.5 5 5.5
1 2 3 4 5
0.35 0.3 0.25 0.2 0.15 0.1 0.05 0
dMDC3
0.5 1 1.5 2 2.5 3 3.5 4 4.5 5 5.5
1 2 3 4 5
0.3 0.25 0.2 0.15 0.1 0.05 0
MDC3
0.5 1 1.5 2 2.5 3 3.5 4 4.5 5 5.5
1 2 3 4 5
0.1 0.05 0 -0.05 -0.1 -0.15
LG
0.5 1 1.5 2 2.5 3 3.5 4 4.5 5 5.5
1 2 3 4 5
1.6 1.4 1.2 1 0.8 0.6 0.4 0.2 0 -0.2 -0.4

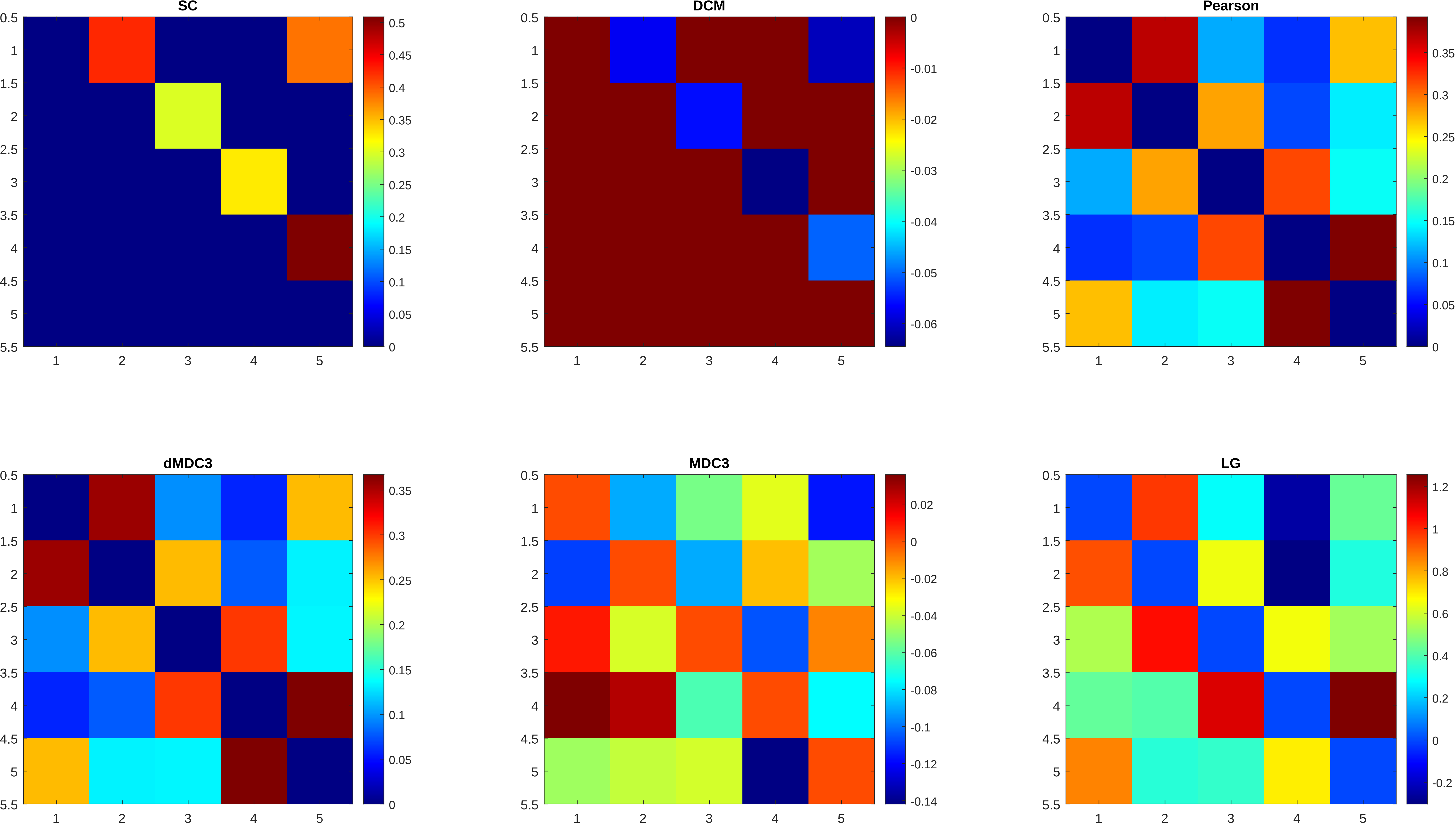

SC
DCM
Pearson
dMDC3
MDC3
LG

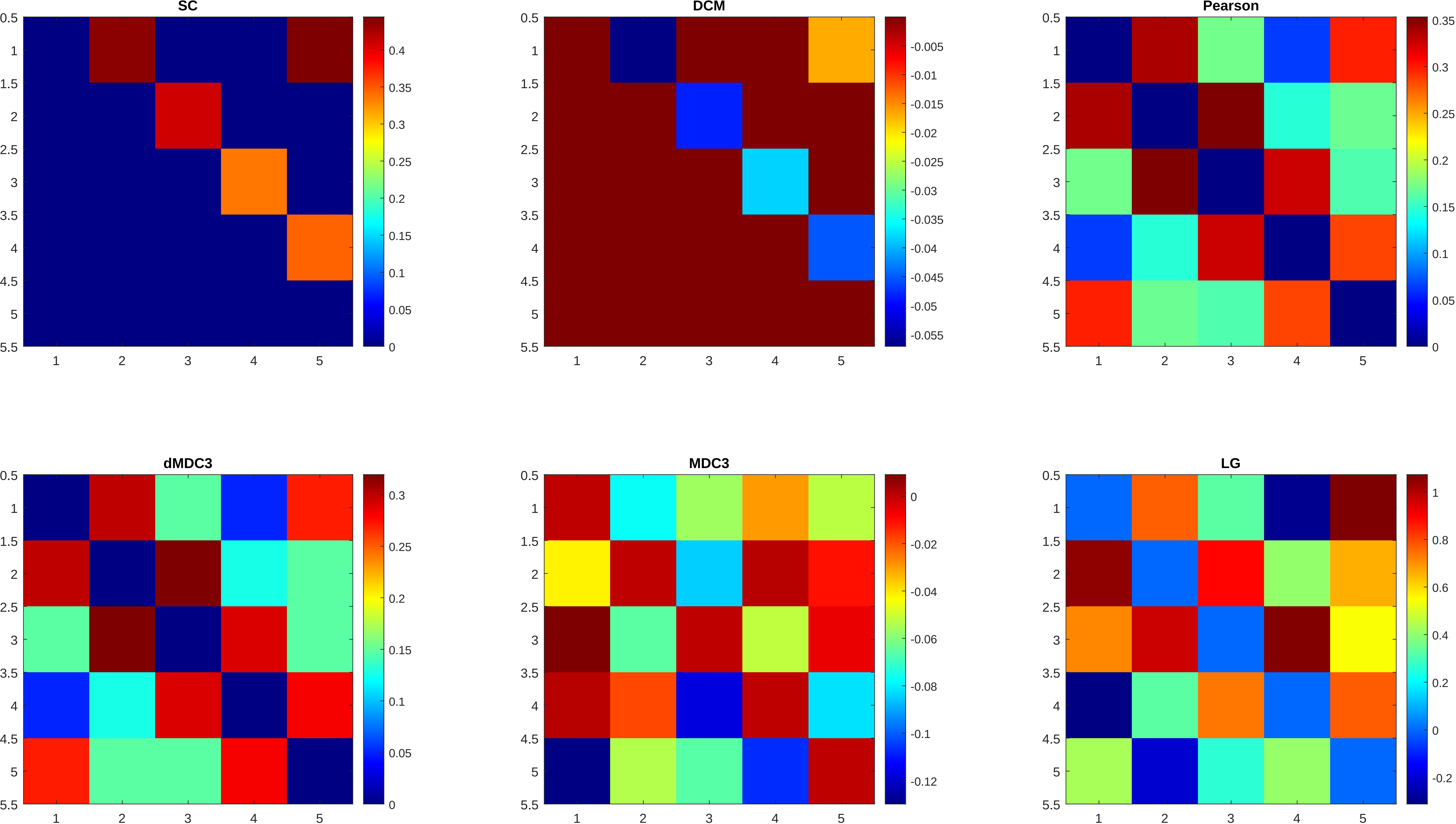
SC
DCM
Pearson
dMDC3
MDC3
LG
0.5
1
1.5
2
2.5
3
3.5
4
4.5
5
5.5
1
2
3
4
5
0
0.05
0.1
0.15
0.2
0.25
0.3
0.35
0.4
-0.005
-0.01
-0.015
-0.02
-0.025
-0.03
-0.035
-0.04
-0.045
-0.05
-0.055
-0.02
-0.04
-0.06
-0.08
-0.1
-0.12
0.6
0.8
1
-0.2

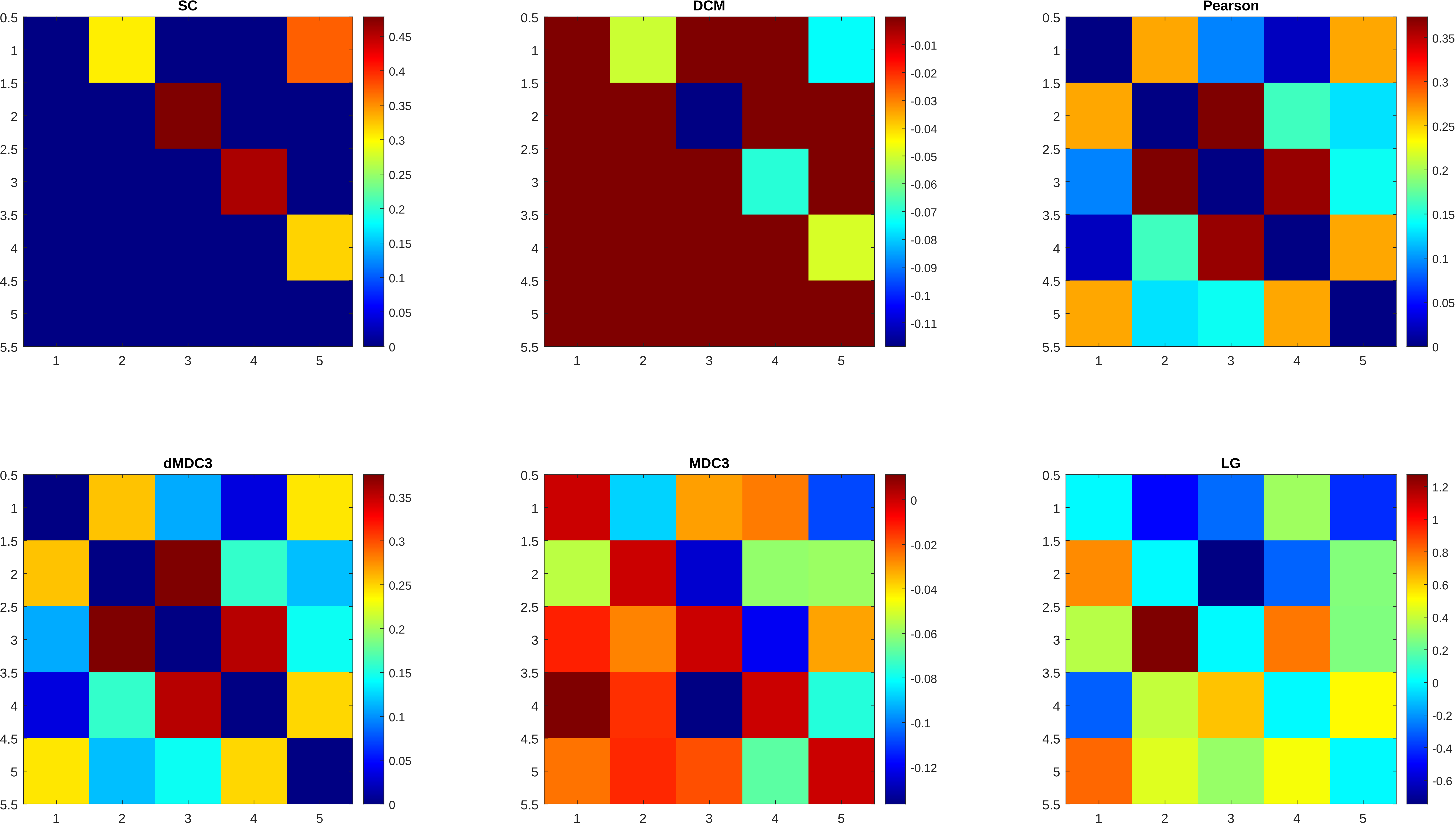
SC
DCM
Pearson
dMDC3
MDC3
LG
0.5 1 1.5 2 2.5 3 3.5 4 4.5 5 5.5
1 2 3 4 5
0 0.05 0.1 0.15 0.2 0.25 0.3 0.35 0.4 0.45
-0.11 -0.1 -0.09 -0.08 -0.07 -0.06 -0.05 -0.04 -0.03 -0.02 -0.01
0 0.05 0.1 0.15 0.2 0.25 0.3 0.35
0 0.05 0.1 0.15 0.2 0.25 0.3 0.35
-0.12 -0.1 -0.08 -0.06 -0.04 -0.02 0
-0.6 -0.4 -0.2 0 0.2 0.4 0.6 0.8 1 1.2

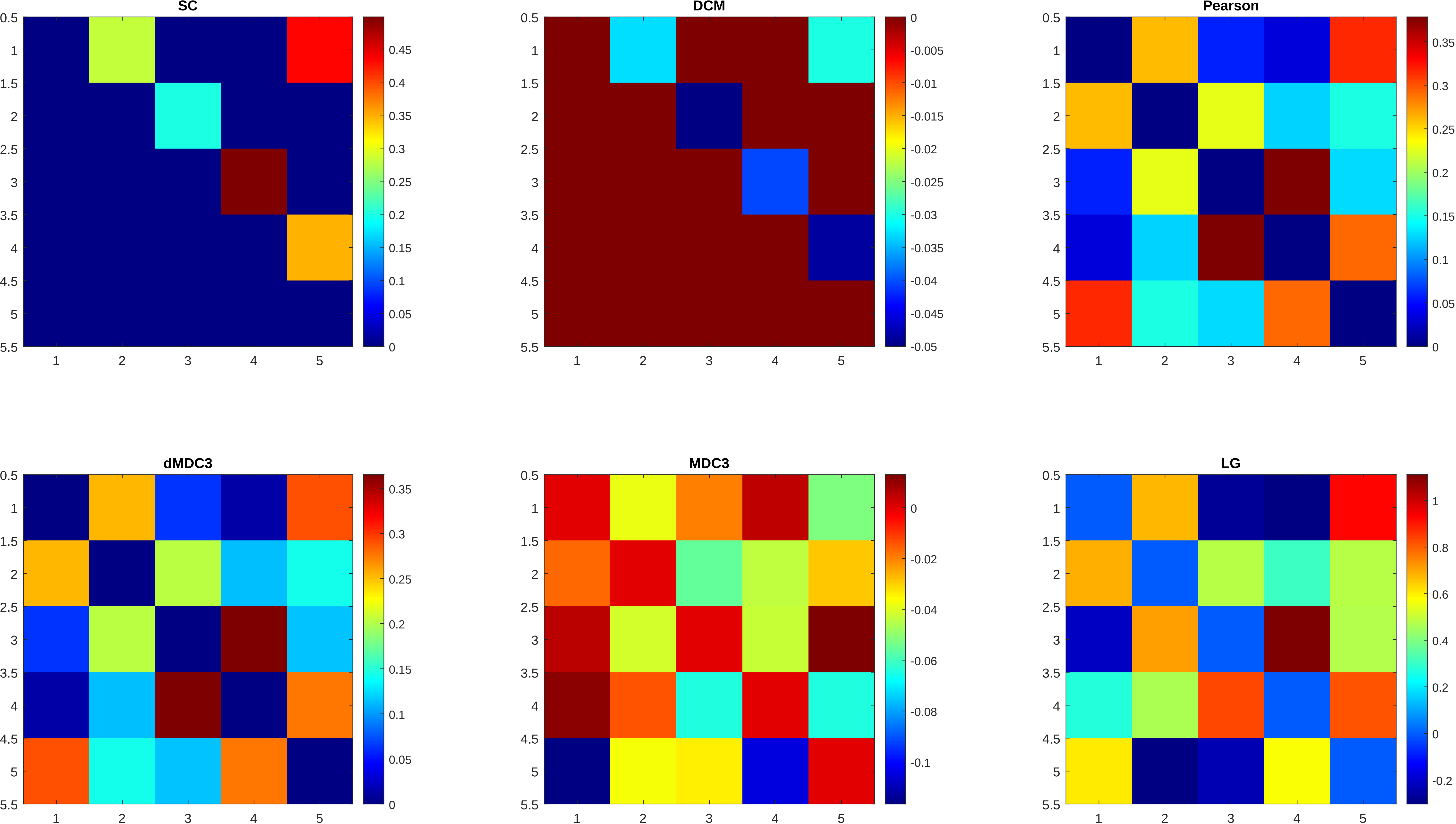
SC
DCM
Pearson
dMDC3
MDC3
LG
0.5
1
1.5
2
2.5
3
3.5
4
4.5
5
5.5
1 2 3 4 5
0 0.05 0.1 0.15 0.2 0.25 0.3 0.35 0.4 0.45
0 -0.005 -0.01 -0.015 -0.02 -0.025 -0.03 -0.035 -0.04 -0.045 -0.05
0 0.05 0.1 0.15 0.2 0.25 0.3 0.35
0 0.05 0.1 0.15 0.2 0.25 0.3 0.35
0 -0.02 -0.04 -0.06 -0.08 -0.1
-0.2 0 0.2 0.4 0.6 0.8 1

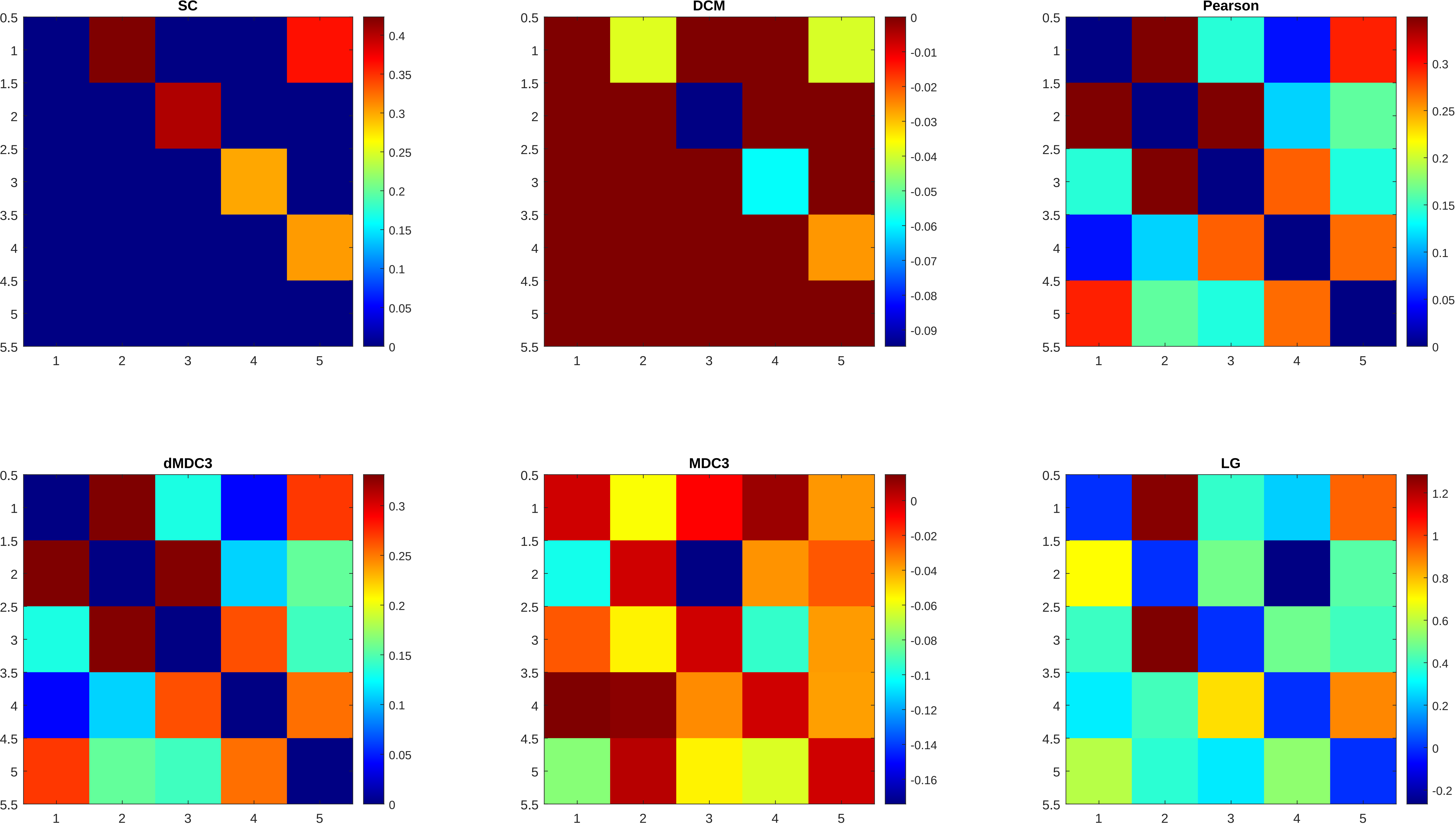

SC
DCM
Pearson
dMDC3
MDC3
LG

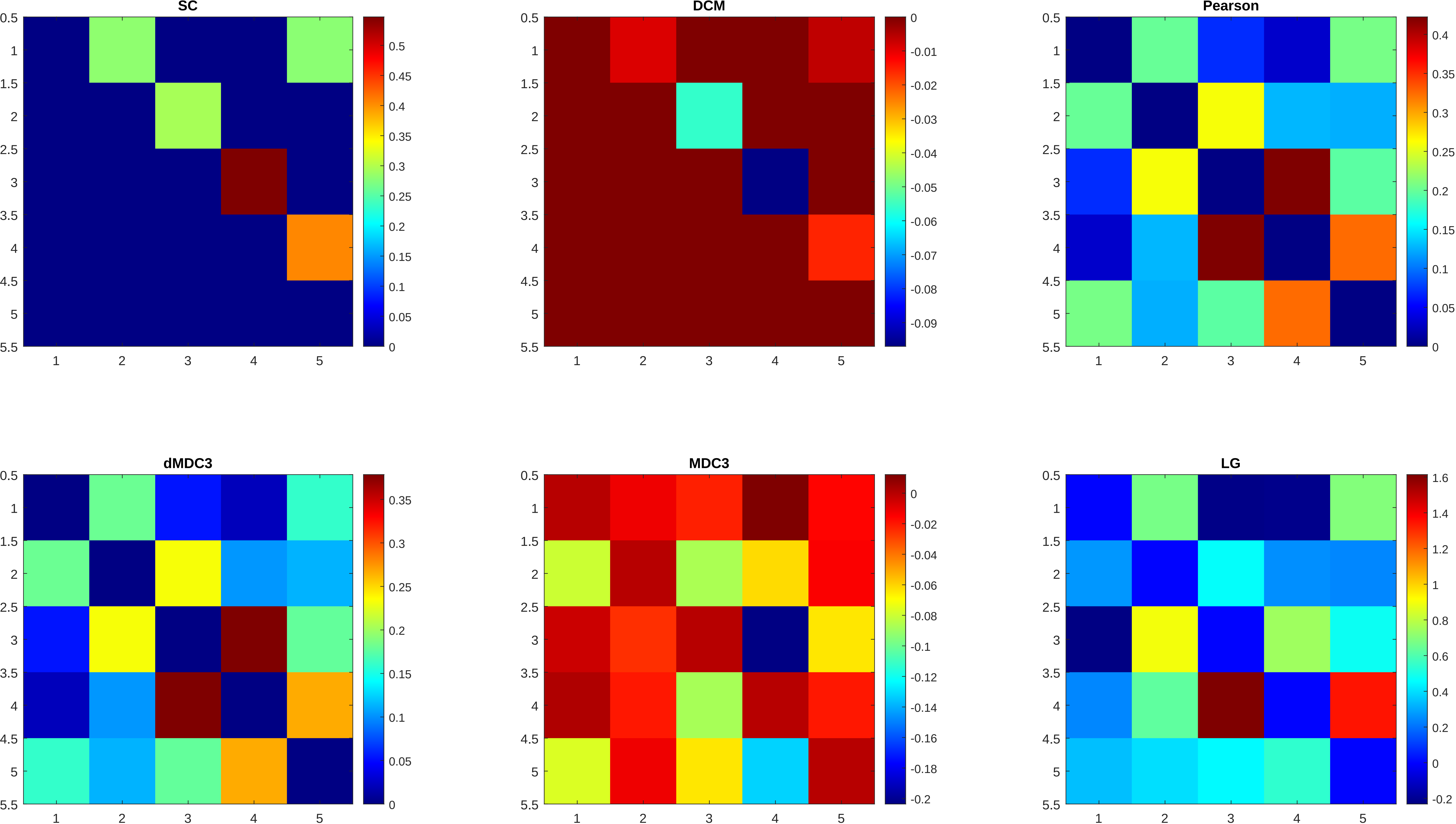
SC
DCM
Pearson
dMDC3
MDC3
LG

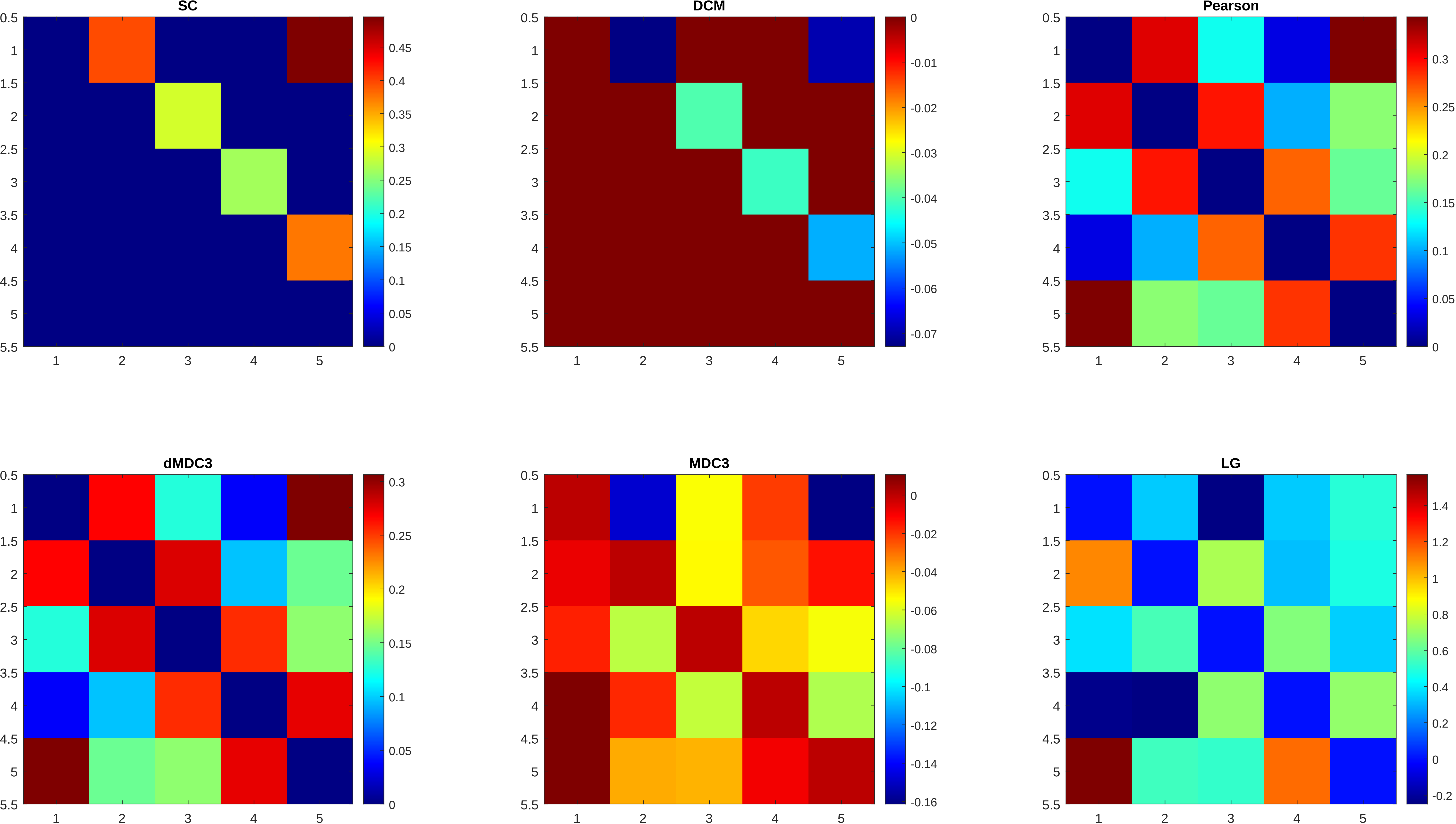

SC
DCM
Pearson
dMDC3
MDC3
LG

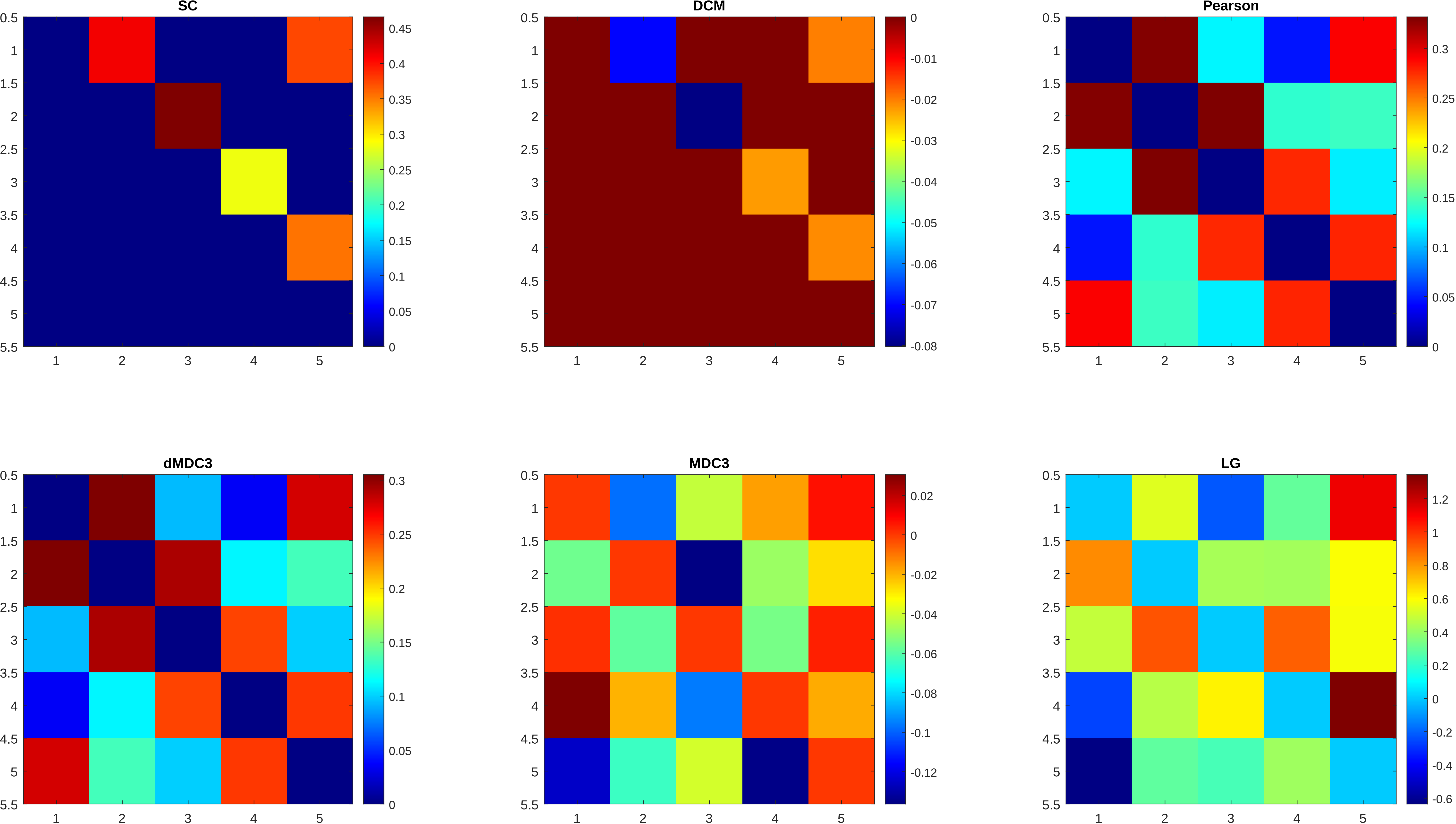
SC
DCM
Pearson
dMDC3
MDC3
LG
0.5
1
1.5
2
2.5
3
3.5
4
4.5
5
5.5
1
2
3
4
5
0.45
0.4
0.35
0.3
0.25
0.2
0.15
0.1
0.05
0
-0.01
-0.02
-0.03
-0.04
-0.05
-0.06
-0.07
-0.08
0.02
-0.1
-0.12
1.2
0.8
0.6
-0.2
-0.4
-0.6

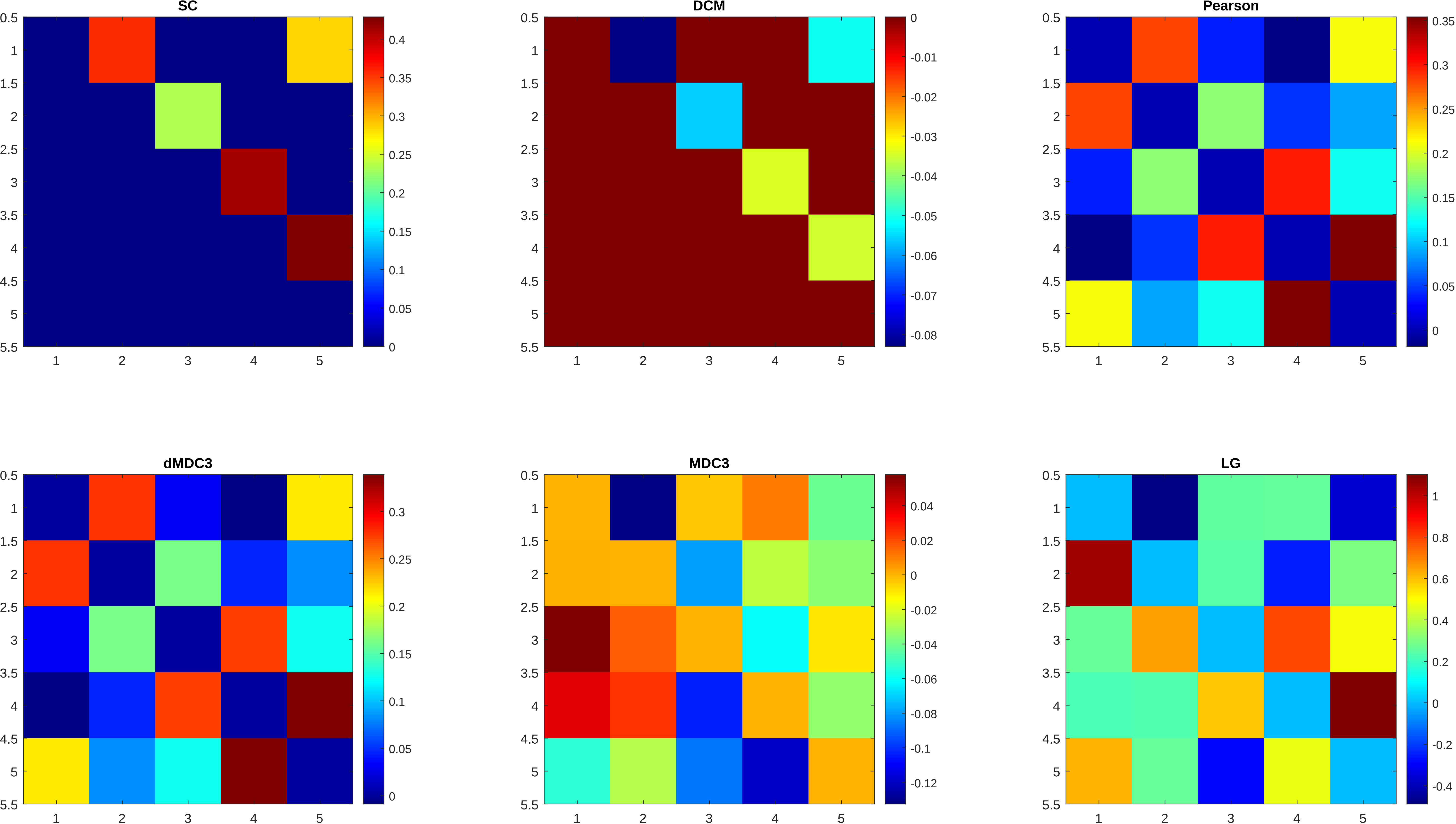
SC
0.5 1 1.5 2 2.5 3 3.5 4 4.5 5 5.5
1 2 3 4 5
0 0.05 0.1 0.15 0.2 0.25 0.3 0.35 0.4
DCM
0.5 1 1.5 2 2.5 3 3.5 4 4.5 5 5.5
1 2 3 4 5
0 -0.01 -0.02 -0.03 -0.04 -0.05 -0.06 -0.07 -0.08
Pearson
0.5 1 1.5 2 2.5 3 3.5 4 4.5 5 5.5
1 2 3 4 5
0.35 0.3 0.25 0.2 0.15 0.1 0.05 0
dMDC3
0.5 1 1.5 2 2.5 3 3.5 4 4.5 5 5.5
1 2 3 4 5
0.3 0.25 0.2 0.15 0.1 0.05 0
MDC3
0.5 1 1.5 2 2.5 3 3.5 4 4.5 5 5.5
1 2 3 4 5
0.04 0.02 0 -0.02 -0.04 -0.06 -0.08 -0.1 -0.12
LG
0.5 1 1.5 2 2.5 3 3.5 4 4.5 5 5.5
1 2 3 4 5
1 0.8 0.6 0.4 0.2 0 -0.2 -0.4

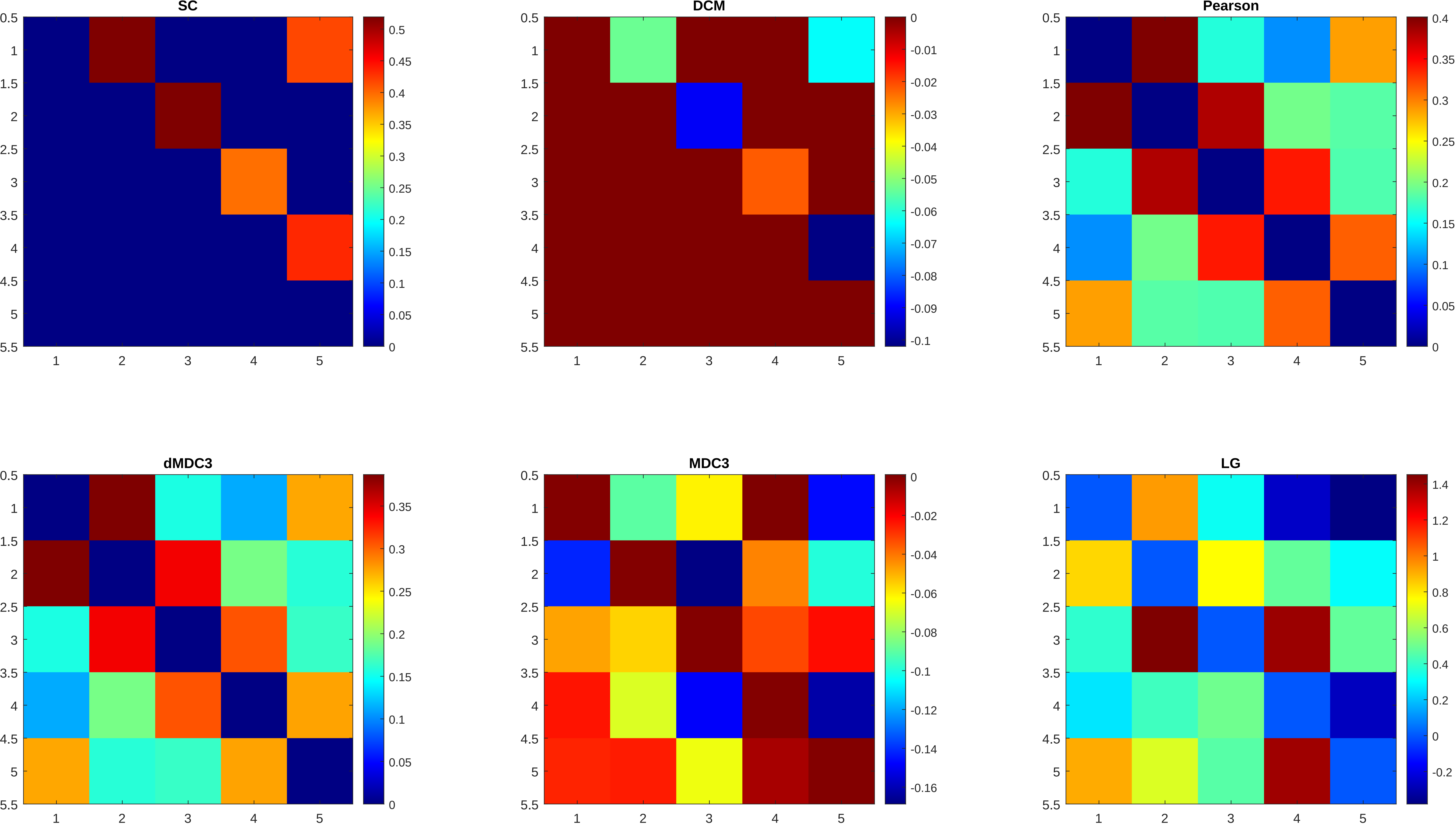
SC
DCM
Pearson
dMDC3
MDC3
LG

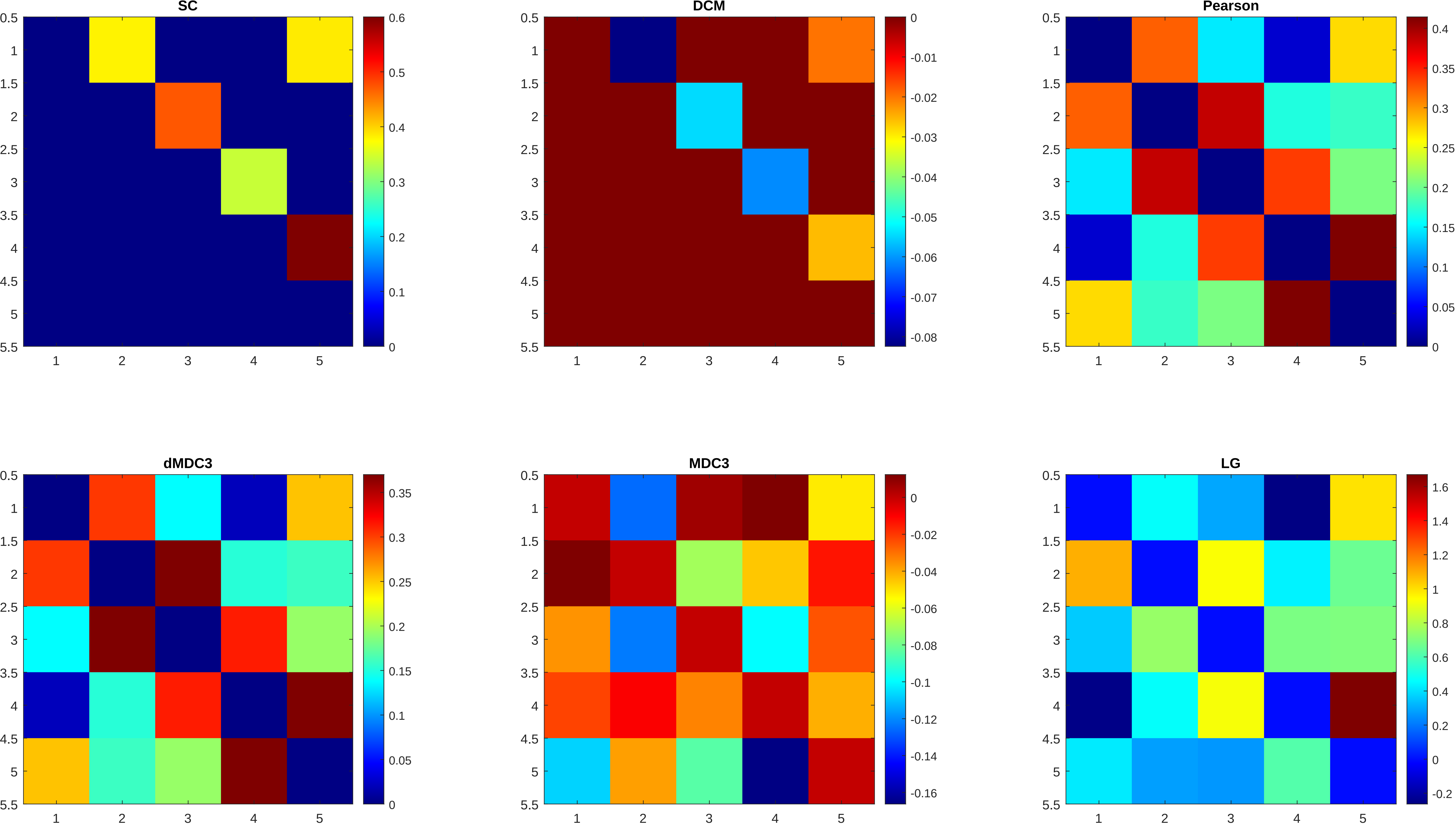
SC
DCM
Pearson
dMDC3
MDC3
LG

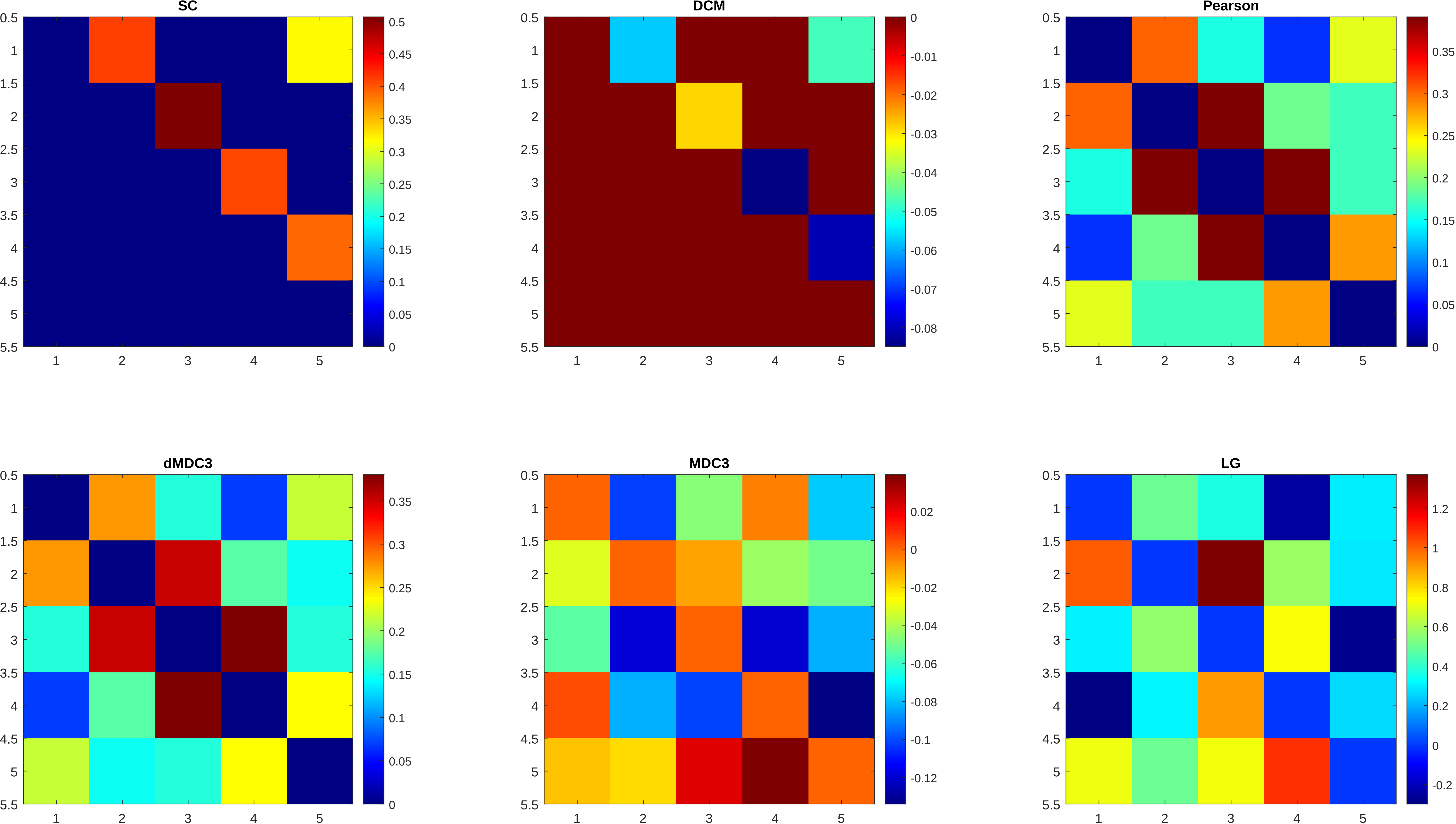

SC
DCM
Pearson
dMDC3
MDC3
LG

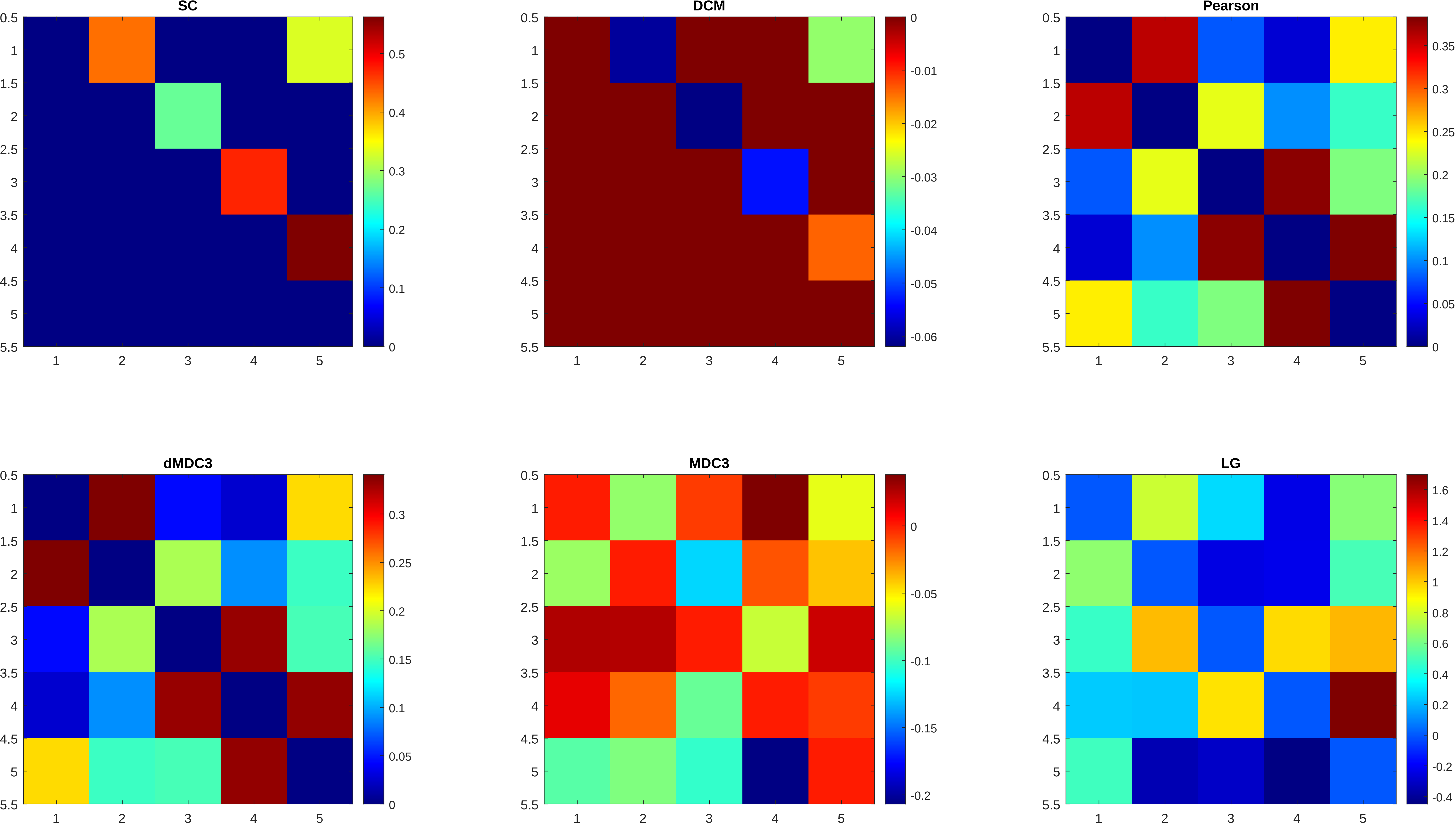
SC
DCM
Pearson
dMDC3
MDC3
LG

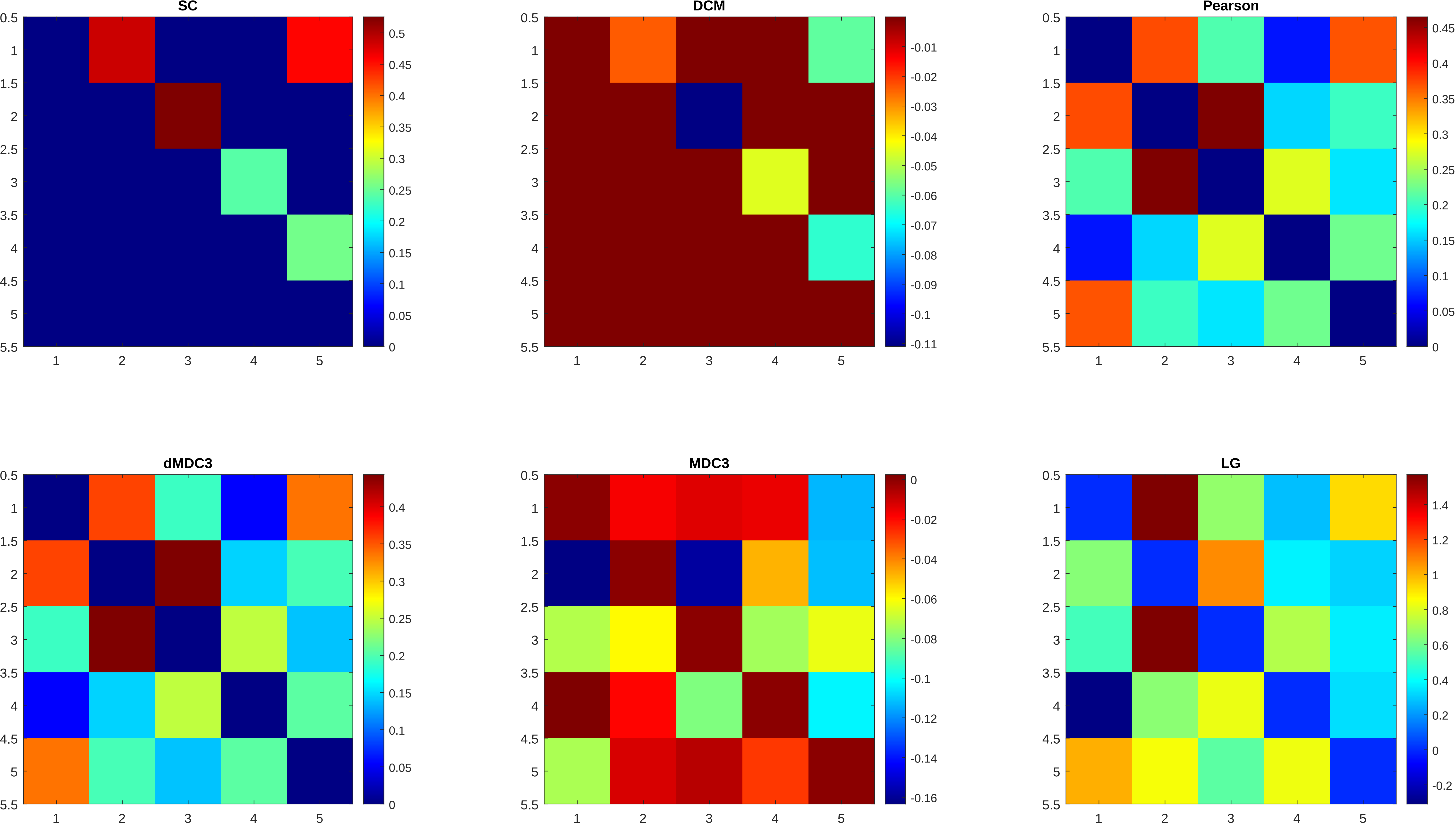

SC
DCM
Pearson
dMDC3
MDC3
LG
0.5
1
1.5
2
2.5
3
3.5
4
4.5
5
5.5
1 2 3 4 5
0 0.05 0.1 0.15 0.2 0.25 0.3 0.35 0.4 0.45 0.5
-0.11 -0.1 -0.09 -0.08 -0.07 -0.06 -0.05 -0.04 -0.03 -0.02 -0.01
0 0.05 0.1 0.15 0.2 0.25 0.3 0.35 0.4 0.45
0 0.05 0.1 0.15 0.2 0.25 0.3 0.35 0.4
-0.16 -0.14 -0.12 -0.1 -0.08 -0.06 -0.04 -0.02 0
-0.2 0 0.2 0.4 0.6 0.8 1 1.2 1.4

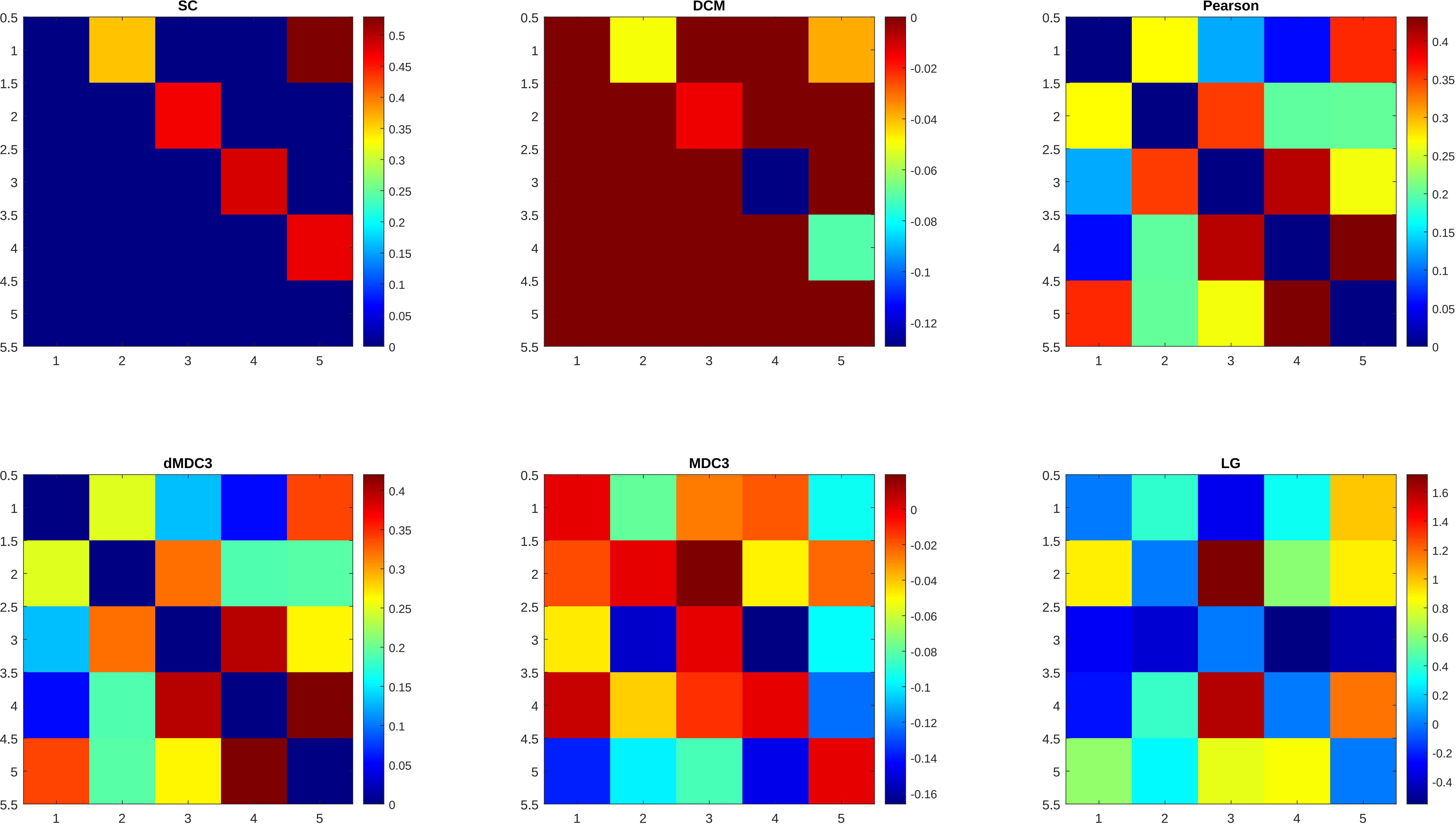
SC
0.5
1
1.5
2
2.5
3
3.5
4
4.5
5
5.5
1 2 3 4 5
0 0.05 0.1 0.15 0.2 0.25 0.3 0.35 0.4 0.45 0.5
DCM
0.5
1
1.5
2
2.5
3
3.5
4
4.5
5
5.5
1 2 3 4 5
0 -0.02 -0.04 -0.06 -0.08 -0.1 -0.12
Pearson
0.5
1
1.5
2
2.5
3
3.5
4
4.5
5
5.5
1 2 3 4 5
0 0.05 0.1 0.15 0.2 0.25 0.3 0.35 0.4
dMDC3
0.5
1
1.5
2
2.5
3
3.5
4
4.5
5
5.5
1 2 3 4 5
0 0.05 0.1 0.15 0.2 0.25 0.3 0.35 0.4
MDC3
0.5
1
1.5
2
2.5
3
3.5
4
4.5
5
5.5
1 2 3 4 5
0 -0.02 -0.04 -0.06 -0.08 -0.1 -0.12 -0.14 -0.16
LG
0.5
1
1.5
2
2.5
3
3.5
4
4.5
5
5.5
1 2 3 4 5
1.6 1.4 1.2 1 0.8 0.6 0.4 0.2 0 -0.2 -0.4

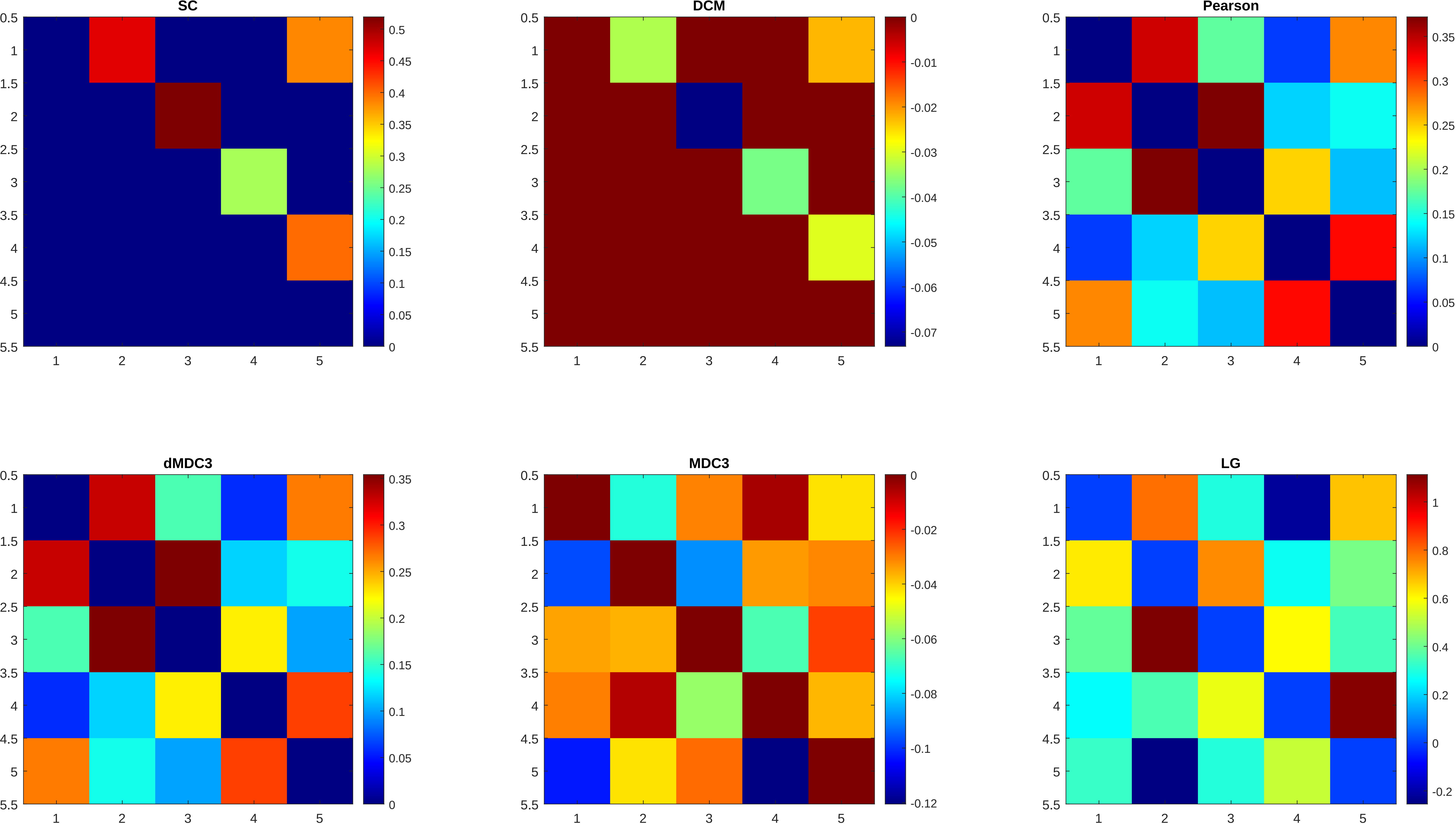
SC
DCM
Pearson
dMDC3
MDC3
LG

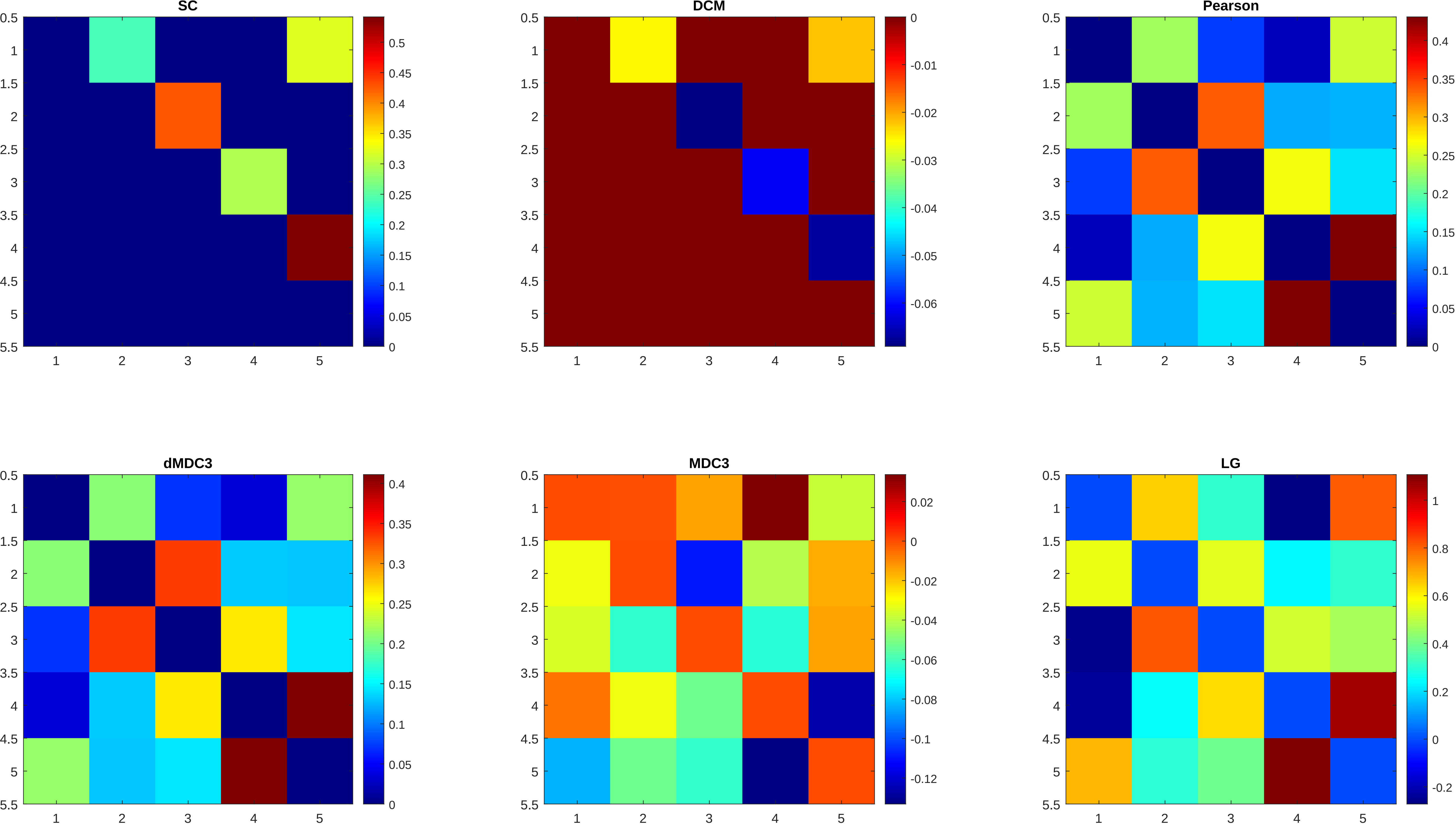
SC
0.5 1 1.5 2 2.5 3 3.5 4 4.5 5 5.5
1 2 3 4 5
0 0.05 0.1 0.15 0.2 0.25 0.3 0.35 0.4 0.45 0.5
DCM
0.5 1 1.5 2 2.5 3 3.5 4 4.5 5 5.5
1 2 3 4 5
0 -0.01 -0.02 -0.03 -0.04 -0.05 -0.06
Pearson
0.5 1 1.5 2 2.5 3 3.5 4 4.5 5 5.5
1 2 3 4 5
0 0.05 0.1 0.15 0.2 0.25 0.3 0.35 0.4
dMDC3
0.5 1 1.5 2 2.5 3 3.5 4 4.5 5 5.5
1 2 3 4 5
0 0.05 0.1 0.15 0.2 0.25 0.3 0.35 0.4
MDC3
0.5 1 1.5 2 2.5 3 3.5 4 4.5 5 5.5
1 2 3 4 5
0.02 0 -0.02 -0.04 -0.06 -0.08 -0.1 -0.12
LG
0.5 1 1.5 2 2.5 3 3.5 4 4.5 5 5.5
1 2 3 4 5
1 0.8 0.6 0.4 0.2 0 -0.2

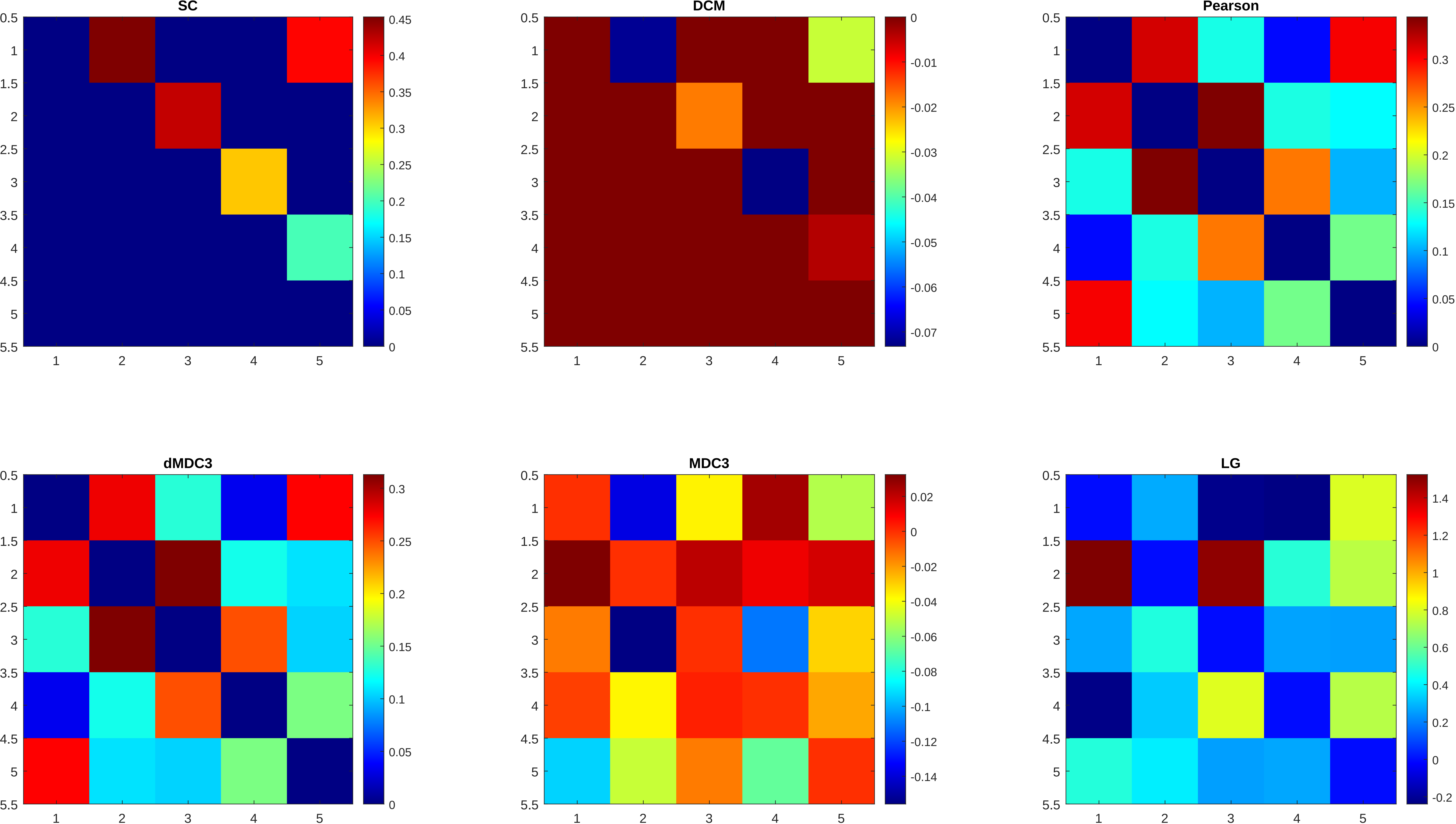
SC
0.45
0.4
0.35
0.3
0.25
0.2
0.15
0.1
0.05
0
DCM
0
-0.01
-0.02
-0.03
-0.04
-0.05
-0.06
-0.07
Pearson
0.3
0.25
0.2
0.15
0.1
0.05
0
dMDC3
0.3
0.25
0.2
0.15
0.1
0.05
0
MDC3
0.02
0
-0.02
-0.04
-0.06
-0.08
-0.1
-0.12
-0.14
LG
1.4
1.2
1
0.8
0.6
0.4
0.2
0
-0.2
0.5
1
1.5
2
2.5
3
3.5
4
4.5
5
5.5
1
2
3
4
5

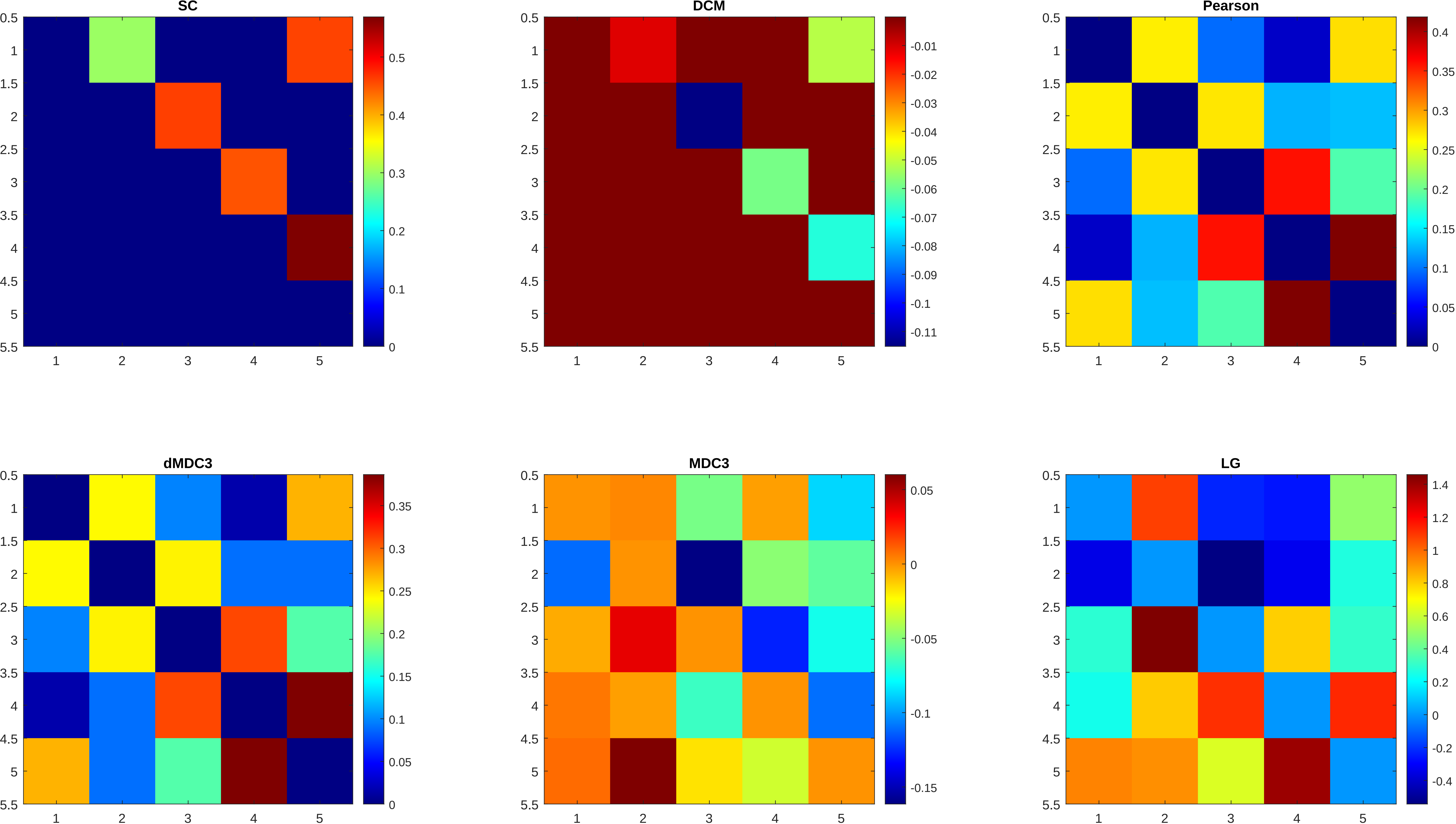
SC
DCM
Pearson
dMDC3
MDC3
LG
0.5
1
1.5
2
2.5
3
3.5
4
4.5
5
5.5
1
2
3
4
5
0
0.1
0.2
0.3
0.4
0.5
-0.01
-0.02
-0.03
-0.04
-0.05
-0.06
-0.07
-0.08
-0.09
-0.1
-0.11
0.05
0.15
0.25
0.35
-0.15
-0.4
-0.2
0.6
0.8
1
1.2
1.4

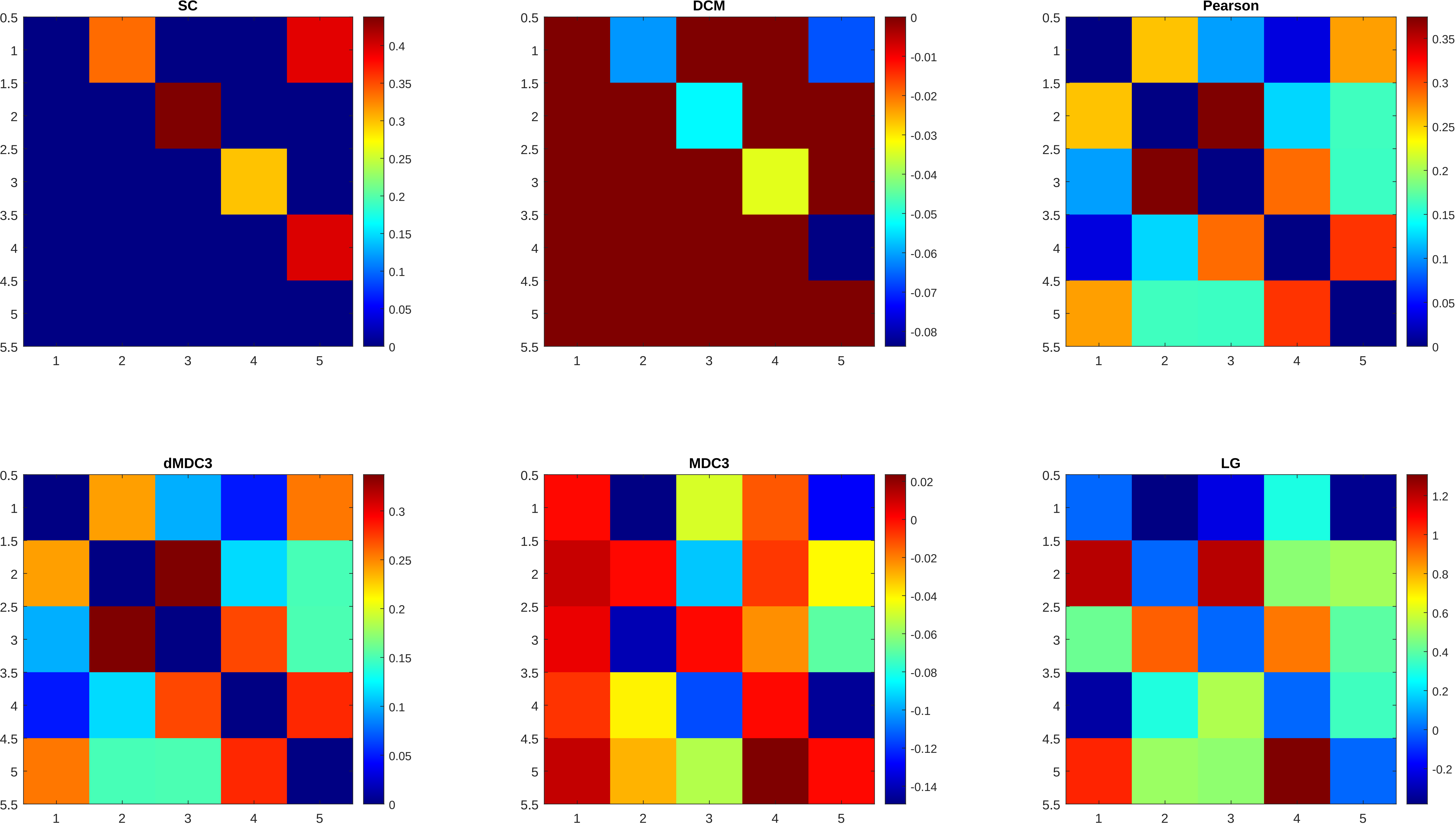
SC
DCM
Pearson
dMDC3
MDC3
LG

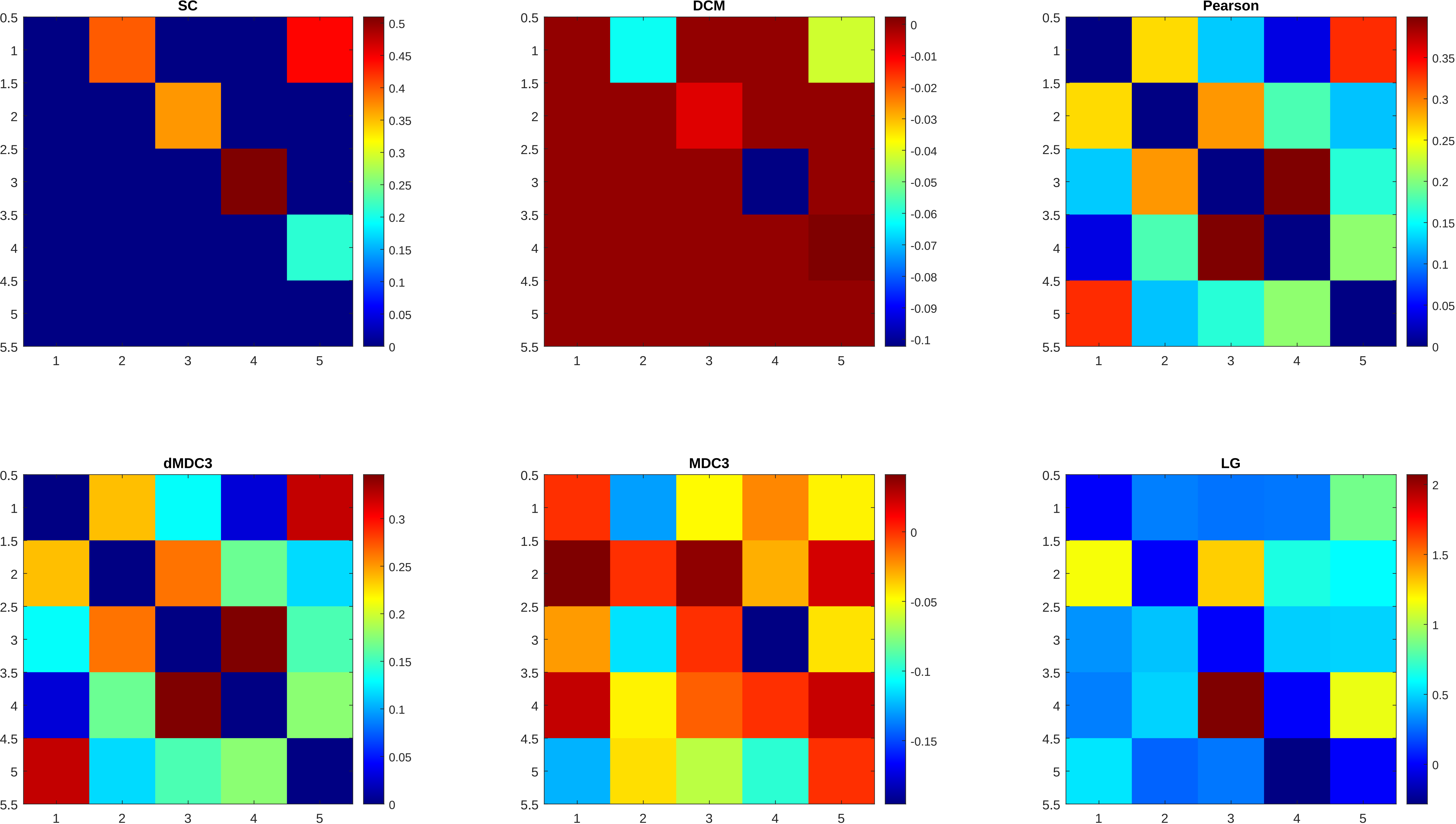
SC
0.5
1
1.5
2
2.5
3
3.5
4
4.5
5
5.5
1 2 3 4 5
0.5 0.45 0.4 0.35 0.3 0.25 0.2 0.15 0.1 0.05 0
DCM
0 -0.01 -0.02 -0.03 -0.04 -0.05 -0.06 -0.07 -0.08 -0.09 -0.1
Pearson
0.35 0.3 0.25 0.2 0.15 0.1 0.05 0
dMDC3
0.3 0.25 0.2 0.15 0.1 0.05 0
MDC3
0 -0.05 -0.1 -0.15
LG
2 1.5 1 0.5 0

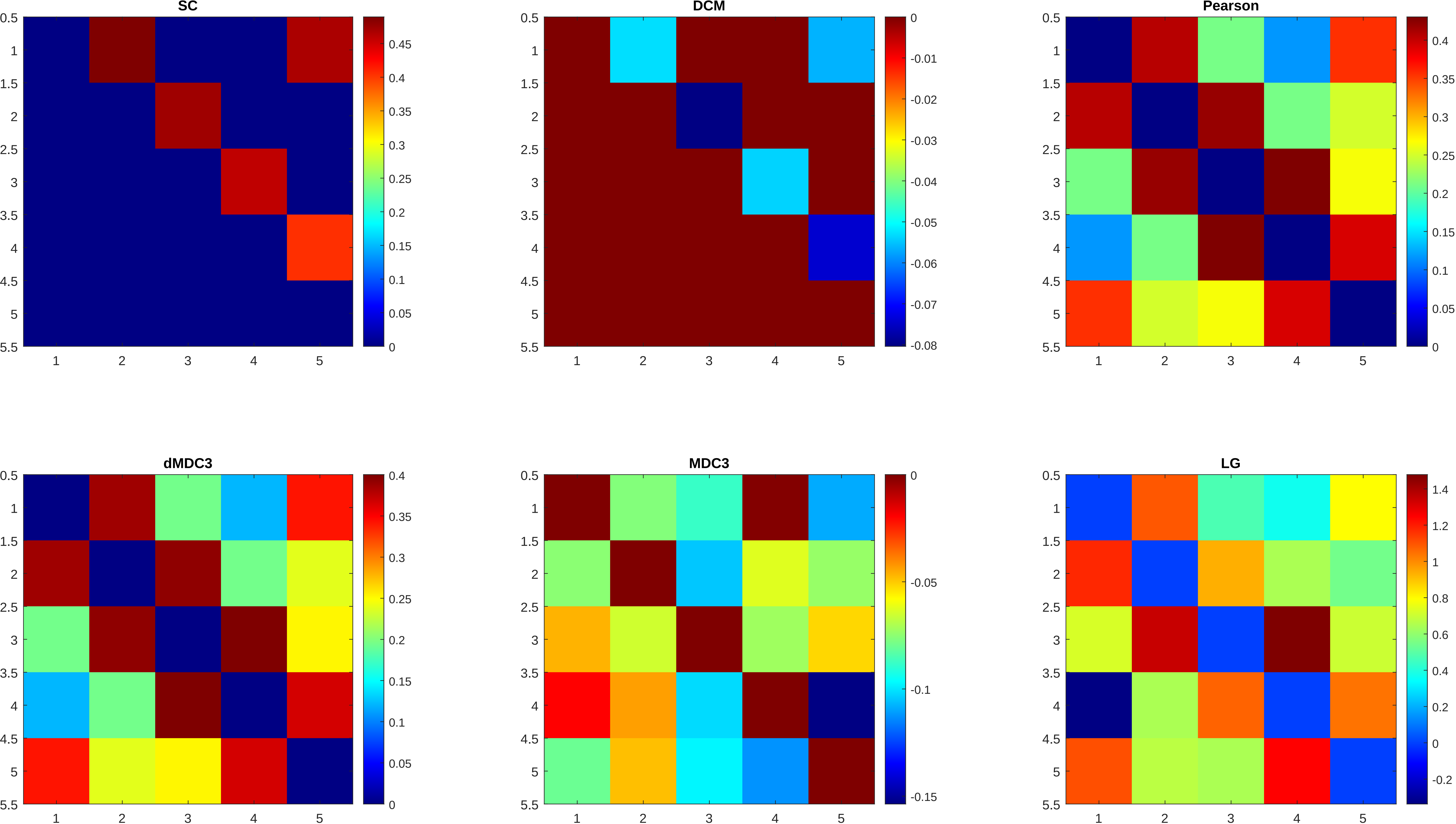
SC
DCM
Pearson
dMDC3
MDC3
LG

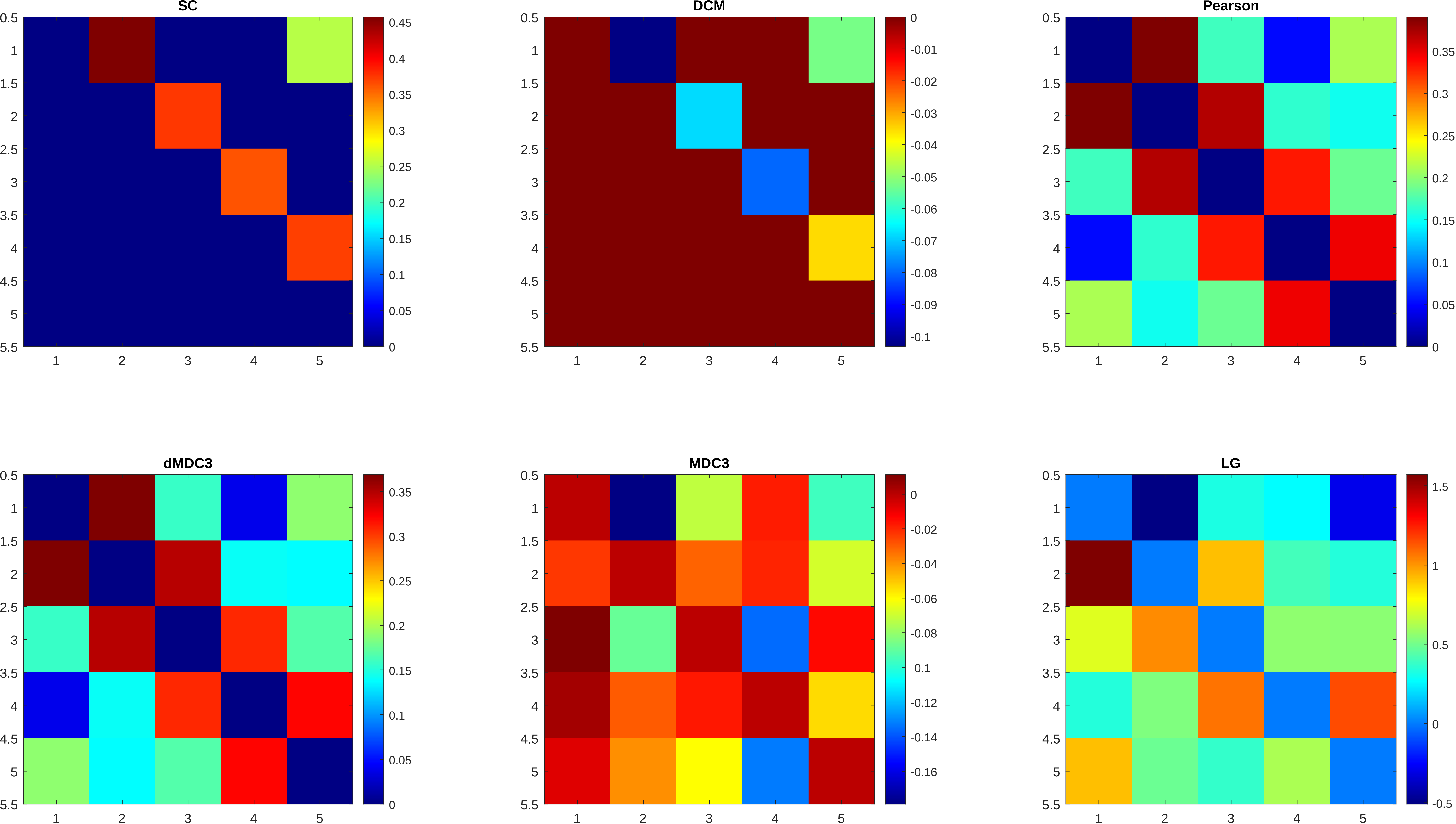
SC
0.5 1 1.5 2 2.5 3 3.5 4 4.5 5 5.5
1 2 3 4 5
0 0.05 0.1 0.15 0.2 0.25 0.3 0.35 0.4 0.45
DCM
0.5 1 1.5 2 2.5 3 3.5 4 4.5 5 5.5
1 2 3 4 5
0 -0.01 -0.02 -0.03 -0.04 -0.05 -0.06 -0.07 -0.08 -0.09 -0.1
Pearson
0.5 1 1.5 2 2.5 3 3.5 4 4.5 5 5.5
1 2 3 4 5
0 0.05 0.1 0.15 0.2 0.25 0.3 0.35
dMDC3
0.5 1 1.5 2 2.5 3 3.5 4 4.5 5 5.5
1 2 3 4 5
0 0.05 0.1 0.15 0.2 0.25 0.3 0.35
MDC3
0.5 1 1.5 2 2.5 3 3.5 4 4.5 5 5.5
1 2 3 4 5
0 -0.02 -0.04 -0.06 -0.08 -0.1 -0.12 -0.14 -0.16
LG
0.5 1 1.5 2 2.5 3 3.5 4 4.5 5 5.5
1 2 3 4 5
-0.5 0 0.5 1 1.5

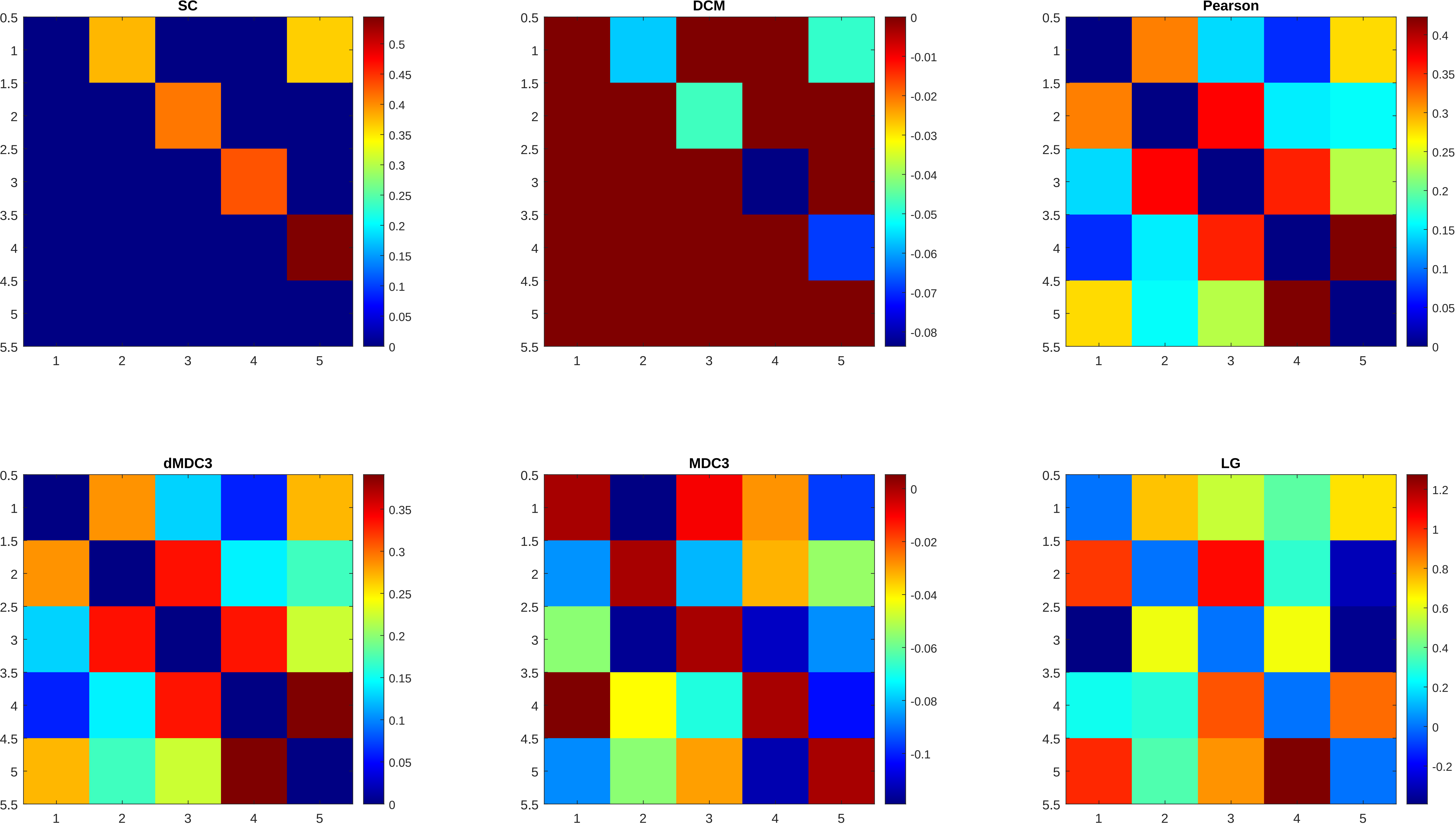
SC
DCM
Pearson
dMDC3
MDC3
LG

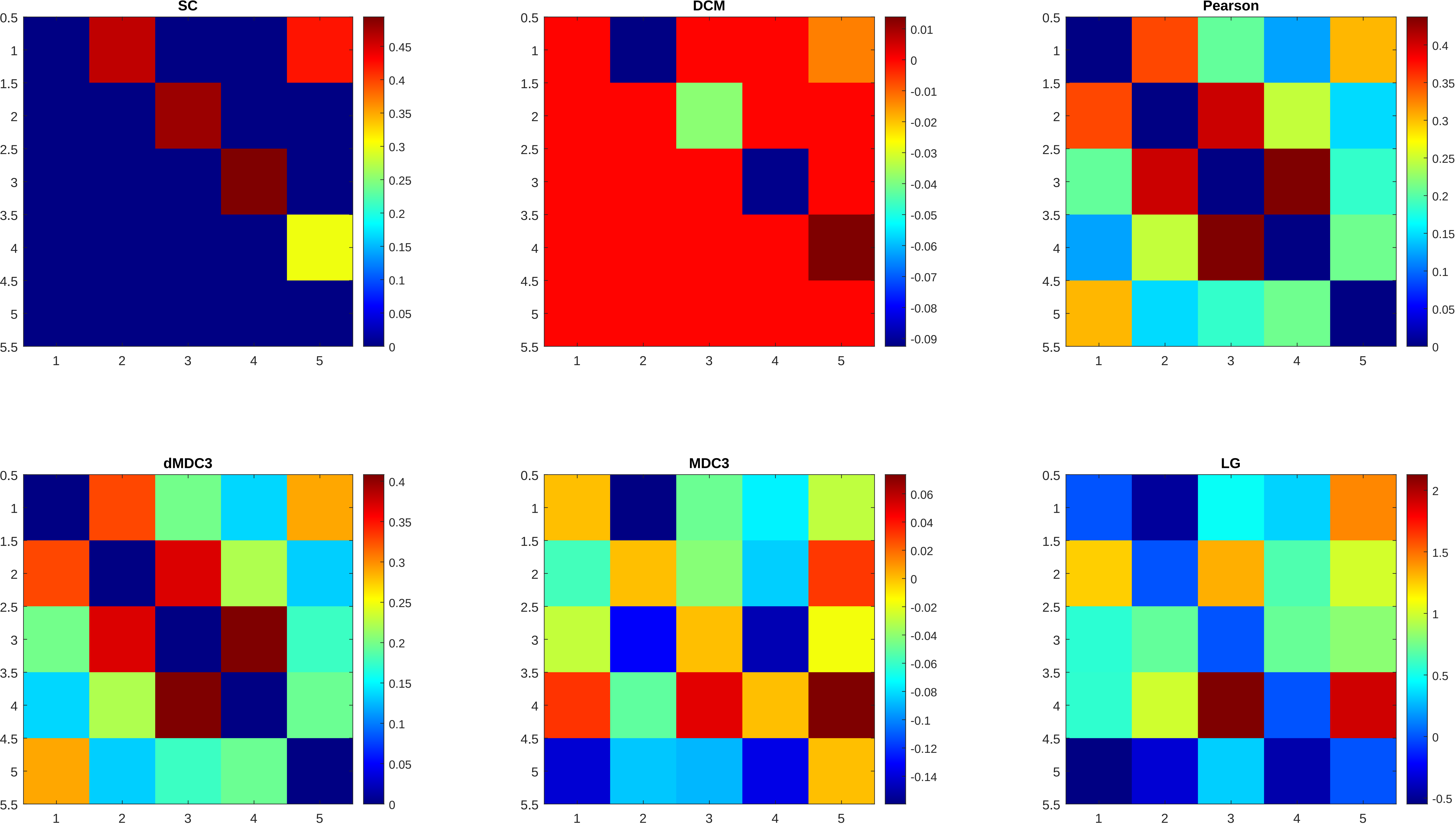
SC
DCM
Pearson
dMDC3
MDC3
LG
0.5
1
1.5
2
2.5
3
3.5
4
4.5
5
5.5
1
2
3
4
5
0.45
0.4
0.35
0.3
0.25
0.2
0.15
0.1
0.05
0
0.01
0
-0.01
-0.02
-0.03
-0.04
-0.05
-0.06
-0.07
-0.08
-0.09
0.06
0.04
0.02
-0.02
-0.04
-0.06
-0.08
-0.1
-0.12
-0.14
2
1.5
1
0.5
-0.5

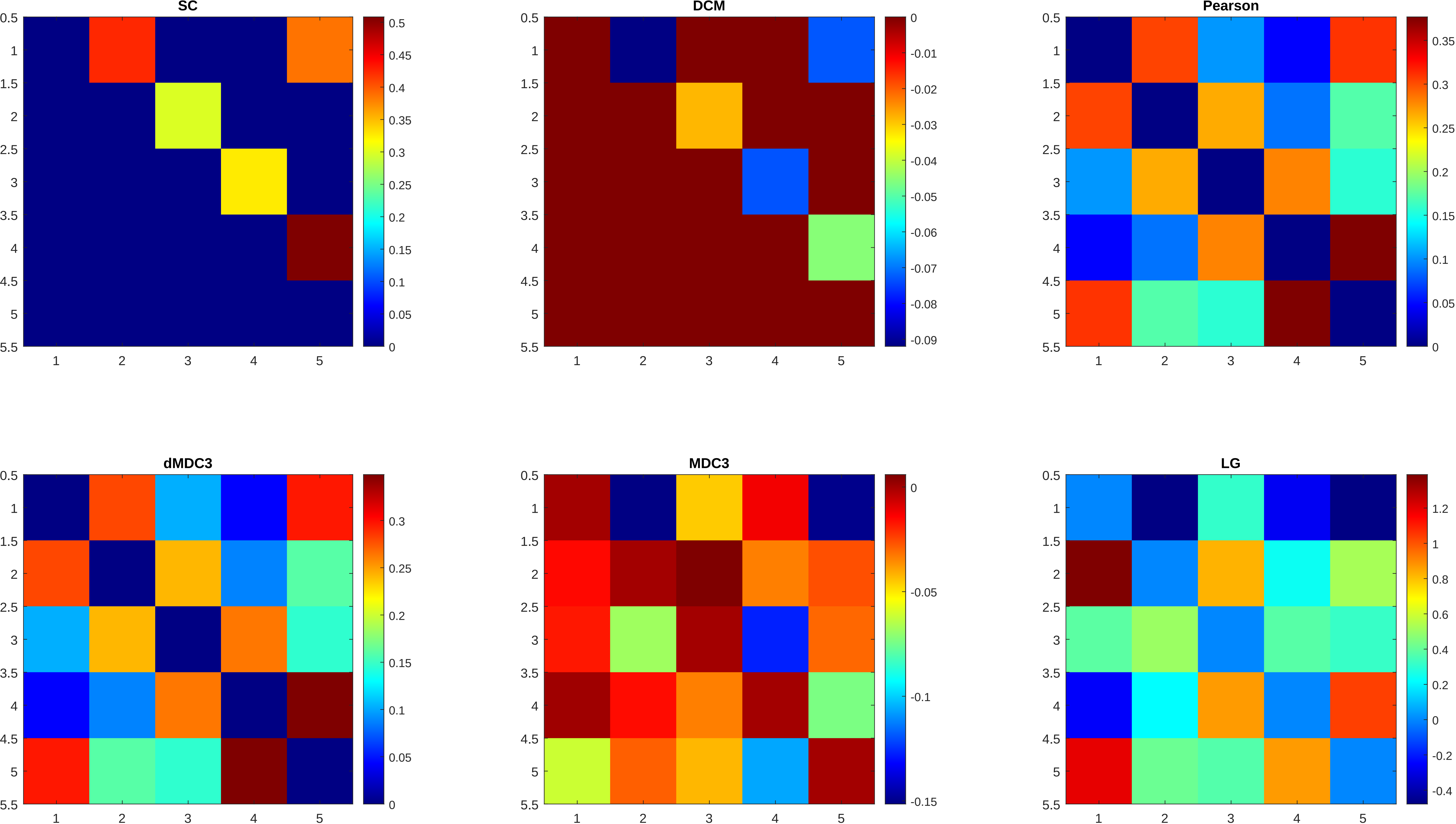
SC
DCM
Pearson
dMDC3
MDC3
LG
0.5
1
1.5
2
2.5
3
3.5
4
4.5
5
5.5
1
2
3
4
5
0.5
0.45
0.4
0.35
0.3
0.25
0.2
0.15
0.1
0.05
0
-0.01
-0.02
-0.03
-0.04
-0.05
-0.06
-0.07
-0.08
-0.09
0.35
0.3
0.25
0.2
0.15
0.1
0.05
0
-0.05
-0.1
-0.15
1.2
1
0.8
0.6
0.4
0.2
0
-0.2
-0.4

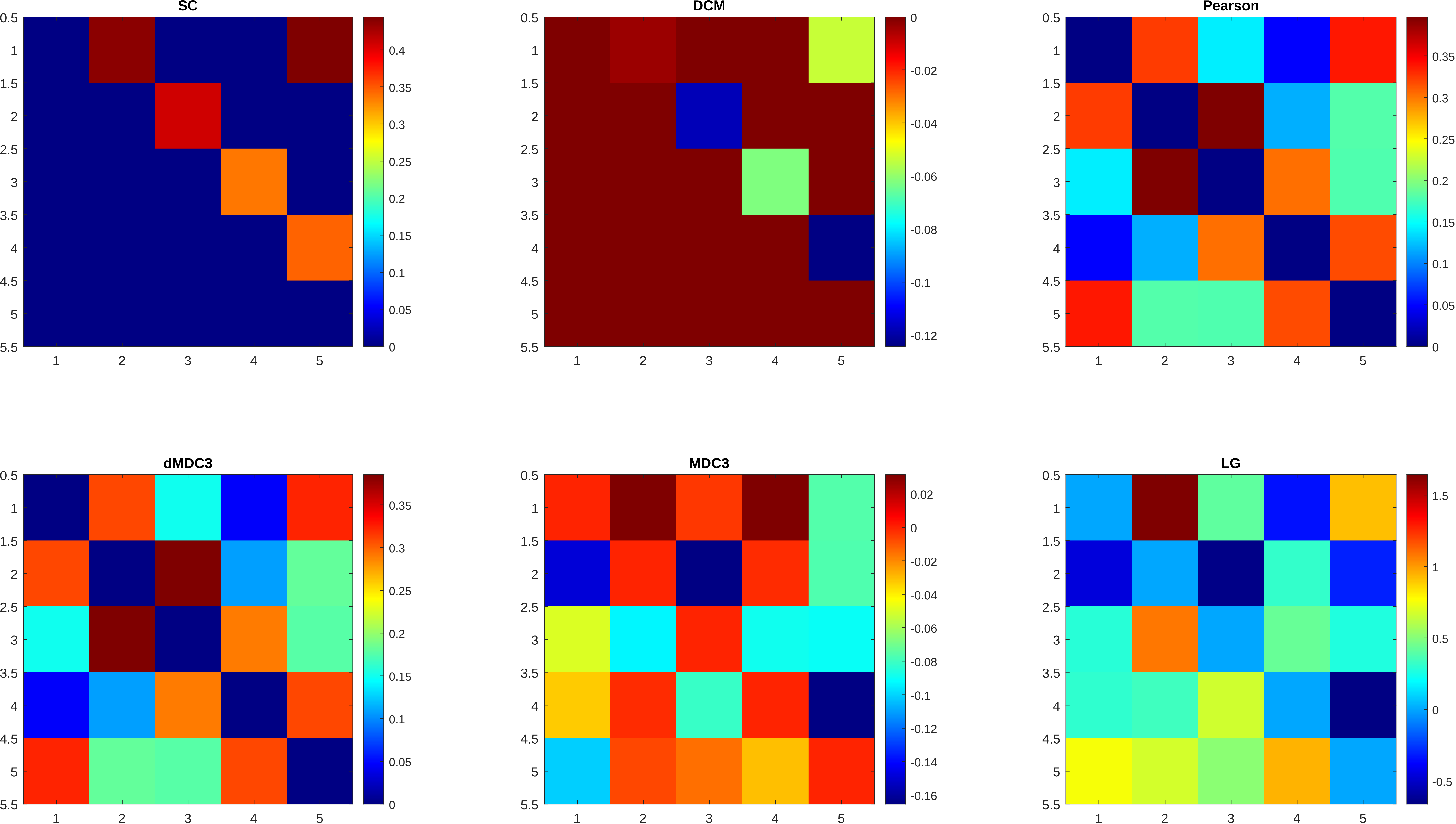
SC
DCM
Pearson
dMDC3
MDC3
LG
0.5 1 1.5 2 2.5 3 3.5 4 4.5 5 5.5
1 2 3 4 5
0 0.05 0.1 0.15 0.2 0.25 0.3 0.35 0.4
0 -0.02 -0.04 -0.06 -0.08 -0.1 -0.12
0.02 0 -0.02 -0.04 -0.06 -0.08 -0.1 -0.12 -0.14 -0.16
1.5 1 0.5 0 -0.5

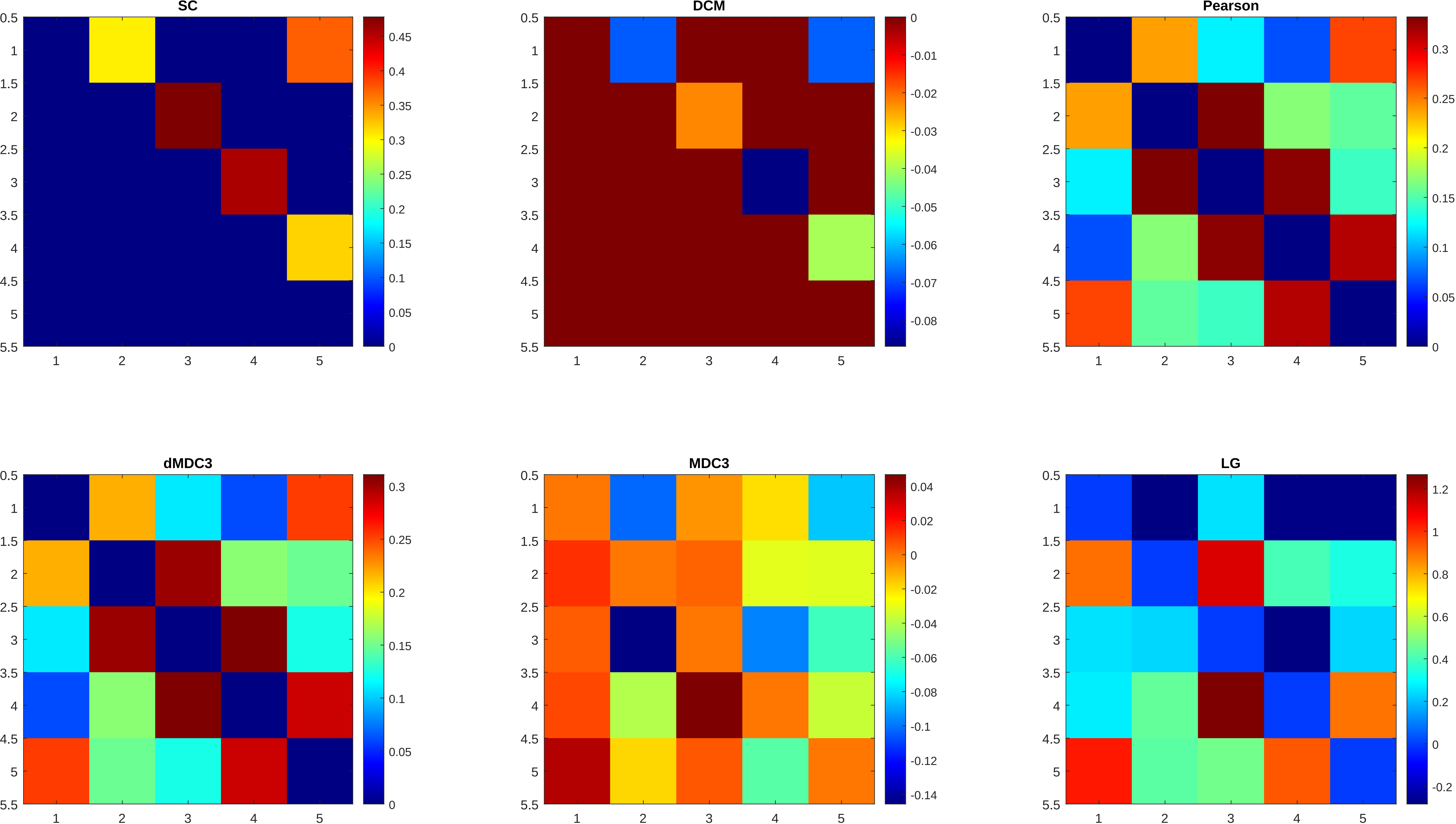
SC
DCM
Pearson
dMDC3
MDC3
LG

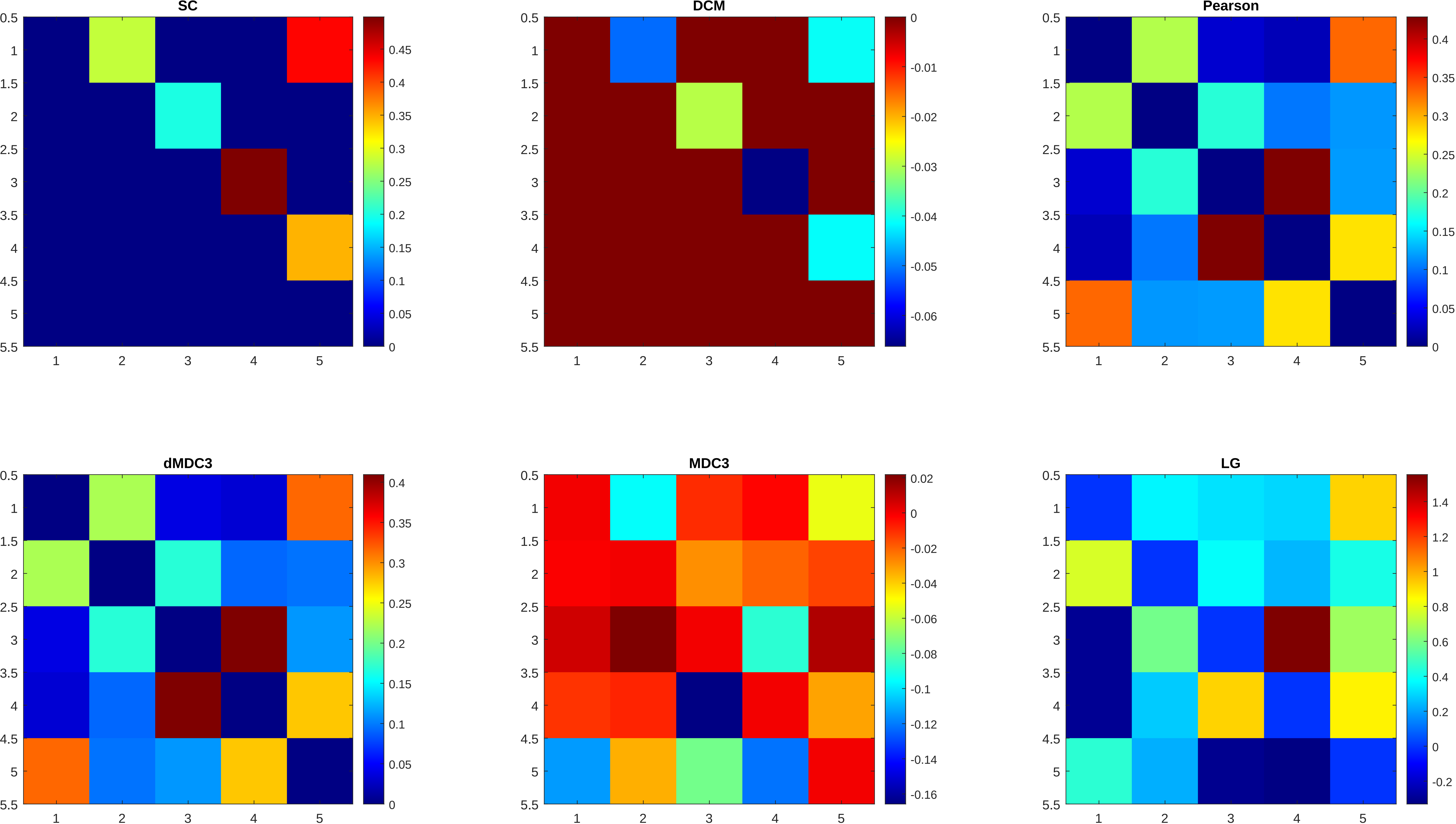
SC
DCM
Pearson
dMDC3
MDC3
LG
0.5
1
1.5
2
2.5
3
3.5
4
4.5
5
5.5
1
2
3
4
5
0
0.05
0.1
0.15
0.2
0.25
0.3
0.35
0.4
0.45
-0.01
-0.02
-0.03
-0.04
-0.05
-0.06
0.02
-0.08
-0.1
-0.12
-0.14
-0.16
1.4
1.2
0.8
0.6
-0.2

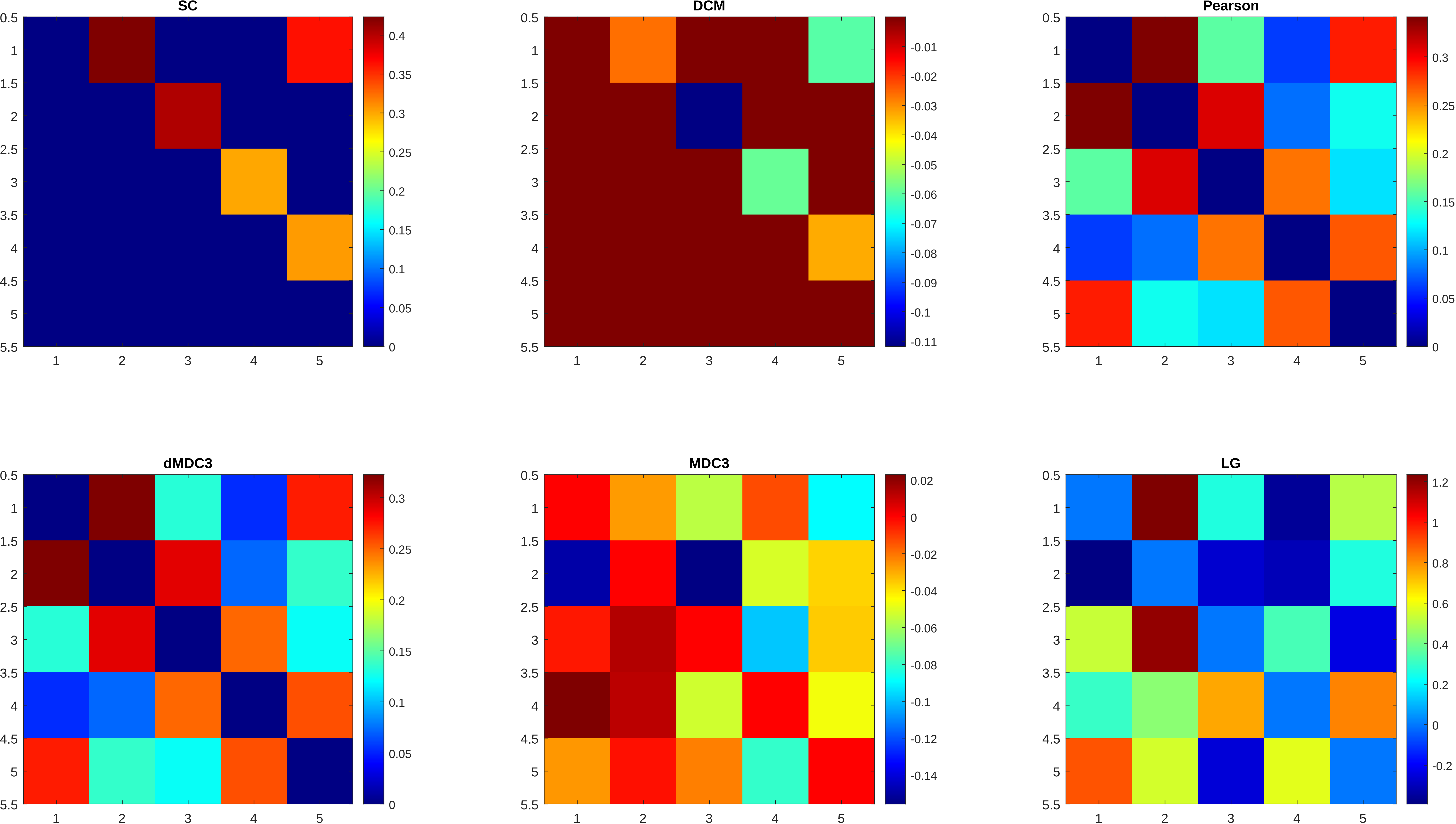
SC
DCM
Pearson
dMDC3
MDC3
LG

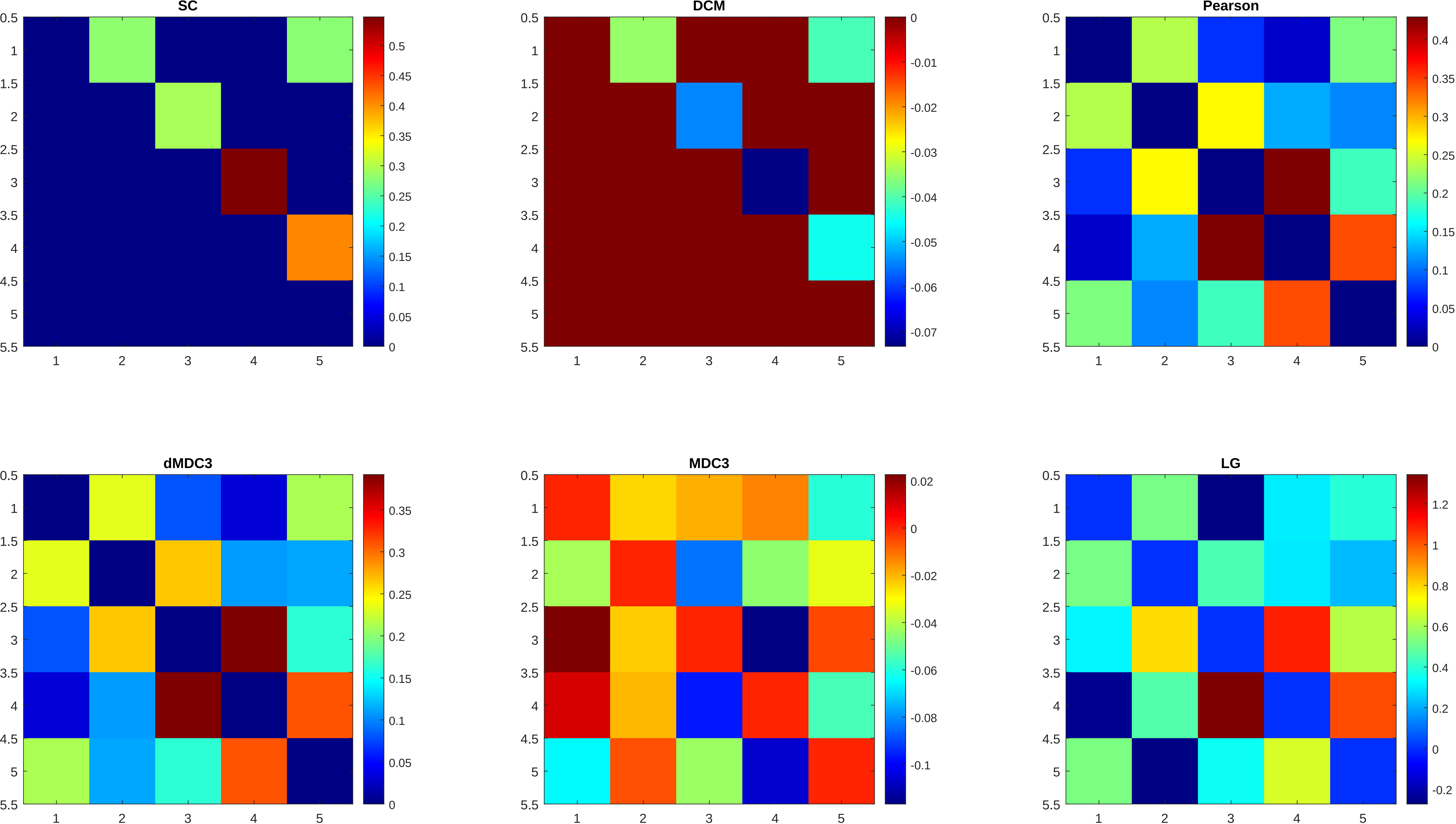
SC
DCM
Pearson
dMDC3
MDC3
LG
0.5
1
1.5
2
2.5
3
3.5
4
4.5
5
5.5
1
2
3
4
5

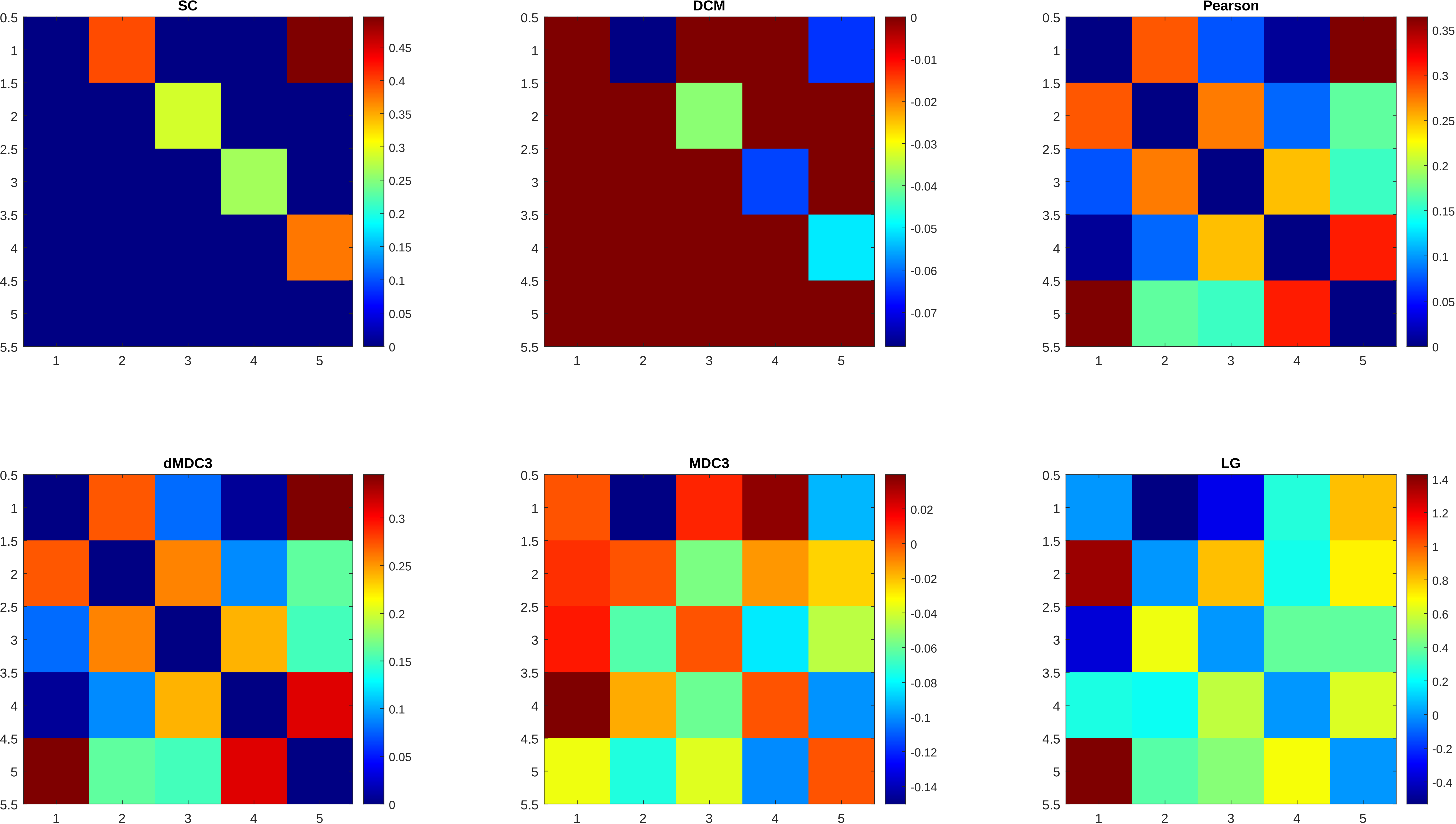
SC
DCM
Pearson
dMDC3
MDC3
LG
0.5 1 1.5 2 2.5 3 3.5 4 4.5 5 5.5
1 2 3 4 5
0 0.05 0.1 0.15 0.2 0.25 0.3 0.35 0.4 0.45
0 -0.01 -0.02 -0.03 -0.04 -0.05 -0.06 -0.07
0 0.05 0.1 0.15 0.2 0.25 0.3 0.35
0 0.05 0.1 0.15 0.2 0.25 0.3
0.02 0 -0.02 -0.04 -0.06 -0.08 -0.1 -0.12 -0.14
1.4 1.2 1 0.8 0.6 0.4 0.2 0 -0.2 -0.4

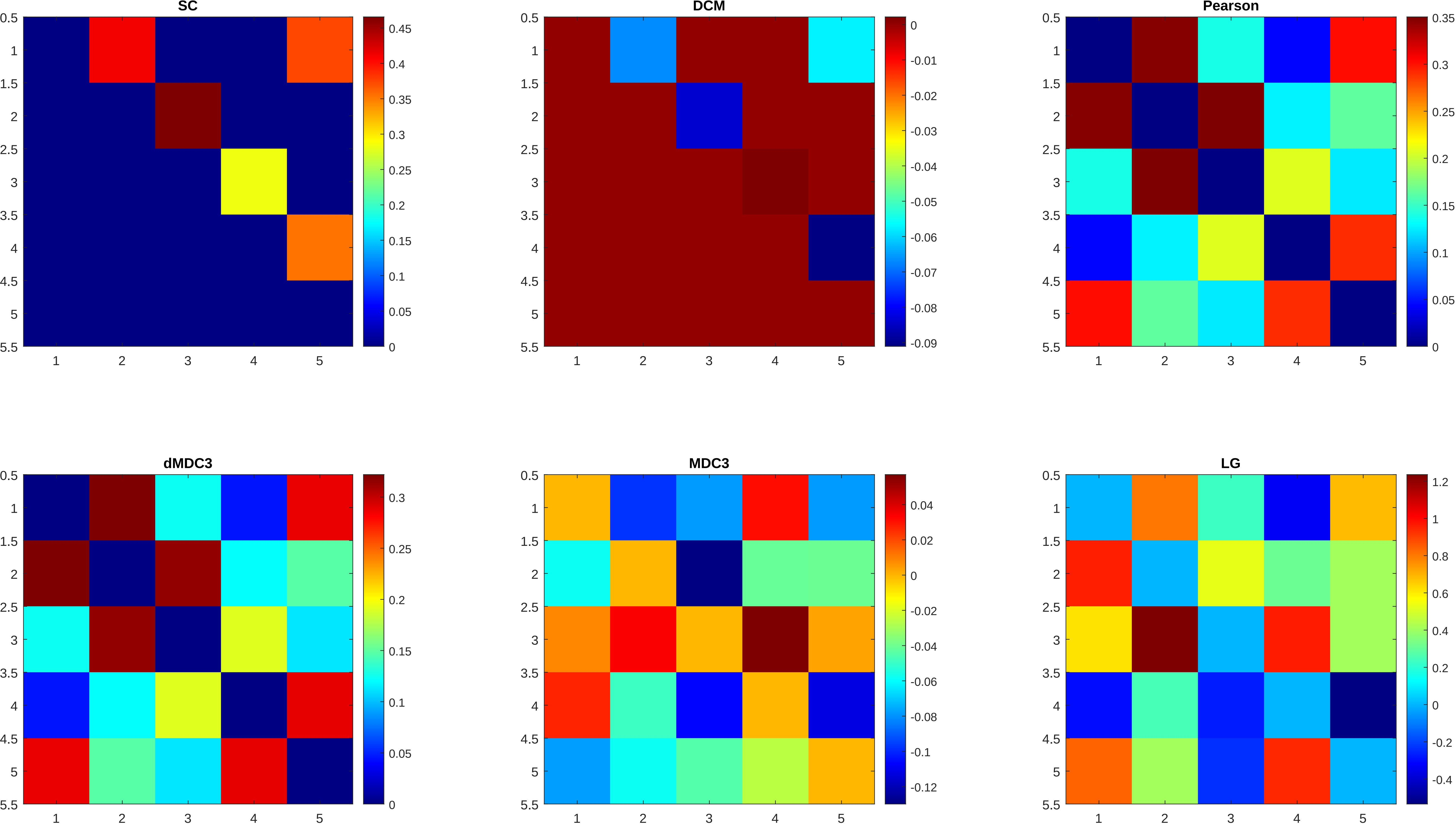
SC
DCM
Pearson
dMDC3
MDC3
LG
