# Supplementary material for: Multiscale detrended cross-correlation coefficient: estimating coupling in non-stationary neurophysiological signals
Source: Front Neurosci. 2024 Nov 13;18:1422085. doi: 10.3389/fnins.2024.1422085 (PMC11599215; doi:10.3389/fnins.2024.1422085)
Supplement: Supplementary file 1 [file Data_Sheet_1.zip › TVB Matrices.zip!/TVB Matrices.pdf]

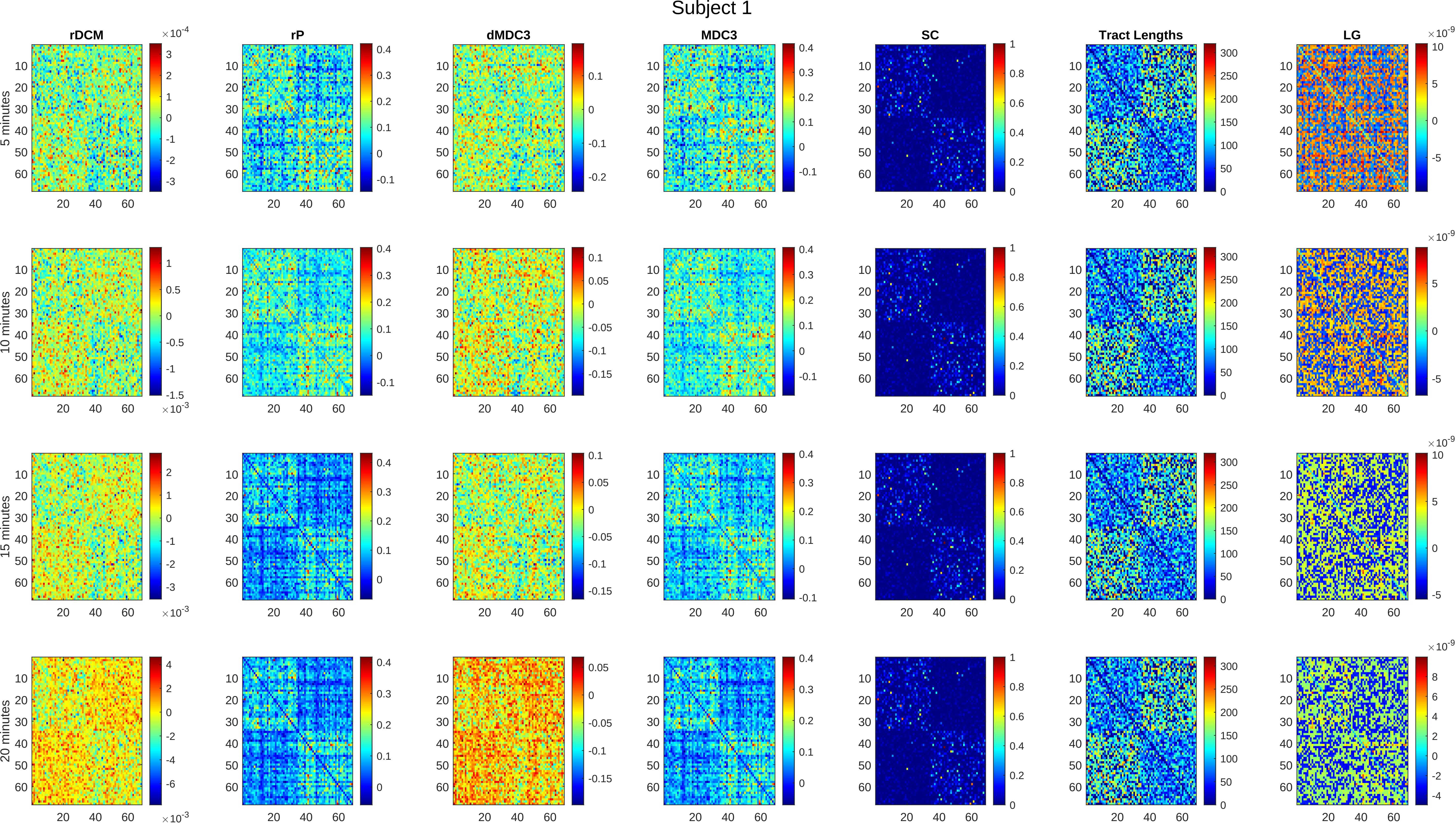

Subject 1
rDCM
rP
dMDC3
MDC3
SC
Tract Lengths
LG
5 minutes
10 minutes
15 minutes
20 minutes

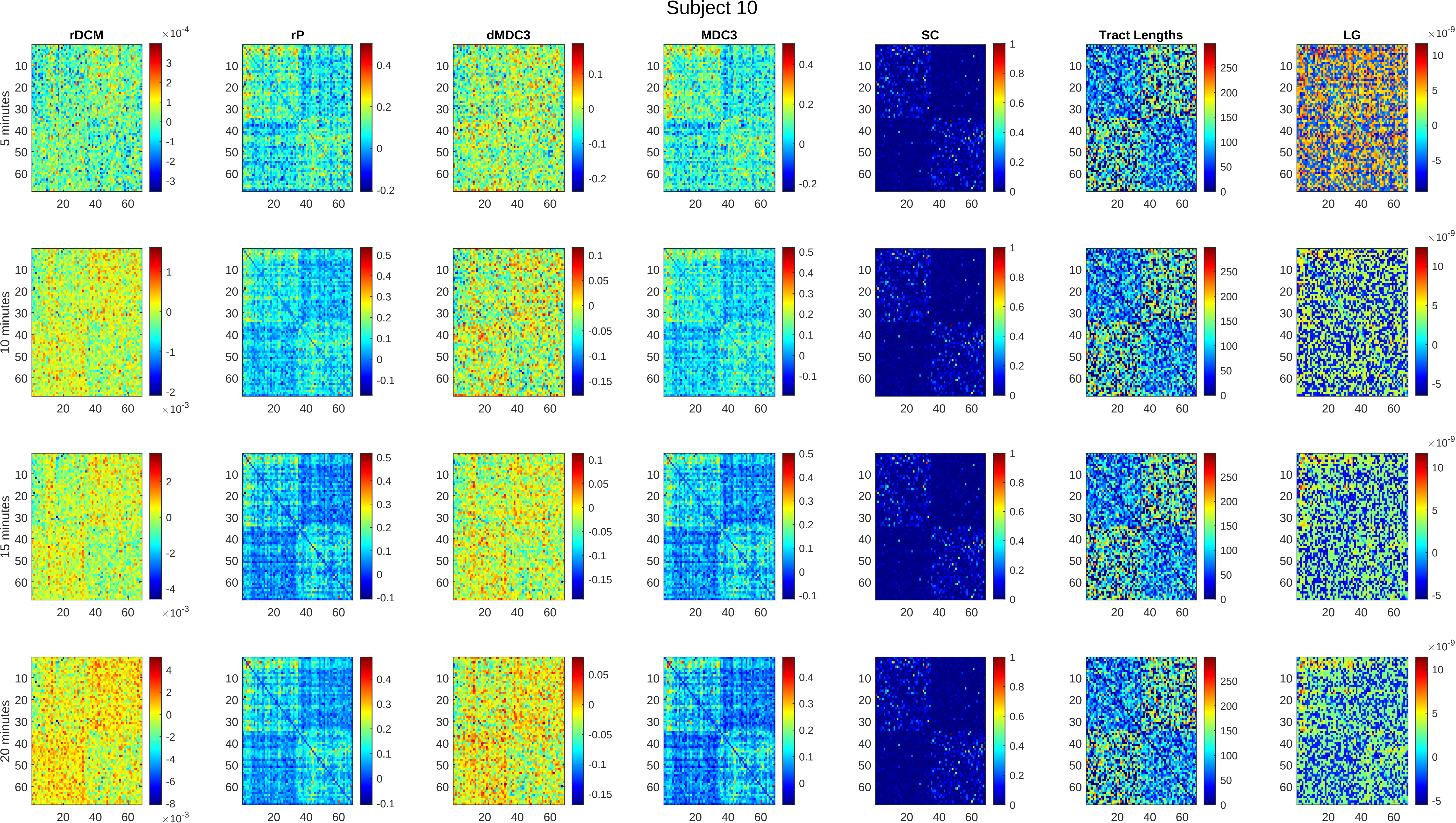
Subject 10
rDCM
rP
dMDC3
MDC3
SC
Tract Lengths
LG
5 minutes
10 minutes
15 minutes
20 minutes

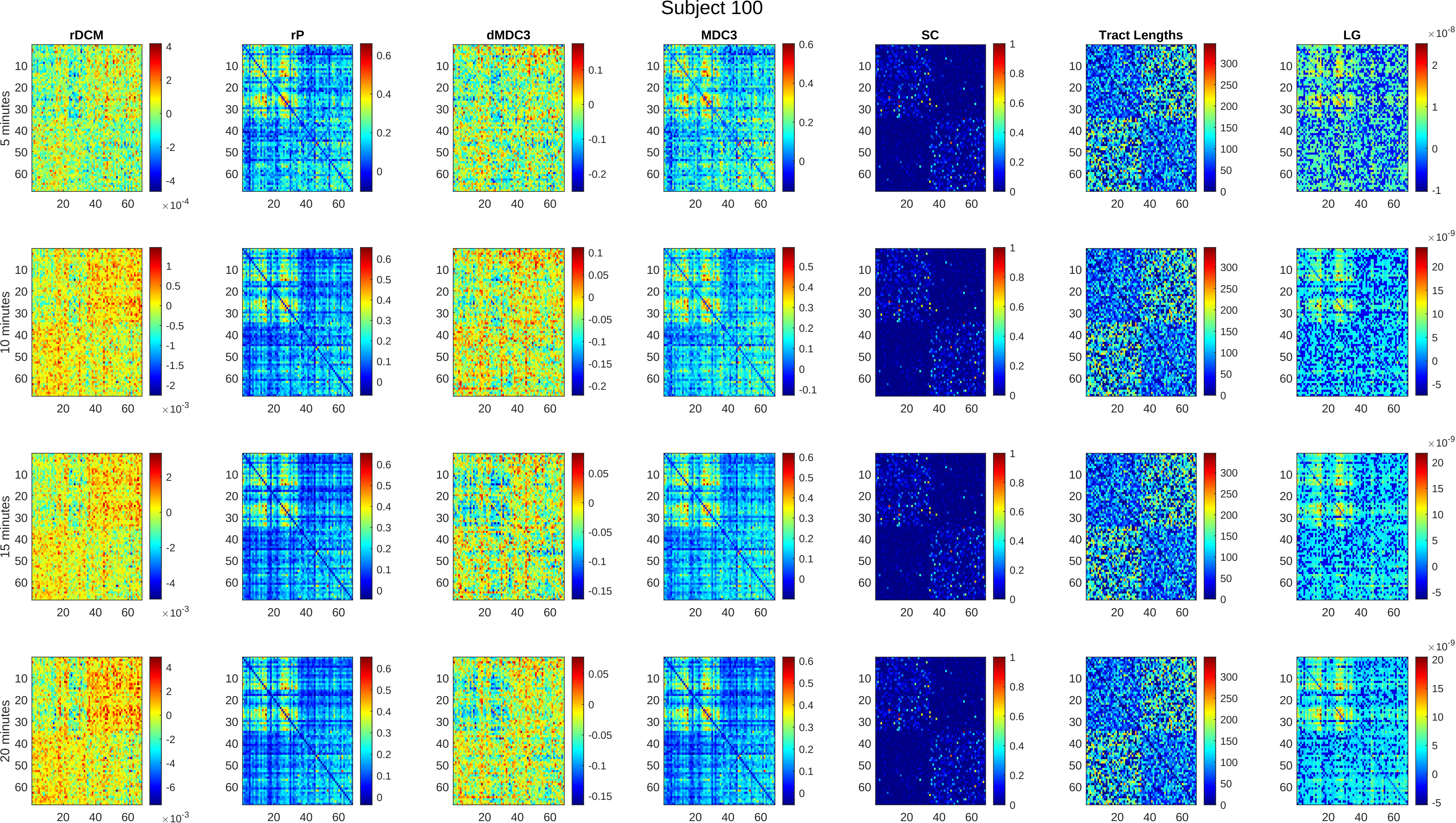
Subject 100
rDCM
rP
dMDC3
MDC3
SC
Tract Lengths
LG
5 minutes
10 minutes
15 minutes
20 minutes
×10^-4
×10^-3
×10^-3
×10^-3
×10^-8
×10^-9
×10^-9
×10^-9

Subject 11

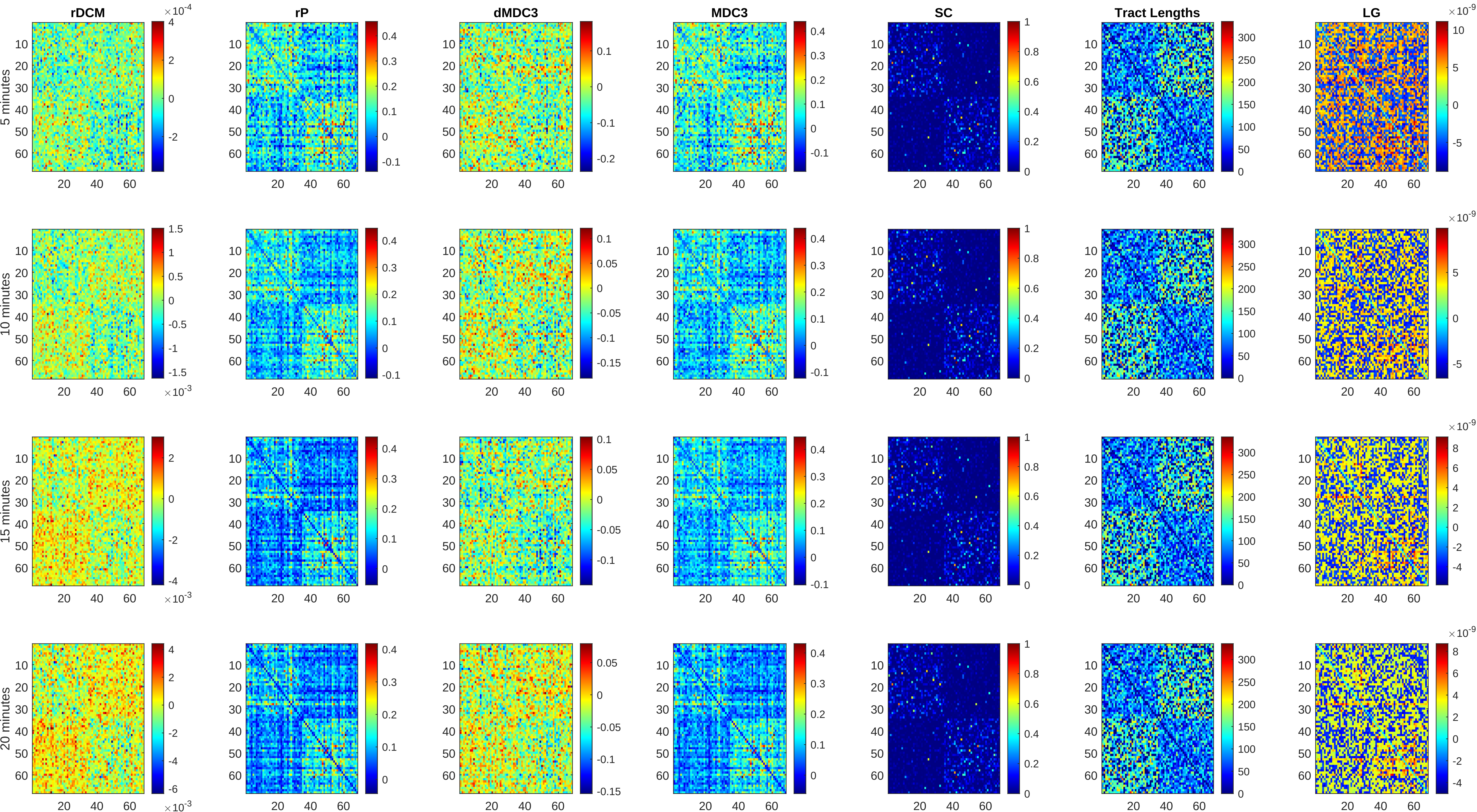

rDCM
rP
dMDC3
MDC3
SC
Tract Lengths
LG
5 minutes
10 minutes
15 minutes
20 minutes

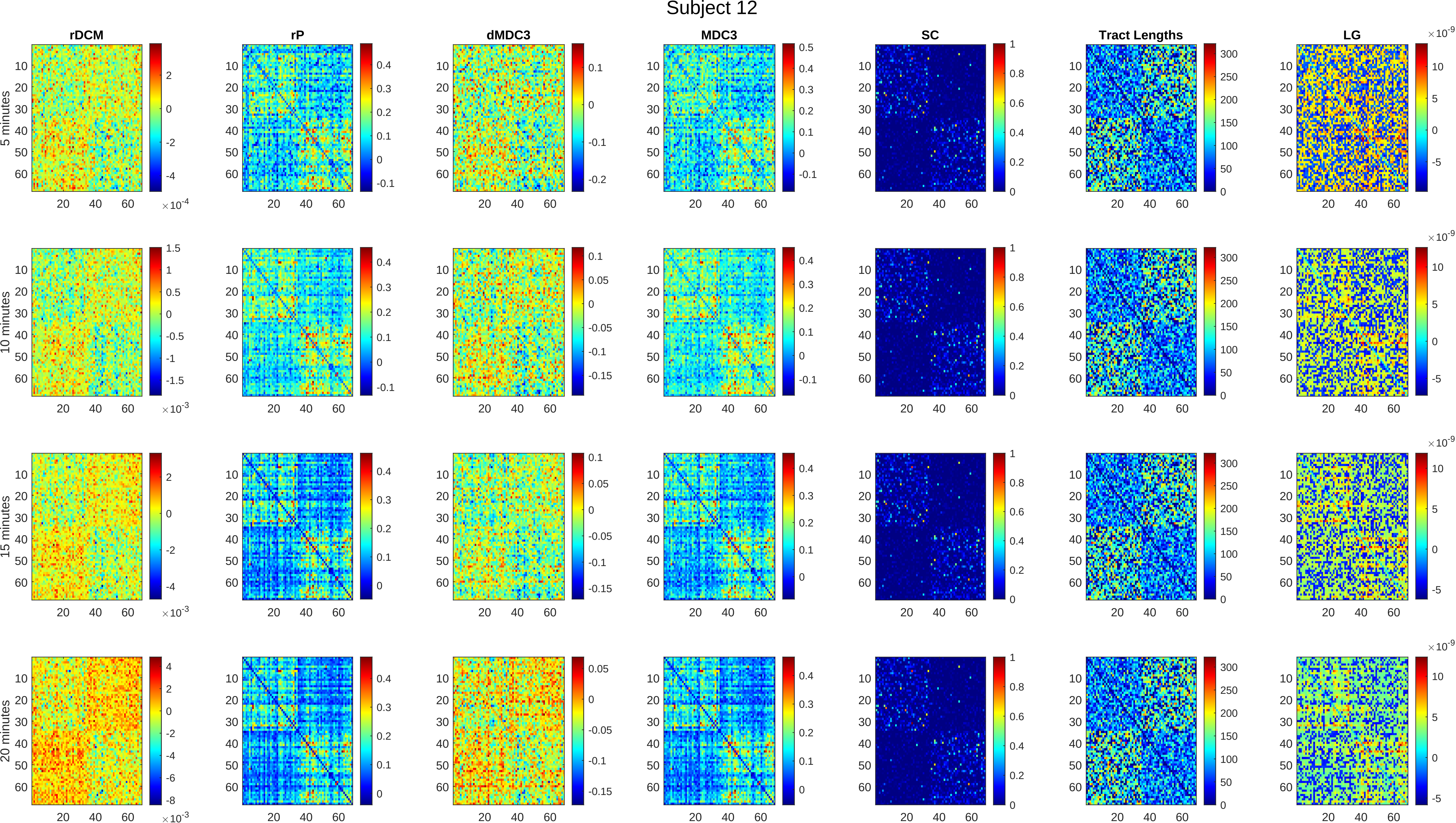

Subject 12
rDCM
rP
dMDC3
MDC3
SC
Tract Lengths
LG
5 minutes
10 minutes
15 minutes
20 minutes

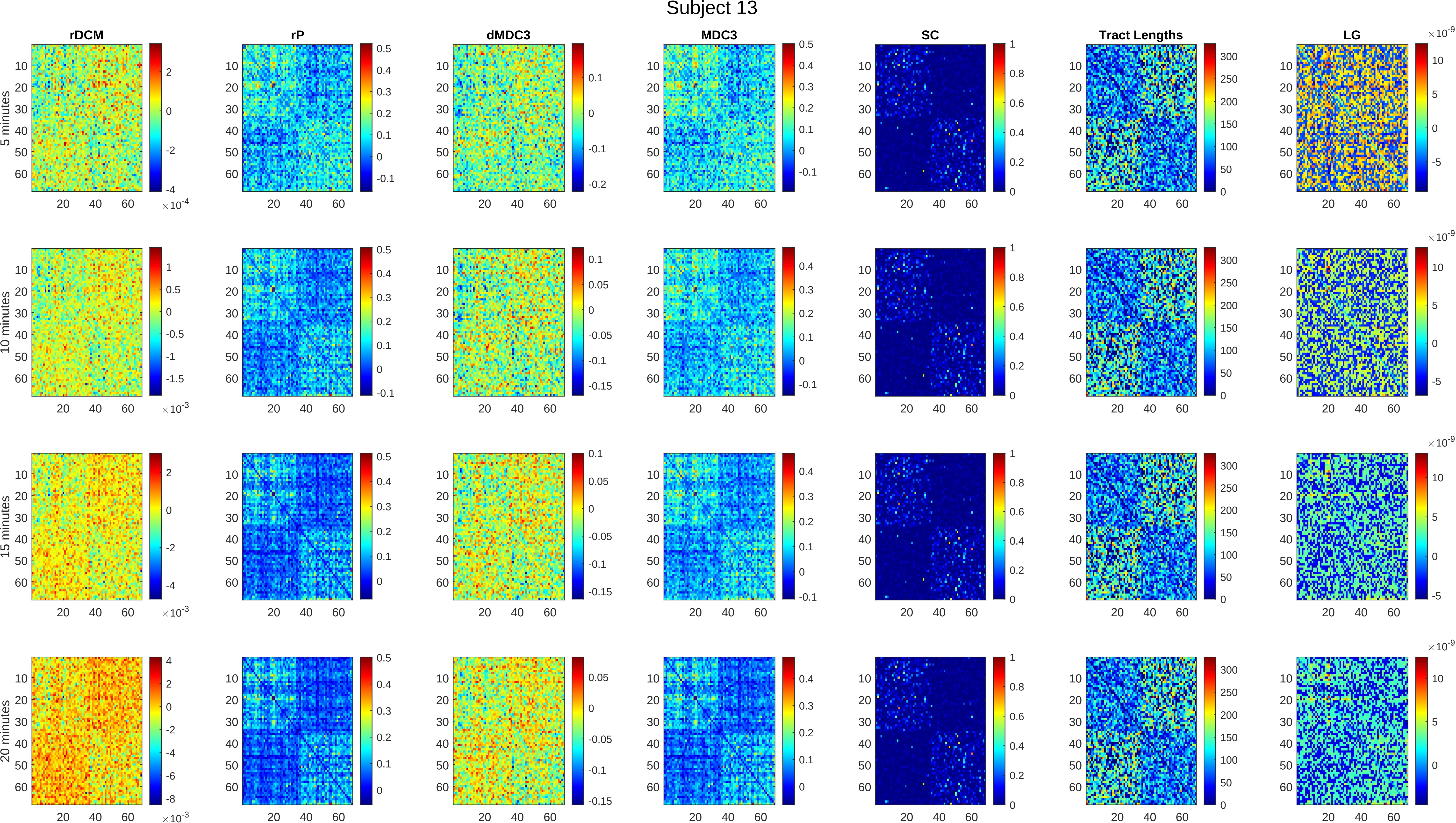

Subject 13
rDCM
rP
dMDC3
MDC3
SC
Tract Lengths
LG
5 minutes
10 minutes
15 minutes
20 minutes

Subject 14

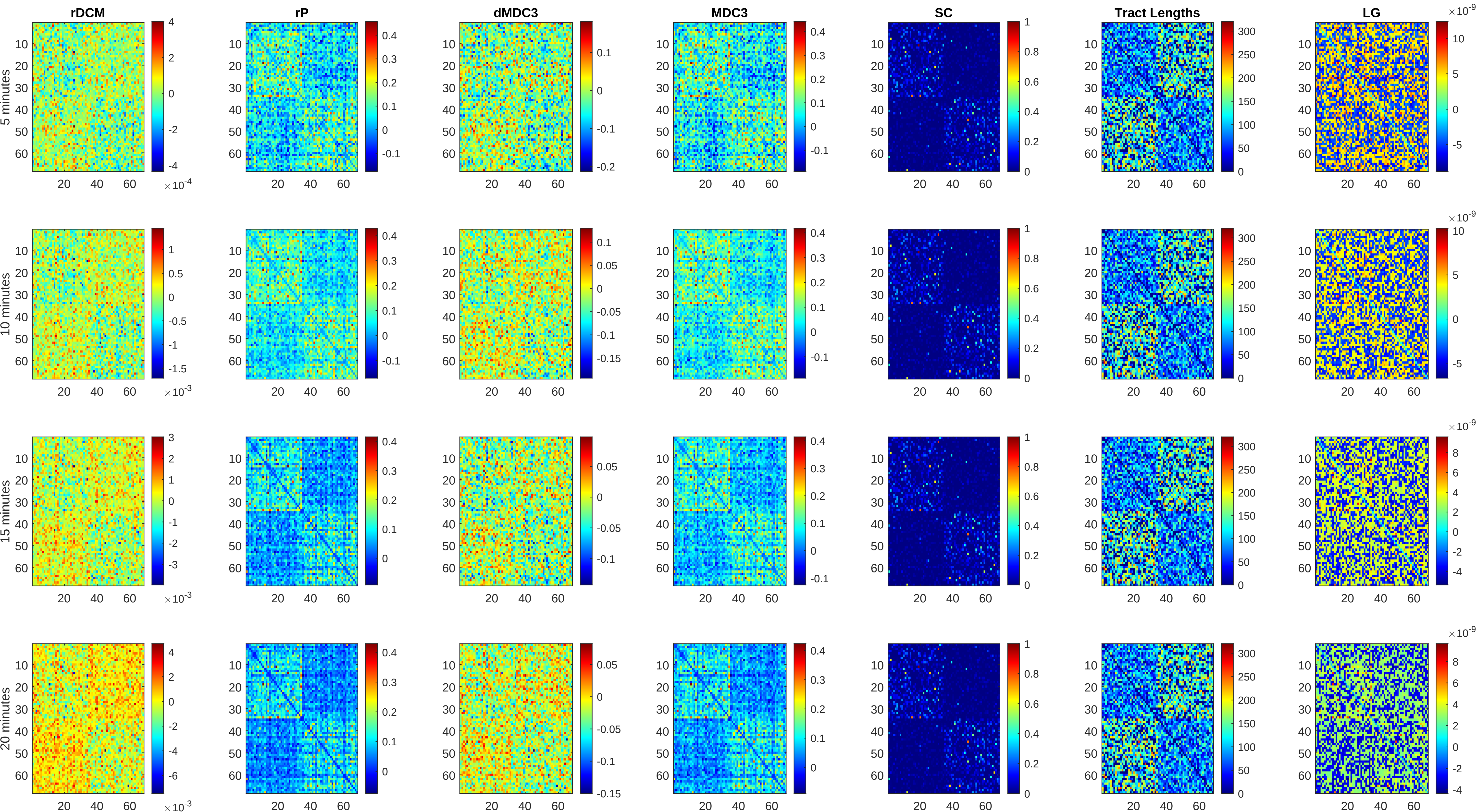

rDCM
rP
dMDC3
MDC3
SC
Tract Lengths
LG
5 minutes
10 minutes
15 minutes
20 minutes

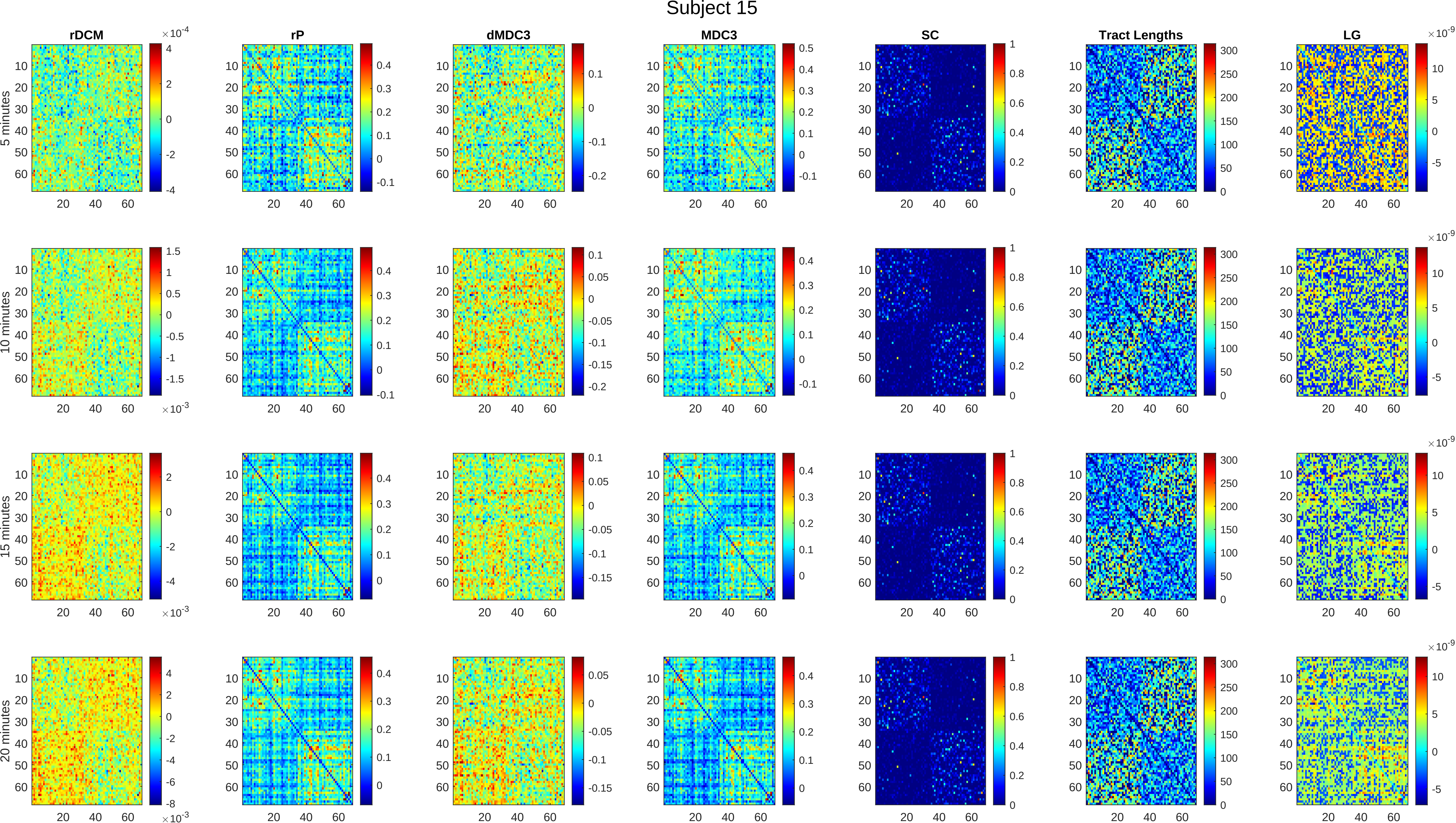

Subject 15
rDCM
rP
dMDC3
MDC3
SC
Tract Lengths
LG
5 minutes
10 minutes
15 minutes
20 minutes

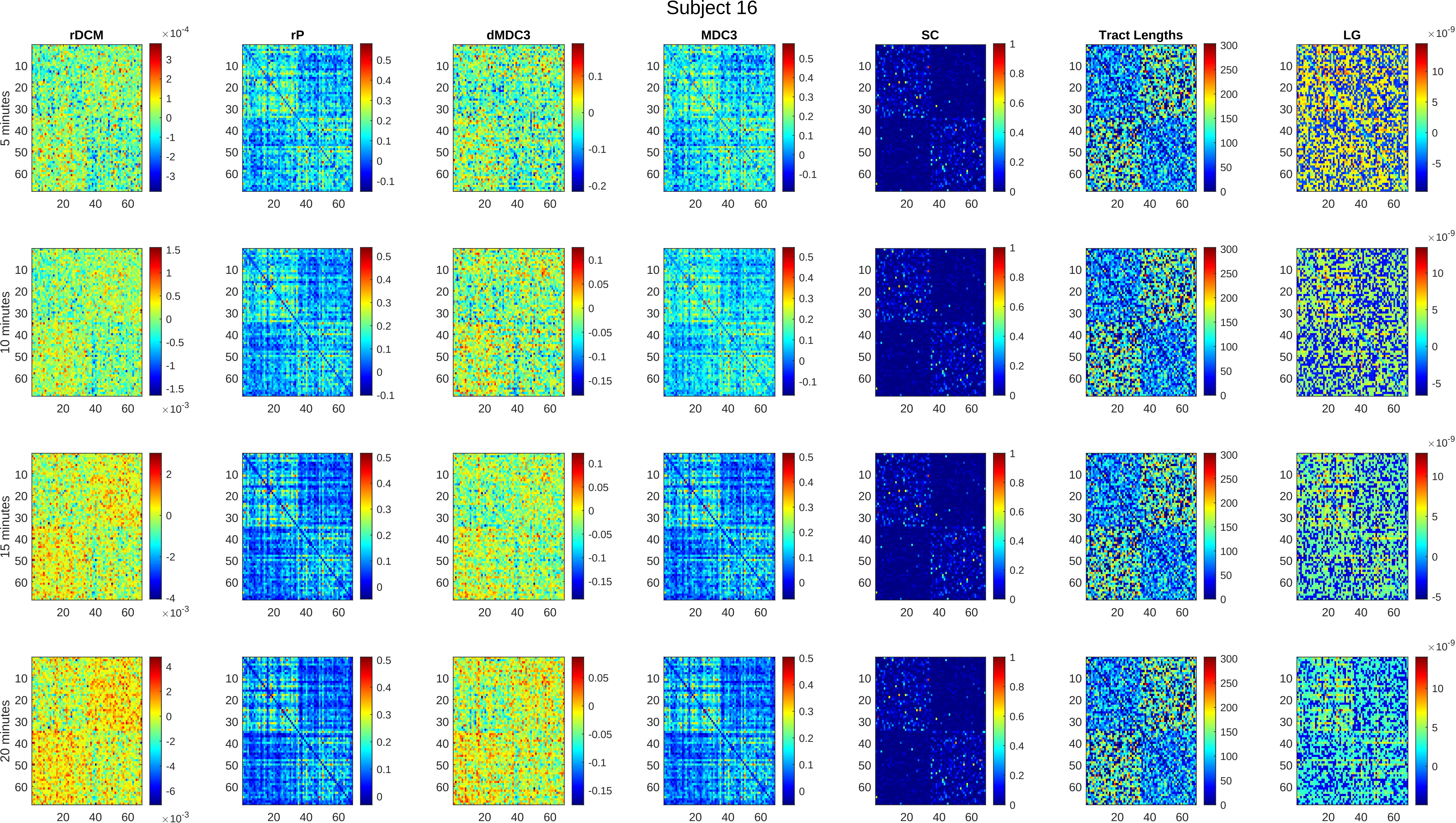

Subject 16
rDCM
rP
dMDC3
MDC3
SC
Tract Lengths
LG
5 minutes
10 minutes
15 minutes
20 minutes

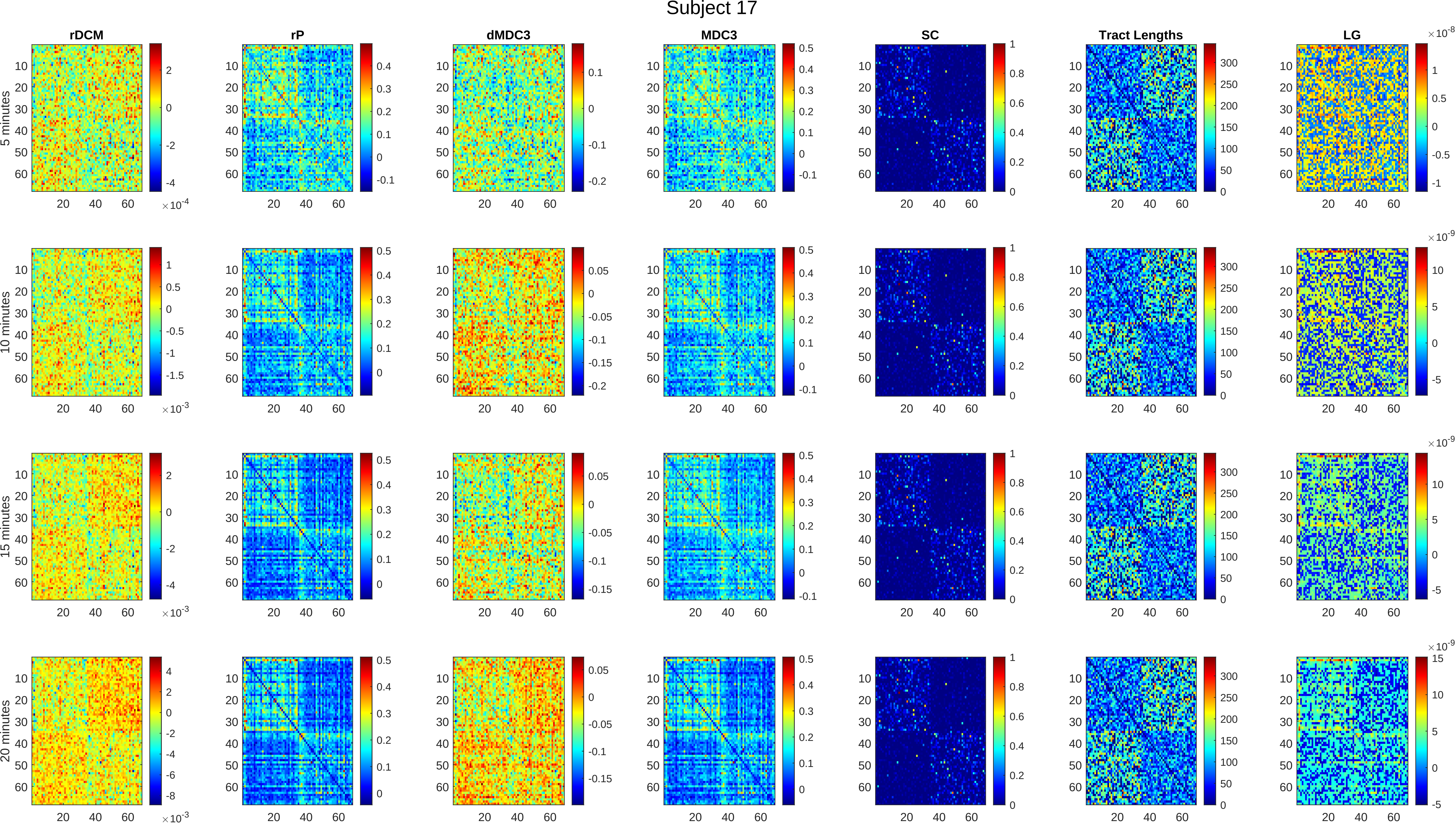

Subject 17
rDCM
rP
dMDC3
MDC3
SC
Tract Lengths
LG
5 minutes
10 minutes
15 minutes
20 minutes

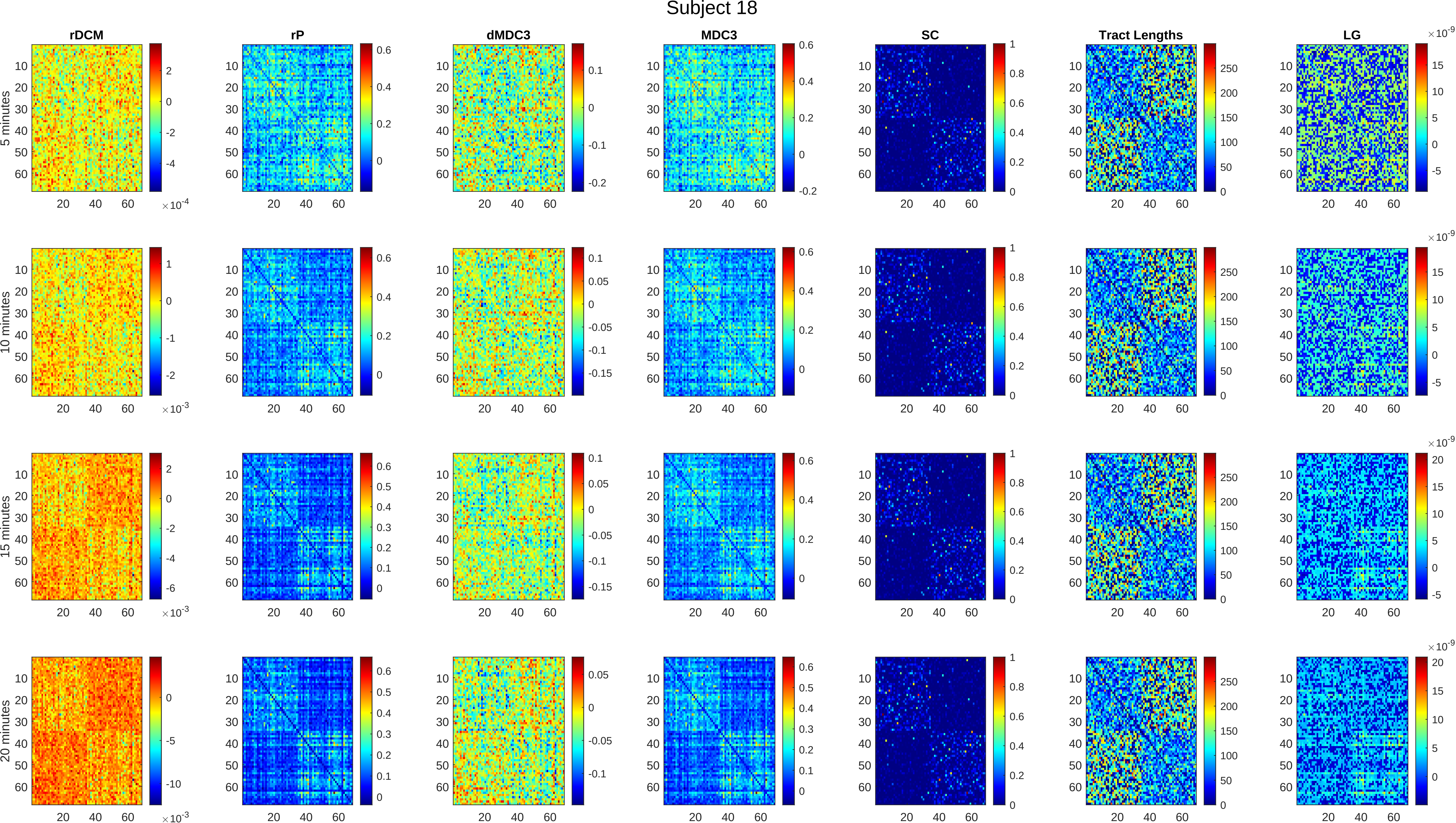

Subject 18
rDCM
rP
dMDC3
MDC3
SC
Tract Lengths
LG
5 minutes
10 minutes
15 minutes
20 minutes

Subject 19

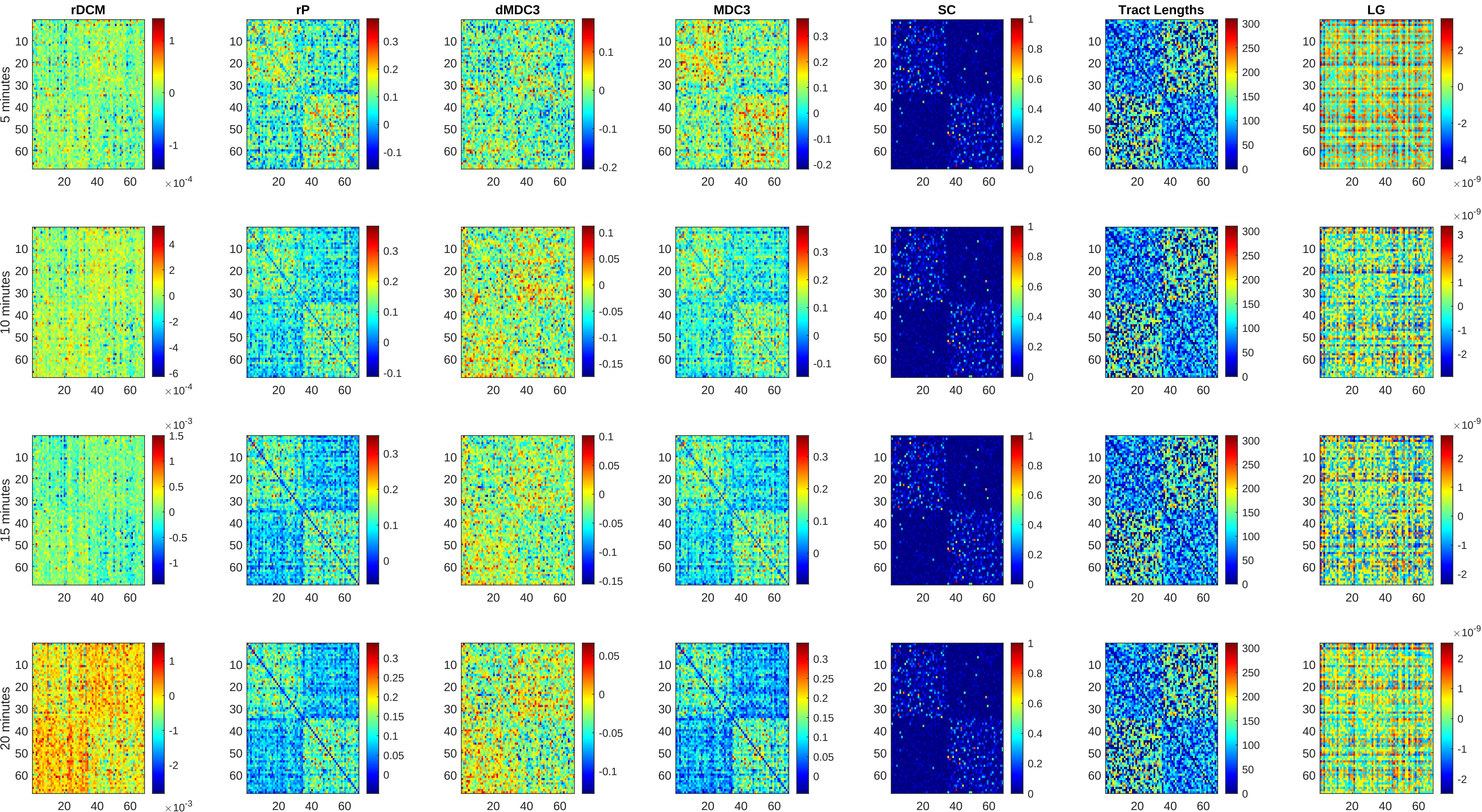

rDCM
rP
dMDC3
MDC3
SC
Tract Lengths
LG
5 minutes
10 minutes
15 minutes
20 minutes

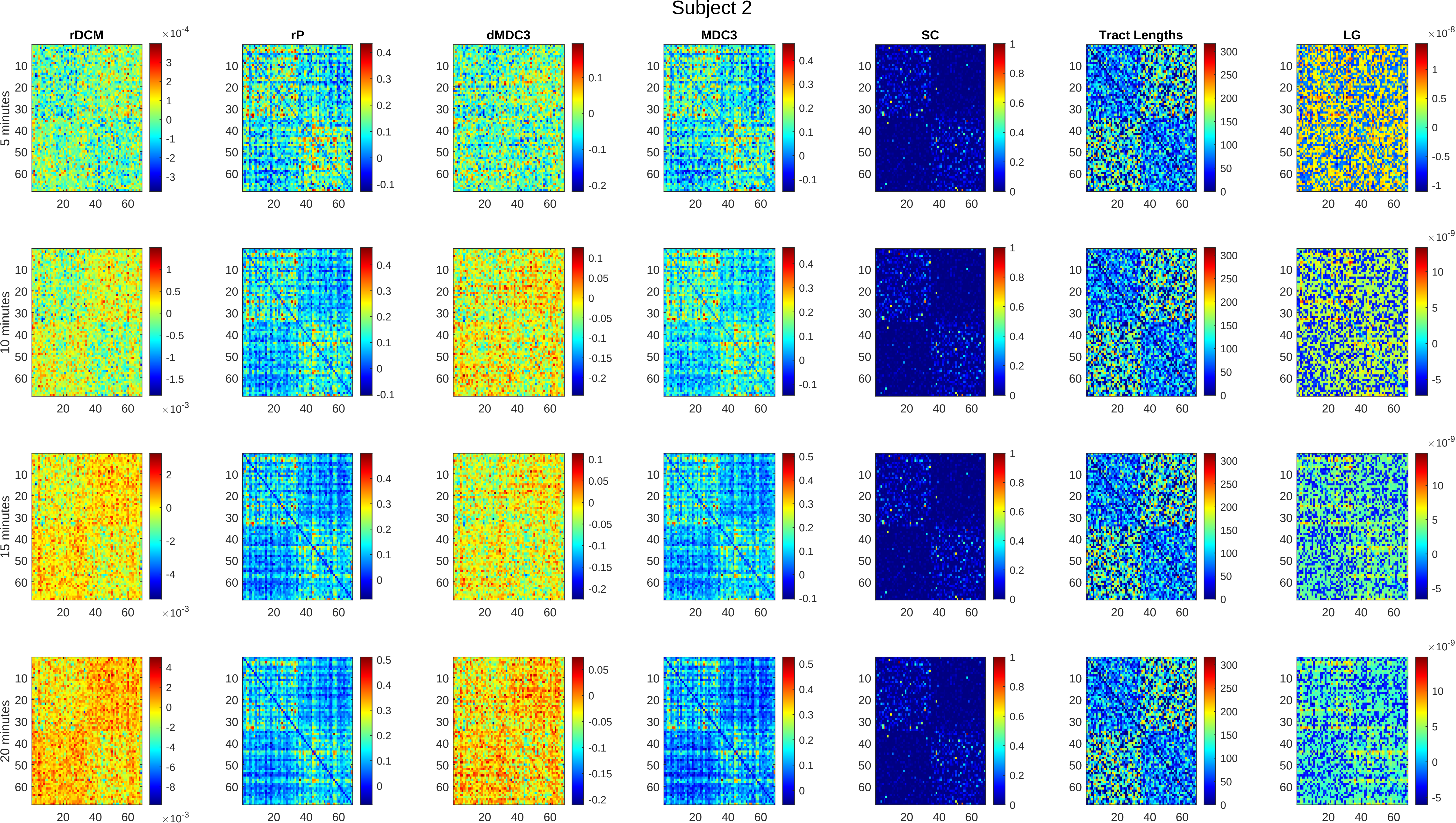

Subject 2
rDCM
rP
dMDC3
MDC3
SC
Tract Lengths
LG
5 minutes
10 minutes
15 minutes
20 minutes

Subject 20

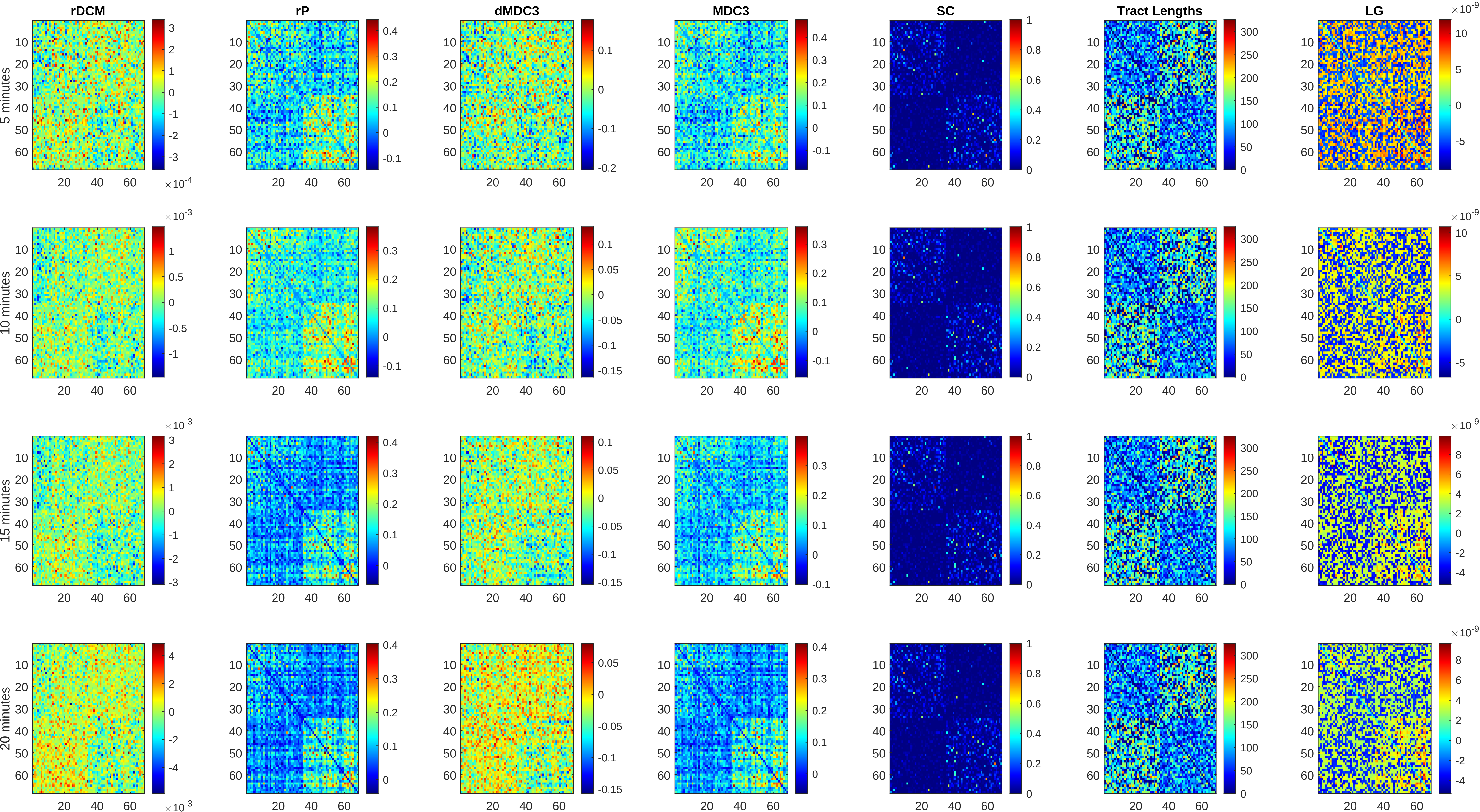

rDCM
rP
dMDC3
MDC3
SC
Tract Lengths
LG
5 minutes
10 minutes
15 minutes
20 minutes

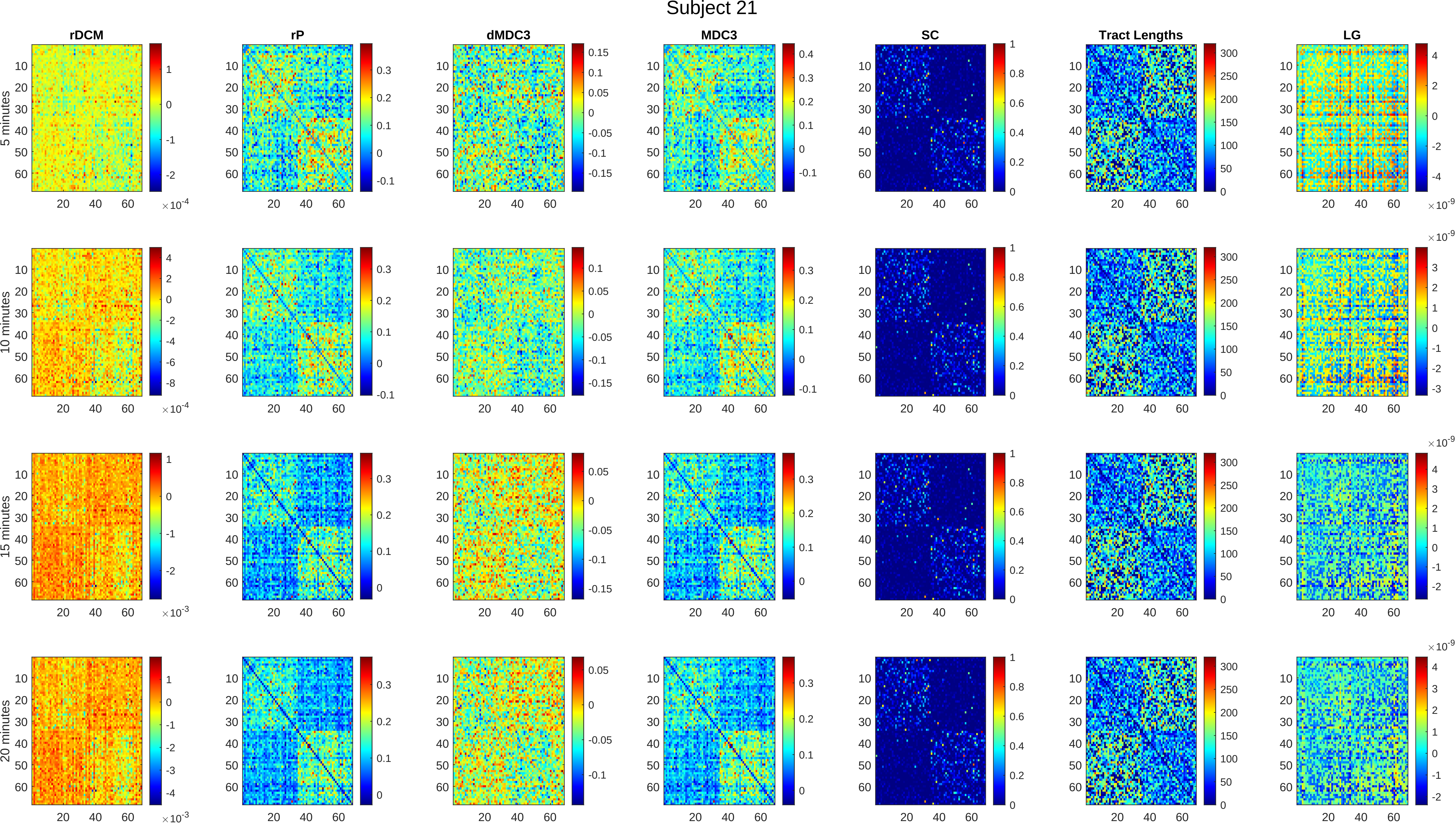
Subject 21
rDCM
rP
dMDC3
MDC3
SC
Tract Lengths
LG
5 minutes
10 minutes
15 minutes
20 minutes

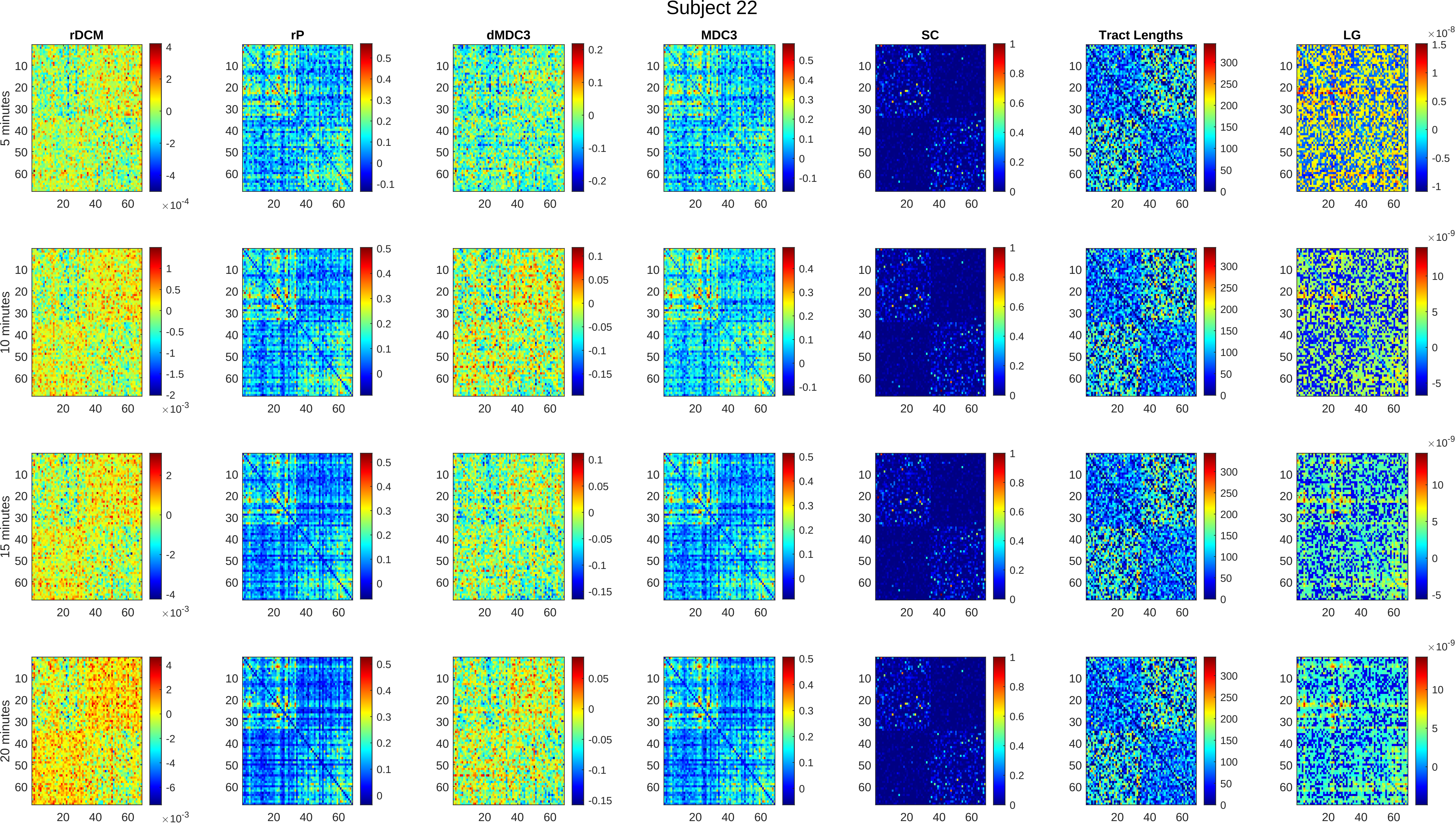
Subject 22
rDCM
rP
dMDC3
MDC3
SC
Tract Lengths
LG
5 minutes
10 minutes
15 minutes
20 minutes

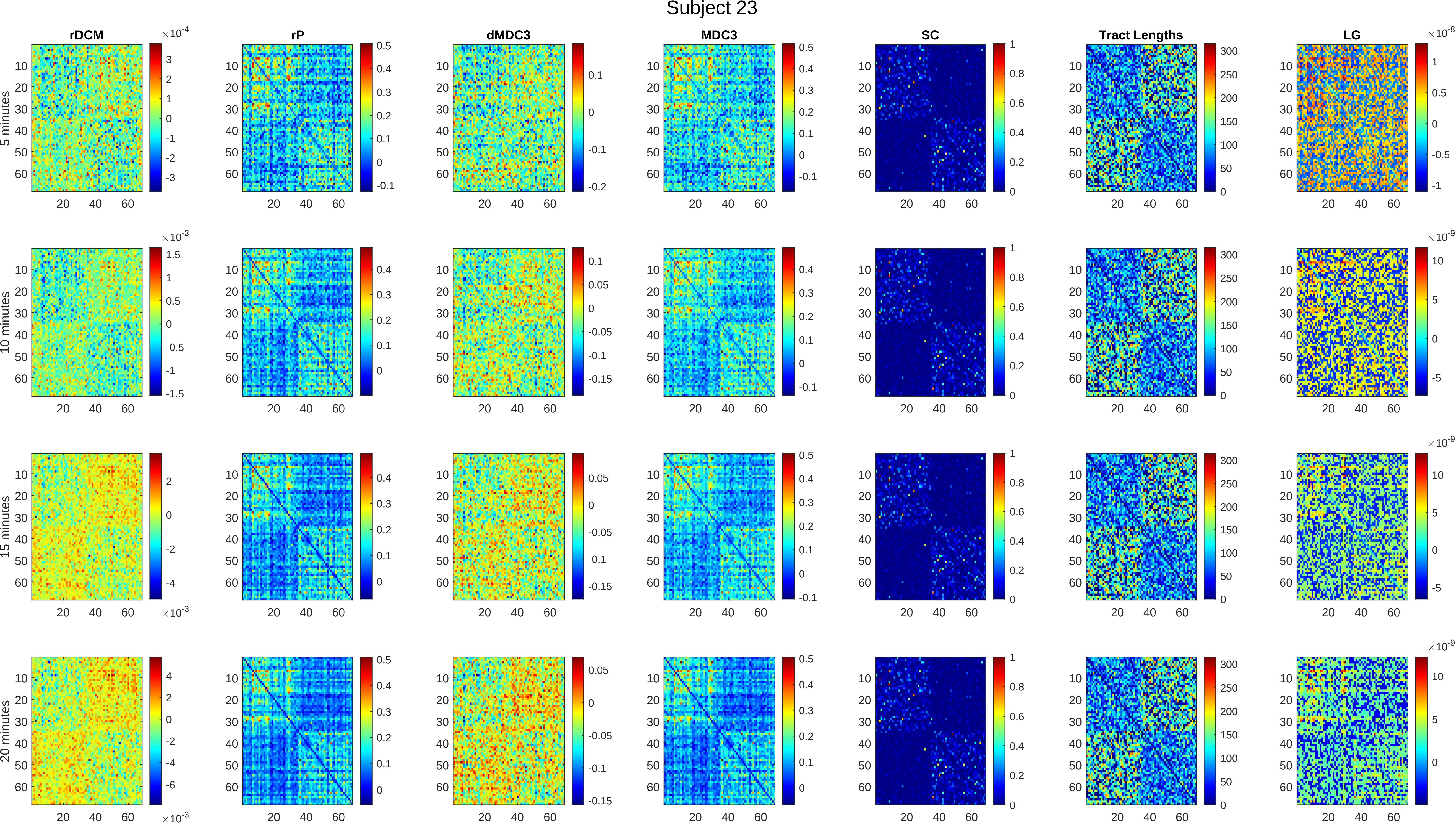
Subject 23
rDCM
rP
dMDC3
MDC3
SC
Tract Lengths
LG
5 minutes
10 minutes
15 minutes
20 minutes

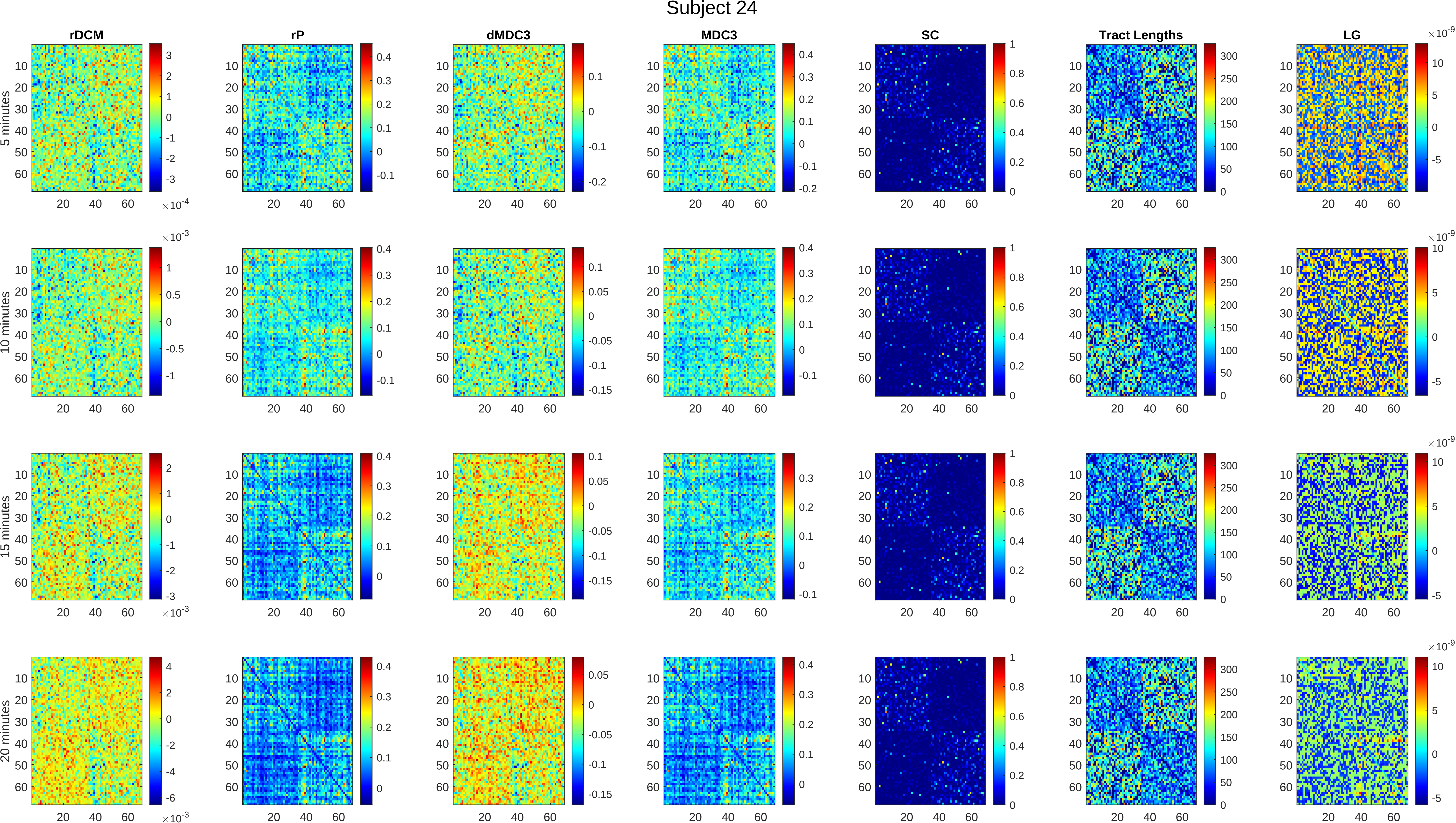

Subject 24
rDCM
rP
dMDC3
MDC3
SC
Tract Lengths
LG
5 minutes
10 minutes
15 minutes
20 minutes

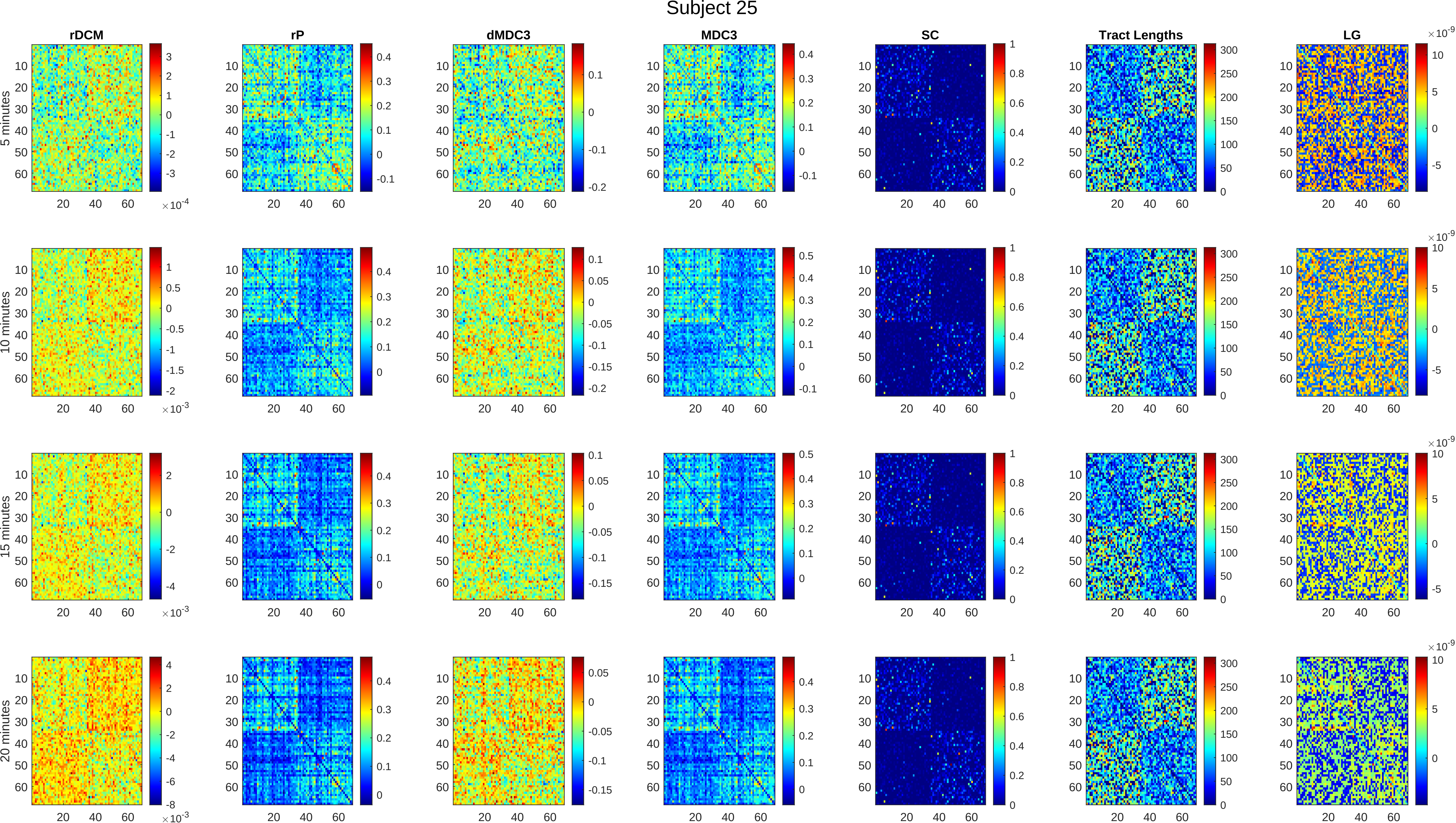
Subject 25
rDCM
rP
dMDC3
MDC3
SC
Tract Lengths
LG
5 minutes
10 minutes
15 minutes
20 minutes

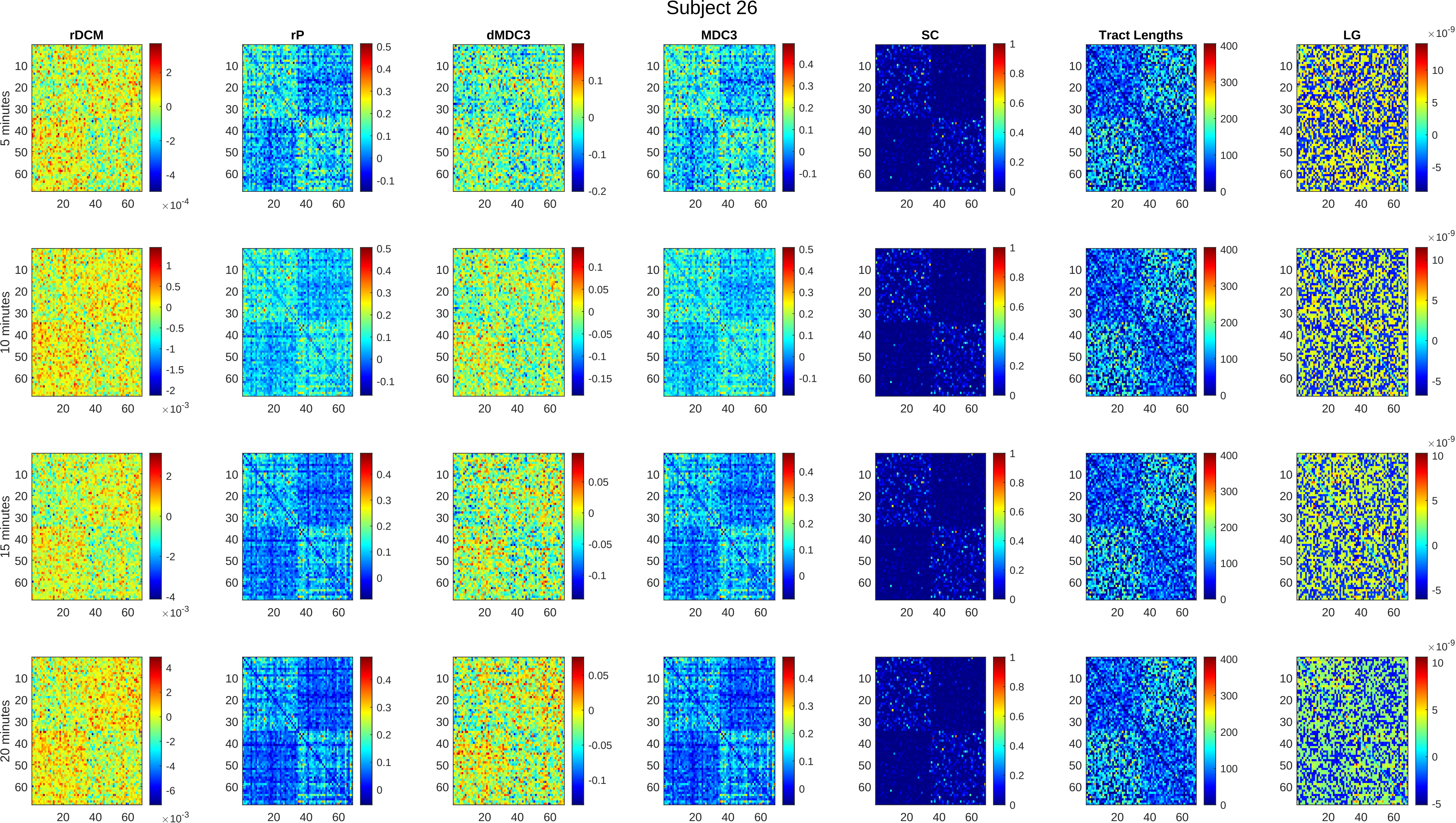
Subject 26
rDCM
rP
dMDC3
MDC3
SC
Tract Lengths
LG
5 minutes
10 minutes
15 minutes
20 minutes

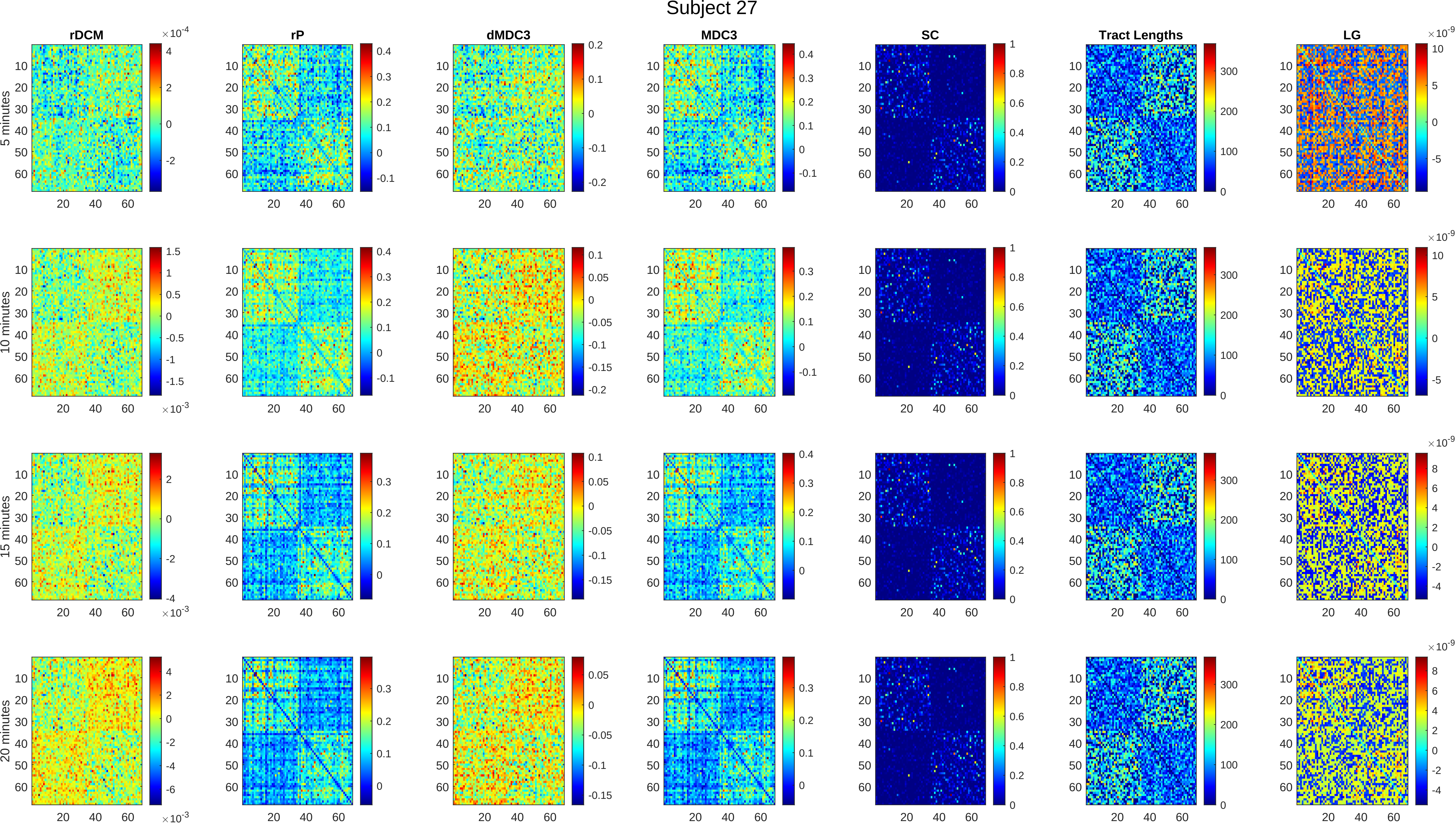

Subject 27
rDCM
rP
dMDC3
MDC3
SC
Tract Lengths
LG
5 minutes
10 minutes
15 minutes
20 minutes

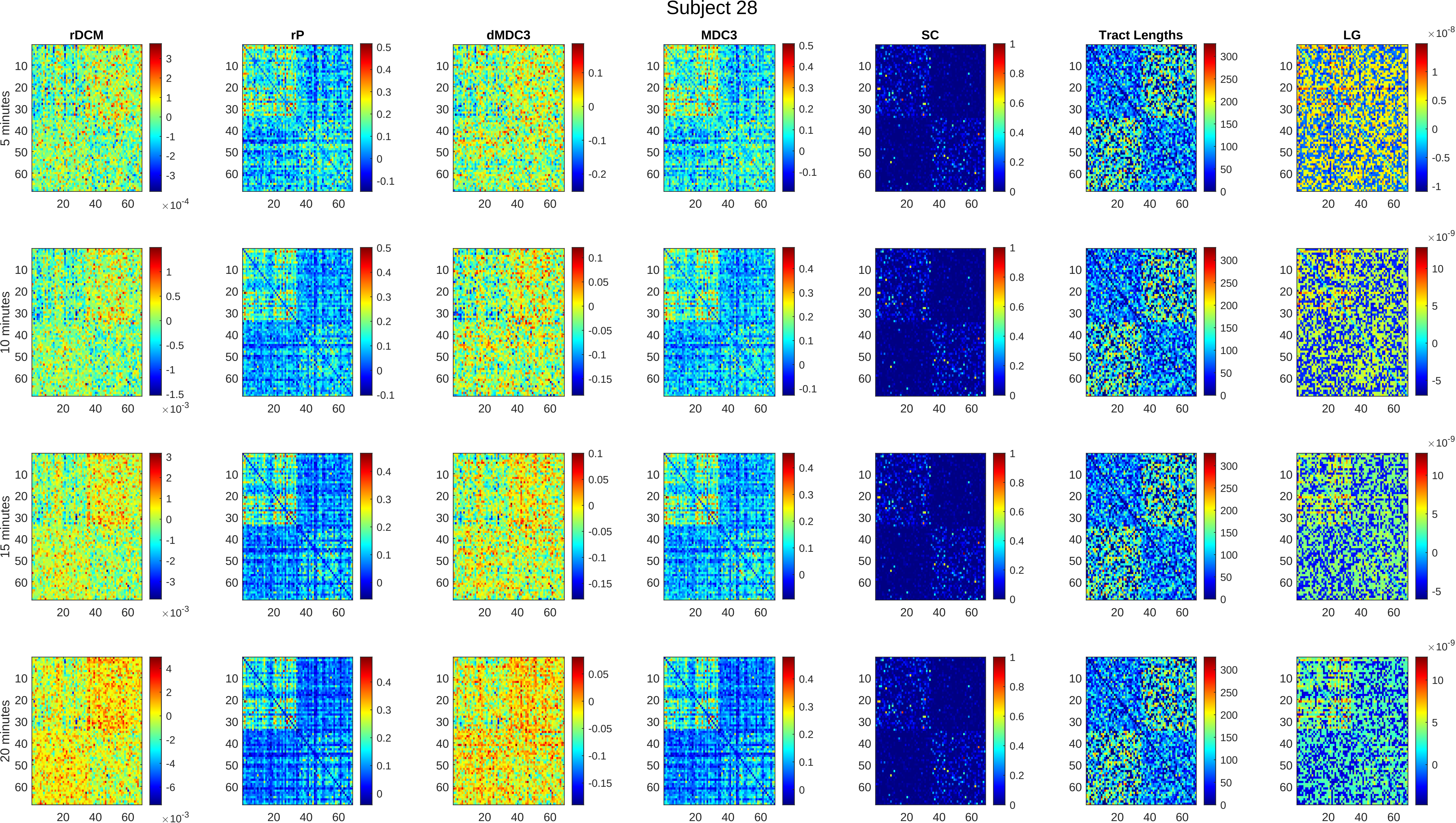

Subject 28
rDCM
rP
dMDC3
MDC3
SC
Tract Lengths
LG
5 minutes
10 minutes
15 minutes
20 minutes

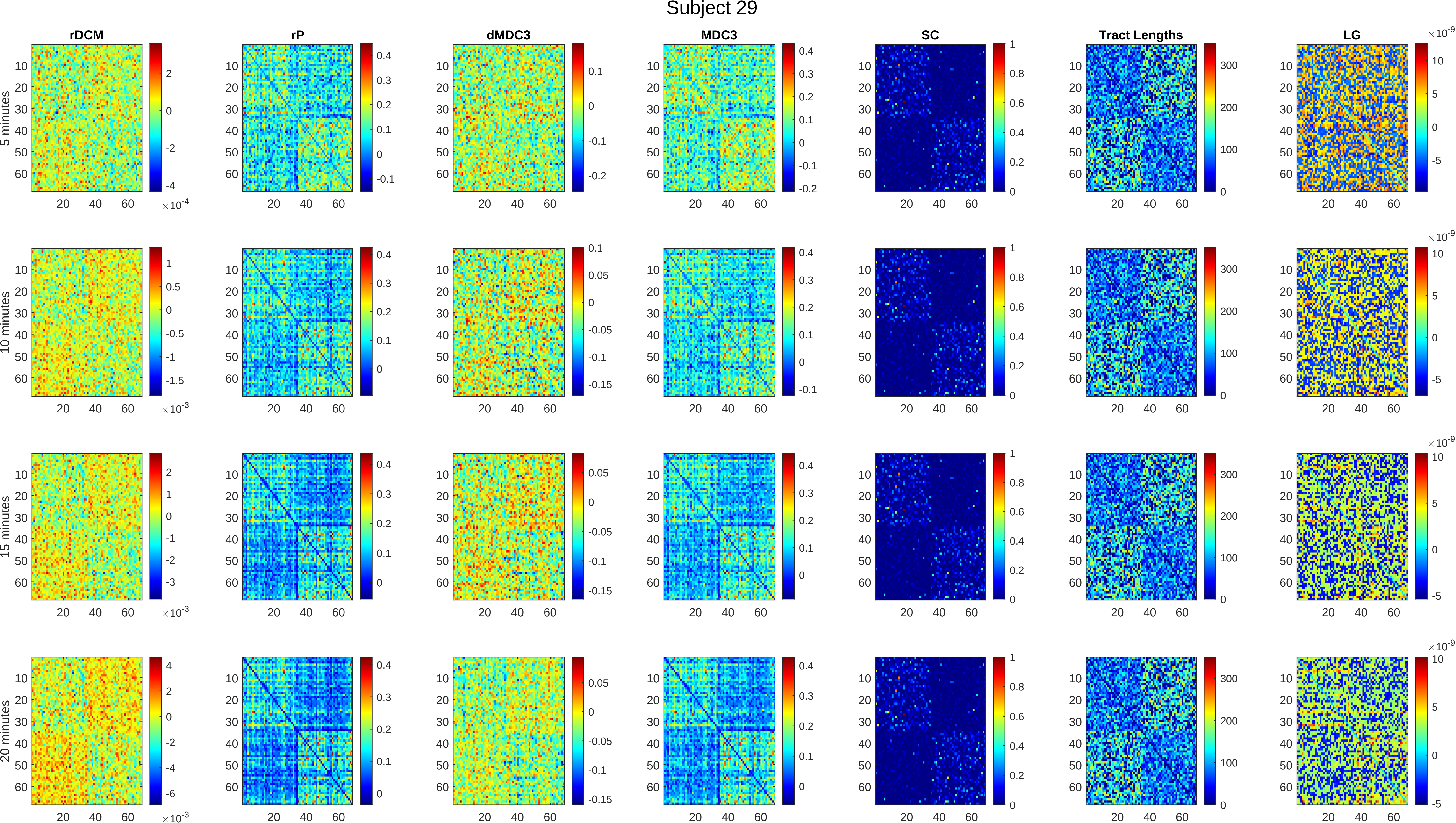

Subject 29
rDCM
rP
dMDC3
MDC3
SC
Tract Lengths
LG
5 minutes
10 minutes
15 minutes
20 minutes

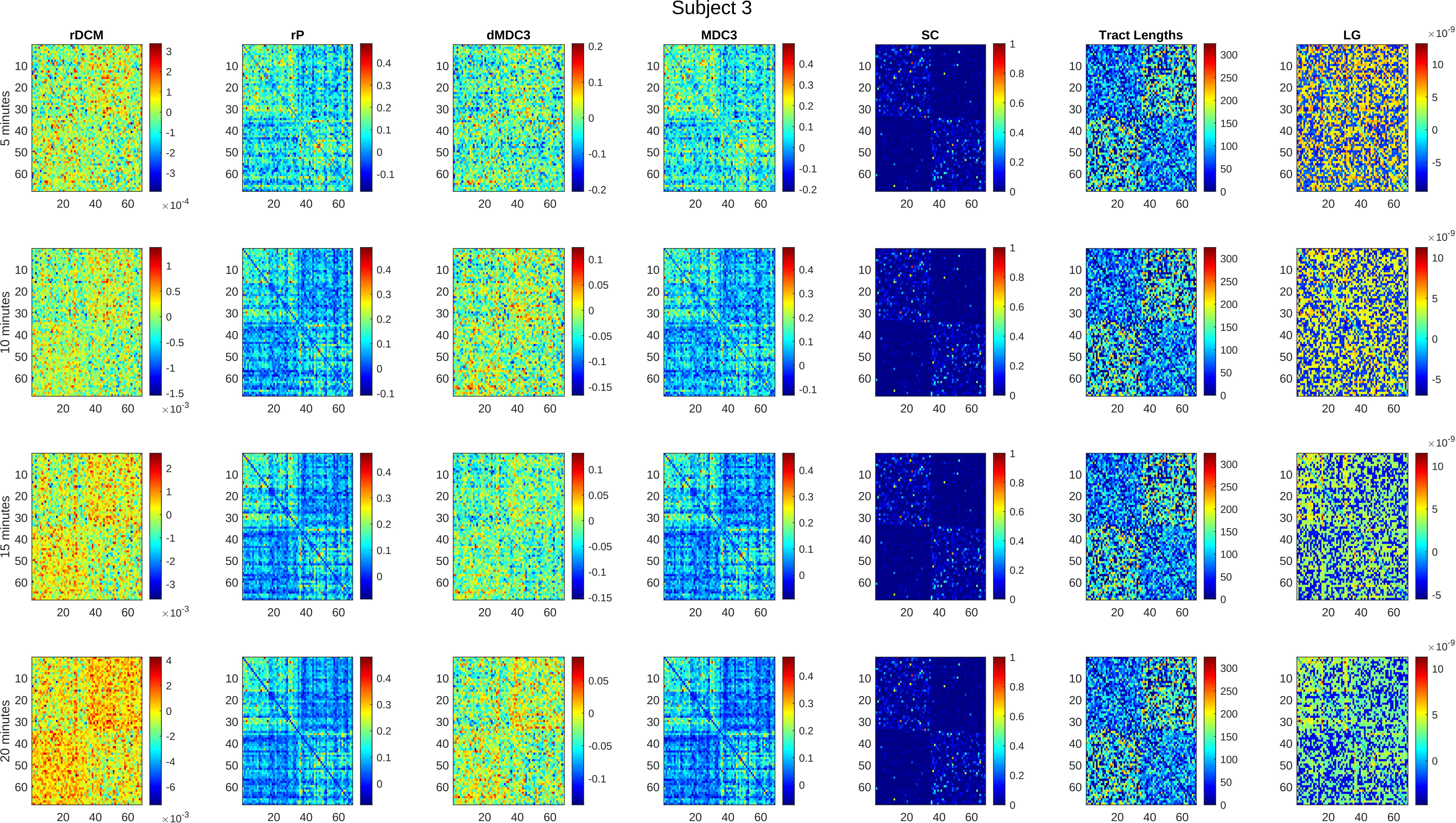

Subject 3
rDCM
rP
dMDC3
MDC3
SC
Tract Lengths
LG
5 minutes
10 minutes
15 minutes
20 minutes

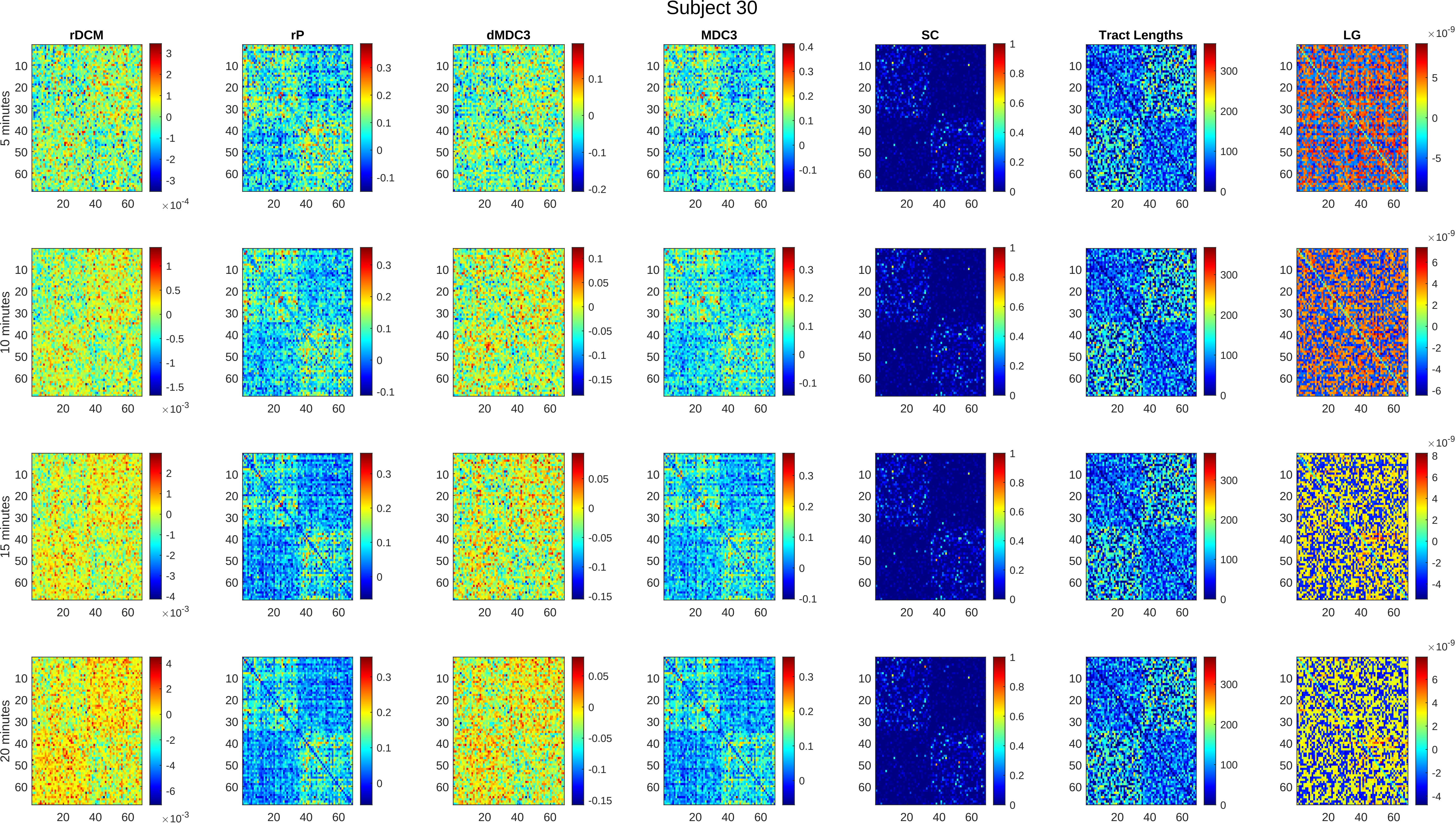
Subject 30
rDCM
rP
dMDC3
MDC3
SC
Tract Lengths
LG
5 minutes
10 minutes
15 minutes
20 minutes

Subject 31

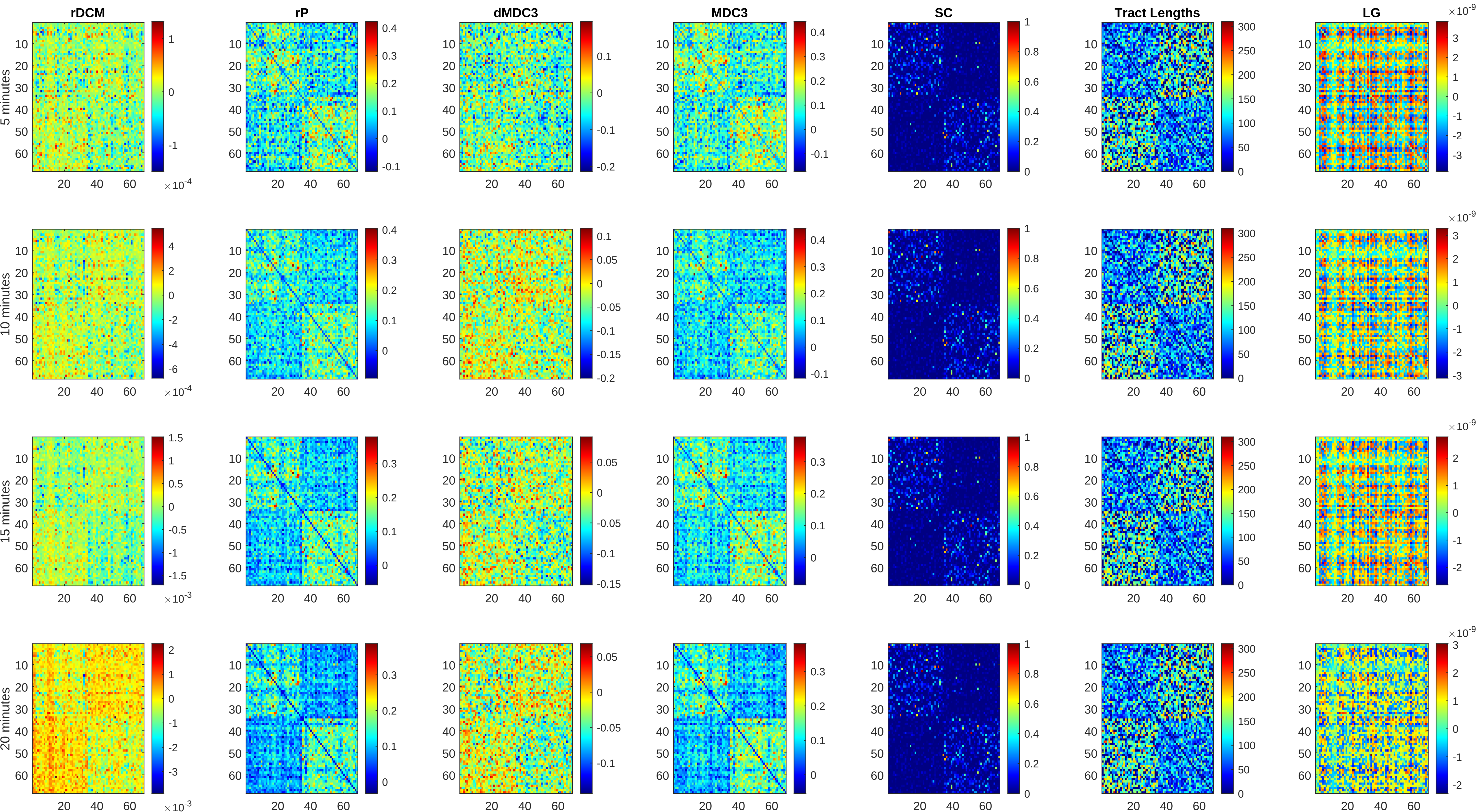

rDCM
rP
dMDC3
MDC3
SC
Tract Lengths
LG
5 minutes
10 minutes
15 minutes
20 minutes

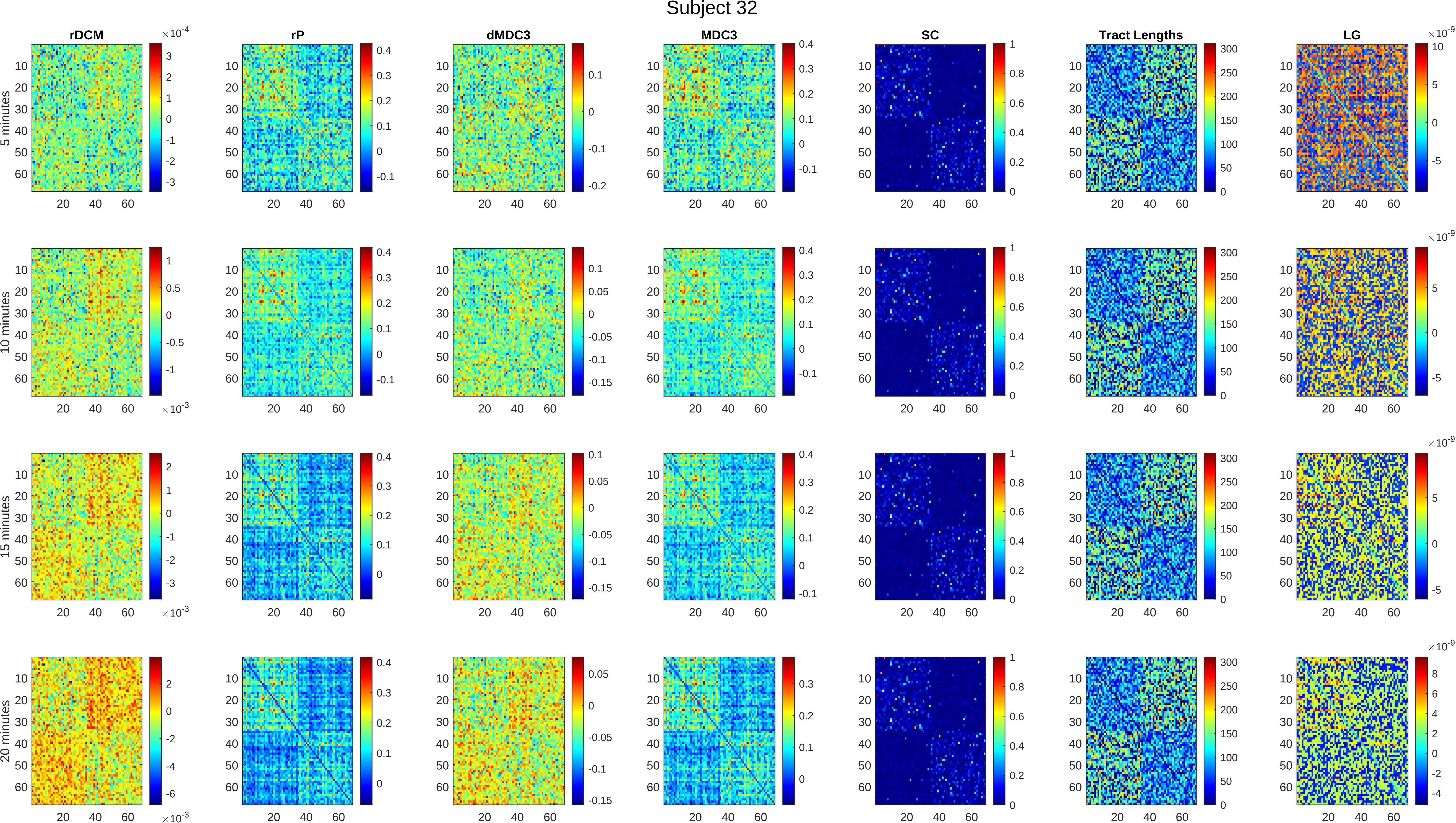
Subject 32
rDCM
rP
dMDC3
MDC3
SC
Tract Lengths
LG
5 minutes
10 minutes
15 minutes
20 minutes

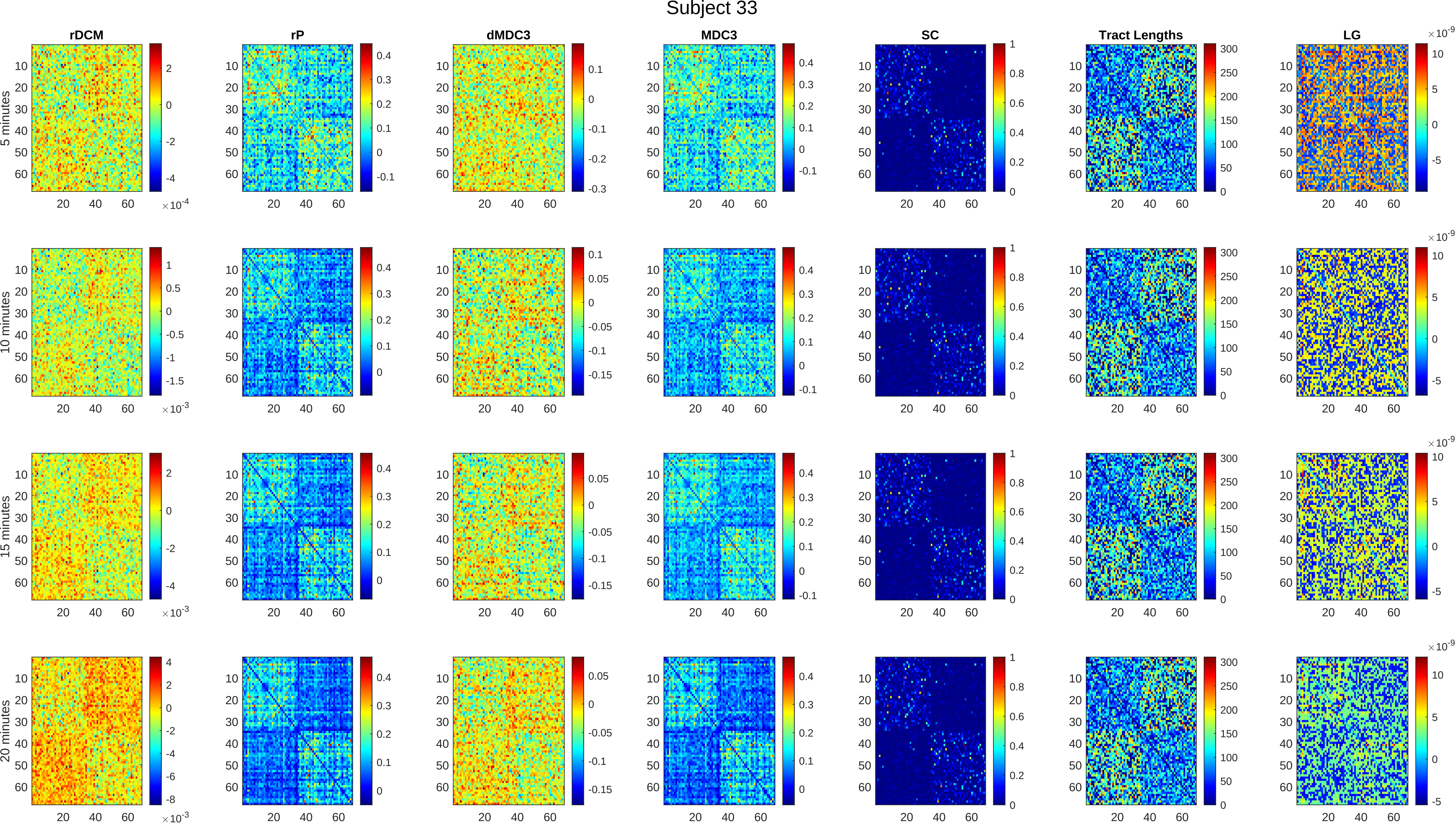
Subject 33
rDCM
rP
dMDC3
MDC3
SC
Tract Lengths
LG
5 minutes
10 minutes
15 minutes
20 minutes

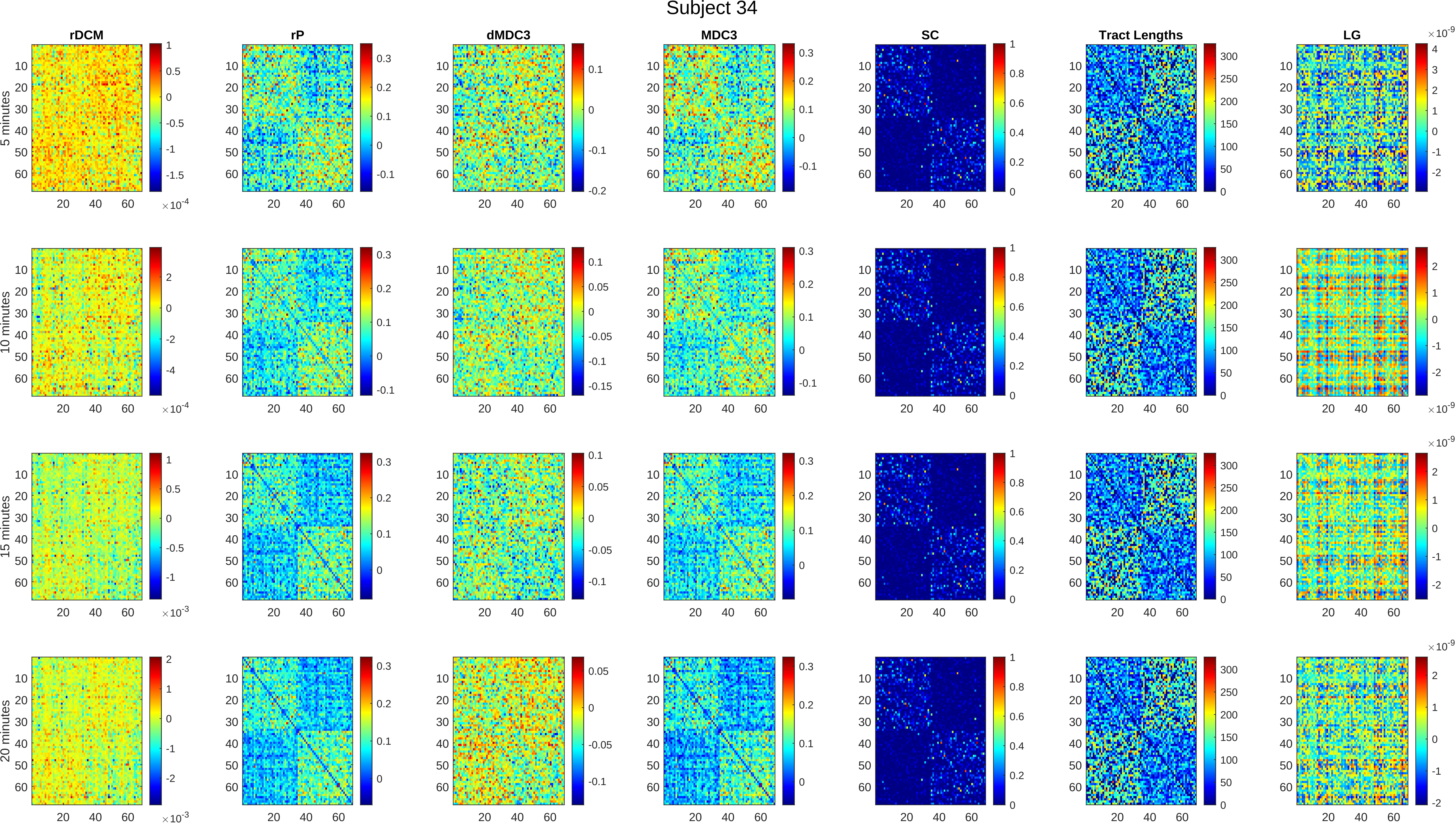
Subject 34
rDCM
rP
dMDC3
MDC3
SC
Tract Lengths
LG
5 minutes
10 minutes
15 minutes
20 minutes

Subject 35

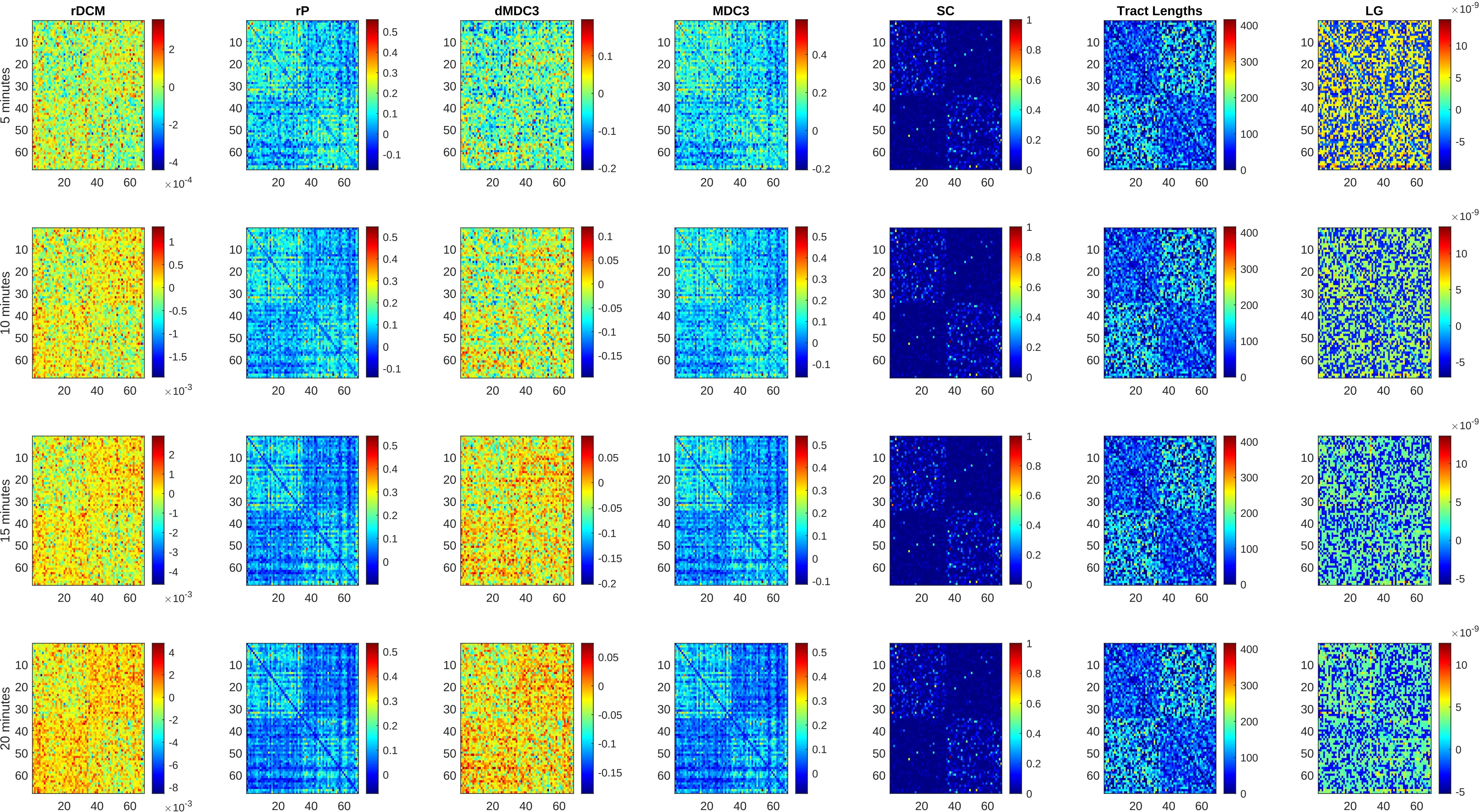
rDCM
rP
dMDC3
MDC3
SC
Tract Lengths
LG
5 minutes
10 minutes
15 minutes
20 minutes

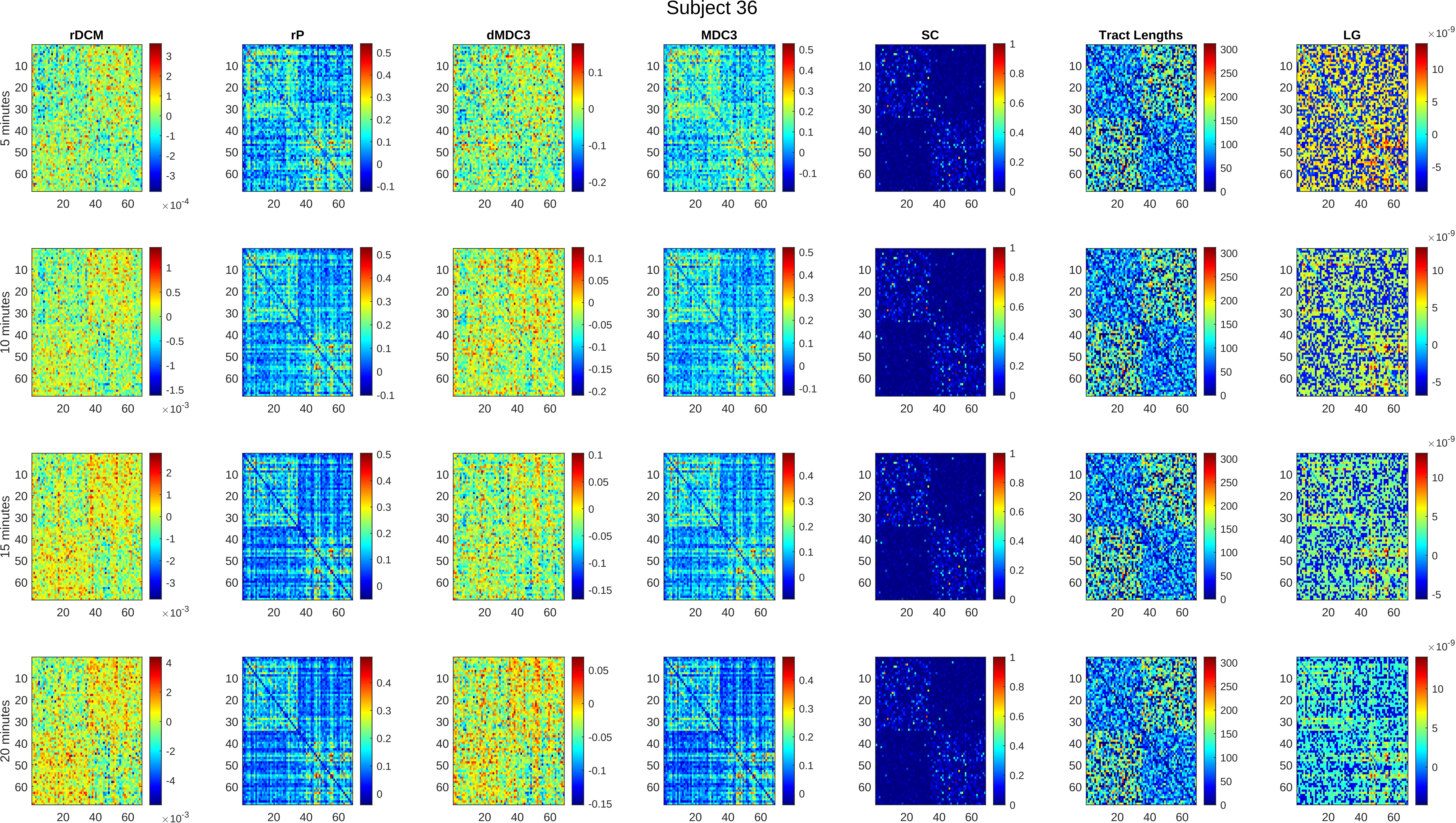
Subject 36
rDCM
rP
dMDC3
MDC3
SC
Tract Lengths
LG
5 minutes
10 minutes
15 minutes
20 minutes

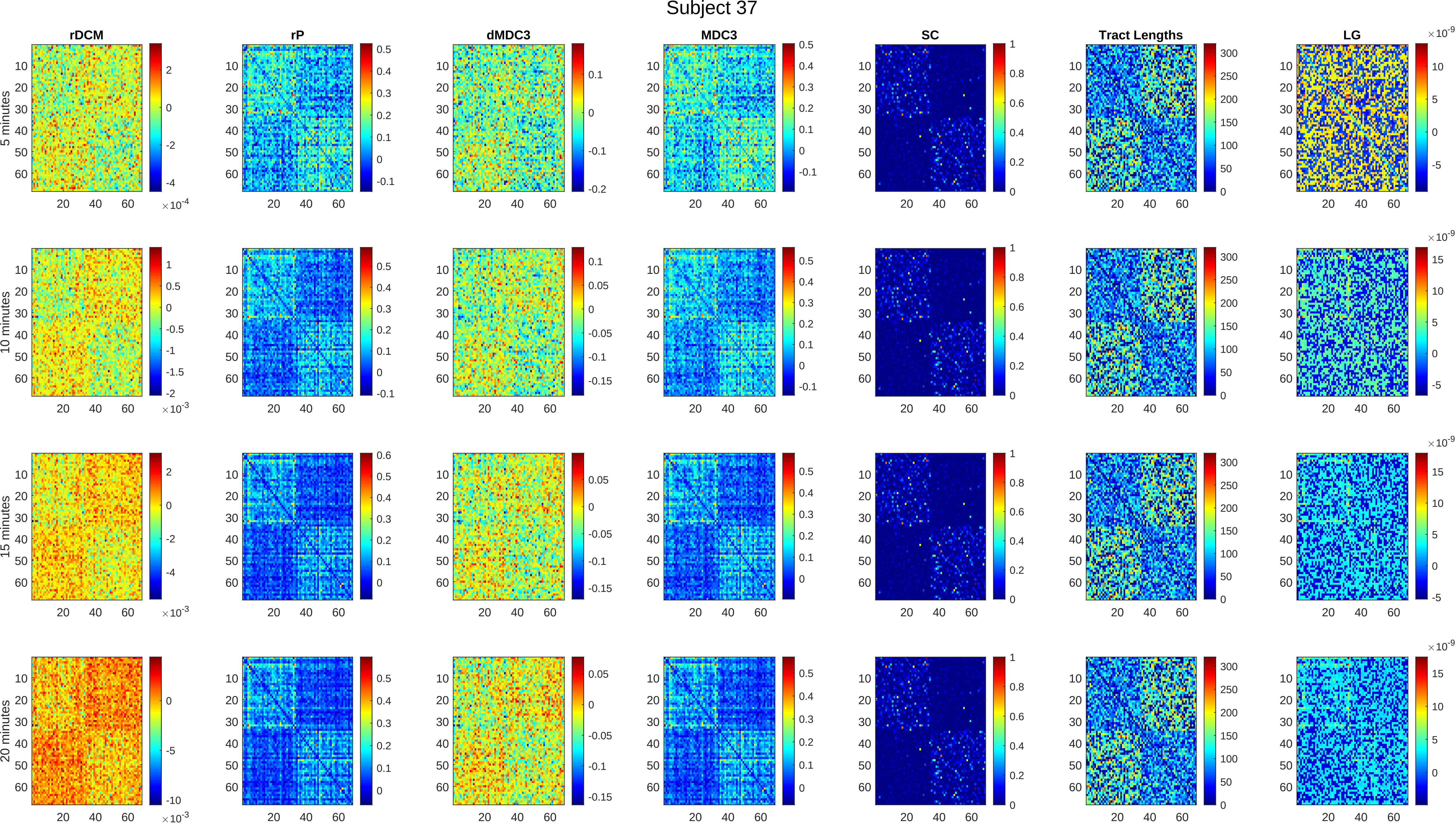

Subject 37
rDCM
rP
dMDC3
MDC3
SC
Tract Lengths
LG
5 minutes
10 minutes
15 minutes
20 minutes

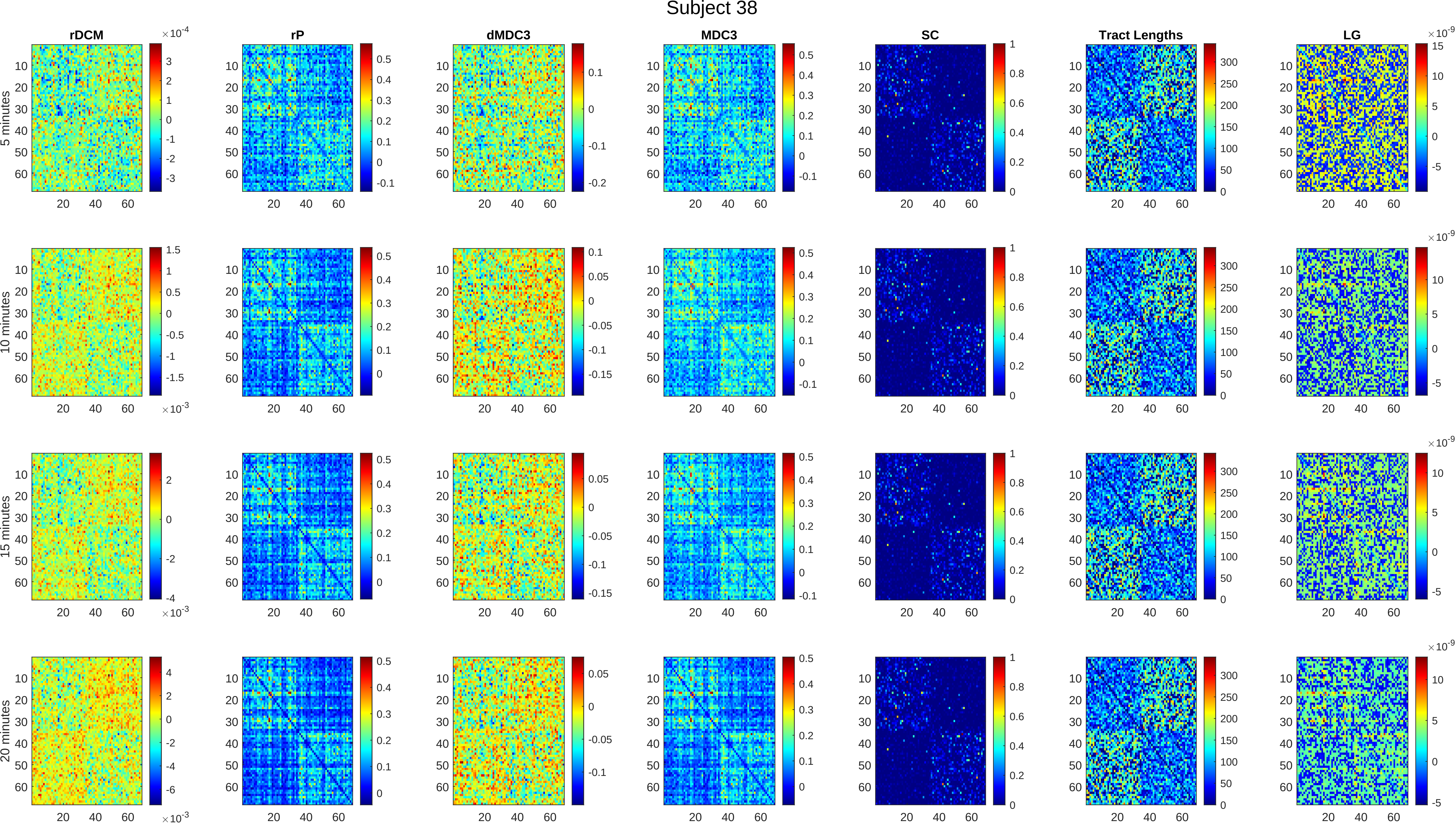

Subject 38
rDCM
rP
dMDC3
MDC3
SC
Tract Lengths
LG
5 minutes
10 minutes
15 minutes
20 minutes

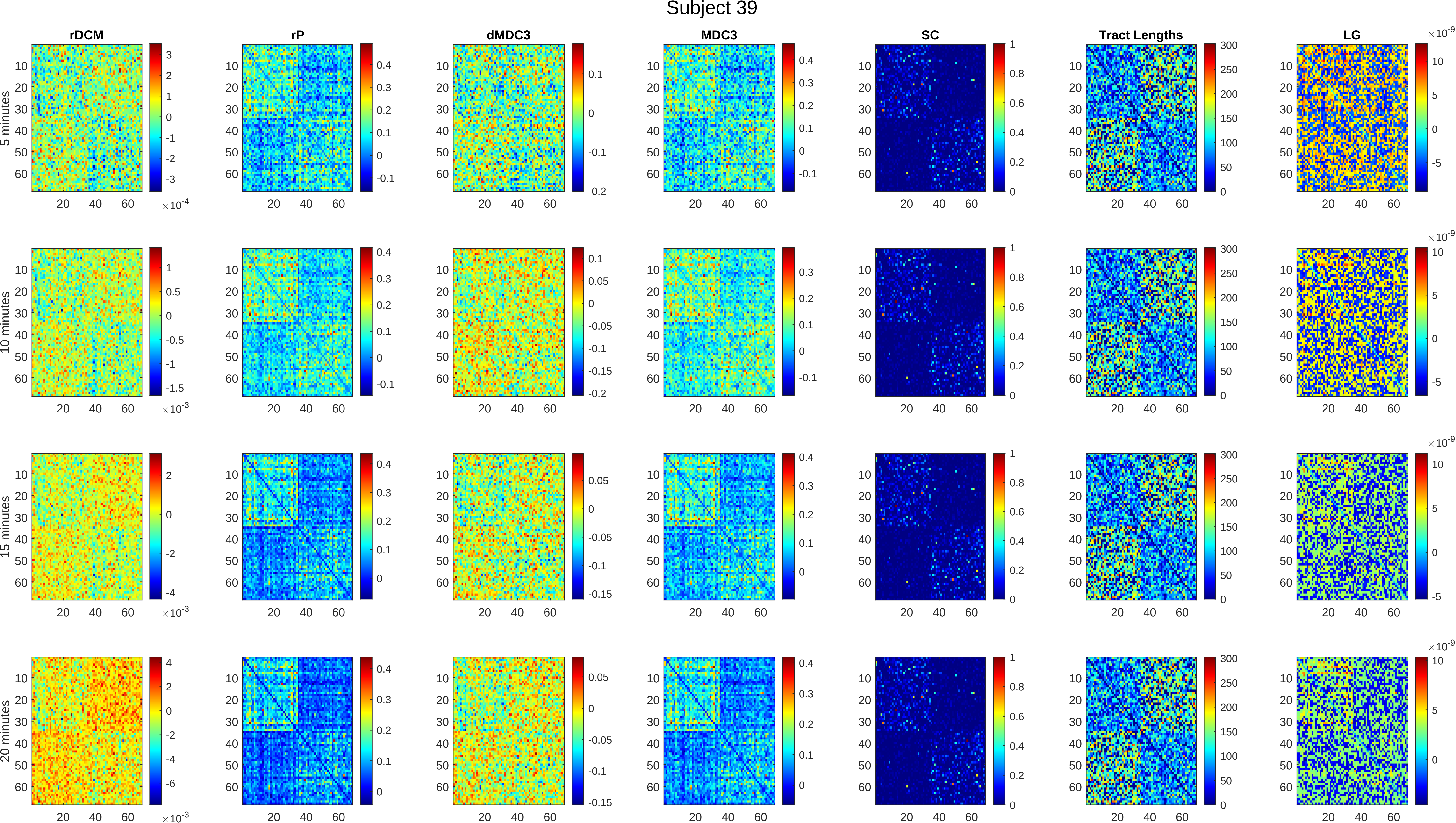

Subject 39
rDCM
rP
dMDC3
MDC3
SC
Tract Lengths
LG
5 minutes
10 minutes
15 minutes
20 minutes

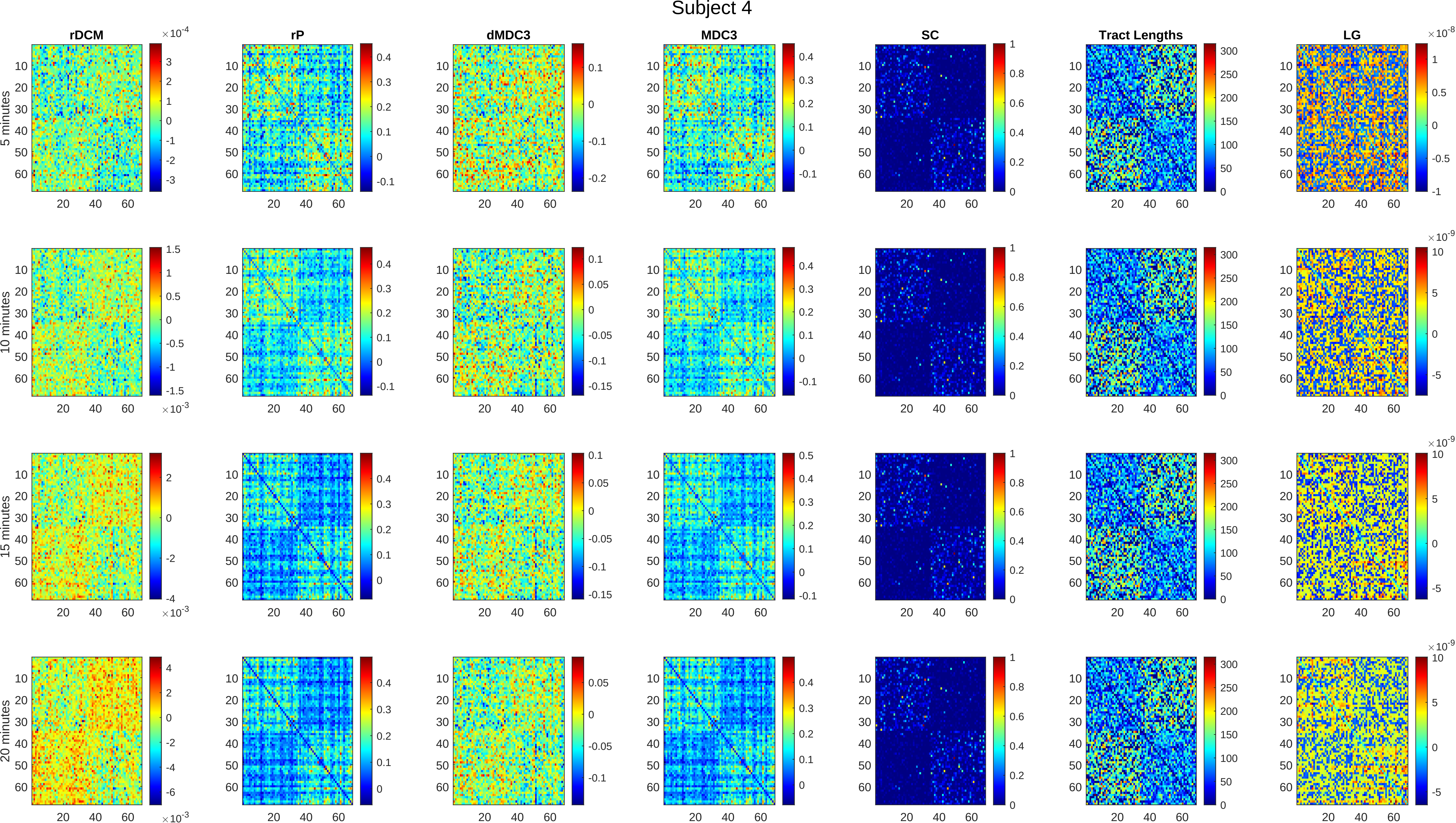
Subject 4
rDCM
rP
dMDC3
MDC3
SC
Tract Lengths
LG
5 minutes
10 minutes
15 minutes
20 minutes

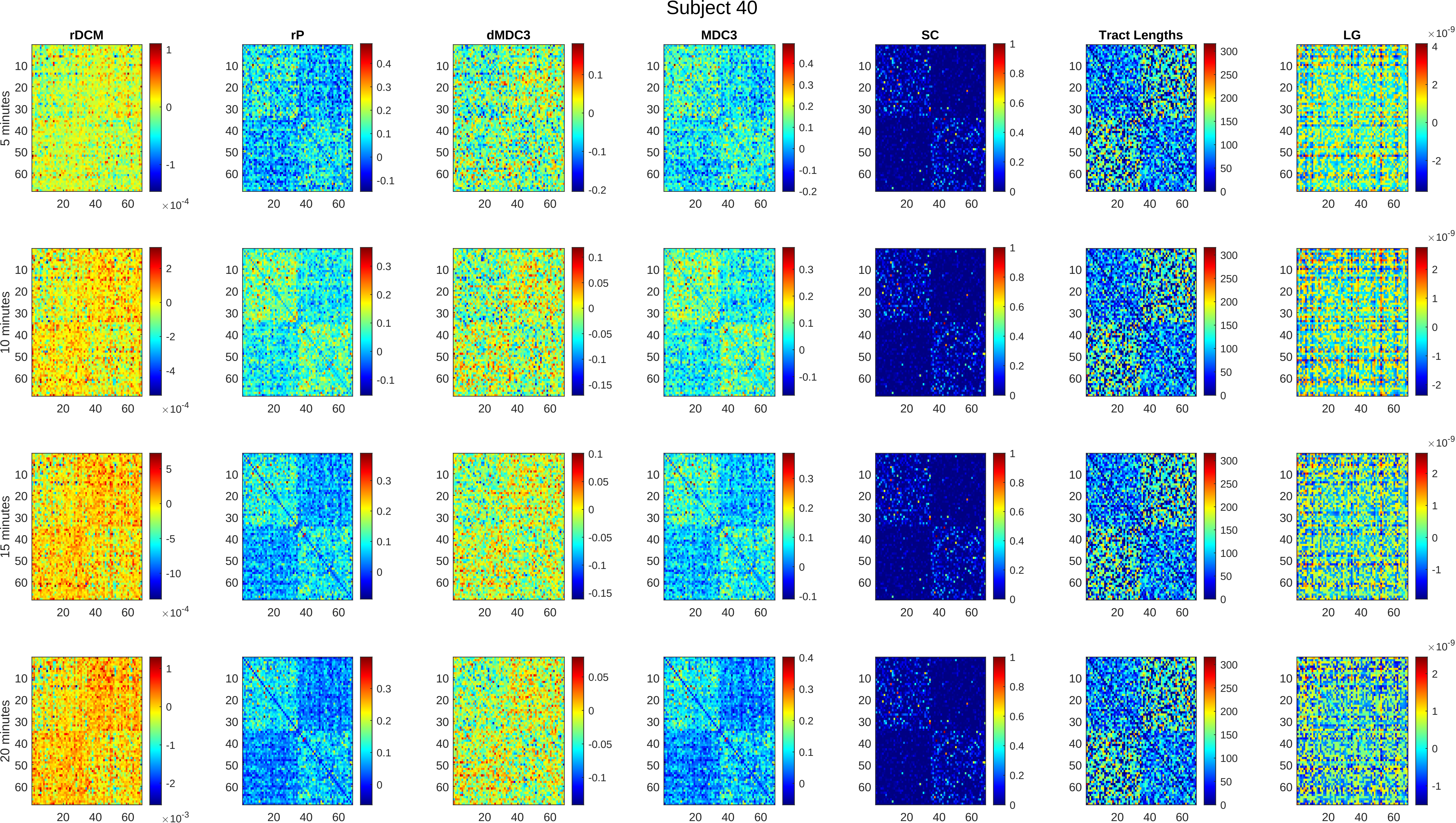
Subject 40
rDCM
rP
dMDC3
MDC3
SC
Tract Lengths
LG
5 minutes
10 minutes
15 minutes
20 minutes

Subject 41

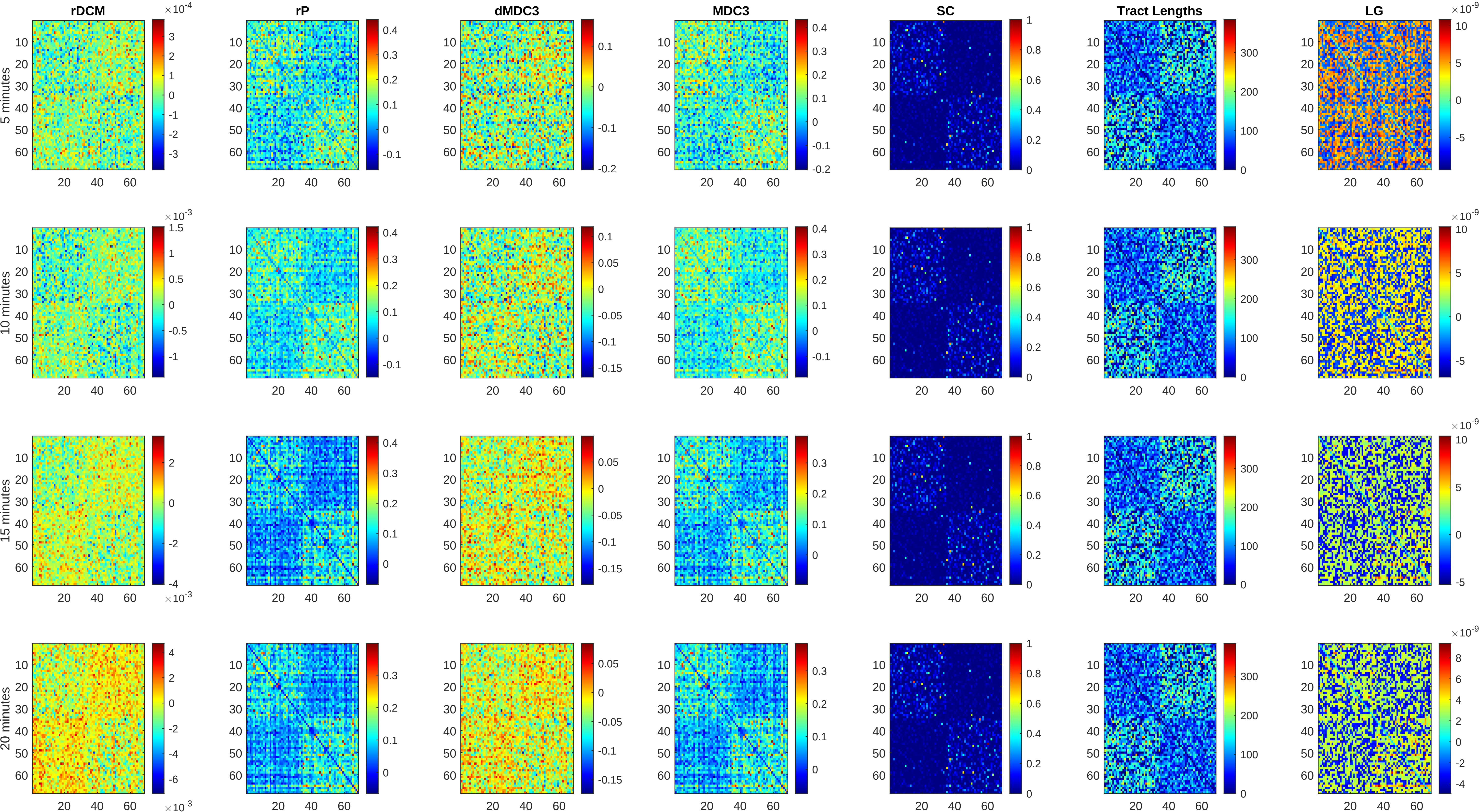
rDCM
rP
dMDC3
MDC3
SC
Tract Lengths
LG
5 minutes
10 minutes
15 minutes
20 minutes

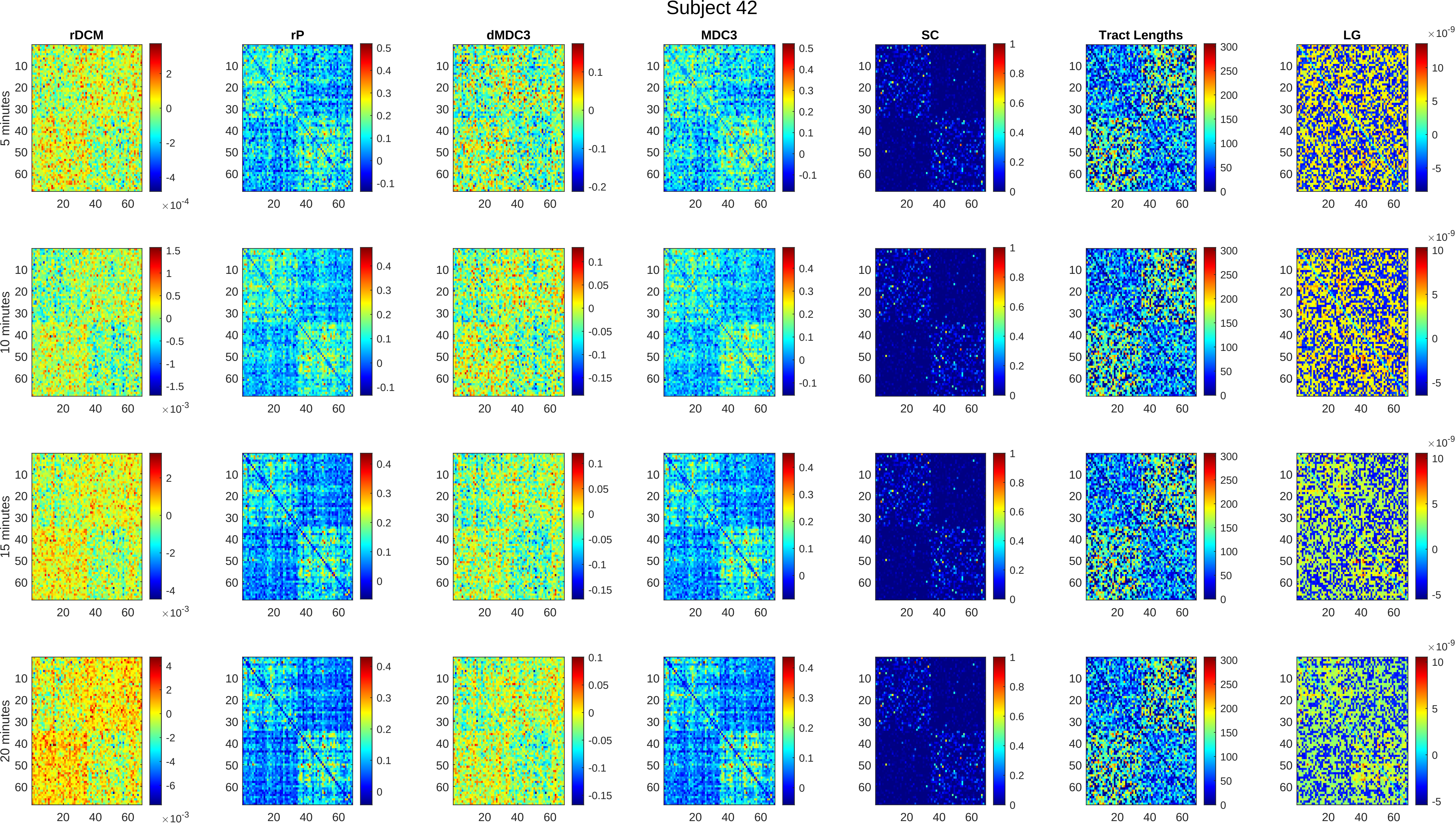

Subject 42
rDCM
rP
dMDC3
MDC3
SC
Tract Lengths
LG
5 minutes
10 minutes
15 minutes
20 minutes

Subject 43

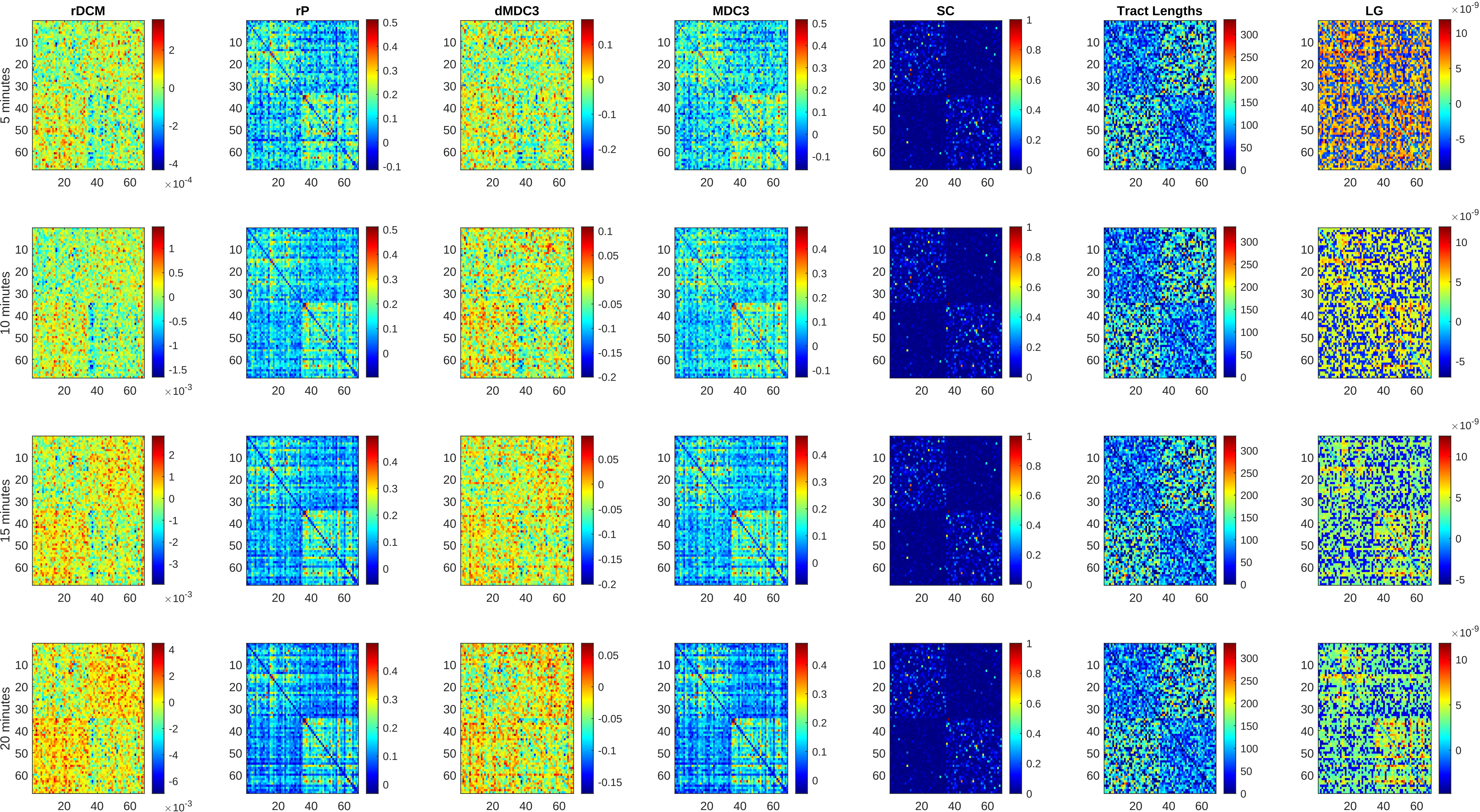

rDCM
rP
dMDC3
MDC3
SC
Tract Lengths
LG
5 minutes
10 minutes
15 minutes
20 minutes

Subject 44

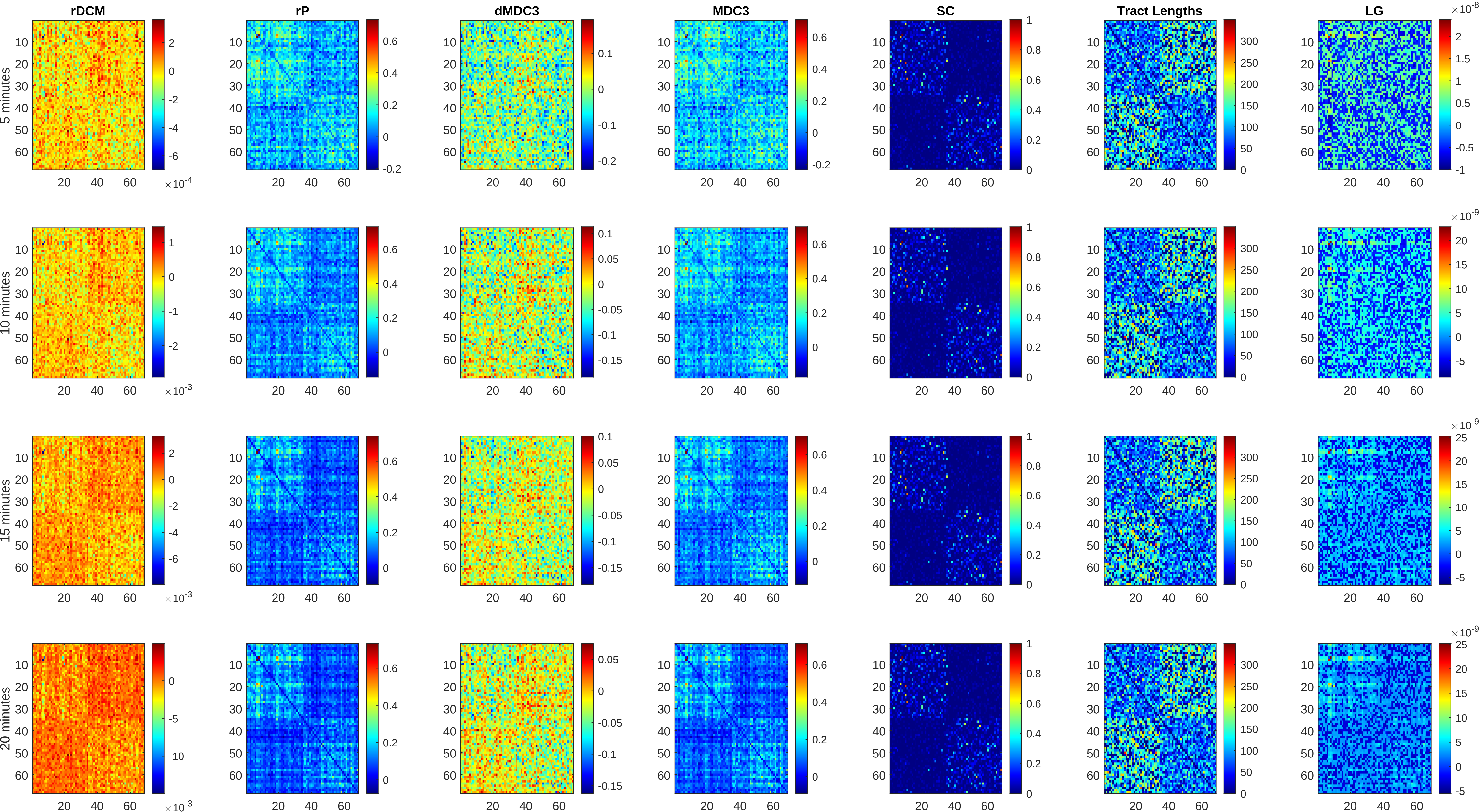
rDCM
rP
dMDC3
MDC3
SC
Tract Lengths
LG
5 minutes
10 minutes
15 minutes
20 minutes

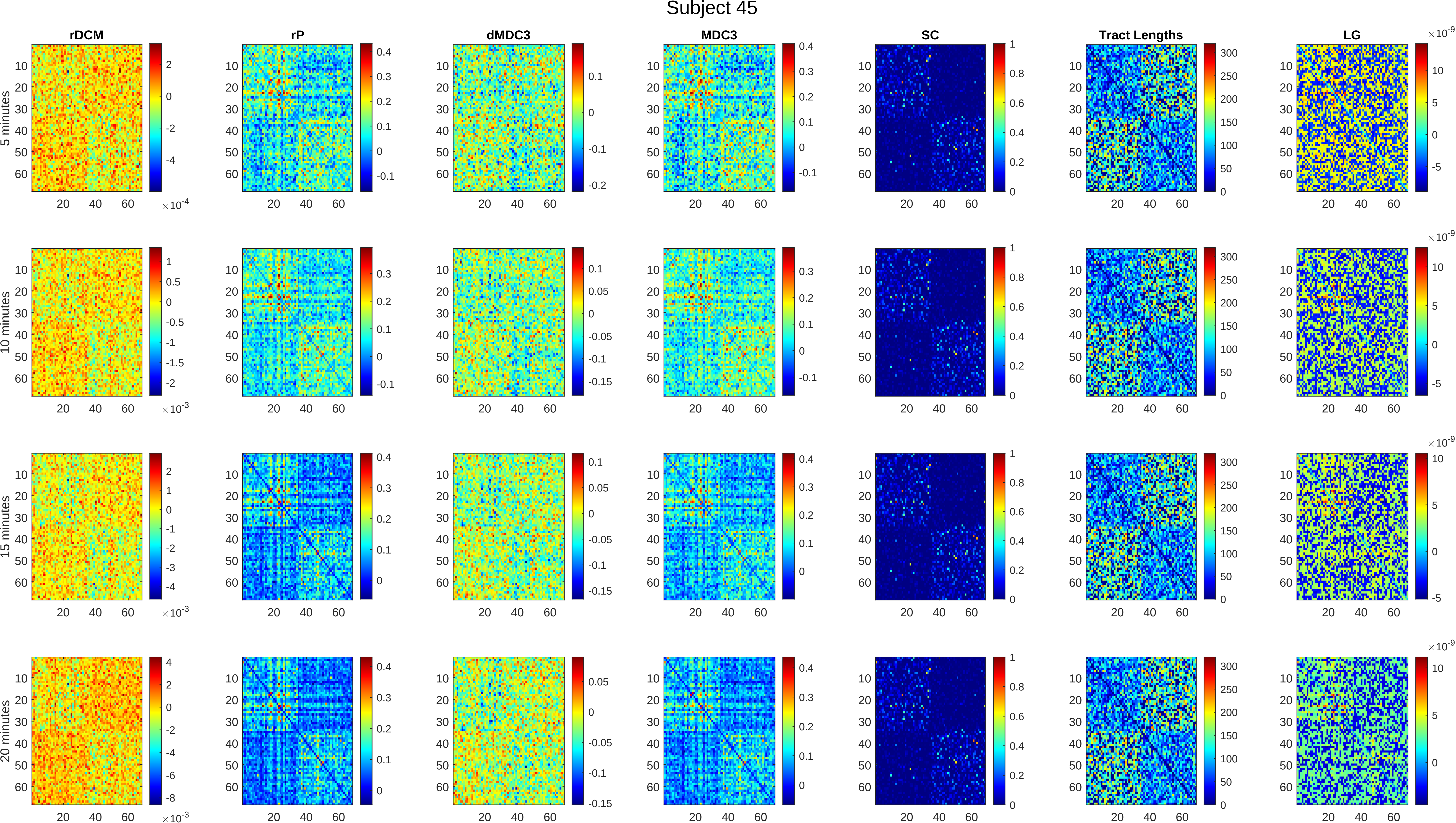
Subject 45
rDCM
rP
dMDC3
MDC3
SC
Tract Lengths
LG
5 minutes
10 minutes
15 minutes
20 minutes

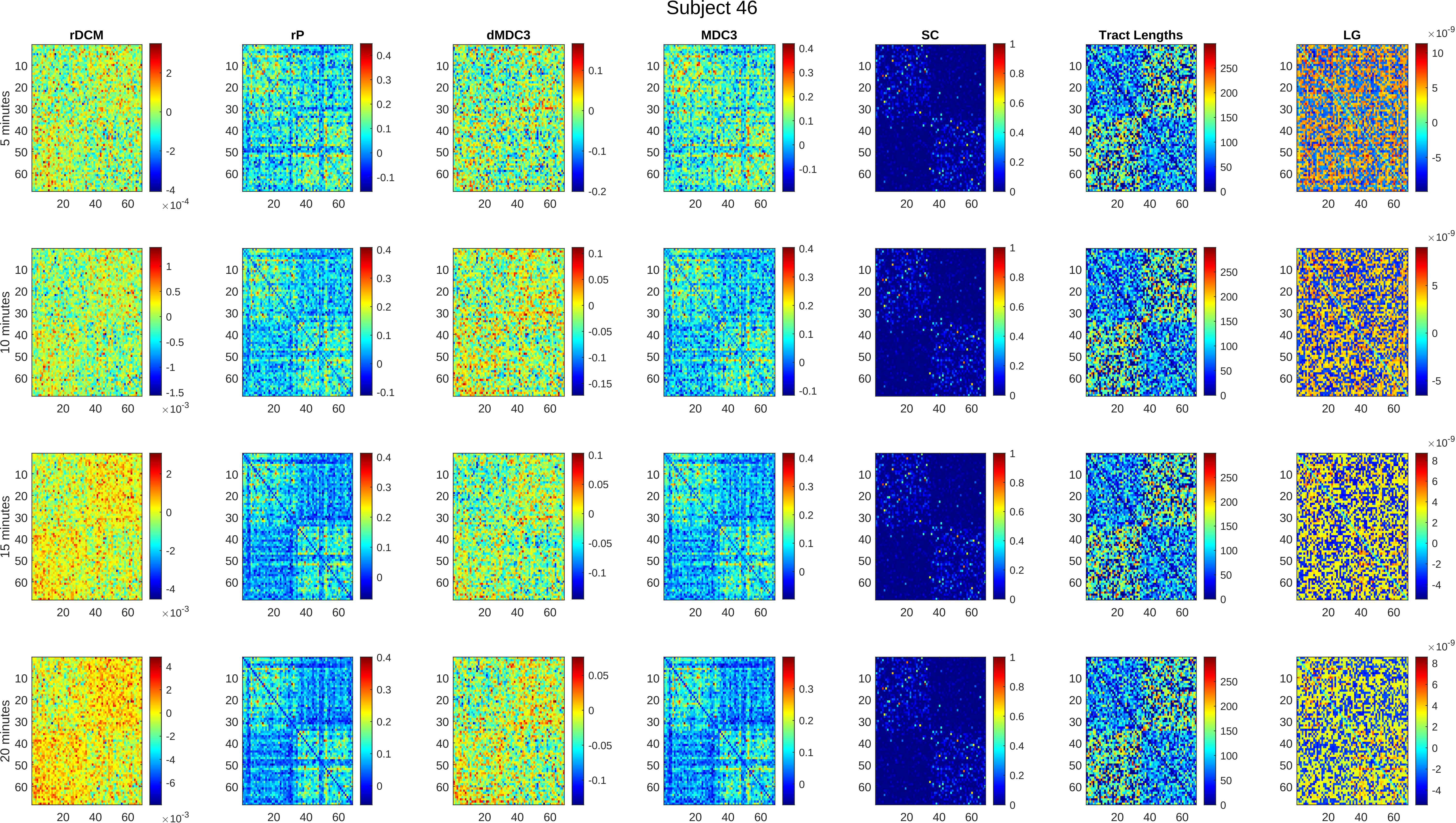

Subject 46
rDCM
rP
dMDC3
MDC3
SC
Tract Lengths
LG
5 minutes
10 minutes
15 minutes
20 minutes

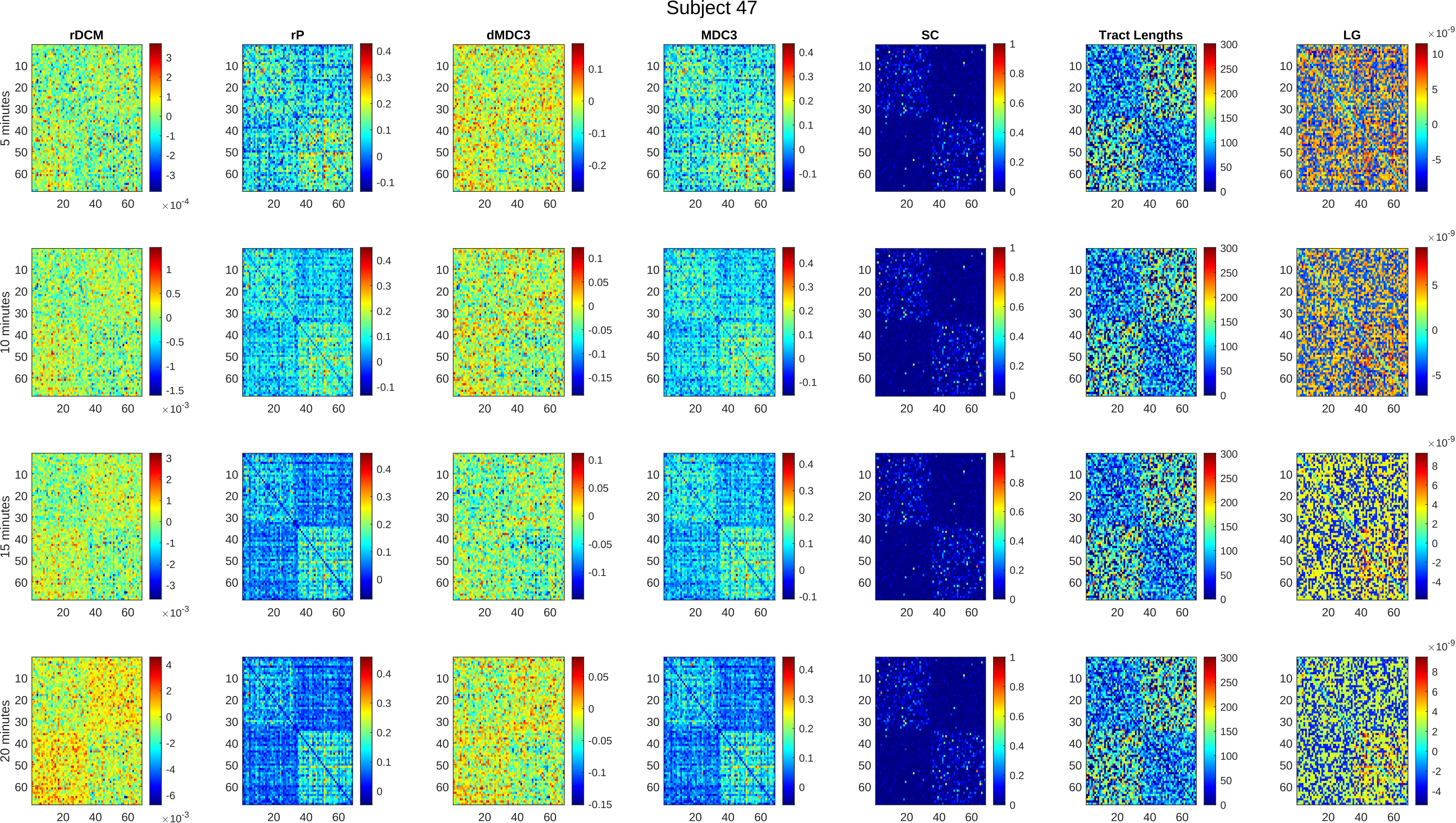
Subject 47
rDCM
rP
dMDC3
MDC3
SC
Tract Lengths
LG
5 minutes
10 minutes
15 minutes
20 minutes

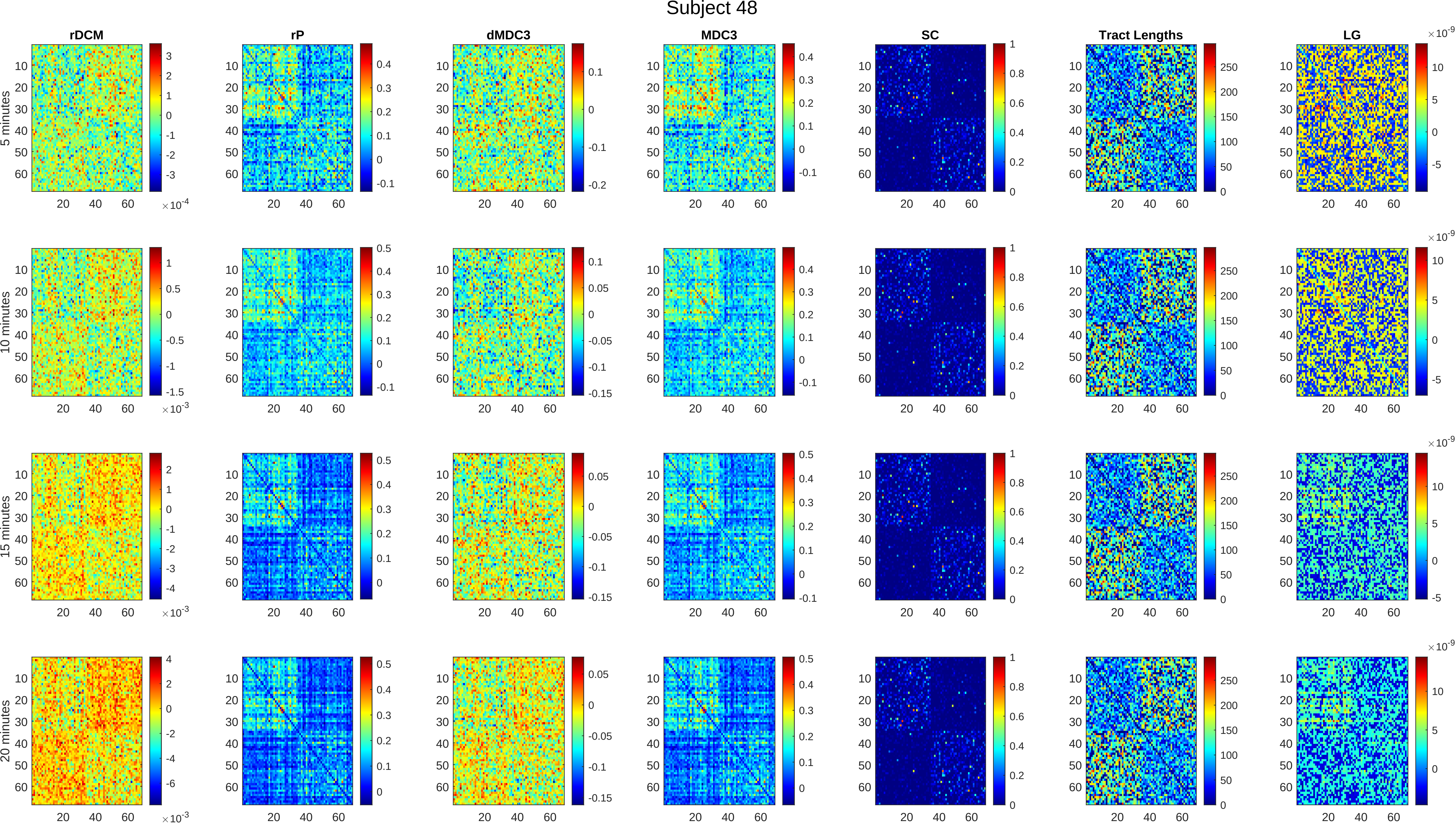

Subject 48
rDCM
rP
dMDC3
MDC3
SC
Tract Lengths
LG
5 minutes
10 minutes
15 minutes
20 minutes

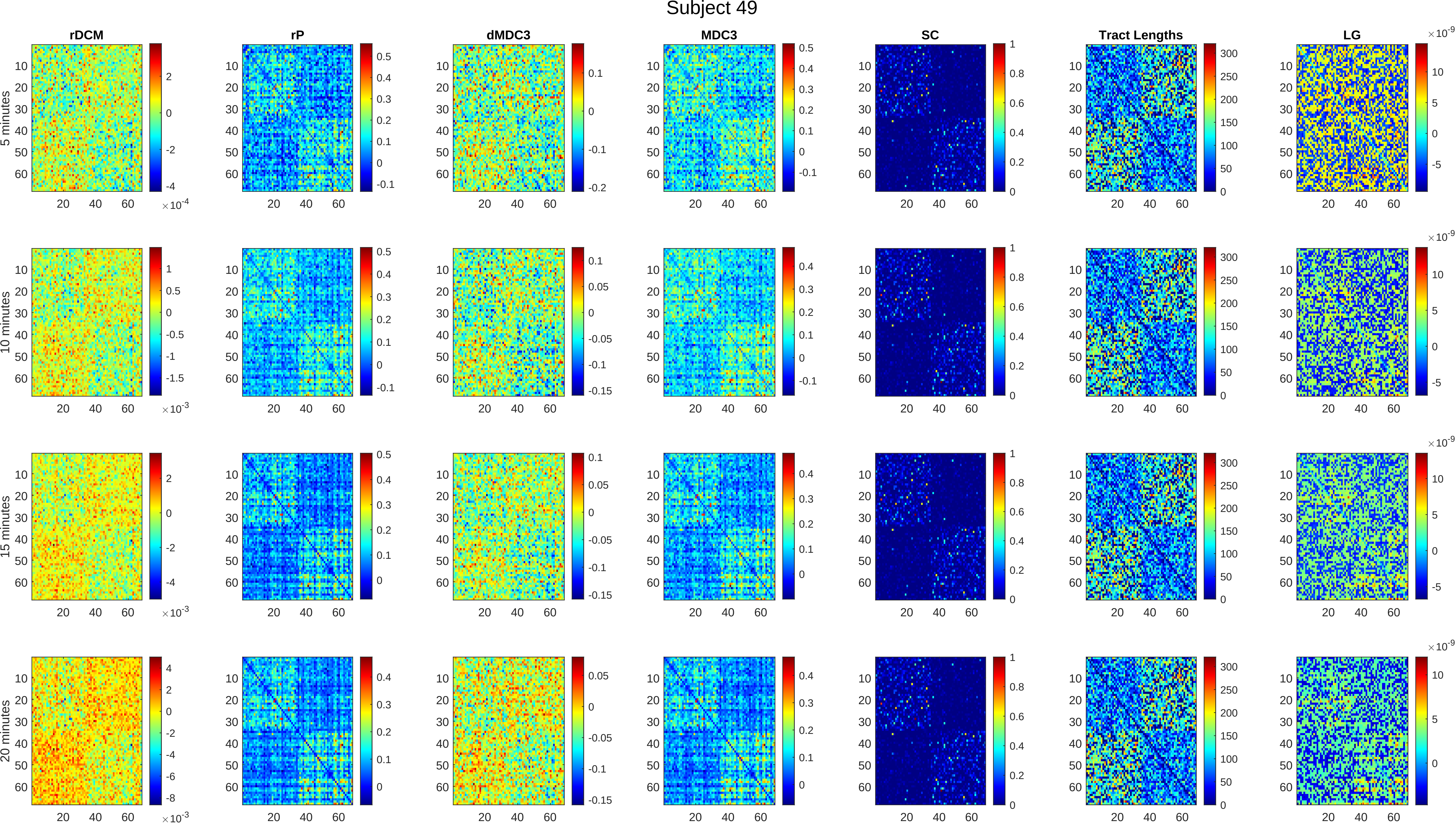
Subject 49
rDCM
rP
dMDC3
MDC3
SC
Tract Lengths
LG
5 minutes
10 minutes
15 minutes
20 minutes

Subject 5

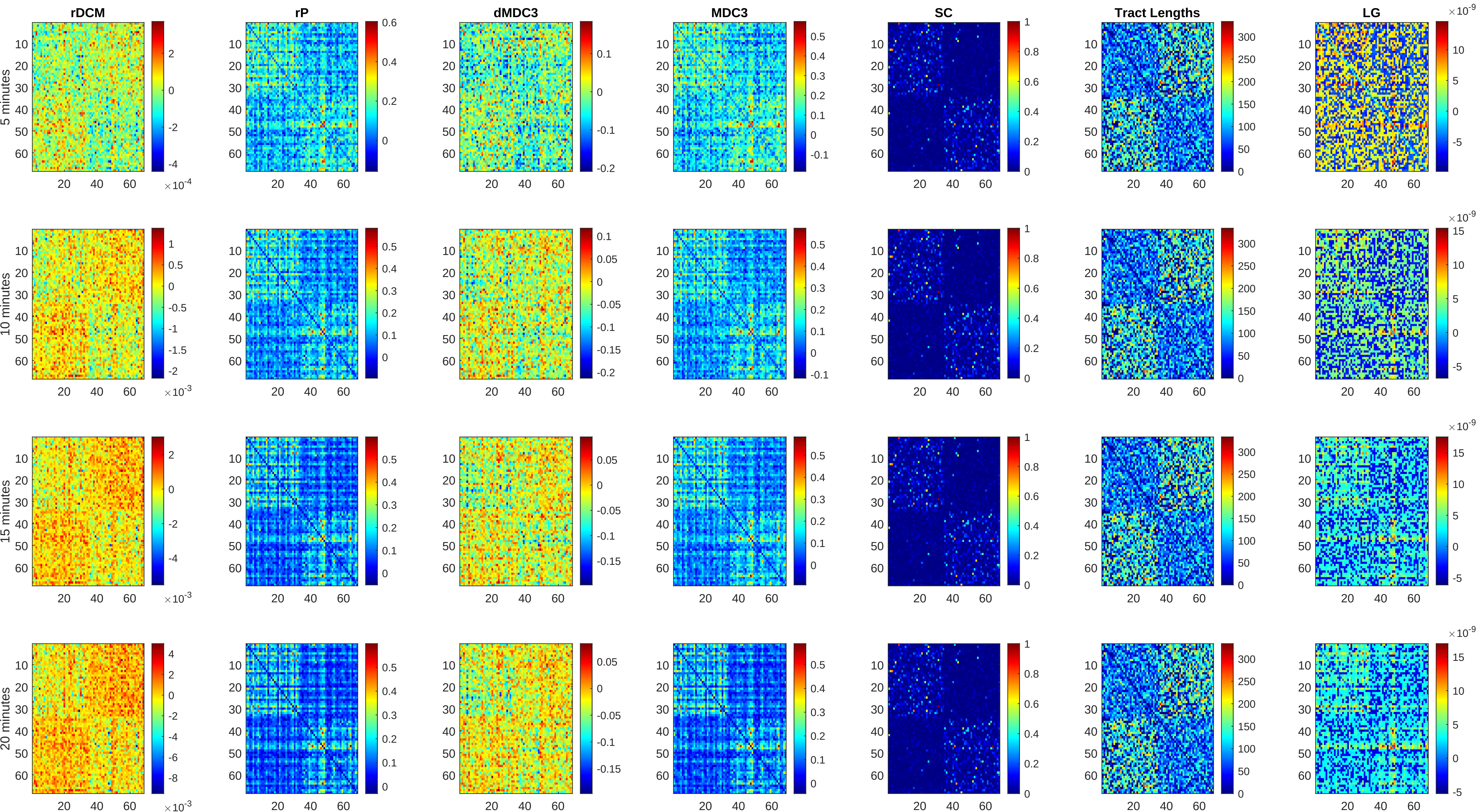
rDCM
rP
dMDC3
MDC3
SC
Tract Lengths
LG
5 minutes
10 minutes
15 minutes
20 minutes

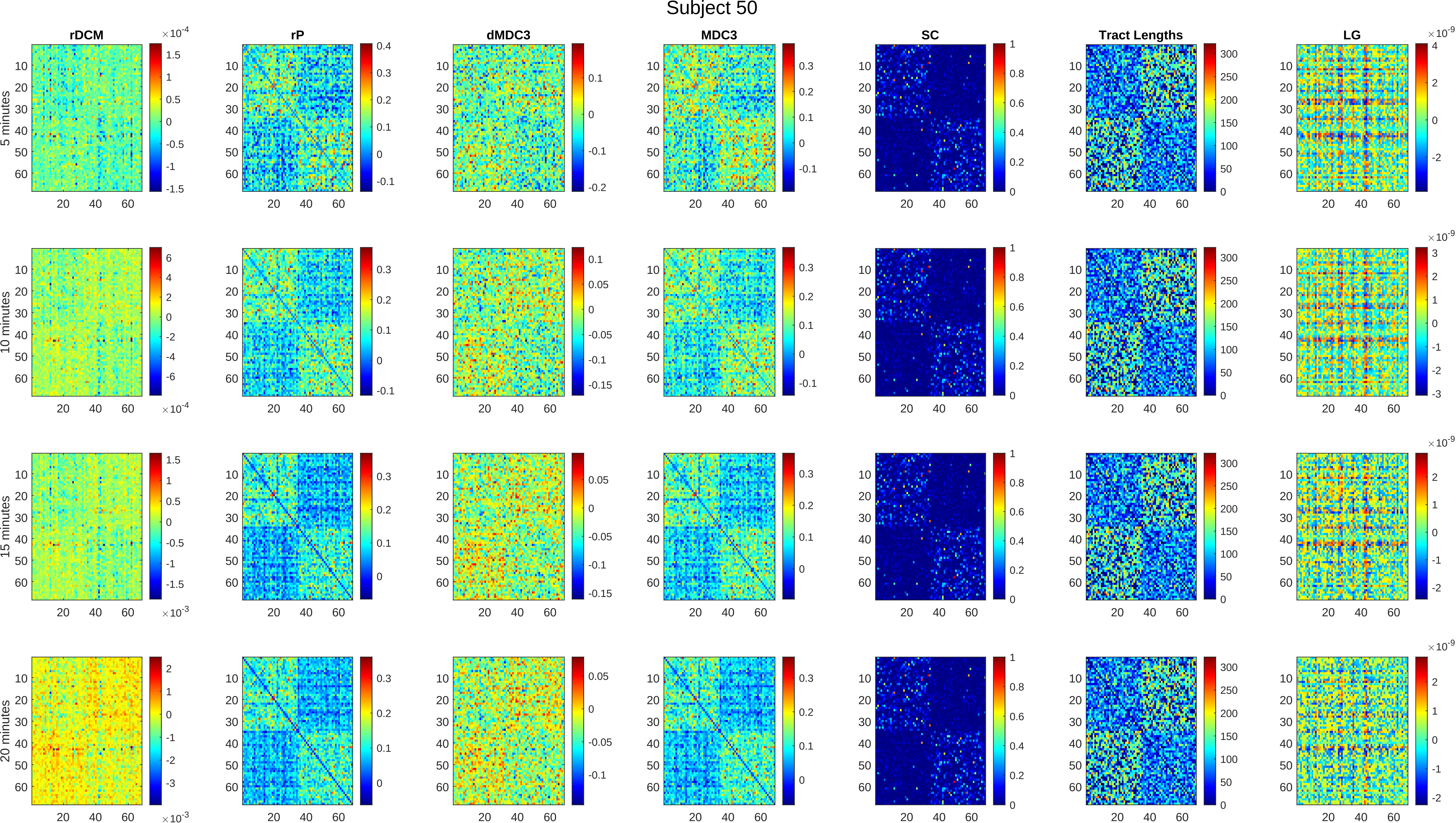

Subject 50
rDCM
rP
dMDC3
MDC3
SC
Tract Lengths
LG
5 minutes
10 minutes
15 minutes
20 minutes

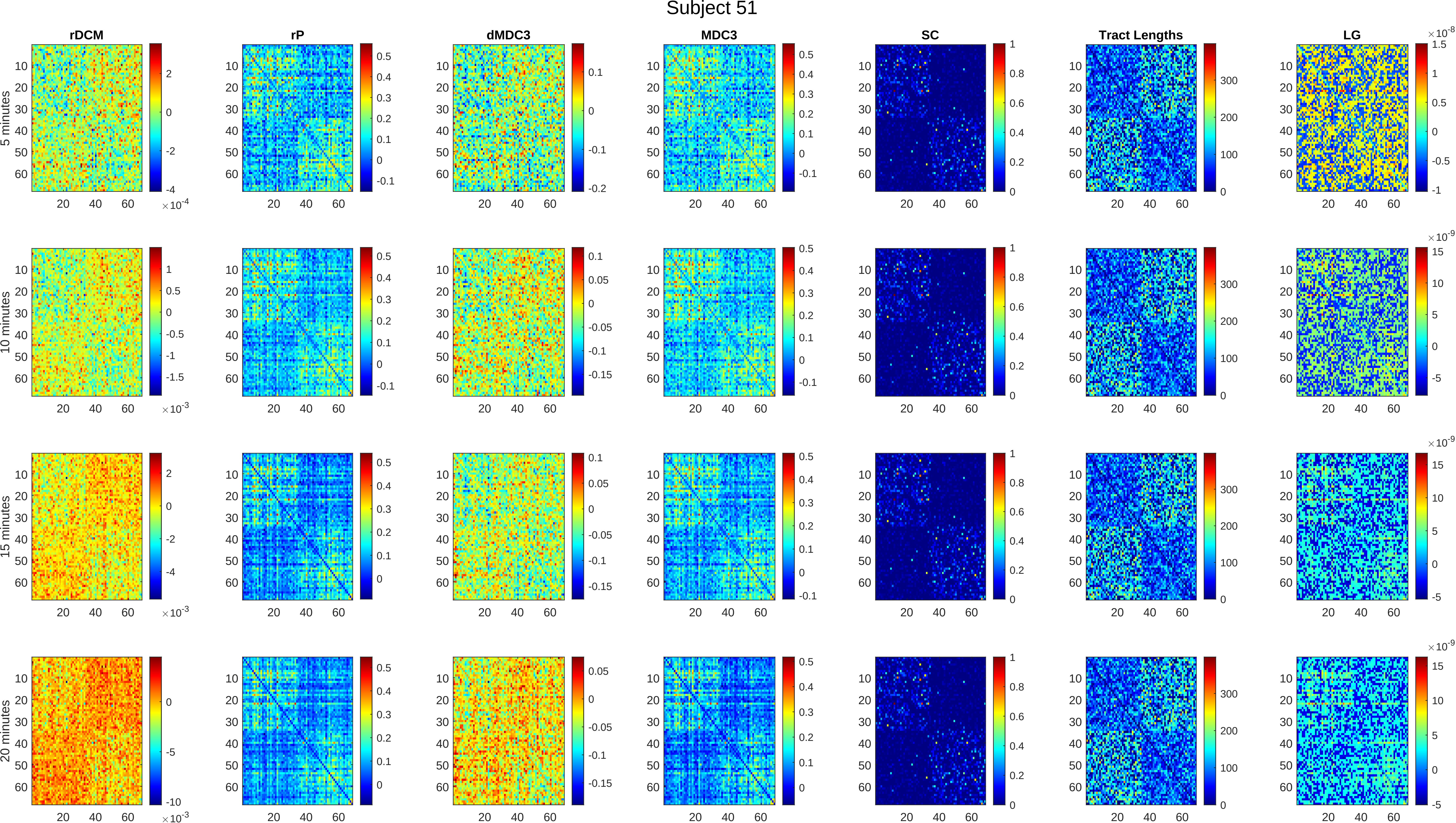

Subject 51
rDCM
rP
dMDC3
MDC3
SC
Tract Lengths
LG
5 minutes
10 minutes
15 minutes
20 minutes

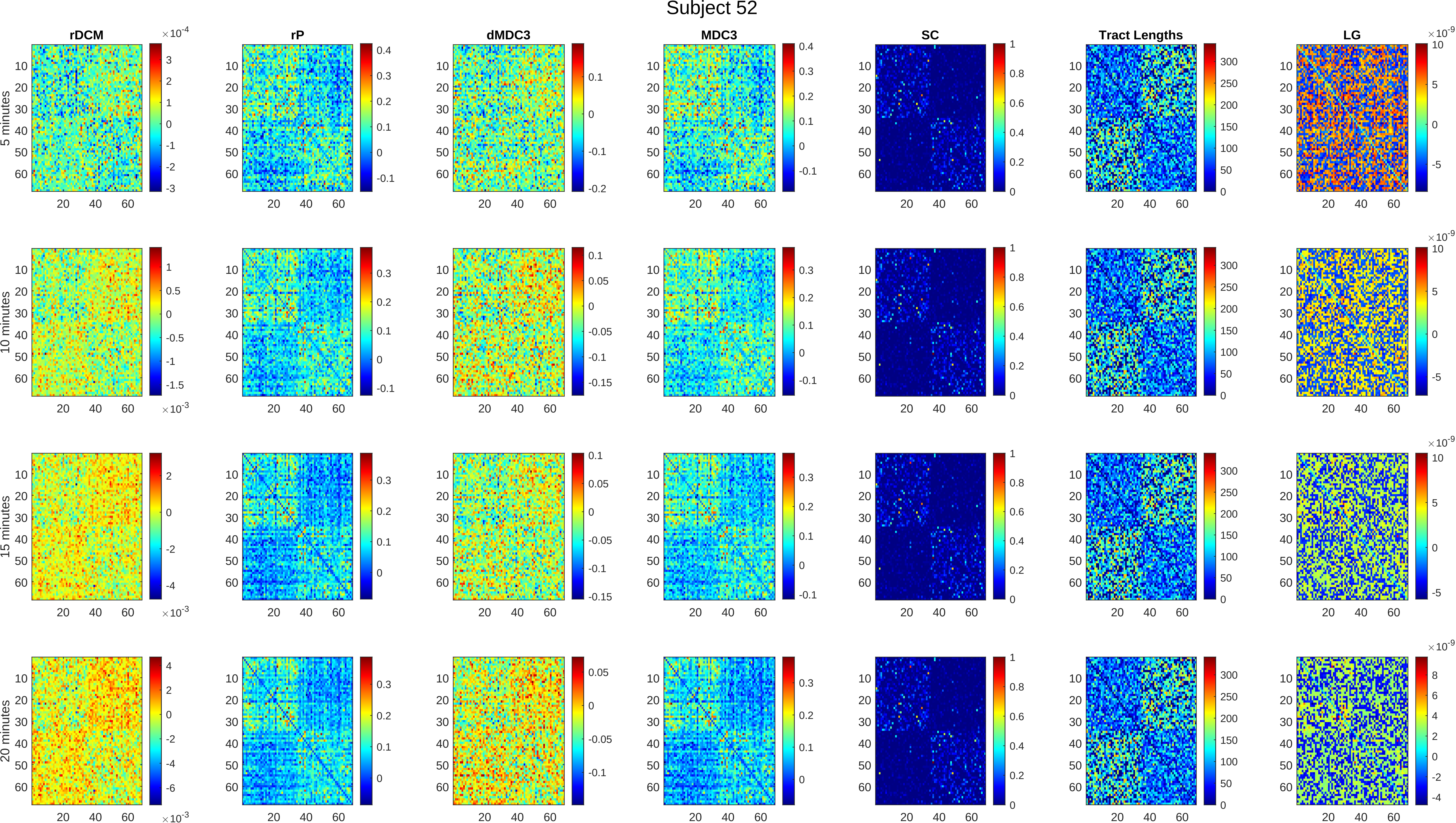
Subject 52
rDCM
rP
dMDC3
MDC3
SC
Tract Lengths
LG
5 minutes
10 minutes
15 minutes
20 minutes

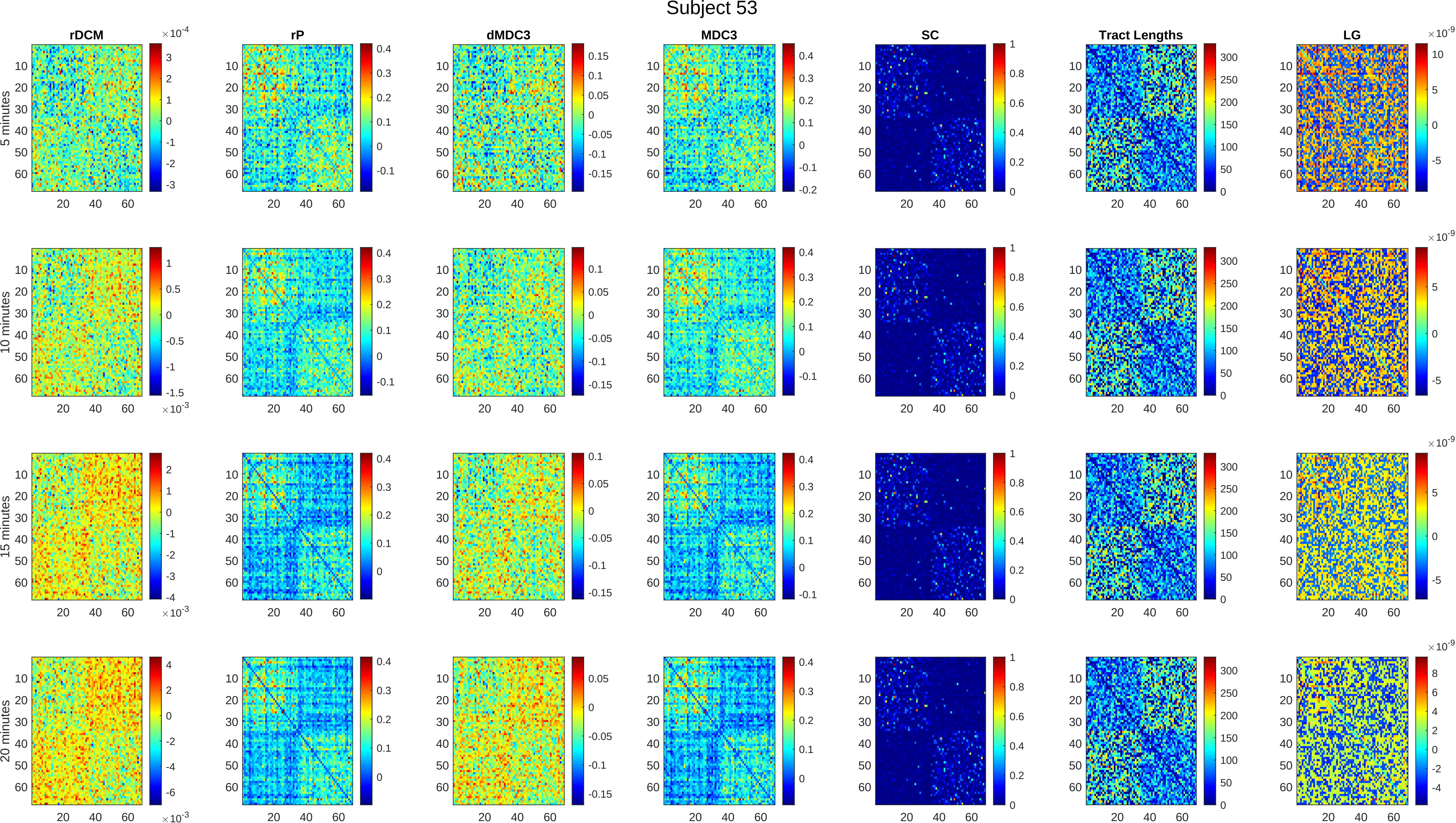

Subject 53
rDCM
rP
dMDC3
MDC3
SC
Tract Lengths
LG
5 minutes
10 minutes
15 minutes
20 minutes

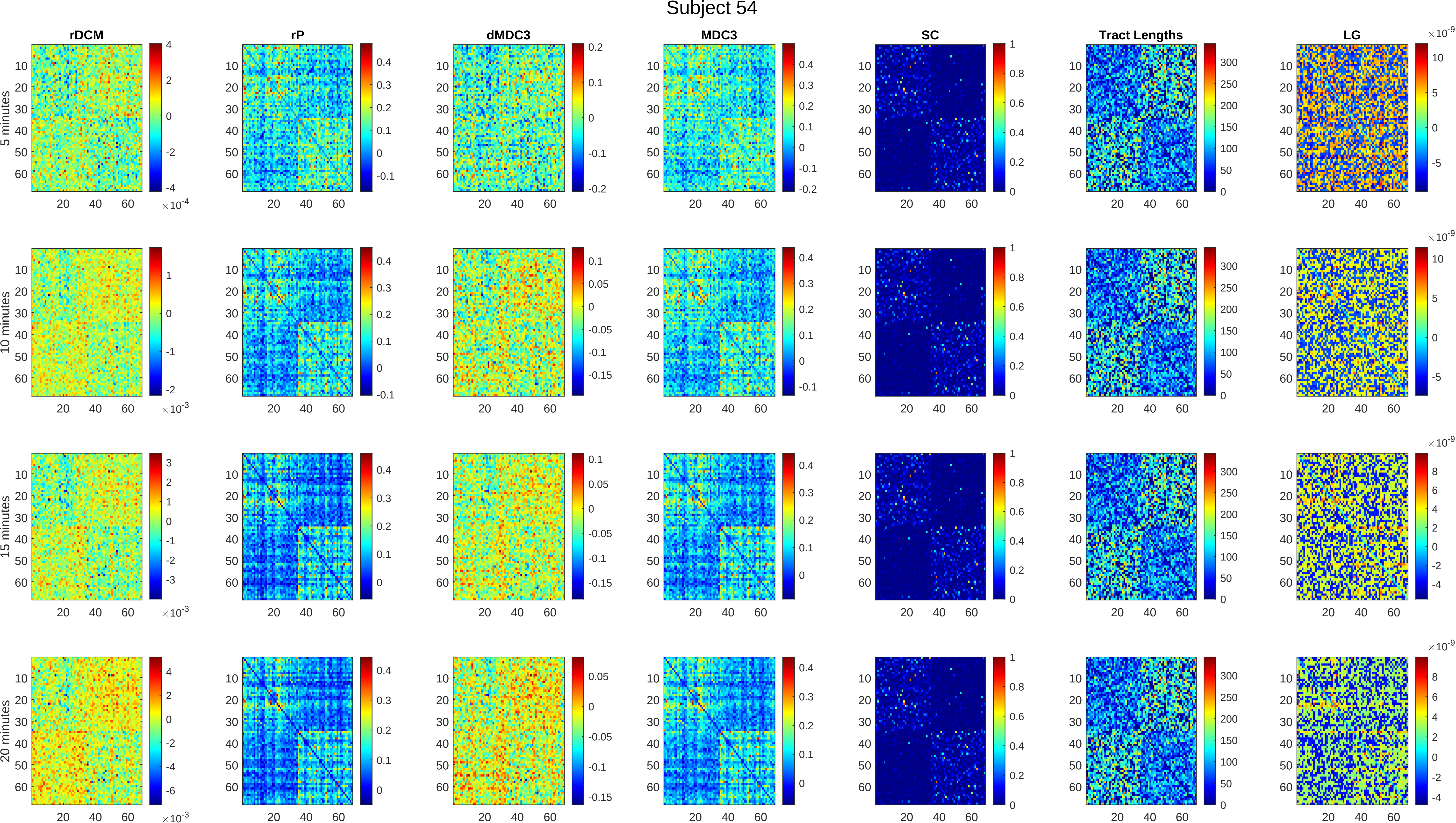

Subject 54
rDCM
rP
dMDC3
MDC3
SC
Tract Lengths
LG
5 minutes
10 minutes
15 minutes
20 minutes

Subject 55

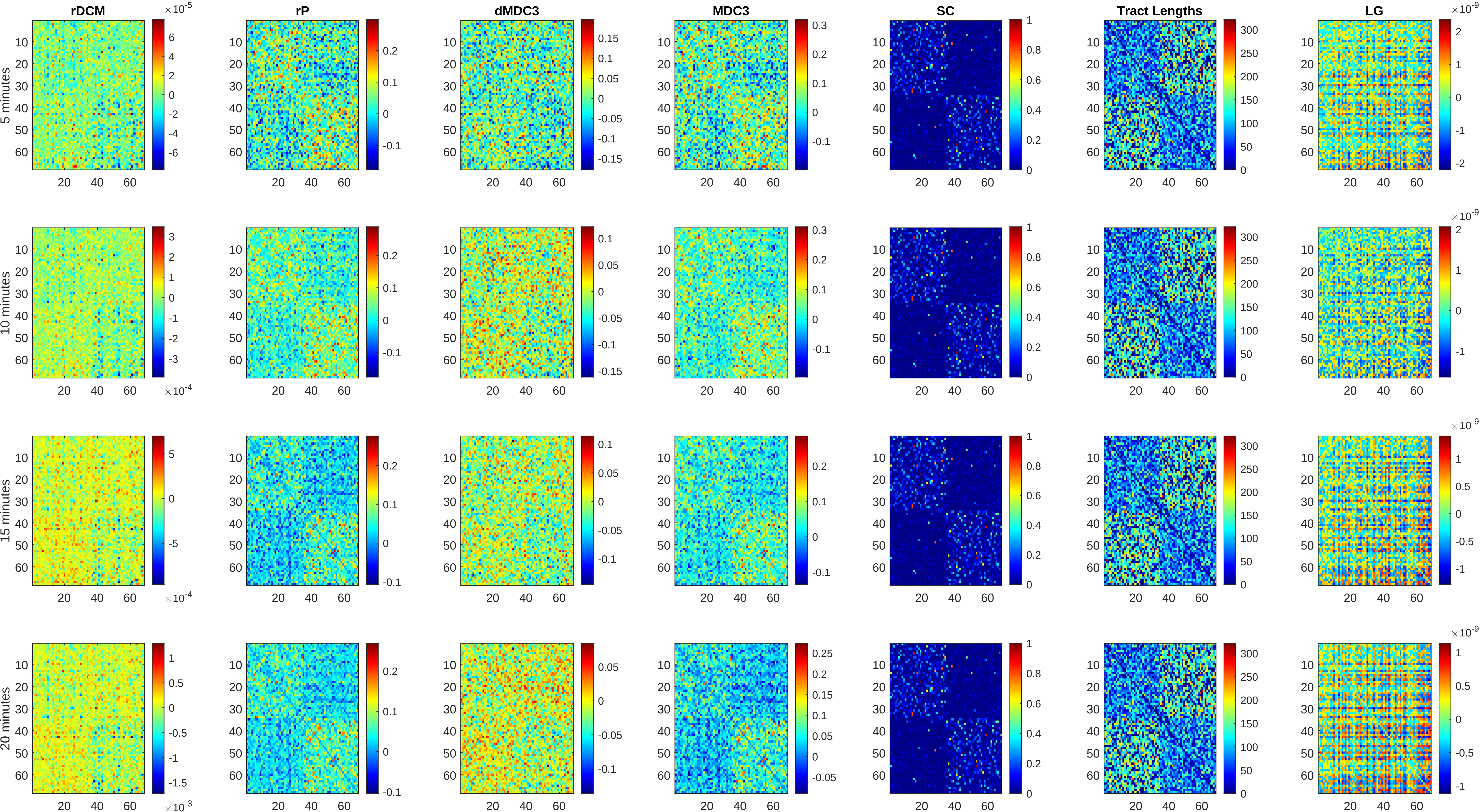

rDCM
rP
dMDC3
MDC3
SC
Tract Lengths
LG
5 minutes
10 minutes
15 minutes
20 minutes

Subject 56

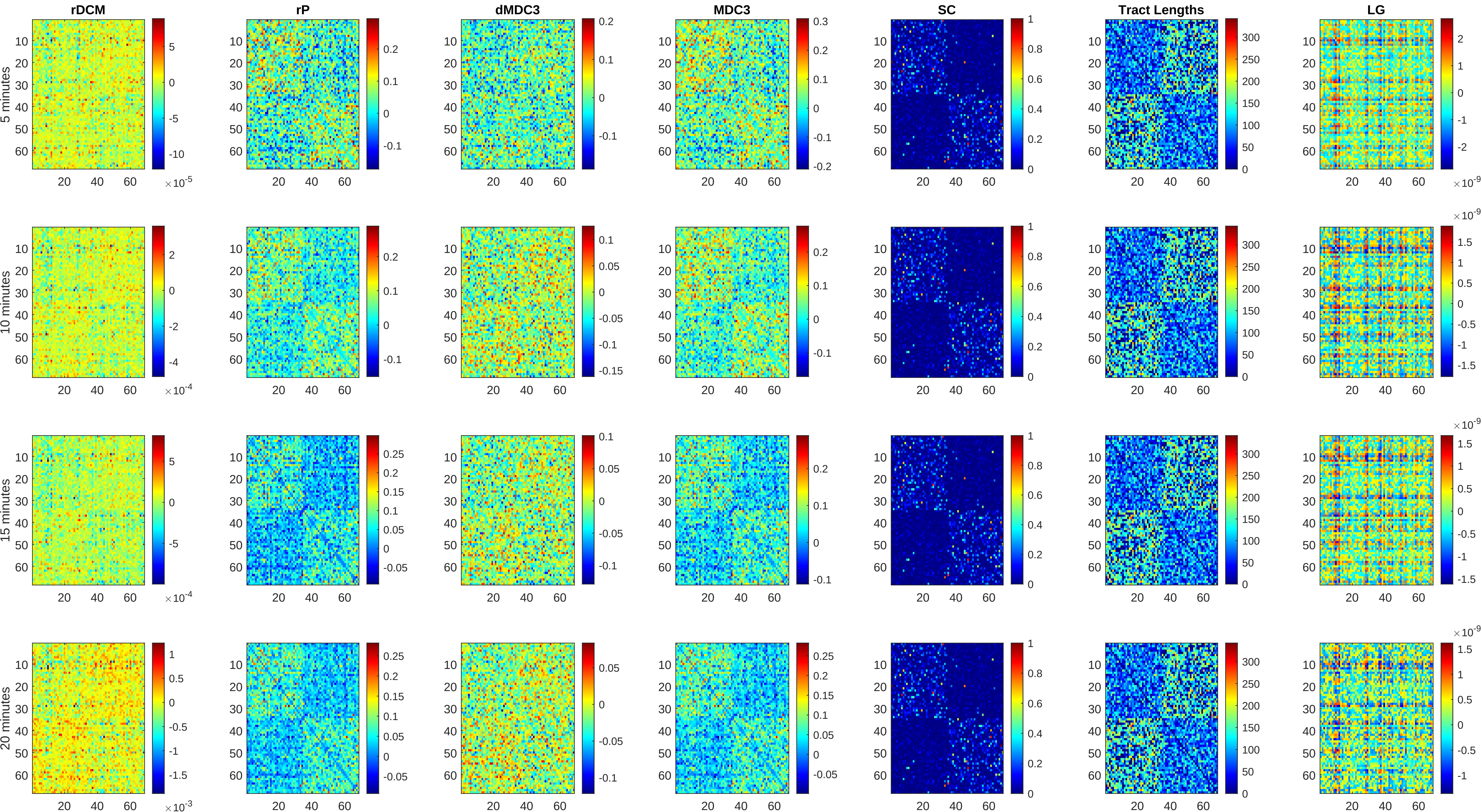
rDCM
rP
dMDC3
MDC3
SC
Tract Lengths
LG
5 minutes
10 minutes
15 minutes
20 minutes

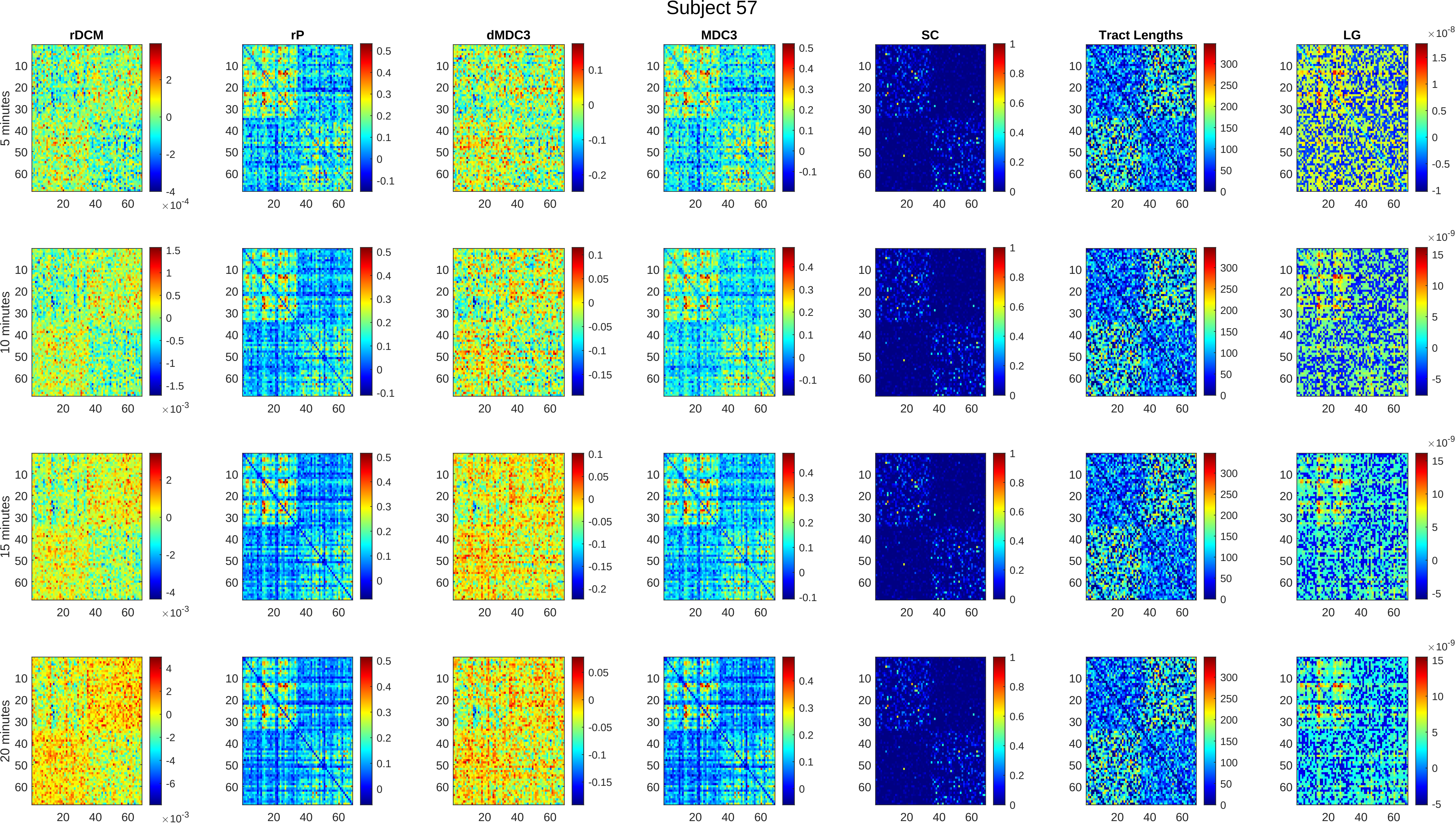
Subject 57
rDCM
rP
dMDC3
MDC3
SC
Tract Lengths
LG
5 minutes
10 minutes
15 minutes
20 minutes

Subject 58

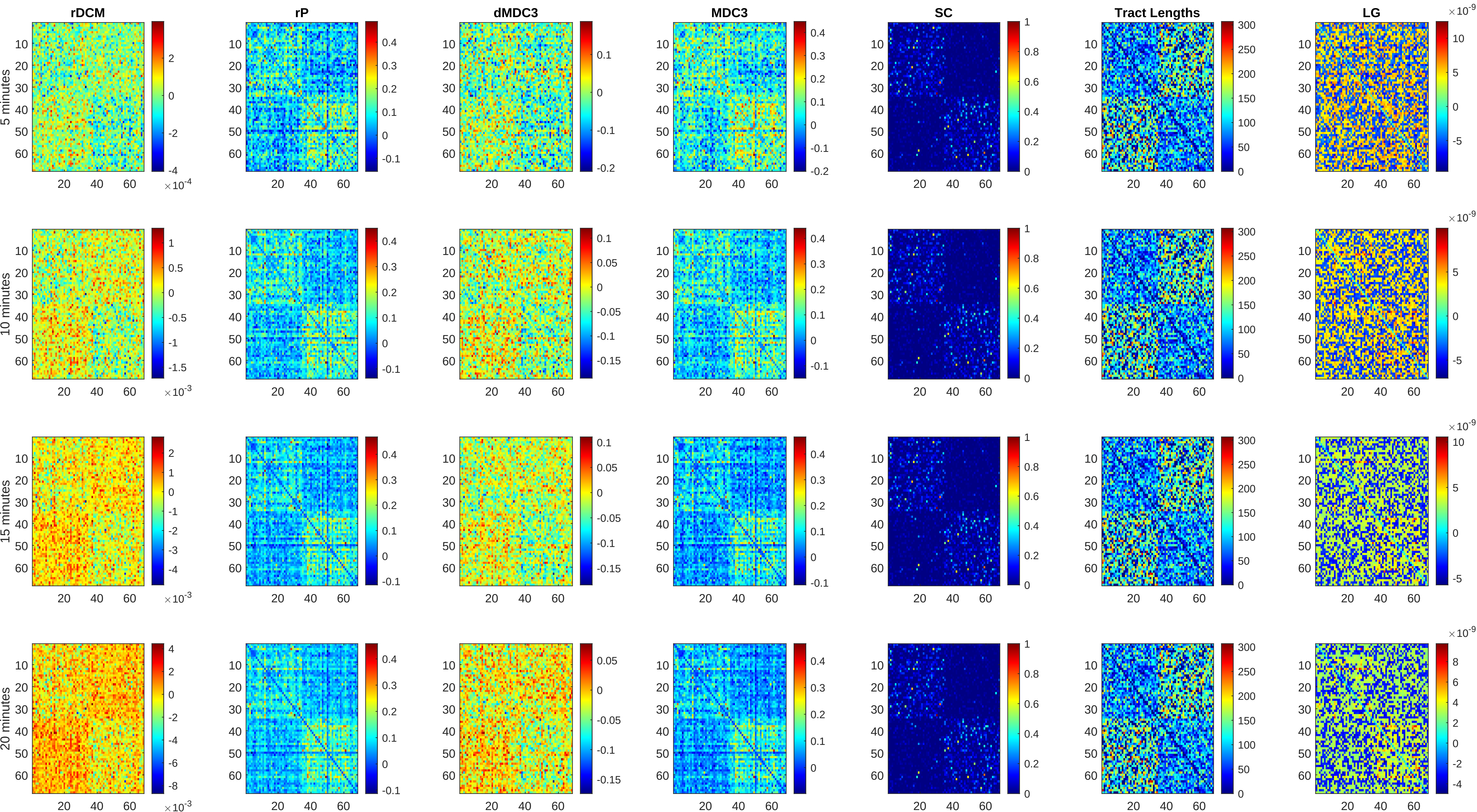

rDCM
rP
dMDC3
MDC3
SC
Tract Lengths
LG
5 minutes
10 minutes
15 minutes
20 minutes

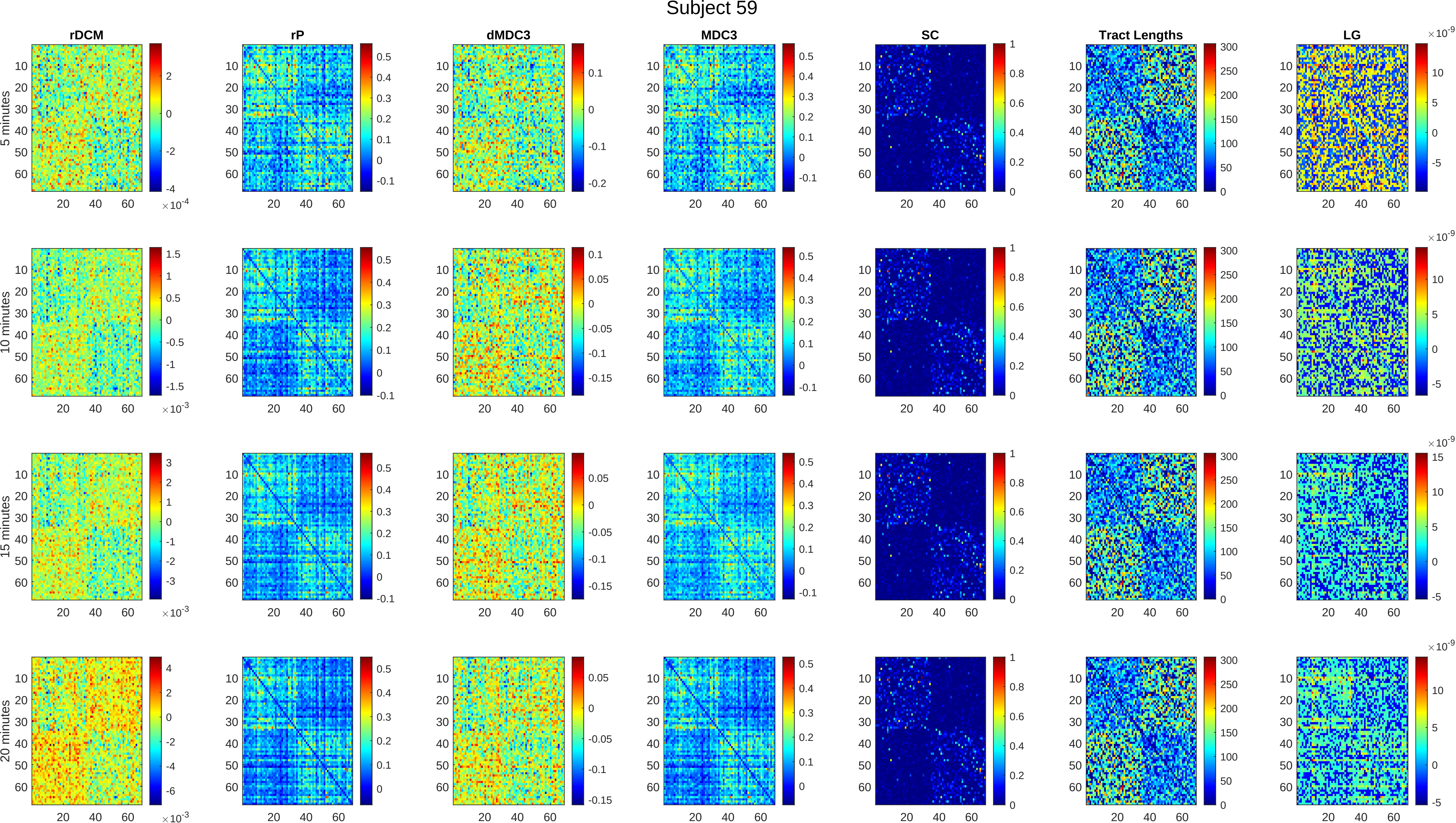
Subject 59
rDCM
rP
dMDC3
MDC3
SC
Tract Lengths
LG
5 minutes
10 minutes
15 minutes
20 minutes

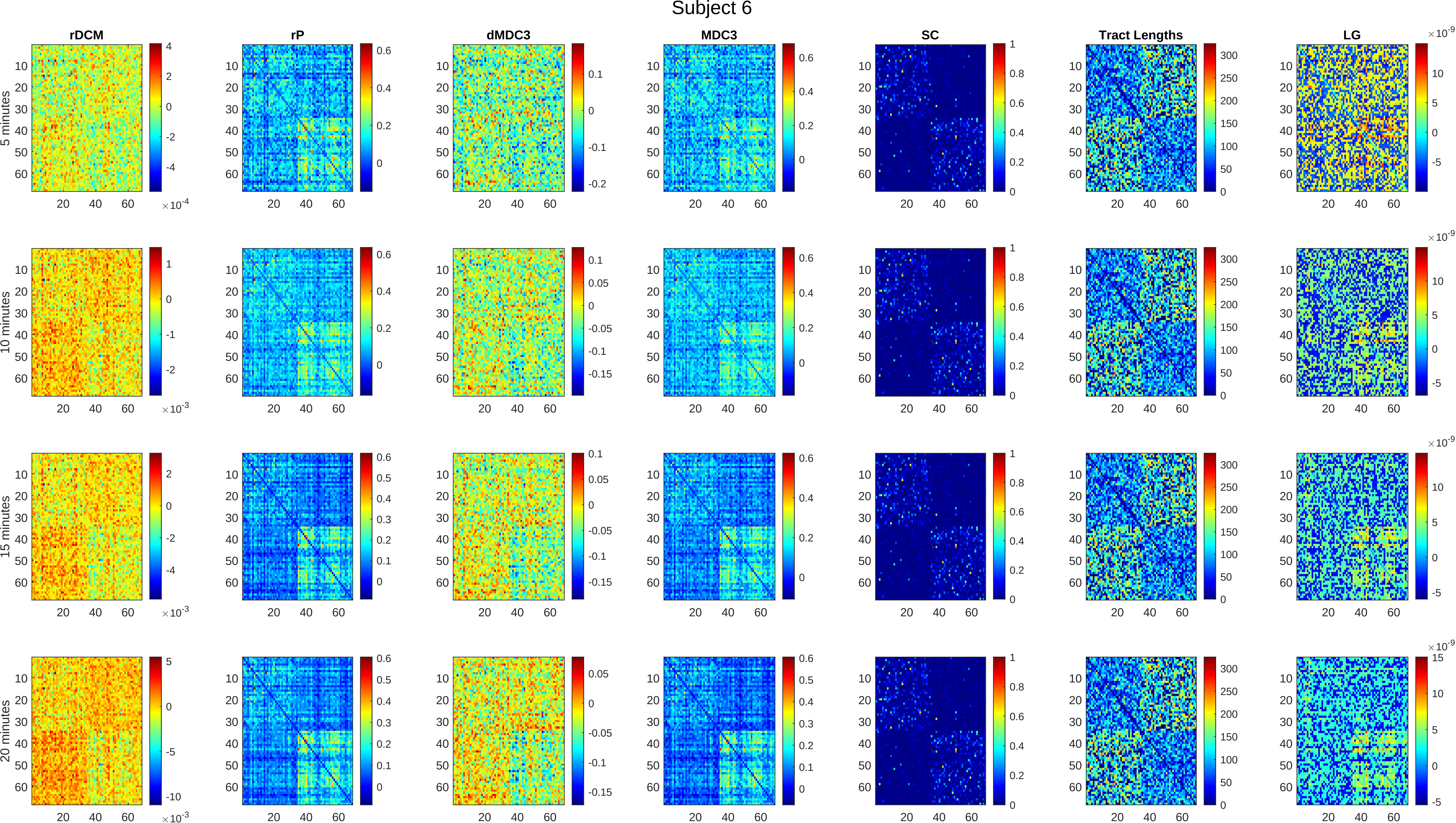
Subject 6
rDCM
rP
dMDC3
MDC3
SC
Tract Lengths
LG
5 minutes
10 minutes
15 minutes
20 minutes

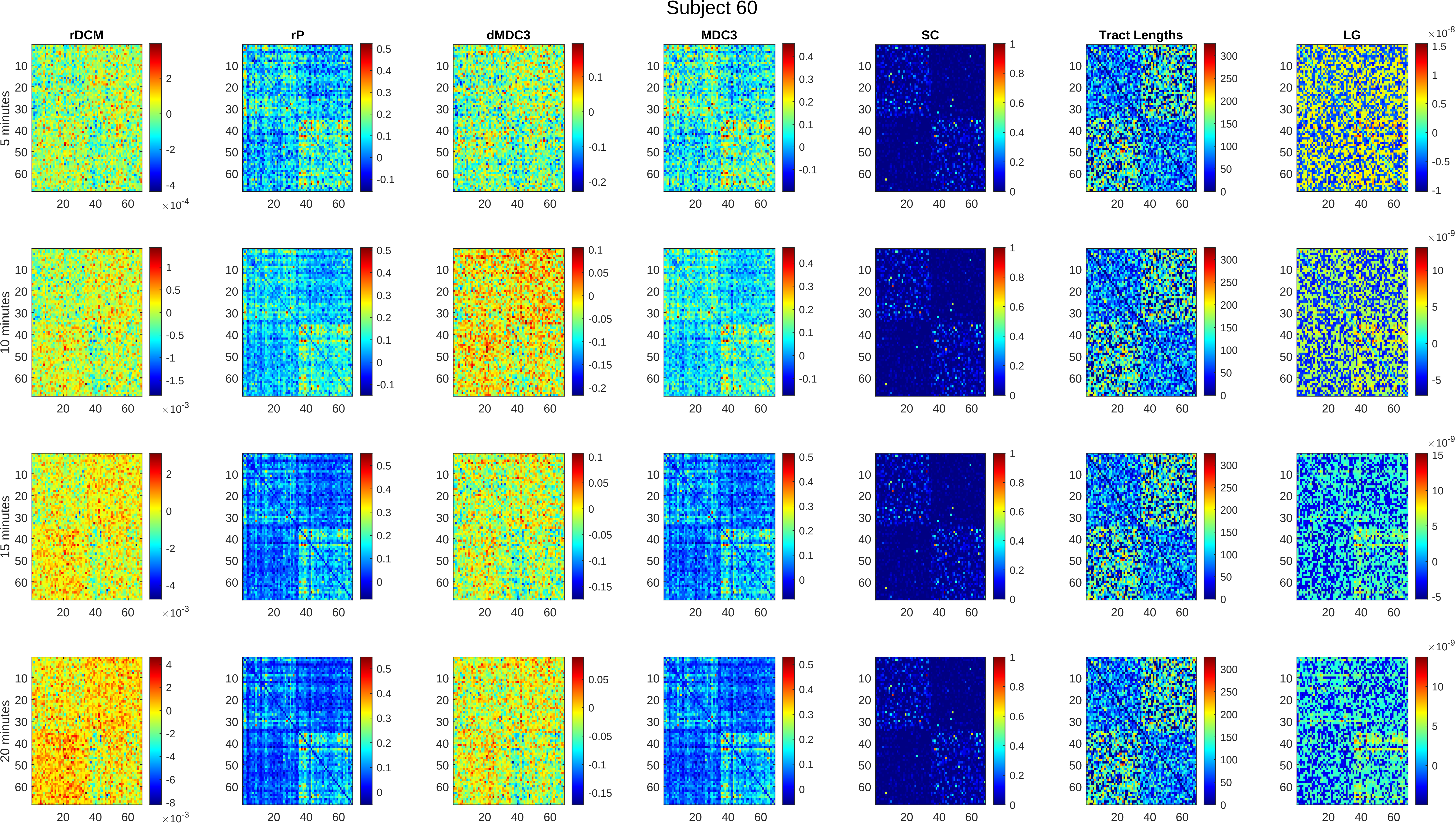
Subject 60
rDCM
rP
dMDC3
MDC3
SC
Tract Lengths
LG
5 minutes
10 minutes
15 minutes
20 minutes

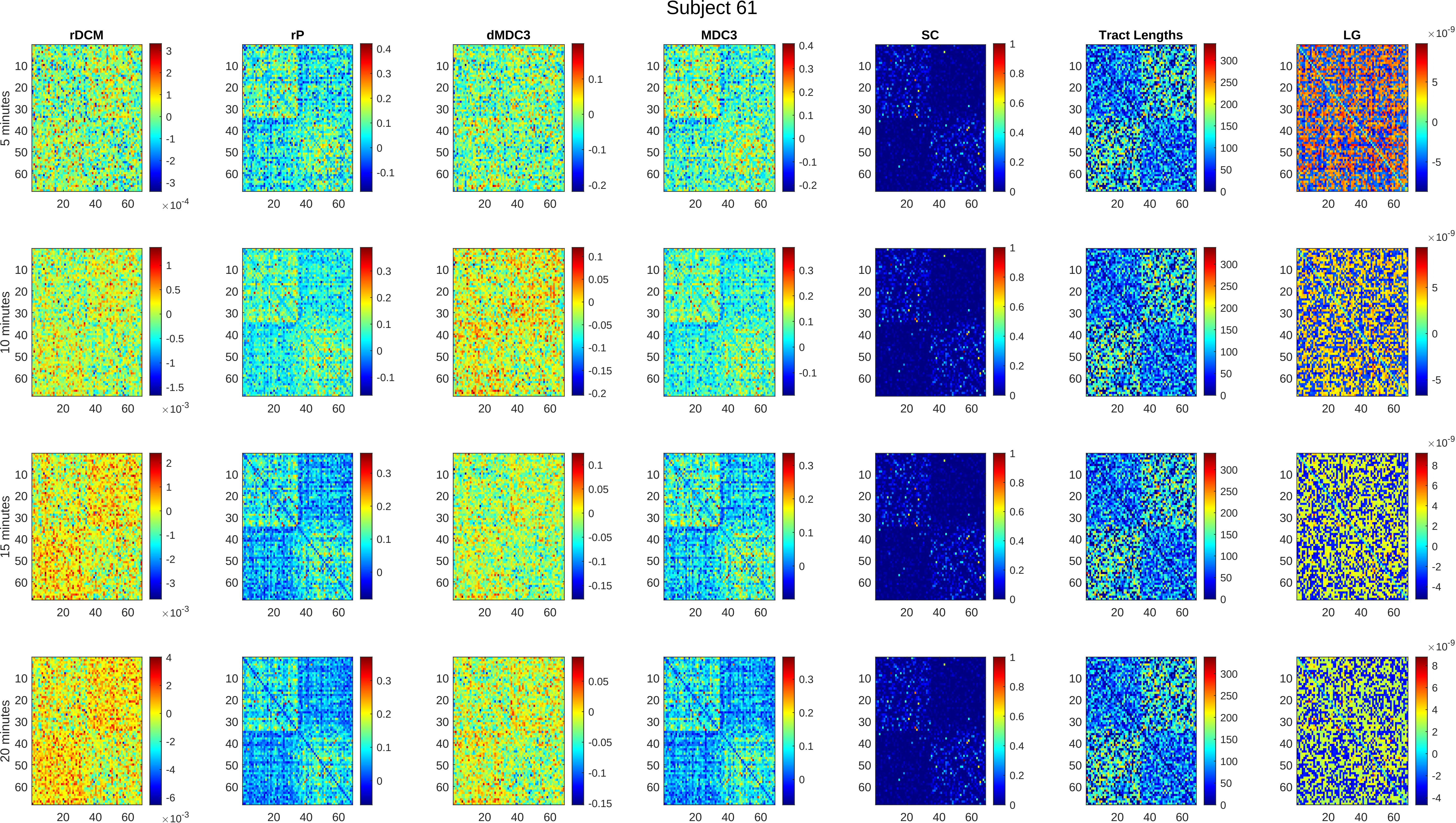

Subject 61
rDCM
rP
dMDC3
MDC3
SC
Tract Lengths
LG
5 minutes
10 minutes
15 minutes
20 minutes
×10^-4
×10^-3
×10^-3
×10^-3
×10^-9

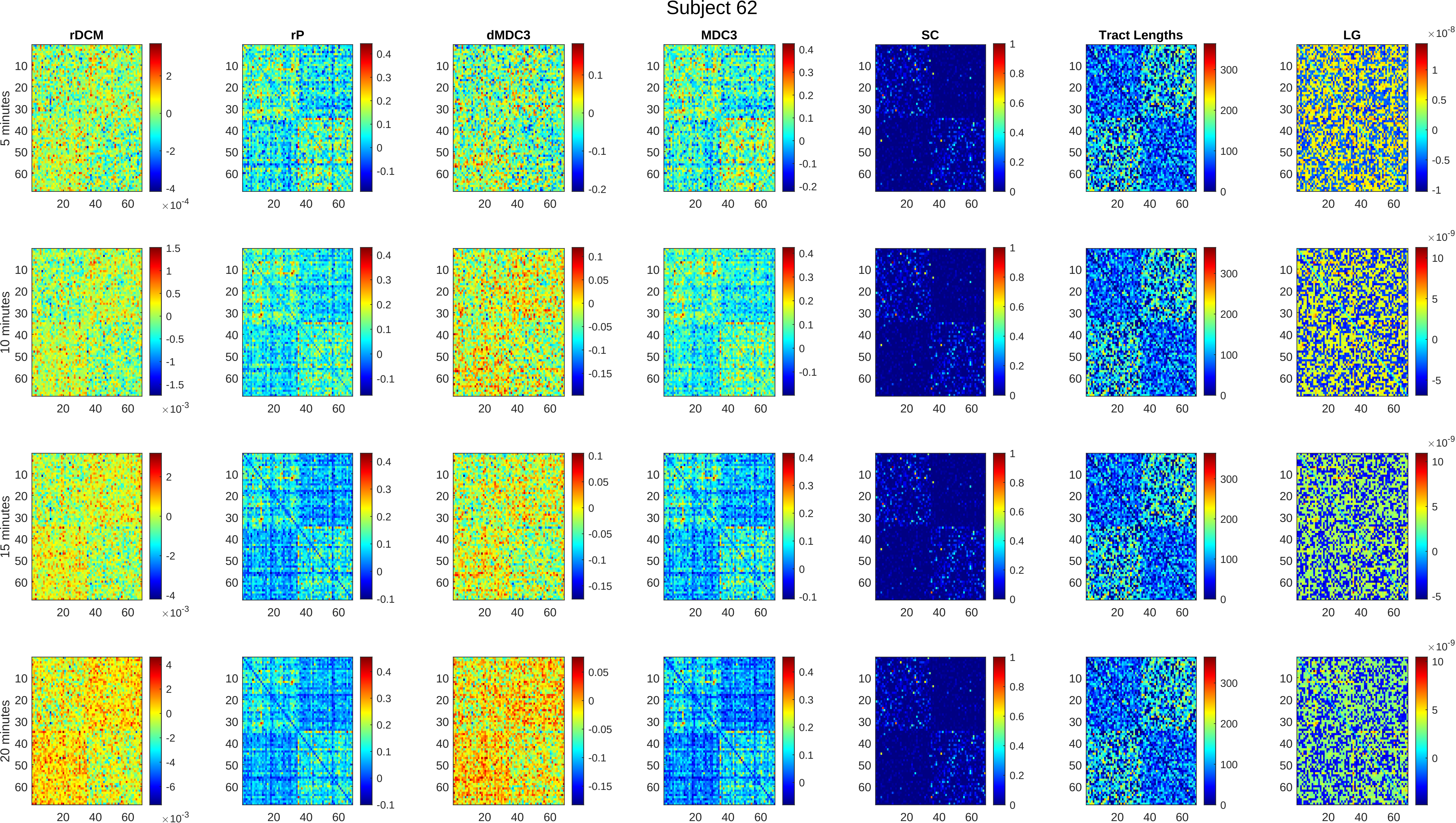

Subject 62
rDCM
rP
dMDC3
MDC3
SC
Tract Lengths
LG
5 minutes
10 minutes
15 minutes
20 minutes

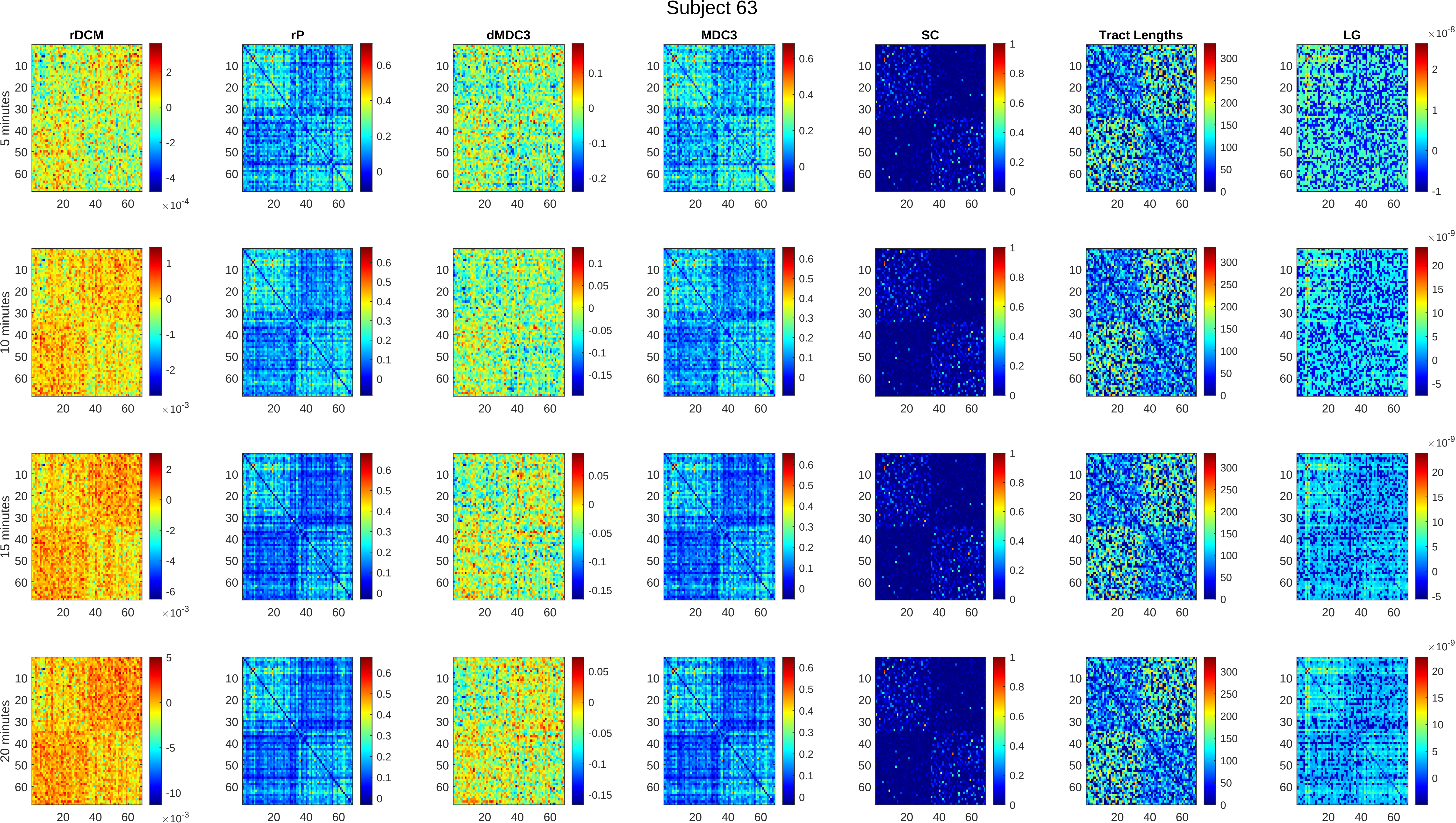
Subject 63
rDCM
rP
dMDC3
MDC3
SC
Tract Lengths
LG
5 minutes
10 minutes
15 minutes
20 minutes

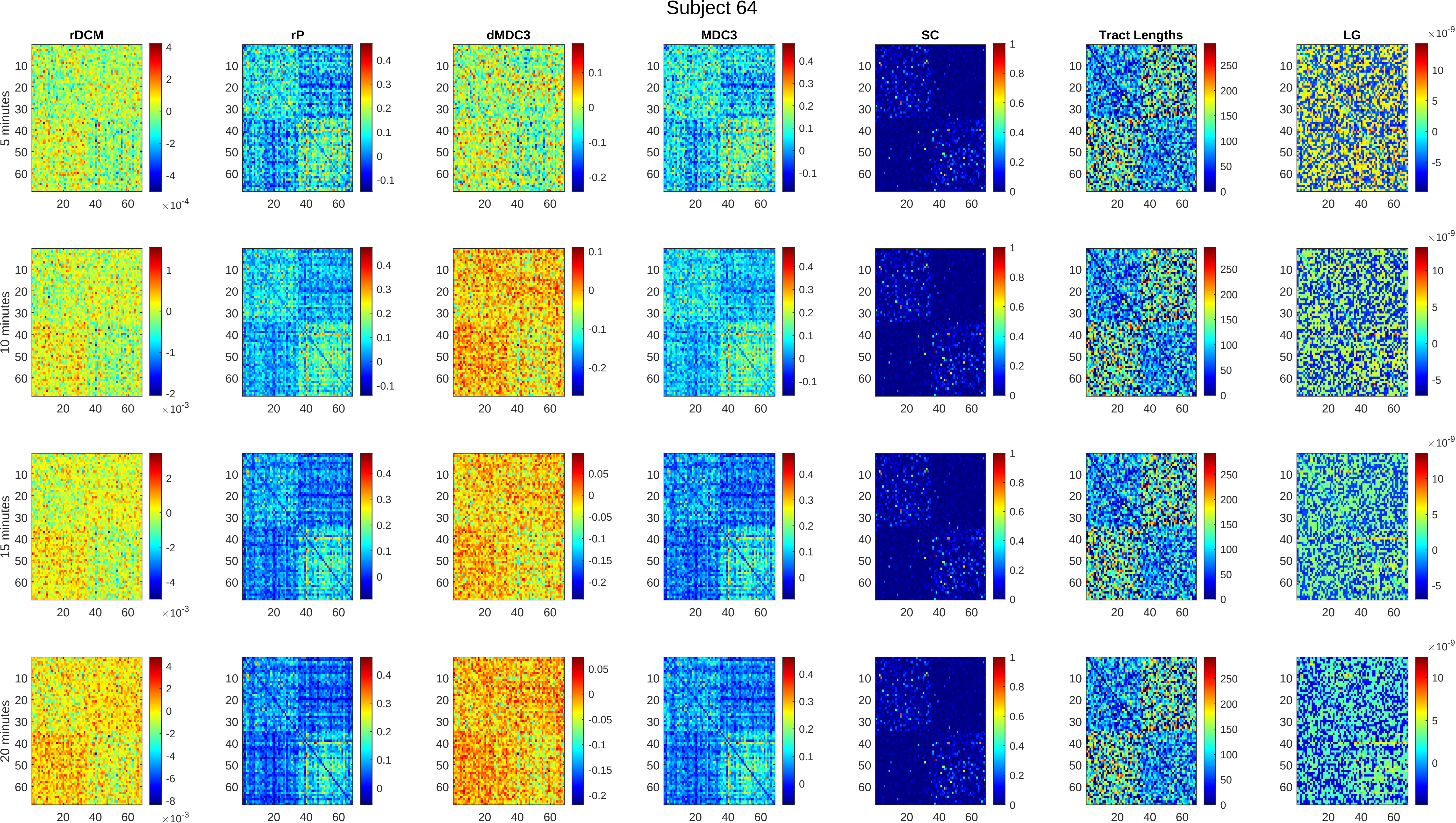

Subject 64
rDCM
rP
dMDC3
MDC3
SC
Tract Lengths
LG
5 minutes
10 minutes
15 minutes
20 minutes

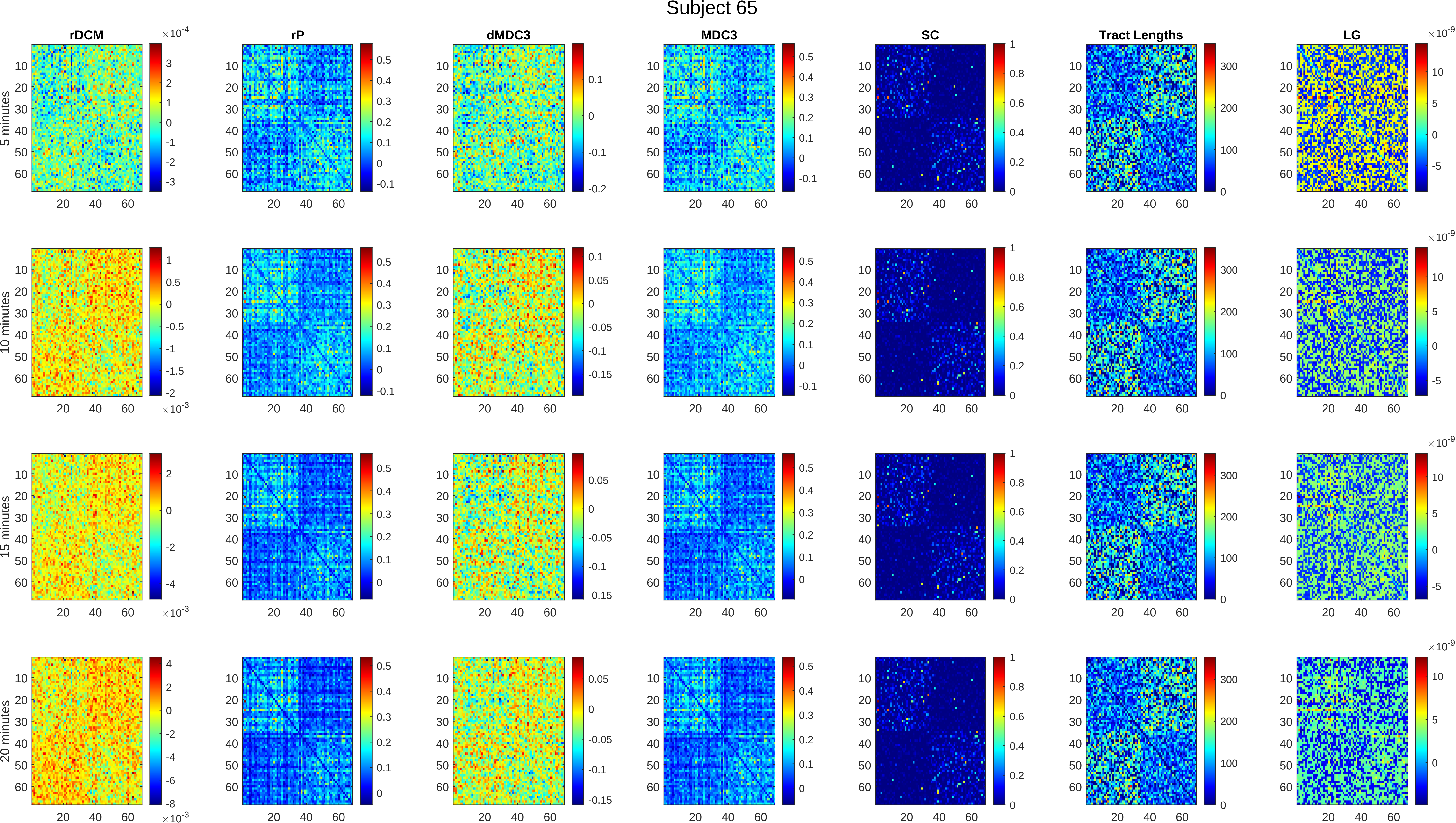

Subject 65
rDCM
rP
dMDC3
MDC3
SC
Tract Lengths
LG
5 minutes
10 minutes
15 minutes
20 minutes

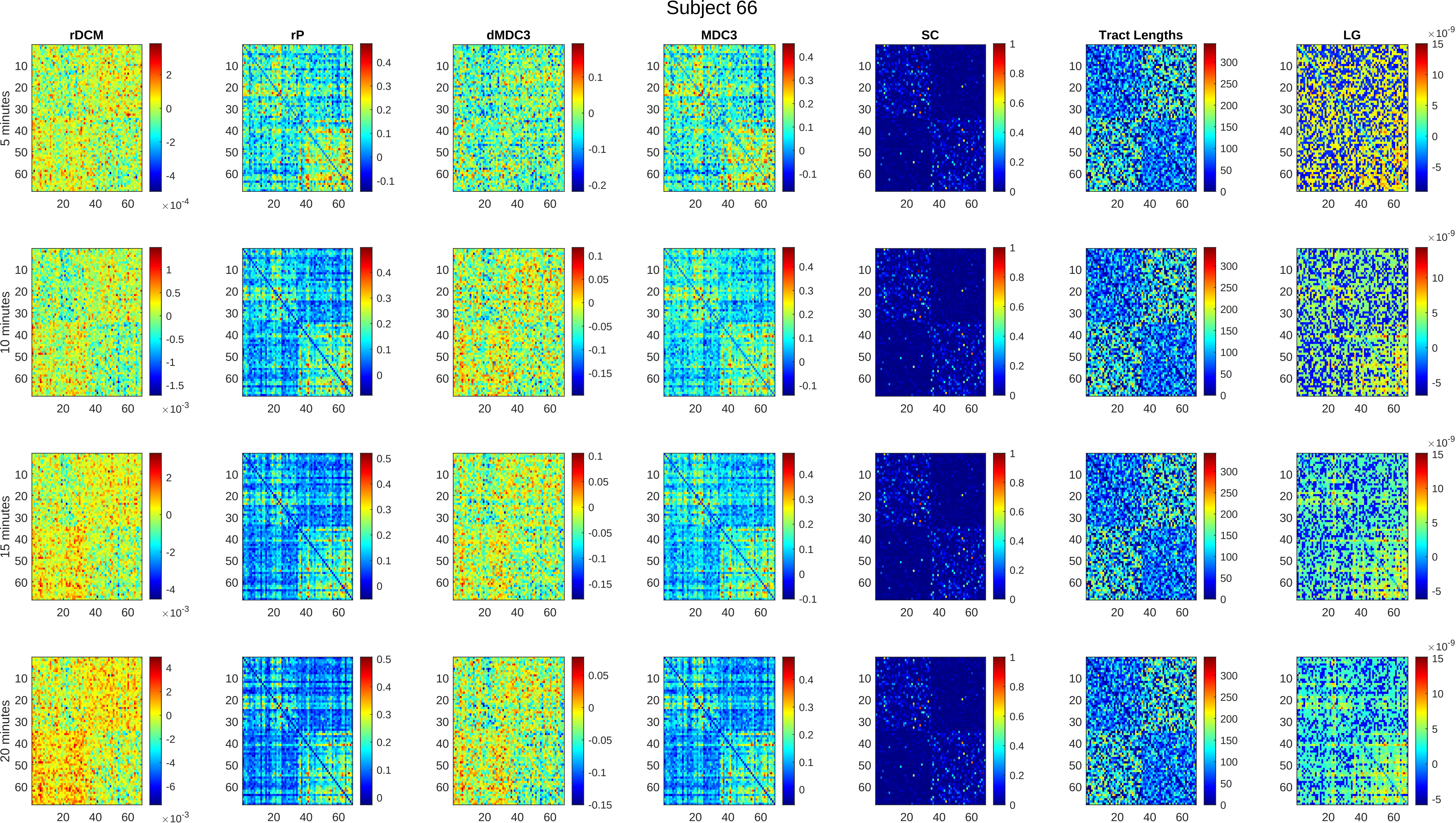
Subject 66
rDCM
rP
dMDC3
MDC3
SC
Tract Lengths
LG
5 minutes
10 minutes
15 minutes
20 minutes
×10^-4
×10^-3
×10^-3
×10^-3
×10^-9
×10^-9
×10^-9
×10^-9

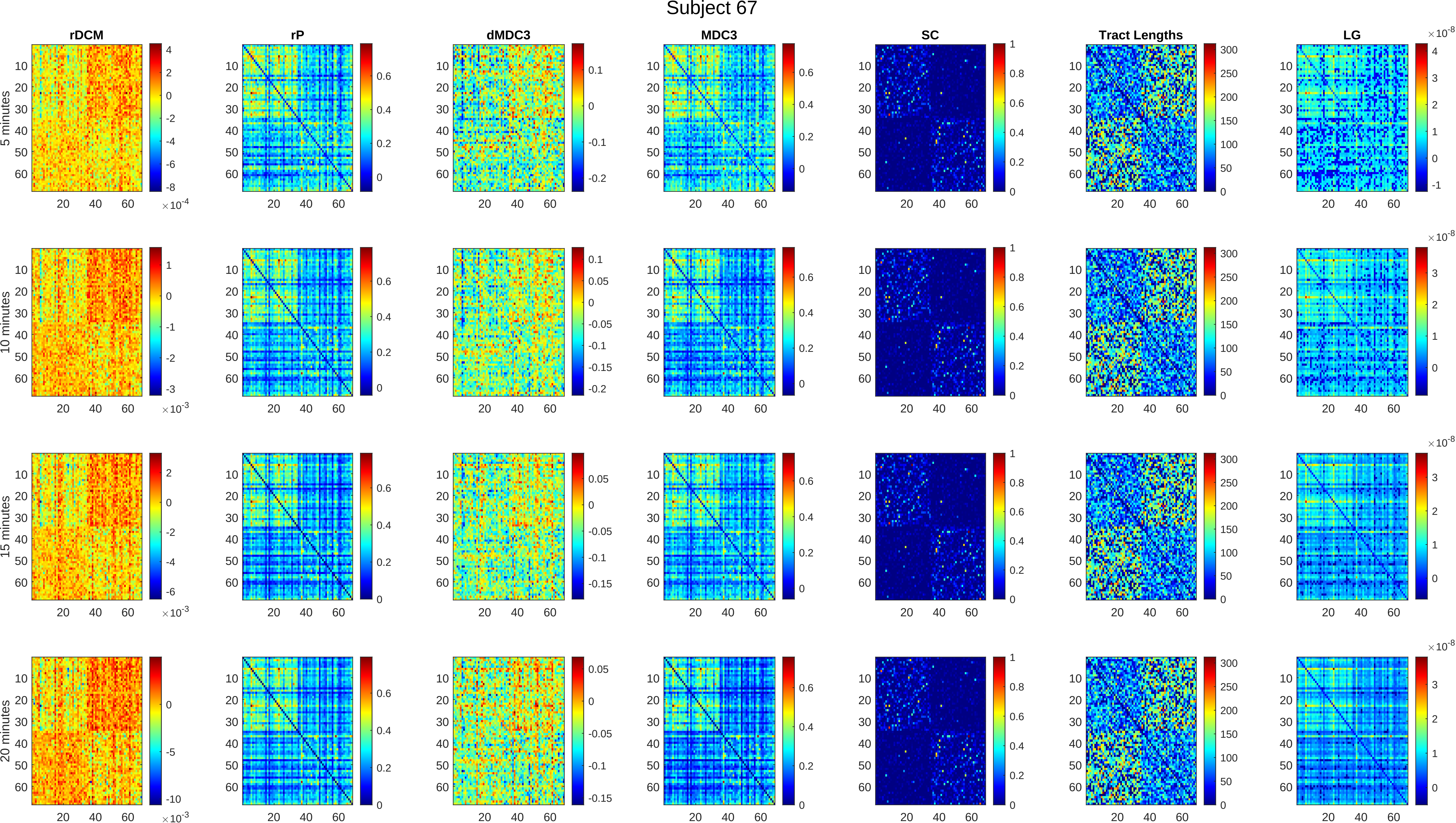

Subject 67
rDCM
rP
dMDC3
MDC3
SC
Tract Lengths
LG
5 minutes
10 minutes
15 minutes
20 minutes

Subject 68

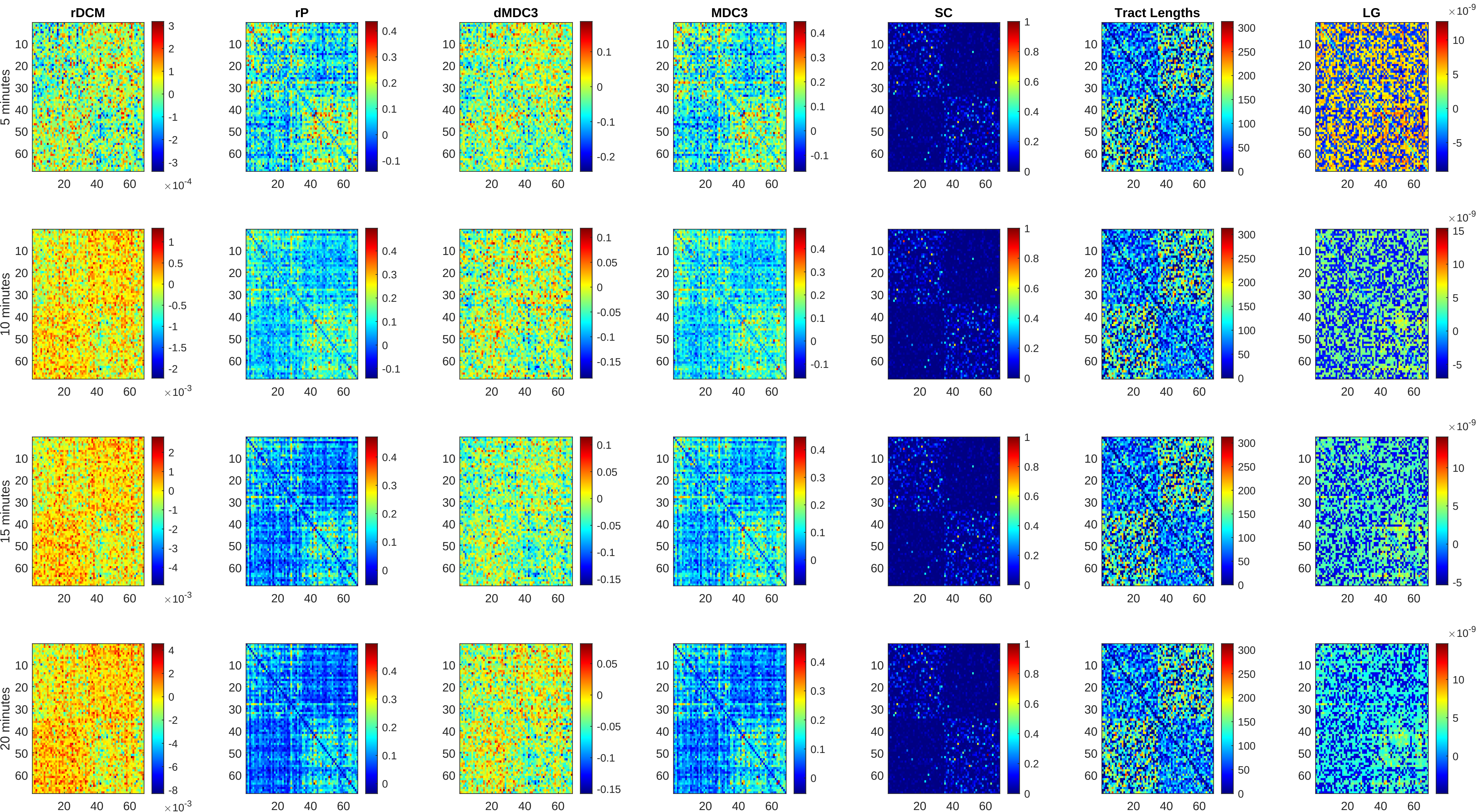

rDCM
rP
dMDC3
MDC3
SC
Tract Lengths
LG
5 minutes
10 minutes
15 minutes
20 minutes

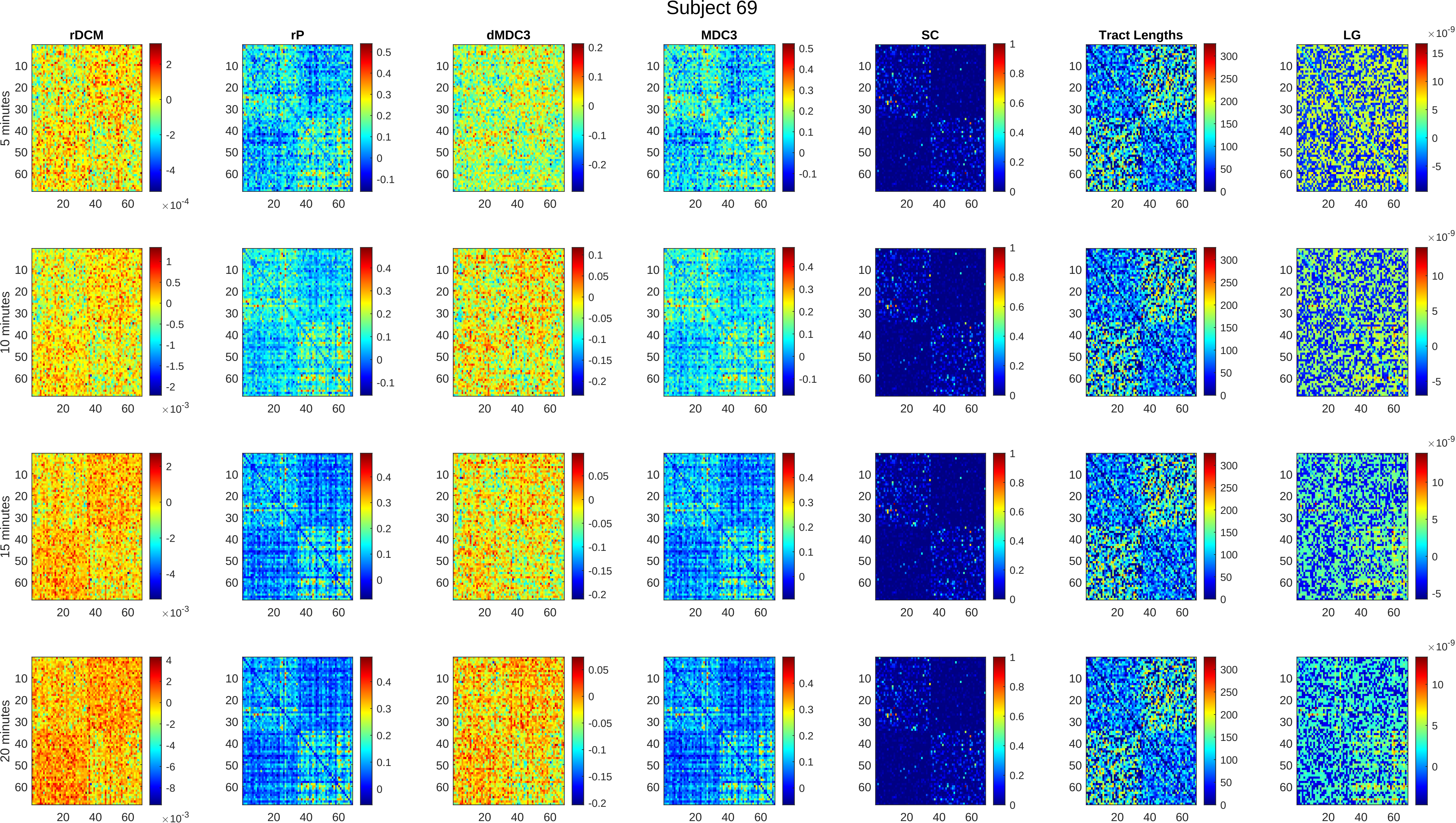

Subject 69
rDCM
rP
dMDC3
MDC3
SC
Tract Lengths
LG
5 minutes
10 minutes
15 minutes
20 minutes

Subject 7

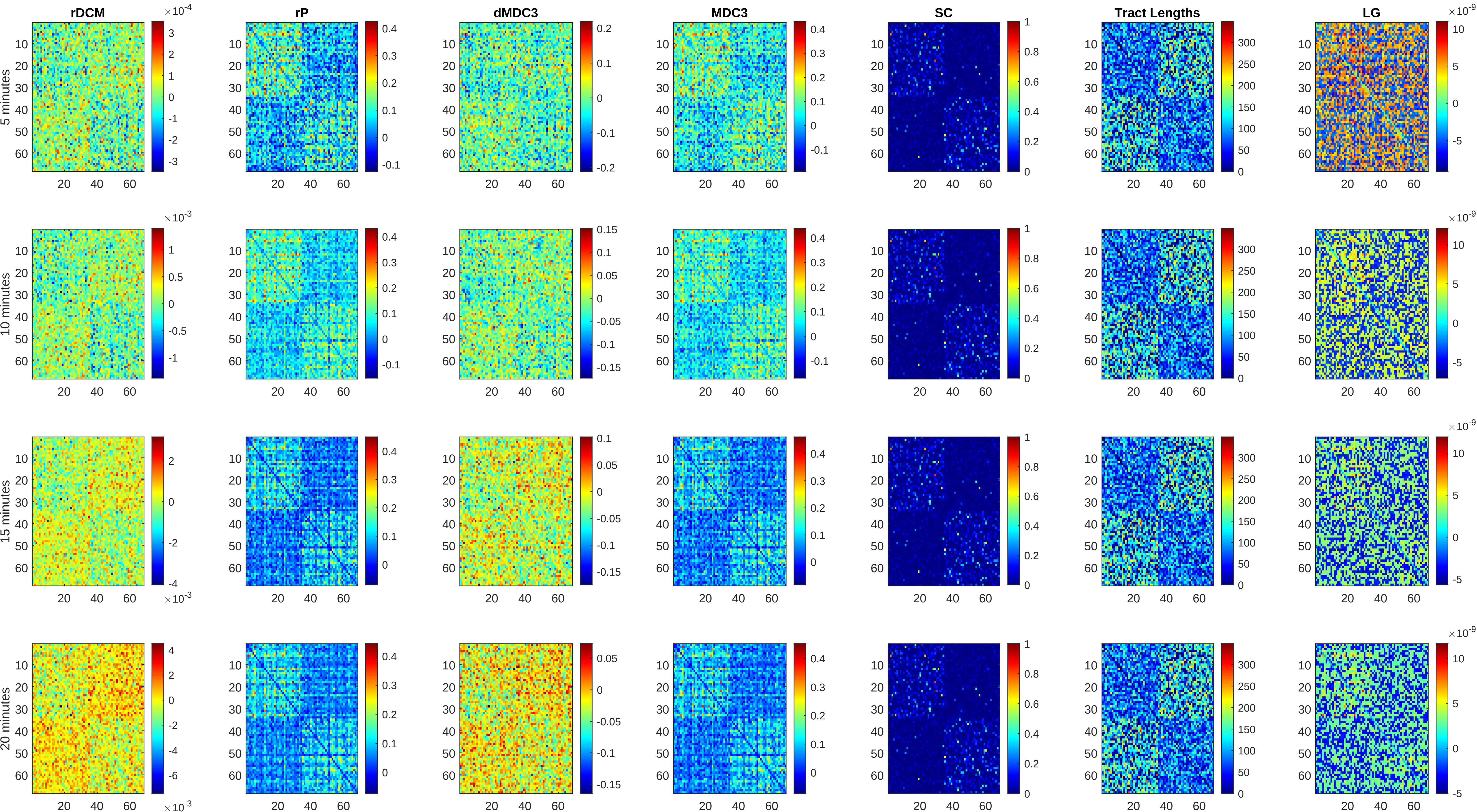
rDCM
rP
dMDC3
MDC3
SC
Tract Lengths
LG
5 minutes
10 minutes
15 minutes
20 minutes

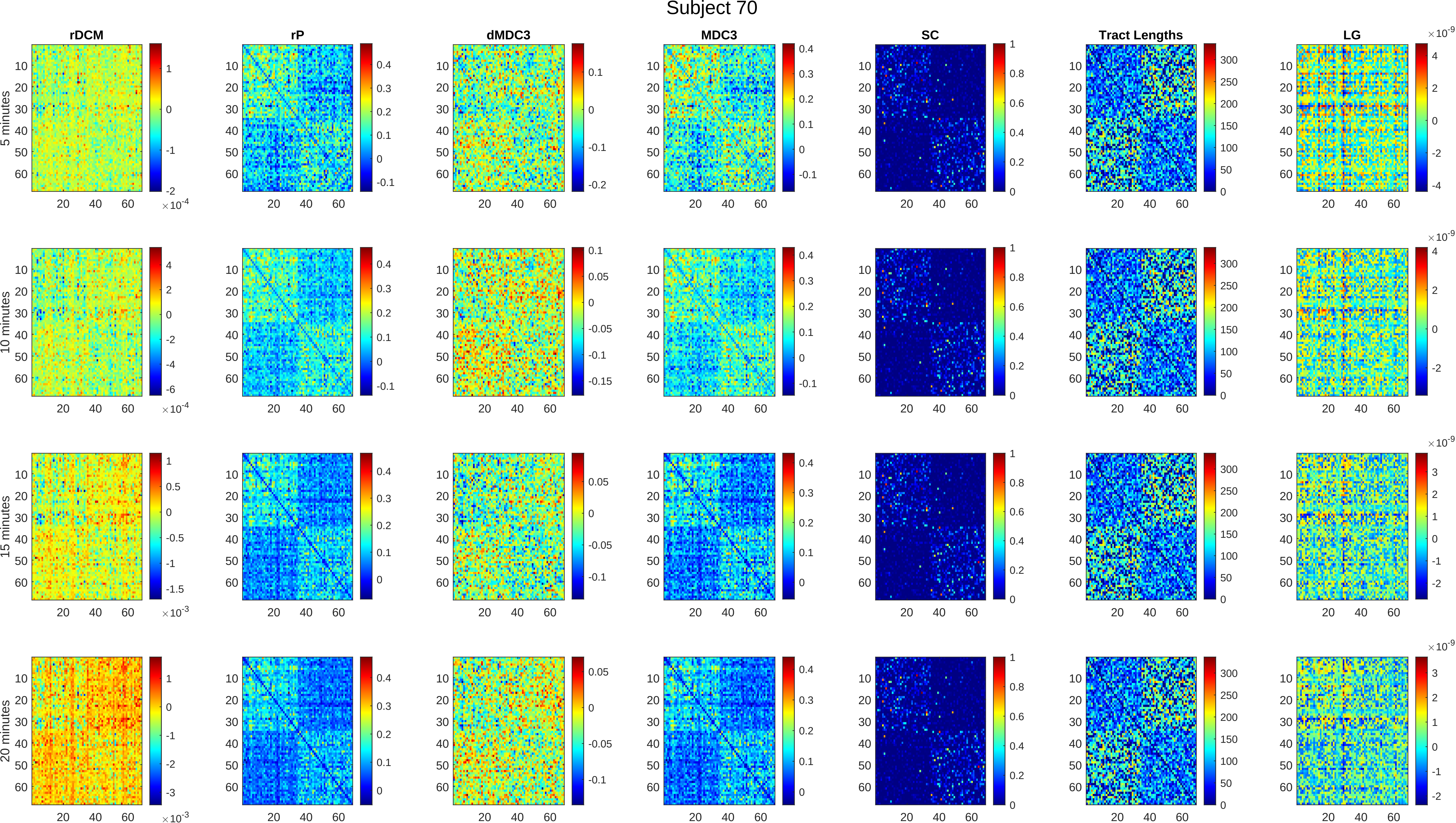

Subject 70
rDCM
rP
dMDC3
MDC3
SC
Tract Lengths
LG
5 minutes
10 minutes
15 minutes
20 minutes

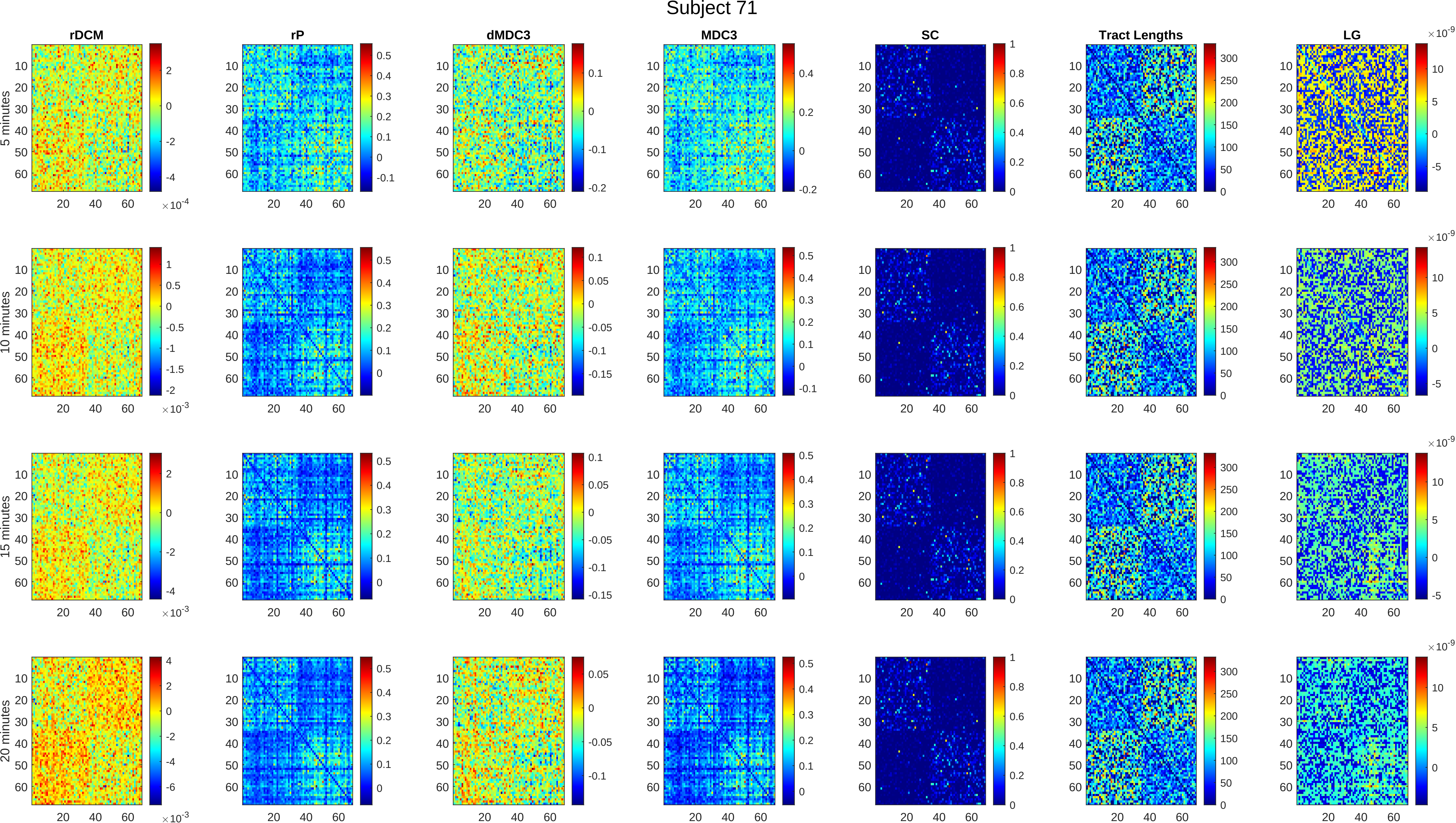

Subject 71
rDCM
rP
dMDC3
MDC3
SC
Tract Lengths
LG
5 minutes
10 minutes
15 minutes
20 minutes

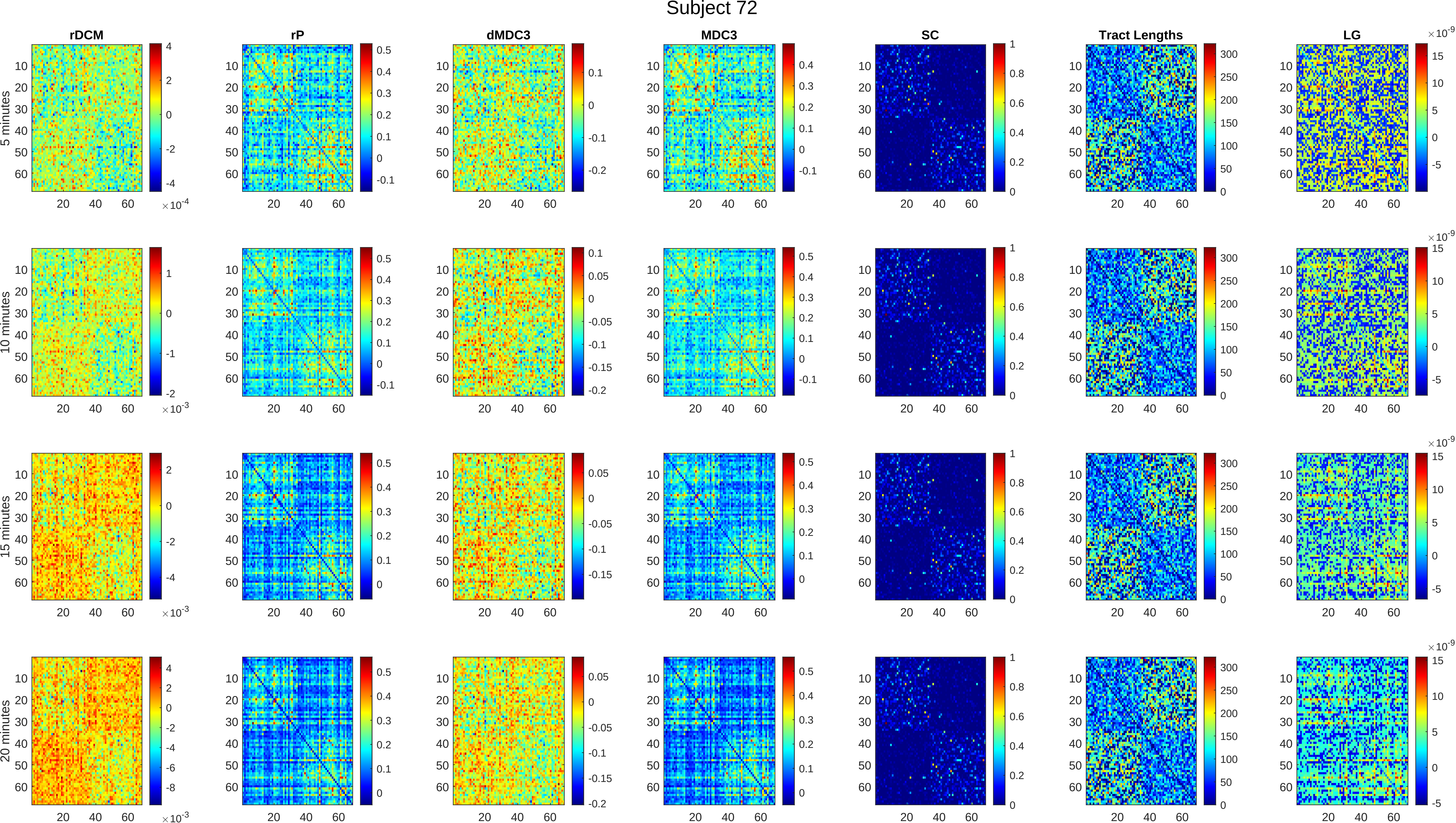

Subject 72
rDCM
rP
dMDC3
MDC3
SC
Tract Lengths
LG
5 minutes
10 minutes
15 minutes
20 minutes

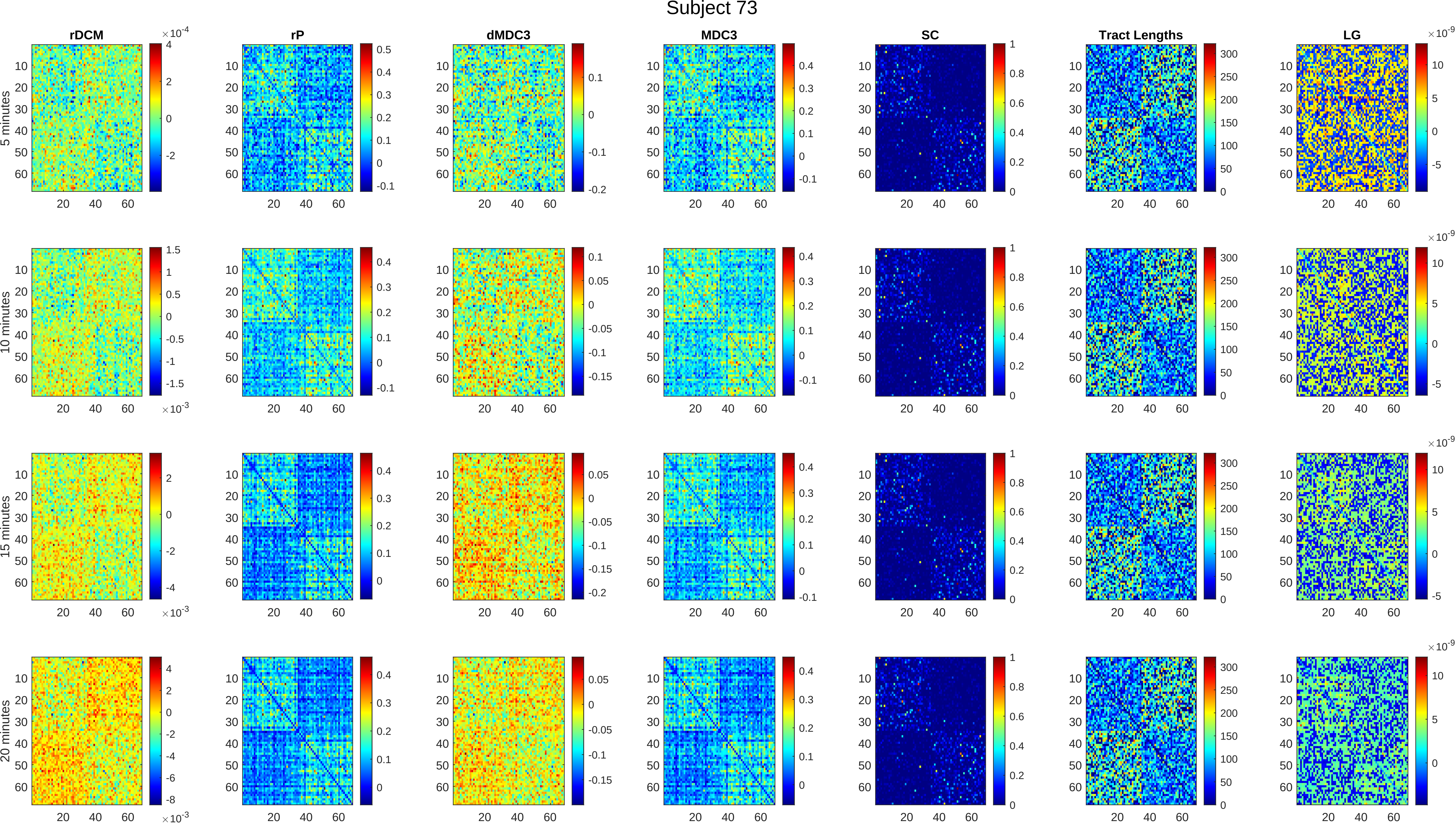

Subject 73
rDCM
rP
dMDC3
MDC3
SC
Tract Lengths
LG
5 minutes
10 minutes
15 minutes
20 minutes

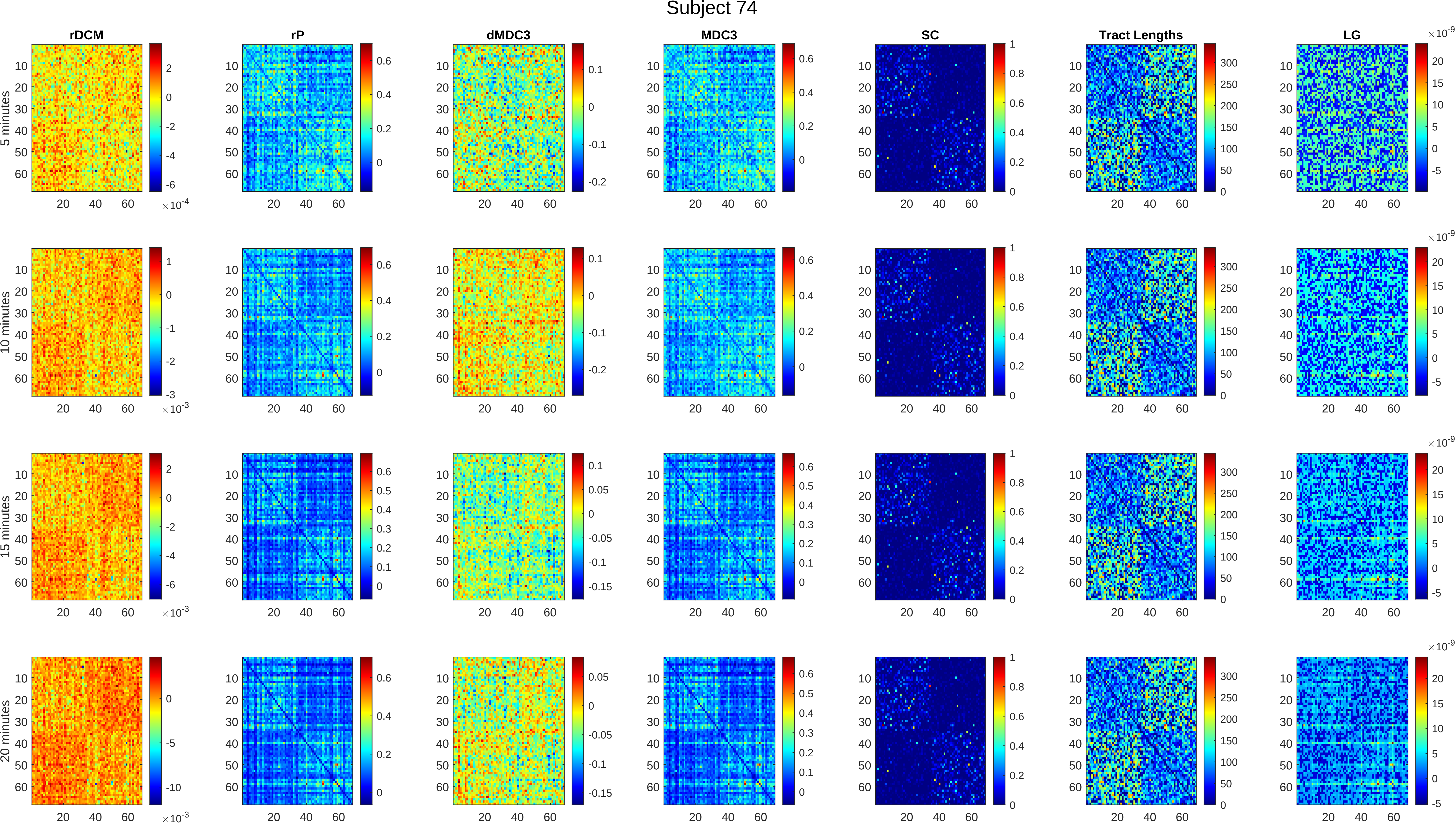

Subject 74
rDCM
rP
dMDC3
MDC3
SC
Tract Lengths
LG
5 minutes
10 minutes
15 minutes
20 minutes

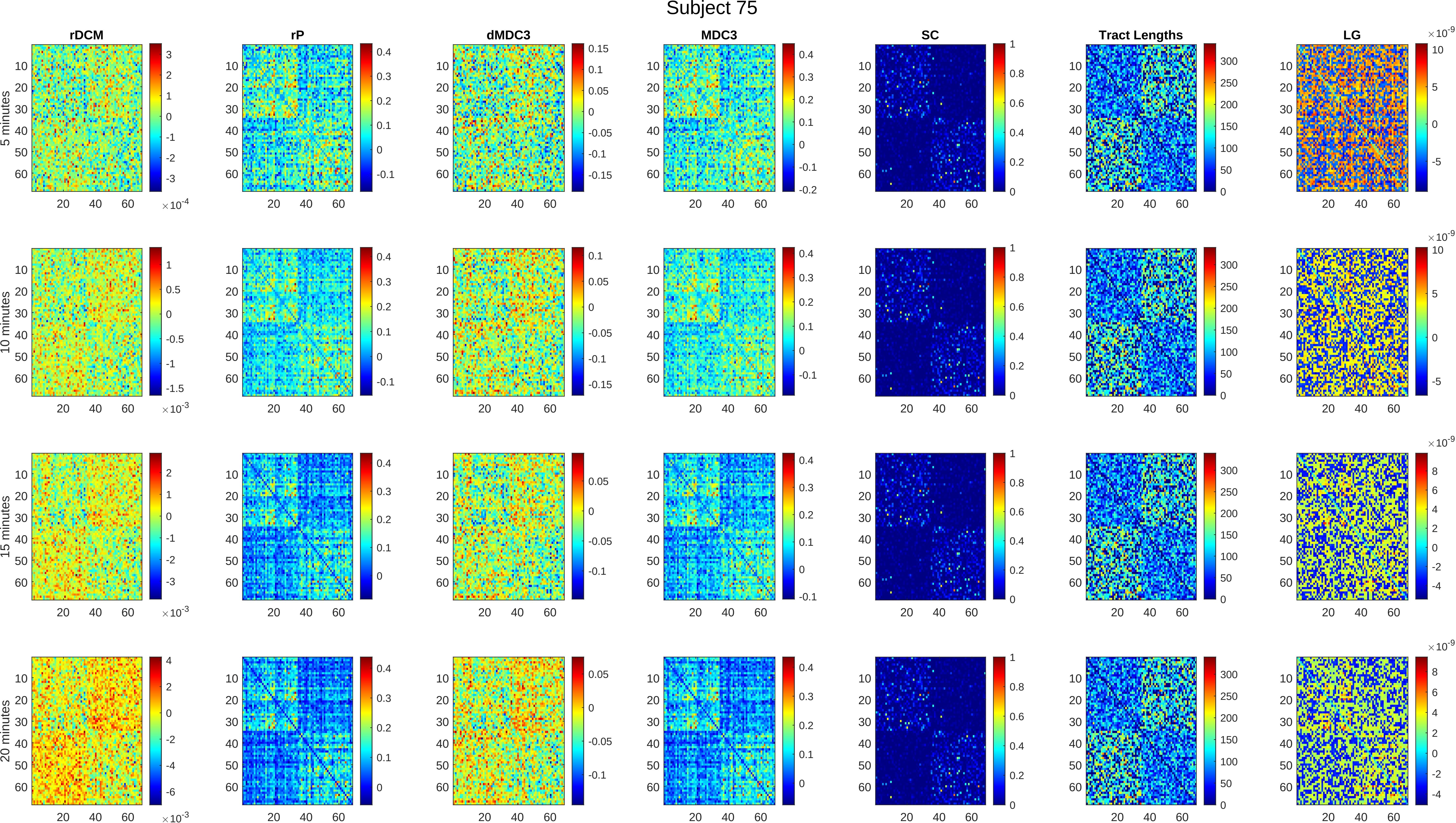

Subject 75
rDCM
rP
dMDC3
MDC3
SC
Tract Lengths
LG
5 minutes
10 minutes
15 minutes
20 minutes

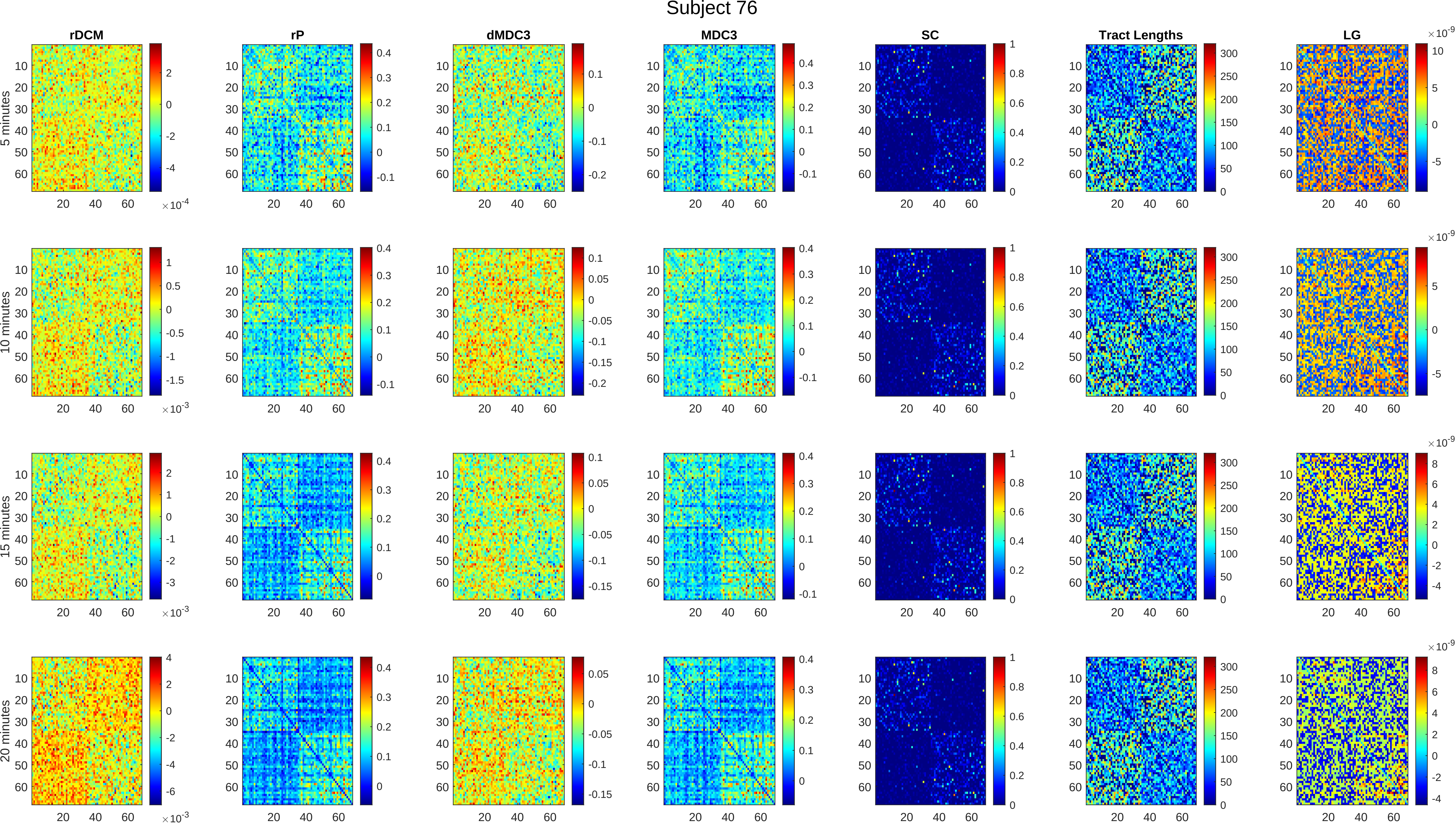
Subject 76
rDCM
rP
dMDC3
MDC3
SC
Tract Lengths
LG
5 minutes
10 minutes
15 minutes
20 minutes

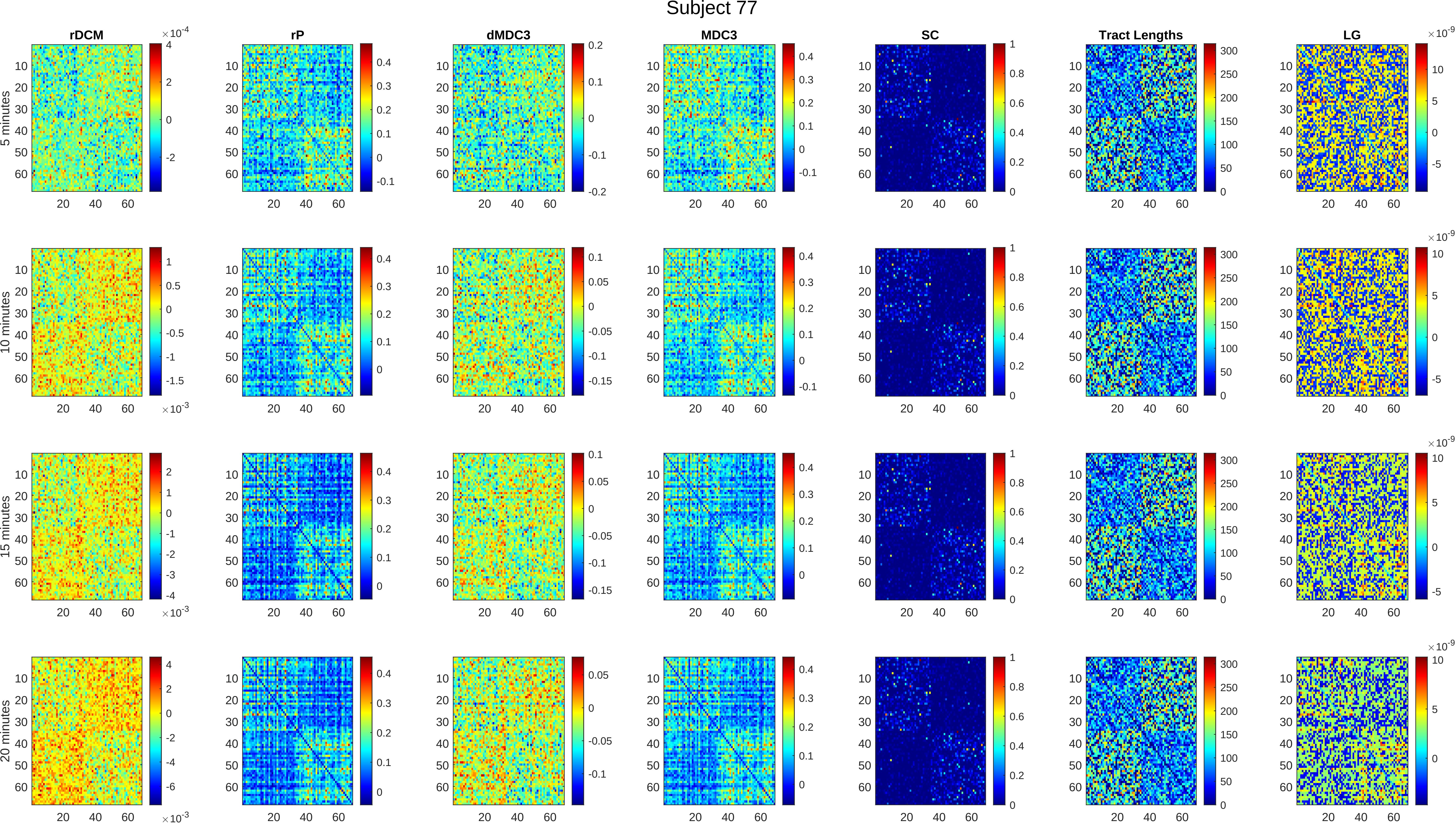
Subject 77
rDCM
rP
dMDC3
MDC3
SC
Tract Lengths
LG
5 minutes
10 minutes
15 minutes
20 minutes

Subject 78

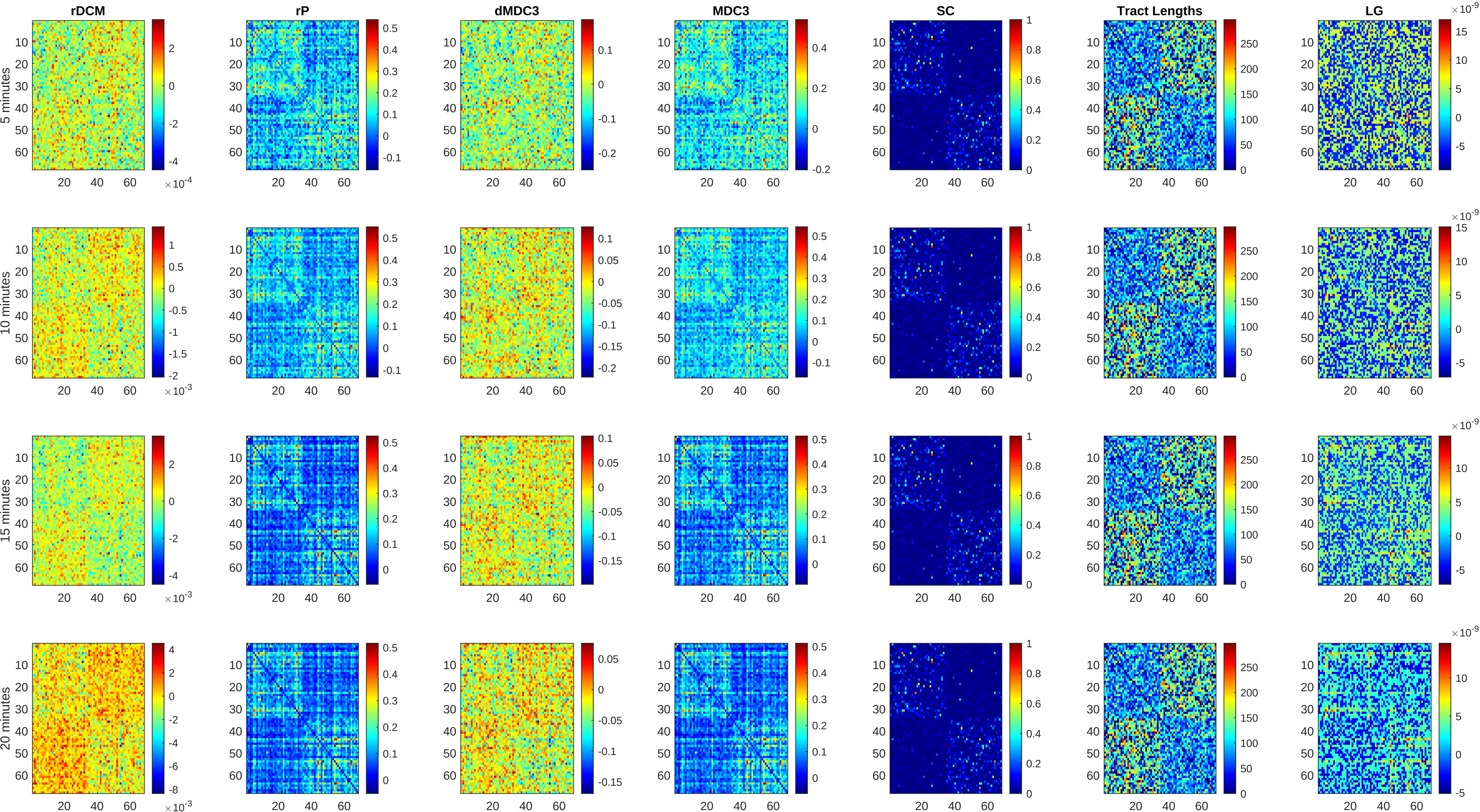
rDCM
rP
dMDC3
MDC3
SC
Tract Lengths
LG
5 minutes
10 minutes
15 minutes
20 minutes

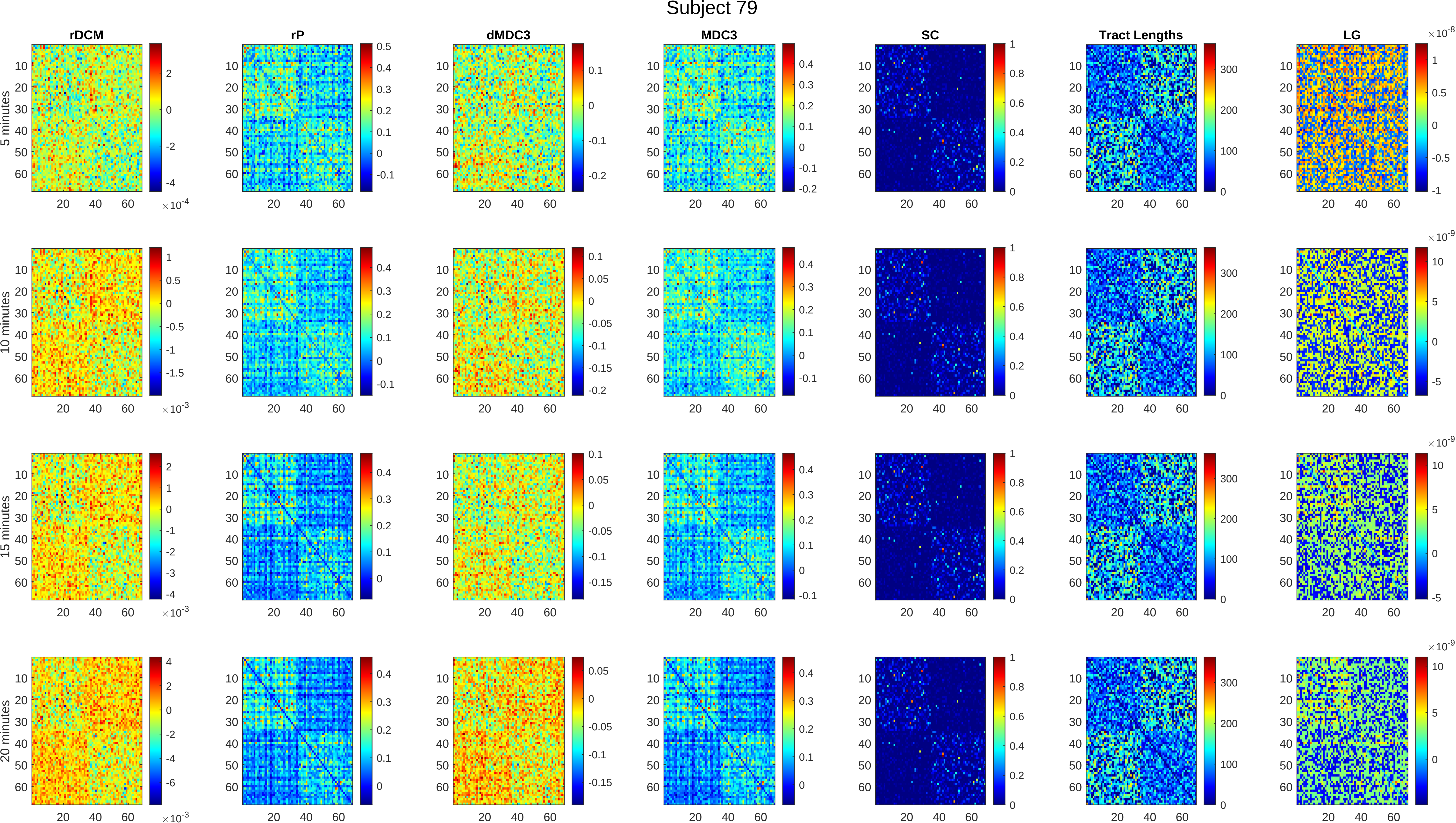

Subject 79
rDCM
rP
dMDC3
MDC3
SC
Tract Lengths
LG
5 minutes
10 minutes
15 minutes
20 minutes

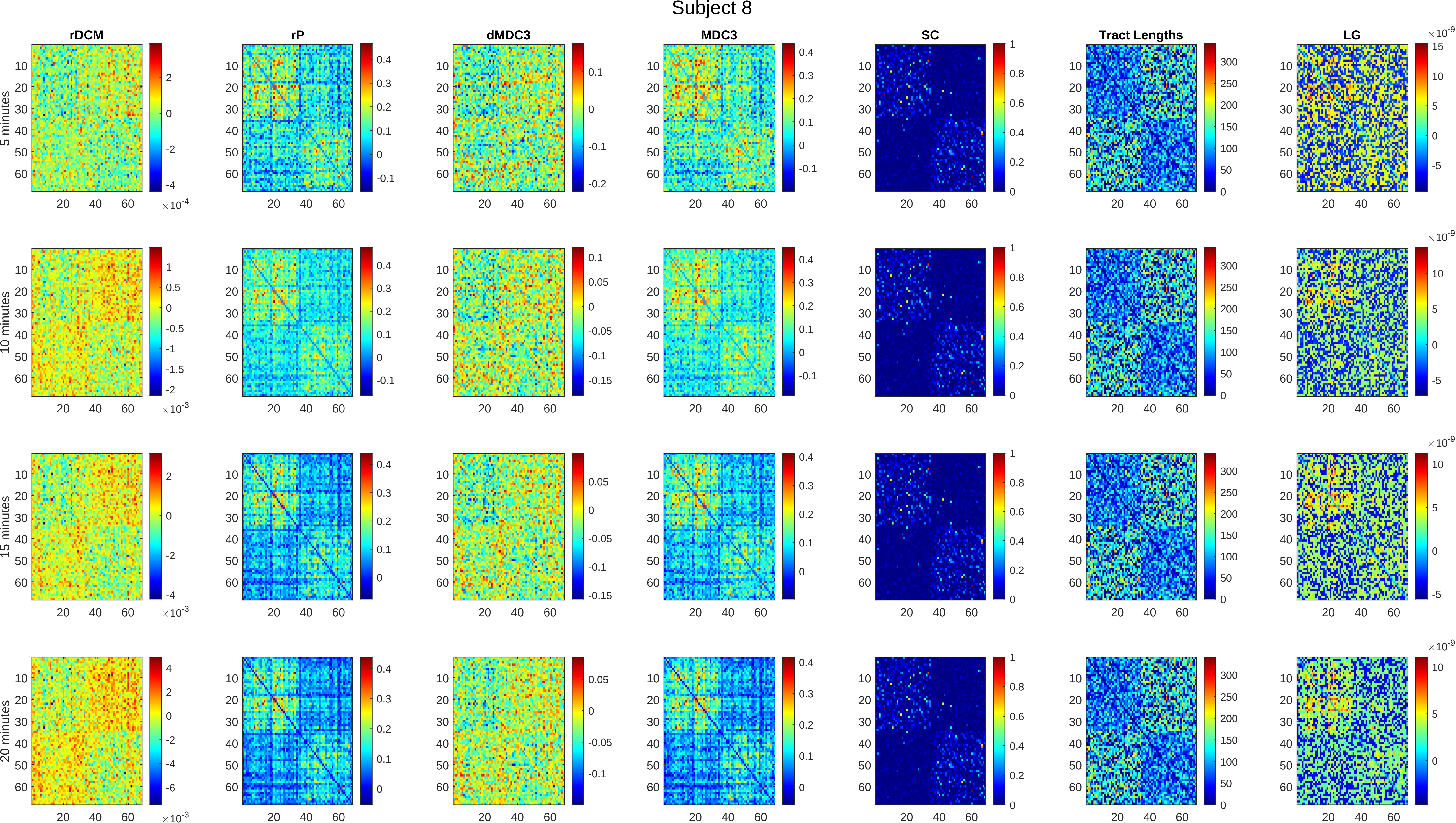

Subject 8
rDCM
rP
dMDC3
MDC3
SC
Tract Lengths
LG
5 minutes
10 minutes
15 minutes
20 minutes

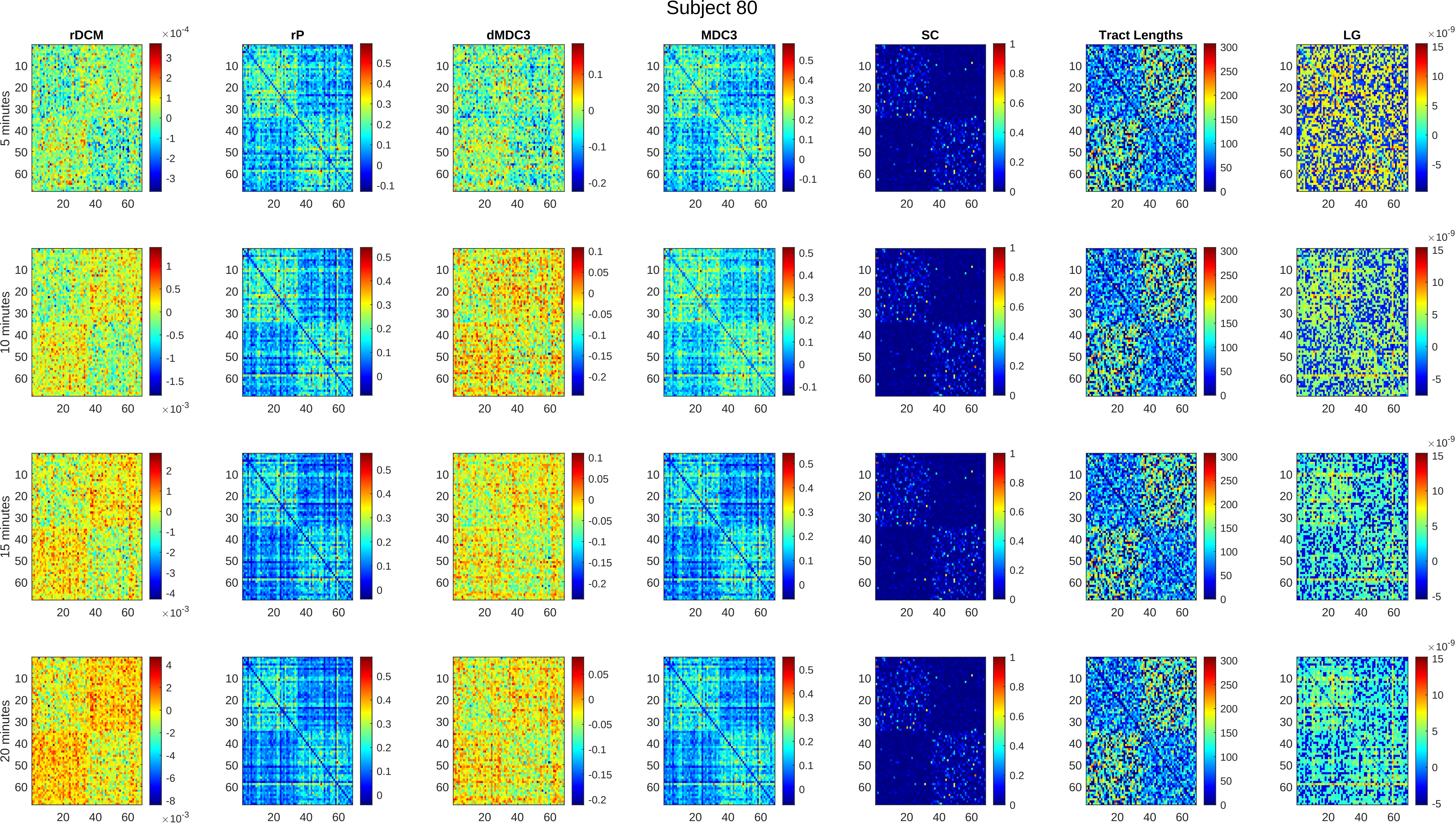
Subject 80
rDCM
rP
dMDC3
MDC3
SC
Tract Lengths
LG
5 minutes
10 minutes
15 minutes
20 minutes

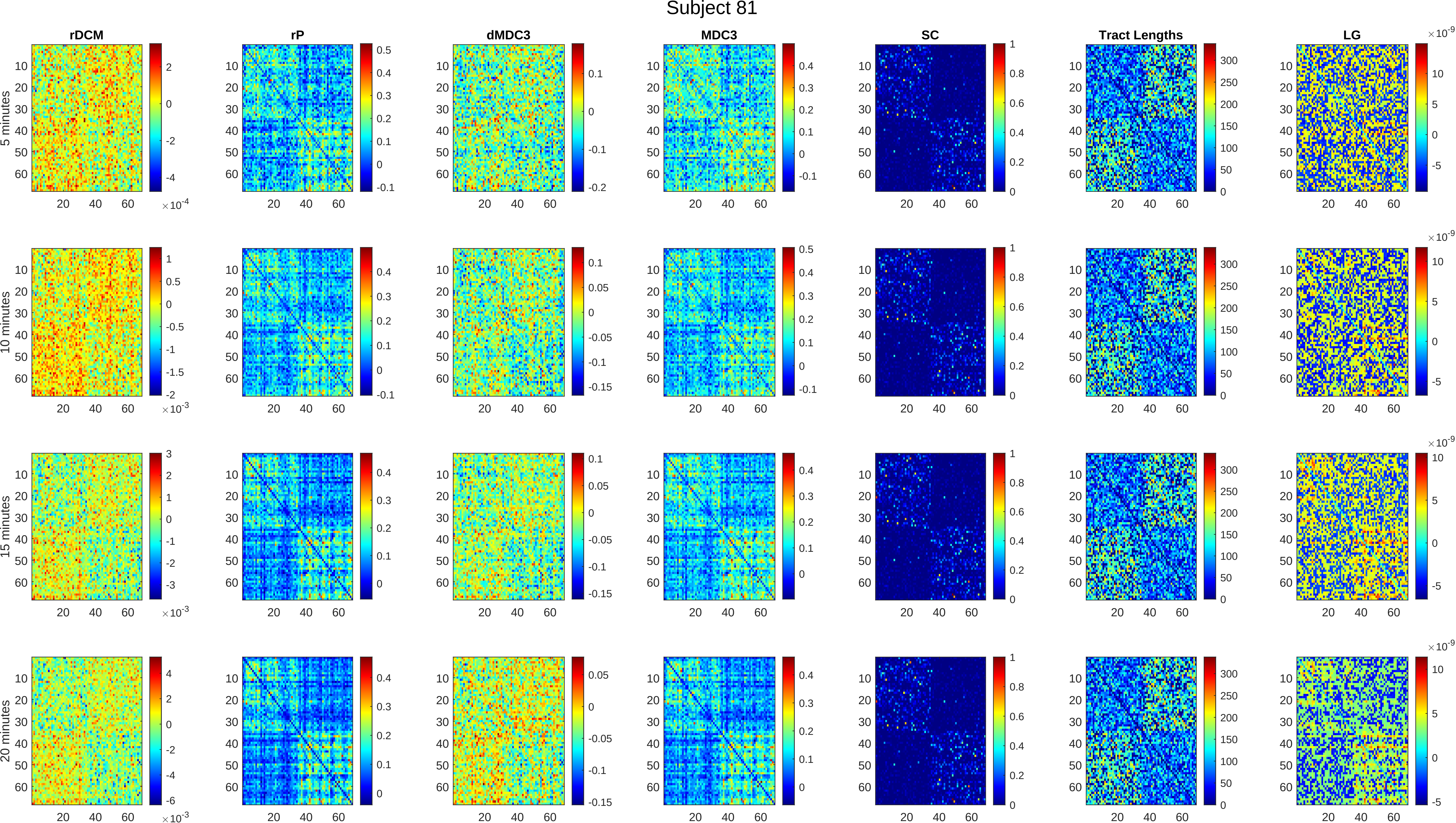

Subject 81
rDCM
rP
dMDC3
MDC3
SC
Tract Lengths
LG
5 minutes
10 minutes
15 minutes
20 minutes

Subject 82

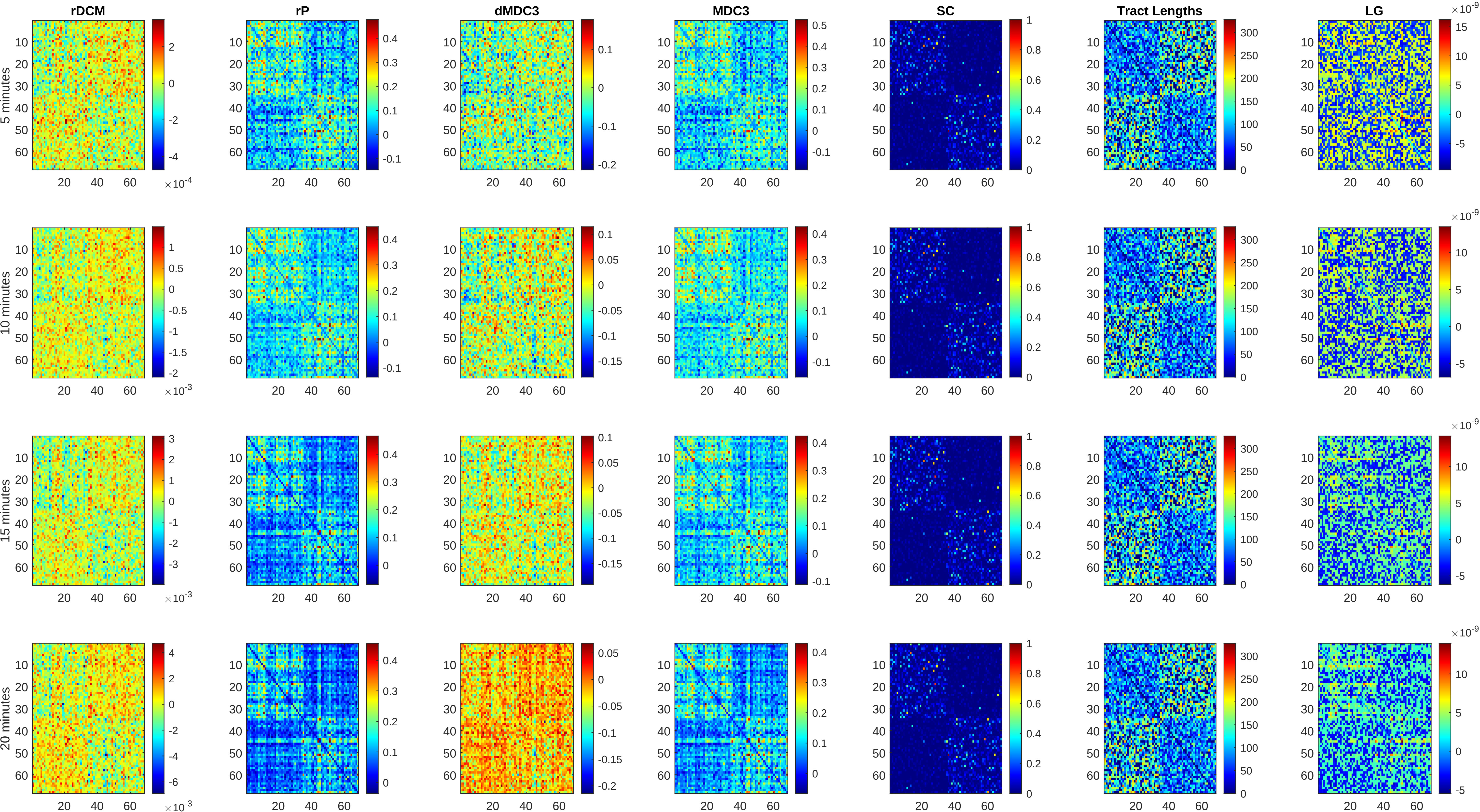

rDCM
rP
dMDC3
MDC3
SC
Tract Lengths
LG
5 minutes
10 minutes
15 minutes
20 minutes

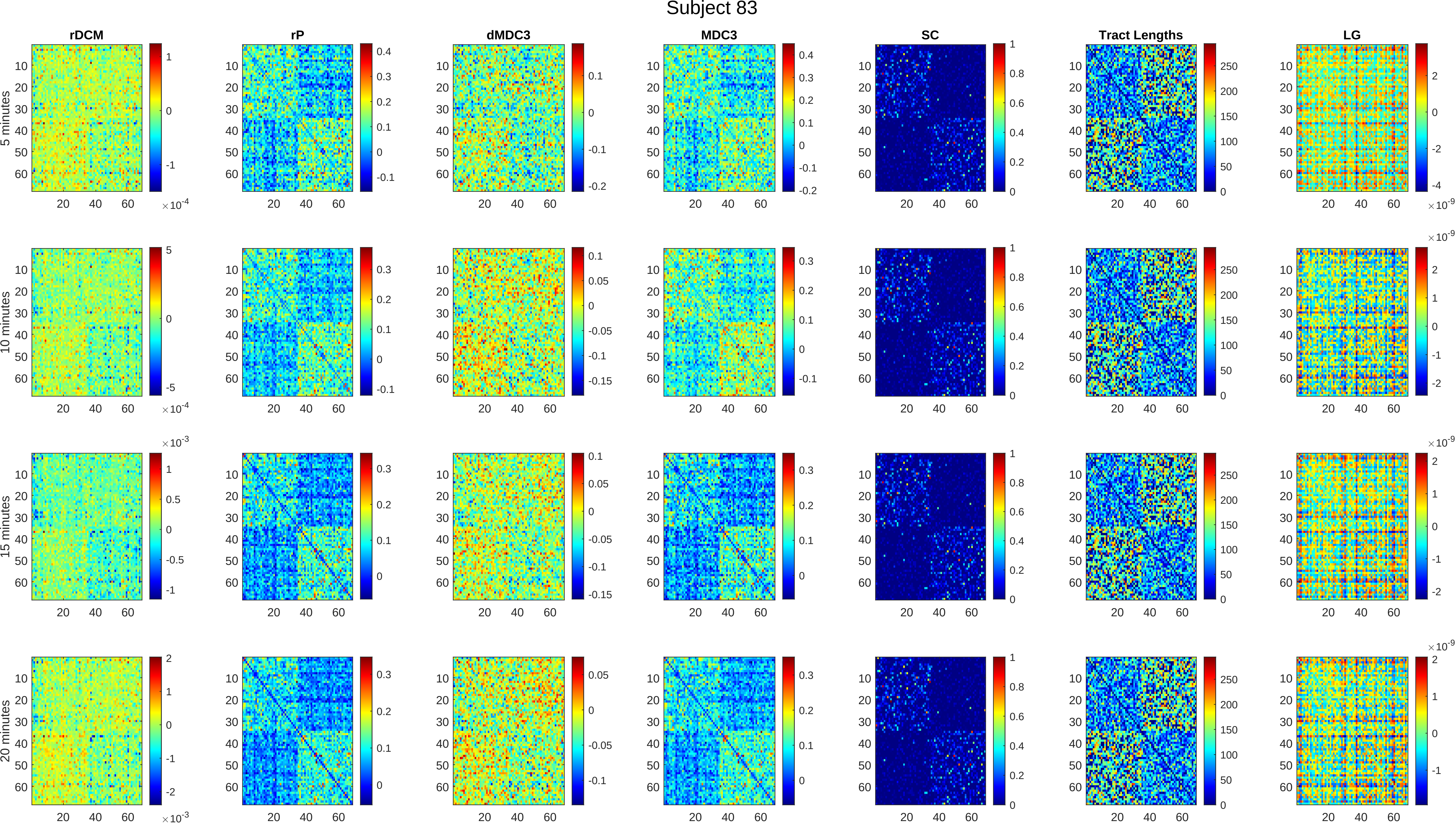
Subject 83
rDCM
rP
dMDC3
MDC3
SC
Tract Lengths
LG
5 minutes
10 minutes
15 minutes
20 minutes

Subject 84

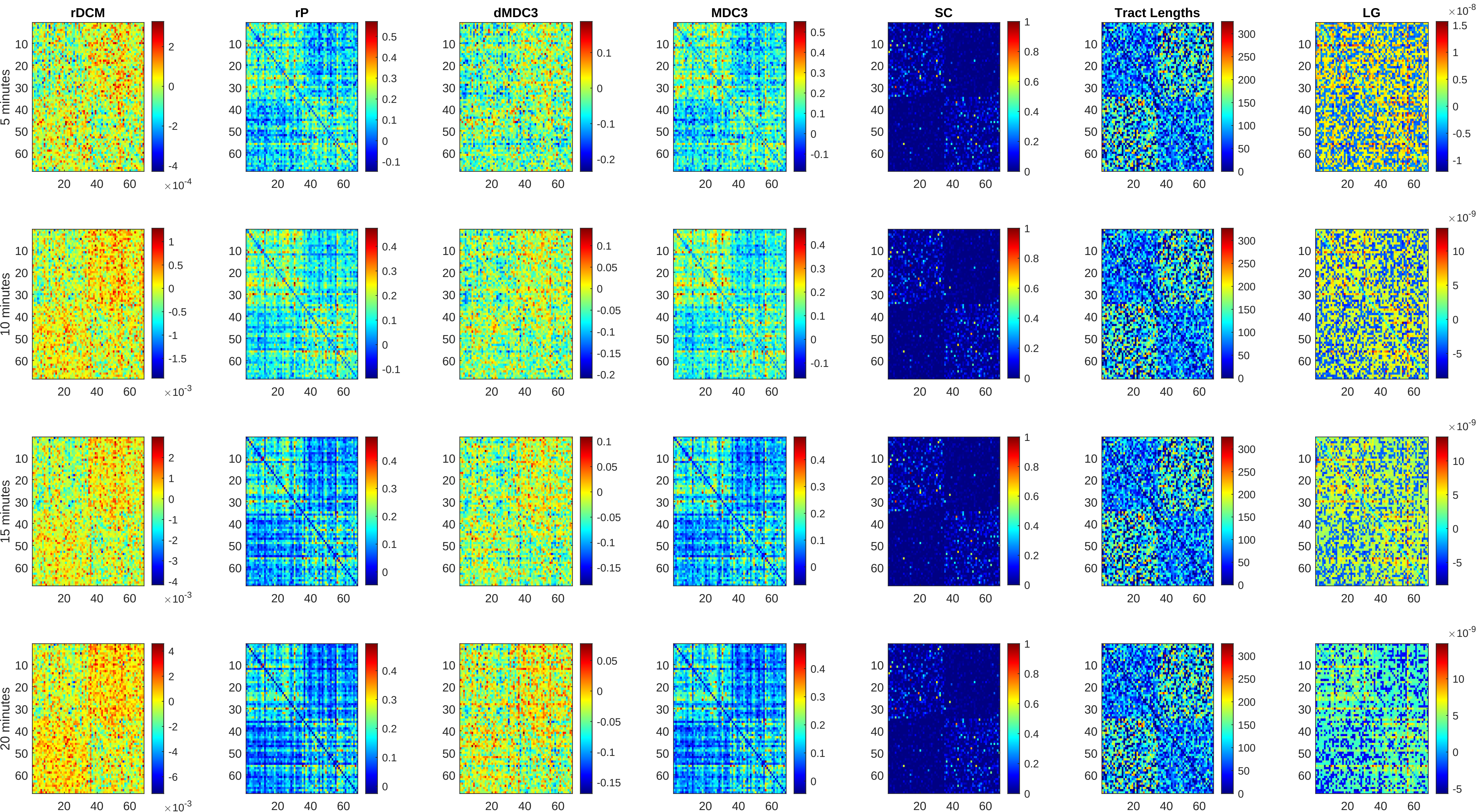

rDCM
rP
dMDC3
MDC3
SC
Tract Lengths
LG
5 minutes
10 minutes
15 minutes
20 minutes

Subject 85

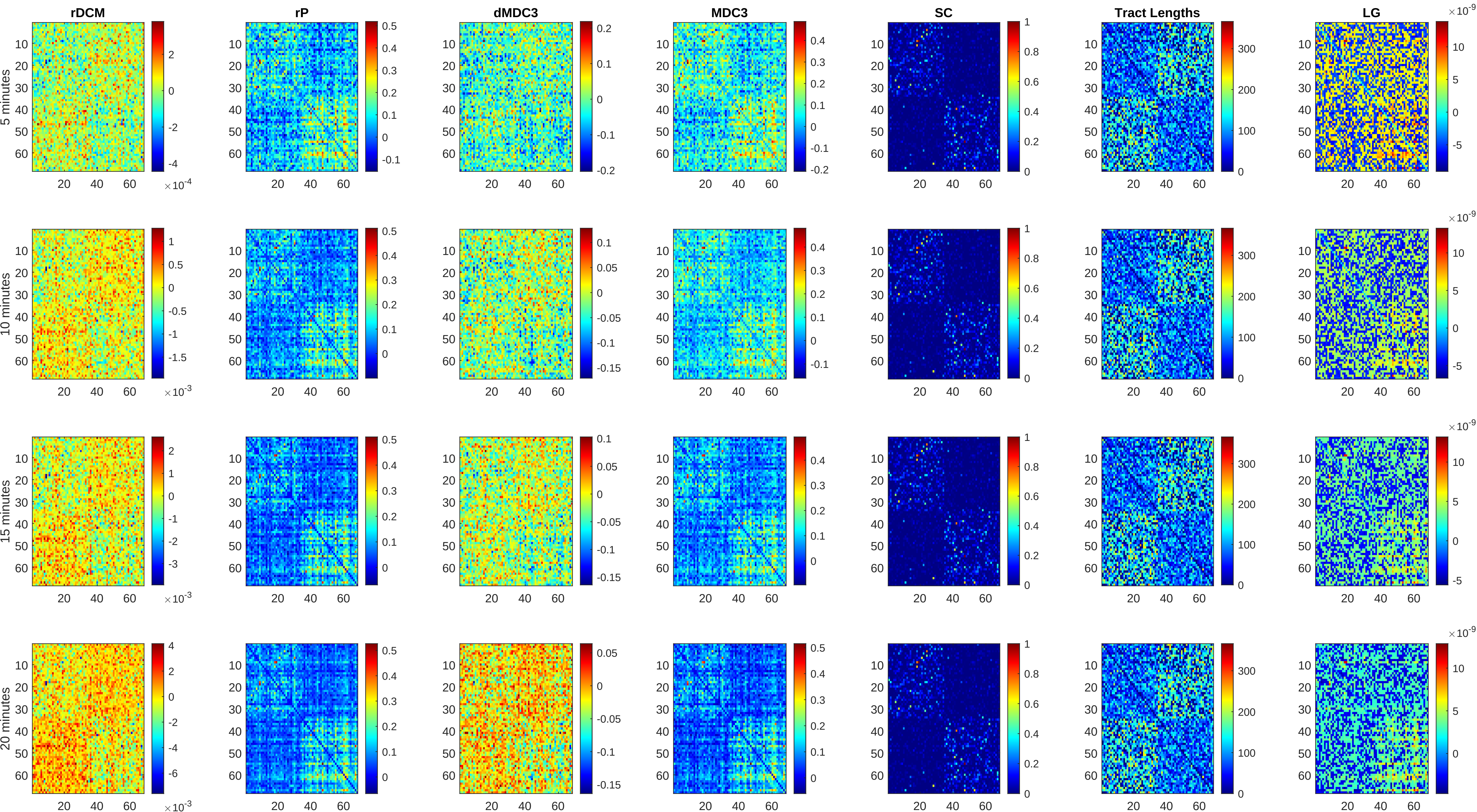
rDCM
rP
dMDC3
MDC3
SC
Tract Lengths
LG
5 minutes
10 minutes
15 minutes
20 minutes

Subject 86

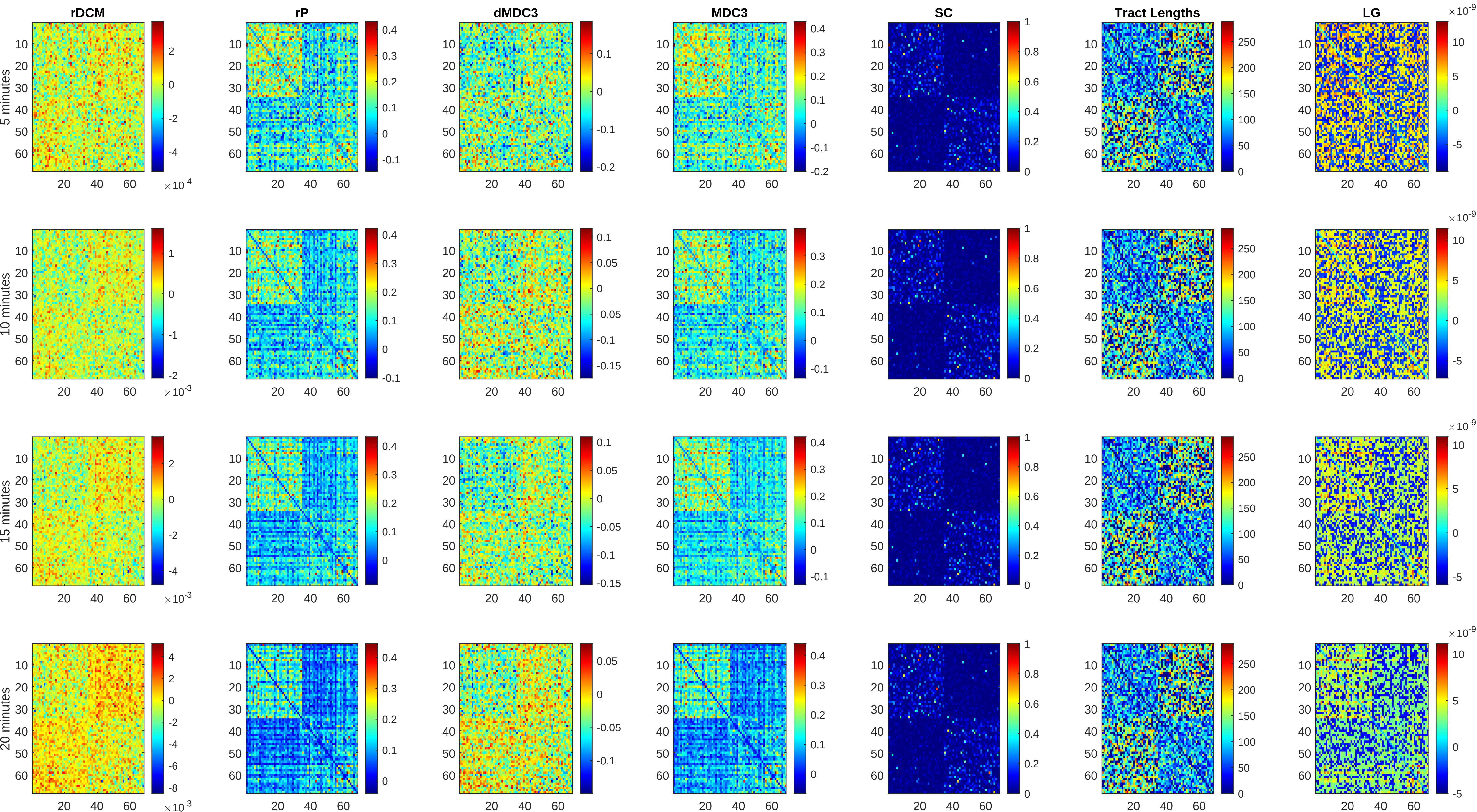

rDCM
rP
dMDC3
MDC3
SC
Tract Lengths
LG
5 minutes
10 minutes
15 minutes
20 minutes

Subject 87

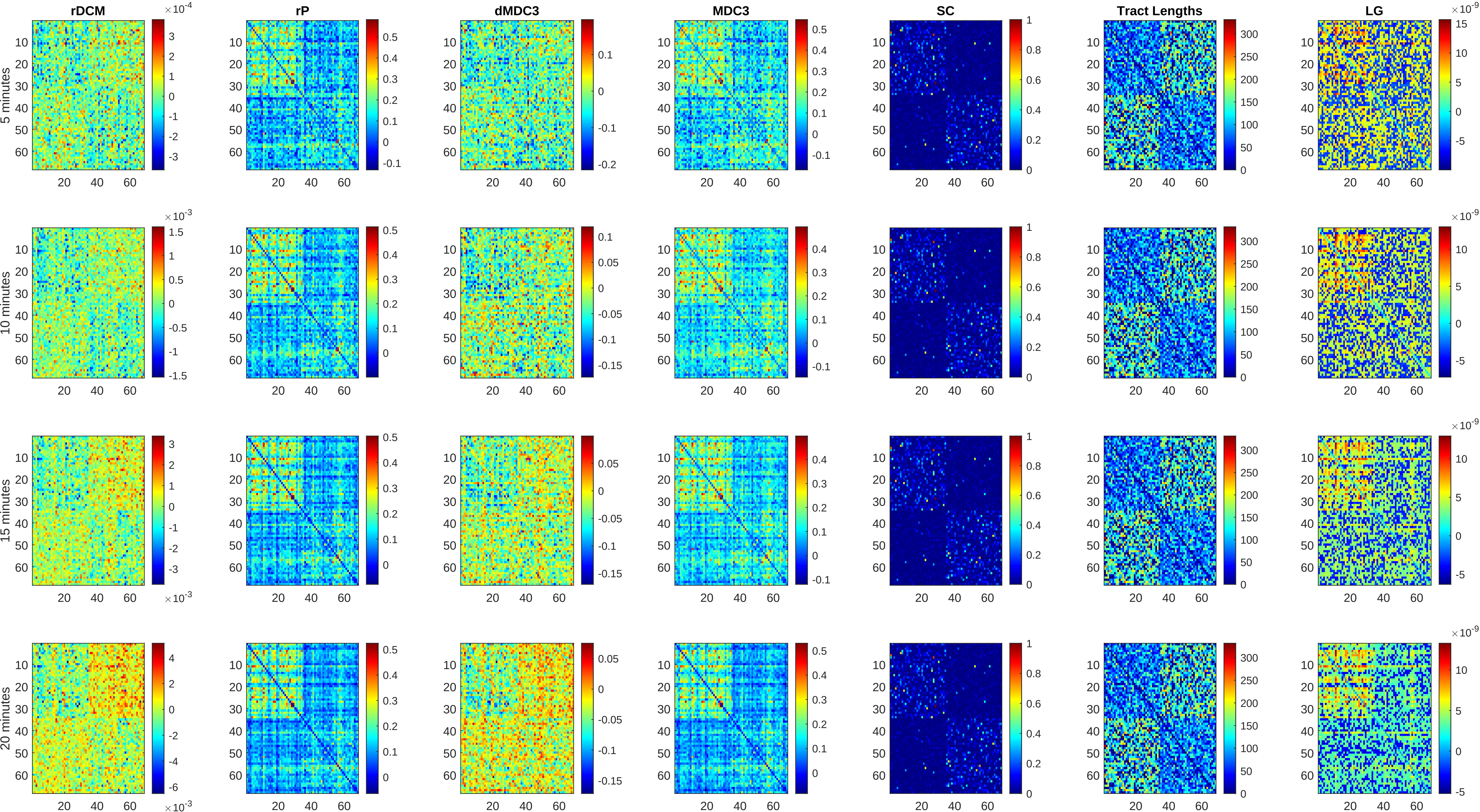

rDCM
rP
dMDC3
MDC3
SC
Tract Lengths
LG
5 minutes
10 minutes
15 minutes
20 minutes

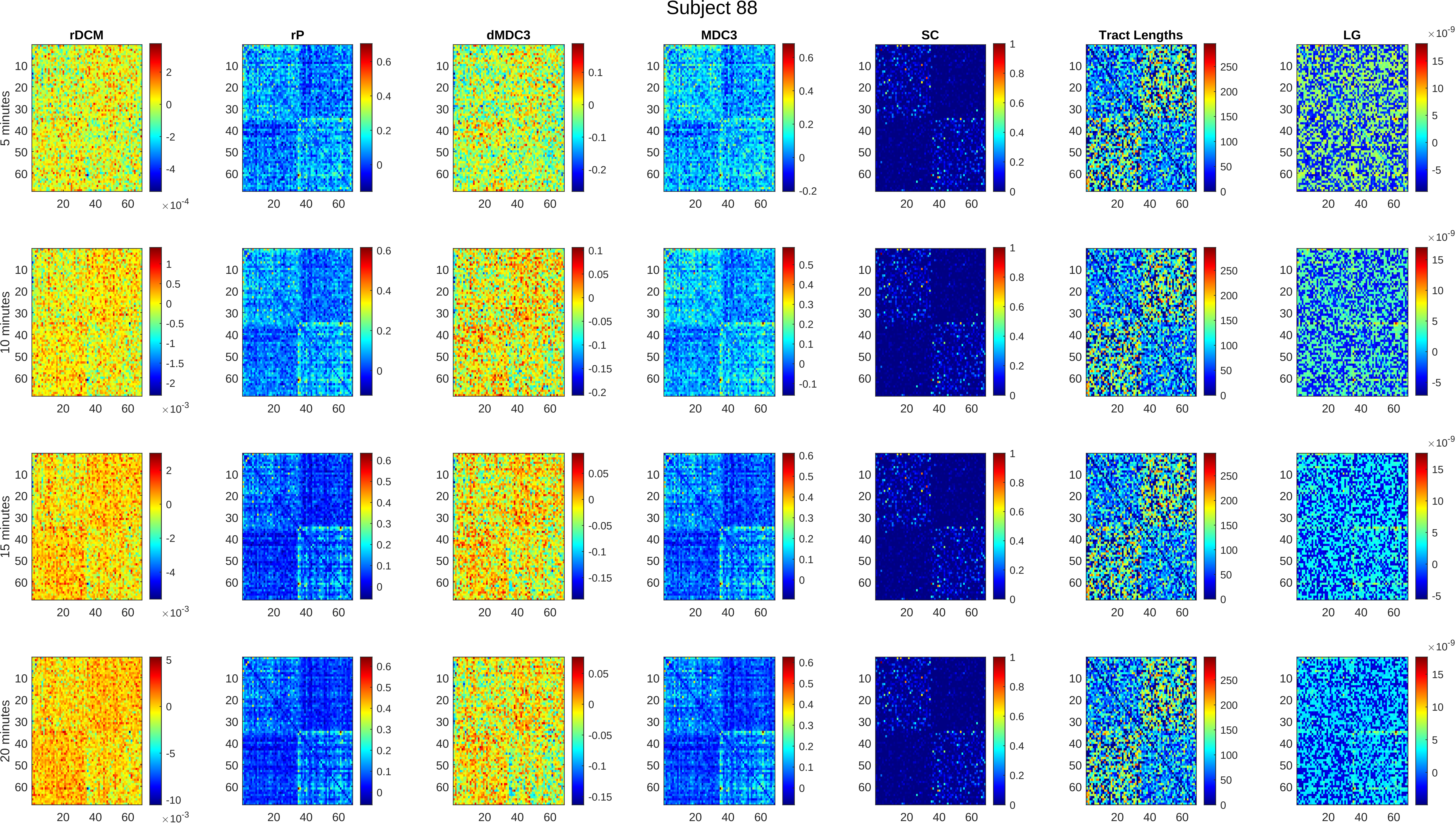
Subject 88
rDCM
rP
dMDC3
MDC3
SC
Tract Lengths
LG
5 minutes
10 minutes
15 minutes
20 minutes

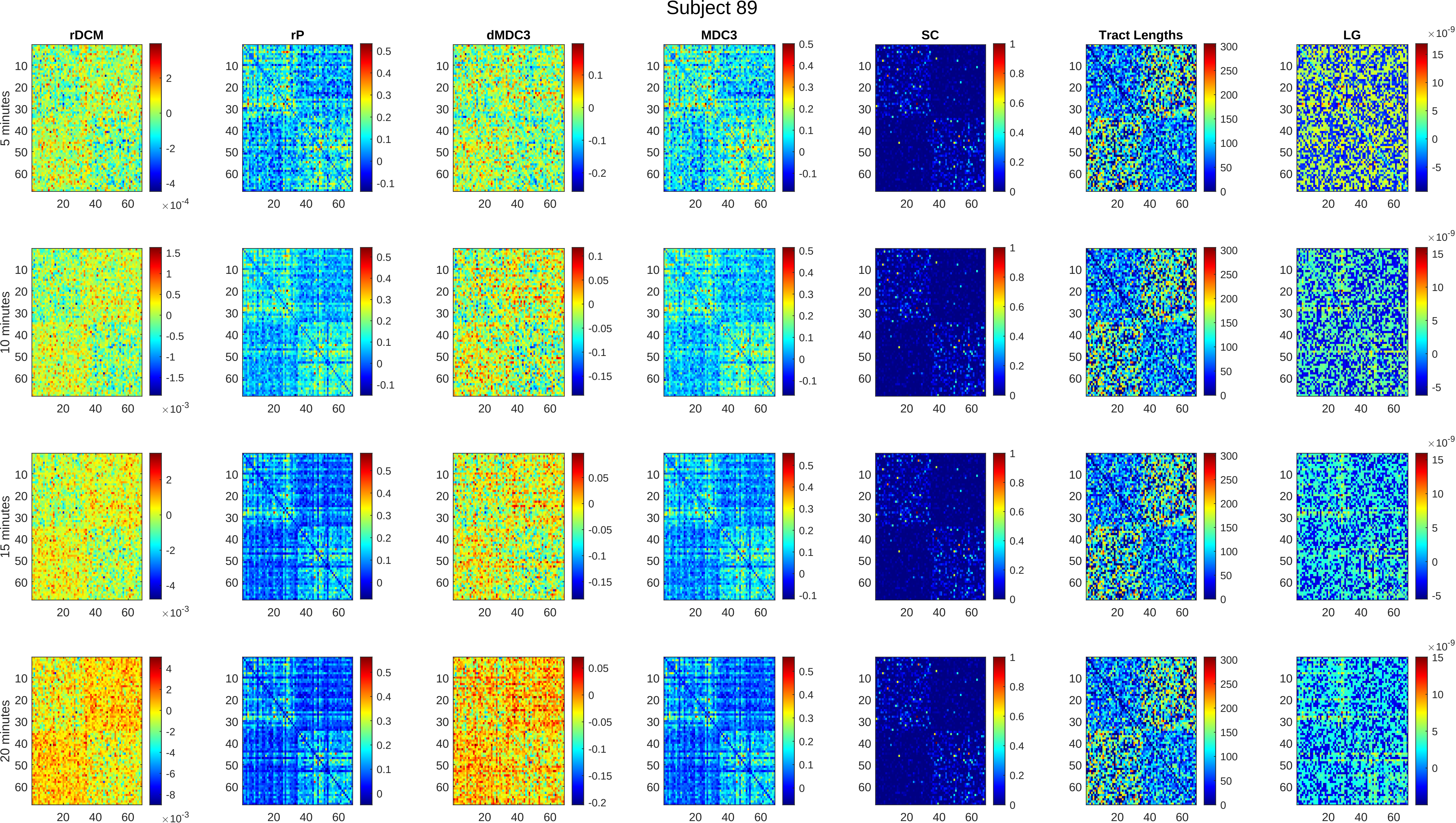
Subject 89
rDCM
rP
dMDC3
MDC3
SC
Tract Lengths
LG
5 minutes
10 minutes
15 minutes
20 minutes

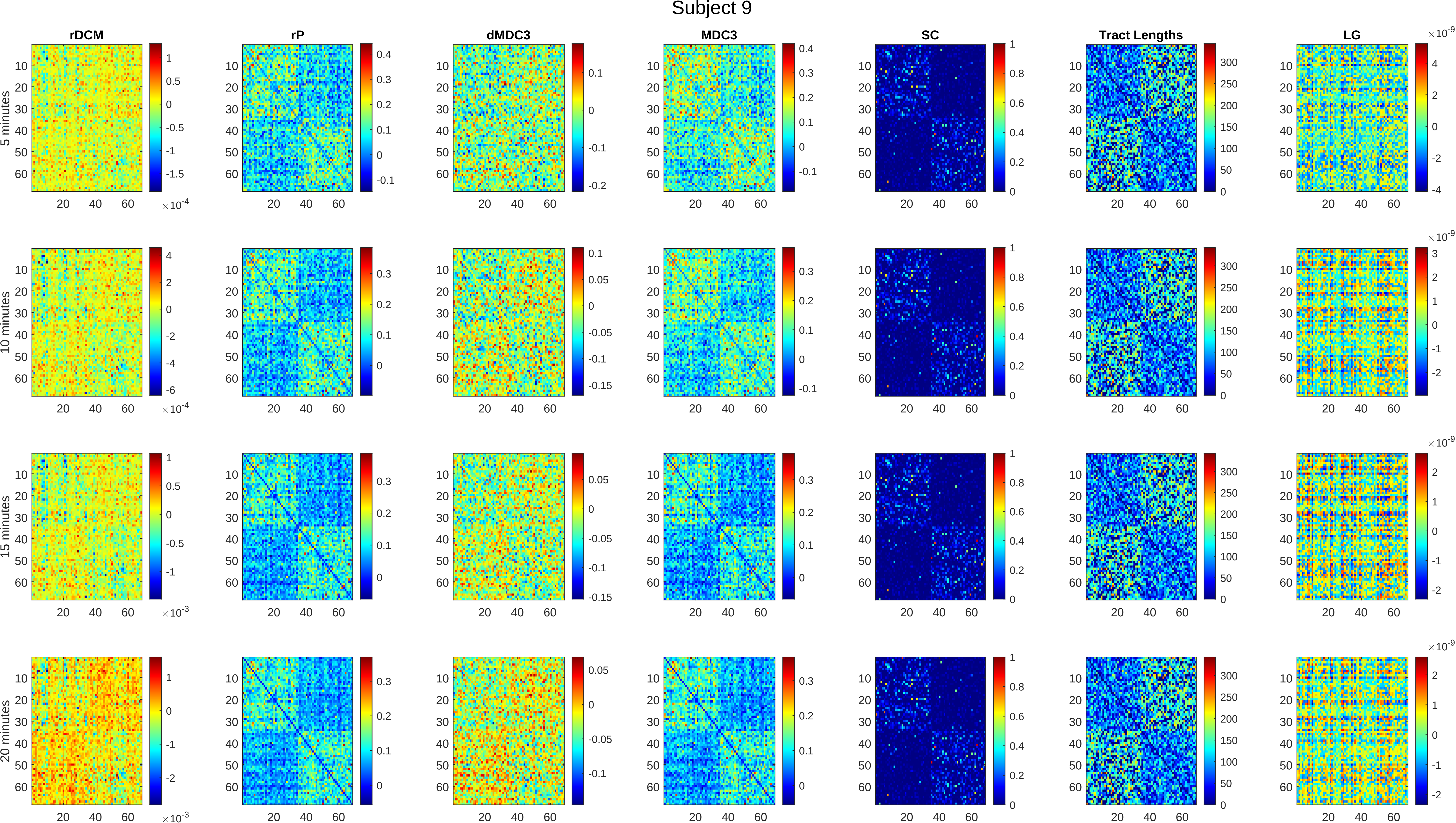

Subject 9
rDCM
rP
dMDC3
MDC3
SC
Tract Lengths
LG
5 minutes
10 minutes
15 minutes
20 minutes

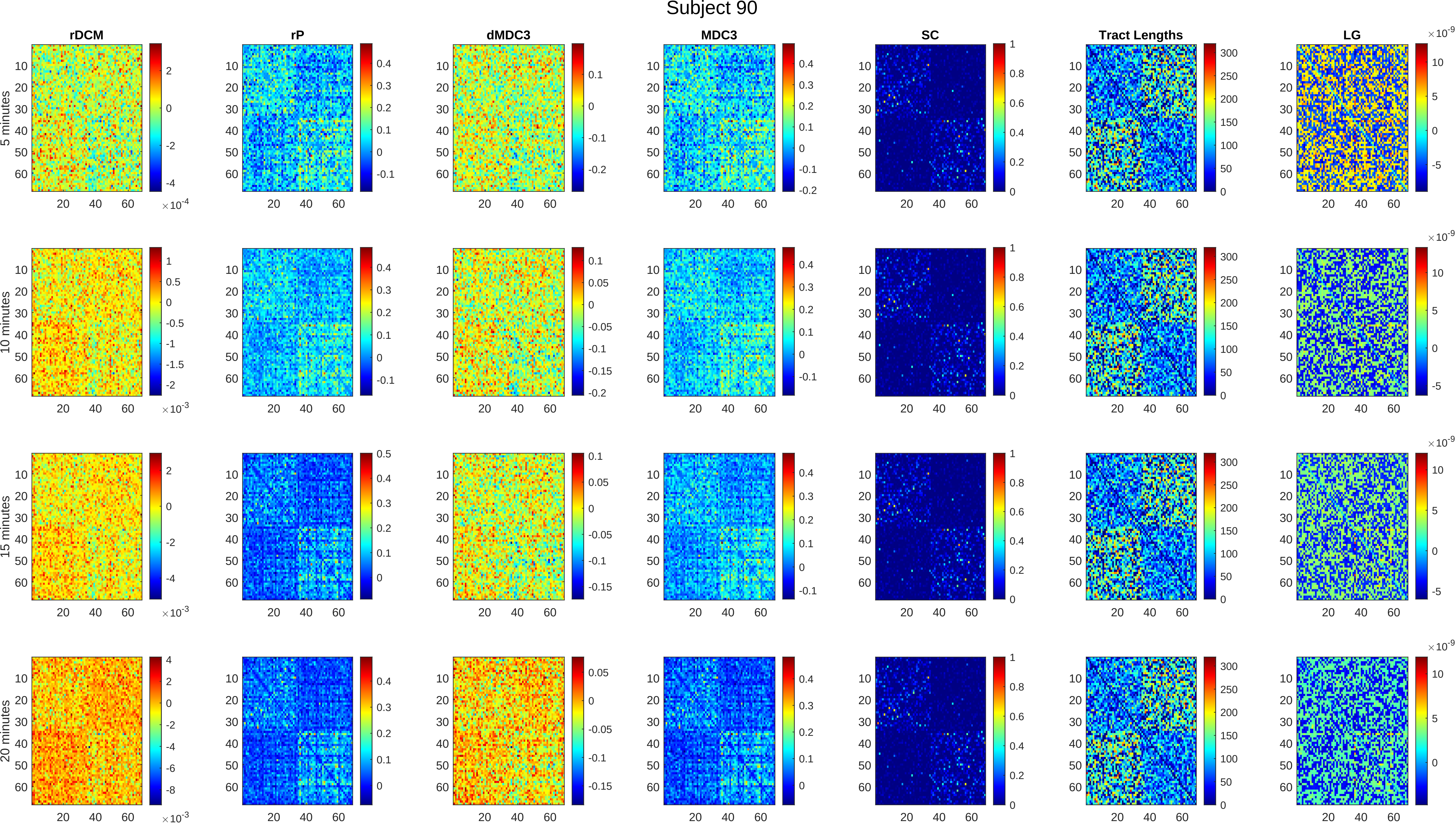

Subject 90
rDCM
rP
dMDC3
MDC3
SC
Tract Lengths
LG
5 minutes
10 minutes
15 minutes
20 minutes

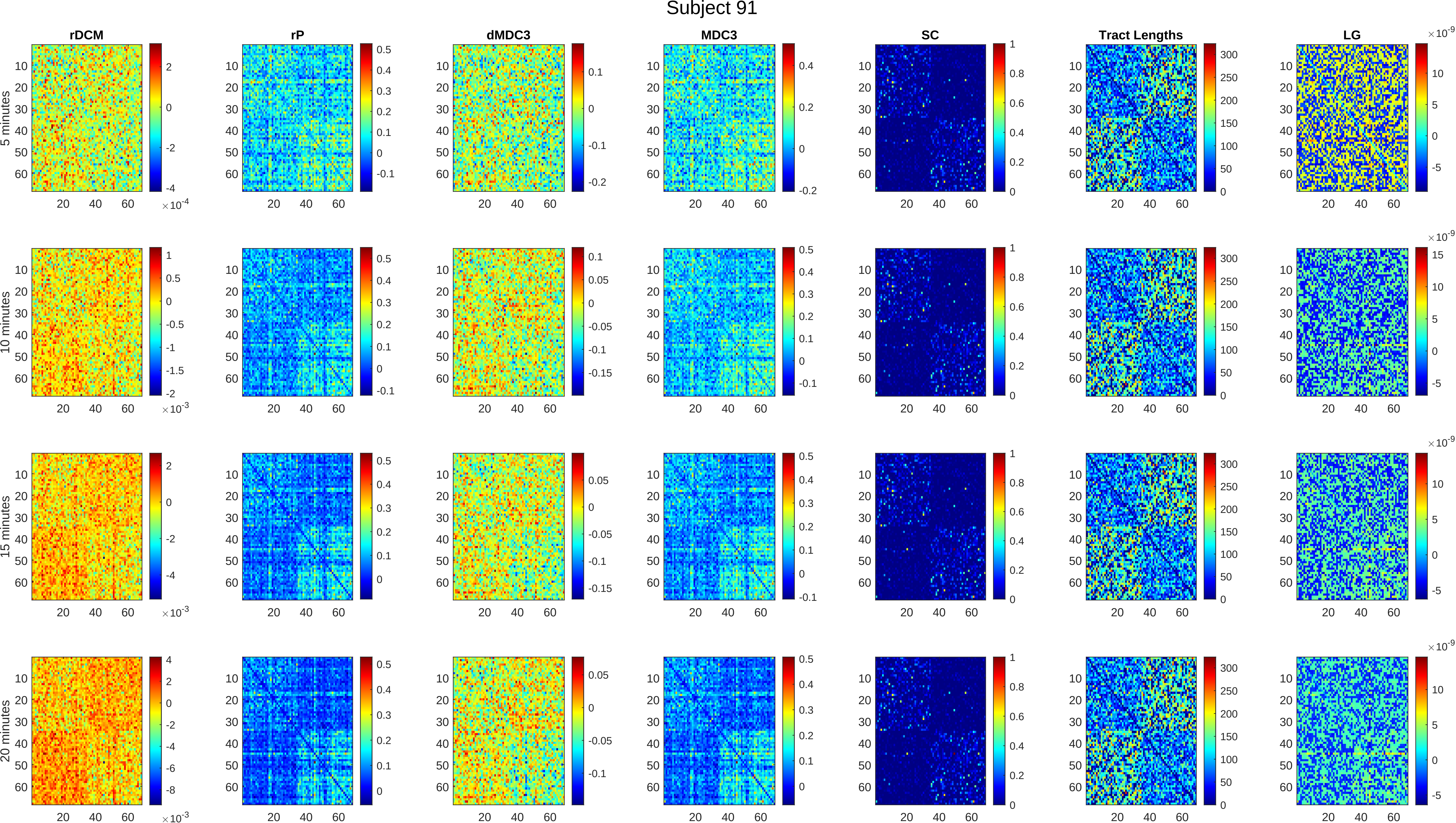

Subject 91
rDCM
rP
dMDC3
MDC3
SC
Tract Lengths
LG
5 minutes
10 minutes
15 minutes
20 minutes

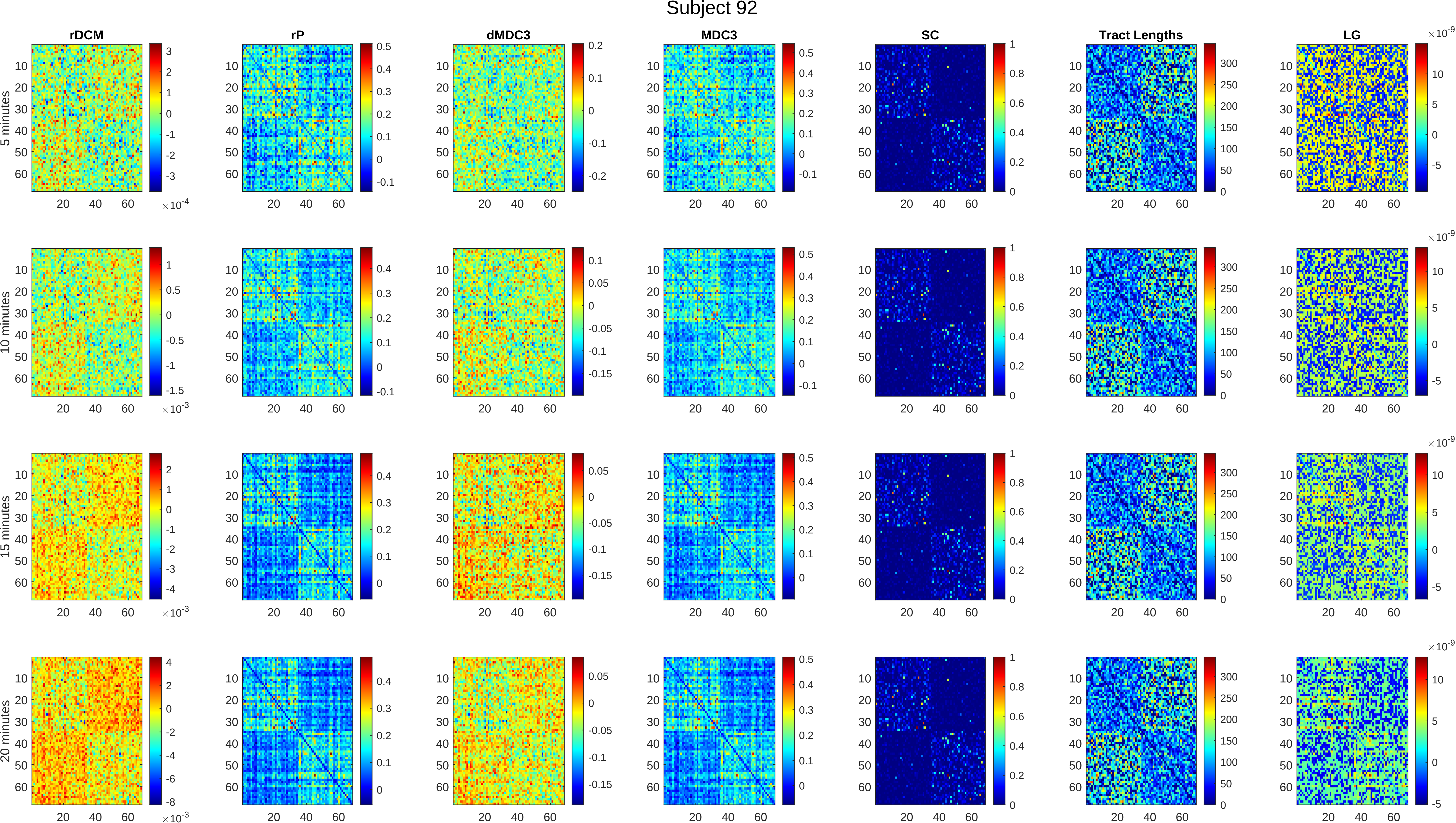
Subject 92
rDCM
rP
dMDC3
MDC3
SC
Tract Lengths
LG
5 minutes
10 minutes
15 minutes
20 minutes
×10^-4
×10^-3
×10^-3
×10^-3
×10^-9
×10^-9
×10^-9
×10^-9

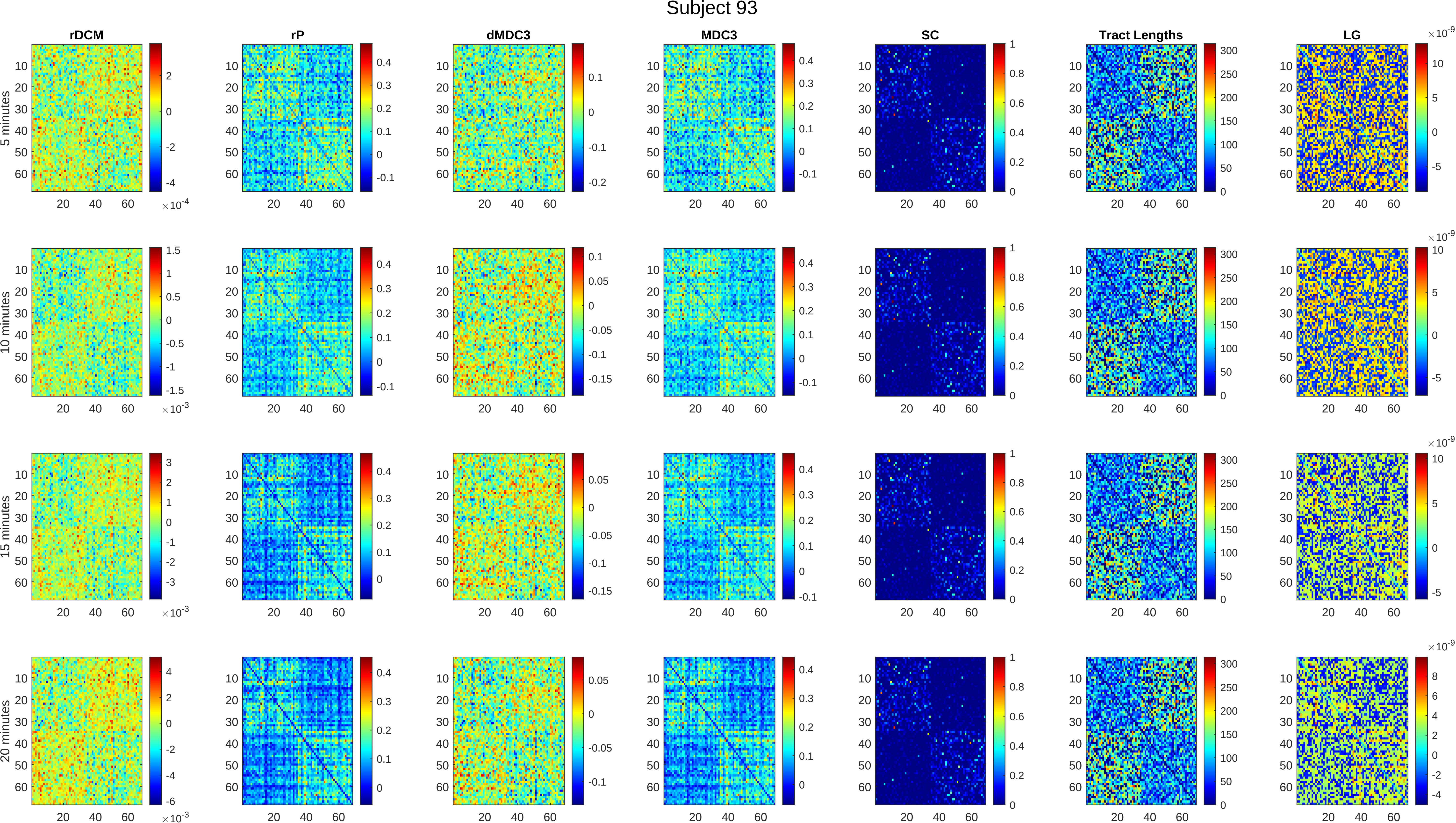

Subject 93
rDCM
rP
dMDC3
MDC3
SC
Tract Lengths
LG
5 minutes
10 minutes
15 minutes
20 minutes

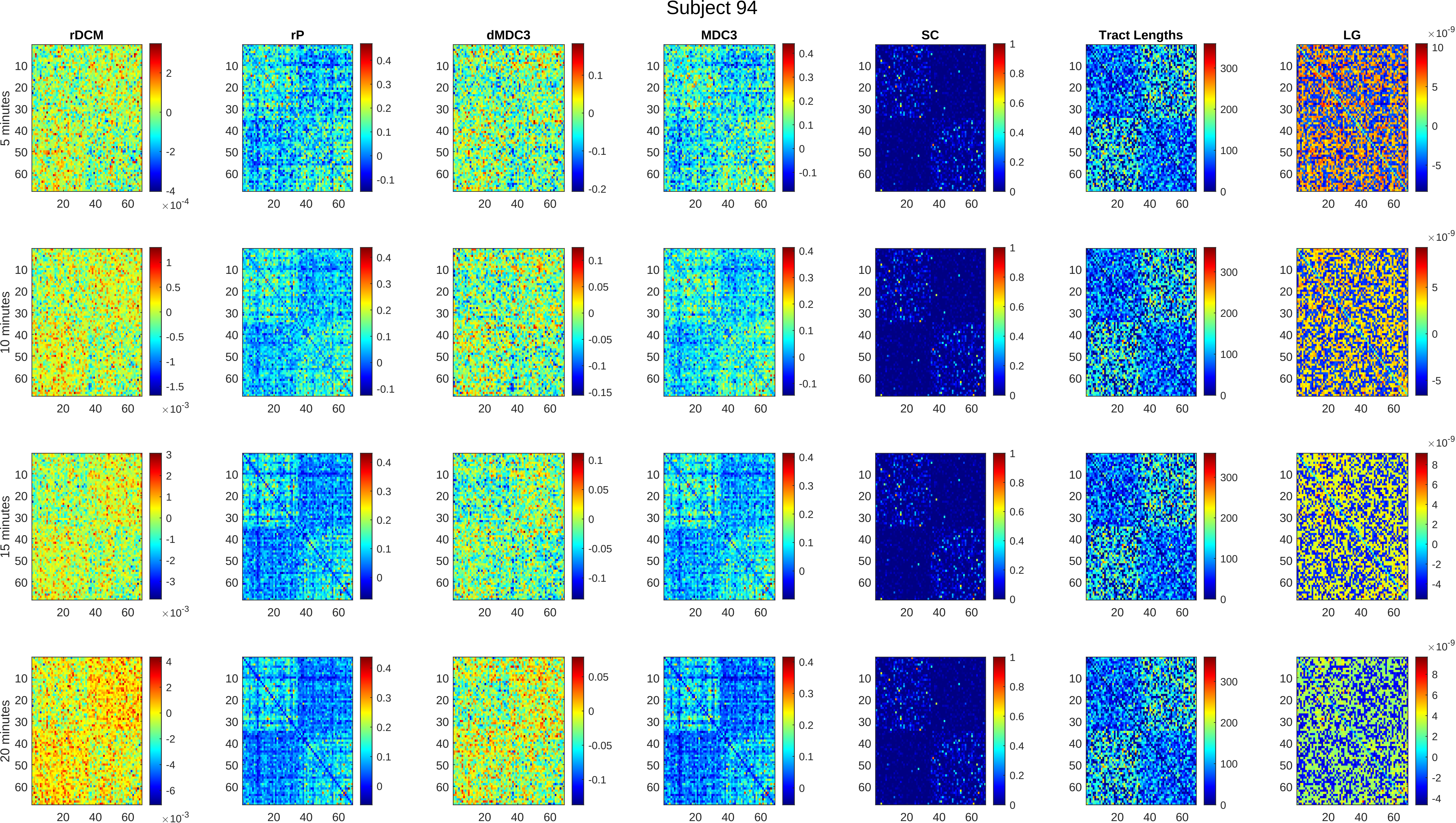
Subject 94
rDCM
rP
dMDC3
MDC3
SC
Tract Lengths
LG
5 minutes
10 minutes
15 minutes
20 minutes

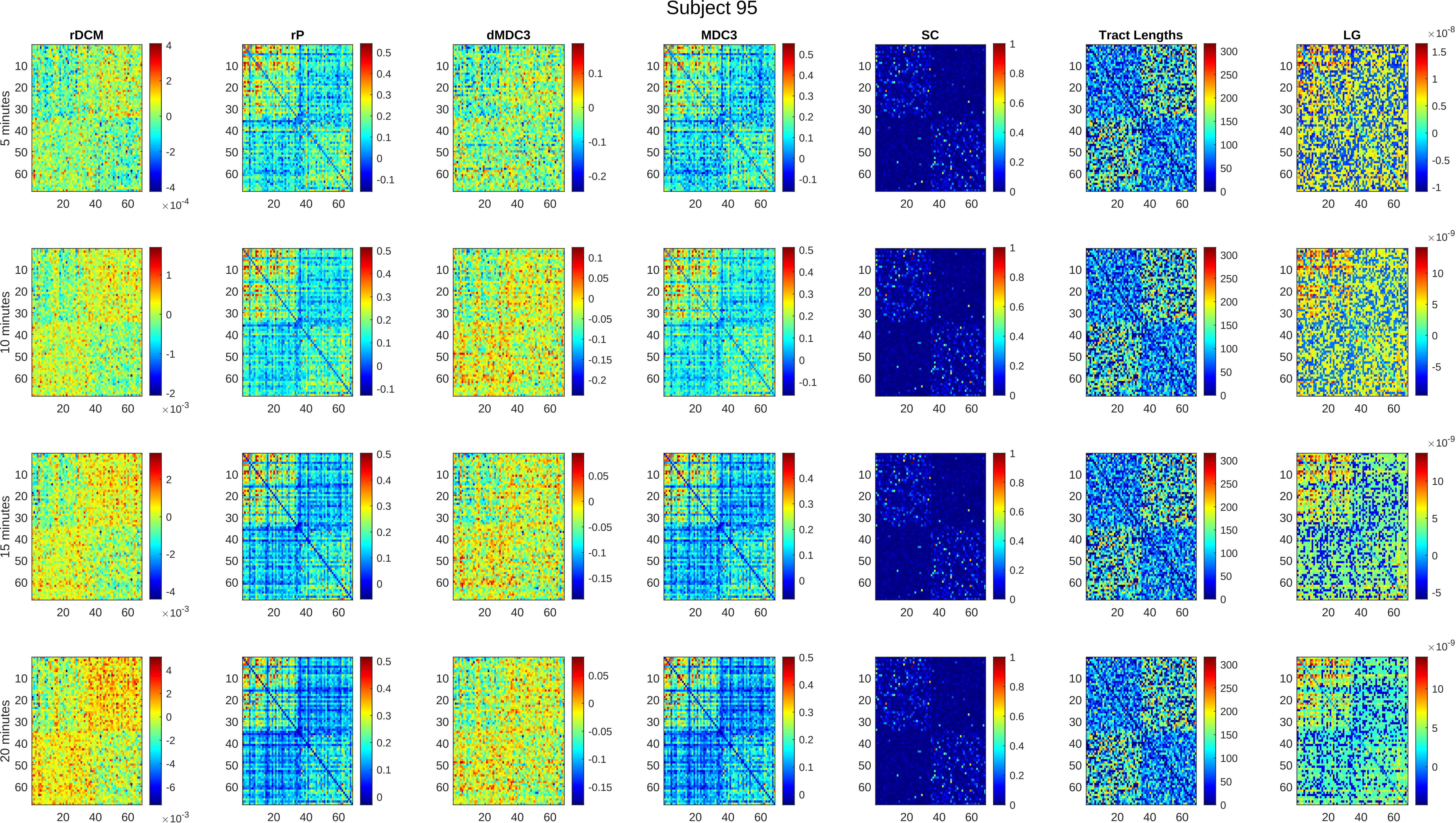
Subject 95
rDCM
rP
dMDC3
MDC3
SC
Tract Lengths
LG
5 minutes
10 minutes
15 minutes
20 minutes

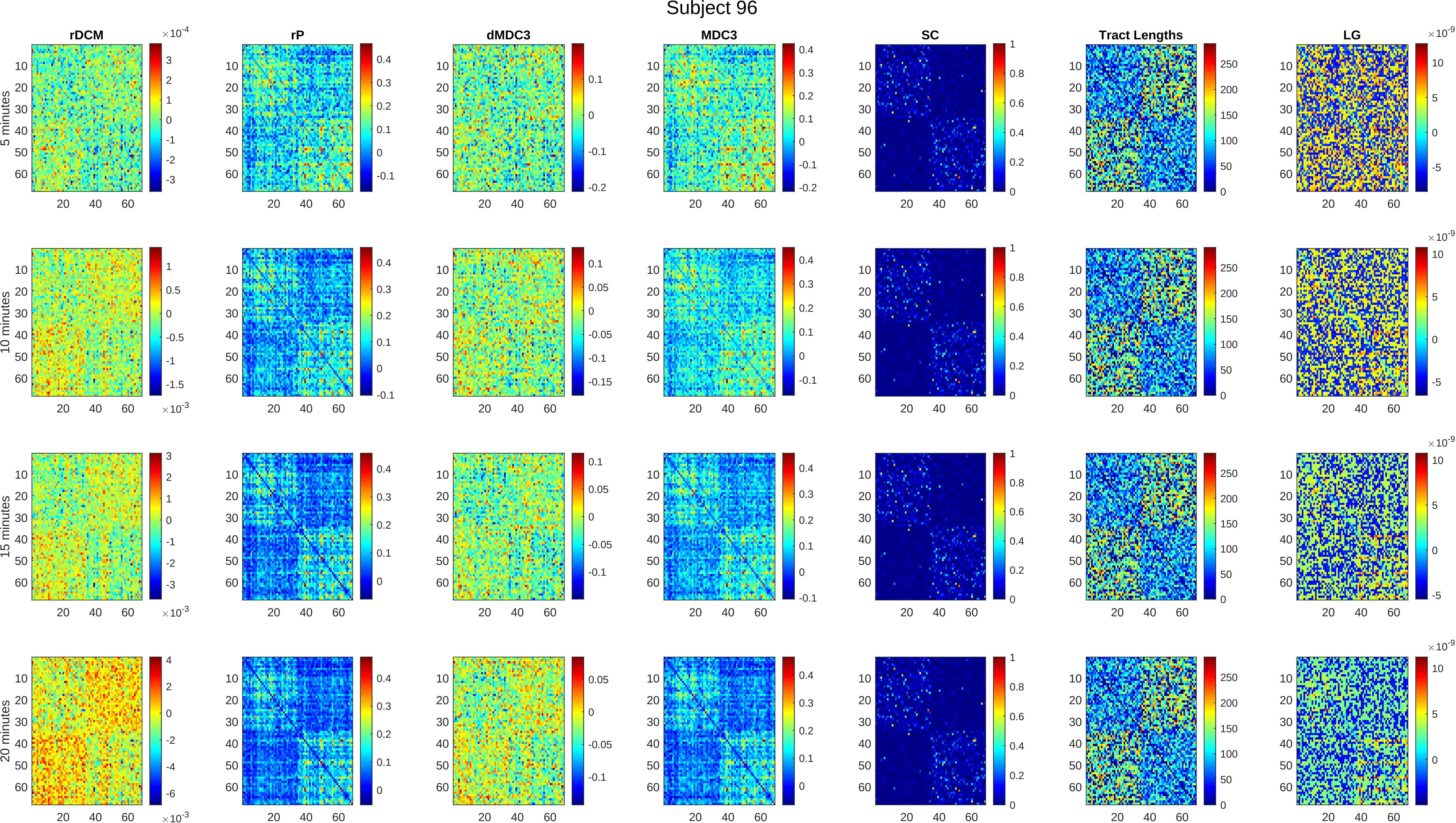

Subject 96
rDCM
rP
dMDC3
MDC3
SC
Tract Lengths
LG
5 minutes
10 minutes
15 minutes
20 minutes

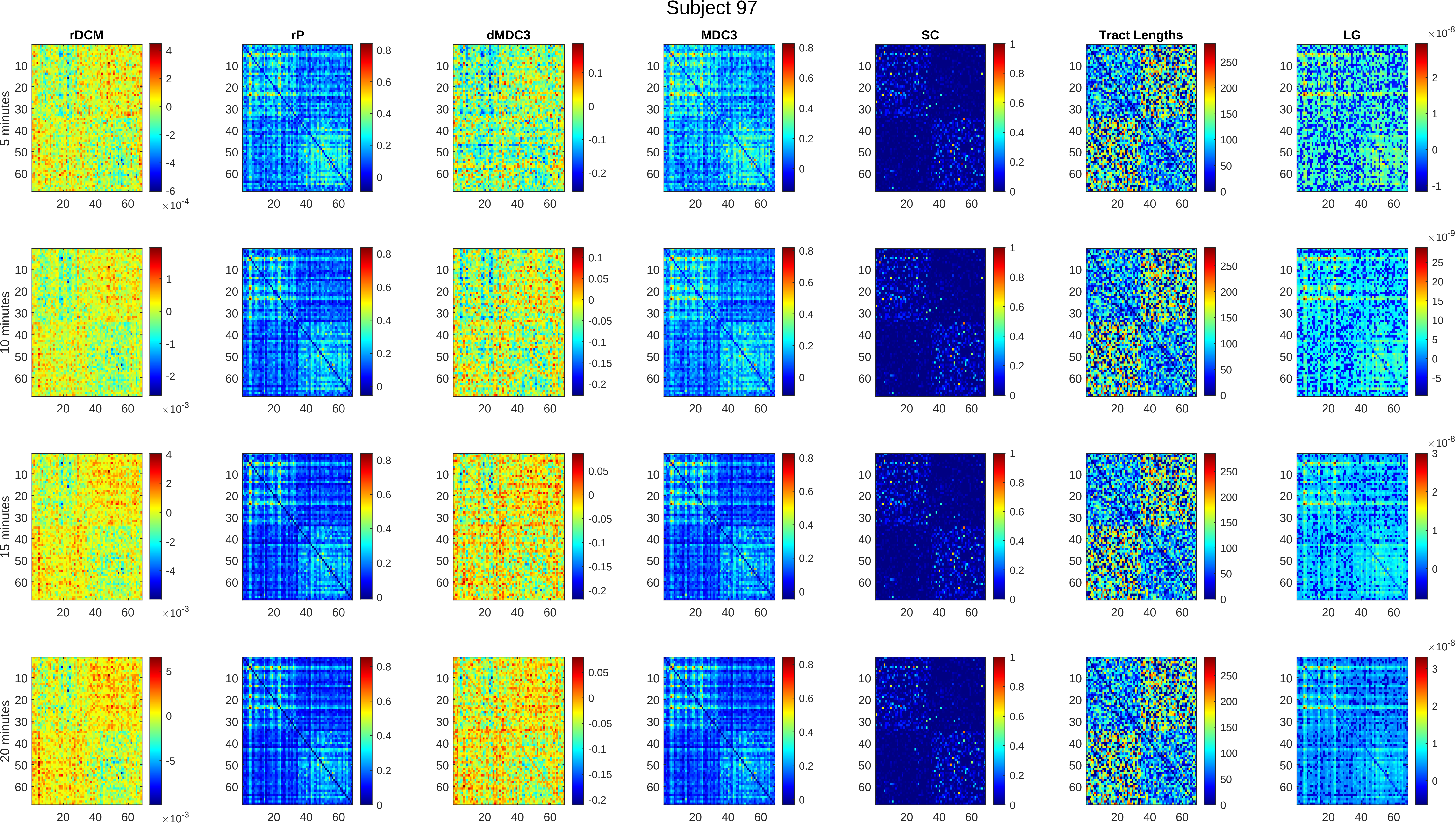
Subject 97
rDCM
rP
dMDC3
MDC3
SC
Tract Lengths
LG
5 minutes
10 minutes
15 minutes
20 minutes

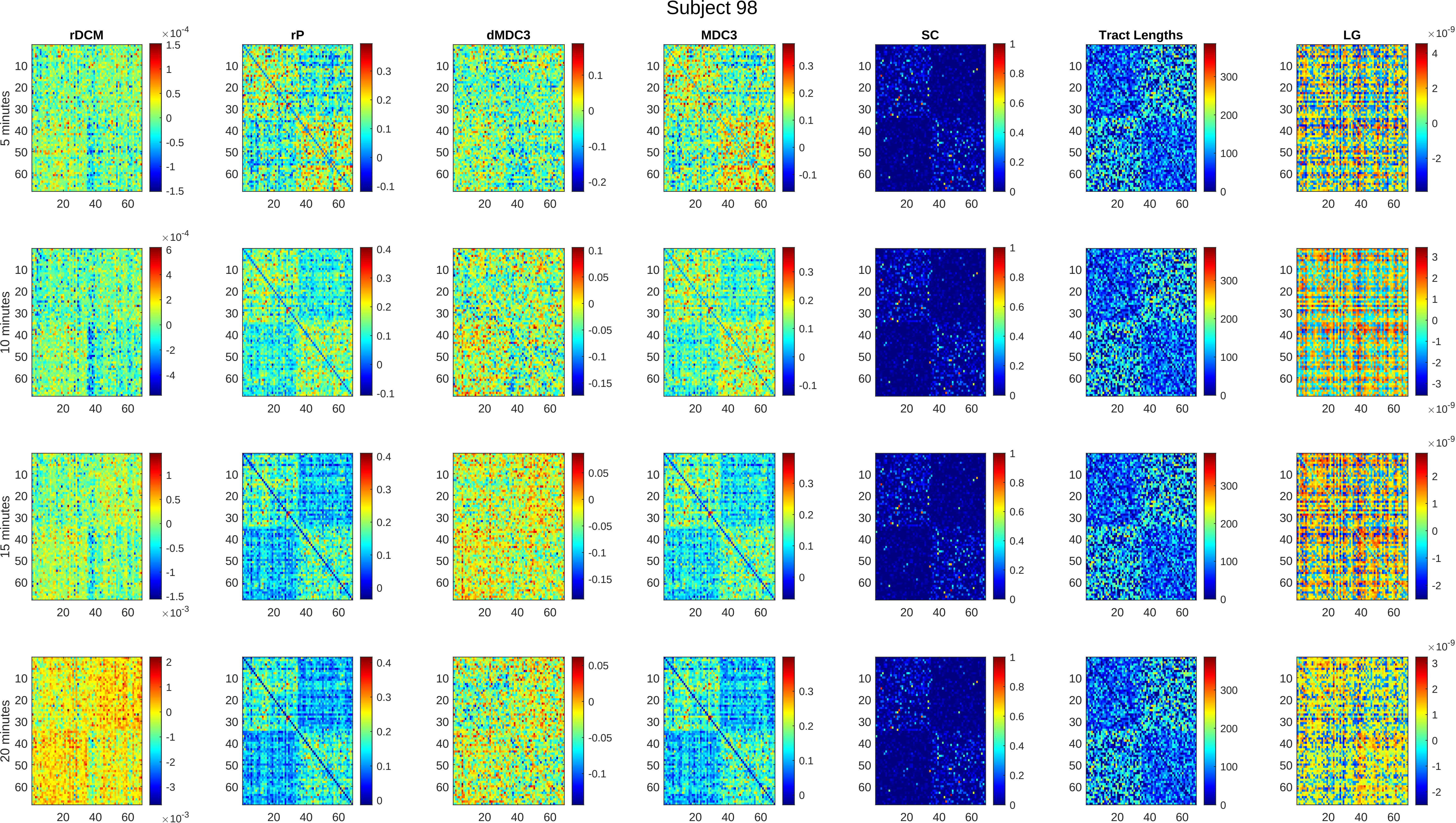

Subject 98
rDCM
rP
dMDC3
MDC3
SC
Tract Lengths
LG
5 minutes
10 minutes
15 minutes
20 minutes

Subject 99

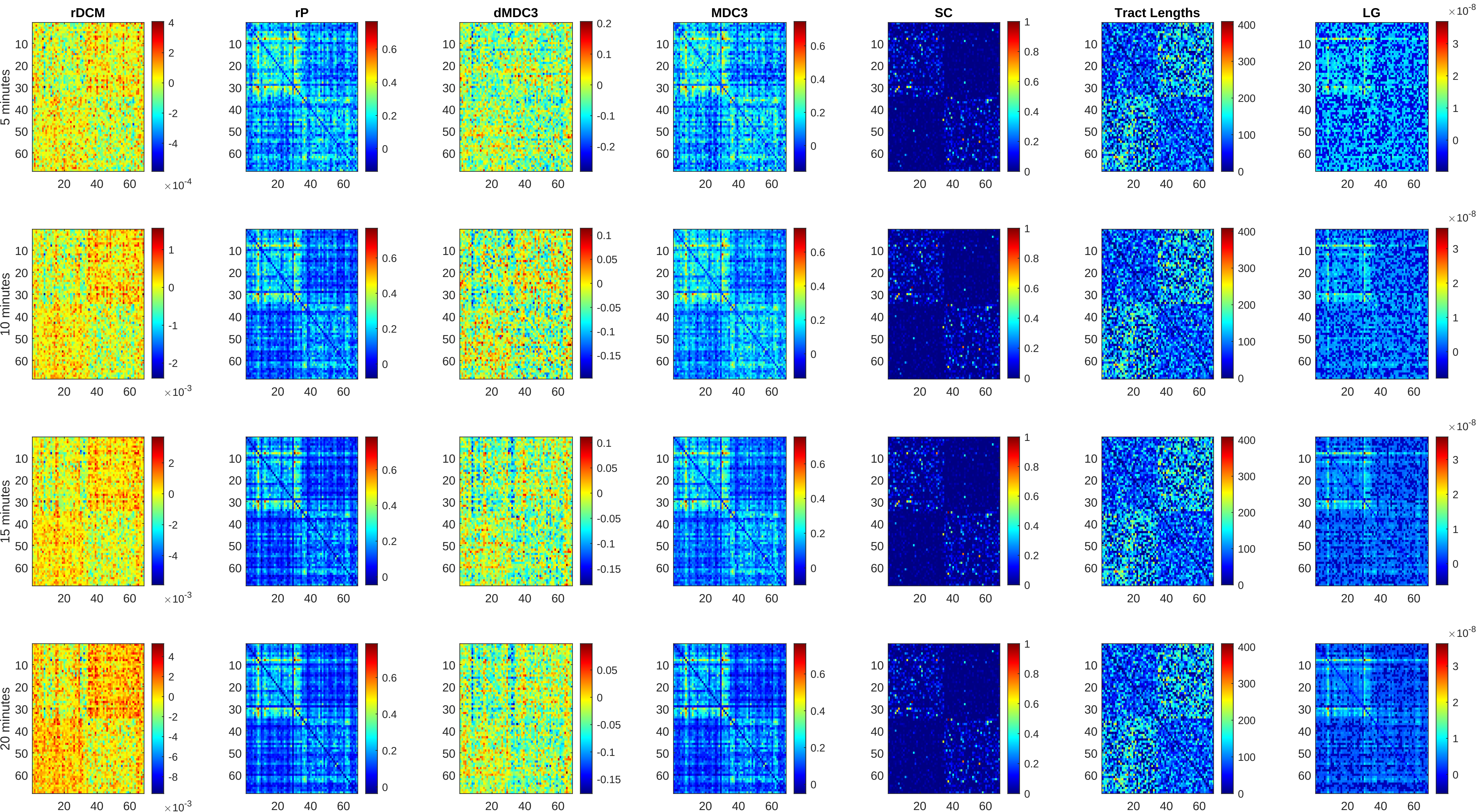

rDCM
rP
dMDC3
MDC3
SC
Tract Lengths
LG
5 minutes
10 minutes
15 minutes
20 minutes
